# Metal-Dependent Coordination Geometry Controls Autolytic Amide Bond Cleavage to Enable Prodrug Activation

**DOI:** 10.1021/jacs.6c15432

**Published:** 2026-09-14

**Authors:** Zhuoran Zhong, Dariusz Ṡmiłowicz, M. Andrey Joaqui-Joaqui, Phuong Nguyen Tran, Leah C. Garman, Ilia A. Guzei, Eszter Boros

**Affiliations:** Department of Chemistry, 5228 University of Wisconsin-Madison, Madison, Wisconsin 53706, United States

## Abstract

We introduce a peptide prodrug activation strategy that employs the trivalent Lewis-acidic metal ions Ga^3+^, Fe^3+^, In^3+^, and Co^3+^, chelated by triazacyclononanediacetic acid monoamides. The metal chelates trigger rate-tunable, metal-mediated, autolytic amide bond cleavage (TMAC) adjacent to *N*-methylated and canonical amino acids. Our studies reveal that the four TMAC-compatible metal ions induce amide bond hydrolysis with divergent pH dependence and mechanistic pathways. We subsequently linked Ga^3+^, Fe^3+^, In^3+^, and Co^3+^ complexes to the N-terminus of a potent cytotoxic cargo, monomethyl auristatin E (MMAE), to produce the proof-of-concept prodrug PD1. In vitro toxicity studies establish IC_50_ values in HeLa and PC-3 cells that broadly correlate with complex cleavage rates; the metal ion-free PD1 conjugate, the Zn-PD1 complex, as well as the Ga^3+^, Fe^3+^, In^3+^, and Co^3+^ chelate hydrolysis products alone confer no toxicity. Attachment of a cancer-targeting peptide to the metal chelate, furnishing PD2, enables targeted delivery of the therapeutic payload in vivo, compatible with noninvasive nuclear imaging using positron emission tomography with ^68^Ga or single-photon computed tomography with ^111^In. The corresponding ^nat^Ga-PD2 complex selectively suppressed tumor growth in vivo with minimal off-target toxicity. These studies establish metal identity as a general design parameter for programming amide bond cleavage and introduce TMAC as a versatile platform to engender peptide prodrugs with tunable release kinetics.

## Graphical abstract

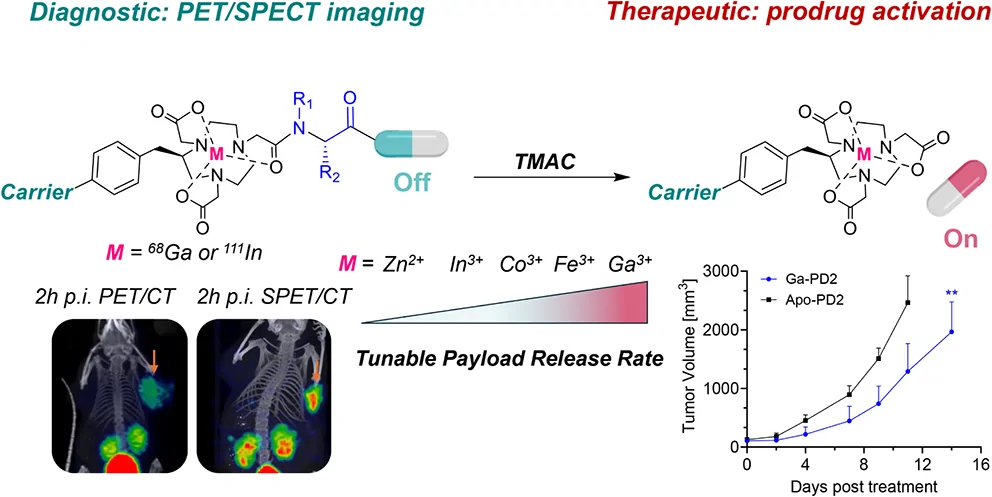

## Introduction

To expand the application and utility of peptide-based diagnostics and therapeutics, innovative chemical strategies are required that enable precise control over molecular behavior in vivo.^1^ This includes the ability to finely tune pharmacokinetics, such as circulation time, tissue penetration, and clearance rates, to match the biological half-lives and targeting profiles of various disease biomarkers.^2–6^ Additionally, next-generation constructs must support molecular design with efficient, stimulus-responsive release mechanisms that ensure payload delivery specifically at the disease site, minimizing off-target toxicity.^7–9^

Enzymatically cleavable linkers, relying on short 2–4 amino acid peptide sequences or ester bonds that are readily recognized by cathepsins, matrix metalloproteases, or esterases, are integral components of FDA-approved antibody-drug conjugates.^10–12^ Enzymatically cleavable linkers depend on efficient binding and recognition of the cleavage site, which, if obstructed by bulky functional groups, can significantly impede release.^13–16^ Off-target tissue expression of cleavage enzymes may contribute to undesirable chemical reactivity and represent additional, dose-limiting factors (Figure 1). Similarly, pH-responsive and redox-triggered release have proven difficult to control and consequently produced moderate success in the clinic.^17–21^

**Figure 1:**
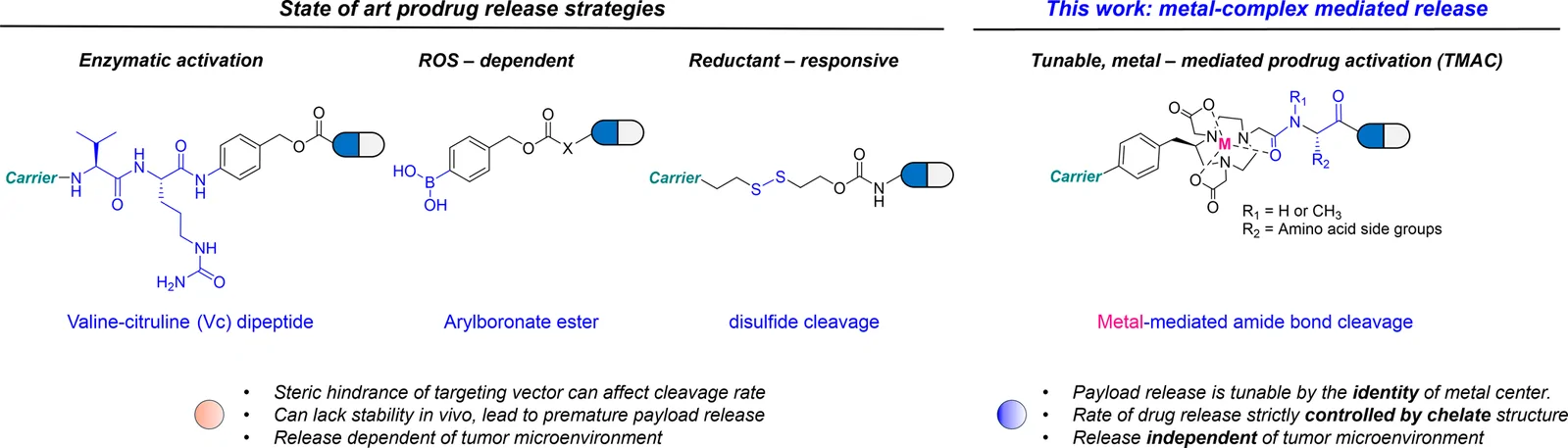
Summary of stimulus-responsive linkers. Conventional linker activation strategies rely on exogenous stimuli such as proteases or cellular ROS species, which can suffer from limited in vivo stability and off-target toxicity. In contrast, the TMAC platform utilizes the endogenous metal complex as a built-in trigger to mediate site-specific amide bond cleavage. The cleavage kinetics are readily tunable by the identity of adjacent amino acids or metal centers, which allows for precise spatiotemporal control of payload release.

Our group has previously introduced tunable, metal-mediated amide bond cleavage (TMAC) as a self-degradative strategy for controlled radiodiagnostic payload release to modulate the in vivo pharmacokinetics of the corresponding Ga-68-labeled, positron-emitting diagnostic tracer.^22^ In this approach, complexation with Ga^3+^ induces conformationally controlled amide bond cleavage that is readily tunable in rate by the nature of the complex-adjacent amino acid. However, the strict peptide-sequence dependence limits the applicability to peptide-based prodrug activation.

Here, we explore modulation of the identity of the metal ion to tune the reactivity profile of the corresponding TMAC-compatible conjugate. We identify the scope of trivalent transition-metal ions and metalloids that can induce TMAC. We employ mechanistic studies to determine the divergent reactivity patterns observed among the different metal ions. Finally, we probe the applicability of TMAC to release a clinically validated cytotoxic in vitro and in vivo.

## Results and Discussion

### Metal Ion Screening and Scope

Following our previous work, where we investigated the cleavage mechanism and scope of complex-adjacent amino acids, we subjected model tripeptide sequences capped at the N-terminus with the azamacrocyclic chelator 1,4,7-triazacyclononane-1,4,7-triacetic acid (NOTA) to chelation with a host of transition-metal ions compatible with the 6-coordinate chelator.^22^ After successful complexation and purification, the pH and reaction temperature of the metal complex solution were adjusted to pH 7.4 and 80 °C to initiate the cleavage. The cleavage process was monitored and quantified using high-performance liquid chromatography and mass spectrometry (HPLC-MS). Based on our prior investigation of the TMAC platform, which showed that noncanonical amino acid residues promote faster cleavage at pH 7.4, we selected glycine (Gly), *N*-methylglycine (^NMe^Gly), and *N*-methylserine (^NMe^Ser) for further investigation. We first assessed the cleavage activity of zinc (Zn^2+^) complexes as a non-reactive control; indeed, none of the Zn^2+^ complexes triggered amide bond hydrolysis, in accordance with our previous work (Figure 2B, Figures S52–S54). We next probed the reactivity profile of iron (Fe^3+^), cobalt (Co^3+^), and indium (In^3+^) complexes due to their known ability to form inert complexes with NOTA derivatives.^23–30^ Indeed, initial screening results indicated that all three metal ions could confer TMAC activity with canonical and *N*-methylated amino acids adjacent to the metal complex. In accordance with our prior findings with Ga^3+^, cleavage rates were accelerated for *N*-methyl amino acid derivatives (Figure 2B). However, we also found that cleavage rates with In^3+^, Co^3+^, and Fe^3+^ readily exceeded those obtained for the previously characterized Ga^3+^ complex, which did not correlate with the metal ion Lewis acidity or hardness.^31–33^ Examining pH dependence indicated that In^3+^, Co^3+^, and Fe^3+^ were subject to divergent reaction mechanisms when compared to the previously investigated Ga^3+^ complex (Figure 2E, Figures S55–S64).

**Figure 2:**
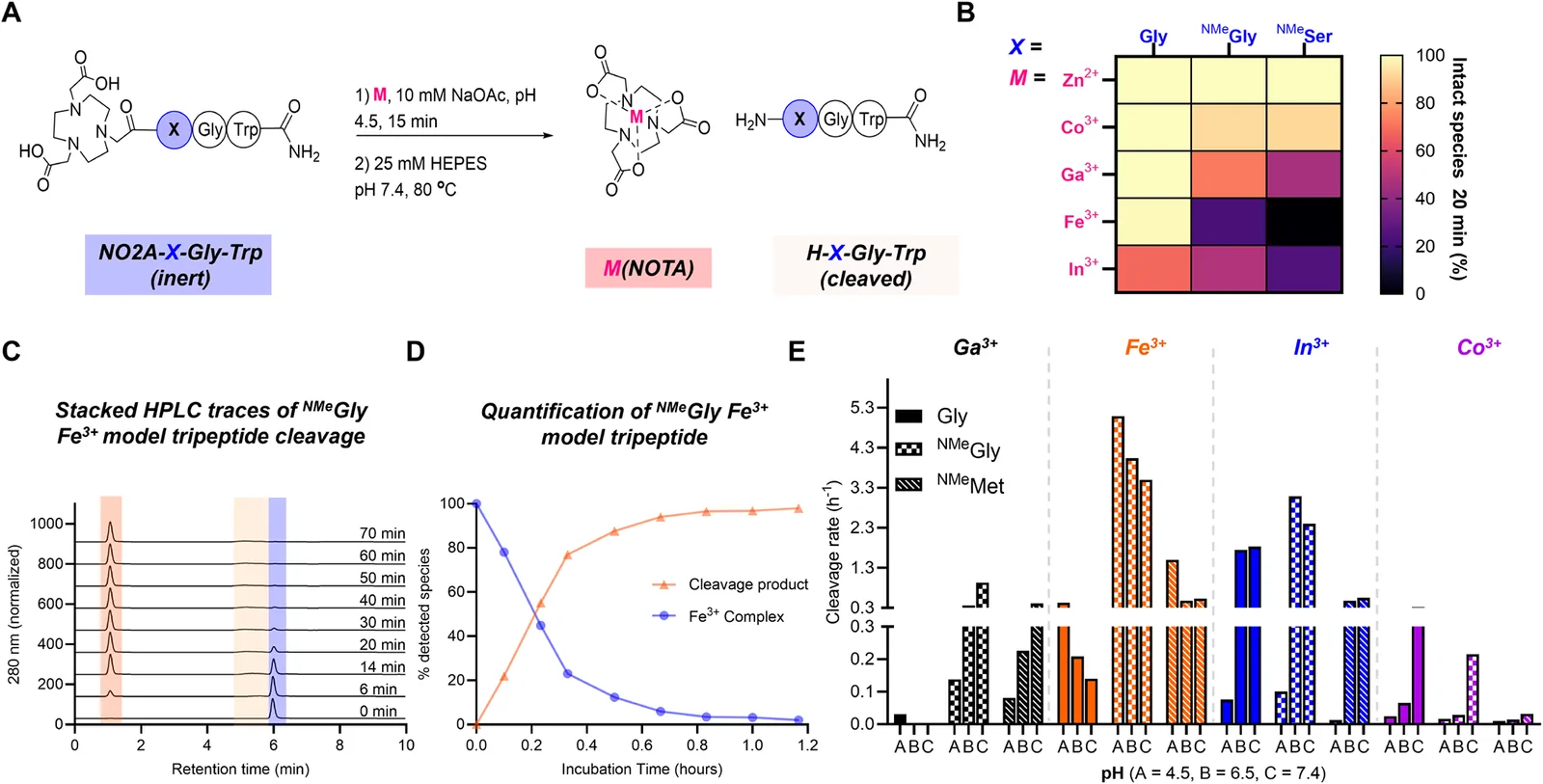
(A) Reaction scheme of M^3+^ with an *N*-methylated glycine (^NMe^Gly)-based model tripeptide, alongside the reaction scheme for monitoring autolytic amide bond cleavage; The Fe^3+^ complex is shown as an example. (B) Quantification of the cleavage at 80 °C and pH 7.4. (C) Comparative heatmap illustrating the time-dependent ^nat^Metal-driven autolytic cleavage kinetics across tested model tripeptide systems at the 20 min time point. (D) pH-dependent autolytic cleavage profiles (pH 4.5, pH 6.5, and pH 7.4) for Ga^3+^, Fe^3+^, In^3+^, and Co^3+^ model tripeptides (Gly, ^NMe^Gly, and ^NMe^Met).

### Mechanistic Studies

The discrepancy in pH dependence for the different TMAC-compatible metal ions suggests differing cleavage mechanisms, arising from divergent complex speciation for each metal ion. To identify the relevant aqueous species at different pH values, we performed pH-dependent nuclear magnetic resonance (NMR) spectroscopy or spectrophotometric titrations (for the paramagnetic Fe^3+^), complemented by single-crystal X-ray analysis. We evaluated the pH-dependent behavior of diamagnetic Zn^2+^, In^3+^, and Co^3+^ complexes at pH 3.0 – 8.0 with^1^H-NMR. Our prior investigations of the prototype Ga^3+^ complex indicated that reversible, coordinative isomerism between the N_3_O_3_ and N_4_O_2_ isomers occurred between pH 5.5–6.5 for canonical amino acids, induced by deprotonation of the amide proton. This resulted in a slowing of reaction rate for TMAC for nonmethylated amino acids, whereas non-canonical, *N*-methyl amino acids remain locked in N_3_O_3_ configuration and accelerate as pH becomes more basic.^22^

In contrast, In^3+^ and Co^3+^ both show an increase in reaction rates as pH increases, independent of the *N*-methylation status (Figure 2E). We conducted NMR spectroscopic analysis of [Zn­(NO2A)]-Ala-Gly-Trp and [In­(NO2A)]^+^-Ala-Gly-Trp complexes at pH values from 3.5 to 8. Our data indicated no pH-dependent chemical shift changes at 1.30 ppm (alanine methyl protons) and 4.20 ppm (methylene protons adjacent to the metal complex (Figures S100–S101). These results imply that neither Zn^2+^ nor In^3+^ undergo detectable interconversion between the N_3_O_3_ and N_4_O_2_ conformations. Using a small-molecule model complex for the nonmethylated (NH-containing) amino acid, we obtained structural characterization in the solid state. The [In­(NO2A)]^+^-NHBn complex crystallizes as the 7-coordinate complex [In­(NO2A)­Cl]-NHBn·H_2_O under acidic conditions, with a chloride ion occupying the 7th coordination site. We posit that TMAC with In^3+^ can occur by nucleophilic attack of the amide by an inner-sphere-coordinated aqua ligand. However, if competing ions occupy the ternary ligand-binding site, cleavage rates may decrease. To probe this hypothesis, we performed a chloride challenge assay: cleavage of [In­(NO2A)]^+^-^NMe^Gly-Gly-Trp was performed at pH 7.4 and room temperature with increasing chloride ion concentration (0 – 1000 mM). Indeed, we observed that 1 M Cl^–^ concentration reduced the cleavage rate by 50%, suggesting that ternary chloride ion coordination competes with water coordination, inhibiting amide bond hydrolysis (Figure 3B, Figures S108–S111). Alternatively, we cannot exclude that the ternary Cl^–^ attenuates the Lewis acidity of the In^3+^ metal center, thereby decreasing the polarization of the coordinated amide carbonyl and reducing its susceptibility to nucleophilic attack. Although the chloride challenge assay demonstrates that the Cl^–^ suppresses In^3+^-mediated TMAC activity, it is not possible to distinguish between these two mechanistic possibilities. In contrast, a control experiment with [Ga­(NO2A)]^+^-^NMe^Gly-Gly-Trp under increasing chloride concentration showed that the cleavage rate of the hexacoordinate Ga^3+^ complex was invariant (Figure 3B, Figures S112–S115).

**Figure 3:**
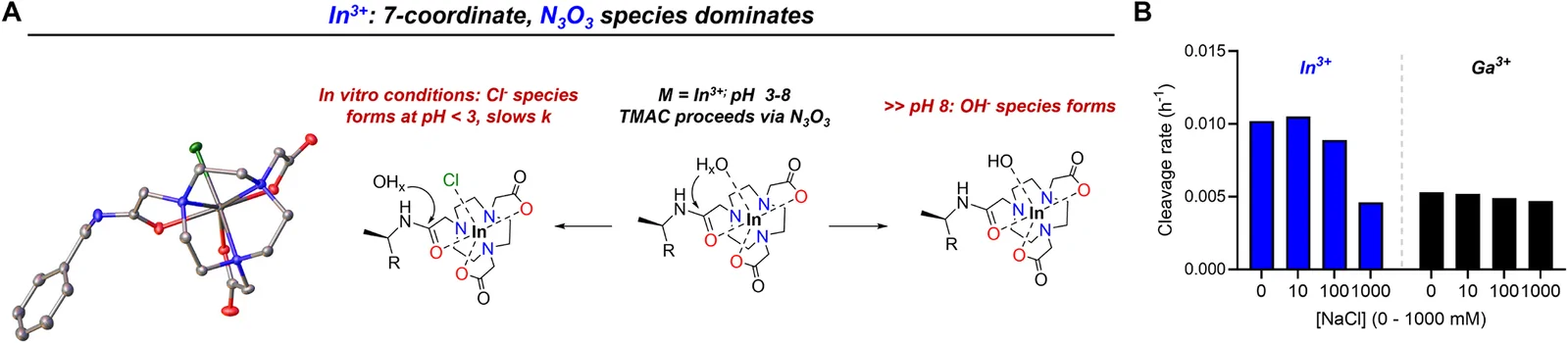
(A) A molecular drawing of [In­(NO2A)­Cl]-NHBn·H_2_O is shown with 50% probability ellipsoids and a schematic description of the cleavage mechanism by exogenous hydroxide/hydrido attack for In complexes under physiologically relevant conditions, under exclusive formation of the N_3_O_3_ complex species with one inner-sphere ternary ligand. Hydrogen atoms and water molecules are omitted for clarity. (B) Characterization of cleavage rates (h^-1^) of [In­(NO2A)]^+^-^NMe^Gly-Gly-Trp and [Ga­(NO2A)]^+^-^NMe^Gly-Gly-Trp complexes in presence of increasing Cl^-^ concentrations (0-1000 mM) at pH 7.4 and room temperature.

Spectral data of [Co­(NO2A)]^+^-Gly-Gly-Trp samples that were oxidized at pH 4.5 or 7, respectively, support that both isomers can form with Co^3+^, but do not interconvert dynamically, putatively due to the ligand-field stabilization of the d^6^-low-spin configuration (Figures S102–S103). Because of the highly positive redox potential of the Co^2+^ aqua ion, all Co^3+^ complexes are formed by way of Co^2+^ complexation, followed by oxidation to the corresponding Co^3+^ species. If the oxidation is conducted at pH 4.5, only the N_3_O_3_ species is observed in the corresponding ^1^H spectrum; however, at pH 7, only the N_4_O_2_ species is detected (Figures S102–S103). We obtained single-crystal X-ray structures of the [Co­(NO2A)]-NHBn complex under acidic (pH 3) and basic (pH 7.4) conditions (Figure 4), where the complex adopts N_3_O_3_ and N_4_O_2_ geometry, respectively, in good correlation with ^1^H NMR data (Figure S103). This structural assignment is consistent with the diminished TMAC reactivity of the Co^3+^ species oxidized at pH 7.0, which predominantly adopts the N_4_O_2_ coordination geometry (Figures S88–S90).

**Figure 4:**
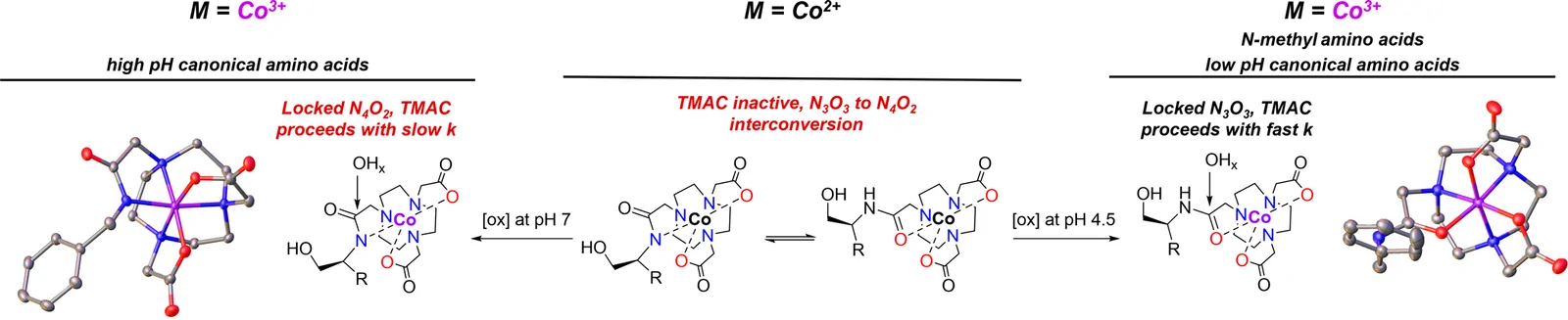
Molecular drawings of [Co­(NO2A)]^+^-NHBn and [Co­(NO2A)]-NHBn·4H_2_O shown with 50% probability ellipsoids, along with a schematic description of the cleavage mechanism by exogenous hydroxide/hydrate attack for Co^3+^ complexes under physiologically relevant conditions following oxidation of the TMAC-inactive, Co^2+^ species. Hydrogen atoms, water molecules, and perchlorate counterions are omitted for clarity.

The pH-dependent behavior of Fe^3+^ complexes displayed an inverse rate trend when compared with the other TMAC-compatible metal ion complexes: both nonmethylated and *N*-methylated conjugates slow in rate as pH increases, although this is less pronounced for *N*-methylated amino acid conjugates (Figure 2E, Figures S65–S74).

Due to the paramagnetic nature of Fe^3+^, NMR was not suitable to study the speciation behavior; as such, we pursued spectrophotometry and potentiometric titrations using the [Fe­(NO2A)]^+^-NHBn and [Fe­(NO2A)]^+^-NMeBn complexes instead. Stepwise increase of the pH of a solution containing [Fe­(NO2A)]^+^-NHBn revealed emergence of a charge-transfer (CT) band at 430 nm (Figure S106). Single-crystal X-ray analysis of [Fe­(NO2A)]^+^-NHBn grown from a pH 7 sample shows the formation of an oxo-bridged dimeric structure, [Fe­(NO2A)-NHBn]_2_O (Figure 5A), in good correlation with observation of a Fe-oxo CT band. According to UV–vis and HPLC analysis, the formation of the oxo-bridged dimer is both pH- and concentration-dependent and represents the dominant species above pH 5 (Figure 5D, Figure S106). The solid-state structure appears further stabilized by the formation of hydrogen bonds between the amide O and the NH, indicating that the coordination of the carbonyl is lost as pH increases. Single-crystal X-ray analysis under acidic conditions (pH 2) suggests N_3_O_3_ coordination geometry of [(Fe­(NO2A)]^+^-NHBn (Figure 5A). This correlates well with diminished TMAC reaction rates due to the lack of metal-mediated bond polarization. In contrast, [Fe­(NO2A)]^+^-NMeBn did not produce a CT band, suggesting the formation of a monomeric structure (Figure S107); potentiometric titration data indicate a deprotonation event with a pKa of 6.15, correlating to the formation of a 6-coordinate species with an inner-sphere hydroxo ligand (Figure 5C). Therefore, the Fe^3+^ complexes favor N_3_O_3_ configuration under acidic conditions, where the amide bond is efficiently polarized and cleaved. As pH increases, the Fe^3+^ complex forms a hydroxo species, which exists in equilibrium with an oxo-bridged dimer with nonmethylated (NH) amino acids, or Fe^3+^ hydroxyl species (*N*-methylated, *N*-Me). For both conjugate types, we posit that coordination of the amide carbonyl is lost, which results in a lack of efficient bond polarization and thus, deceleration of the cleavage rate at elevated pH.

**Figure 5:**
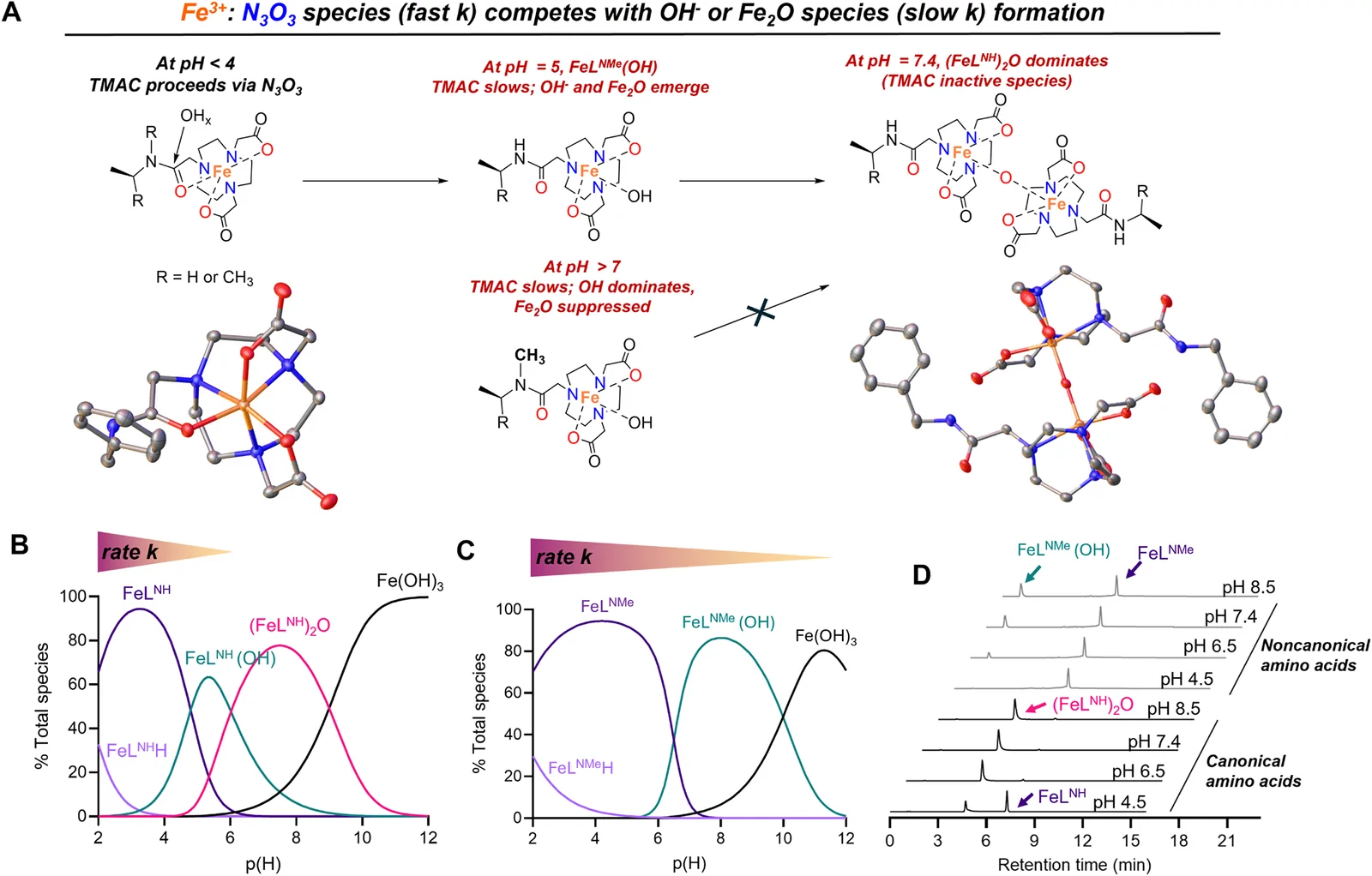
(A) Molecular drawings of [Fe­(NO2A)]^+^-NHBn and [Fe­(NO2A)-NHBn]_2_O shown with 50% probability ellipsoids, along with a schematic description of the cleavage mechanism by exogenous hydroxide/hydrido attack for Fe complexes under physiologically relevant conditions, under formation of the oxo/hydroxo species above pH 6. Hydrogen atoms and perchlorate counterions are omitted for clarity. (B) Best fit of pH-dependent speciation for FeLNHBn and (C) FeLNMeBn species correlated with reaction rate trends. (D) Stacked HPLC traces showing the pH-dependent speciation of FeLNHBn and FeLNMeBn complexes. The binary solvent system (water + 0.1% formic acid; acetonitrile + 0.1% formic acid) was used as the HPLC mobile phase.

### Metal-Mediated Prodrug Release In Vitro

Previously, we have shown that the rate of cleavage of [^68^Ga]-labeled radiopharmaceuticals can be tuned by modulation of the complex-adjacent amino acids.^22^ This strategy is not suitable for tuning the rate of drug release of drugs with defined chemical structures or peptide sequences. As such, we proposed that the rate of release and activity can be readily tuned by changing the identity of the metal ion instead.^34–36^ We identified monomethyl auristatin E (MMAE), a synthetic tubulin polymerization inhibitor, with a terminal *N*-methylvaline to validate rate-tunable TMAC.^37–40^

We proposed that incorporation of the [M­(NO2A)]^n+^ complex to the N-terminus could modulate the drug’s potency, with TMAC serving as a slow-release linker strategy akin to the maleimidocaproyl-l-valine-l-citrulline-*p*-aminobenzyl alcohol (MC-Val-Cit-PAB) linker, a peptide linker designed for Cathepsin B-specific cleavage.^41^ The synthesis of a corresponding conjugate, PD1, was achieved by solution-phase amidation and subsequent acid-catalyzed saponification of orthogonally protected 1,4,7-triazacyclonane-1,4,7-triacetic acid (Figure 6A, Scheme S1). The resulting construct, PD1, was then complexed with Zn^2+^, Ga^3+^, Fe^3+^, Co^3+^, and In^3+^ to form Zn-PD1, Ga-PD1, Fe-PD1, Co-PD1, and In-PD1, respectively (Scheme S4). We first determined the amide bond cleavage activity of PD1 in vitro. In the absence of metals, apo-PD1 could not trigger cleavage at pH 7.4 and 80 °C (Figure 6B, Figure S155).

**Figure 6:**
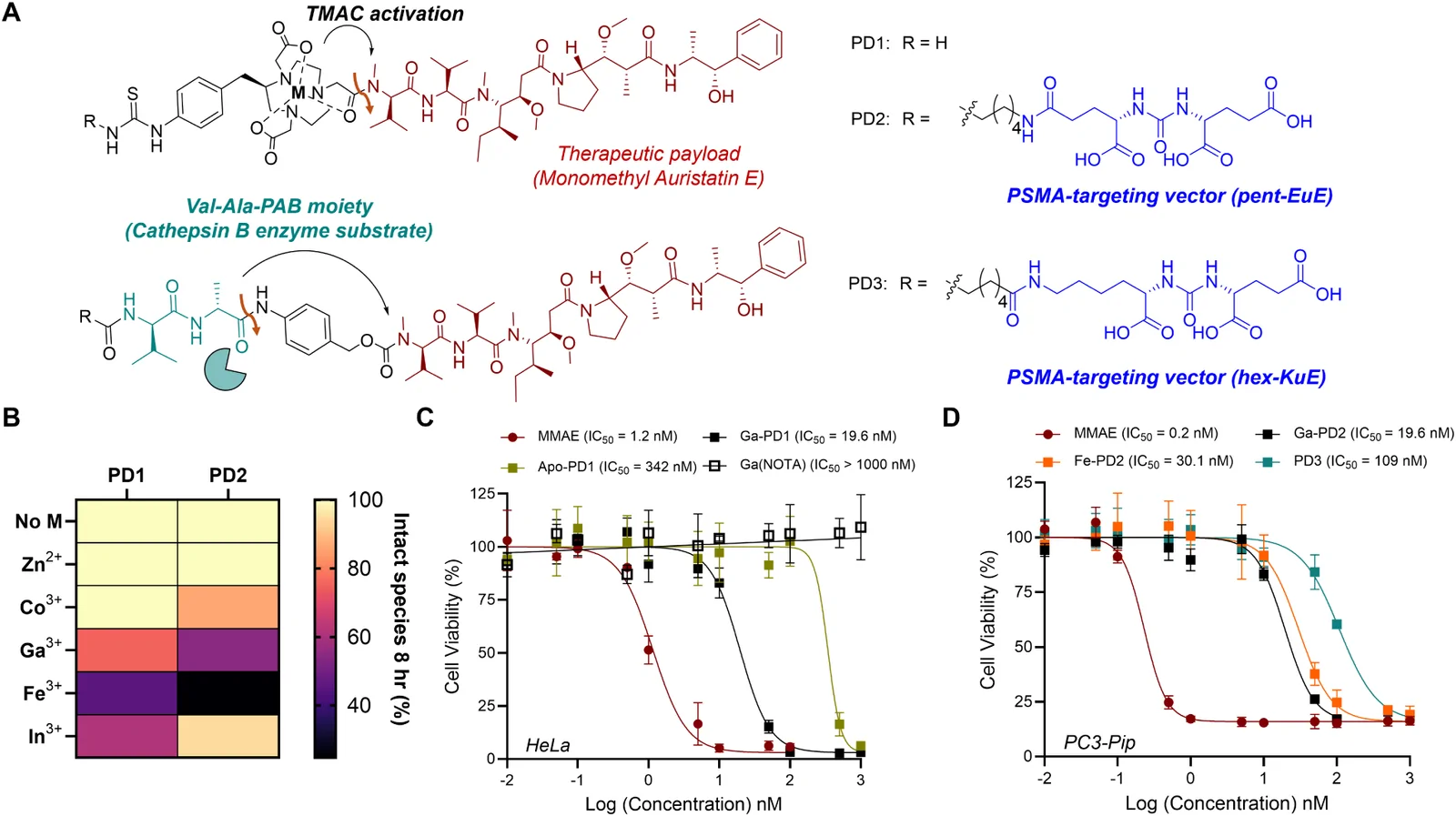
(A) Chemical structures of PD1, PD2, and PD3 conjugates and the corresponding payload release mechanism. (B) Comparative heatmap illustrating the time-dependent autolytic cleavage kinetics of PD1 and PD2 complexes. (C) HeLa cell viability after 72 h incubation with MMAE, Ga-PD1, apo-PD1, or the Ga­(NOTA) complex. Cells were treated with the indicated concentrations. Data represent mean ± standard deviation (SD) of 3 technical replicate wells from a representative experiment. Each IC_50_ value is reported with a 95% confidence interval (CI): MMAE (95% CI: 0.9–1.5 nM), Ga-PD1 (95% CI: 16.1–23.9 nM), apo-PD1 (95% CI: values undetermined). (D) PC3-pip cell viability after 72 h incubation with MMAE, Ga-PD2, Fe-PD2, or PD3. Data represent mean ± SD of 3 technical replicate wells from a representative experiment. Each IC_50_ value is reported with 95% CI: MMAE (95% CI: 0.2–0.3 nM), Ga-PD2 (95% CI: 15.7–24.0 nM), Fe-PD2 (95% CI: 22.9–39.3 nM), and PD3 (95% CI: 91–133 nM).

To evaluate the cytotoxicity (IC_50_) of the PD1 complexes, we conducted a standard MTT (3-(4,5-dimethylthiazol-2-yl)-2,5-diphenyltetrazolium bromide) assay in HeLa cervical cancer cells (Figure 6C).^42^ Under these conditions, MMAE produced an IC_50_ value of 1.2 nM after 72 h of incubation, which is in good agreement with literature reports (Figure 6C).^43–45^ For the metal-free PD1 ligand (apo-PD1), we observed a 400-fold reduction (IC_50_ = 342 nM), with the TMAC-inactive complex Zn-PD1 performing similarly (IC_50_ = 502 nM) (Figure 6C, Figures S172–S173). In contrast, all TMAC-active PD1 complexes showed greater potency, ranging from 19.6 nM for the Ga-PD1 complex to 214 nM and 396 nM for the Fe-PD1 and Co-PD1 complexes, respectively (Figure 6C, Figures S173–S175). Control experiments with M­(NOTA) complexes showed that the metal complex itself imparted no additional toxicity, affirming that the released MMAE was responsible for the cytotoxic effect of the Ga-PD1 conjugate (Figure 6C and Figures S176–S177).

While these results were encouraging, in vivo applications of prodrugs require targeted delivery to a cancer subtype to prevent systemic toxicity. To this end, we synthesized a prostate-specific membrane antigen (PSMA)-targeting MMAE prodrug conjugate, PD2 (Figure 6A, Scheme S2). The PD2 molecule consists of a backbone-functionalized NO2A with the PSMA-targeting pentyl-EuE peptide linked using a benzylthiourea bond, while one carboxylic acid arm was amidated with the MMAE payload. Zn-PD2, Ga-PD2, Fe-PD2, Co-PD2, and In-PD2 were formed and characterized for TMAC-mediated MMAE payload release in vitro; in all cases, cleavage rates followed the same trends but were accelerated, putatively due to the enhanced steric bulk introduced by the functionalized triazamacrocycle (Figure 6B, Figures S161–S166).

Subsequently, we conducted an MTT assay to assess the potency of the metal complexes in the PC3-pip cell line, which is engineered to express high levels of PSMA receptor. Notably, PD2 displayed metal-dependent differences in cytotoxic potency. Free MMAE had an IC_50_ of 0.2 nM, whereas the potency of apo-PD2 was significantly reduced (IC_50_ > 1000 nM at 72-h incubation) (Figure 6D, Figure S181). Ga-PD2 and Fe-PD2 showed comparable IC_50_ values of 20 and 30 nM, respectively, and Co-PD2 displayed a more moderate IC_50_ of 182 nM (Figure 6D, Figure S180). These values correlated well with the observed trends for rates of release. Additionally, a valine–alanine (Val–Ala) dipeptide sequence was selected as an enzymatically cleavable benchmark to compare performance with metal-mediated bond cleavage. This dipeptide sequence is recognized by the cathepsin B enzyme and employed as a release trigger for antibody-drug conjugate (ADC) payloads.^46,47^ Consequently, an additional control molecule, PD3, linking the PSMA-targeting vector with MMAE via the Val–Ala dipeptide, achieved only an IC_50_ = 109 nM at 72h (Figure 6D). These data are consistent with other literature reports, which indicate that linking potent cytotoxins to targeting vectors leads to diminished potency.^48,49^

Both In-PD1 and In-PD2 displayed the lowest potency with IC_50_ values greater than 1000 nM and 407 nM, respectively, despite the in vitro cleavage rates correlating well with those observed for the Ga-PD2 and Fe-PD2 complexes (Figures S174, S179). However, we note the susceptibility of the In^3+^ species to inhibition of TMAC in the presence of high chloride ion concentrations: the cell culture media contains approximately 110 mM chloride, which is within the concentration range of Cl^–^ that was found to suppress TMAC for In^3+^ derivatives. Therefore, this high chloride concentration may suppress In^3+^-mediated TMAC activity in cell culture, reducing the cellular potency of In^3+^ conjugates.

### Demonstrating In Vivo Efficacy of a TMAC-Compatible Theranostic Pair

Peptide-drug conjugates generally lack a companion diagnostic, preventing patient stratification for better clinical outcomes. Ga^3+^ and In^3+^ both have short-lived radioactive isotopes that are routinely used as diagnostic agents in the clinic: ^68^Ga (β_avg_= 890 keV, 89%, *t*_1/2_ = 1.1 h) offers positron emission tomography (PET), and ^111^In (EC, γ = 171, 245 keV, *t*_1/2_ = 67 h) is compatible with single-photon emission computed tomography (SPECT). However, neither element has an isotope or congener with therapeutic emission properties, limiting their use to diagnostic imaging. The combination of these diagnostic isotopes and their compatibility with TMAC provides a viable avenue for the development of a new generation of theranostic agents.

To this end, we first evaluated the in vivo diagnostic efficacy of the PD2 construct. PD2 was radiolabeled with ^68^GaCl_3_ to form [^68^Ga]­[Ga-PD2] (Figure S183). We employed a bilateral murine tumor model bearing a PSMA-expressing tumor on the right flank and a PSMA-negative tumor on the left flank. [^68^Ga]­[Ga-PD2] was administered systemically by tail-vein injection, and mice were imaged at 30, 60, and 90 min, followed by terminal biodistribution and urine metabolite analysis at 120 min post-injection (p.i., Figure 7A). [^68^Ga]­[Ga-PD2] selectively accumulated in the PSMA-positive tumor (9.61% ± 4.47% ID/g) with minimal off-target uptake, affirming that target-specific delivery was feasible (Figure S193). Metabolite analysis indicated that the radiolabeled compound remained 98% intact in vivo 2 h p.i. (Figure S194). A corresponding, analogous in vivo experiment with [^111^In]­[In-PD2] showed comparable target-specific uptake in PSMA-expressing tumors (7.97% ± 0.41% ID/g), remaining 91% intact in vivo at 2 h p.i. (Figures S193, S195).

**Figure 7:**
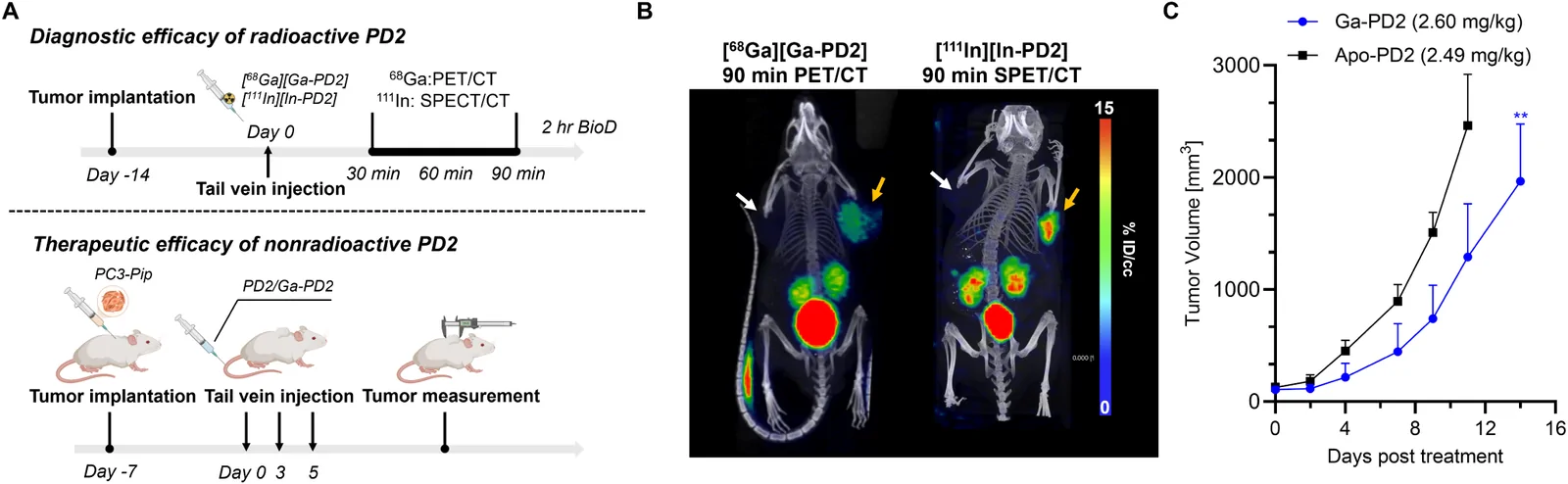
(A) Schematic illustration of in vivo experiments. Top: schematic timeline of PET/CT and SPECT/CT imaging study with [^68^Ga]­[Ga-PD2] and [^111^In]­[In-PD2]. Bottom: schematic timeline of therapy study with Ga-PD2 treatment. (B) Left: [^68^Ga]­[Ga-PD2] 90 min p.i. PET/CT image. Right: [^111^In]­[In-PD2] 90 min p.i. SPECT/CT image. White arrow indicates a PSMA (−) tumor. Yellow arrow indicates a PSMA (+) tumor. (C) Tumor growth curve over the therapy study. Data are represented as mean ± SEM (*n* = 7). An unpaired *t*-test indicated a significant treatment effect (**P < 0.01).

Next, we evaluated the in vivo therapeutic efficacy of the corresponding, nonradioactive complex. Cell-based toxicity assays identified Ga-PD2 as the most promising candidate. As such, we conducted a therapy study with Ga-PD2 treatment using a unilateral murine tumor model bearing a PSMA-positive tumor on the right flank. Ga-PD2 was administered systemically by tail-vein injection three times at a dose of 2.60 mg/kg, and tumor growth was monitored and compared with PD2-treated control cohorts (Figure 7A). Treatment with Ga-PD2 effectively attenuated tumor growth and prolonged survival by 5 days relative to the apo-PD2 cohort (Figures S196, S197). Body-weight measurements indicated that the treatment was well tolerated, with less than 10% body loss, suggesting limited systemic toxicity and demonstrating the in vivo compatibility of TMAC with a theranostic paradigm (Figure S198).

## Conclusions

Here, we explore how the identity of the metal center influences the reactivity of metal-mediated amide-bond cleavage (TMAC) to release a potent cytotoxin. Our studies reveal that the TMAC platform can be readily expanded beyond Ga^3+^ to Fe^3+^, Co^3+^, and In^3+^. Mechanistic studies using spectroscopy, potentiometry, and crystallography provided insight into how metal-dependent coordination geometries dictate pH- and coordinating-anion-dependent TMAC reactivity. We subsequently adapted TMAC to selectively release a potent cytotoxin, MMAE, in vitro; release rates correlated well with cytotoxicity in various cell lines and upon incorporation of a PSMA-targeting peptide. Finally, in vivo evaluation of the PSMA-functionalized PD2 construct demonstrated the feasibility of TMAC to deliver a diagnostic and therapeutic payload; a low-dose treatment with Ga-PD2 showed a delay in tumor growth, without evidence of off-target toxicity. The pH dependence of TMAC may further influence in vivo efficacy, especially in the acidic tumor microenvironment. The pH-dependent behavior of TMAC under more physiologically relevant culture conditions requires further investigation. In addition, the established in vivo stability and kinetic inertness of NOTA-based metal complexes exclude degradation by transmetalation under physiological conditions.^24,29,50^ The cell viability measurements and in vivo studies of the TMAC conjugates are consistent with this expectation.

While the therapy study did not identify Ga-PD2 as a particularly potent anticancer agent, we consider these results a starting point that warrants structure-activity studies to further tune release rate and in vivo properties of trivalent metal ion chelates compatible with TMAC.

## Supplementary Material



## Notes

The Supporting Information including detailed synthesis procedures, characterization data, cleavage experiments, mechanistic studies, images, and related biodistribution analyses is provided within the supporting information (PDF)

## References

[1] Xiao W., Jiang W., Chen Z., Huang Y., Mao J., Zheng W., Hu Y., Shi J. (2025). Advance in peptide-based
drug development: Delivery platforms, therapeutics and vaccines. Signal Transduction Targeted Ther.

[2] Muttenthaler M., King G. F., Adams D. J., Alewood P. F. (2021). Trends in peptide
drug discovery. Nat. Rev. Drug Discovery.

[3] Wang L., Wang N., Zhang W., Cheng X., Yan Z., Shao G., Wang X., Wang R., Fu C. (2022). Therapeutic
peptides: Current applications and future directions. Signal Transduction Targeted Ther.

[4] Fetse J., Kandel S., Mamani U.-F., Cheng K. (2023). Recent advances in
the development of therapeutic peptides. Trends
Pharmacol. Sci.

[5] Shabsigh M., Solomon L. A. (2024). Peptide PET Imaging: A Review of
Recent Developments
and a Look at the Future of Radiometal-Labeled Peptides in Medicine. Chem. Biomed. Imaging.

[6] Zhang S., Wang X., Gao X., Chen X., Li L., Li G., Liu C., Miao Y., Wang R., Hu K. (2025). Radiopharmaceuticals
and their applications in medicine. Signal Transduction
Targeted Ther.

[7] Rizvi S. F. A., Zhang L., Zhang H., Fang Q. (2024). Peptide-Drug Conjugates:
Design, Chemistry, and Drug Delivery System as a Novel Cancer Theranostic. ACS Pharmacol. Transl. Sci.

[8] Mudd G. E., Scott H., Chen L., van Rietschoten K., Ivanova-Berndt G., Dzionek K., Brown A., Watcham S., White L., Park P. U. (2022). Discovery
of BT8009:
A Nectin-4 Targeting Bicycle Toxin Conjugate for the Treatment of
Cancer. J. Med. Chem..

[9] He H., Deng X., Wang Z., Chen J. (2025). Recent progress in
the development of peptide-drug conjugates (PDCs) for cancer therapy. Eur. J. Med. Chem.

[10] Dubowchik G. M., Mosure K., Knipe J. O., Firestone R. A. (1998). Cathepsin
B-sensitive dipeptide prodrugs. 2. Models of anticancer drugs paclitaxel
(Taxol®), mitomycin C and doxorubicin. Biorg. Med. Chem. Lett.

[11] Li S., Guo Y., Che J., Dai H., Dong X. (2025). Recent Advances in
Peptide Linkers for Antibody-Drug Conjugates. J. Med. Chem.

[12] Carl P. L., Chakravarty P. K., Katzenellenbogen J. A. (1981). A novel
connector linkage applicable
in prodrug design. J. Med. Chem.

[13] Watanabe T., Arashida N., Fujii T., Shikida N., Ito K., Shimbo K., Seki T., Iwai Y., Hirama R., Hatada N. (2024). Exo-Cleavable
Linkers: Enhanced Stability and Therapeutic
Efficacy in Antibody–Drug Conjugates. J. Med. Chem..

[14] Chuprakov S., Ogunkoya A. O., Barfield R. M., Bauzon M., Hickle C., Kim Y. C., Yeo D., Zhang F., Rabuka D., Drake P. M. (2021). Tandem-Cleavage
Linkers Improve the In Vivo Stability
and Tolerability of Antibody–Drug Conjugates. Bioconjugate Chem.

[15] Tedeschini T., Campara B., Grigoletto A., Zanotto I., Cannella L., Gabbia D., Matsuno Y., Suzuki A., Yoshioka H., Armirotti A. (2024). Optimization of a pendant-shaped PEGylated
linker for antibody-drug conjugates. J. Controlled
Release.

[16] Balamkundu S., Liu C.-F. (2023). Lysosomal-Cleavable Peptide Linkers in Antibody–Drug
Conjugates. Biomedicines.

[17] Lei Y., Zheng M., Chen P., Seng Ng C., Peng Loh T., Liu H. (2025). Linker Design for the
Antibody Drug Conjugates: A Comprehensive Review. ChemMedchem.

[18] Zhao S., Yu N., Han H., Guo S., Murthy N. (2025). Advances in acid-degradable
and enzyme-cleavable linkers for drug delivery. Curr. Opin. Chem. Biol.

[19] Nazli A., Irshad Khan M. Z., Rácz Á., Béni S. (2024). Acid-sensitive
prodrugs; a promising approach for site-specific and targeted drug
release. Eur. J. Med. Chem.

[20] Tu Y., Gong J., Mou J., Jiang H., Zhao H., Gao J. (2024). Strategies for the development of stimuli-responsive small molecule
prodrugs for cancer treatment. Front. Pharmacol.

[21] Wang Q., Guan J., Wan J., Li Z. (2020). Disulfide based prodrugs
for cancer therapy. RSC Adv.

[22] Zhong Z., Ṡmiłowicz D., Gork M. J., Garman L. C., Guzei I. A., Boros E. (2025). Rate-Tunable,
Metal-Mediated Amide
Bond Cleavage for the Controlled Release of Pharmaceuticals. J. Am. Chem. Soc.

[23] Kras E. A., Snyder E. M., Sokolow G. E., Morrow J. R. (2022). Distinct Coordination
Chemistry of Fe­(III)-Based MRI Probes. Acc.
Chem. Res.

[24] Kras E. A., Abozeid S. M., Eduardo W., Spernyak J. A., Morrow J. R. (2021). Comparison
of phosphonate, hydroxypropyl and carboxylate pendants in Fe­(III)
macrocyclic complexes as MRI contrast agents. J. Inorg. Biochem.

[25] Heskamp S., Laverman P., Rosik D., Boschetti F., van der Graaf W. T. A., Oyen W. J. G., van Laarhoven H. W. M., Tolmachev V., Boerman O. C. (2012). Imaging of Human Epidermal Growth
Factor Receptor Type 2 Expression with ^18^F-Labeled Affibody
Molecule Z_HER2: 2395_ in a Mouse Model for Ovarian
Cancer. J. Nucl. Med.

[26] Lin W., Ṡmiłowicz D., Joaqui-Joaqui M. A., Bera A., Zhong Z., Aluicio-Sarduy E., Mixdorf J. C., Barnhart T. E., Engle J. W., Boros E. (2024). Controlling
the Redox Chemistry of Cobalt Radiopharmaceuticals. Angew. Chem. Int. Ed.

[27] Vágner A., Forgács A., Brücher E., Tóth I., Maiocchi A., Wurzer A., Wester H. J., Notni J., Baranyai Z. (2018). Equilibrium Thermodynamics,
Formation, and Dissociation
Kinetics of Trivalent Iron and Gallium Complexes of Triazacyclononane-Triphosphinate
(TRAP) Chelators: Unraveling the Foundations of Highly Selective Ga-68
Labeling. Front. Chem.

[28] Price E. W., Orvig C. (2014). Matching chelators to radiometals
for radiopharmaceuticals. Chem. Soc. Rev.

[29] Wadas T. J., Wong E. H., Weisman G. R., Anderson C. J. (2010). Coordinating Radiometals
of Copper, Gallium, Indium, Yttrium, and Zirconium for PET and SPECT
Imaging of Disease. Chem. Rev.

[30] Boros E., Packard A. B. (2019). Radioactive Transition
Metals for Imaging and Therapy. Chem. Rev.

[31] Pearson R. G. (1963). Hard and
Soft Acids and Bases. J. Am. Chem. Soc.

[32] Parr R. G., Pearson R. G. (1983). Absolute hardness: Companion parameter to absolute
electronegativity. J. Am. Chem. Soc.

[33] Pearson R. G. (1988). Absolute
electronegativity and hardness: Application to inorganic chemistry. Inorg. Chem.

[34] Lahnif H., Grus T., Salvanou E.-A., Deligianni E., Stellas D., Bouziotis P., Rösch F. (2023). Old Drug,
New Delivery Strategy: MMAE Repackaged. Int.
J. Mol. Sci.

[35] Moquist P. N., Bovee T. D., Waight A. B., Mitchell J. A., Miyamoto J. B., Mason M. L., Emmerton K. K., Stevens N., Balasubramanian C., Simmons J. K. (2021). Novel Auristatins with High Bystander and Cytotoxic
Activities in Drug Efflux–positive Tumor Models. Mol. Cancer Ther.

[36] Doronina S. O., Toki B. E., Torgov M. Y., Mendelsohn B. A., Cerveny C. G., Chace D. F., DeBlanc R. L., Gearing R. P., Bovee T. D., Siegall C. B. (2003). Development of potent
monoclonal antibody auristatin conjugates for cancer therapy. Nat. Biotechnol..

[37] Akaiwa M., Martin T., Mendelsohn B. A. (2018). Synthesis
and Evaluation of Linear
and Macrocyclic Dolastatin 10 Analogues Containing Pyrrolidine Ring
Modifications. ACS Omega.

[38] Sokka I. K., Ekholm F. S., Johansson M. P. (2019). Increasing
the Potential of the Auristatin
Cancer-Drug Family by Shifting the Conformational Equilibrium. Mol. Pharmaceutics.

[39] Davis R. A., Ganguly T., Harris R., Hausner S. H., Kovacs L., Sutcliffe J. L. (2023). Synthesis
and Evaluation of a Monomethyl Auristatin
E—Integrin αvβ6 Binding Peptide–Drug Conjugate
for Tumor Targeted Drug Delivery. J. Med. Chem.

[40] Hingorani D. V., Allevato M. M., Camargo M. F., Lesperance J., Quraishi M. A., Aguilera J., Franiak-Pietryga I., Scanderbeg D. J., Wang Z., Molinolo A. A. (2022). Monomethyl
auristatin antibody and peptide drug conjugates for trimodal cancer
chemo-radio-immunotherapy. Nat. Commun..

[41] Yaghoubi S., Gharibi T., Karimi M. H., Sadeqi Nezhad M., Seifalian A., Tavakkol R., Bagheri N., Dezhkam A., Abdollahpour-Alitappeh M. (2021). Development and biological
assessment
of MMAE-trastuzumab antibody–drug conjugates (ADCs). Breast Cancer.

[42] Supino, R. MTT Assays. In In Vitro Toxicity Testing Protocols. O’hare, S. ; Atterwill, C. K. , Eds.; Humana Press, 1995, pp. 137–149.

[43] Koga Y., Manabe S., Aihara Y., Sato R., Tsumura R., Iwafuji H., Furuya F., Fuchigami H., Fujiwara Y., Hisada Y. (2015). Antitumor
effect of
antitissue factor antibody-MMAE conjugate in human pancreatic tumor
xenografts. Int. J. Cancer.

[44] Qi R., Wang Y., Bruno P. M., Xiao H., Yu Y., Li T., Lauffer S., Wei W., Chen Q., Kang X. (2017). Nanoparticle conjugates
of a highly potent toxin enhance safety and
circumvent platinum resistance in ovarian cancer. Nat. Commun..

[45] Abuhay M., Kato J., Tuscano E., Barisone G. A., Sidhu R. S., O’donnell R. T., Tuscano J. M. (2016). The HB22.7–vcMMAE antibody–drug
conjugate has efficacy against non-Hodgkin lymphoma mouse xenografts
with minimal systemic toxicity. Cancer Immunol.,
Immunother.

[46] Li Y., Wang Y., Lu Y., Xie J., Hu J., Pan F., Wang Q., Lu W., Zhang J., Zhu S. (2026). Valine-Alanine-Based
Peptidomimetic Linker for Antibody-Drug Conjugates. J. Med. Chem.

[47] Tian H., Pan W., Shi H., Shao X., Sun J., Liang J., Chen B., Cheng B., Liu K., Yao G. (2026). Coibamide
Analogue as a Novel-Class Payload for Antibody-Drug Conjugate. J. Med. Chem..

[48] Burns K. E., Hensley H., Robinson M. K., Thévenin D. (2017). Therapeutic
Efficacy of a Family of pHLIP-MMAF Conjugates in Cancer Cells and
Mouse Models. Mol. Pharmaceutics.

[49] Gomena J., Modena D., Cordella P., Vári B., Ranđelović I., Borbély A., Bottani M., Vári-Mező D., Halmos G., Juhász É. (2024). In vitro and in vivo evaluation of Bombesin-MMAE
conjugates for targeted tumour therapy. Eur.
J. Med. Chem..

[50] Ferreira C. L., Yapp D. T. T., Mandel D., Gill R. K., Boros E., Wong M. Q., Jurek P., Kiefer G. E. (2012). 68Ga Small Peptide
Imaging: Comparison of NOTA and PCTA. Bioconjugate
Chem.

