# Supplementary material for: Metal-Dependent Coordination Geometry Controls Autolytic Amide Bond Cleavage to Enable Prodrug Activation
Source: J Am Chem Soc. 2026 Sep 14;148(38):41603–11. doi: 10.1021/jacs.6c15432 (PMC13632579; doi:10.1021/jacs.6c15432)
Supplement: Supplementary file 1 [file ja6c15432_si_001.pdf]

Supporting Information for: Metal-Dependent Coordination Geometry Controls Autolytic Amide Bond Cleavage to Enable Prodrug Activation

Zhuoran Zhong, Dariusz Śmiłowicz, M. Andrey Joaqui-Joaqui, Phuong Nguyen Tran, Leah C. Garman, Ilia A. Guzei, and Eszter Boros*.

1 Table of Contents

2 Experimental procedures 3
2.1 Materials 3
2.2 General Methods for Characterization 3
3 Synthesis and Characterization of Model Peptides 6
3.1 Synthesis of Model Tripeptides 6
3.2 HRMS, HR-ESI-MS and HPLC Characterization Data 7
3.3 NMR Spectra of Peptides 13
4 Complex synthesis and characterization 27
4.1 General Complexation and Cleavage Assay Protocol 27
4.2 HRMS and HPLC Data for Coordination Complexes 27
4.3 Cleavage assays of model tripeptide complexes 34
5 Mechanism Studies 57
5.1 Metal Complex Speciation 57
5.2 Chloride ion challenge assay on indium and gallium complexes cleavage experiment 63
6 Synthesis and Characterizations of MMAE Conjugates. 68
6.1 Synthesis and characterization of PD1 and PD2 conjugates 68
6.2 PD1 and PD2 Complexation Protocol 87
6.3 Cleavage assay of PD1 and PD2 complexes 97
6.4 Ultraviolate-Visible Spectroscopy Data of PD1 and PD2 104
7 In vitro and in vivo characterization 106
7.1 Cell viability studies 106
7.2 Tumor xenograft model 110
7.3 ^{68}Ga Radiolabeling Procedure 111
7.4 ^{111}In Radiolabeling Procedure 112
7.5 ^{111}In ^{NMe}Gly-Gly-Trp Radiolabeling Procedure 112
7.6 Chloride ion challenge assay on [^{111}In][In(NO2A)]$^{+}$-^{NMe}G-G-W 113
7.7 Tabulated data 116
7.8 In vivo PET-CT imaging and Ex Vivo Biodistribution Results of PD1 and PD2. 117
7.9 Urine metabolite analysis 119

7.10 Therapy study results of apo-PD2 and Ga-PD2 complex 120
8 Crystallographic data 123
8.1 Crystallization methods 123
8.2 X-ray Diffraction Analysis of [In(NO2A)Cl]-NHBn·H_2O ([In(6)Cl]·H_2O). 123
8.2.1 Data Collection 123
8.2.2 Structure Solution and Refinement 123
8.2.3 Summary 124
8.3 X-ray Diffraction Analysis of $[Co(NO2A)]^+$-NHBn ([Co(6)]ClO_4). 128
8.3.1 Data Collection 128
8.3.2 Structure Solution and Refinement 128
8.3.3 Summary 128
8.4 X-ray Diffraction Analysis of [Co(NO2A)]-NHBn·4H_2O ([Co(6)]·4H_2O). 132
8.4.1 Data Collection 132
8.4.2 Structure Solution and Refinement 132
8.4.3 Summary 132
8.5 X-ray Diffraction Analysis of $[Fe(NO2A)]^+$-NHBn ([Fe(6)]ClO_4). 137
8.5.1 Data Collection 137
8.5.2 Structure Solution and Refinement 137
8.5.3 Summary 137
8.6 X-ray Diffraction Analysis of $[Fe(NO2A)\text{-}NHBn]_2O$ ($[Fe(6)]_2O$). 141
8.6.1 Data Collection 141
8.6.2 Structure Solution and Refinement 141
8.6.3 Summary 142
8.7 Acknowledgement 145
9 References 146

2 Experimental procedures

2.1 Materials

All starting materials were purchased from Acros Organics, Alfa Aesar, Millipore Sigma or TCI America and used without further purification. Fmoc-protected amino acids were purchased from Bachem. NOTA-bis(t-Bu ester) and p-SCN-Bn-NOTA compounds were obtained from Macrocyclics. Fmoc-Trp(Boc)-Rink amide resin was purchased from AAPPTec. $^{68}GaCl_3$ was obtained from a $^{68}Ge/^{68}Ga$ generator (Eckhard & Ziegler, 15 - 25 mCi in 1 mL). $^{111}InCl_3$ was obtained from BWXT Medical (22 mCi in 70 µL).

2.2 General Methods for Characterization

Nuclear magnetic resonance (NMR) spectra (^1H, $^{13}C\{^1H\}$, COSY, HSQC) were collected at University of Wisconsin Madison a 500 and 600 MHz III Bruker instrument at 25 °C and processed using MestReNova. and at the University of Wisconsin-Madison Department of Chemistry Paul Bender Chemical Instrumentation Center (CIC) using a Bruker Avance III 500 with a DCH liquid He cryoprobe (Bender Fund), a Bruker Avance Neo 500 with a 5mm Prodigy-BBO liquid N_2 cryoprobe (NSF CHE-2017891), and a Bruker Avance III 600 with a TCI-F liquid He cryoprobe (NIH S10 OD012245). Data were processed using MestReNova 14.3.3-33362. Chemical shifts (δ) are reported in parts per million (ppm) relative to tetramethylsilane. ^1H, $^{13}C\{^1H\}$, COSY, HSQC and HMBC NMR spectra are referenced to residual solvent signals. ***Variable-temperature ^1H NMR (VT ^1H NMR) spectra*** were recorded on a Bruker Avance 400 MHz spectrometer (EOS) operating at a Larmor frequency of 400.13 MHz and equipped with a 5 mm BBFO probe. Spectra were acquired using Bruker TopSpin software. For the cleavage experiment, the sample temperature was maintained at 80 °C, and spectra were collected every 4 min using water suppression method (VT NMR method).

High resolution electrospray ionization-mass spectrometry (HRESI-MS) was carried out at the University of Wisconsin-Madison Department of Chemistry Paul Bender Chemical Instrumentation Center (CIC) using a Thermo Scientific Q Exactive Focus Orbitrap MS system, and at the University of Wisconsin-Madison School of Pharmacy Analytical Instrumentation Cluster using a Bruker MaXis Ultra-High Resolution Quadrupole Time-of-Flight MS system.

Ultraviolet-visible (UV-Vis) spectra were collected with the NanoDrop 1C instrument (AZY1706045). Spectra were recorded from 190 to 850 nm in a quartz cuvette with 1 cm path length. Copper titration and molar extinction measurements were made in ammonium acetate buffer (10 mM, pH 5.5).

Inductively coupled plasma-optical emission spectrometry (ICP-OES) *analysis* was carried out using an Agilent 5110 inductively coupled plasma optical emission spectrometer and an Agilent 4210 microwave plasma atomic emission spectrometer, respectively. For both techniques, a 6-point standard curve (1-100 ppm) with respect to zinc, gallium, iron, cobalt, indium, and copper was used and fits were found to be $R^2 > 0.99$.

Analytical- and radio-HPLC methods were carried out using a Agilent 1260 Infinity II system equipped with a binary gradient pump, UV-vis detector, and autoinjector. The instrument was set to detect UV absorption was recorded at 220 nm and 254 nm and was coupled to an in-line LabLogic Dual Scan-RAM detector and were controlled using the LabLogic Laura software package. Radio-HPLC analyses utilised a LabLogic 1" NaI photomultiplier tube detector with 2" lead shielding and radioTLC analyses utilised a LabLogic plastic photomultiplier tube detector.

Semipreparative HPLC was carried out using a Agilent 1260 Infinity II system equipped with a binary gradient pump, UV-vis detector, and manual injector. UV absorption was recorded at 220

and 254 nm. Flash chromatography was carried out using a Combi Flash Rf+ system with UV detection at 220 and 254 nm.

Liquid chromatography mass spectrometry (LCMS) was carried out on a Phenomenex Luna C18 column (5 µm, 150 mm × 3 mm, 100 Å, AXIA packed) at a flow rate of 0.8 mL/min using a single quadrupole Agilent 1260 Infinity II LC/MSD system equipped with a binary gradient pump, UV-vis detector, automatic injector, and an atmospheric pressure electrospray ionization (AP-ESI) source. Ultraviolet absorption was recorded at 220 nm and 254 nm, and positive and negative mass spectra were collected from m/z = 200 – 1200.

Positron emission tomography/computed tomography (PET/CT) imaging was performed using a Siemens Inveon Hybrid MicroPET/CT Scanner (Siemens Medical Solutions USA, Inc., Knoxville, TN). Mice were anesthetized with 4% isoflurane gas, and anesthesia was maintained during scans at 2% isoflurane in oxygen. CT scans were acquired prior to PET scans for anatomical coregistration as well as attenuation corrections. CT scan parameters were as follows: 220 rotation degrees, 120 rotation steps, binning factor of 4, exposure time of 250 ms, x-ray energy of 80 KVp, 1 mA current, and 105 µm resolution. PET scans were acquired with 40 million coincidence events per mouse, an energy window of 350 – 650 keV, and a timing window.

Single photon emission computed tomography/ computed tomography (SPECT/CT) imaging was performed using a MILabs U-SPECT/CT^{UHR} microsingle photon emission computed tomography/computed tomography (µSPECT/CT) system. Mice were anesthetized with 4% isoflurane gas, and anesthesia was maintained during scans at 2.5% isoflurane in oxygen. SPECT data were acquired using the General Purpose Rat/Mouse collimator (GP-RM; 1.5mm pinholes) with 4 frames in spiral scan mode using normal steps for a total of 40 minutes of effective acquisition time. SPECT data were reconstructed based on the 114 keV and 208 keV photopeaks with a 20% window of the photopeak, and background subtraction using the automatic triple energy windows (8.3 keV and 4.6 keV above/below the 20% width of the photopeak for the 208 keV and 114 keV photopeaks, respectively) into one static image with 0.4 mm isotropic vocels, SROSEM reconstruction with 128 subsets and 5 iterations, and a 1.4 mm full width half max (FWHM) smoothing filter.

Chromatography Solvent Systems:

Analytical HPLC - Method A: binary solvent system (A: water + 0.1% FA; B: MeCN + 0.1% FA); gradient (0 – 2 min: 5% B; 2 – 9 min: 5 – 40% B; 9 – 15 min: 40 – 95 % B; 15–16 min: 5% B; 16 – 17 min 95 – 5% B; 17 – 20 min: 5% B); flow rate: 0.8 mL/min; column: Phenomenex Luna C18 column (5 µm, 150 mm × 3 mm, 100 Å, AXIA packed).

Analytical HPLC - Method B: binary solvent system (A: water + 0.1% FA; B: MeCN + 0.1% FA); gradient (0 – 3 min: 5% B; 3 – 8 min: 5– 95% B; 8 – 11 min: 95% B; 11 – 13 min: 95 – 5% B; 13 – 16 min 5% B); flow rate: 0.8 mL/min; column: Phenomenex Luna C18 column (5 µm, 150 mm × 3 mm, 100 Å, AXIA packed).

Analytical HPLC - Method C: binary solvent system (A: water + 0.1% FA; B: MeCN + 0.1% FA); gradient (0 – 3 min: 5% B; 3 – 7 min: 5– 77% B; 7 – 7.30 min: 77 – 5% B; 7.30 – 10 min: 5% B); flow rate: 0.8 mL/min; column: Phenomenex Luna C18 column (5 µm, 150 mm × 3 mm, 100 Å, AXIA packed).

Analytical HPLC - Method D: binary solvent system (A: water + 0.1% FA; B: MeCN + 0.1% FA); gradient (0 – 2 min: 5% B; 2 – 8 min: 5– 20% B; 8 – 35 min: 20 – 95% B; 35 – 36 min: 5% B; 36 – 37 min: 95 – 5% B; 37 – 40 min: 5% B); flow rate: 0.8 mL/min; column: Phenomenex Luna C18 column (5 µm, 150 mm × 3 mm, 100 Å, AXIA packed).

Analytical HPLC - Method E: binary solvent system (A: 10 mM ammonium acetate in water; B: 90% MeCN + 10% 10 mM ammonium acetate in water); gradient (0 – 1 min: 0% B; 1 – 11 min: 5 – 95% B; 11 – 13 min: 95% B; 13 – 14 min: 95 – 5% B; 14 – 16 min: 5% B); flow rate: 0.7 mL/min; column: Phenomenex Luna C18 column (5 µm, 150 mm × 3 mm, 100 Å, AXIA packed).
Radio HPLC – Method F: binary solvent system (A: water + 0.1% TFA; B: MeCN + 0.1% TFA); gradient (0 – 2 min: 5% B; 2 – 14 min: 5 – 95% B; 14 – 16 min: 95% B; 16 – 16.50 min: 95 – 5% B; 16.50 – 20 min: 5% B); flow rate: 0.8 mL/min; column: Phenomenex Luna C18 column (5 µm, 150 mm × 3 mm, 100 Å, AXIA packed).
Semi-preparative HPLC - Method G: binary solvent system (A: water + 0.1% FA; B: MeCN + 0.1% FA); gradient: (0–3 min: 5% B; 3–17 min: 5–50% B; 17–21 min: 50–95% B; 21–25 min: 95% B; 25–27 min: 95–5% B; 27–30 min: 5% B); flow rate: 10 mL/min; column: Phenomenex Luna C18 column (250 mm × 21.2 mm, 100 Å, AXIA packed).
Flash chromatography - Method H: Binary solvent system (A: water + 0.1% TFA; B: MeCN + 0.1% TFA); gradient (0 – 1 min: 0% B; 1 – 16 min: 0 – 100 % B; 16 – 19 min: 100% B; 19 min: 100 – 5% B; 19 – 20 min: 5% B); flow rate: 40 mL/min; column: RediSep C18 column (50 g HP gold).
LCMS - Method I: binary solvent system (A: water + 0.1% FA; B: MeCN + 0.1% FA); gradient (0-3 min: 5% B; 3-10 min: 5-95% B; 10-13 min: 95% B; 13-13.5 min: 95-5% B; 13.5-16 min: 5% B); flow rate: 0.8 mL/min; column: Phenomenex Luna C18 column (5 µm, 150 mm × 3 mm, 100 Å, AXIA packed).

3 Synthesis and Characterization of Model Peptides

3.1 Synthesis of Model Tripeptides

The model tripeptides **1** – **5** were synthesized according to previously reported procedure.[1]
The canonical amino acid model compound **6** was synthesized according to reported procedure.[2]
The noncanonical amino acid model compound **7** was synthesized according to reported procedure.[3]

3.2 HRMS, HR-ESI-MS and HPLC Characterization Data

NOTA-G-G-W-CONH$_2$, 1. Compound **1** was isolated as a white solid following the deprotection and purification using semi-preparative HPLC (Method G) and characterized using ESI-MS and HPLC chromatography. Yield: 39%. R_t (Method I): 6.95 min. ^{1}H NMR (500 MHz, D_2O) δ 7.69 (d, 1H), 7.53 (d, 1H), 7.31 – 7.23 (m, 2H), 7.19 (t, 1H), 4.72 (t, 1H), 3.98 (d, 2H), 3.94 – 3.81 (m, 2H), 3.72 – 3.53 (m, 4H), 3.48 (d, 2H), 3.39 – 2.68 (m, 14H). ^{13}C NMR (126 MHz, D_2O): δ 176.43 ($C^{21,19}$), 174.40 (C^{10}), 171.86 (C^{12}), 171.49 ($C^{14,16}$), 136.12 (C^{1}), 126.90 (C^{6}), 123.99 (C^{7}), 122.03 (C^{3}), 119.39 (C^{4}), 118.39 (C^{5}), 111.88 (C^{2}), 109.25 (C^{8}), 59.02 (C^{17}), 56.83 (C^{18}), 53.85 (C^{20}), 50.63 ($C^{cycl.}$), 49.07 ($C^{cycl.}$), 48.26 ($C^{cycl.}$), 42.53 (C^{13}), 42.35 (C^{15}), 26.77 (C^{19}). HR-ESI-MS $[M+H]^+$ calc. for $C_{27}H_{38}N_8O_8$ 603.2885, found 603.2884.

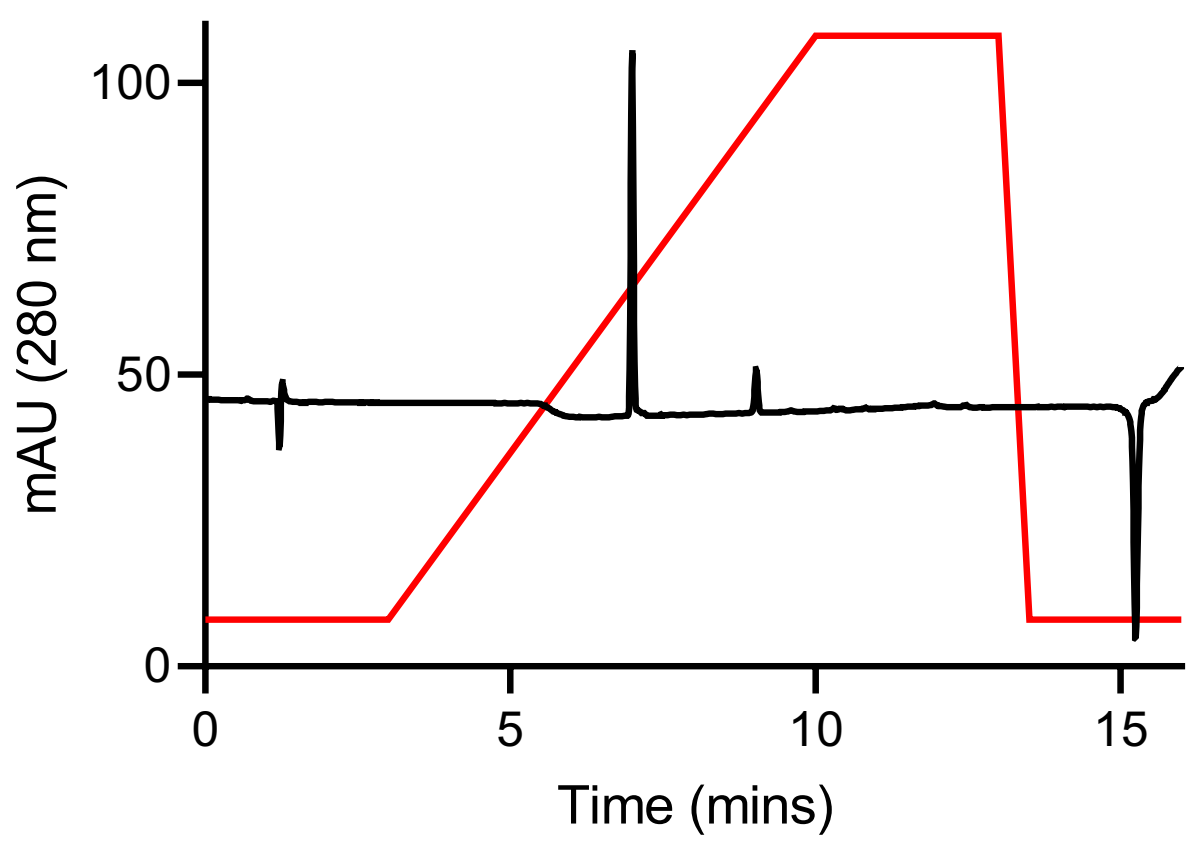

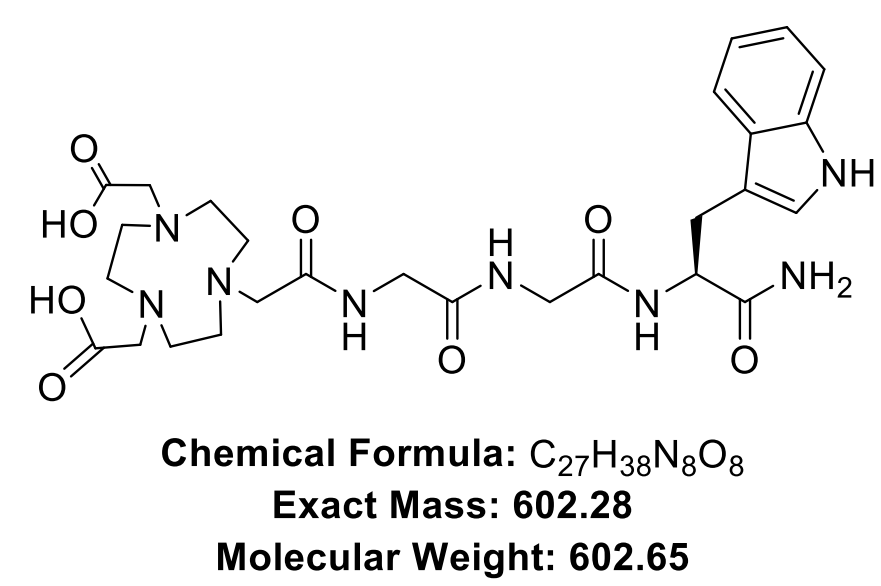

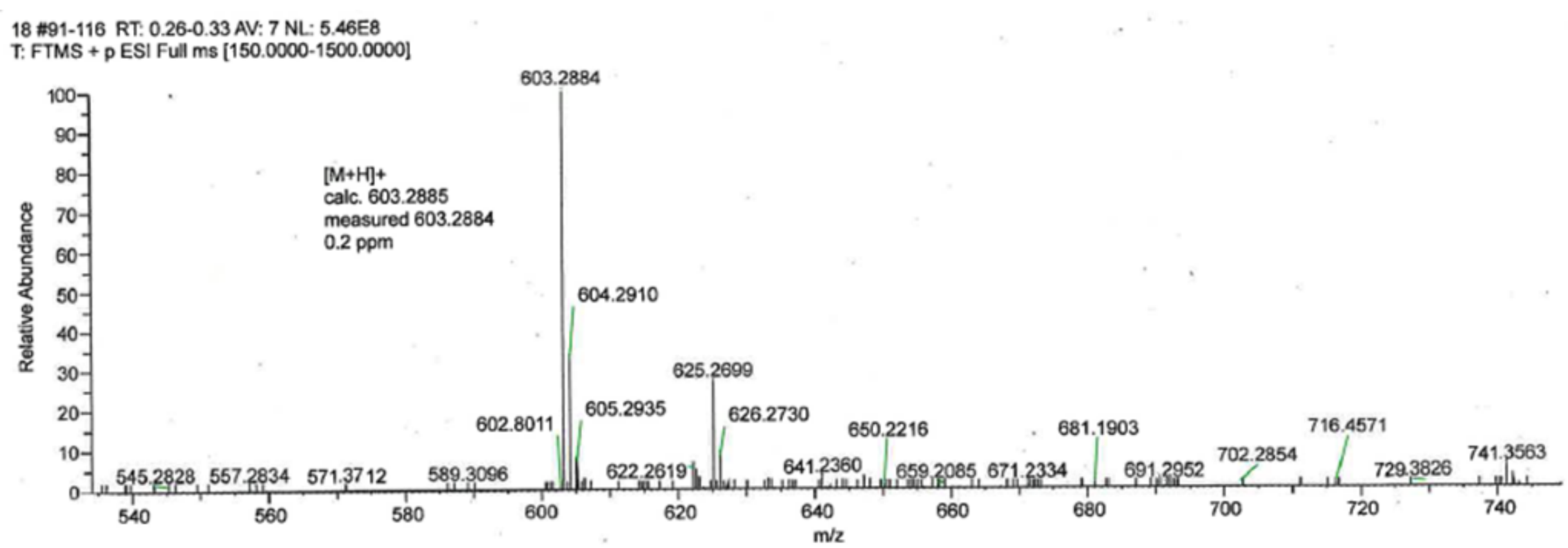

Figure S1. Characterization data of compound **1.**

NOTA-^{NMe}G-G-W-CONH$_2$, 2. Compound **2** was isolated as a white solid following purification using semi-preparative HPLC (Method G) and characterized using ESI-MS and HPLC chromatography. Yield: 36%. R_t (Method I): 6.61 min. ^{1}H NMR (500 MHz, D_2O) δ 7.72 (d, 1H), 7.55 (d, 1H), 7.35 – 7.26 (m, 2H), 7.21 (t, 1H), 4.74 – 4.67 (m, 1H), 4.19 – 4.00 (m, 2H), 4.00 – 3.88 (m, 2H), 3.81 (s, 2H), 3.70 – 3.49 (m, 4H), 3.45 – 3.03 (m, 9H), 3.00 (s, 2H), 2.91 (s, 1H). ^{13}C NMR (126 MHz, D2O) δ 176.21, 173.93, 171.75, 171.18, 170.79, 136.11, 126.92, 124.37, 122.01, 119.42, 118.39, 111.91, 109.00, 58.65, 55.40, 53.91, 51.83, 51.65, 50.19, 49.18, 42.46, 35.75, 35.33, 26.93.HR-ESI-MS $[M+H]^+$ calc. for $C_{28}H_{40}N_8O_8$ 617.3042, found 617.3035.

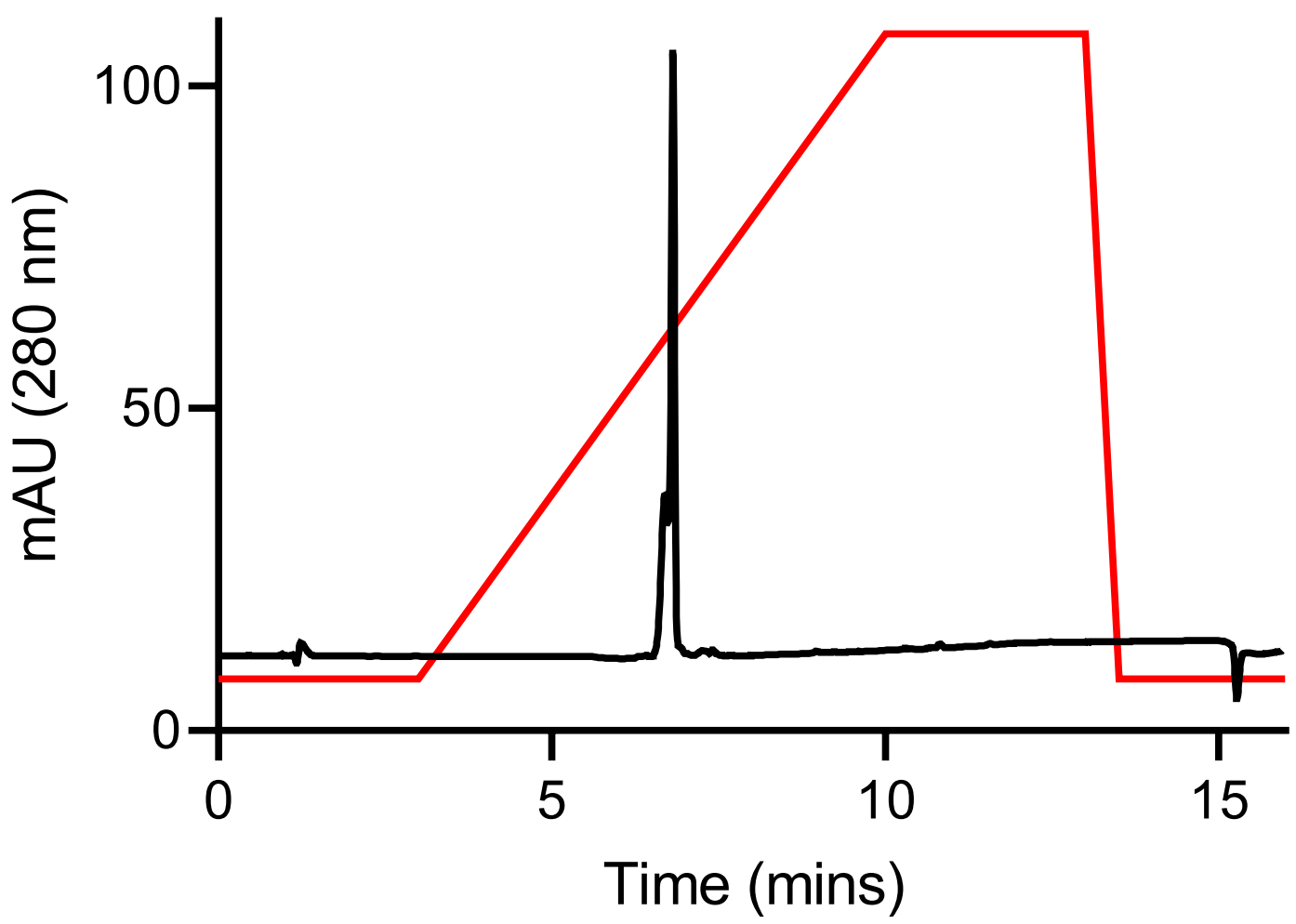

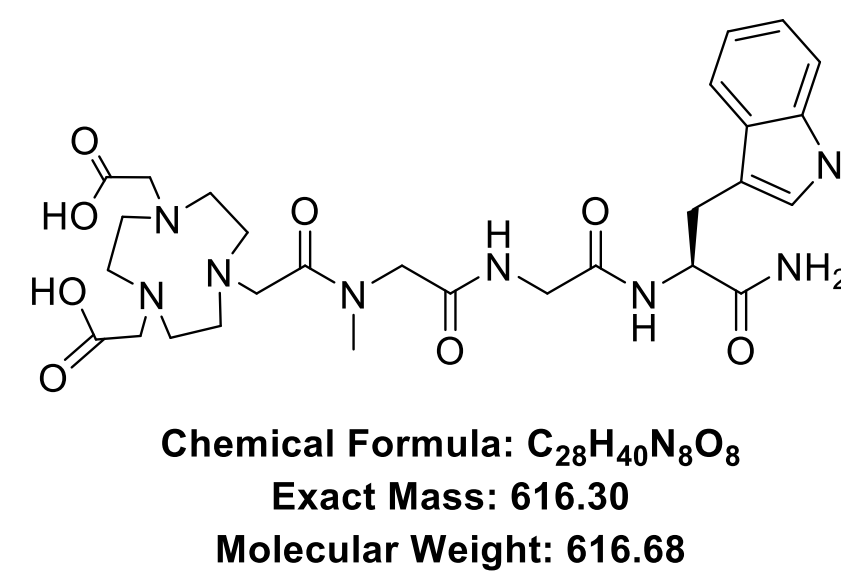

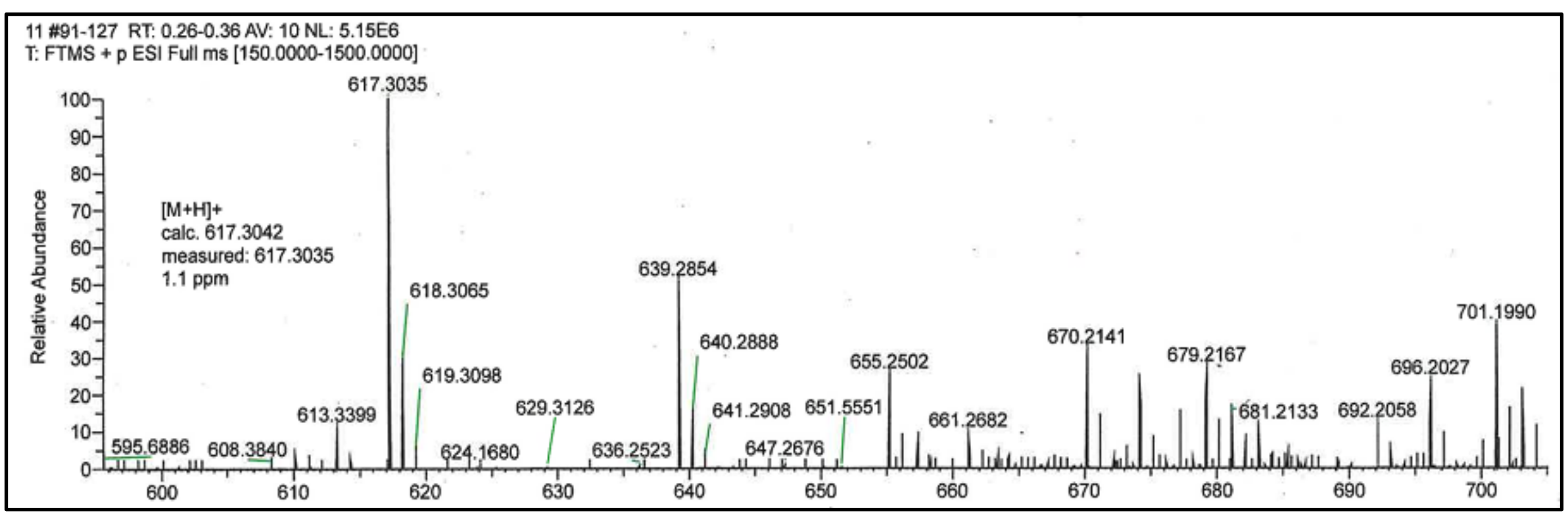

Figure S2. Characterization data for compound **2.**

NOTA-^{NMe}S-G-W-CONH$_2$, 3. Compound **3** was isolated as a white solid following purification using semi-preparative HPLC (Method G) and characterized using ESI-MS and HPLC chromatography. Yield: 36%. R_t (Method I): 6.93 min. ^{1}H NMR (500 MHz, D_2O): δ 7.61 (d, 1H), 7.49 – 7.43 (d, 1H), 7.39 – 7.16 (m, 2H), 7.15 – 7.09 (t, 1H), 4.82 – 4.76 (m, 1H), 4.61 (dd, 1H), 3.89 – 3.83 (m, 2H), 3.80 – 3.66 (m, 8H), 3.50 (s, 4H), 3.33 – 3.12 (m, 6H), 3.07 (d, 4H), 2.78 (s, 3H). ^{13}C NMR (126 MHz, D_2O): δ 176.21 ($C^{21,23}$), 174.43 (C^{10}), 171.32 ($C^{12,14}$), 171.22 (C^{18}), 136.12 (C^1), 126.98 (C^6), 124.27 (C^7), 122.04 (C^3), 119.45 (C^4), 118.42 (C^5), 111.91 (C^2), 109.02 (C^8), 61.04 (C^{15}), 58.75 ($C^{20,22}$), 58.02 (C^{16}), 55.67 (C^{19}), 53.85 (C^{11}), 51.91 ($C^{cylc.}$), 50.22 ($C^{cycl.}$), 49.21 ($C^{cycl.}$), 42.51 (C^{13}), 31.75 (C^{17}), 26.94 (C^9). HR-ESI-MS $[M+H]^+$ calc. for $C_{29}H_{42}N_8O_9$ 647.3147, found 647.3142.

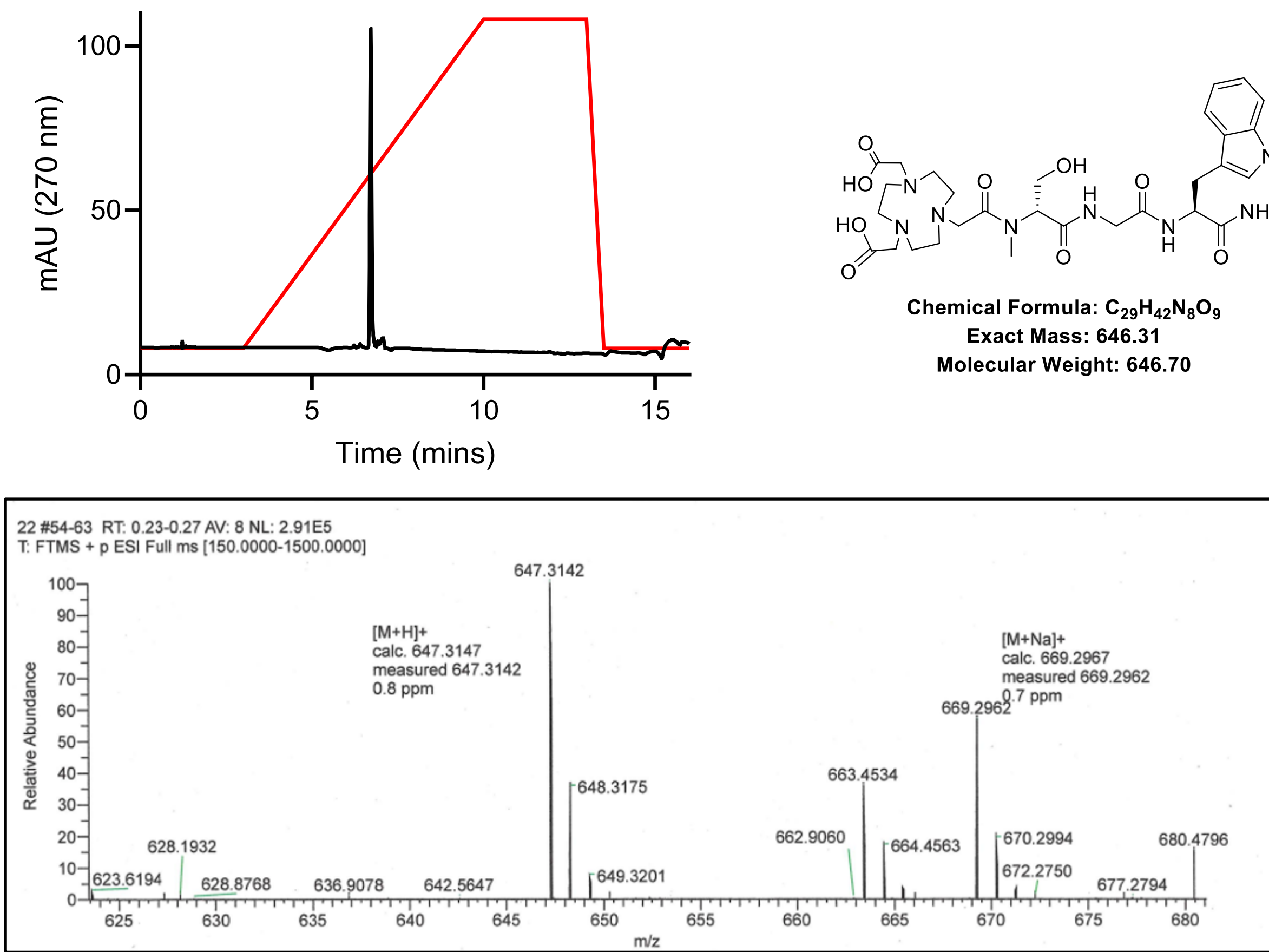

Figure S3. Characterization data for compound **3**.

NOTA-[NMe]M-G-W-CONH$_2$, 4. Compound **4** was isolated as a white solid following purification using semi-preparative HPLC (Method G) and characterized using ESI-MS and HPLC chromatography. Yield: 36%. R_t (Method I): 6.93 min. ^{1}H NMR (500 MHz, D_2O) δ 7.69 (d, 1H), 7.55 (d, 1H), 7.33 – 7.24 (m, 2H), 7.21 (t, 1H), 5.03 (dd, 1H), 4.75 – 4.68 (m, 1H), 4.16 – 3.90 (m, 2H), 3.94 – 3.82 (m, 6H), 3.55 – 3.28 (m, 14H), 2.76 (s, 3H), 2.54 – 2.36 (m, 2H), 2.10 (s, 3H), 2.01 – 1.88 (m, 1H). ^{13}C NMR (126 MHz, D_2O): δ 176.14 ($C^{23,25}$), 172.26 (C^{10}), 172.15 (C^{12}), 171.66 (C^{14}), 170.83 (C^{20}), 136.12 (C^1), 126.98 (C^6), 124.32 (C^7), 122.04 (C^3), 119.46 (C^4), 118.38 (C^5), 111.92 (C^5), 108.84 (C^8), 57.65 ($C^{22,24}$), 56.99 (C^{15}), 56.34 (C^{21}), 53.73 (C^{11}), 51.09 (C^{cycl}), 50.41 ($C^{cycl.}$), 42.66 (C^{13}), 30.60 (C^{19}), 29.48 (C^{17}), 26.96 (C^9), 26.06 (C^{18}), 14.10 (C^{16}). HR-ESI-MS $[M-H]^-$ calc. for $C_{31}H_{46}N_8O_8S$ 689.3087, found 689.3094.

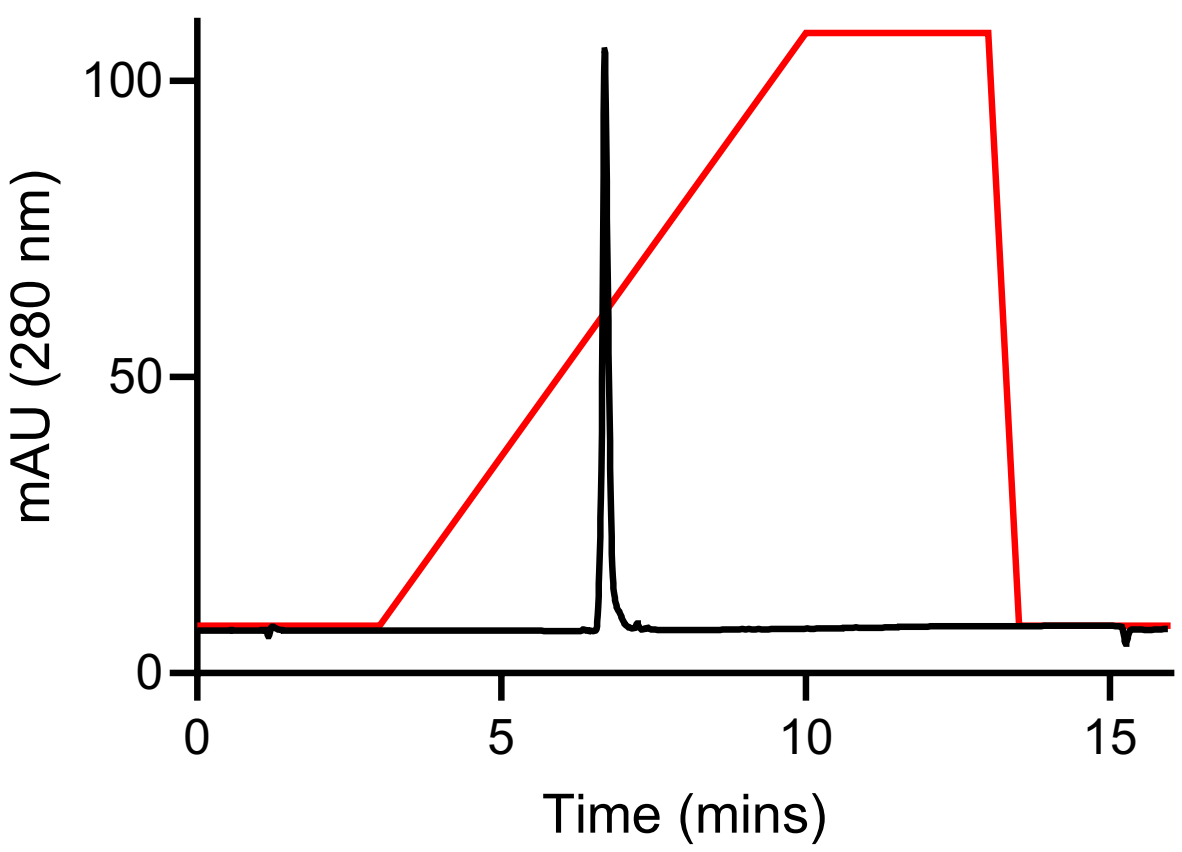

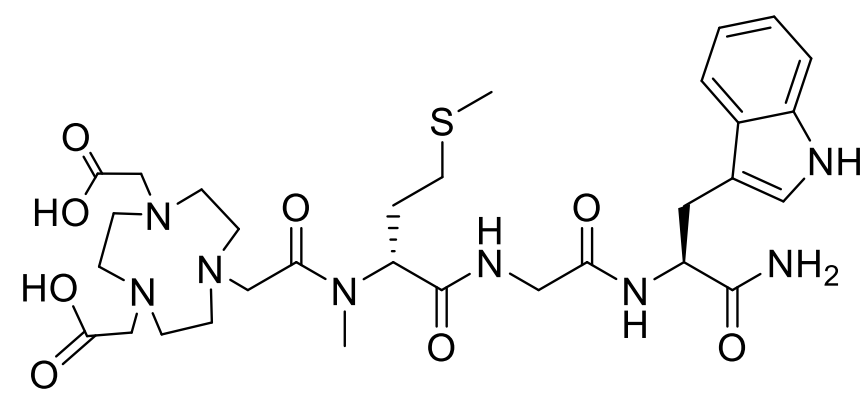

Chemical Formula: $C_{31}H_{46}N_8O_8S$
Exact Mass: 690.32
Molecular Weight: 690.82

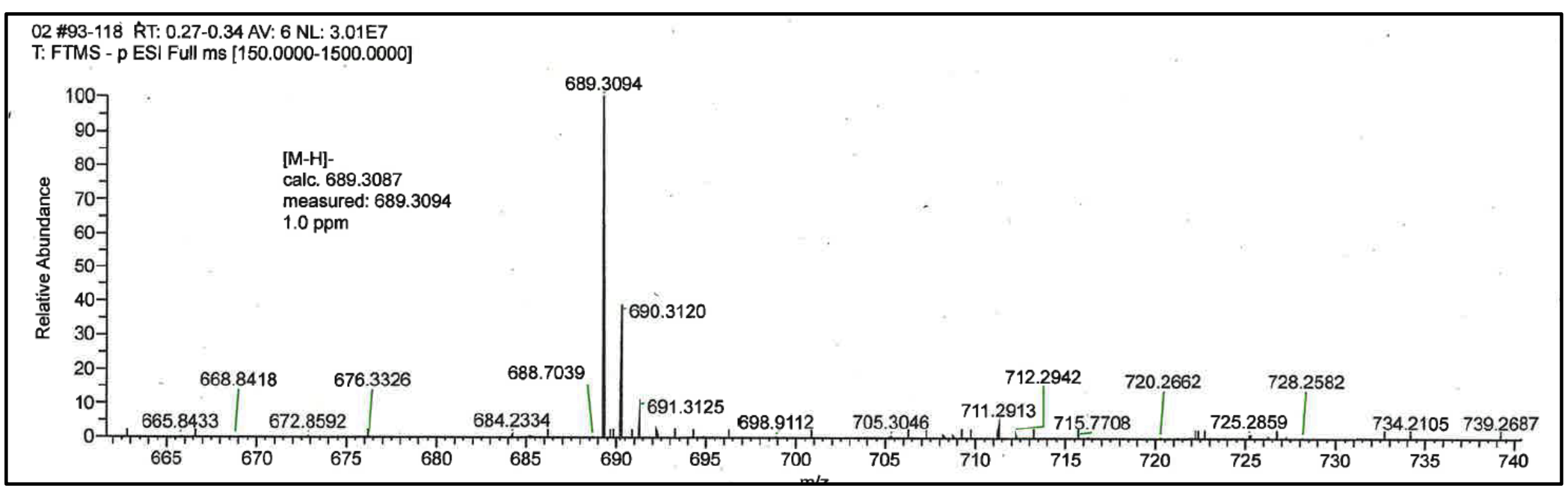

Figure S4. Characterization data for compound **4.**

NOTA-A-G-W-CONH$_2$, 5. Compound **5** was isolated as a white solid following the deprotection and purification using semi-preparative HPLC (Method G) and characterized using ESI-MS and HPLC chromatography. Yield: 39%. R_t (Method I): 6.34 min. ^{1}H NMR (500 MHz, D_2O) δ 7.68 (d, 1H), 7.52 (d, 1H), 7.27 (t, 1H), 7.24 – 7.16 (m, 2H), 4.70 (dd, 1H), 4.28 (q, 1H), 4.08 – 3.84 (m, 2H), 3.70 (s, 4H), 3.47 – 3.32 (m, 3H), 3.30 – 3.15 (m, 5H), 3.00 (s, 4H), 2.76 (s, 4H), 1.33 (d, 3H). ^{13}C NMR (126 MHz, D_2O) δ 176.25 (C^{20}), 175.64 (C^{22}), 173.56 (C^{10}), 172.52 ($C^{12,14}$), 171.42 (C^{17}), 136.08 (C^{1}), 126.96 (C^{6}), 123.89 (C^{7}), 122.04 (C^{3}), 119.39 (C^{4}), 118.41 (C^{5}), 111.88 (C^{2}), 109.29 (C^{8}), 58.15 (C^{18}), 57.08 ($C^{19,21}$), 53.89 (C^{101}), 50.82 ($C^{cycl.}$), 49.95 ($C^{cycl.}$), 49.27 ($C^{cycl.}$), 48.15 (C^{15}), 42.56 (C^{13}), 26.92 (C^{9}), 16.07 (C^{16}).HR-ESI-MS $[M-H]^-$ calc. for $C_{28}H_{40}N_8O_8$ 615.2896, found 615.2905.

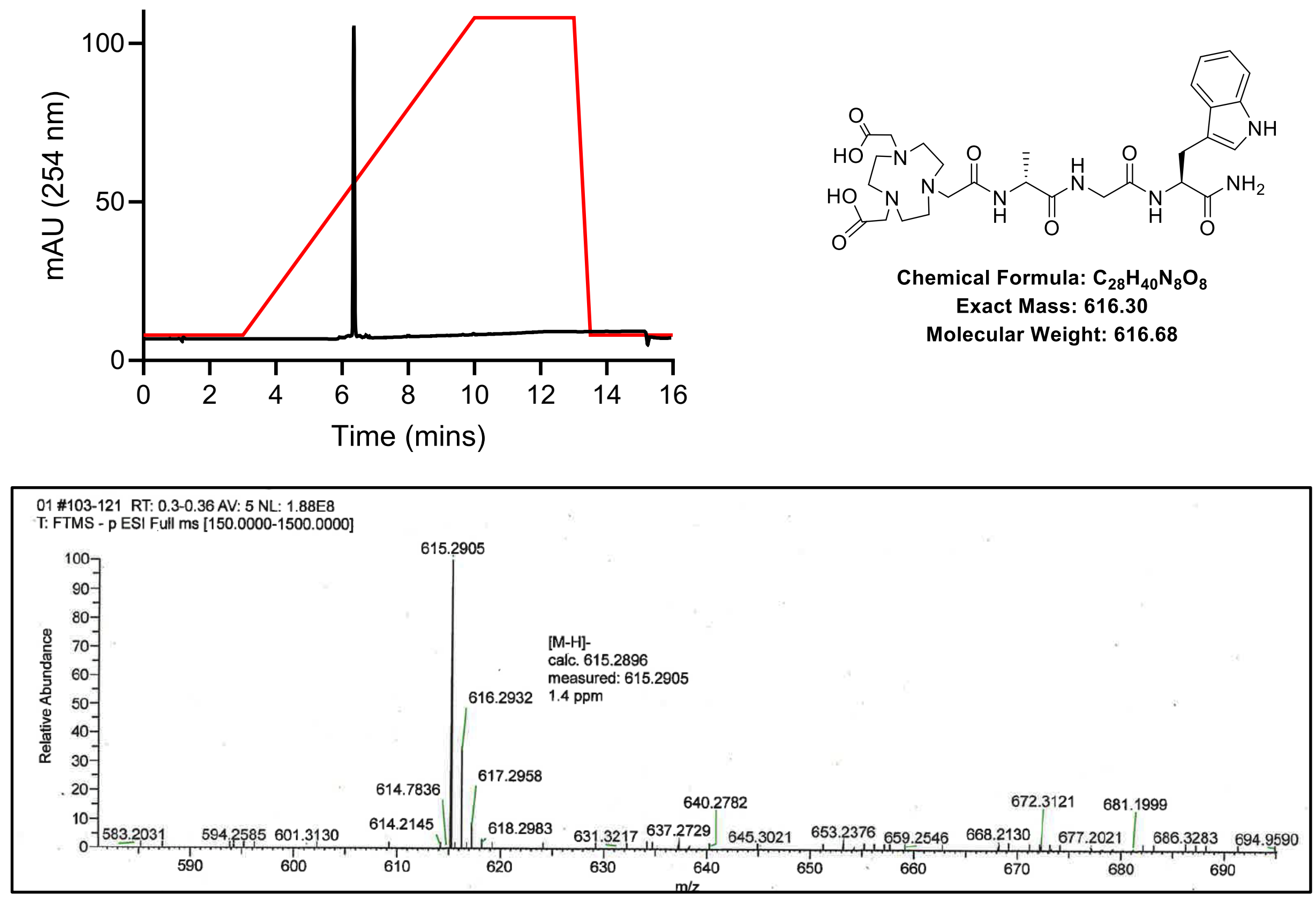

Figure S5. Characterization data of compound **5.**

Synthesis of canonical amino acid model compound, 6. ^{1}H NMR (500 MHz, D_2O) δ 7.45 – 7.18 (m, 5H), 4.37 (s, 2H), 3.79 – 3.64 (m, 6H), 3.37 – 2.96 (m, 12H). ^{13}C NMR (126 MHz, D_2O) δ 171.90, 171.33, 137.70, 128.88, 127.66, 127.37, 58.79, 56.05, 50.61, 49.72, 49.62, 43.05.

Synthesis of noncanonical amino acid model compound, 7. ^{1}H NMR (500 MHz, MeOD) δ 7.46 – 7.14 (m, 5H), 4.88 (s, 5H), 4.59 (d, J = 24.9 Hz, 2H), 4.16 (d, J = 11.8 Hz, 2H), 3.73 (s, 4H), 3.29 – 2.97 (m, 12H), 2.95 (d, J = 11.9 Hz, 3H). ^{13}C NMR (126 MHz, MeOD) δ 172.18, 172.11, 167.94, 167.73, 136.57, 135.93, 128.72, 128.36, 127.67, 127.51, 127.25, 126.43, 55.16, 55.05, 54.59, 54.55, 51.92, 51.13, 50.97, 50.69, 49.70, 32.97, 32.79.

3.3 NMR Spectra of Peptides

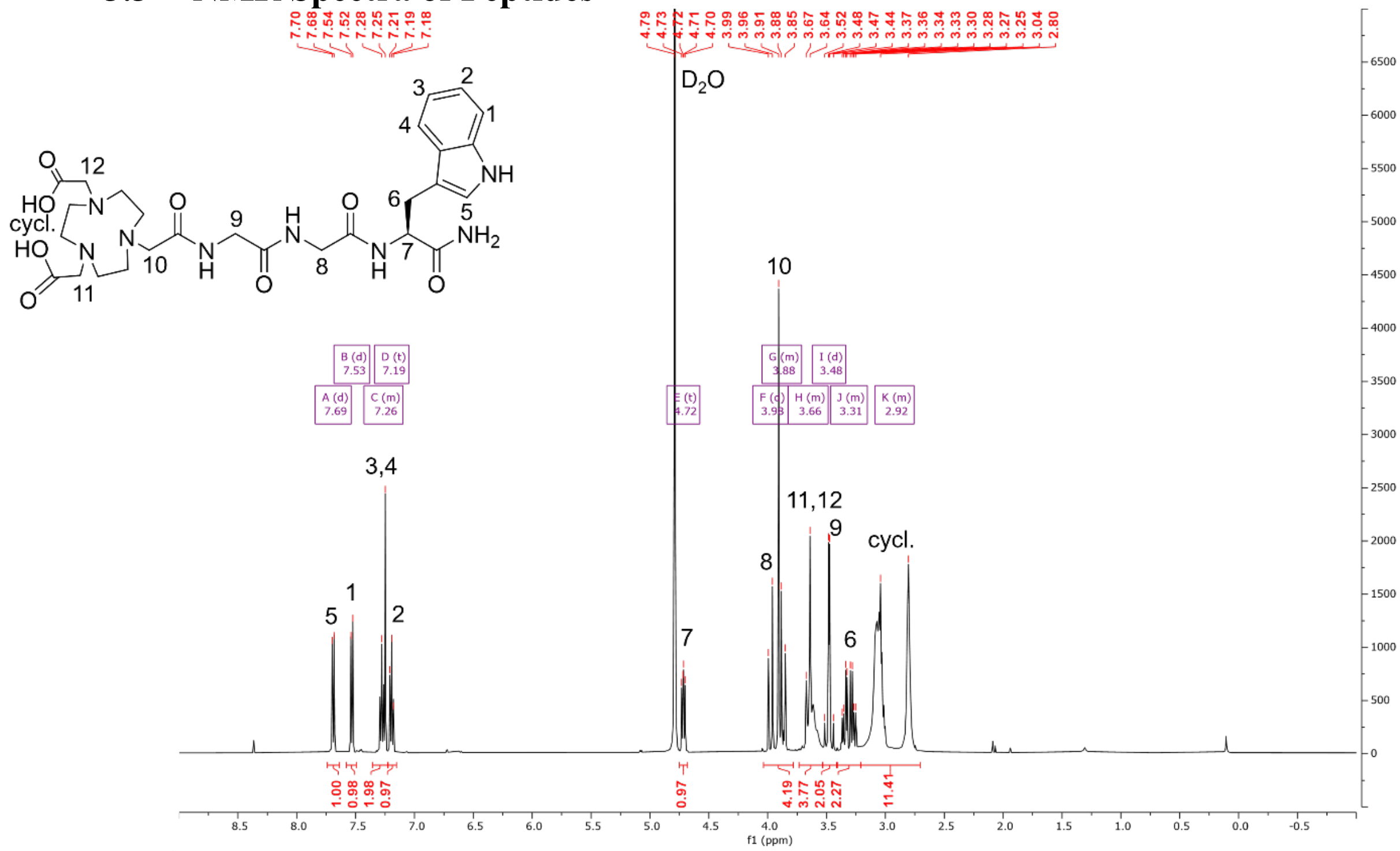

Figure S6. ^{1}H NMR (500 MHz, D_2O) for compound **1**.

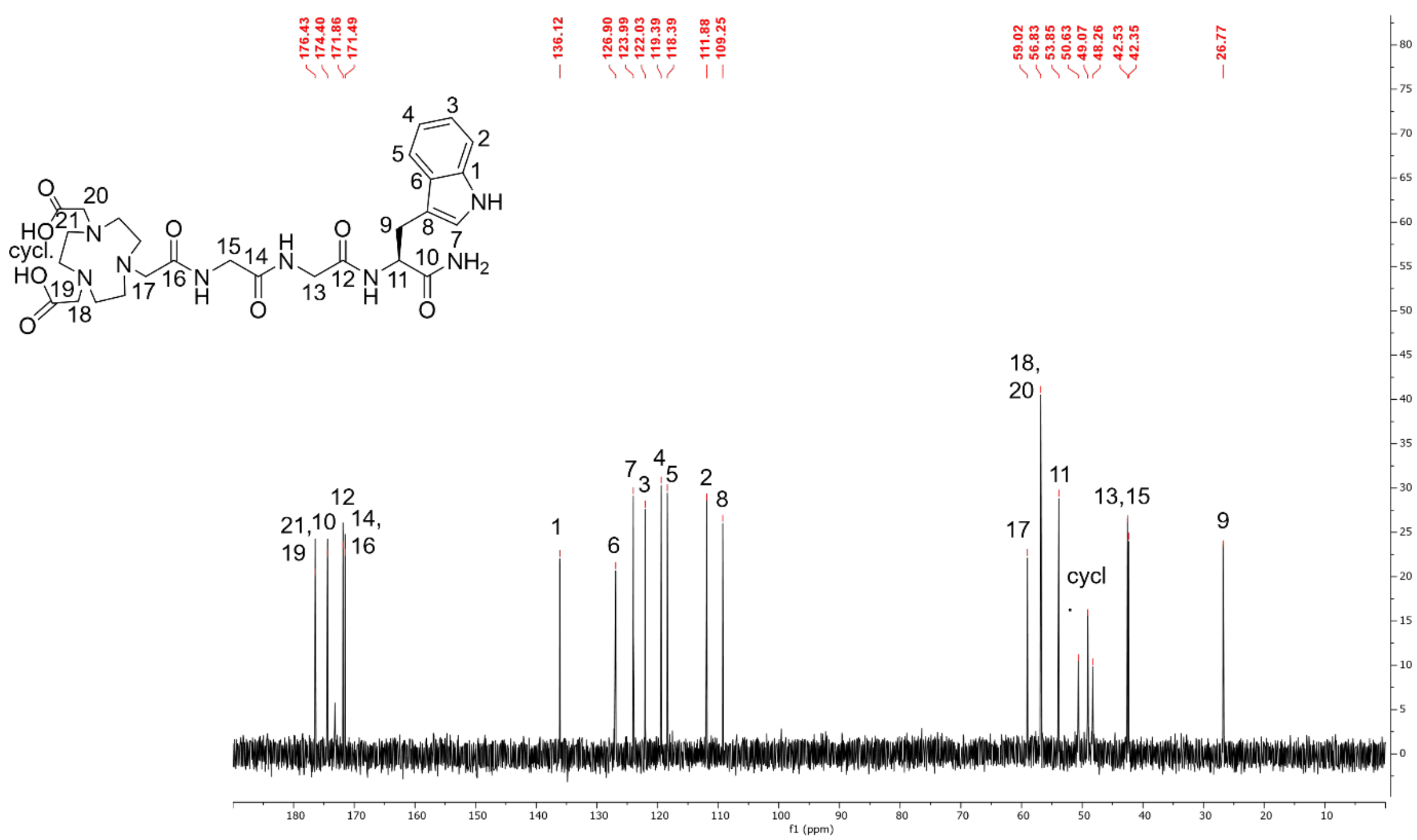

Figure S7. ^{13}C NMR (126 MHz, D_2O) for compound **1**.

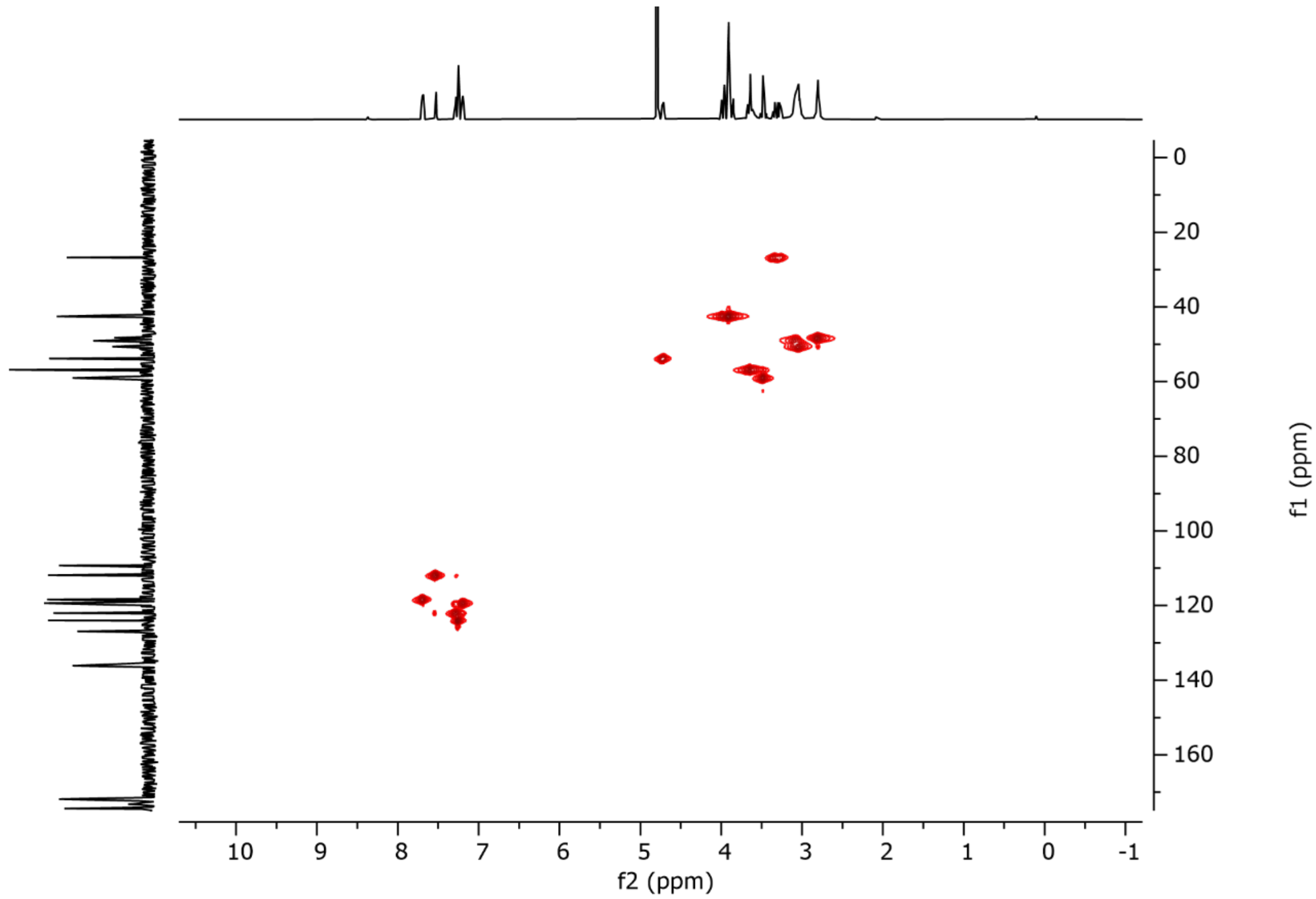

Figure S8. The ^{1}H-^{13}C HSQC NMR spectrum of compound **1** in D_2O.

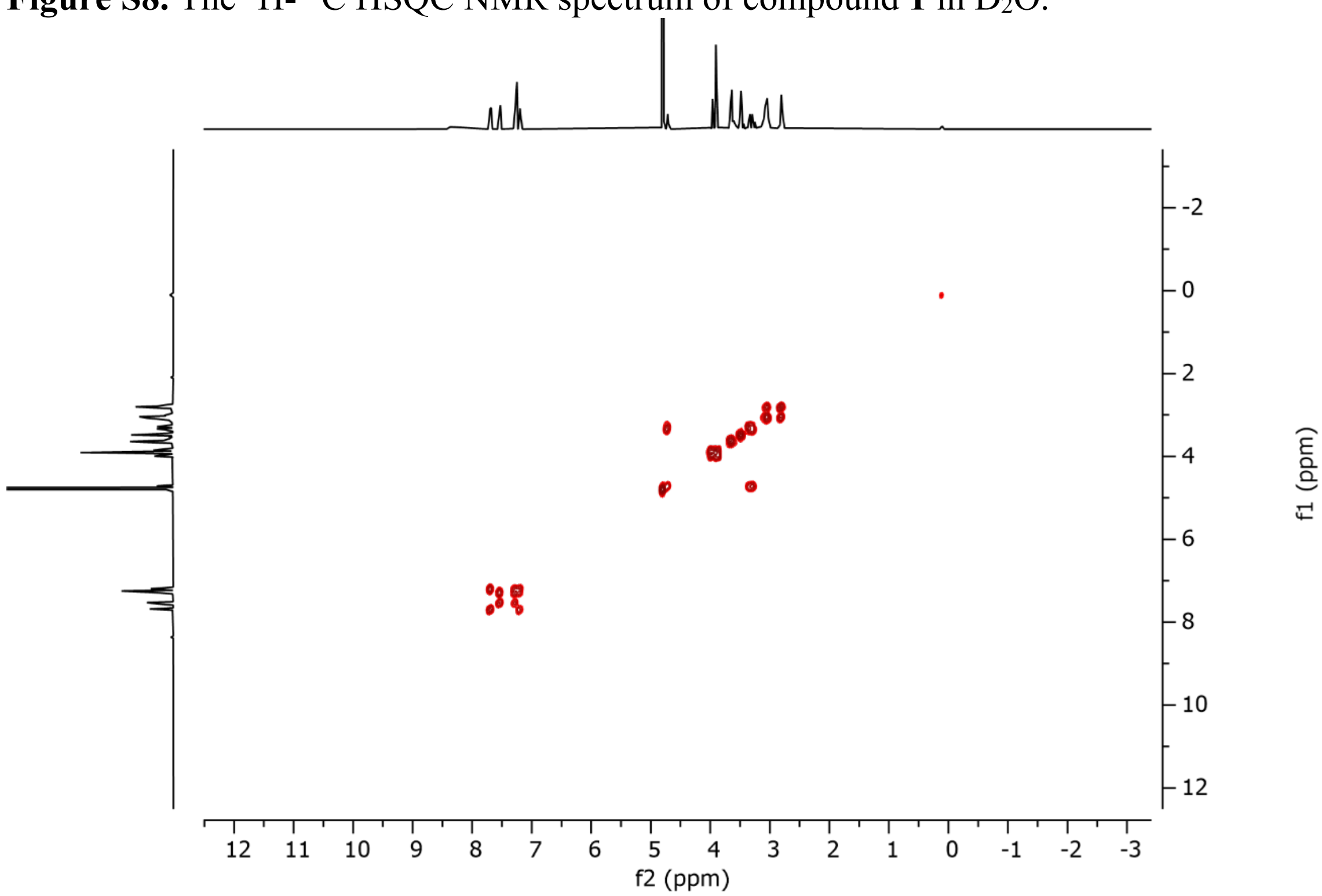

Figure S9. The ^{1}H-^{1}H COSY NMR spectrum of compound **1** in D_2O.

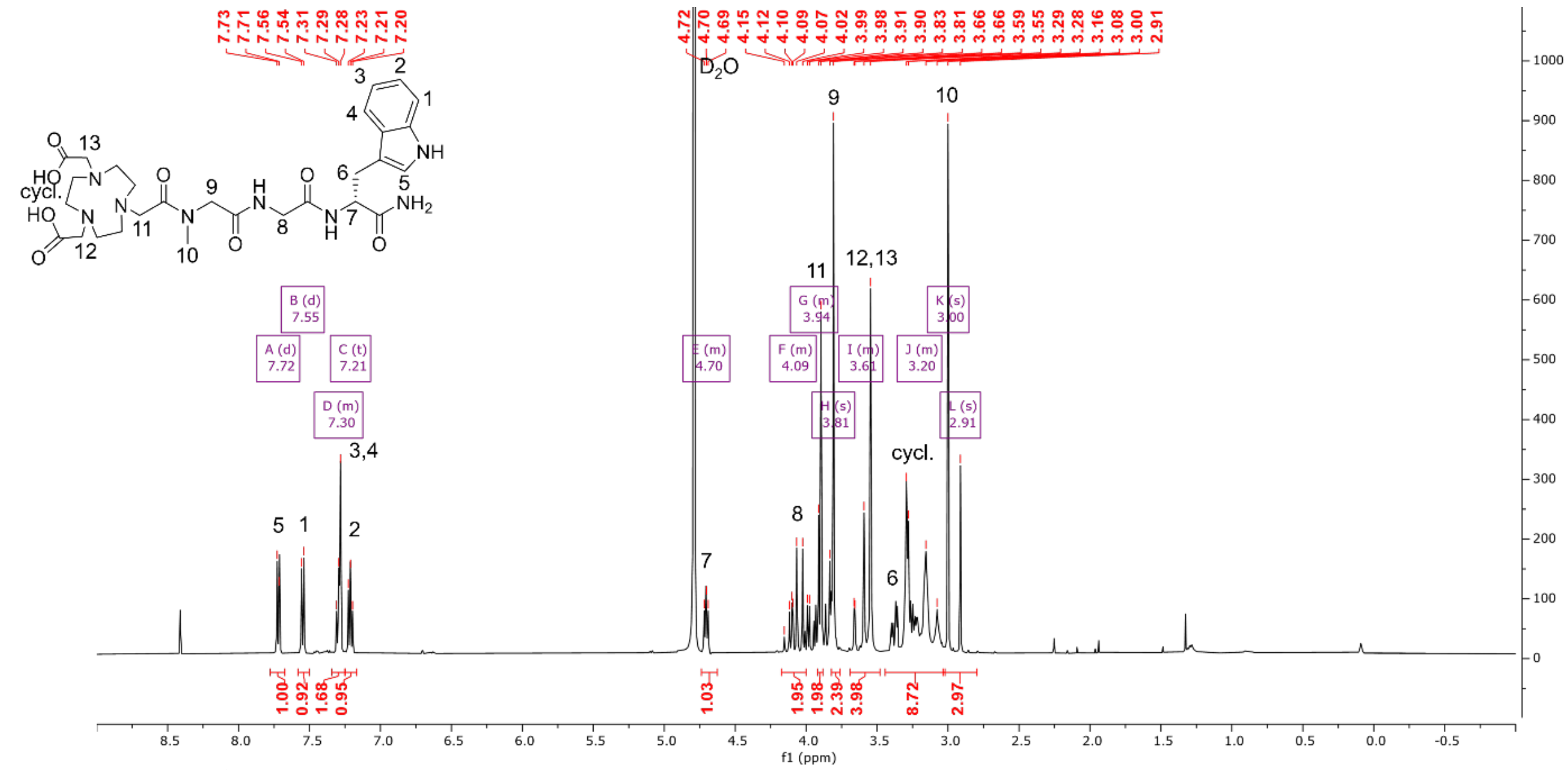

Figure S10. ^{1}H NMR (500 MHz, D_2O) for compound **2**.

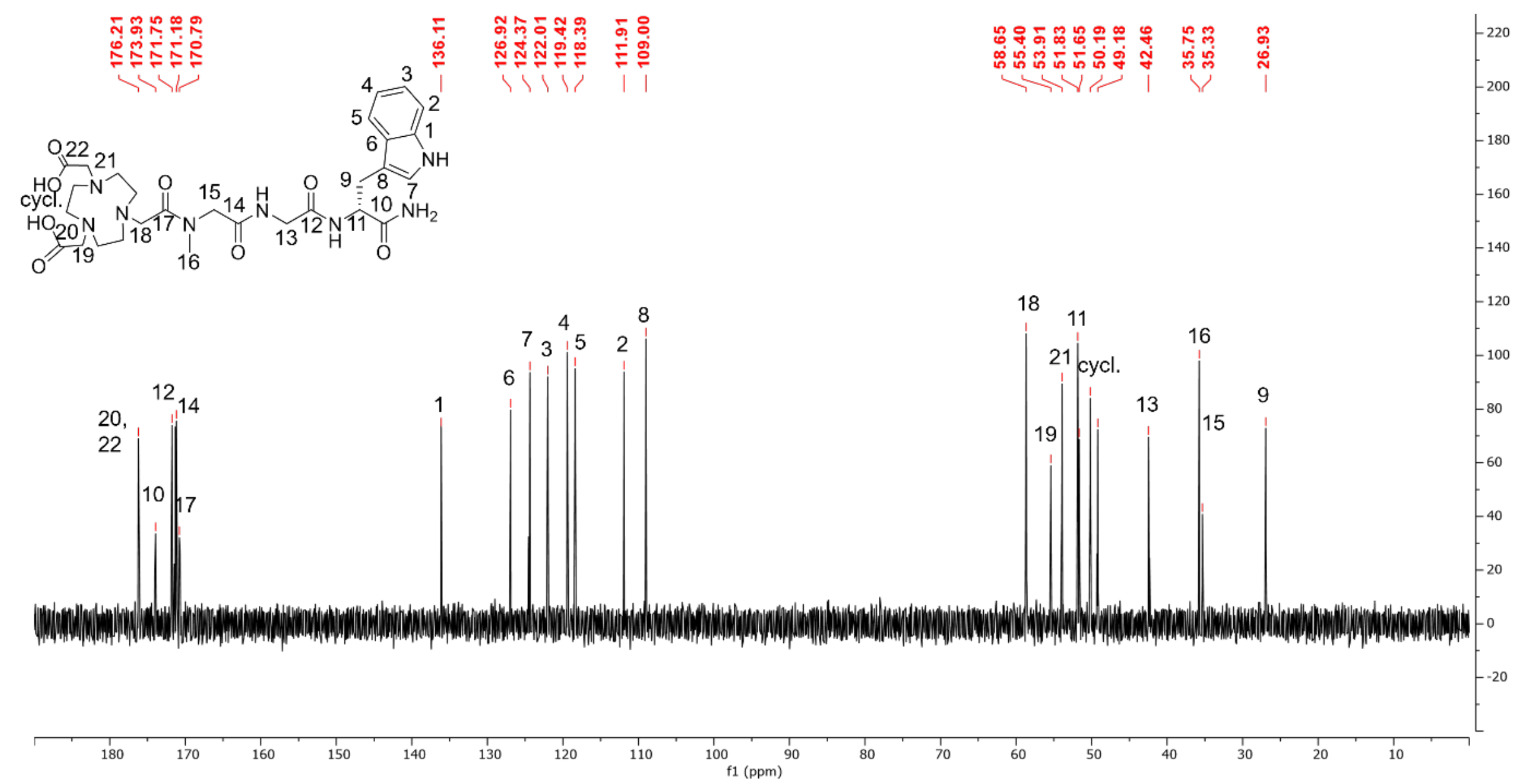

Figure S11. ^{13}C NMR (126 MHz, D_2O) for compound **2**.

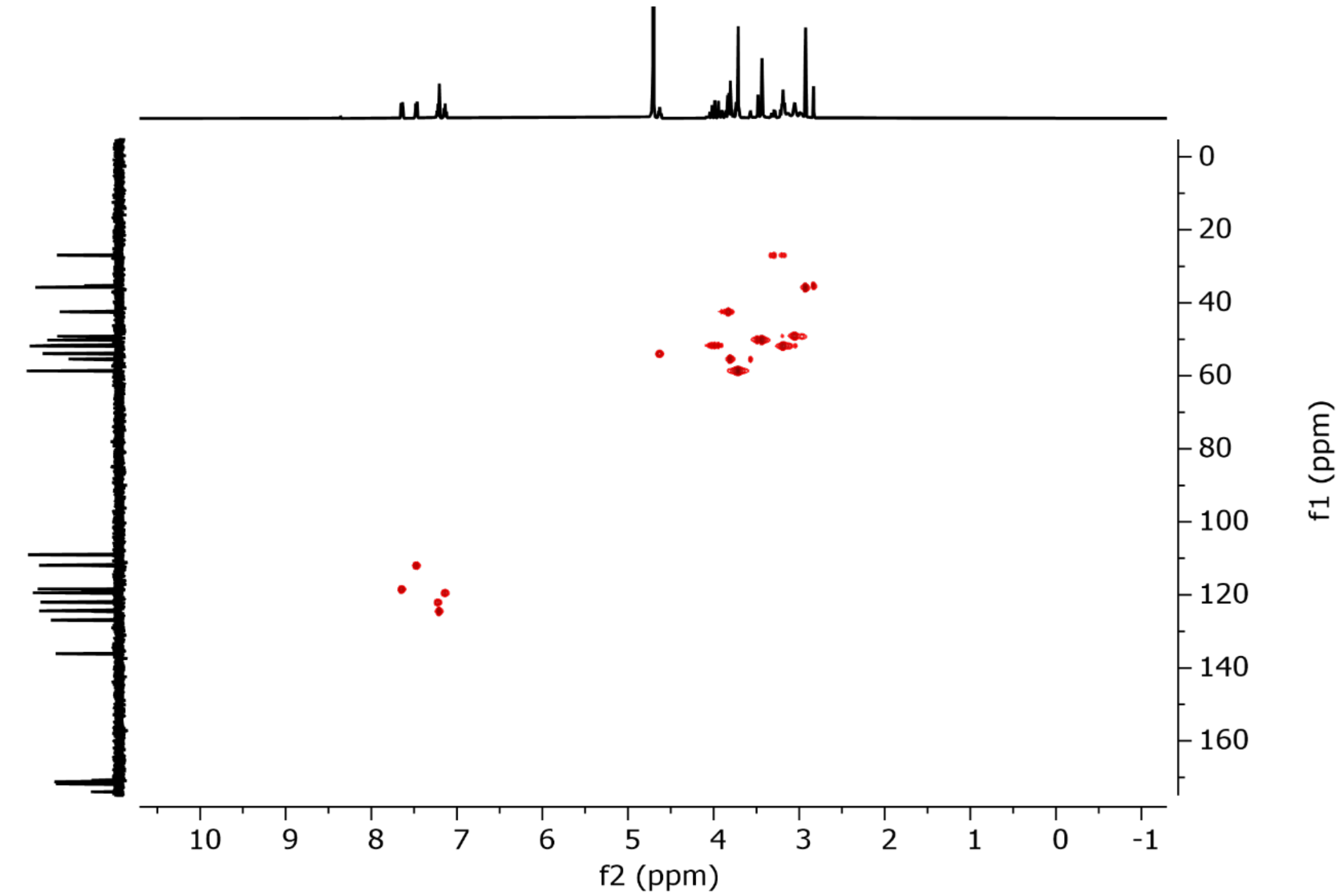

Figure S12. The ^{1}H-^{13}C HSQC NMR spectrum for compound **2**.

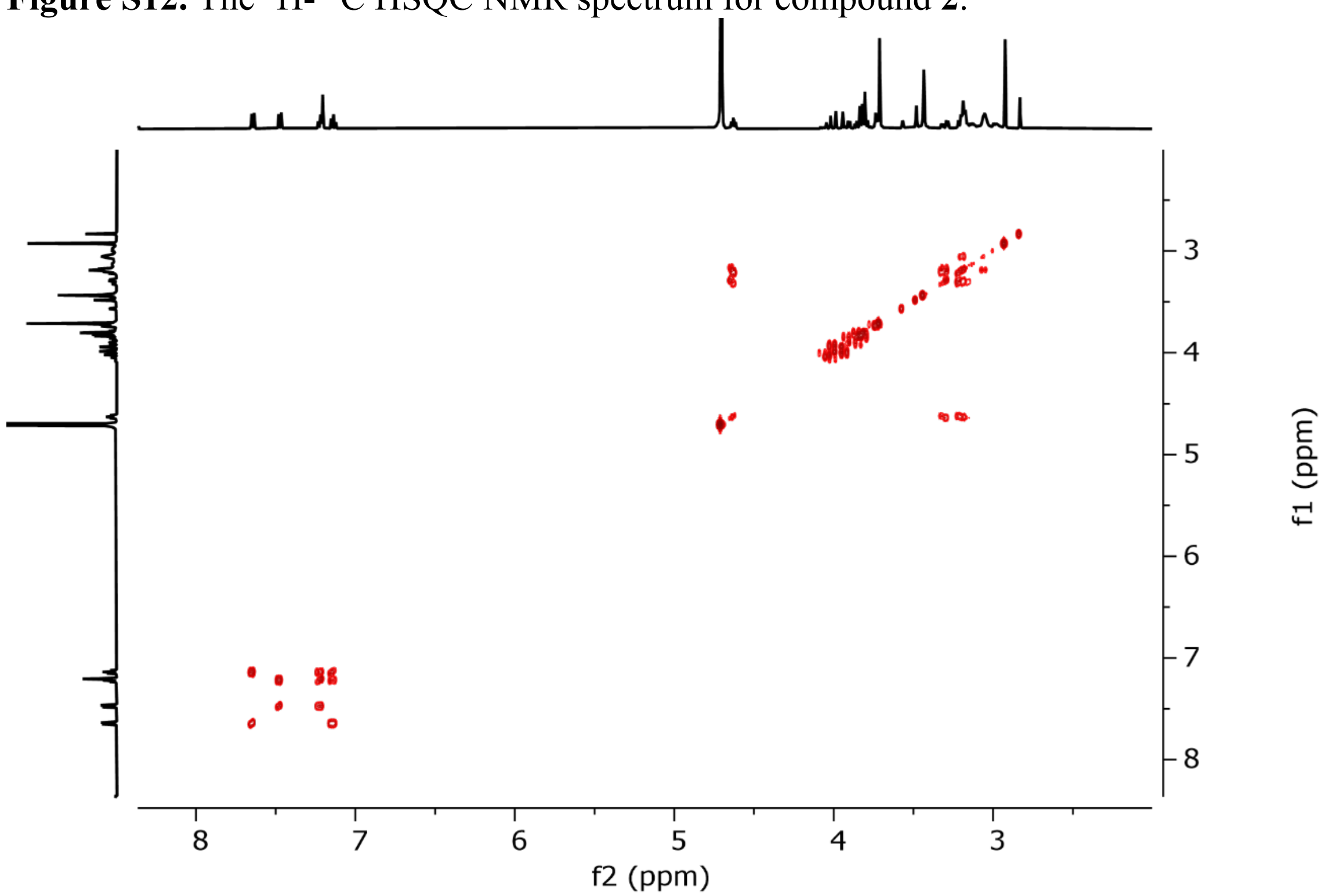

Figure S13. The ^{1}H-^{1}H COSY NMR spectrum for compound **2**.

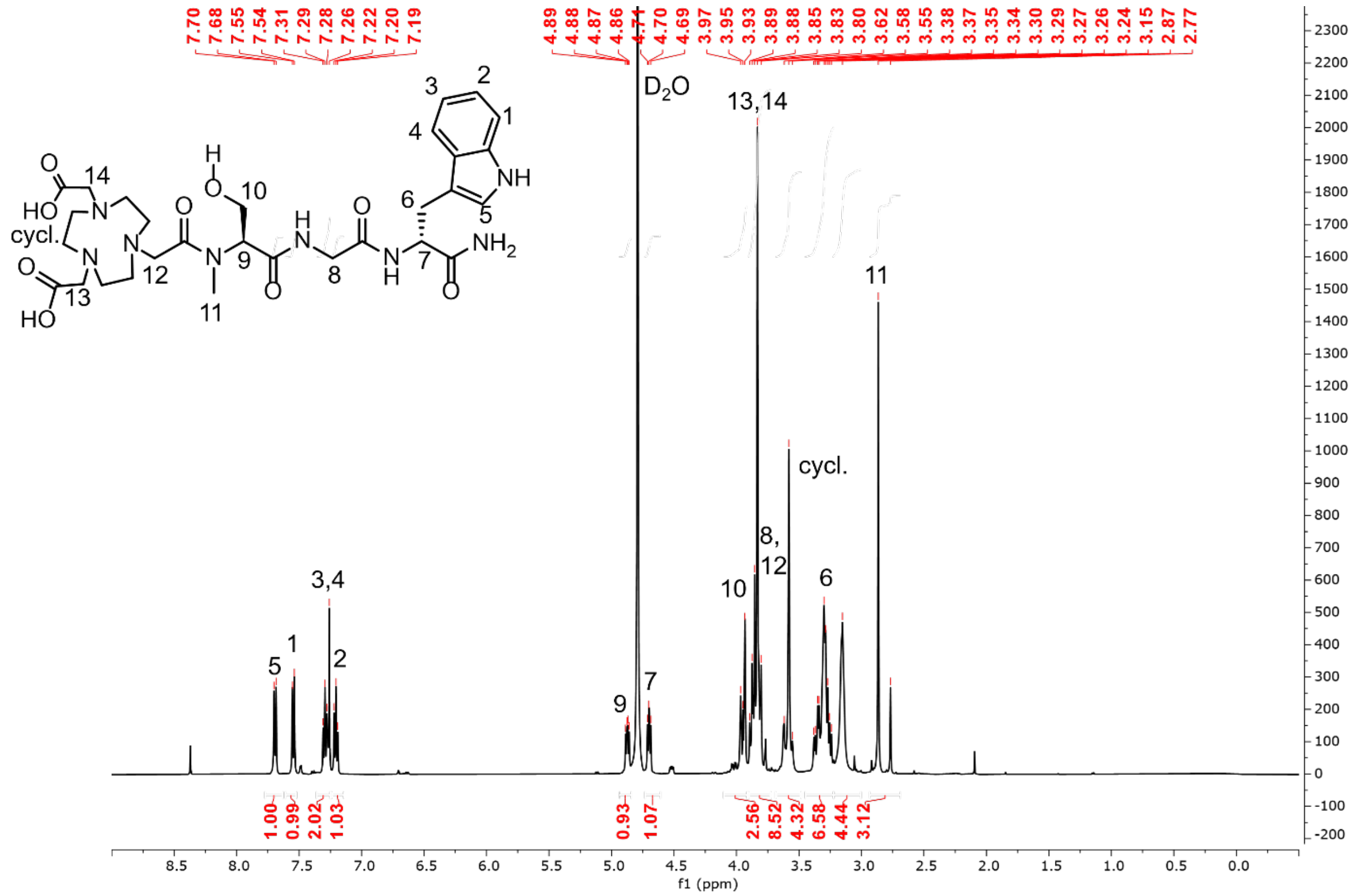

Figure S14. ^{1}H NMR (500 MHz, D_2O) for compound **3**.

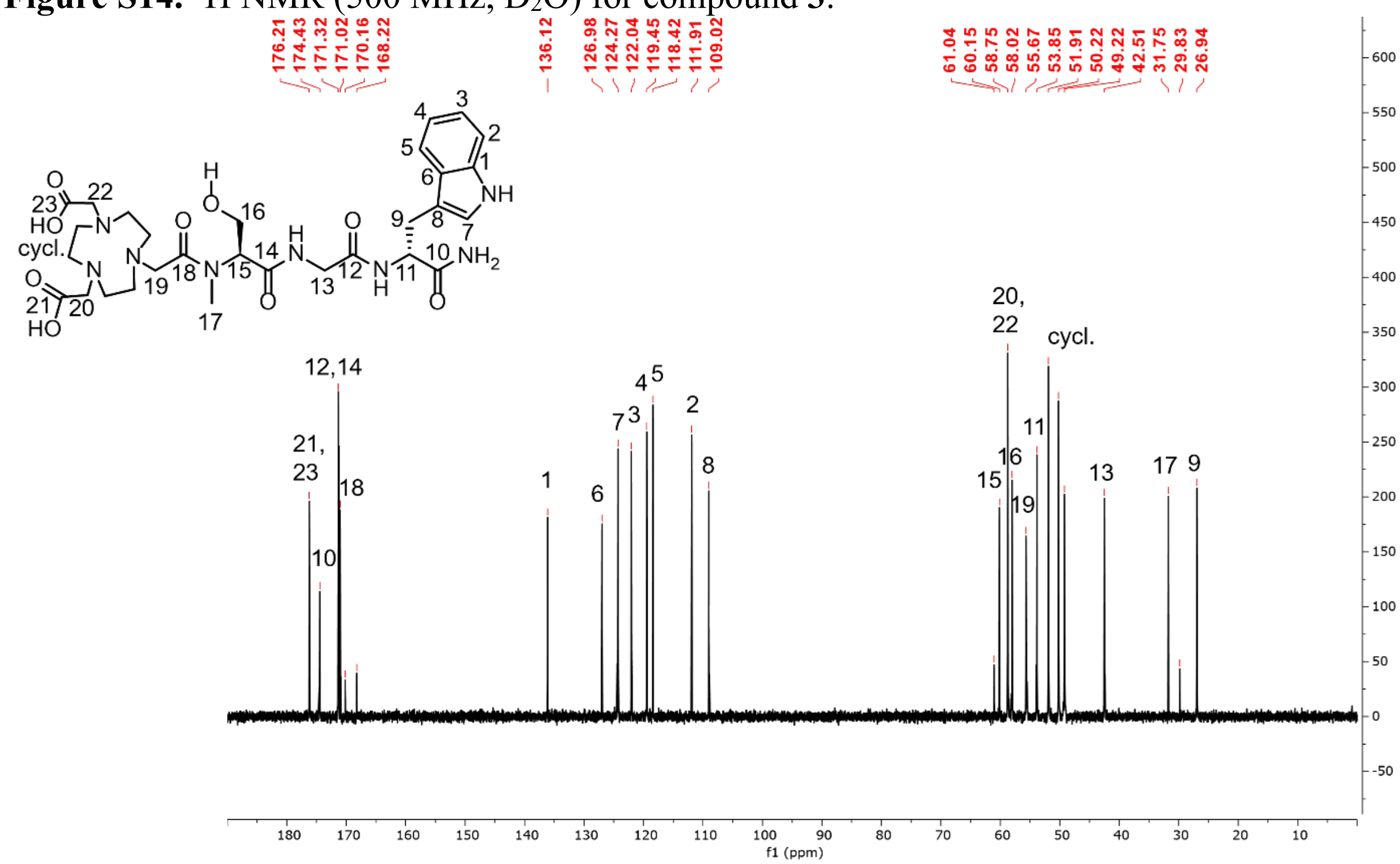

Figure S15. ^{13}C NMR (126 MHz, D_2O) for compound **3**.

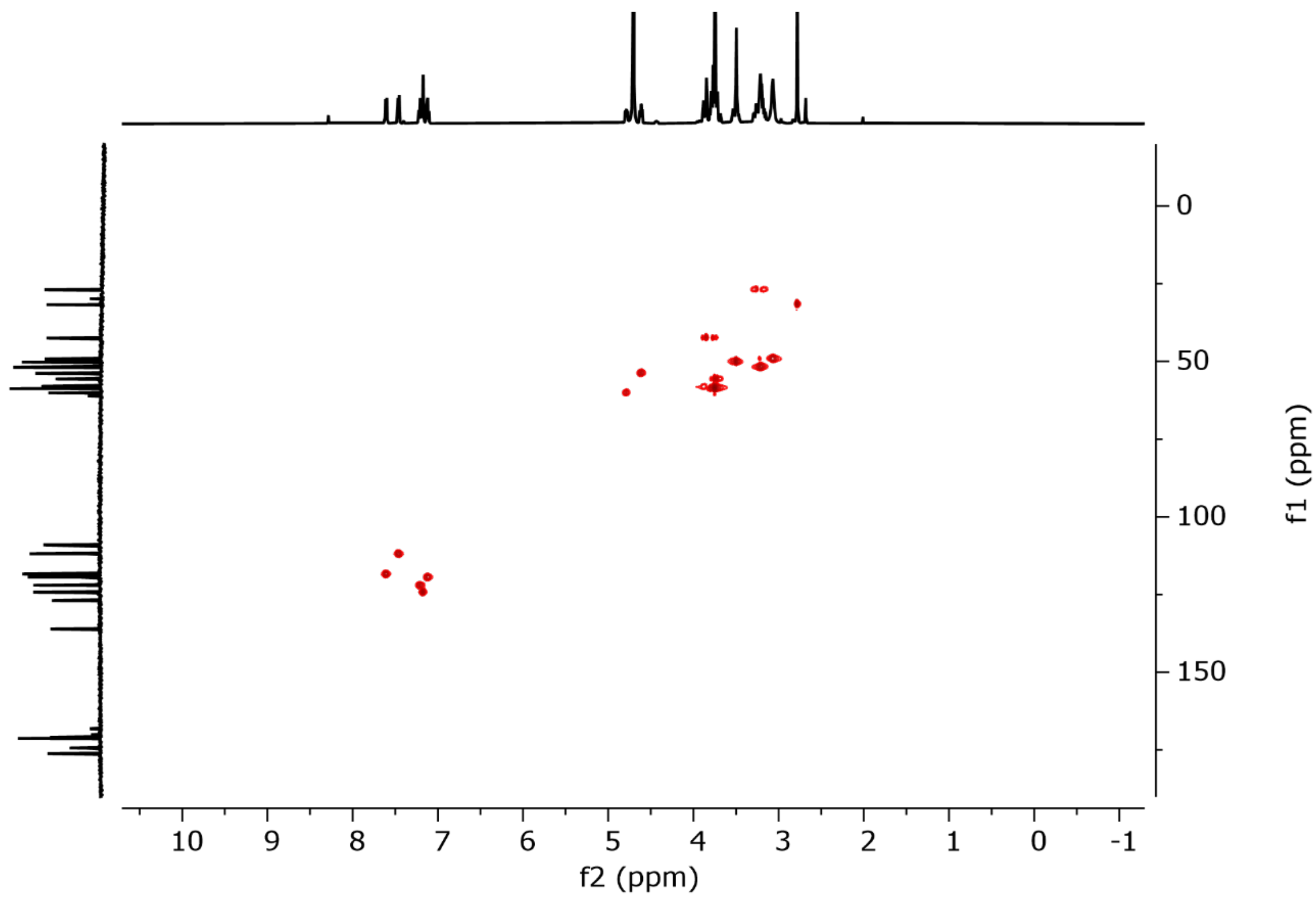

Figure S16. The ^{1}H-^{13}C HSQC NMR spectrum for compound **3**.

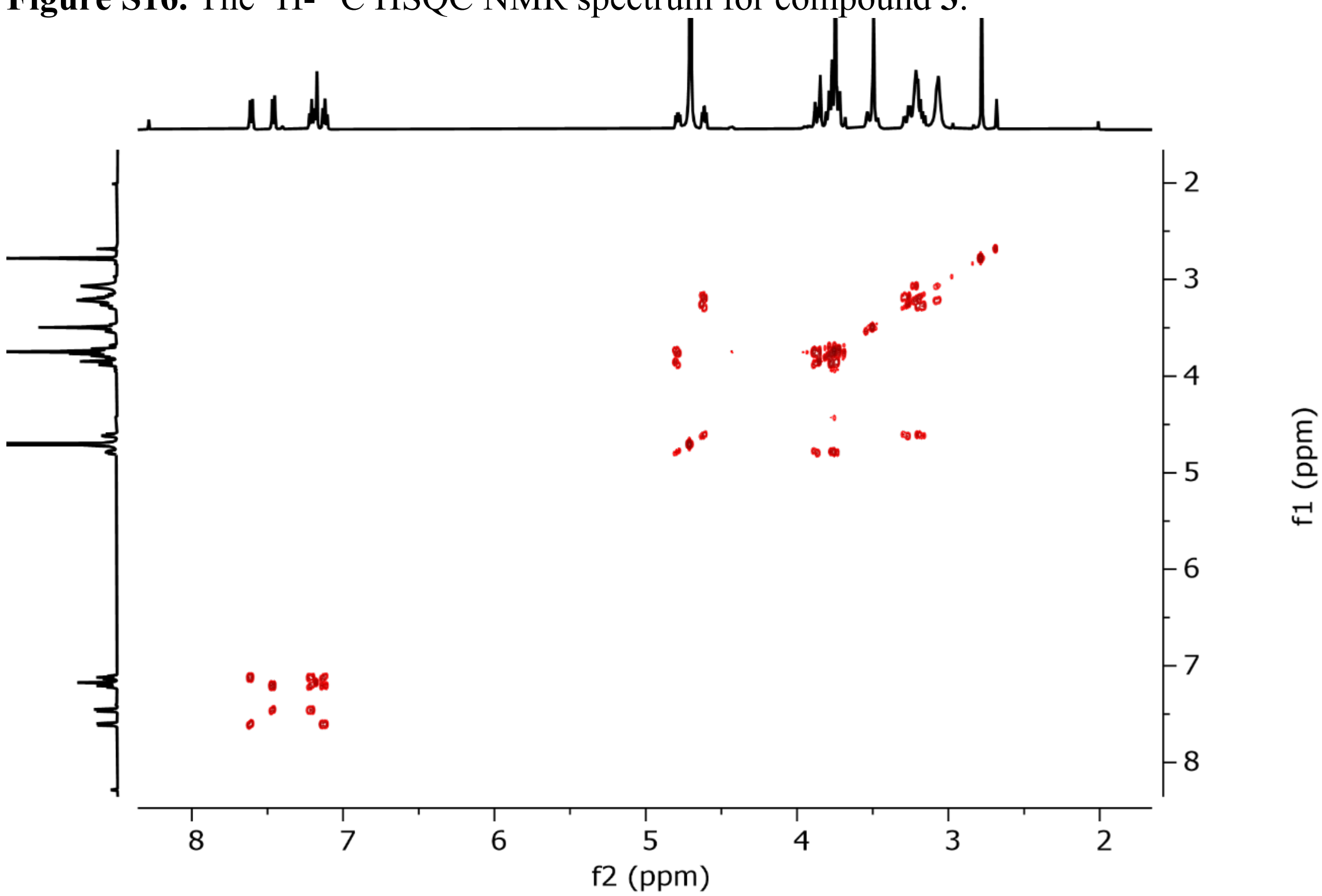

Figure S17. The ^{1}H-^{1}H COSY NMR spectrum for compound **3**.

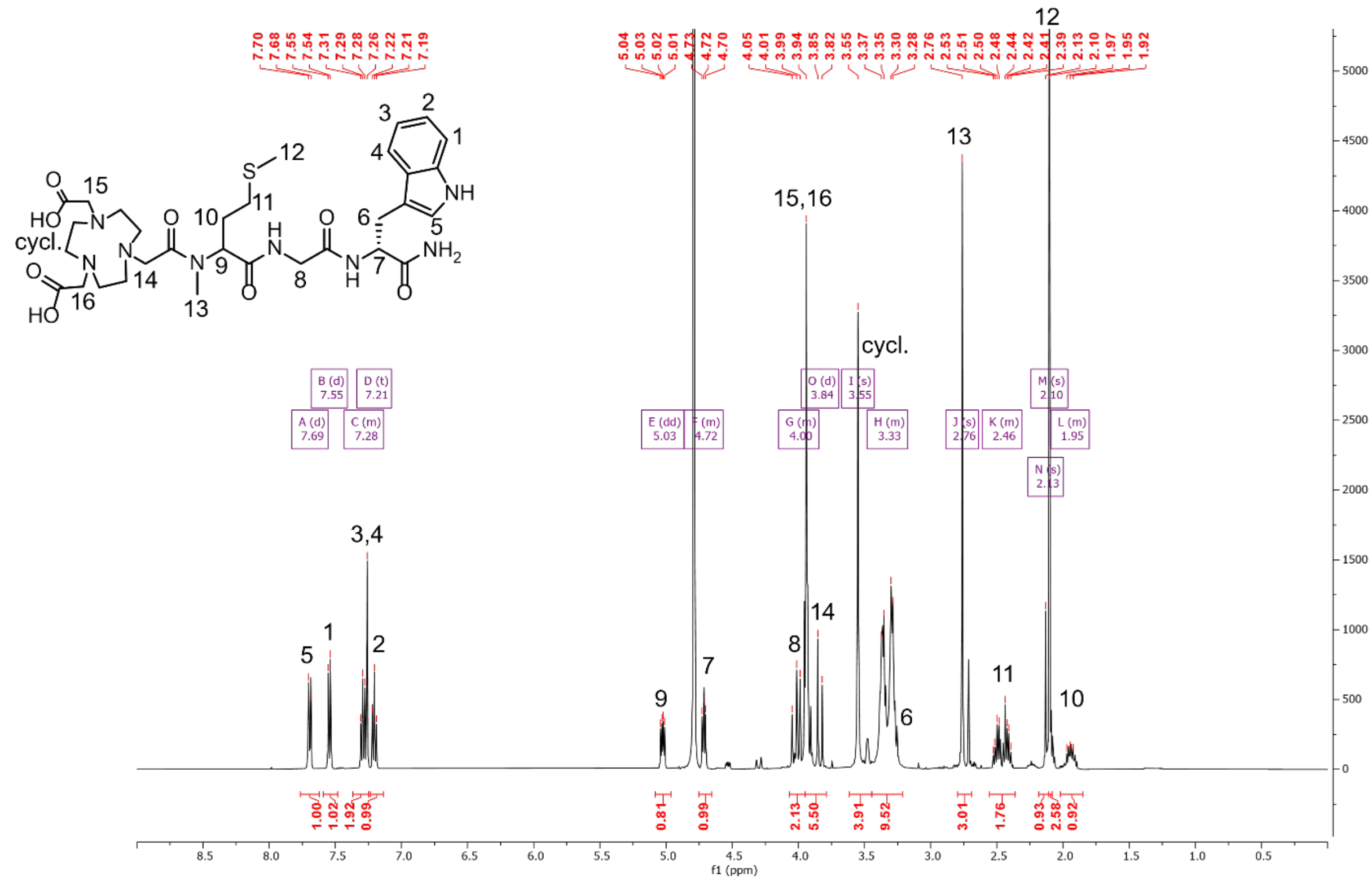

Figure S18. ^{1}H NMR (500 MHz, D_2O) for compound **4**.

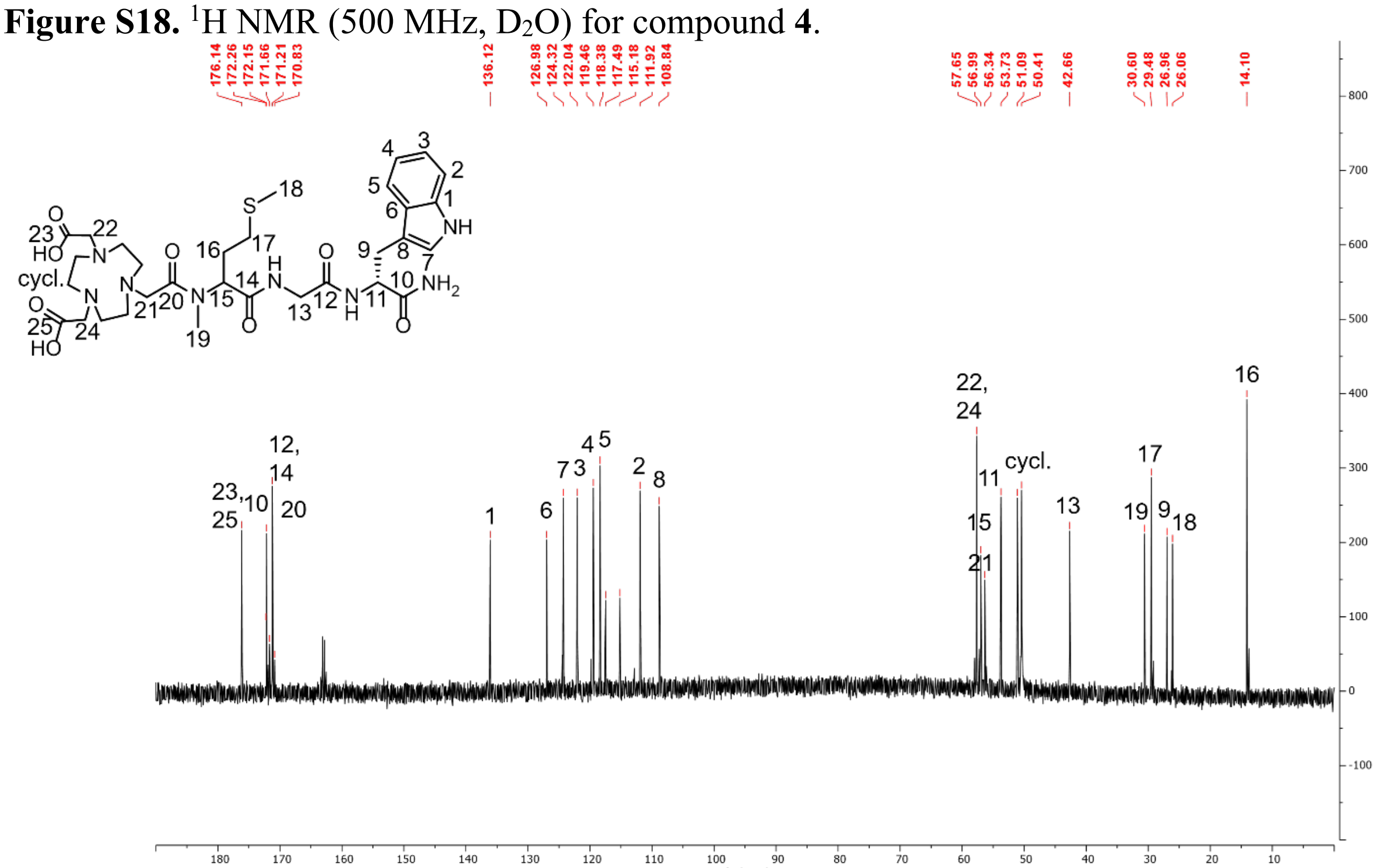

Figure S19. ^{13}C NMR (126 MHz, D_2O) for compound **4**.

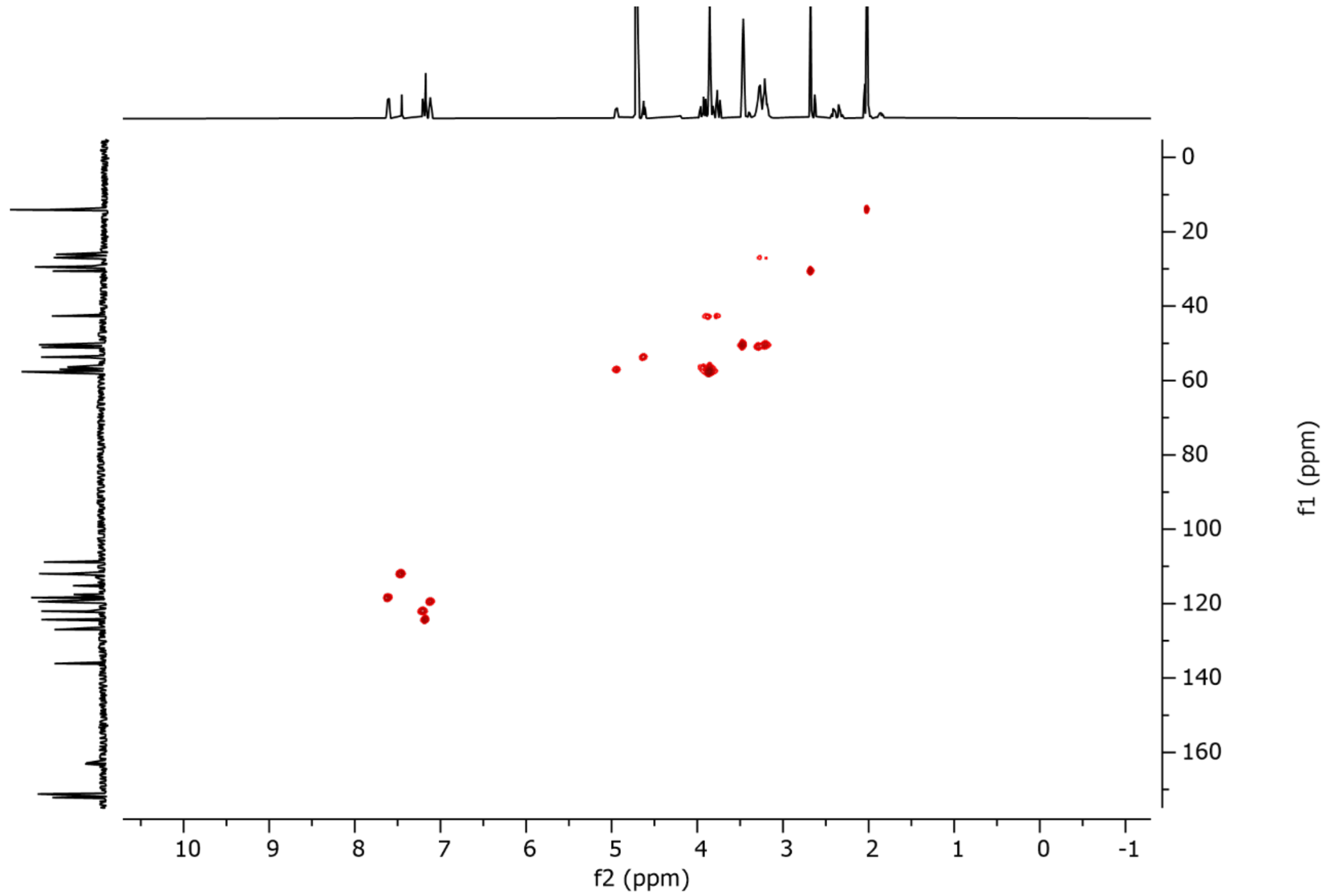

Figure S20. The ^{1}H-^{13}C HSQC NMR spectrum for compound **4**.

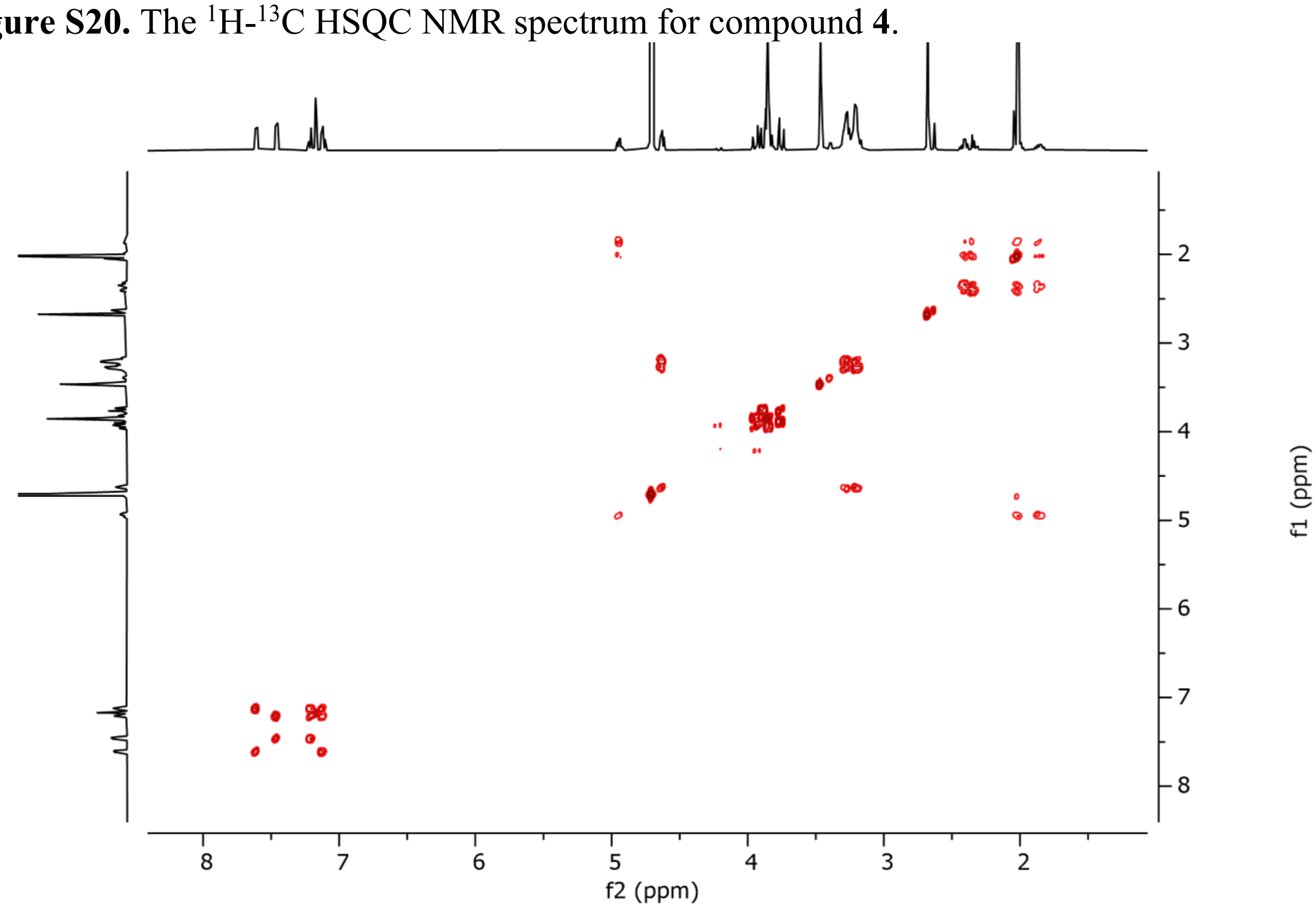

Figure S21. The ^{1}H-^{1}H COSY NMR spectrum for compound **4**.

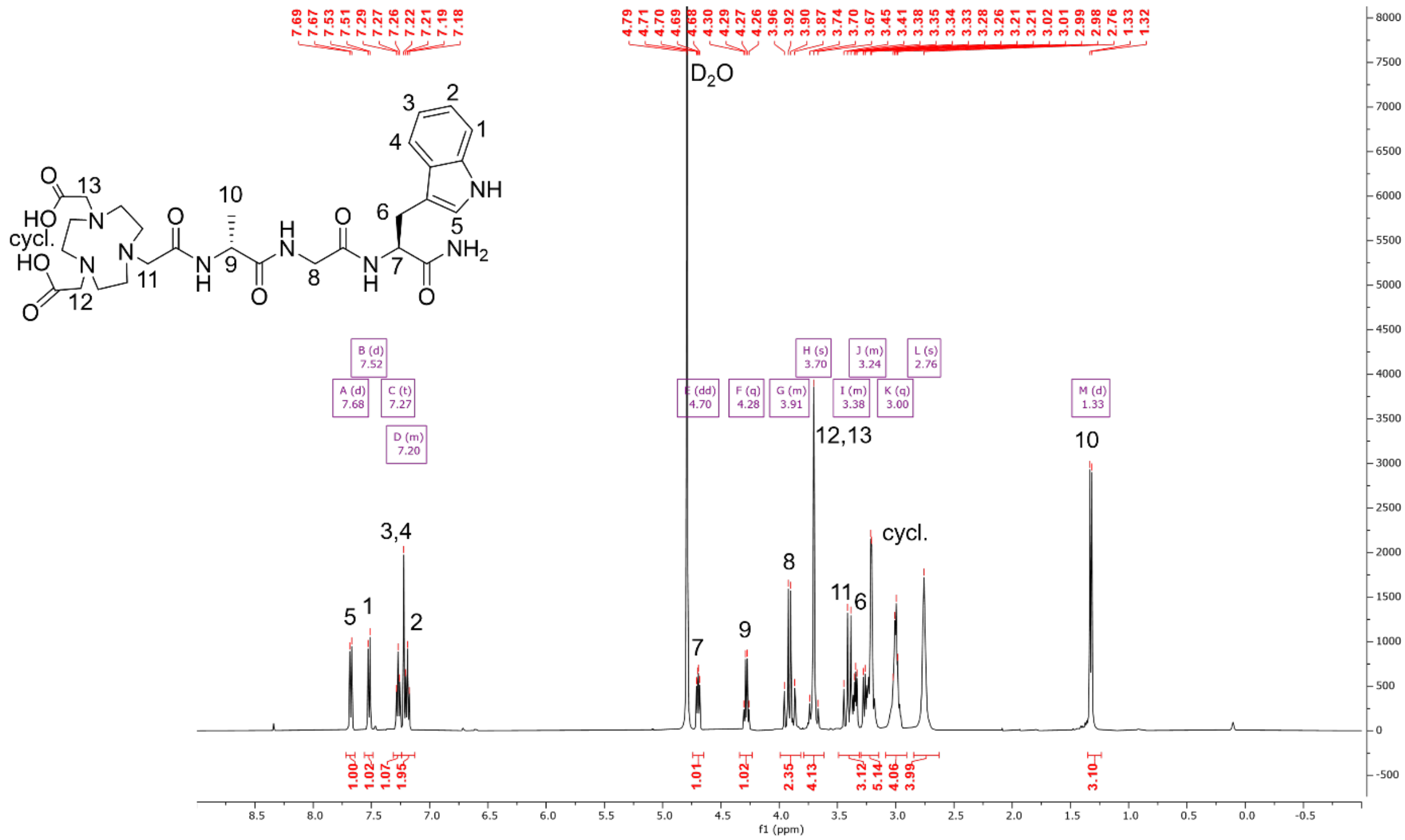

Figure S22. ^{1}H NMR (500 MHz, D_2O) for compound **5**.

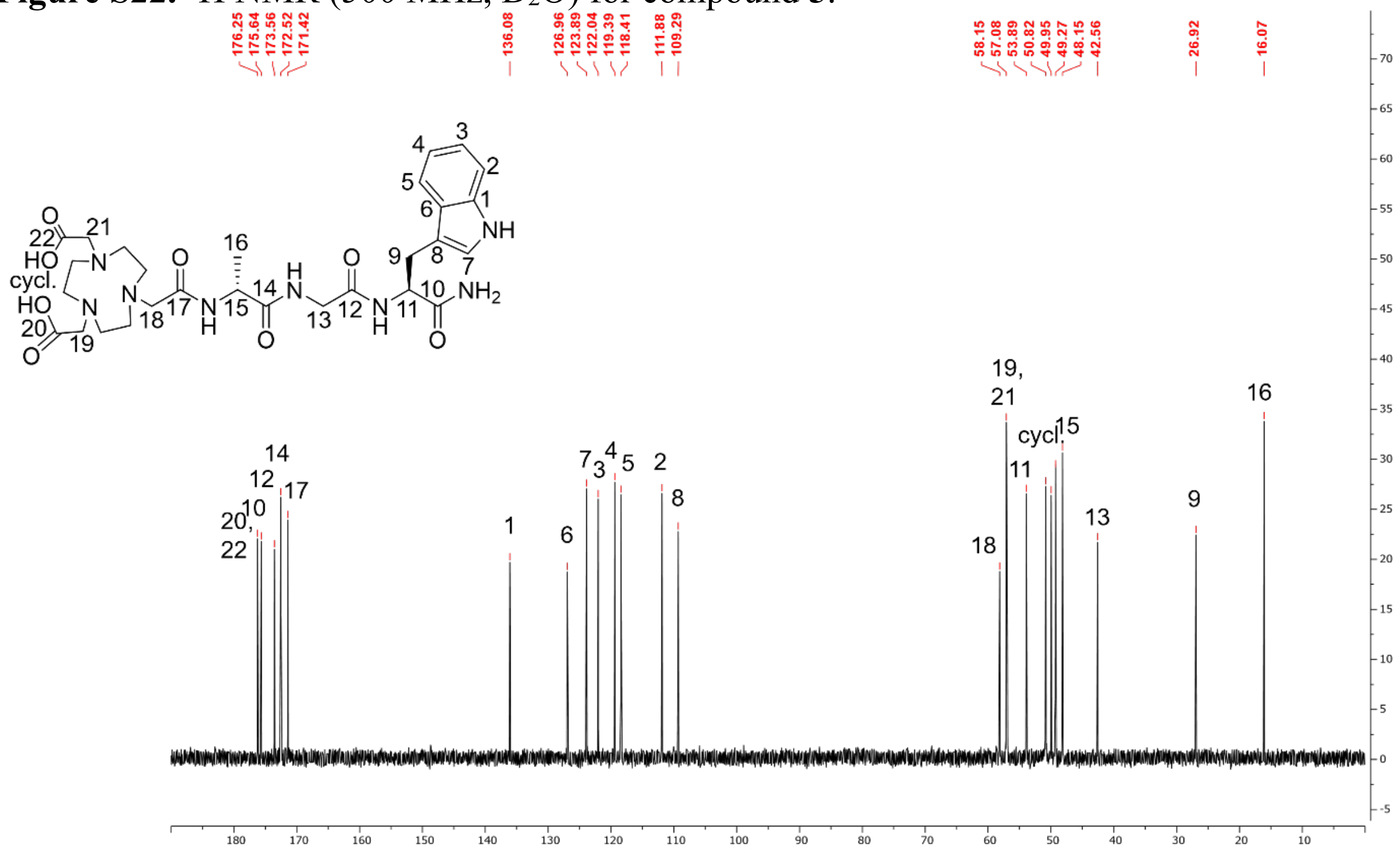

Figure S23. ^{13}C NMR (126 MHz, D_2O) for compound **5**.

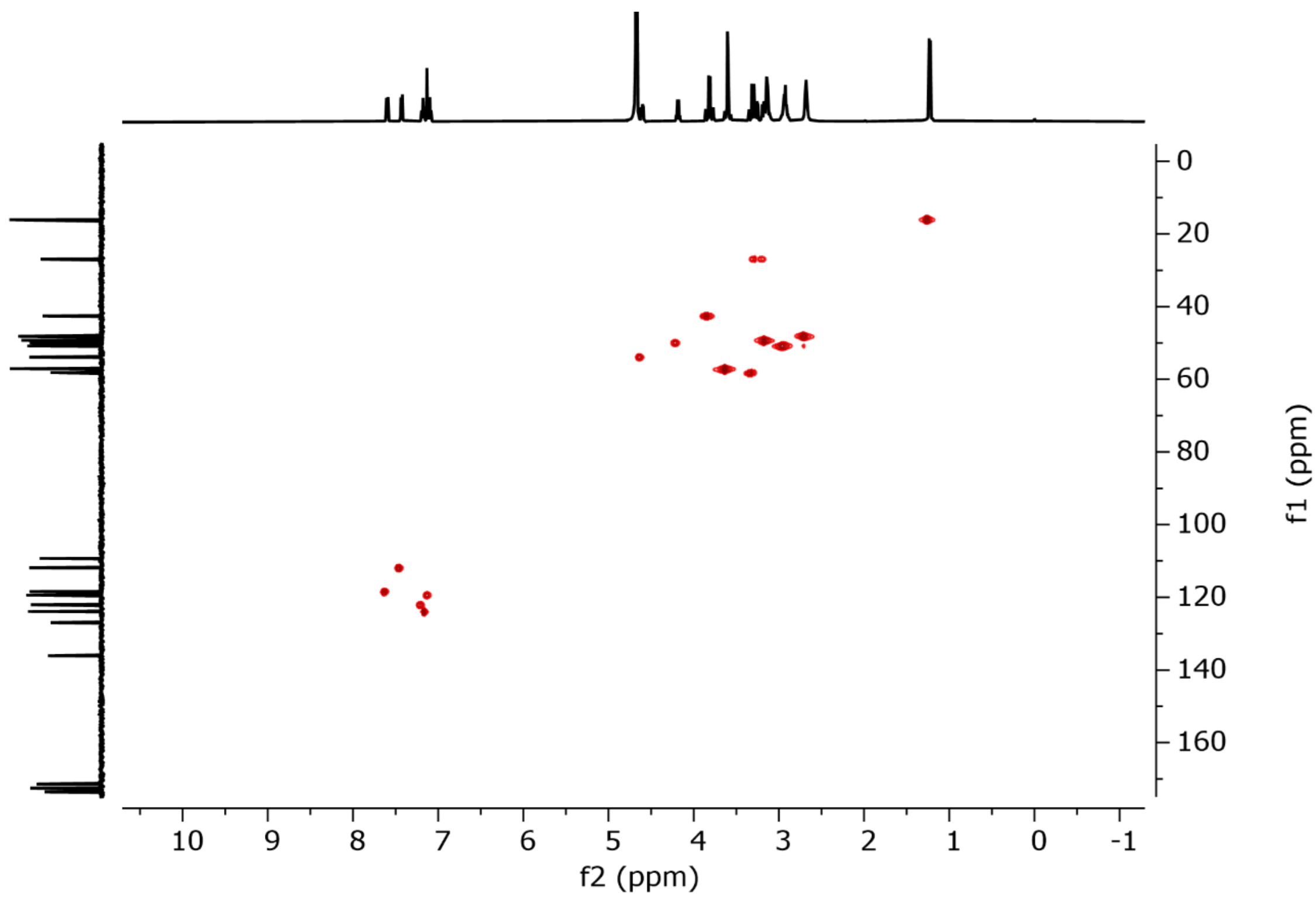

Figure S24. The ^{1}H-^{13}C HSQC NMR spectrum for compound **5**.

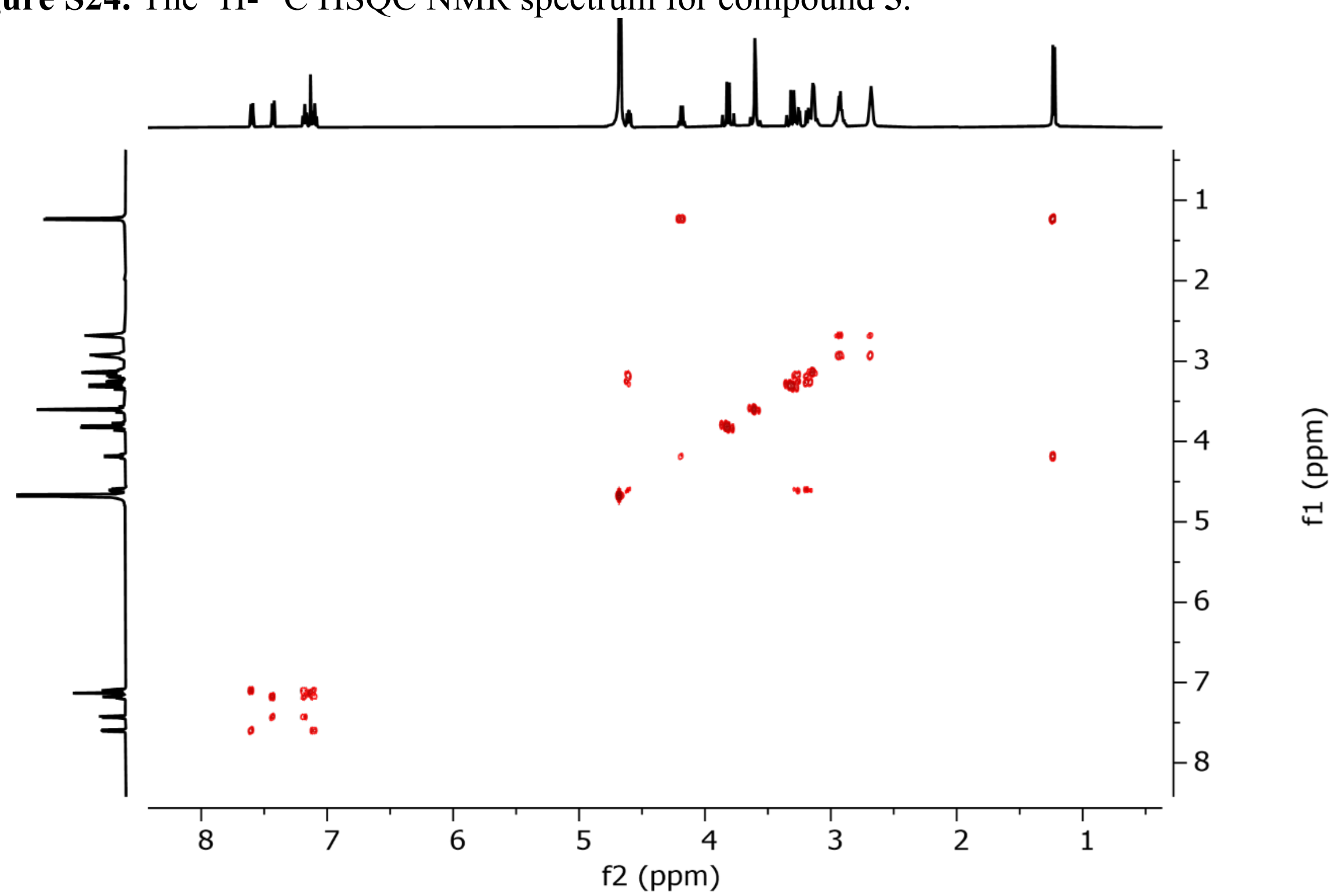

Figure S25. The ^{1}H-^{1}H COSY NMR spectrum for compound **5**.

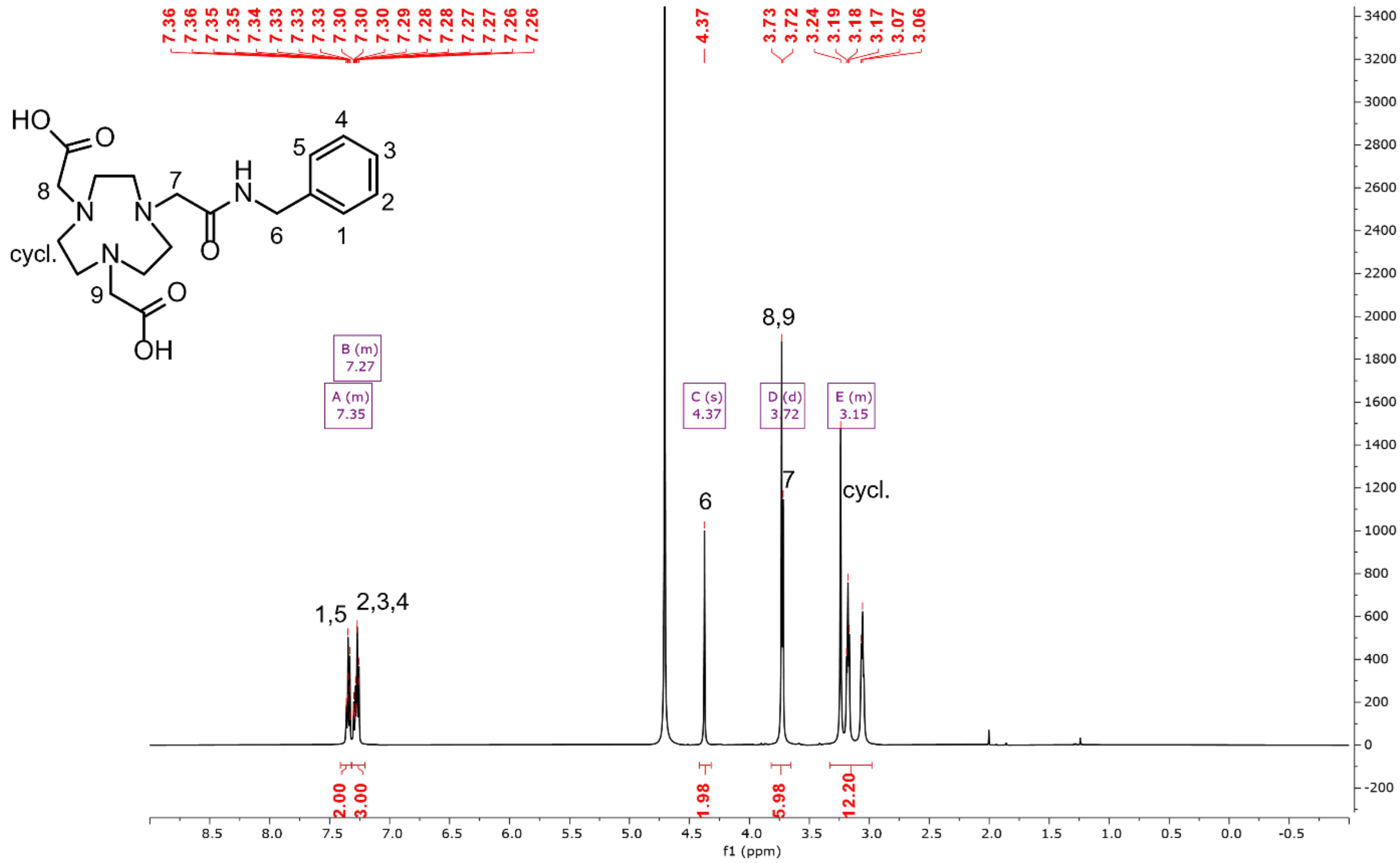

Figure S26. ^{1}H NMR (500 MHz, D_2O) for compound **6**.

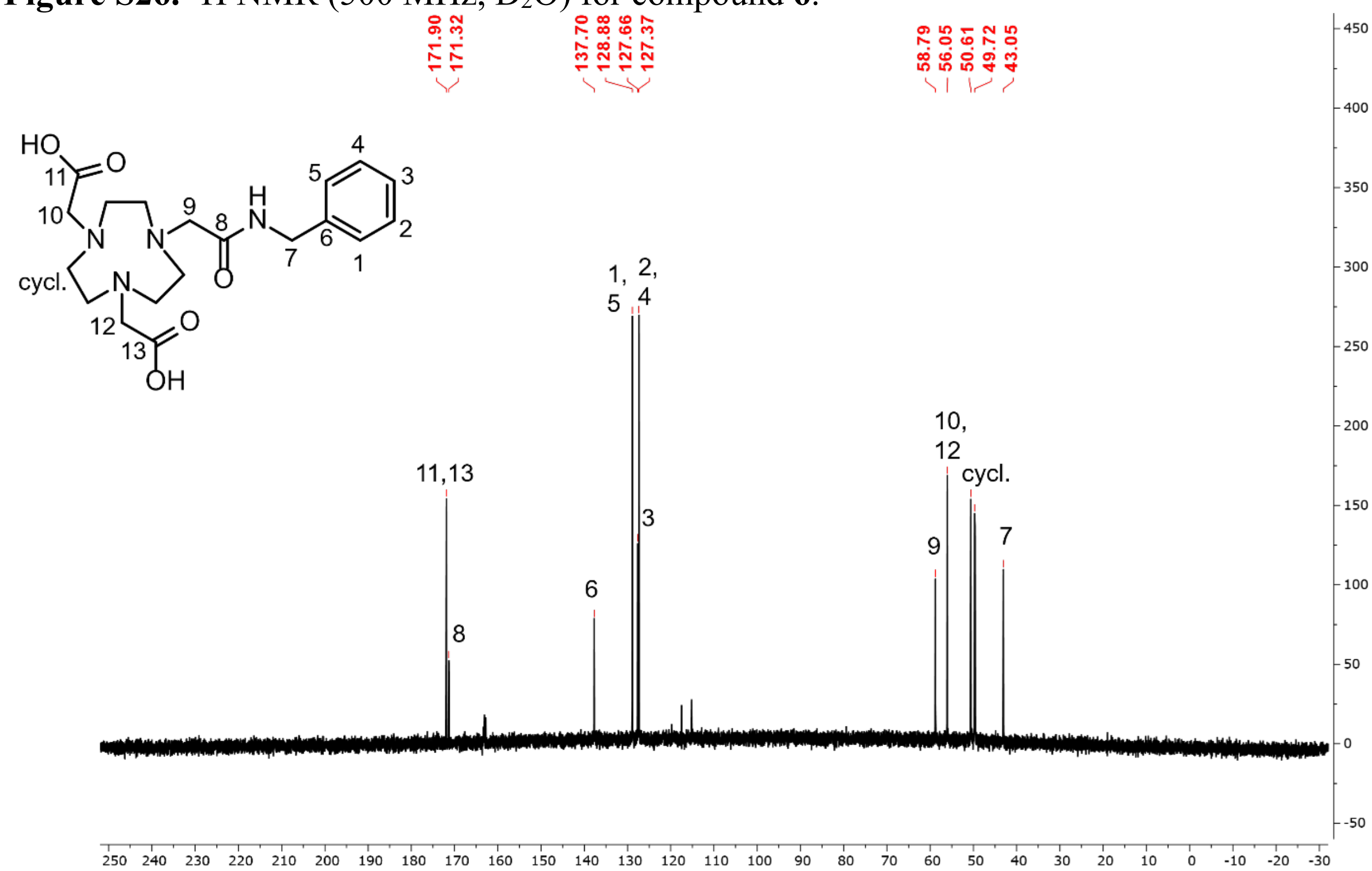

Figure S27. ^{13}C NMR (126 MHz, D_2O) for compound **6**.

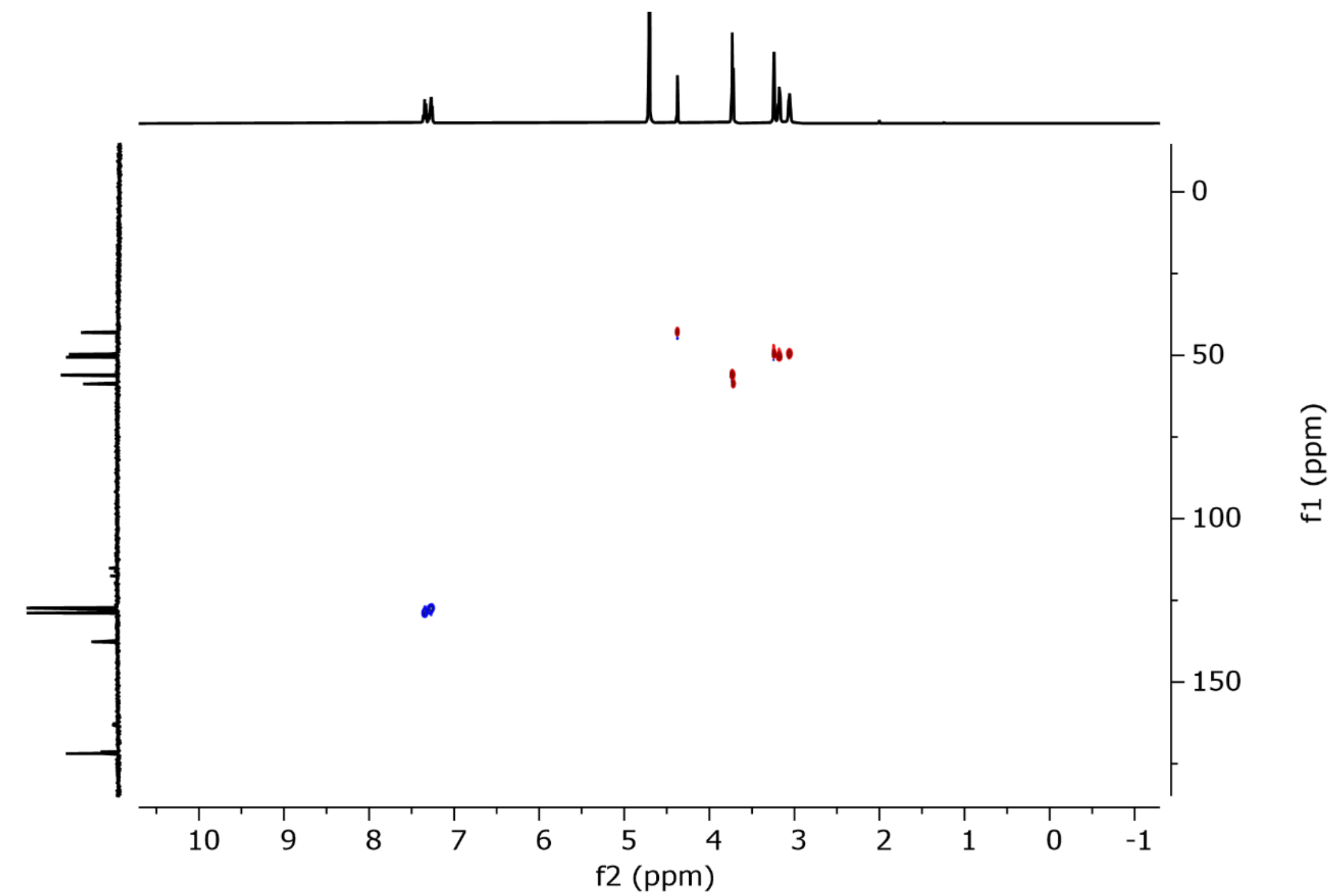

Figure S28. The ^{1}H-^{13}C HSQC NMR spectrum for compound **6**. Edited HSQC method: CH + CH_3 peak shows positive, and CH_2 peak shows negative.

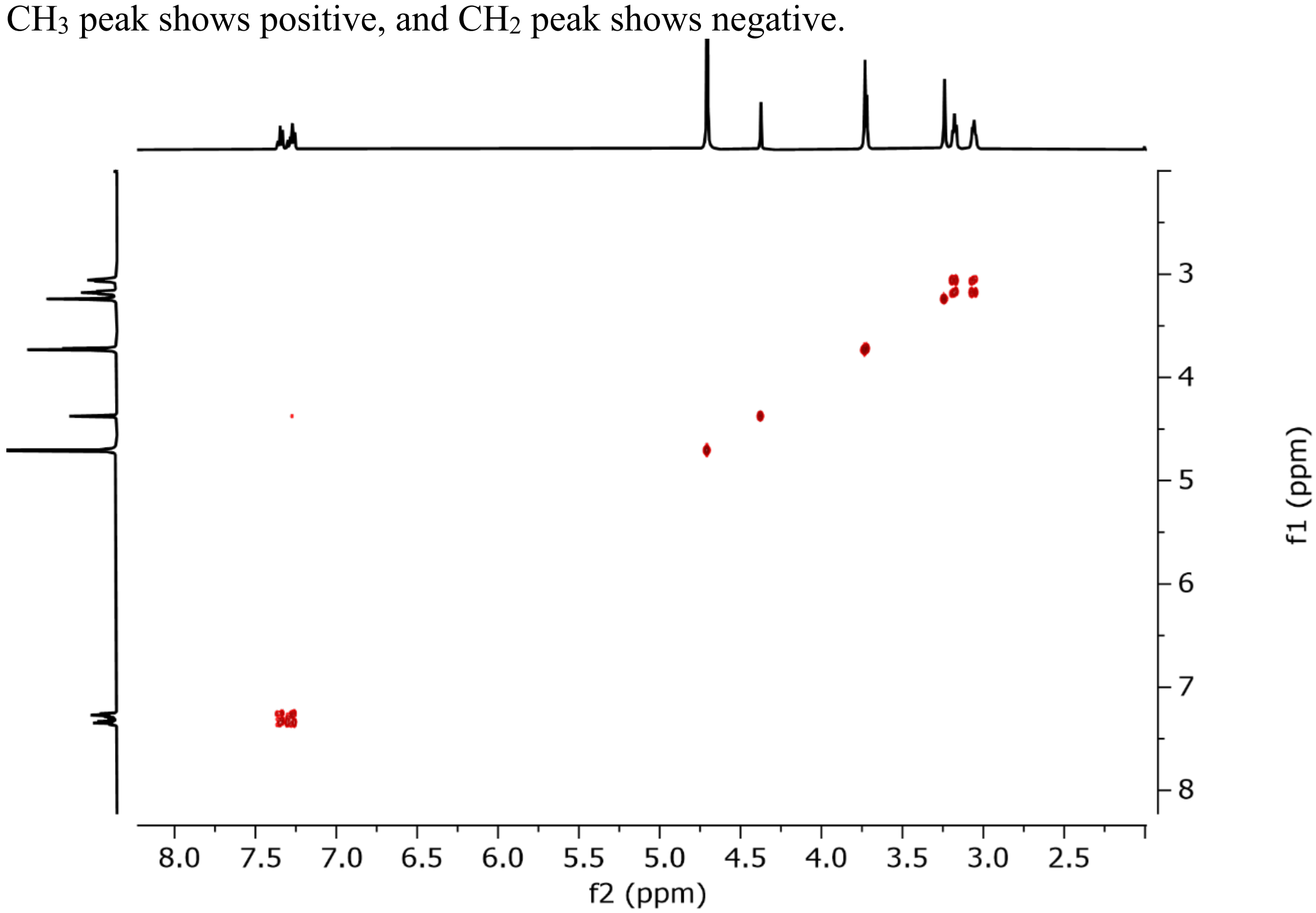

Figure S29. The ^{1}H-^{1}H COSY NMR spectrum for compound **6**.

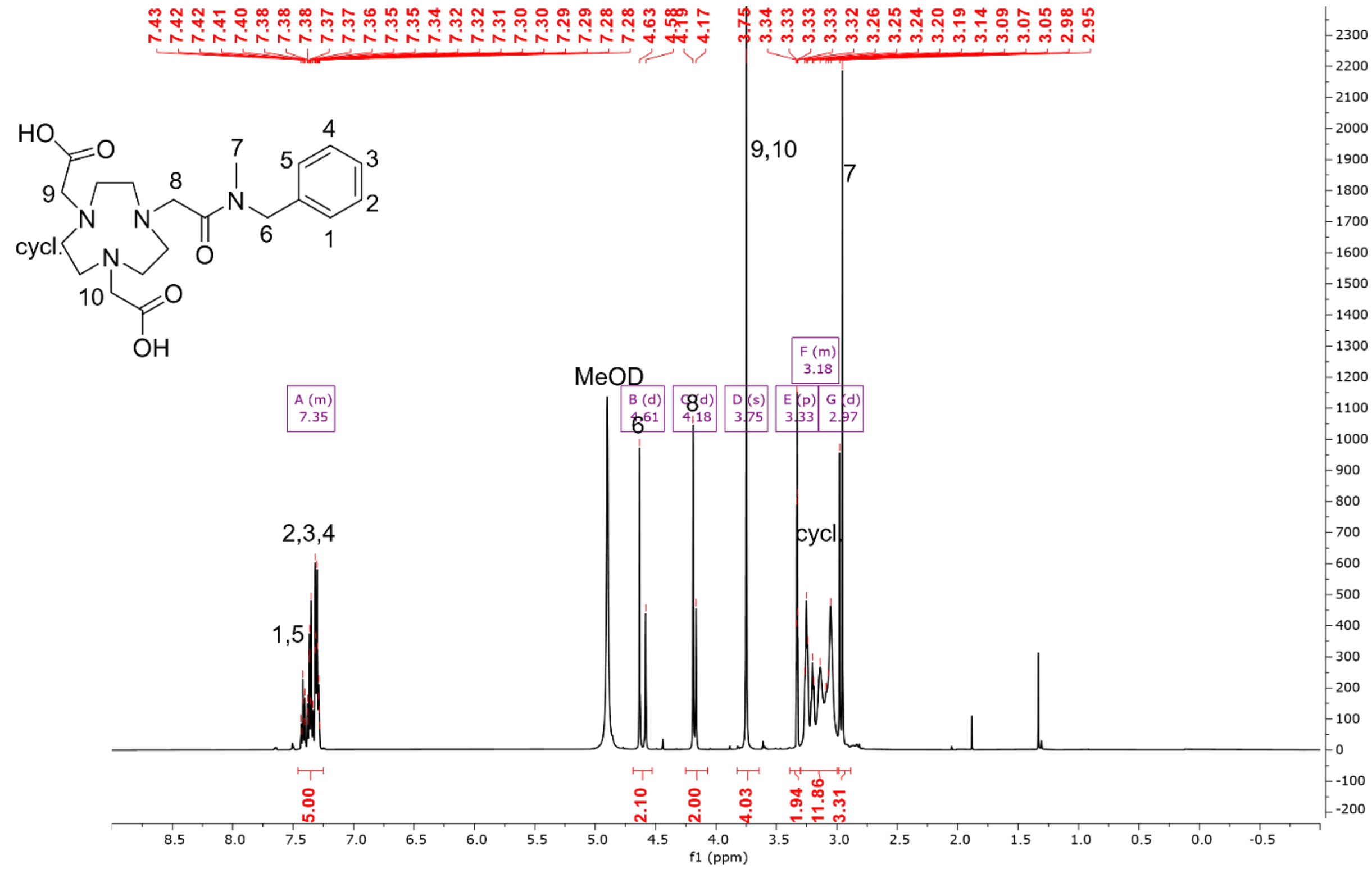

Figure S30. ^{1}H NMR (500 MHz, MeOD-d4) for compound **7**.

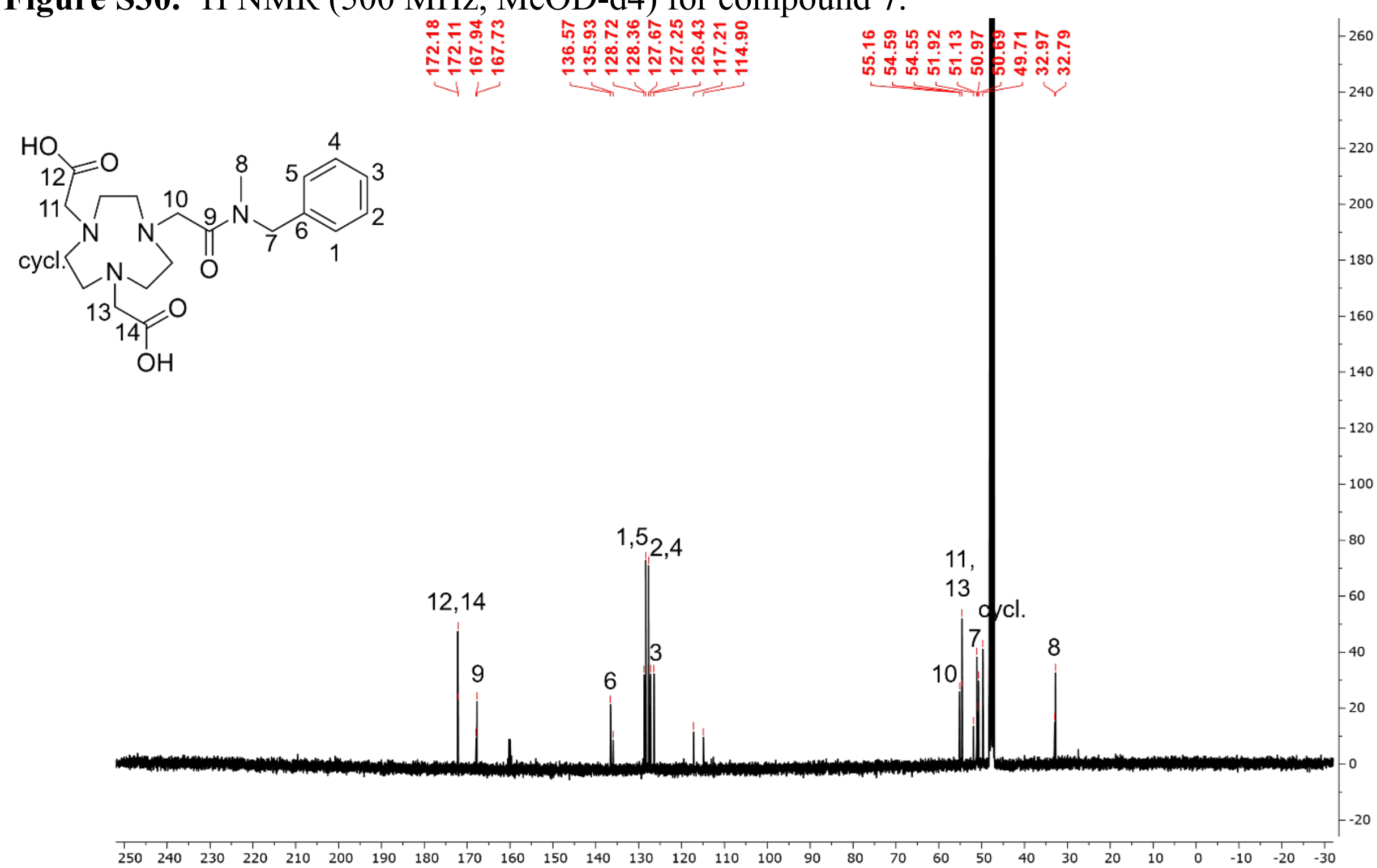

Figure S31. ^{13}C NMR (126 MHz, MeOD-d4) for compound **7**.

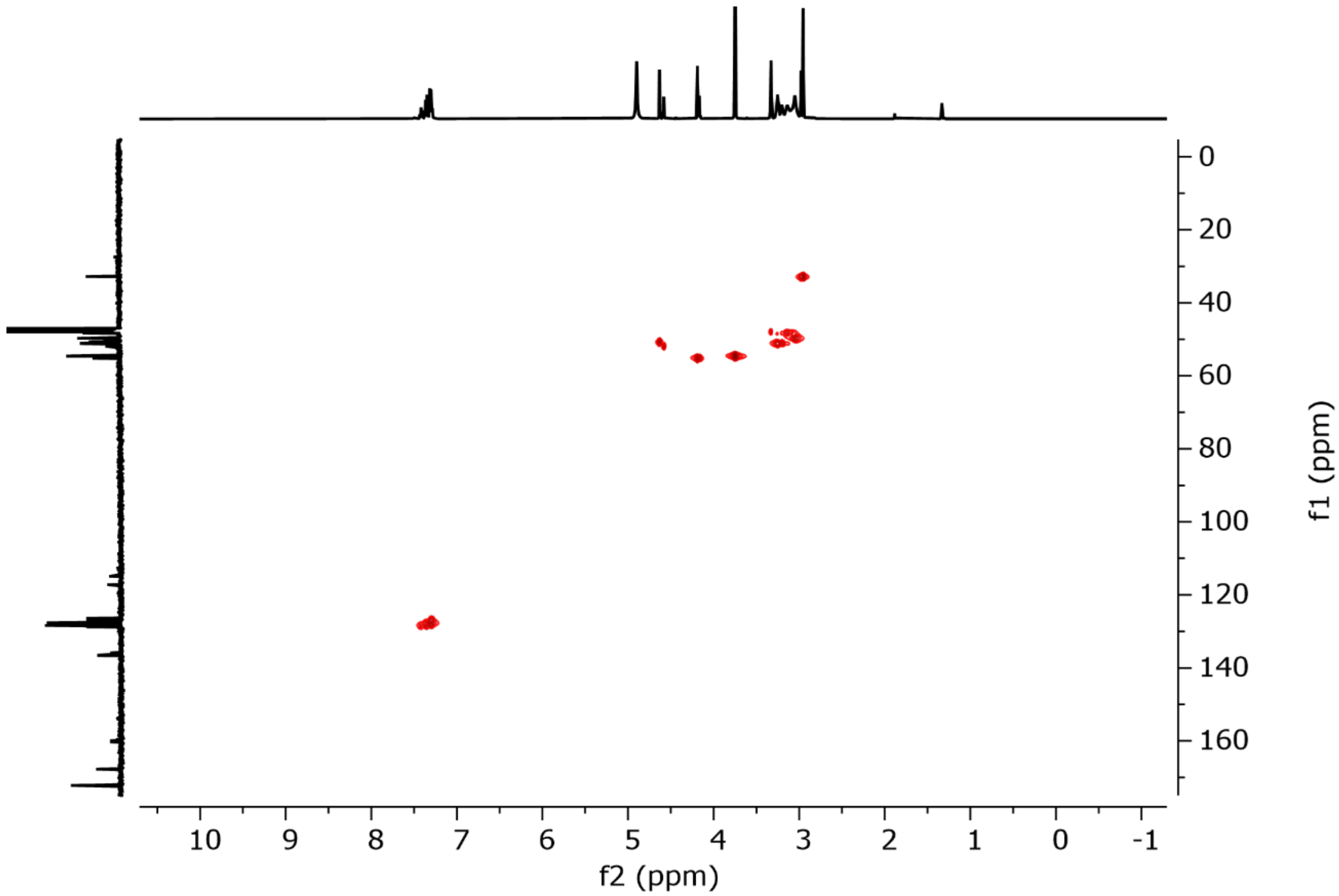

Figure S32. The ^{1}H-^{13}C HSQC NMR spectrum for compound **7**.

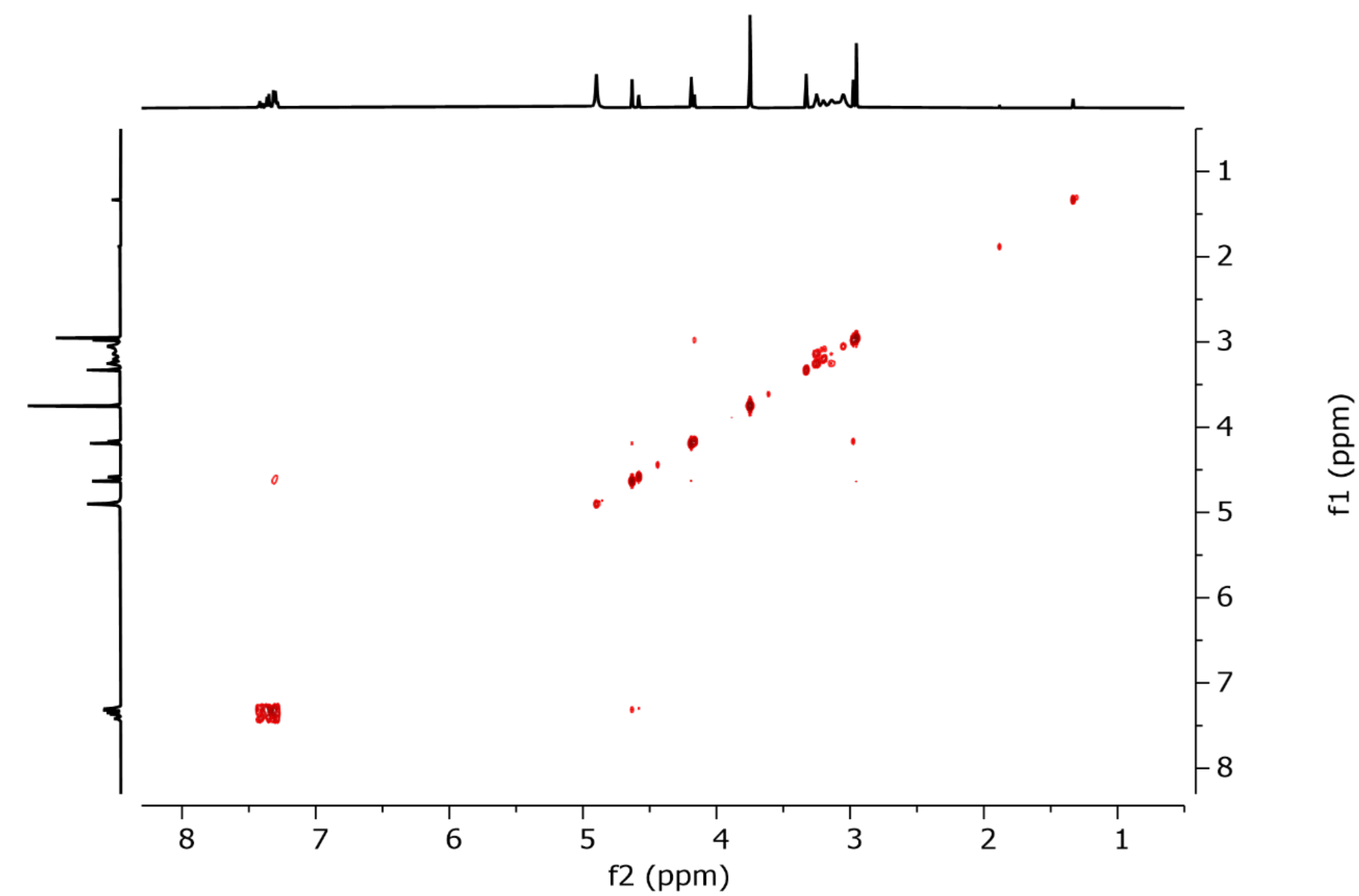

Figure S33. The ^{1}H-^{1}H COSY NMR spectrum for compound **7**.

4 Complex synthesis and characterization

4.1 General Complexation and Cleavage Assay Protocol

A 1 mg aliquot of the model tripeptide, **1 – 5**, was dissolved in 200 µL of room-temperature distilled water. The concentrations of model tripeptides were determined based on extinction coefficient of tryptophan (5600 M^{-1} cm^{-1}at 280 nm at pH 7.4) using UV-Vis spectroscopy. The concentrations of metal stock solutions (Zn^{2+}, Ga^{3+}, Fe^{3+}, In^{3+}, and Co^{2+}) were determined by ICP-OES. The ligand and metal stock solution were mixed in 1:1 ratio, and the pH was adjusted to pH 4.5, and stirred further for 15 minutes. Full complexation was confirmed by LC-MS. The complex was characterized by HRESI-MS.

Cleavage of the cold model complexes (0.75 mM) was monitored at 80 °C and pH values of 4.5 – 7.4. Aqueous buffer solutions were prepared using 10 mM NaOAc (pH 4.5), 10 mM MOPS (pH 6.5), and 10 mM HEPES (pH 7.4) which were subsequently adjusted to the desired pH using NaOH and/or HCl. An aliquot of the complex was placed in an aqueous solution of the desired pH and incubated for 24 hours at 80 ºC. The reaction was monitored via analytical HPLC or VT NMR at relevant time periods.

Cleavage of the cold model complexes $[Ga(3)]^+$ and $[In(3)]^+$ (1 mM) was monitored at 80 ºC and pH 7.4 by VT ^1H NMR spectroscopy because of their rapid cleavage kinetics. Water-suppressed 1H NMR spectra were acquired every 4-5 minutes to monitor the formation of the cleavage product.

4.2 HRMS and HPLC Data for Coordination Complexes

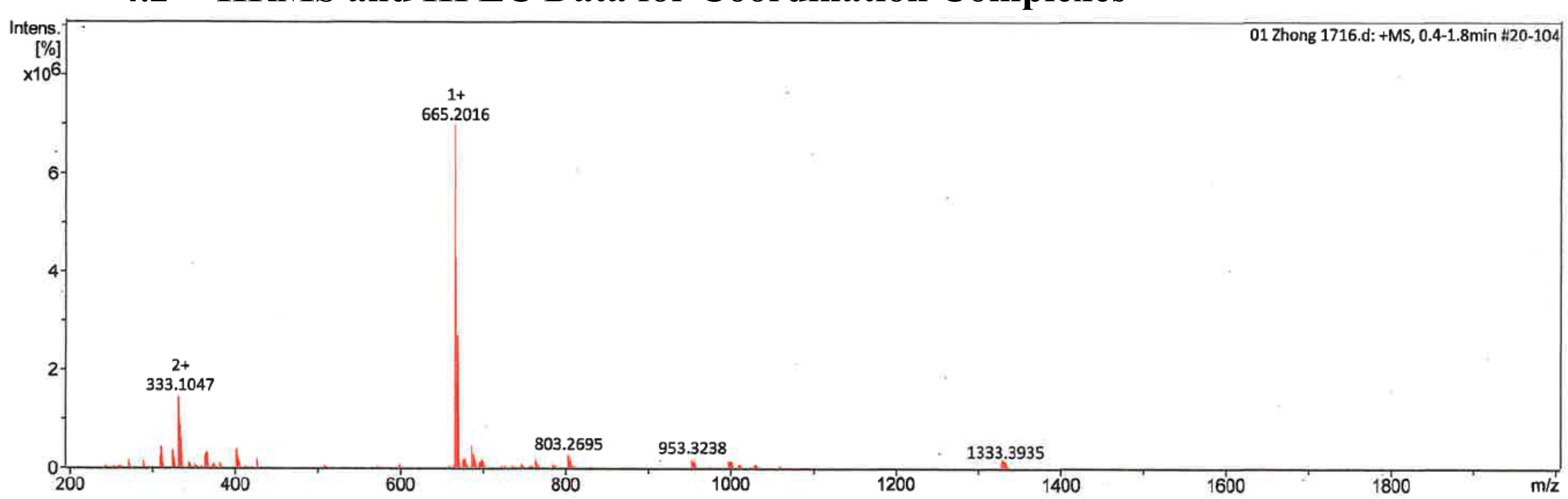

Figure S34. HRESI-MS of [Zn(NO2A)]-G-G-W, Zn(1). HRMS calc. for $C_{27}H_{36}N_8O_8Zn$ 665.2020. Found 665.2016 $[M+H]^+$.

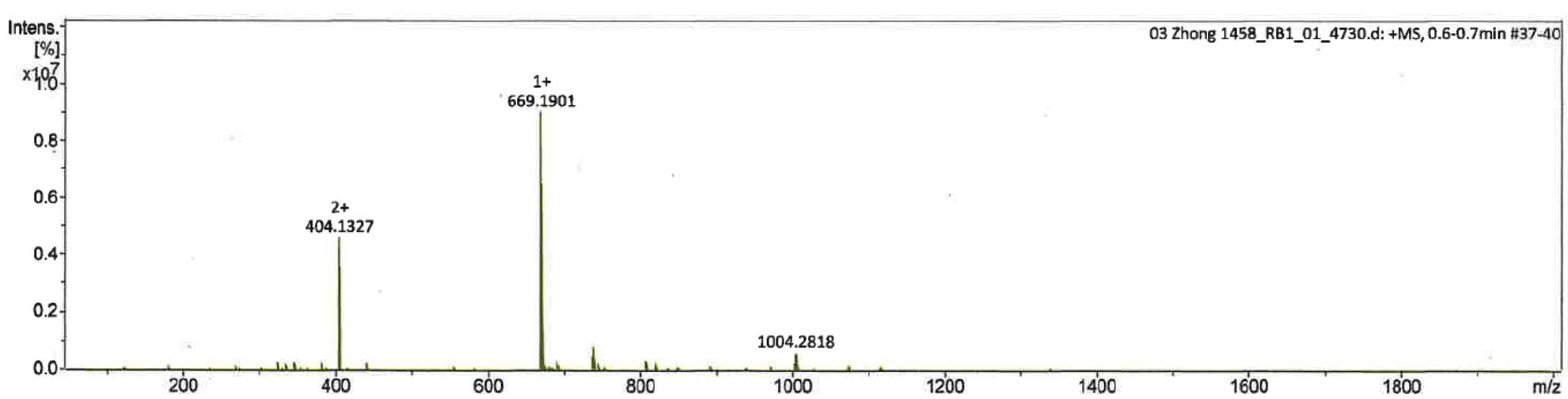

Figure S35. HRESI-MS of $[Ga(NO2A)]^+$-G-G-W, $[Ga(1)]^+$. HRMS calc. for $C_{27}H_{36}GaN_8O_8$ 669.1906. Found 669.1901 $[M]^+$.

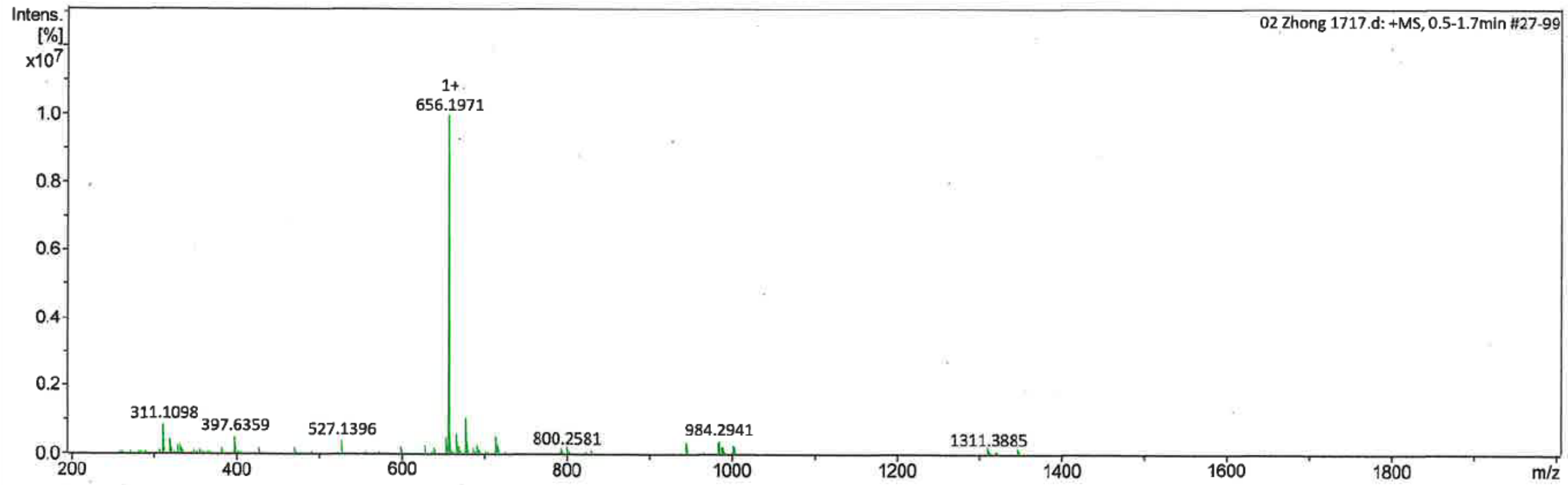

Figure S36. HRESI-MS of $[Fe(NO2A)]^{+}$-G-G-W, $[Fe(1)]^{+}$. HRMS calc. for $C_{27}H_{36}FeN_8O_8$ 656.2000. Found 656.1971 $[M]^{+}$.

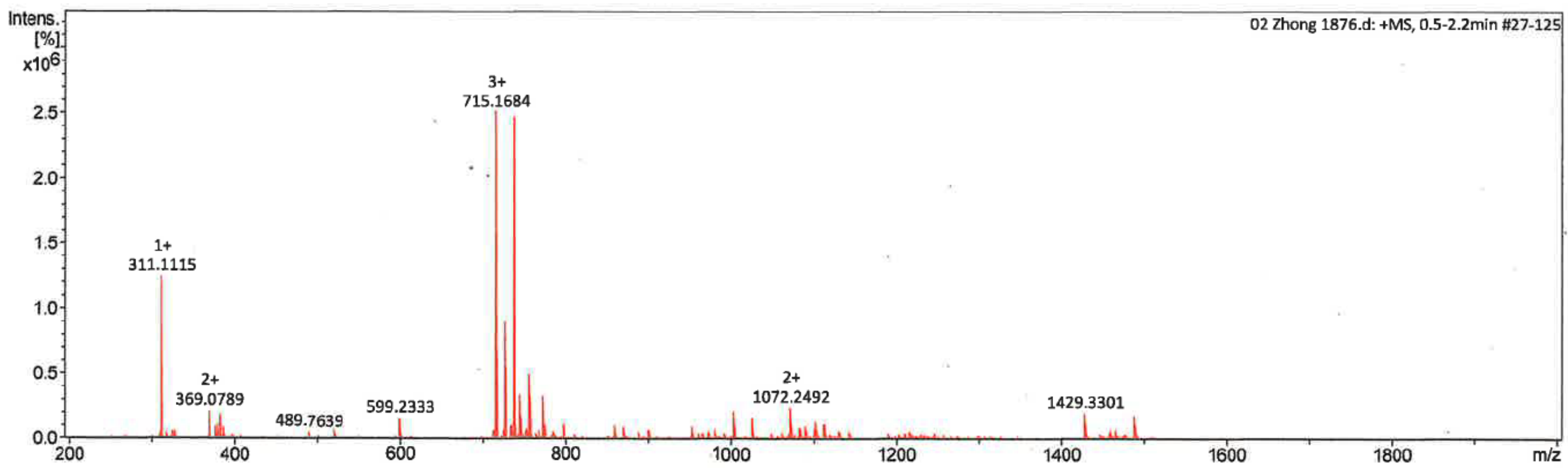

Figure S37. HRESI-MS of $[In(NO2A)]^{+}$-G-G-W, $[In(1)]^{+}$. HRMS calc. for $C_{27}H_{36}InN_8O_8$ 715.1690. Found 715.1684 $[M]^{+}$.

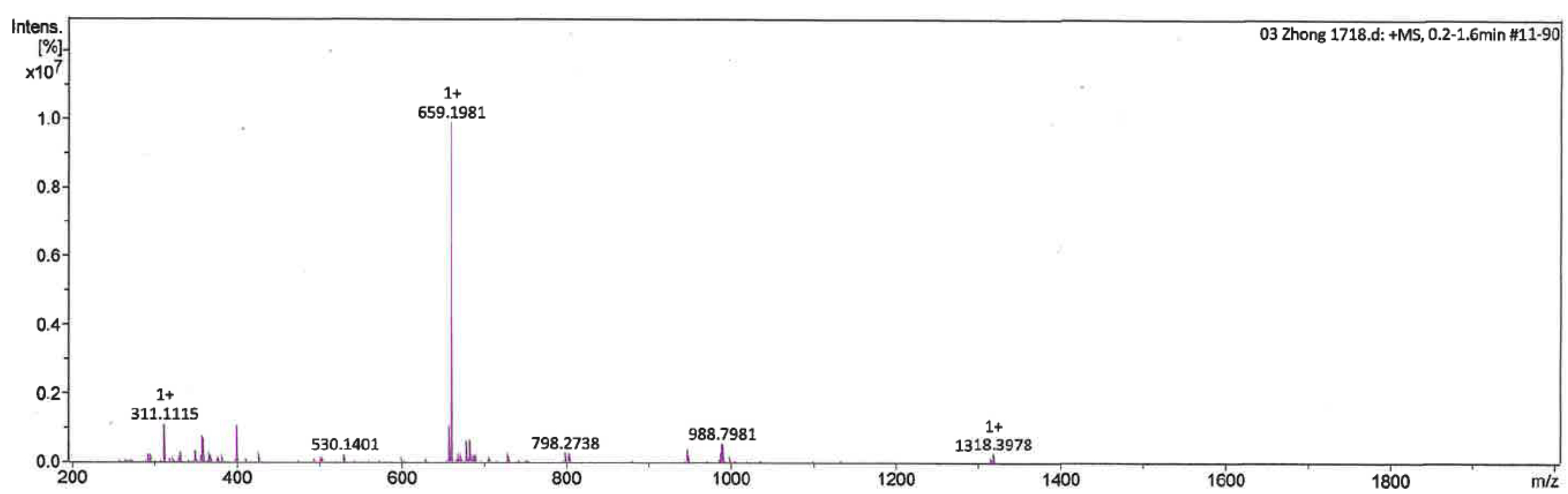

Figure S38. HRESI-MS of $[Co(NO2A)]^{+}$-G-G-W, $[Co(1)]^{+}$. HRMS calc. for $C_{27}H_{36}CoN_8O_8$ 659.1983. Found 7659.1981 $[M]^{+}$.

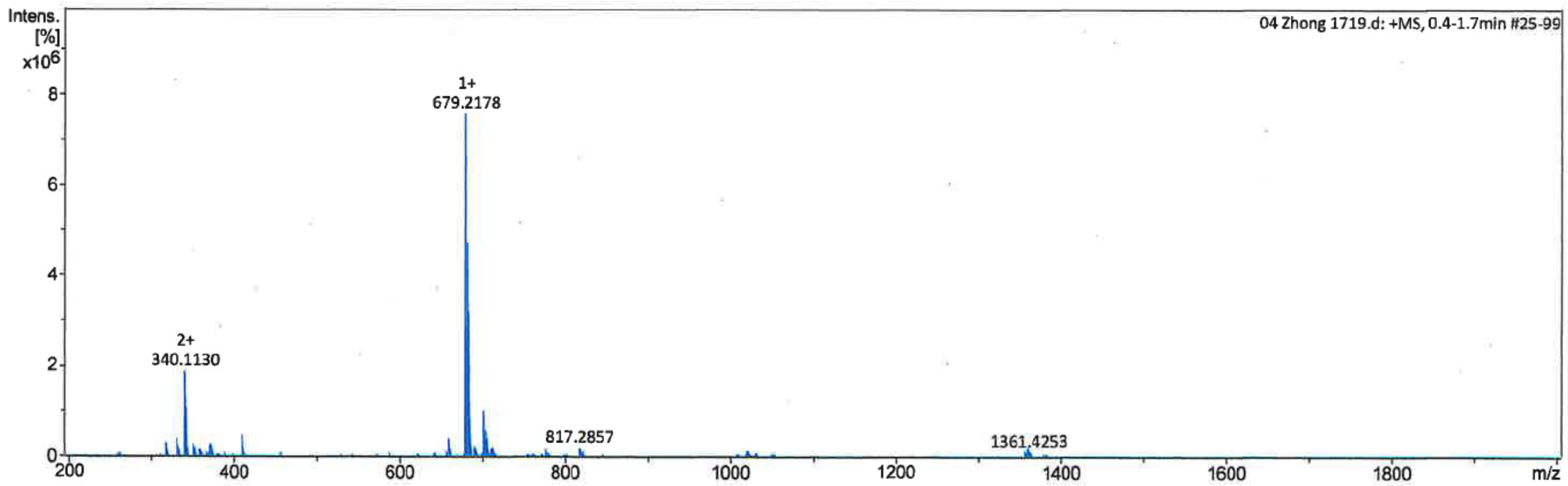

Figure S39. HRESI-MS of [Zn(NO2A)]-^{NMe}G-G-W, Zn(2). HRMS calc. for C28H38N8O8Zn 679.2177. Found 679.2178 $[M+H]^+$.

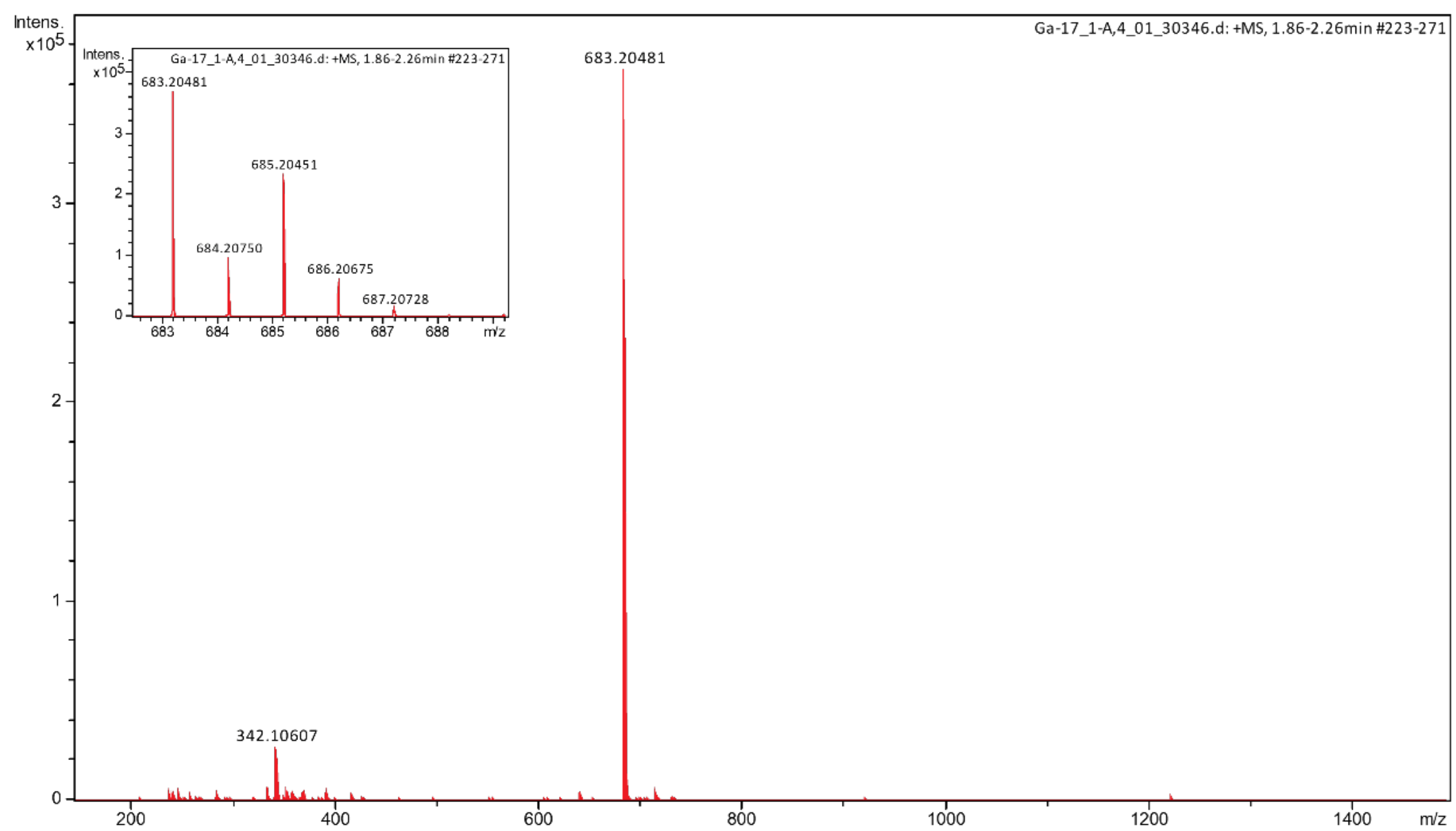

Figure S40. HRESI-MS of $[Ga(NO2A)]^+$-^{NMe}G-G-W, $[Ga(2)]^+$. HRMS calc. for $C_{28}H_{38}GaN_8O_8$ 683.20628. Found 683.20481 $[M]^+$.

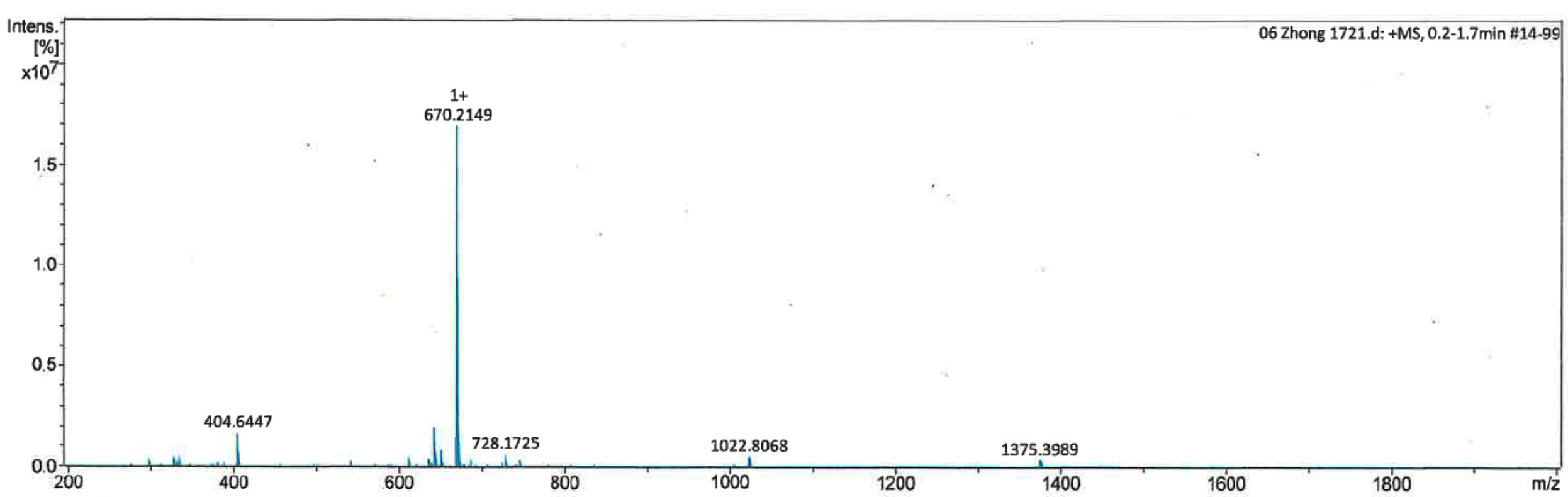

Figure S41. HRESI-MS of $[Fe(NO2A)]^+$-^{NMe}G-G-W, $[Fe(2)]^+$. HRMS calc. for C28H38FeN8O8 670.2157. Found 670.2149 $[M]^+$.

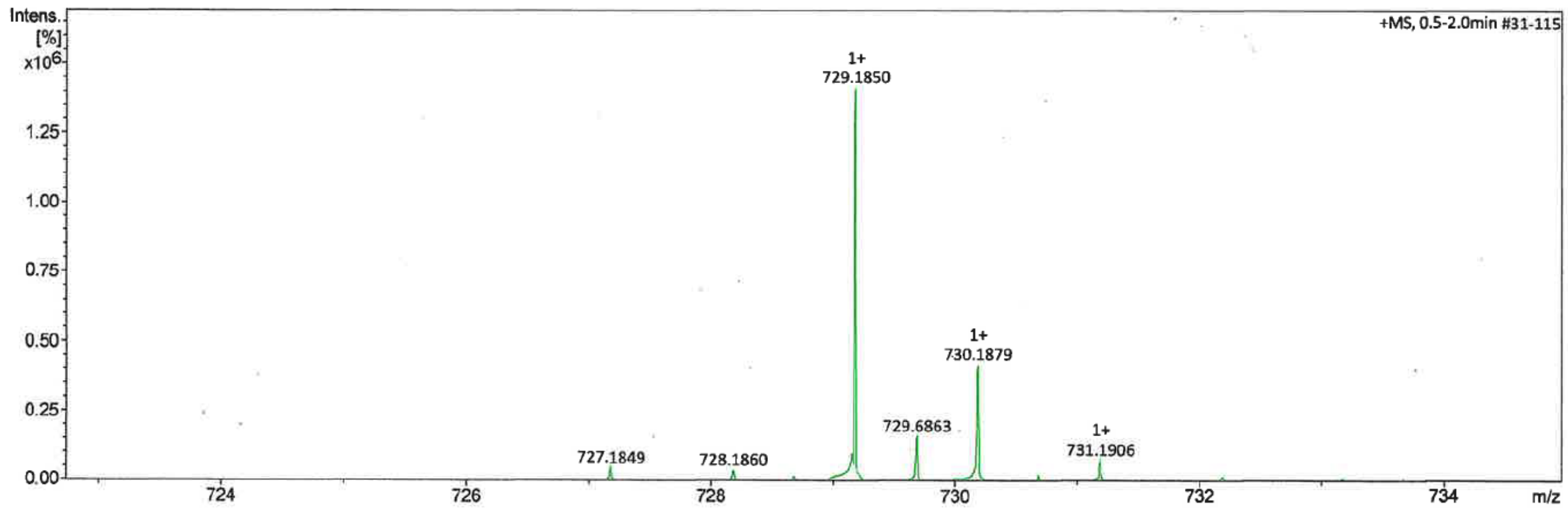

Figure S42. HRESI-MS of [In(NO2A)]$^{+}$-^{NMe}G-G-W, [In(2)]$^{+}$. HRMS calc. for $C_{28}H_{38}InN_8O_8$ 729.1846. Found 729.1850 [M]$^{+}$.

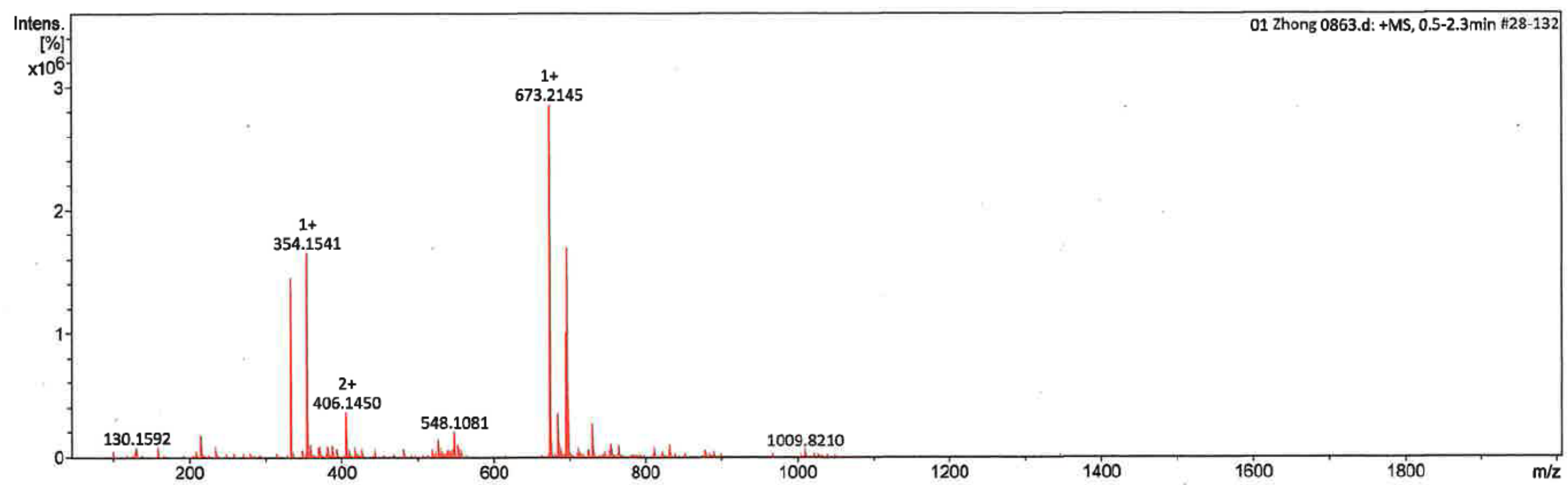

Figure S43. HRESI-MS of [Co(NO2A)]$^{+}$-^{NMe}G-G-W, [Co(2)]$^{+}$. HRESI calc. for $C_{28}H_{38}CoN_8O_8$ 673.2144. Found 673.2145 [M]$^{+}$.

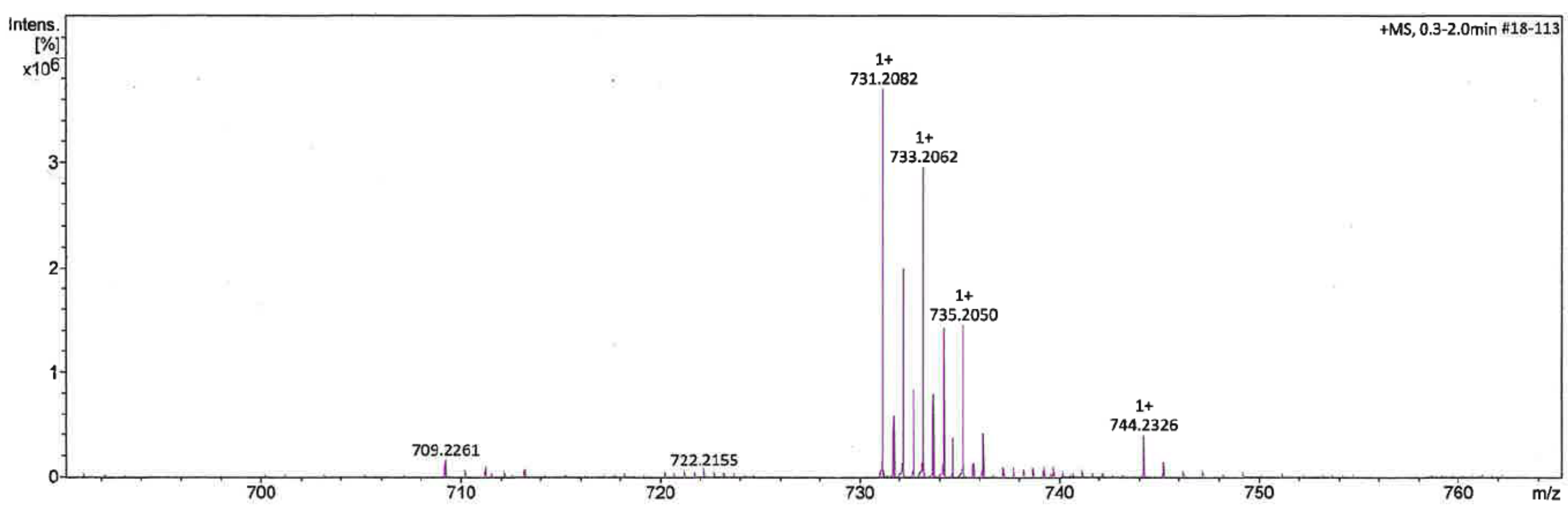

Figure S44. HRESI-MS of [Zn(NO2A)]-^{NMe}S-G-W, Zn(3). HRESI calc. for $C_{29}H_{40}ZnN_8O_9$ 708.2210 Found 731.2082 [M+Na]$^{+}$.

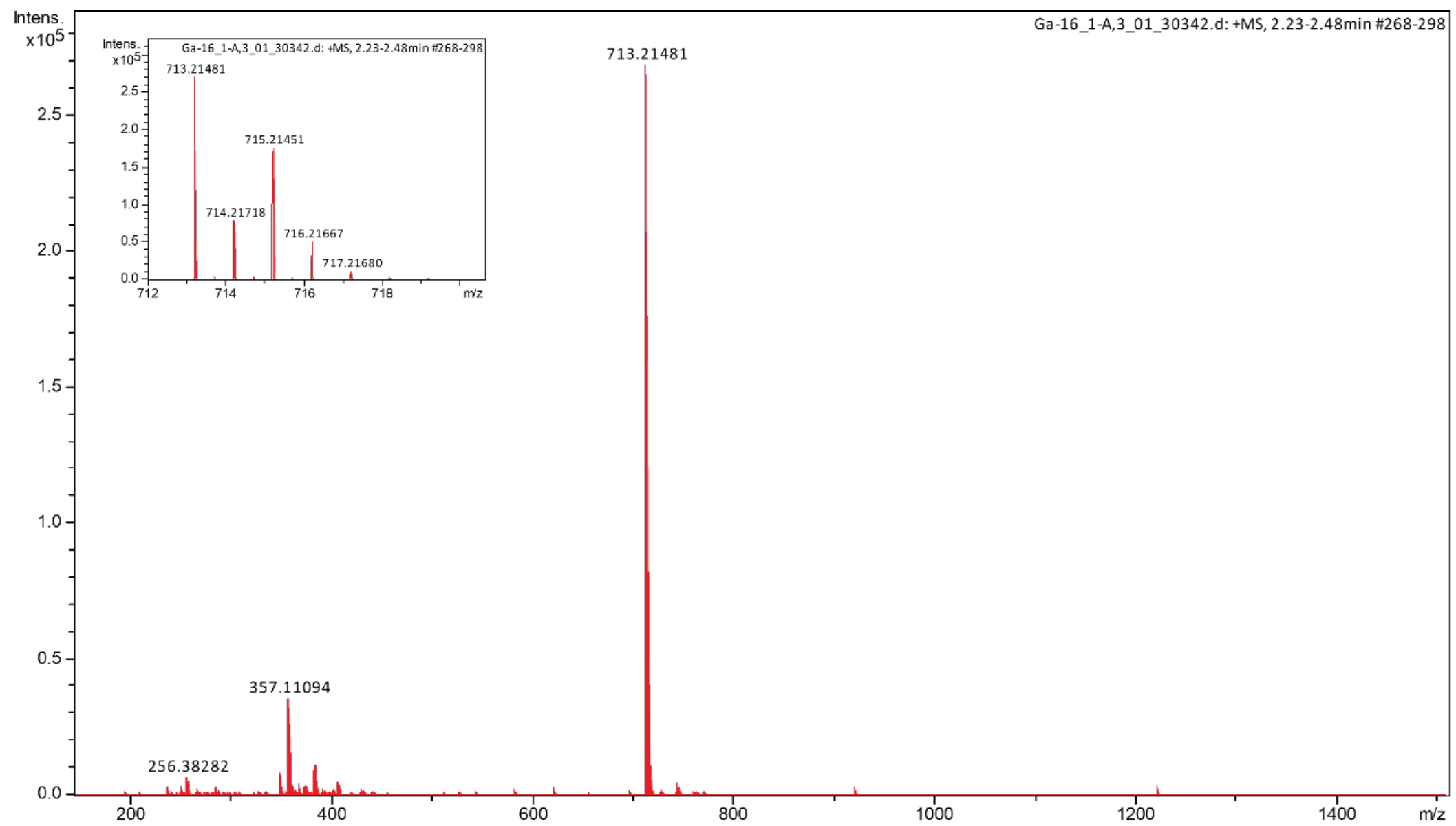

Figure S45. HRESI-MS of $[Ga(NO2A)]^{+}$-^{NMe}S-G-W, $[Ga(3)]^{+}$. HRESI calc. for $C_{29}H_{40}GaN_{8}O_{9}$ 713.2174. Found 713.21481 $[M]^{+}$.

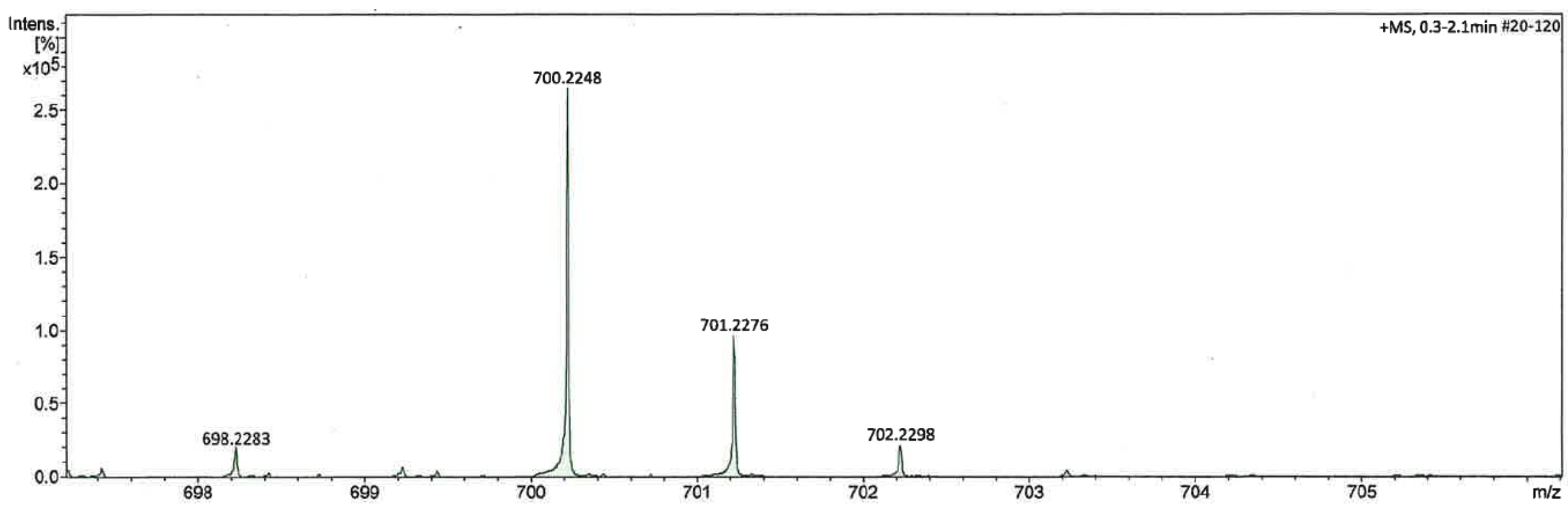

Figure S46. HRESI-MS of $[Fe(NO2A)]^{+}$-^{NMe}S-G-W, $[Fe(3)]^{+}$. HRESI calc. for $C_{29}H_{40}FeN_{8}O_{9}$ 700.2268. Found 700.2248 $[M]^{+}$.

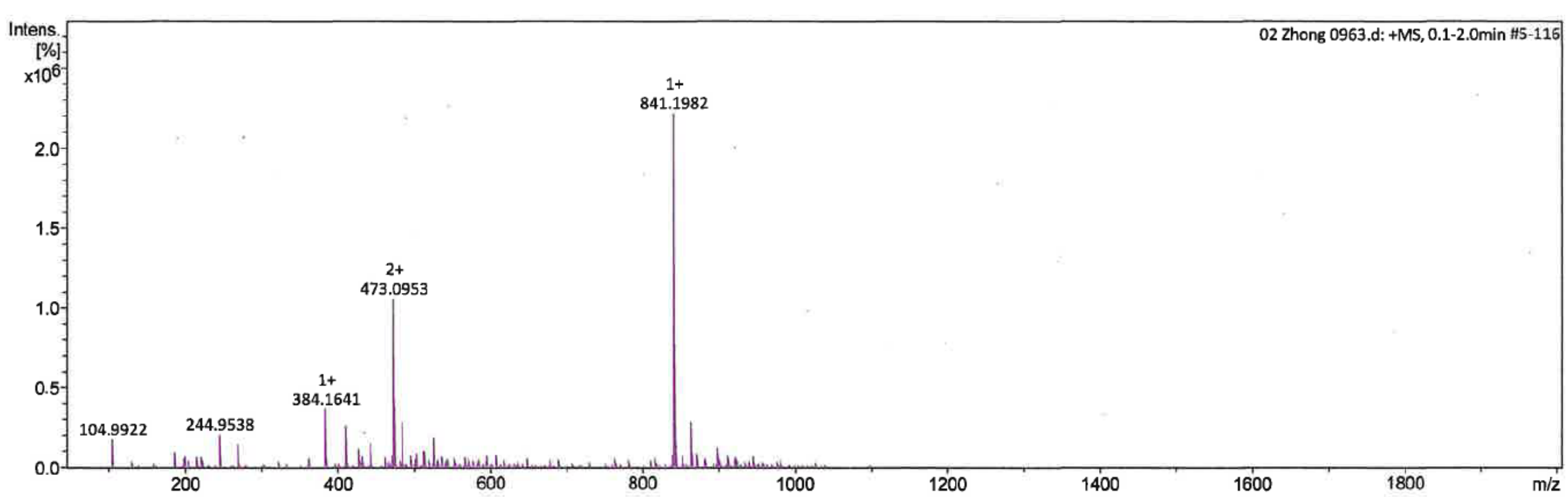

Figure S47. HRESI-MS of $[In(NO2A)]^{+}$-^{NMe}S-G-W, $[In(3)]^{+}$. HRESI calc. for $C_{29}H_{40}InN_{8}O_{9}$ 759.1957. Found 841.1982 $[M+CH_{3}CO_{2}Na]^{+}$.

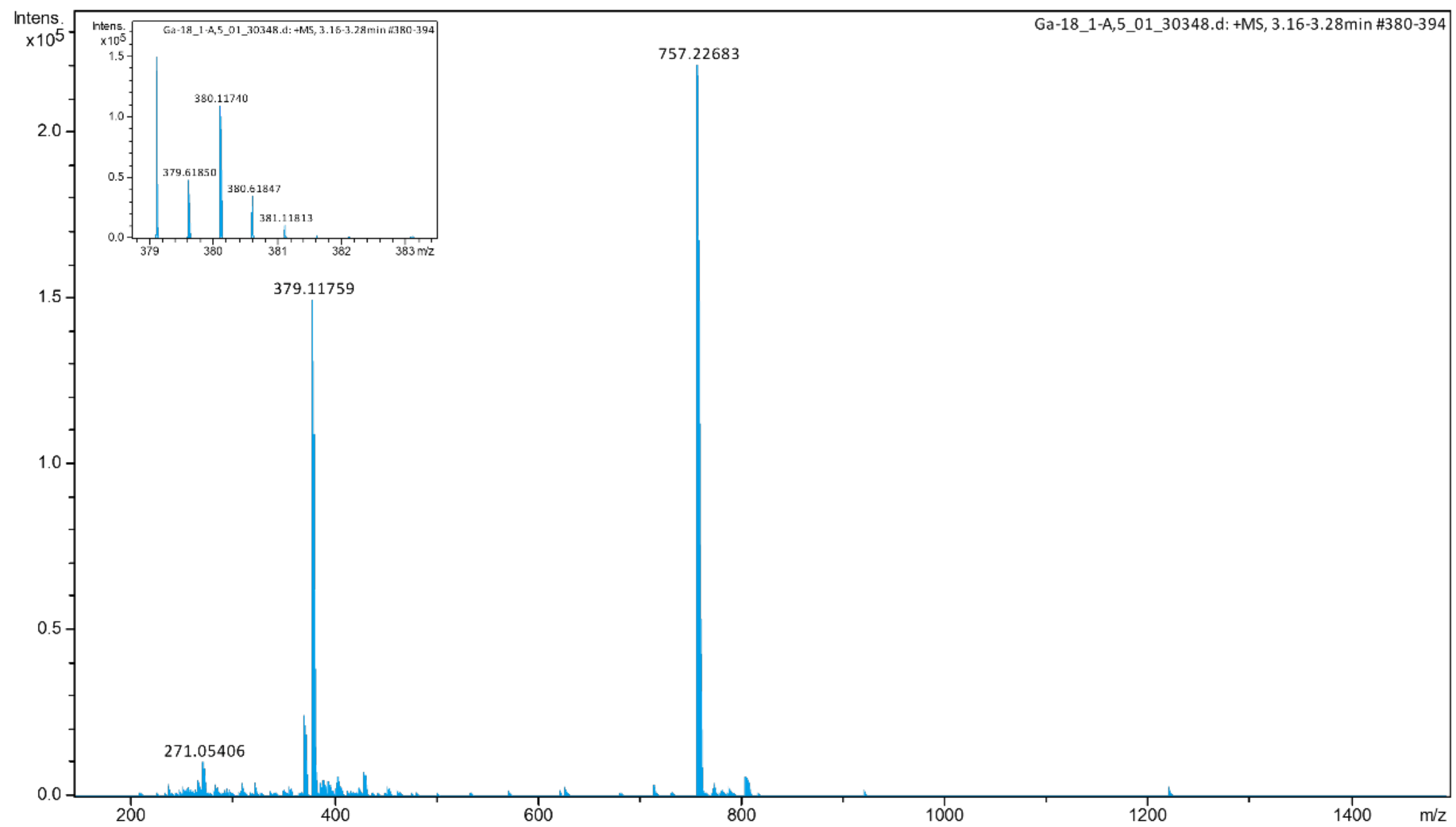

Figure S48. HRESI-MS of $[Ga(NO2A)]^{+}$-^{NMe}M-G-W, $[Ga(4)]^{+}$. HRESI of $C_{31}H_{44}GaN_8O_8S$ 757.22530, found 757.22683 $[M]^{+}$.

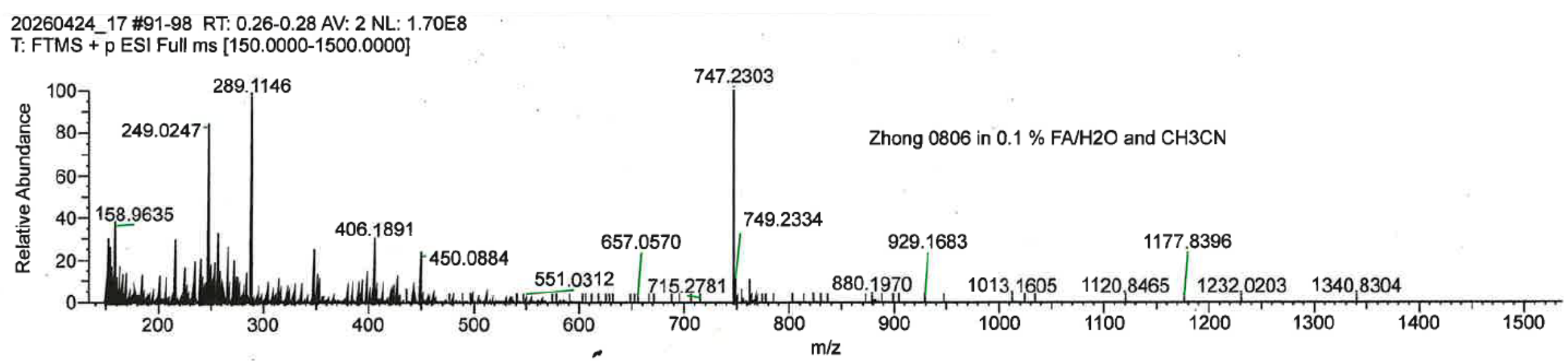

Figure S49. HRESI-MS of $[Co(NO2A)]^{+}$-^{NMe}Met-G-W, $[Co(4)]^{+}$. HRESI of $C_{31}H_{44}CoN_8O_8S$ 747.2335, found 747.2303 $[M]^{+}$.

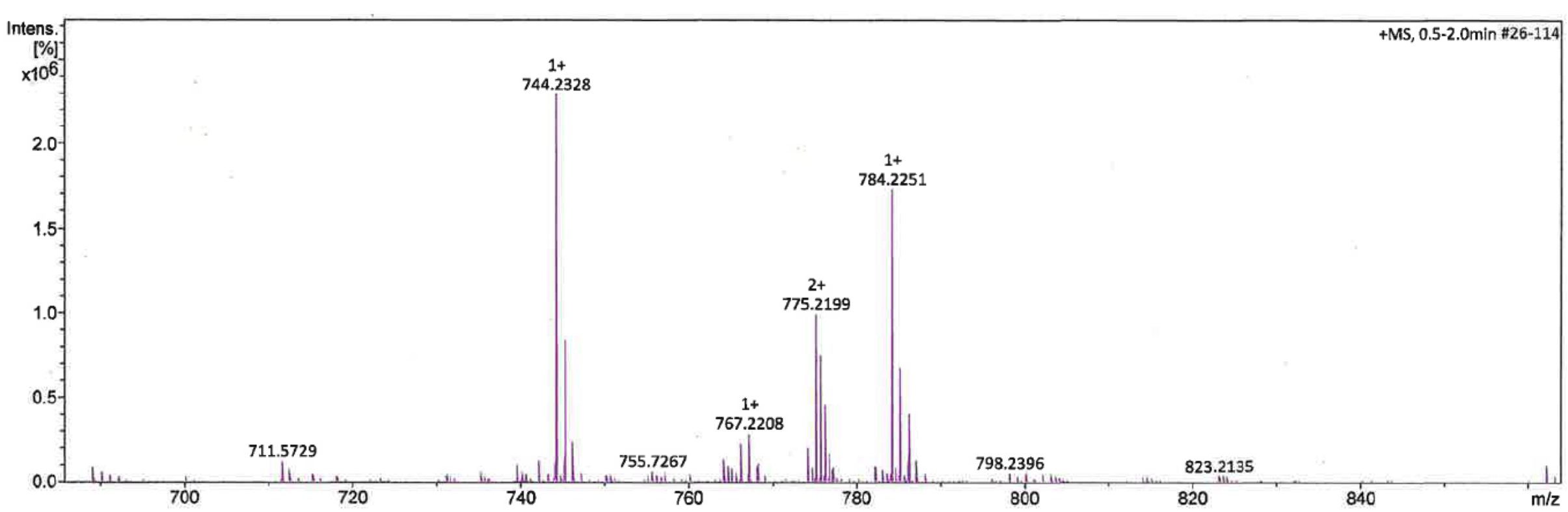

Figure S50. HRESI-MS of $[Fe(NO2A)]^{+}$-^{NMe}M-G-W, $[Fe(4)]^{+}$. HRESI of $C_{31}H_{44}FeN_8O_8S$ 744.2352, found 744.2328 $[M]^{+}$.

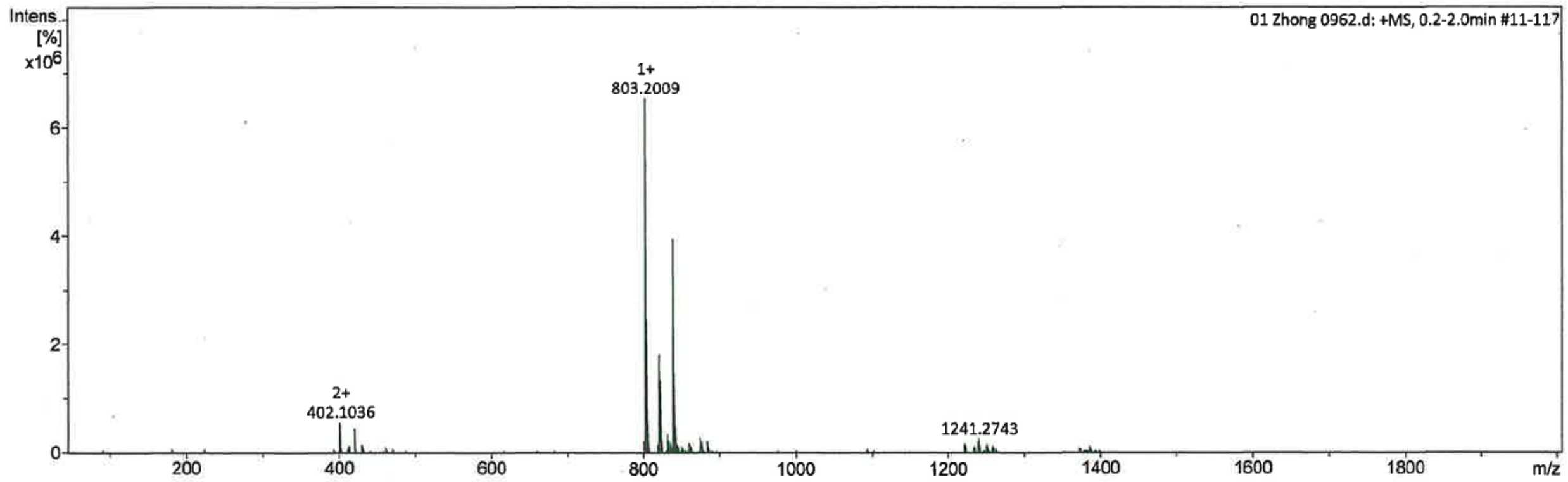

Figure S51. HRESI-MS of [In(NO2A)]$^{+}$-^{NMe}M-G-W, [In(4)]$^{+}$. HRESI of $C_{31}H_{44}InN_8O_8S$ 803.2042, found 803.2009 [M]$^{+}$.

4.3 Cleavage assays of model tripeptide complexes

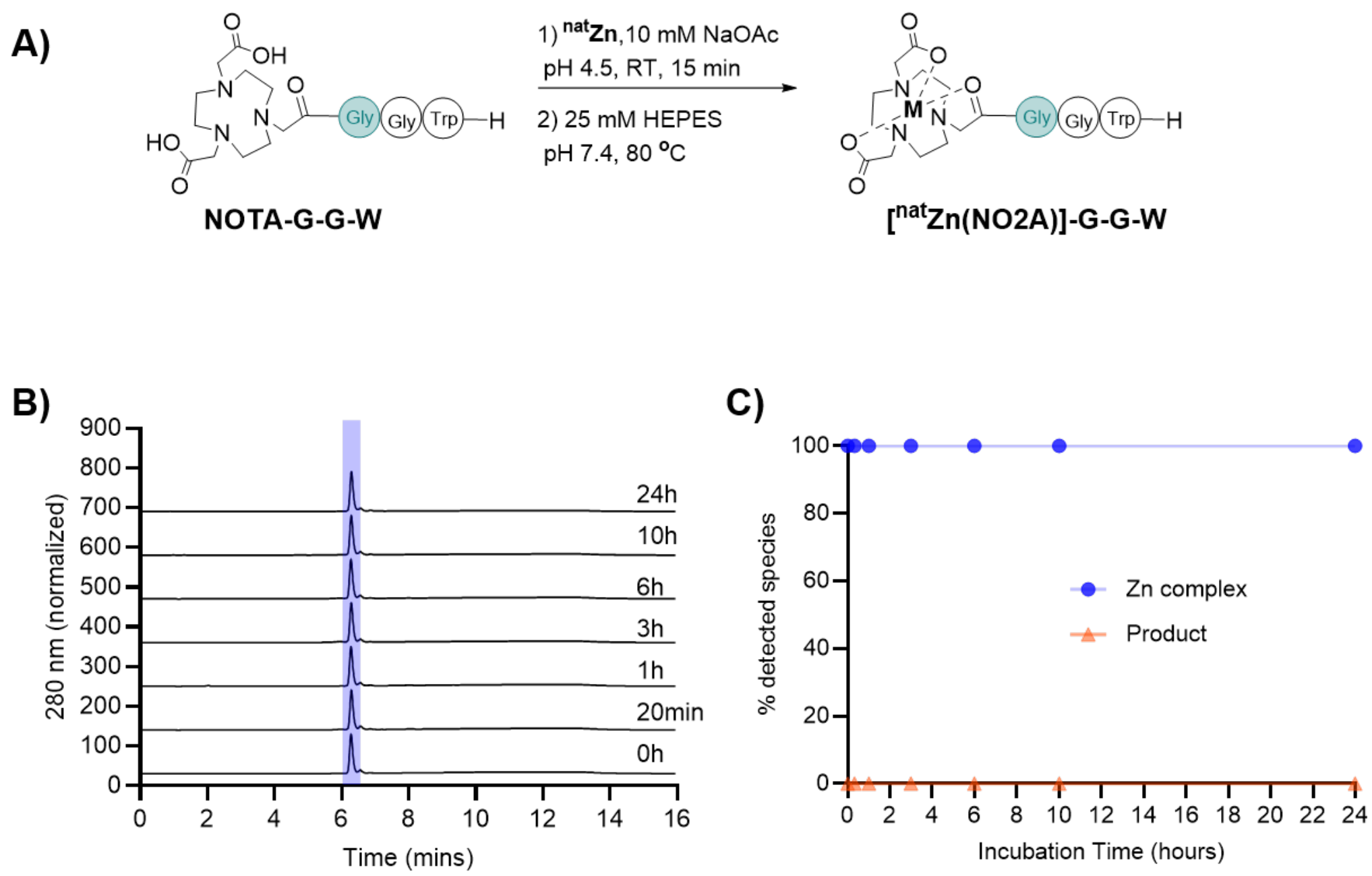

Figure S52. (A) Synthesis scheme and conditions for cleavage of **natZn(1)**. **(B)** Analytical HPLC chromatograms (Method B) show no autolytic release of the natZn-NOTA. **(C)** Quantification of the pH dependent cleavage at 80 ºC and pH 7.4.

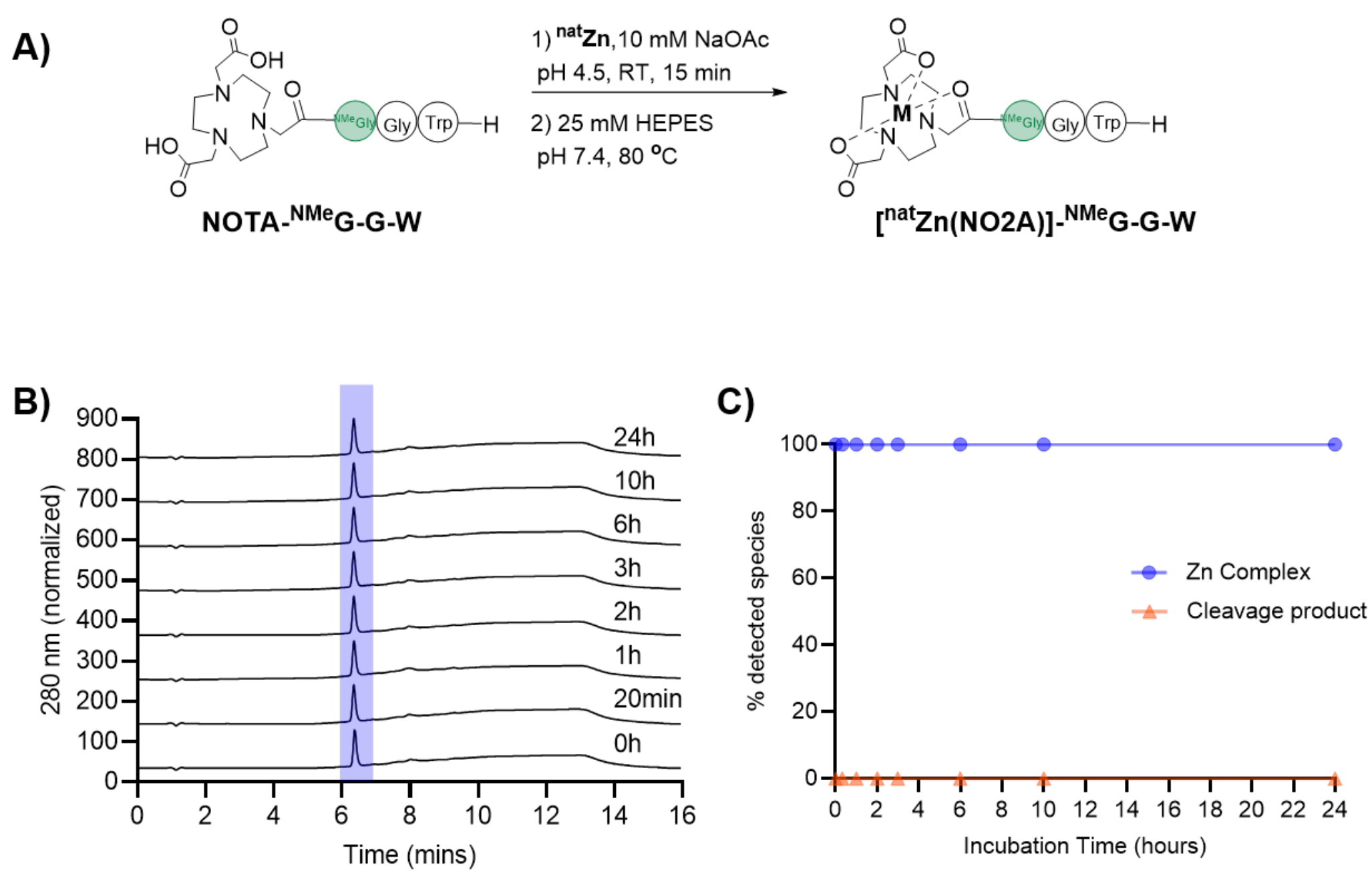

Figure S53. (A) Synthesis scheme and conditions for cleavage of **natZn(2)**. **(B)** Analytical HPLC chromatograms (Method B) show no autolytic release of the natZn-NOTA. **(C)** Quantification of the pH-dependent cleavage at 80 ºC and pH 7.4.

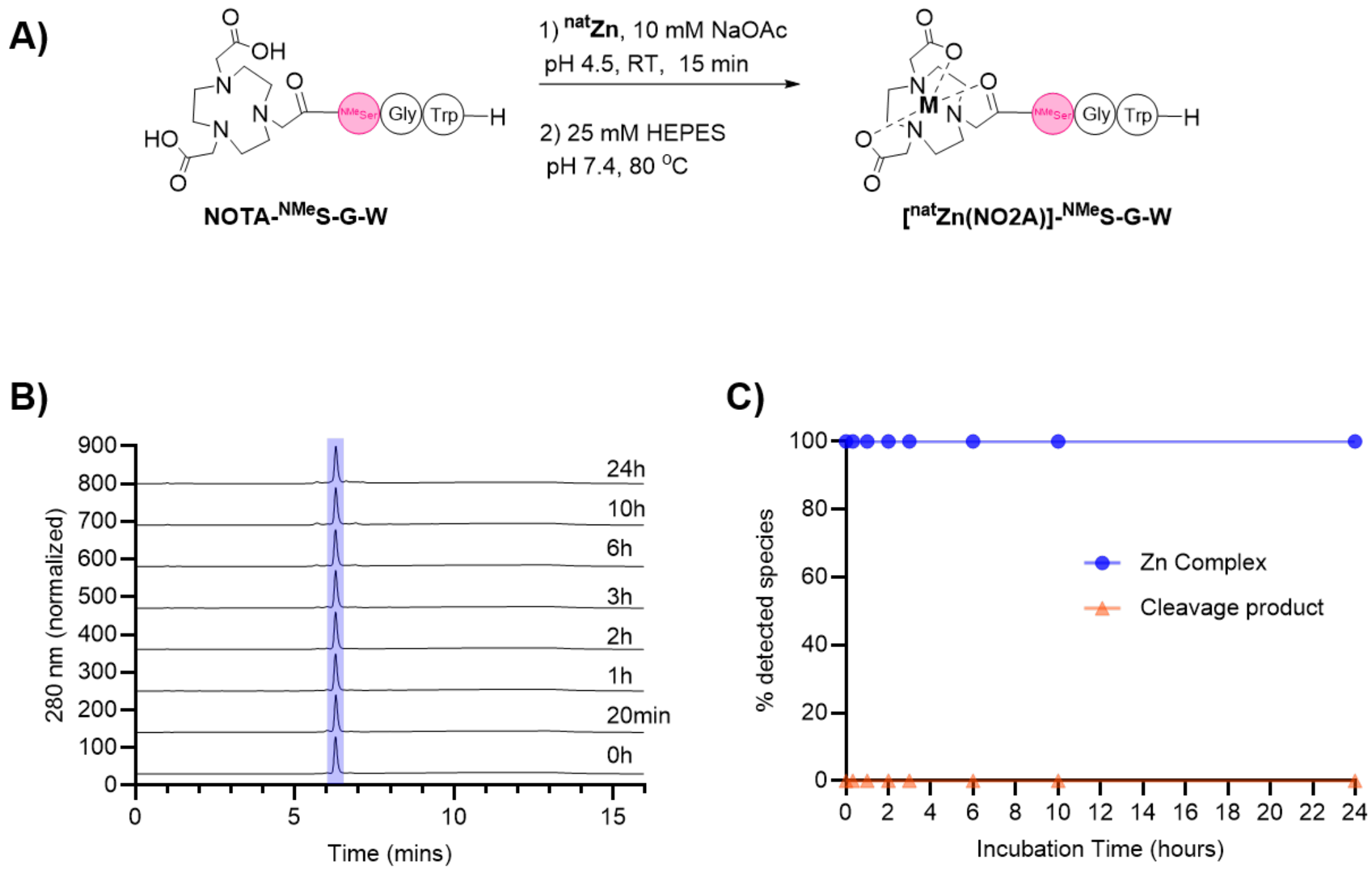

Figure S54. **(A)** Synthesis scheme and conditions for cleavage of **^{nat}Zn(3)**. **(B)** Analytical HPLC chromatograms (Method B) show no autolytic release of the ^{nat}Zn-NOTA. **(C)** Quantification of the pH-dependent cleavage at 80 ºC and pH 7.4.

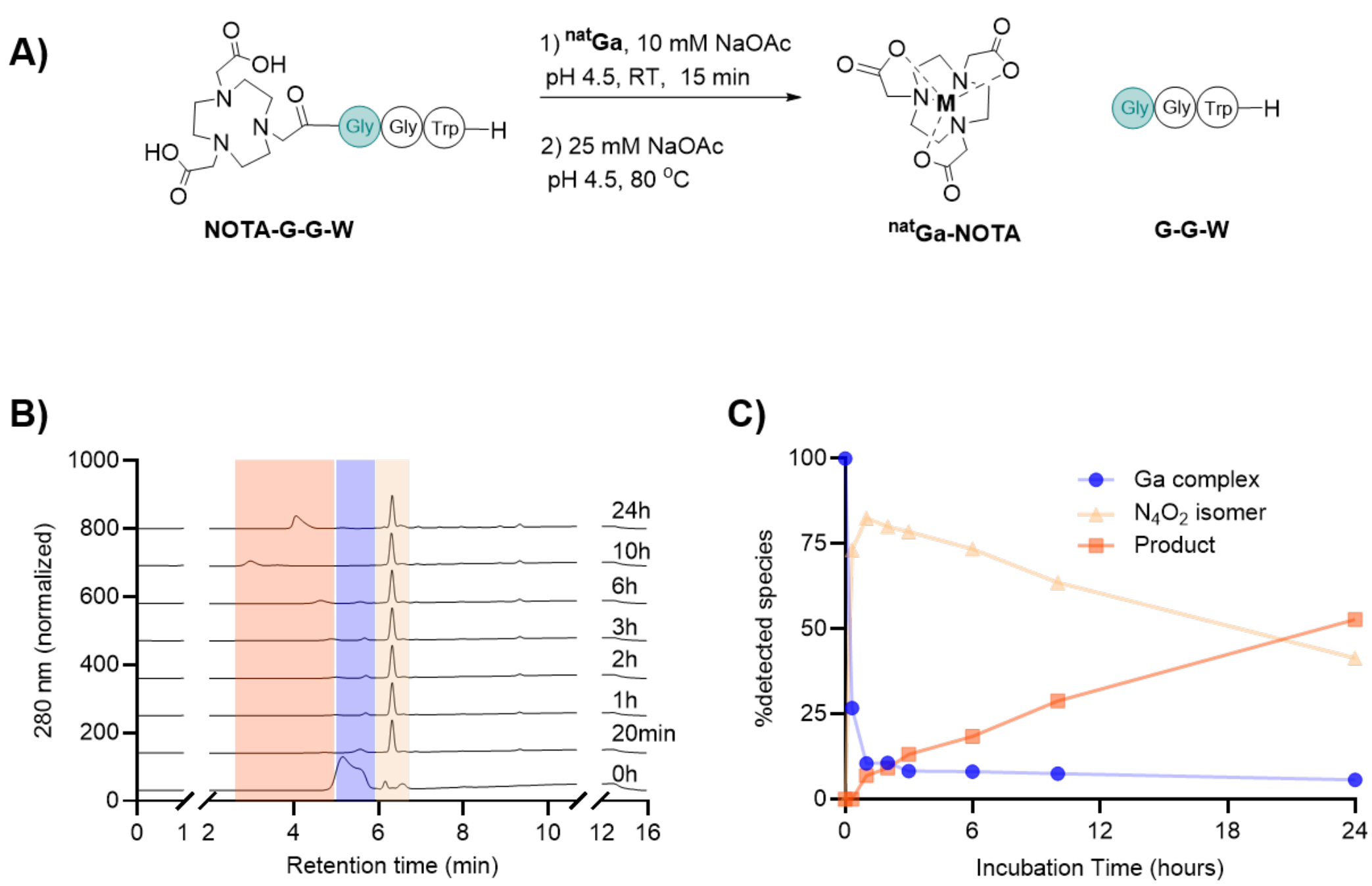

Figure S55. **(A)** Synthesis scheme and conditions for cleavage of **[^{nat}Ga(1)]$^{+}$**. **(B)** Analytical HPLC chromatograms (Method B) show the autolytic release of the ^{nat}Ga-NOTA. **(C)** Quantification of the pH-dependent cleavage at 80 ºC and pH 4.5.

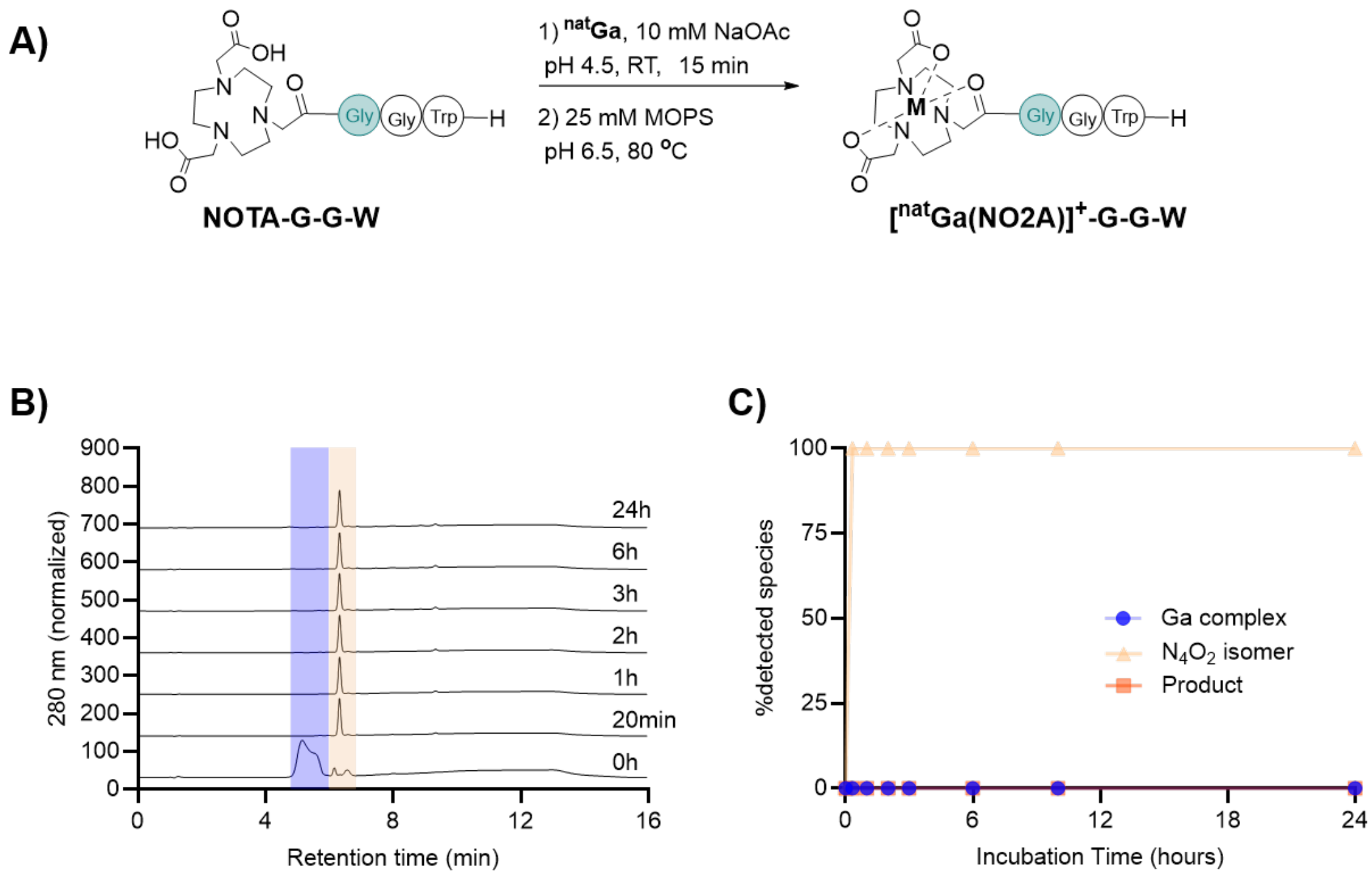

Figure S56. (A) Synthesis scheme and conditions for cleavage of **[^{nat}Ga(1)]$^{+}$**. **(B)** Analytical HPLC chromatograms (Method B) show no autolytic release of the ^{nat}Ga-NOTA. **(C)** Quantification of the pH-dependent cleavage at 80 ºC and pH 6.5.

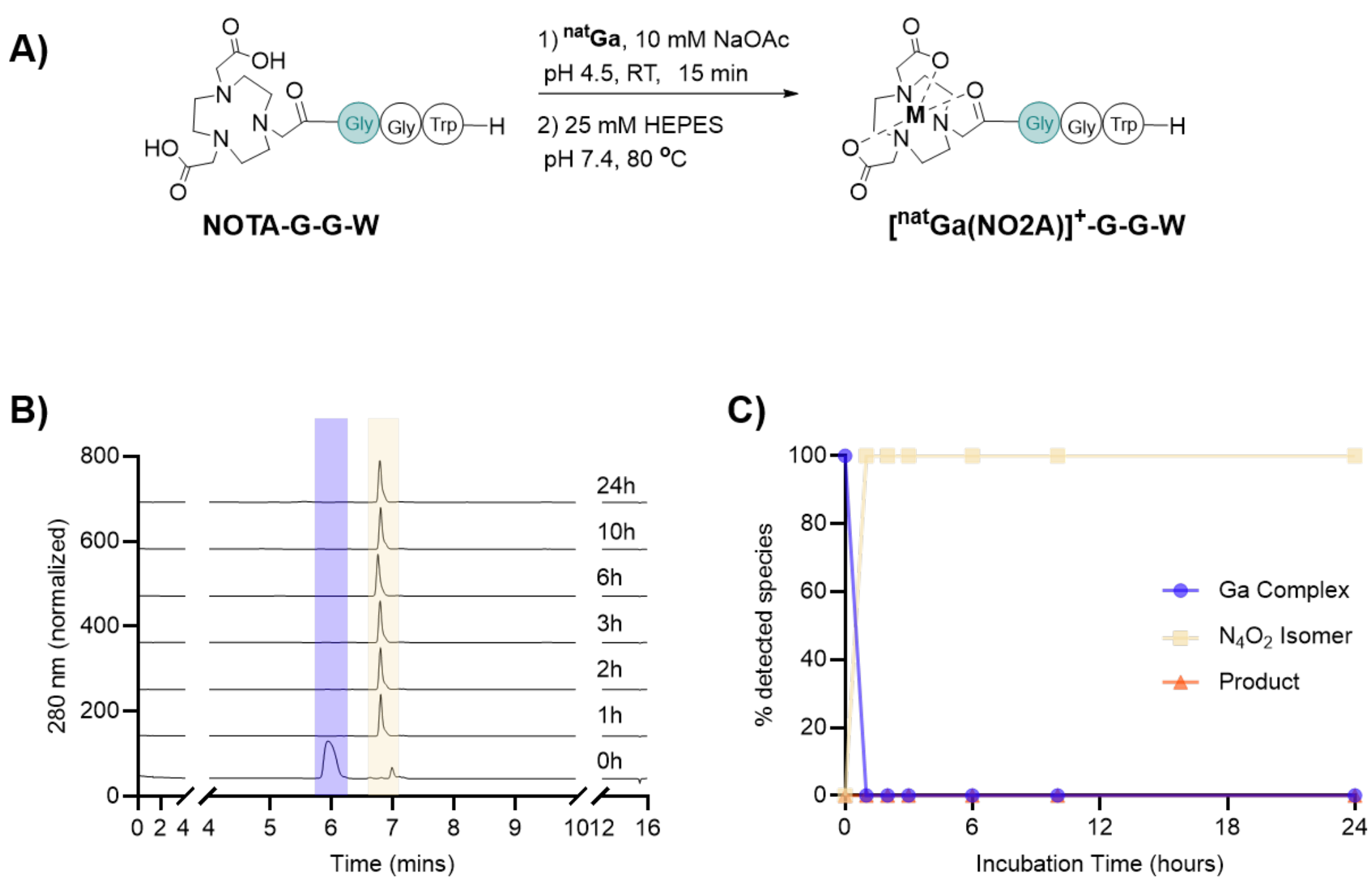

Figure S57. (A) Synthesis scheme and conditions for cleavage of **[^{nat}Ga(1)]$^{+}$**. **(B)** Analytical HPLC chromatograms (Method B) show no autolytic release of the ^{nat}Ga-NOTA. **(C)** Quantification of the pH-dependent cleavage at 80 ºC and pH 7.4.

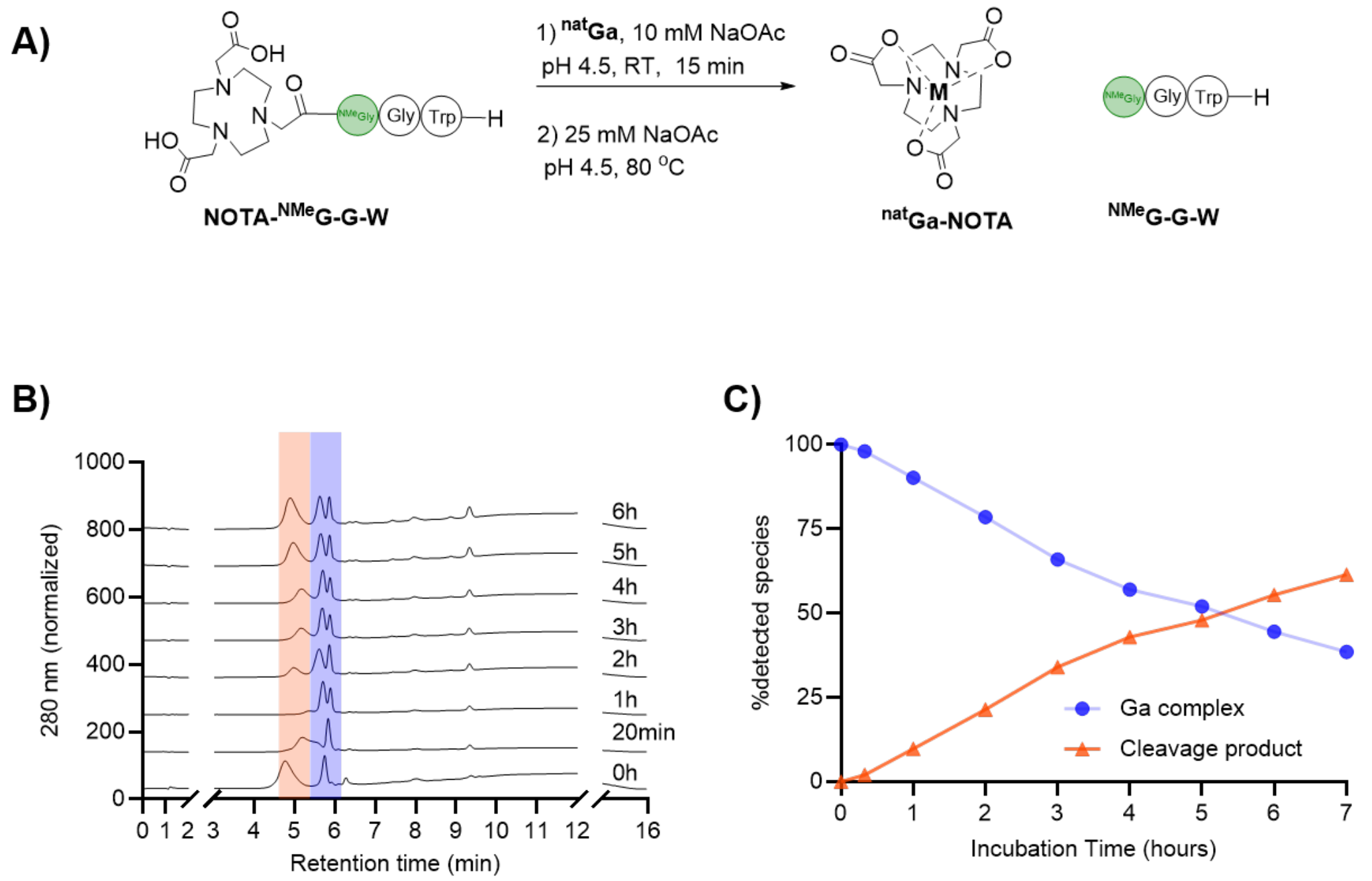

Figure S58. (A) Synthesis scheme and conditions for cleavage of **[natGa(2)]+**. **(B)** Analytical HPLC chromatograms (Method B) show the autolytic release of the natGa-NOTA. **(C)** Quantification of the pH-dependent cleavage at 80 ºC and pH 4.5.

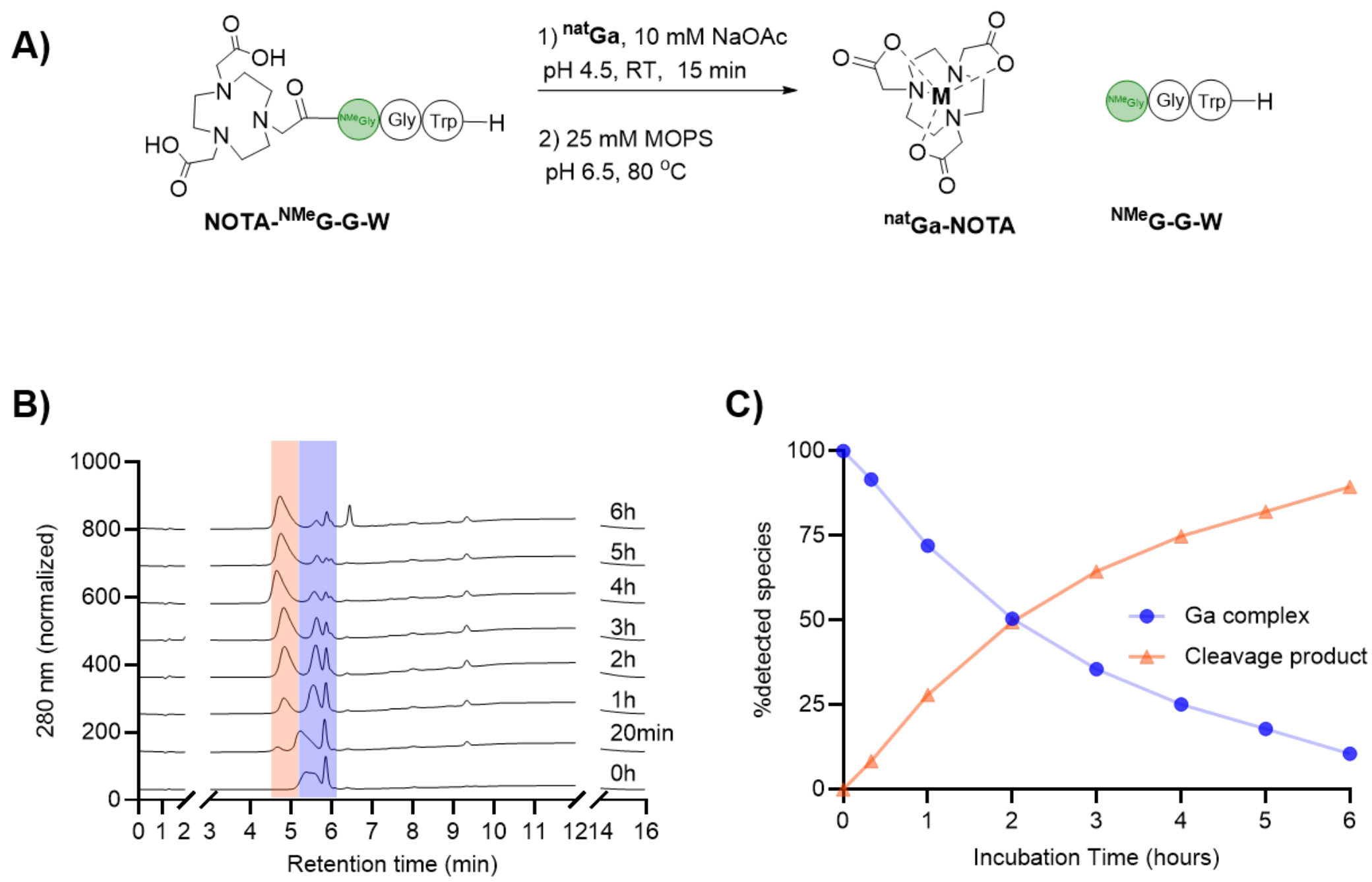

Figure S59. (A) Synthesis scheme and conditions for cleavage of **[natGa(2)]+**. **(B)** Analytical HPLC chromatograms (Method B) show the autolytic release of the natGa-NOTA. **(C)** Quantification of the pH-dependent cleavage at 80 ºC and pH 6.5.

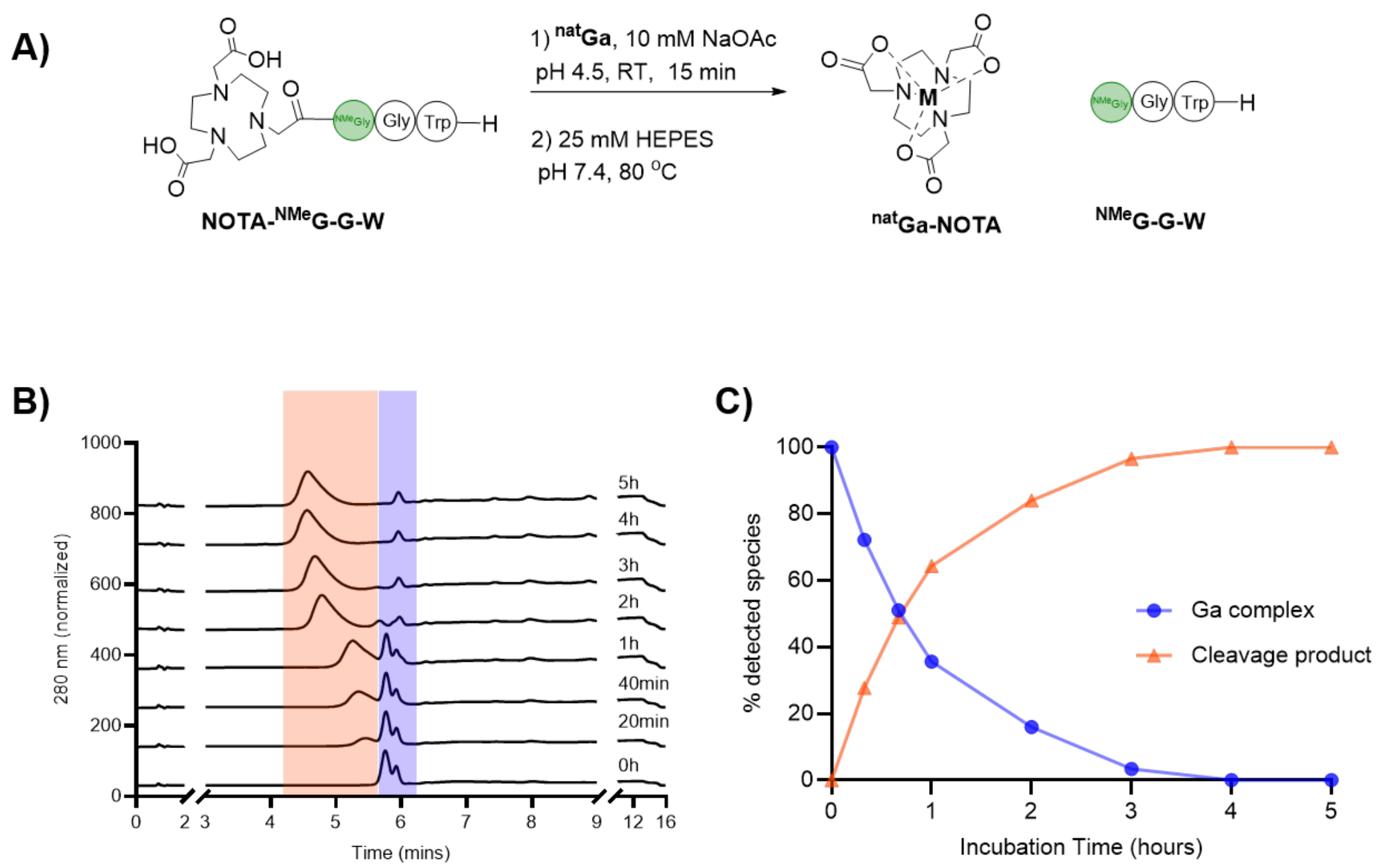

Figure S60. (A) Synthesis scheme and conditions for cleavage of **[^{nat}Ga(2)]$^{+}$**. **(B)** Analytical HPLC chromatograms (Method B) show the autolytic release of the ^{nat}Ga-NOTA. **(C)** Quantification of the pH-dependent cleavage at 80 ºC and pH 7.4.

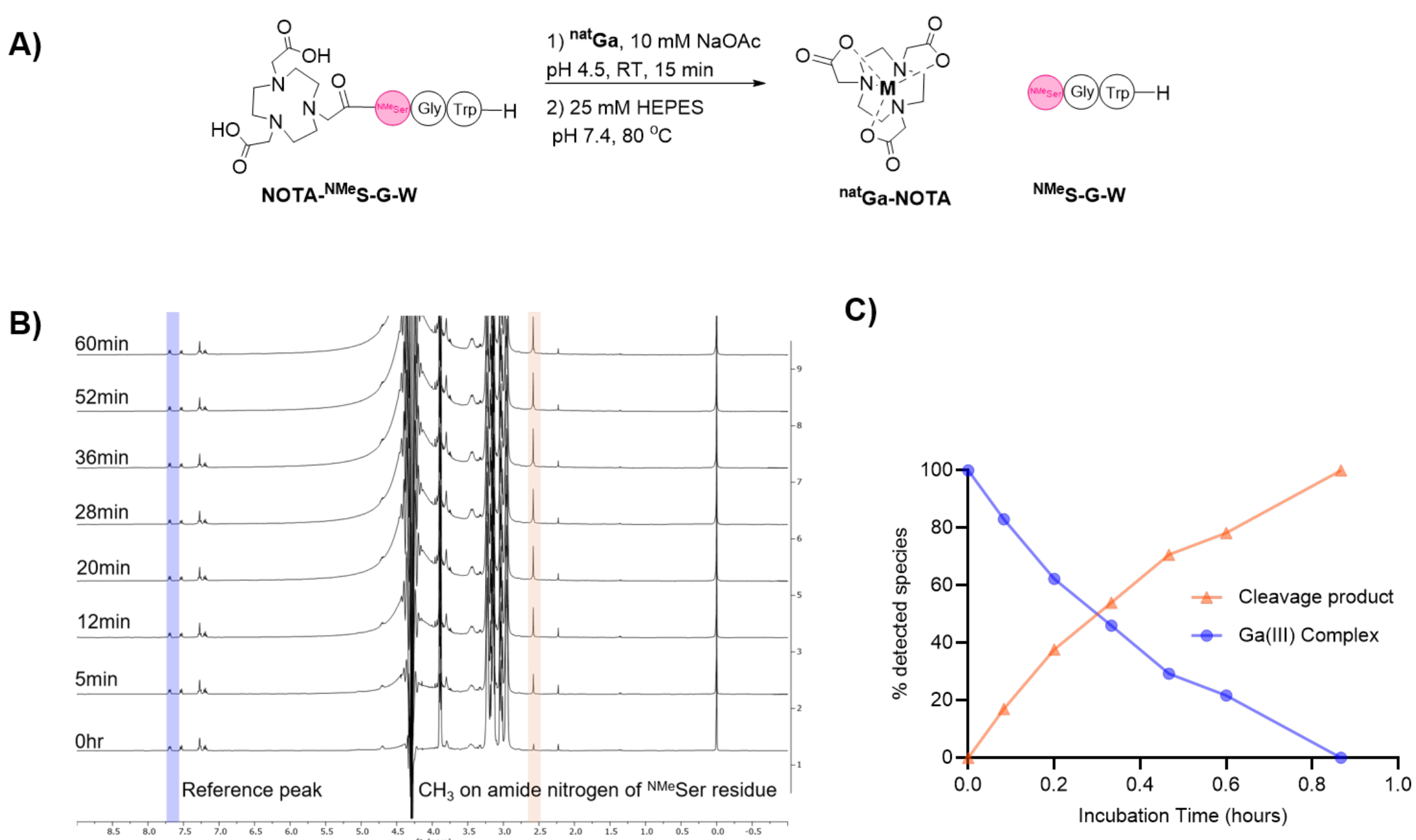

Figure S61. (A) Synthesis scheme and conditions for cleavage of **[^{nat}Ga(3)]$^{+}$**. **(B)** Stacked VT-NMR spectra (80 ºC) show the autolytic release of the ^{nat}Ga-NOTA. **(C)** Quantification of the pH-dependent cleavage at 80 ºC and pH 7.4 based on the integration of CH_3 peak chemical shift highlighted in the orange box.

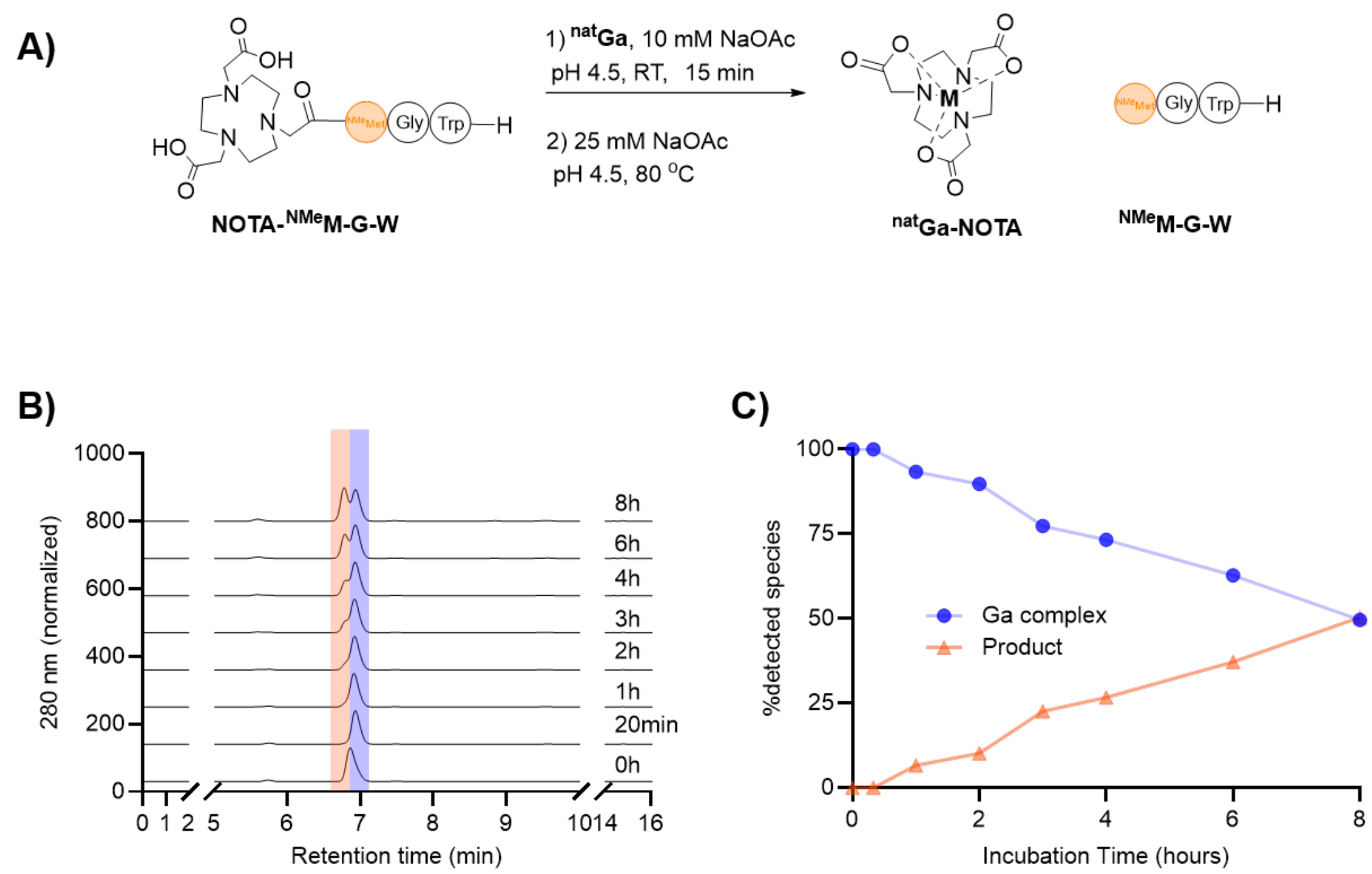

Figure S62. (A) Synthesis scheme and conditions for cleavage of **[^{nat}Ga(4)]$^{+}$**. **(B)** Analytical HPLC chromatograms (Method B) show the autolytic release of the ^{nat}Ga-NOTA. **(C)** Quantification of the pH-dependent cleavage at 80 ºC and pH 4.5.

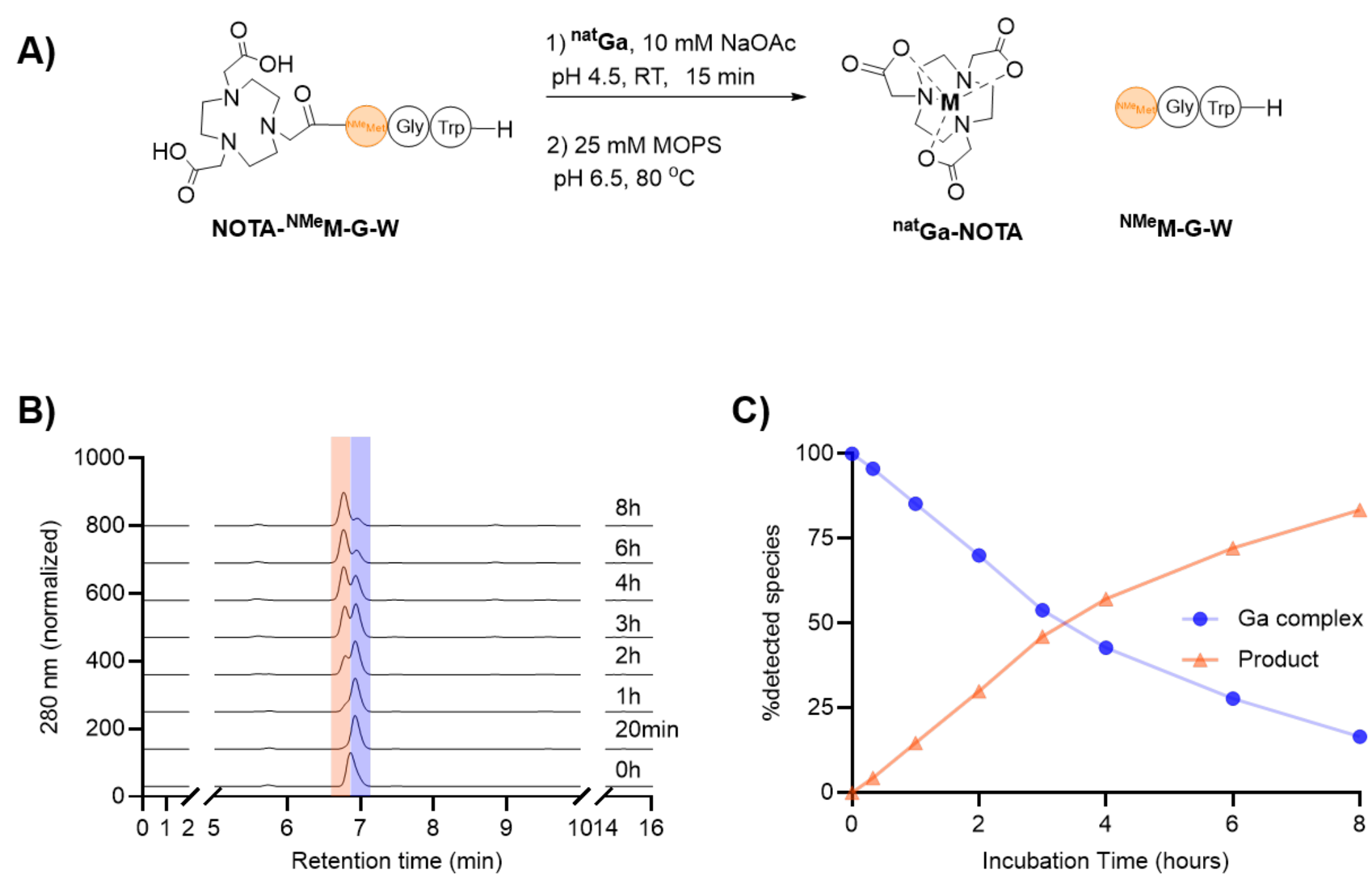

Figure S63. (A) Synthesis scheme and conditions for cleavage of **[^{nat}Ga(4)]$^{+}$**. **(B)** Analytical HPLC chromatograms (Method B) show the autolytic release of the ^{nat}Ga-NOTA. **(C)** Quantification of the pH-dependent cleavage at 80 ºC and pH 6.5.

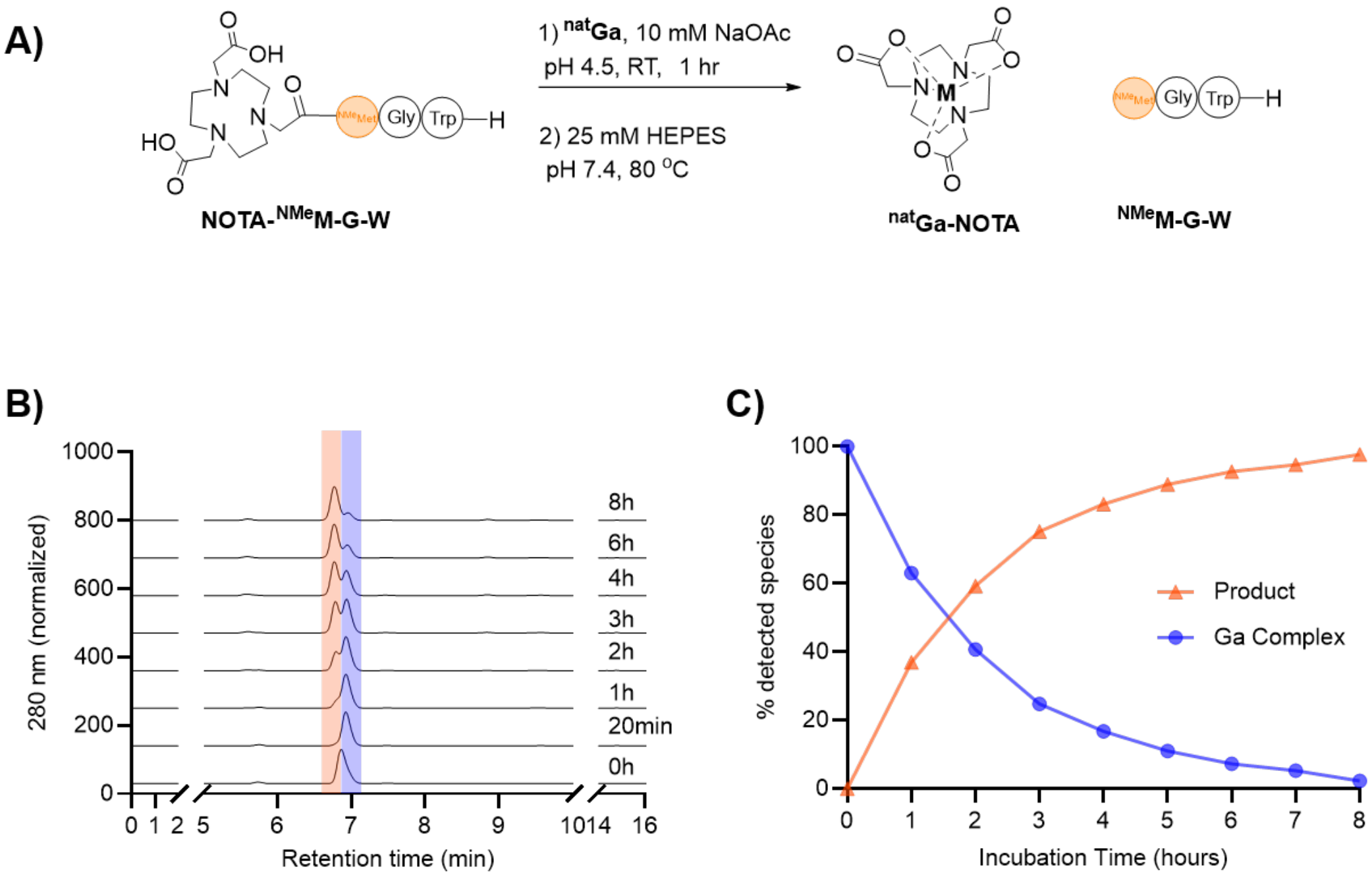

Figure S64. (A) Synthesis scheme and conditions for cleavage of **[natGa(4)]**$^{+}$. **(B)** Analytical HPLC chromatograms (Method B) show the autolytic release of the natGa-NOTA. **(C)** Quantification of the pH-dependent cleavage at 80 °C and pH 7.4.

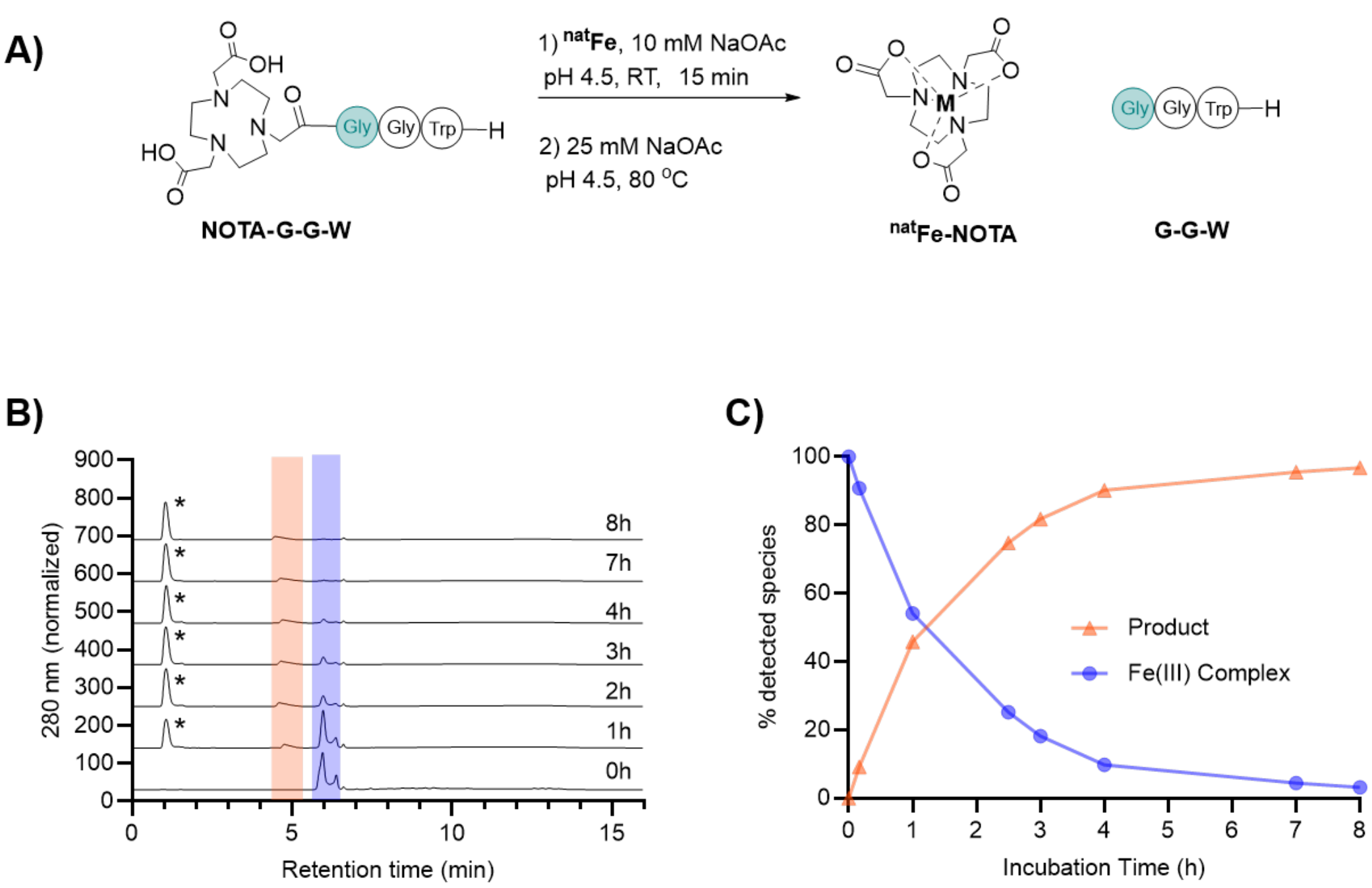

Figure S65. (A) Synthesis scheme and conditions for cleavage of **[natFe(1)]**$^{+}$. **(B)** Analytical HPLC chromatograms (Method B) show the autolytic release of the natFe-NOTA (*). **(C)** Quantification of the pH-dependent cleavage at 80 °C and pH 4.5.

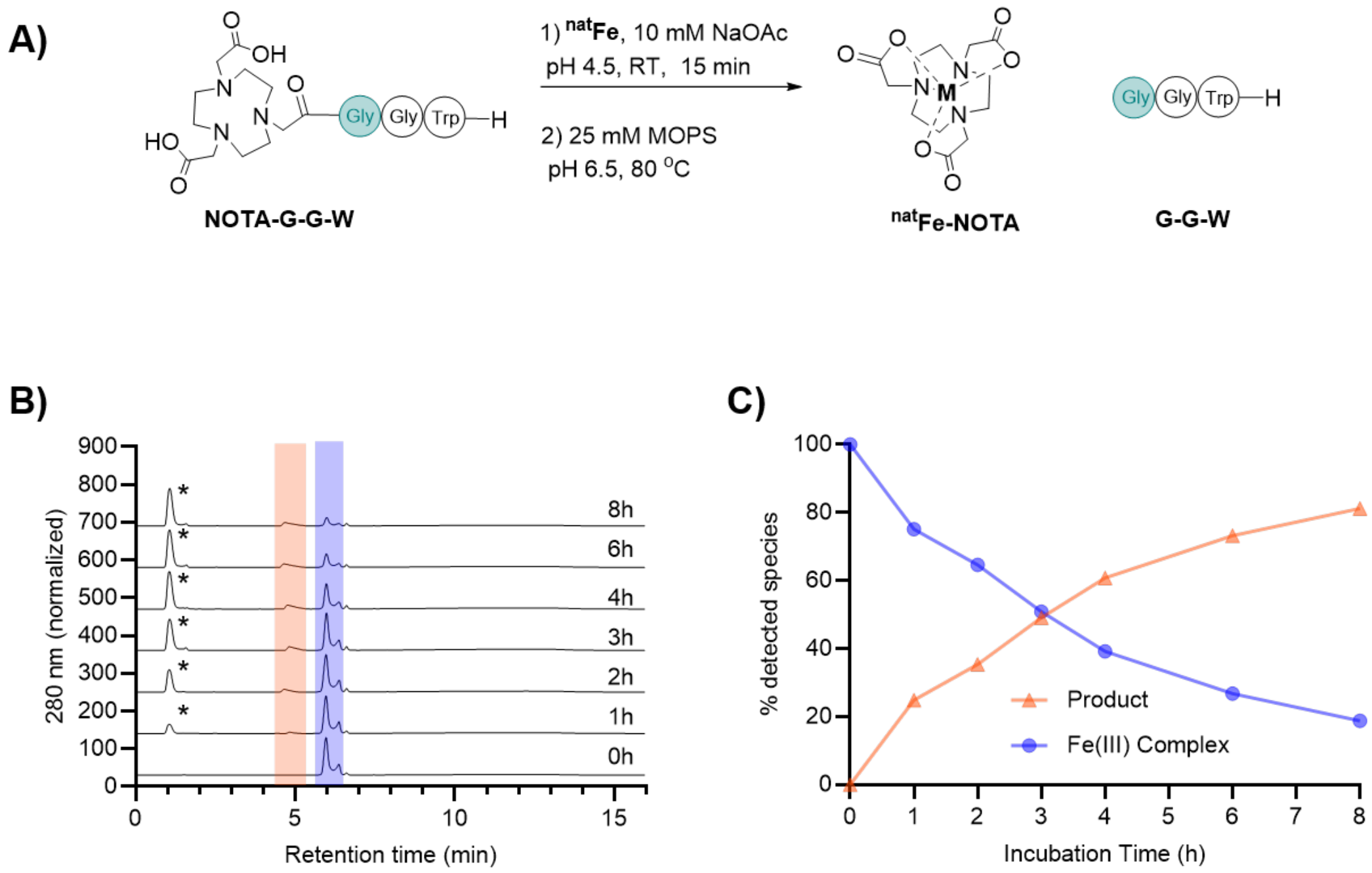

Figure S66. (A) Synthesis scheme and conditions for cleavage of **[natFe(1)]**$^{+}$. **(B)** Analytical HPLC chromatograms (Method B) show the autolytic release of the natFe-NOTA (*). **(C)** Quantification of the pH-dependent cleavage at 80 ºC and pH 6.5.

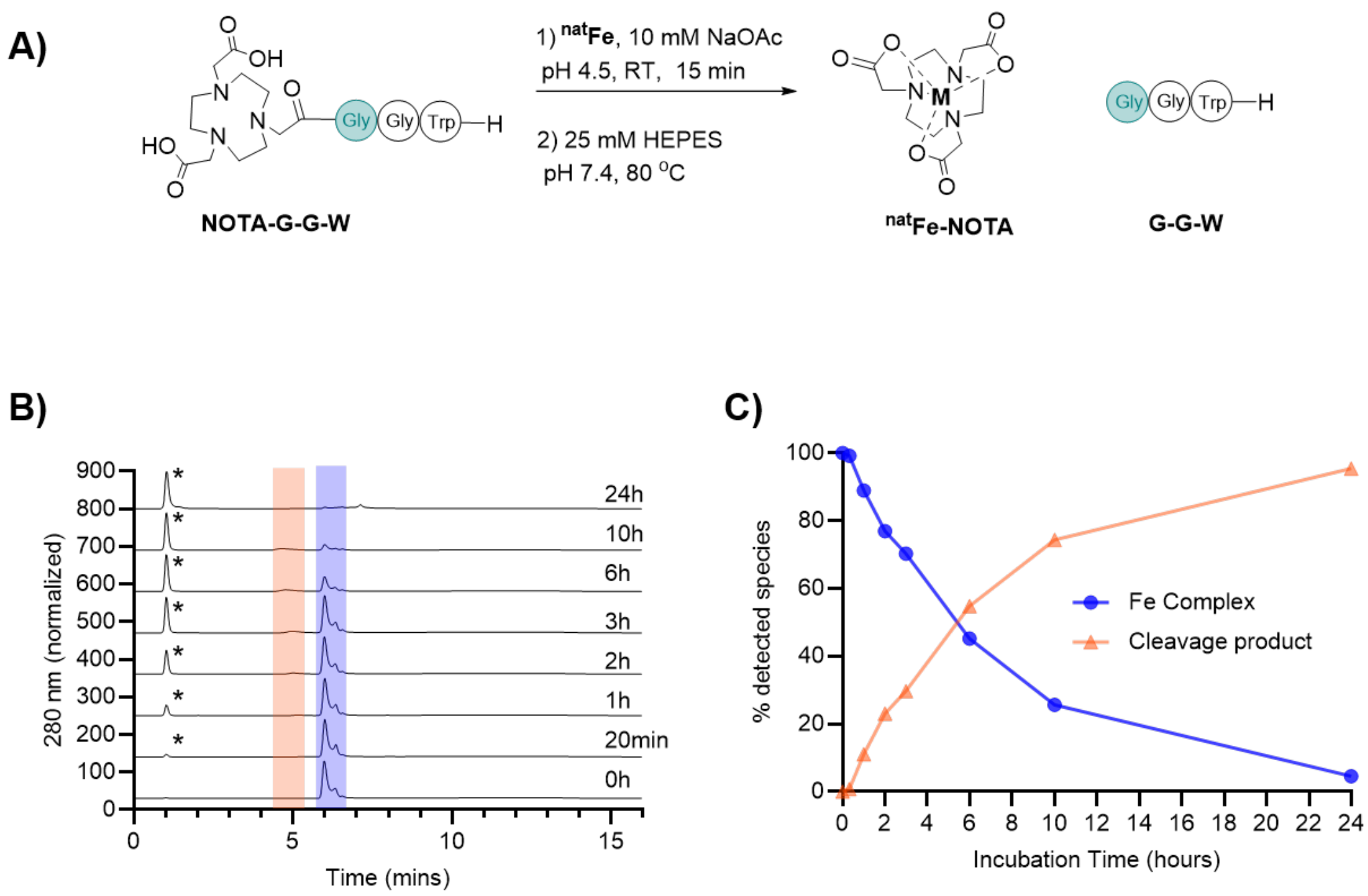

Figure S67. (A) Synthesis scheme and conditions for cleavage of **[natFe(1)]**$^{+}$. **(B)** Analytical HPLC chromatograms (Method B) show the autolytic release of the natFe-NOTA (*). **(C)** Quantification of the pH-dependent cleavage at 80 ºC and pH 7.4.

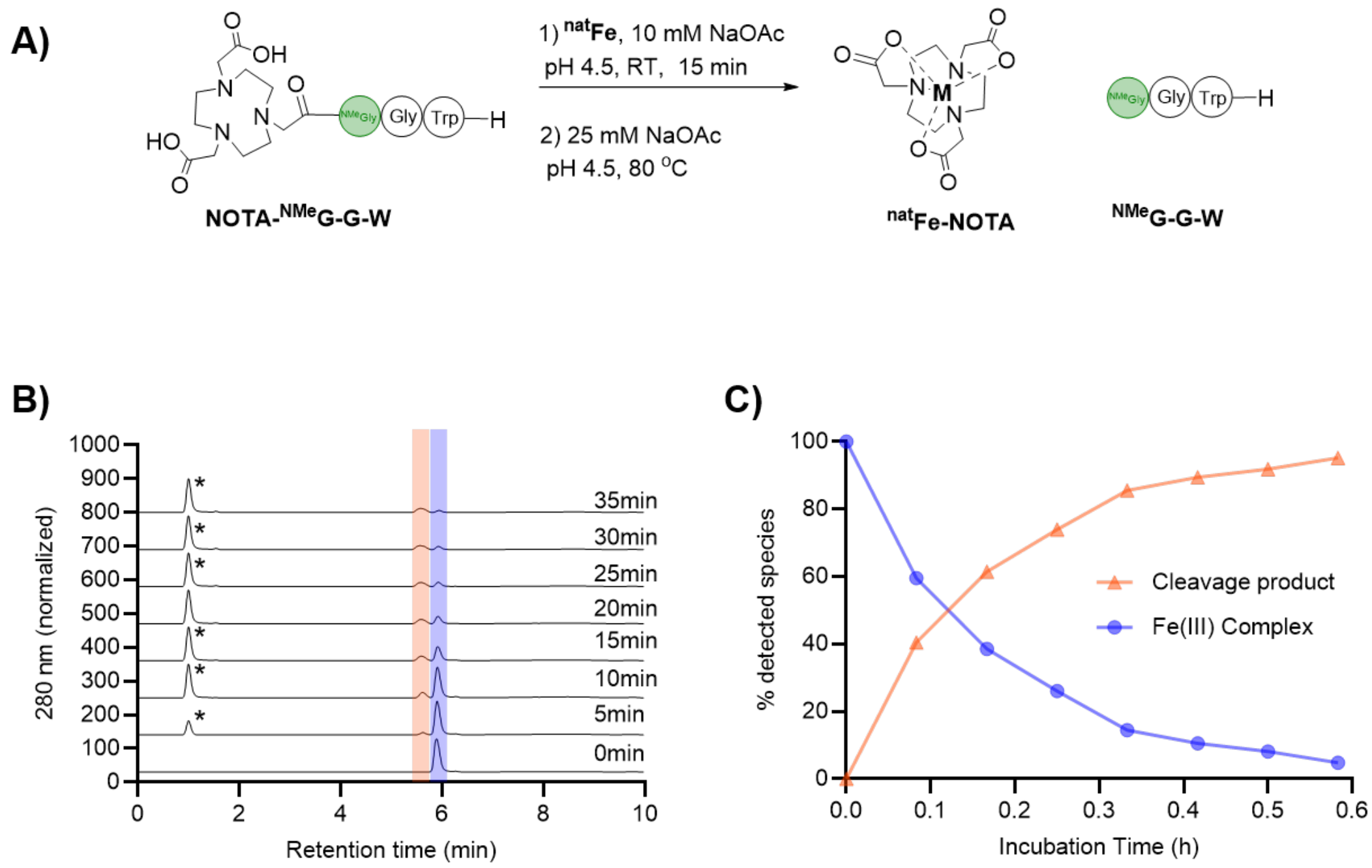

Figure S68. (A) Synthesis scheme and conditions for cleavage of **[natFe(2)]**$^{+}$. **(B)** Analytical HPLC chromatograms (Method C) show the autolytic release of the natFe-NOTA (*). **(C)** Quantification of the pH-dependent cleavage at 80 ºC and pH 4.5.

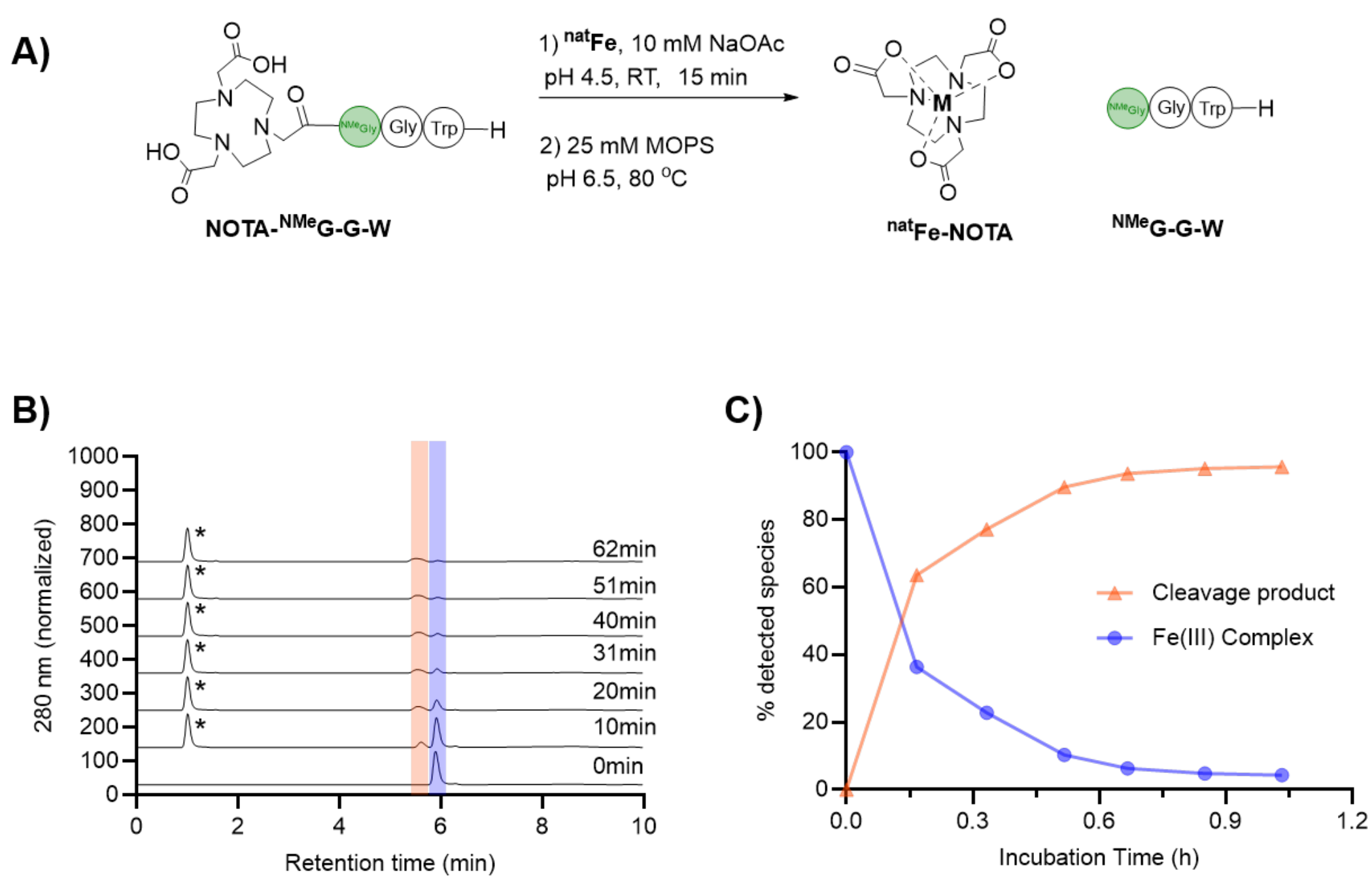

Figure S69. (A) Synthesis scheme and conditions for cleavage of **[natFe(2)]**$^{+}$. **(B)** Analytical HPLC chromatograms (Method C) show the autolytic release of the natFe-NOTA (*). **(C)** Quantification of the pH-dependent cleavage at 80 ºC and pH 6.5.

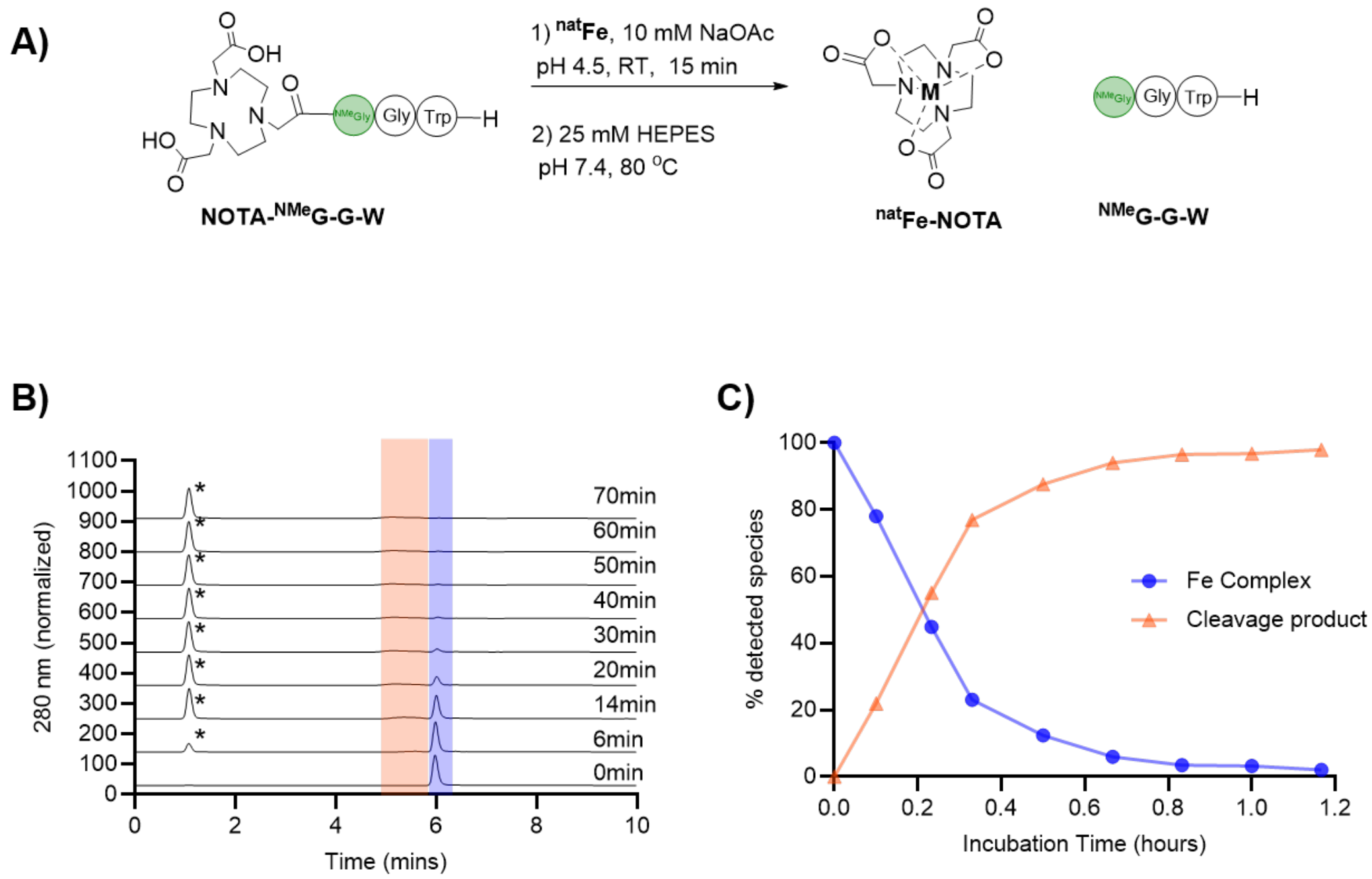

Figure S70. (A) Synthesis scheme and conditions for cleavage of **[natFe(2)]**$^+$. **(B)** Analytical HPLC chromatograms (Method C) show the autolytic release of the natFe-NOTA (*). **(C)** Quantification of the pH-dependent cleavage at 80 ºC and pH 7.4.

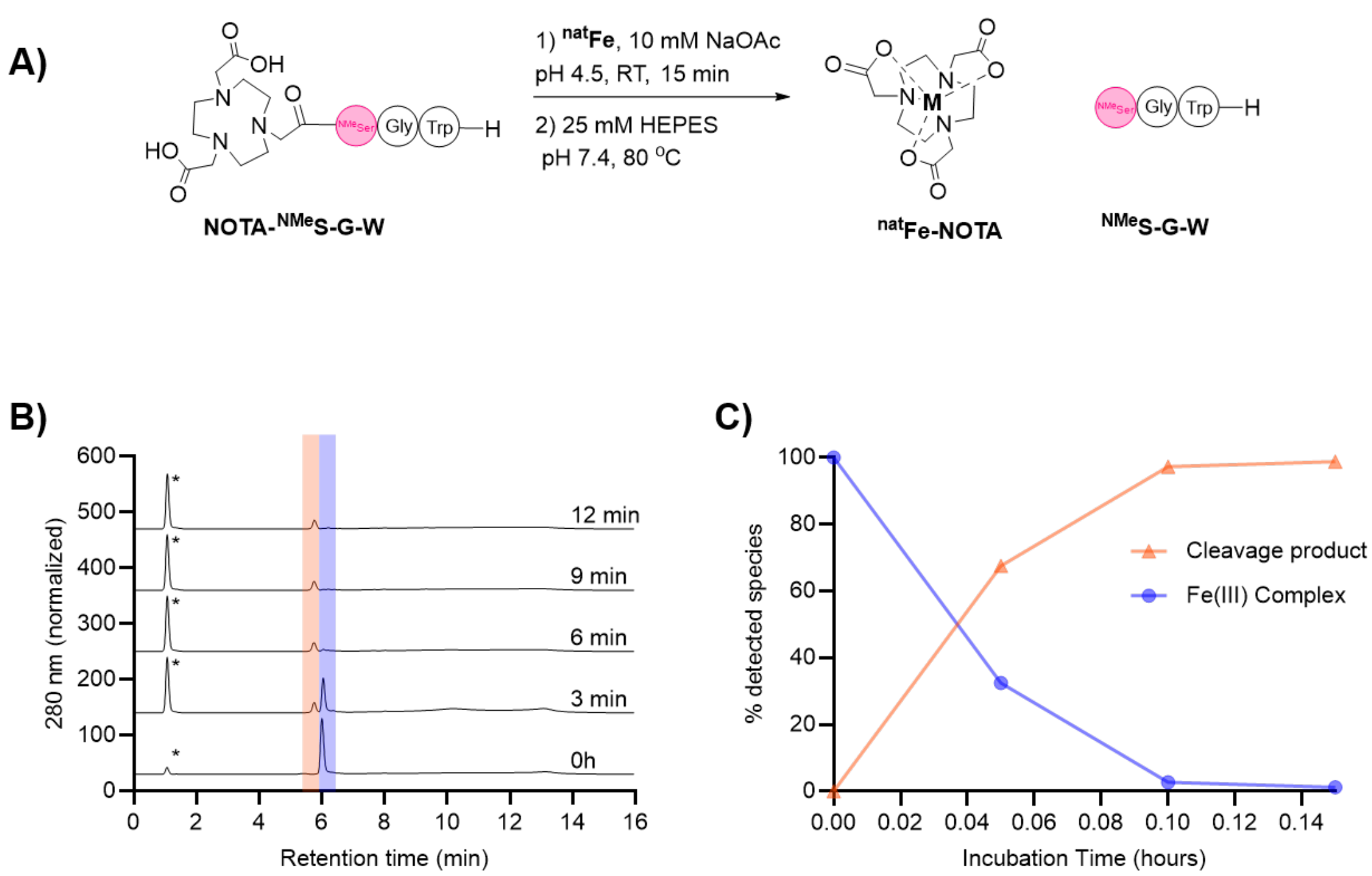

Figure S71. (A) Synthesis scheme and conditions for cleavage of **[natFe(3)]**$^+$. **(B)** Analytical HPLC chromatograms (Method B) show the autolytic release of the natFe-NOTA (*). **(C)** Quantification of the pH-dependent cleavage at 80 ºC and pH 7.4.

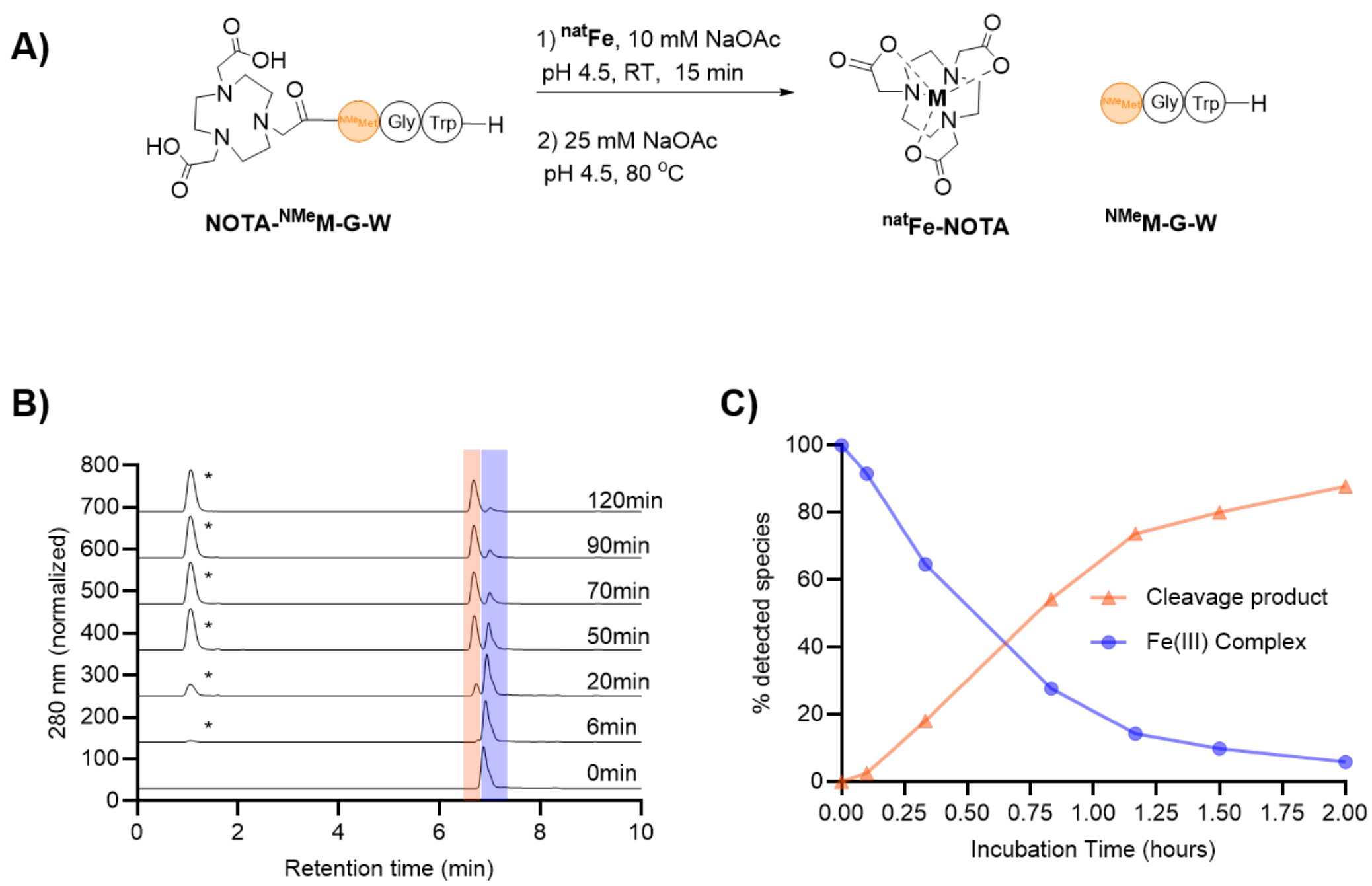

Figure S72. (A) Synthesis scheme and conditions for cleavage of **[^{nat}Fe(4)]$^{+}$**. **(B)** Analytical HPLC chromatograms (Method C) show the autolytic release of the ^{nat}Fe-NOTA (*). **(C)** Quantification of the pH-dependent cleavage at 80 ºC and pH 4.5.

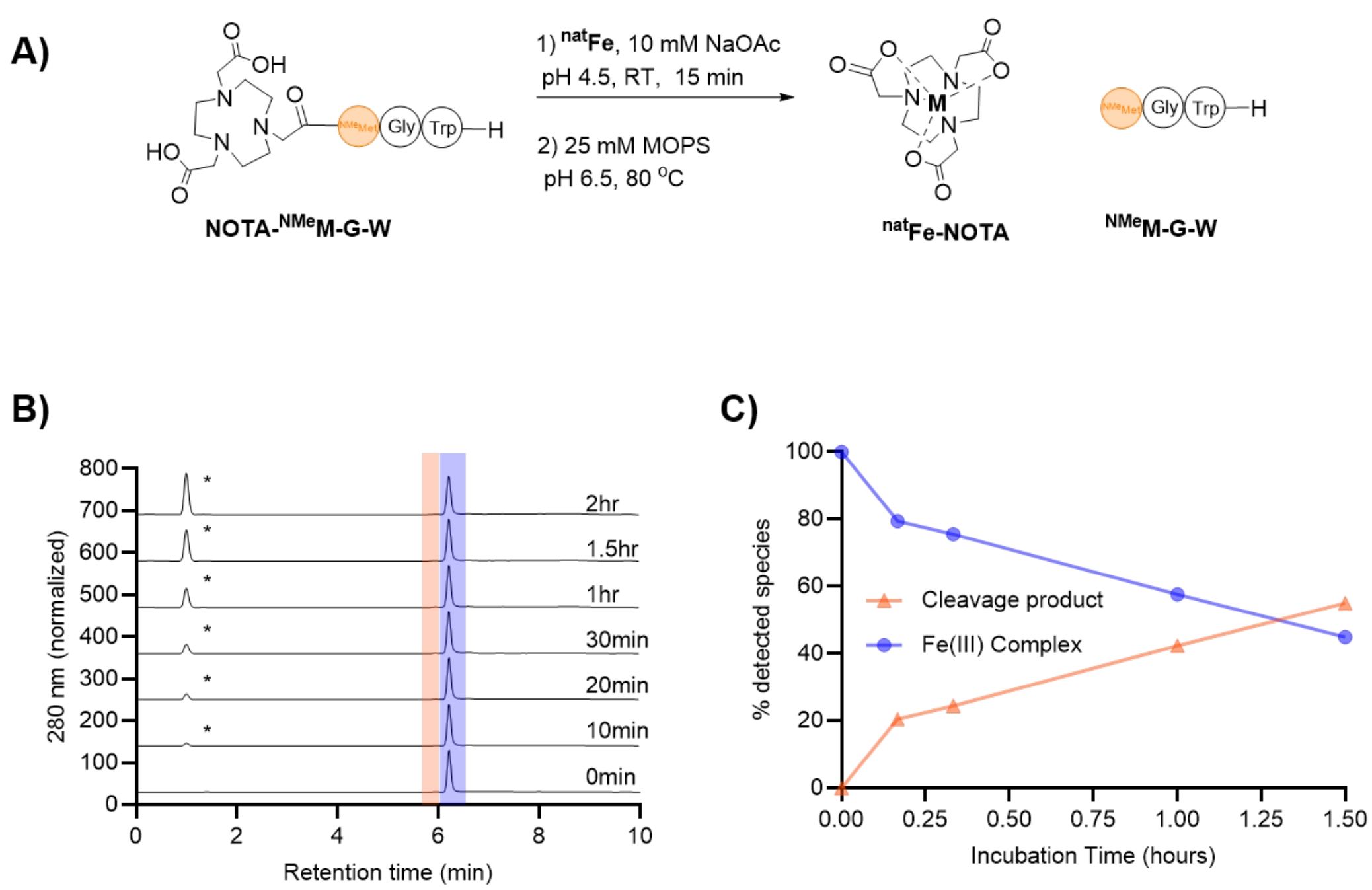

Figure S73. (A) Synthesis scheme and conditions for cleavage of **[^{nat}Fe(4)]$^{+}$**. **(B)** Analytical HPLC chromatograms (Method C) show the autolytic release of the ^{nat}Fe-NOTA (*). **(C)** Quantification of the pH-dependent cleavage at 80 ºC and pH 6.5.

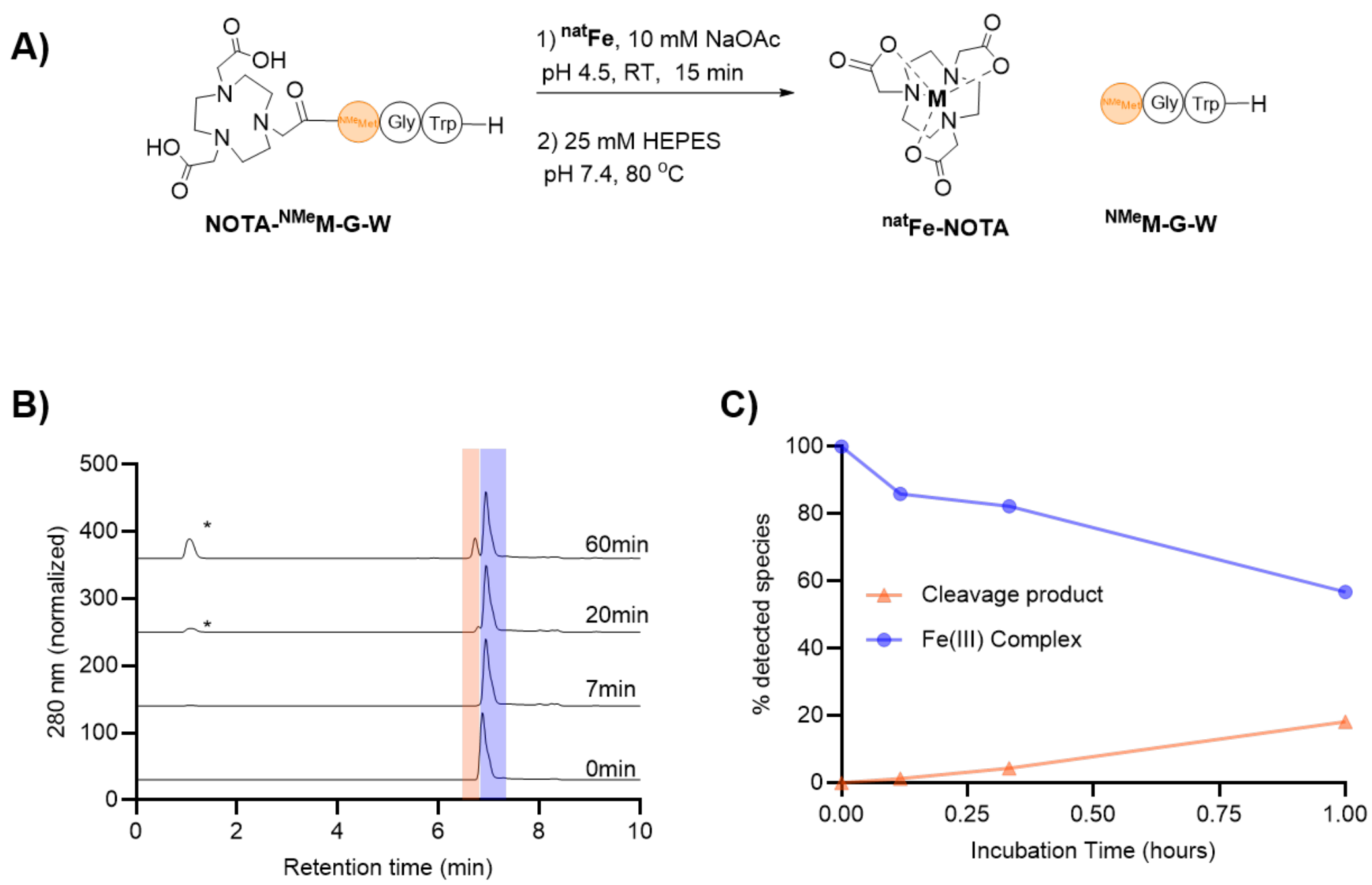

Figure S74. (A) Synthesis scheme and conditions for cleavage of **[natFe(4)]**$^{+}$. **(B)** Analytical HPLC chromatograms (Method C) show the autolytic release of the natFe-NOTA (*). **(C)** Quantification of the pH-dependent cleavage at 80 ºC and pH 7.4.

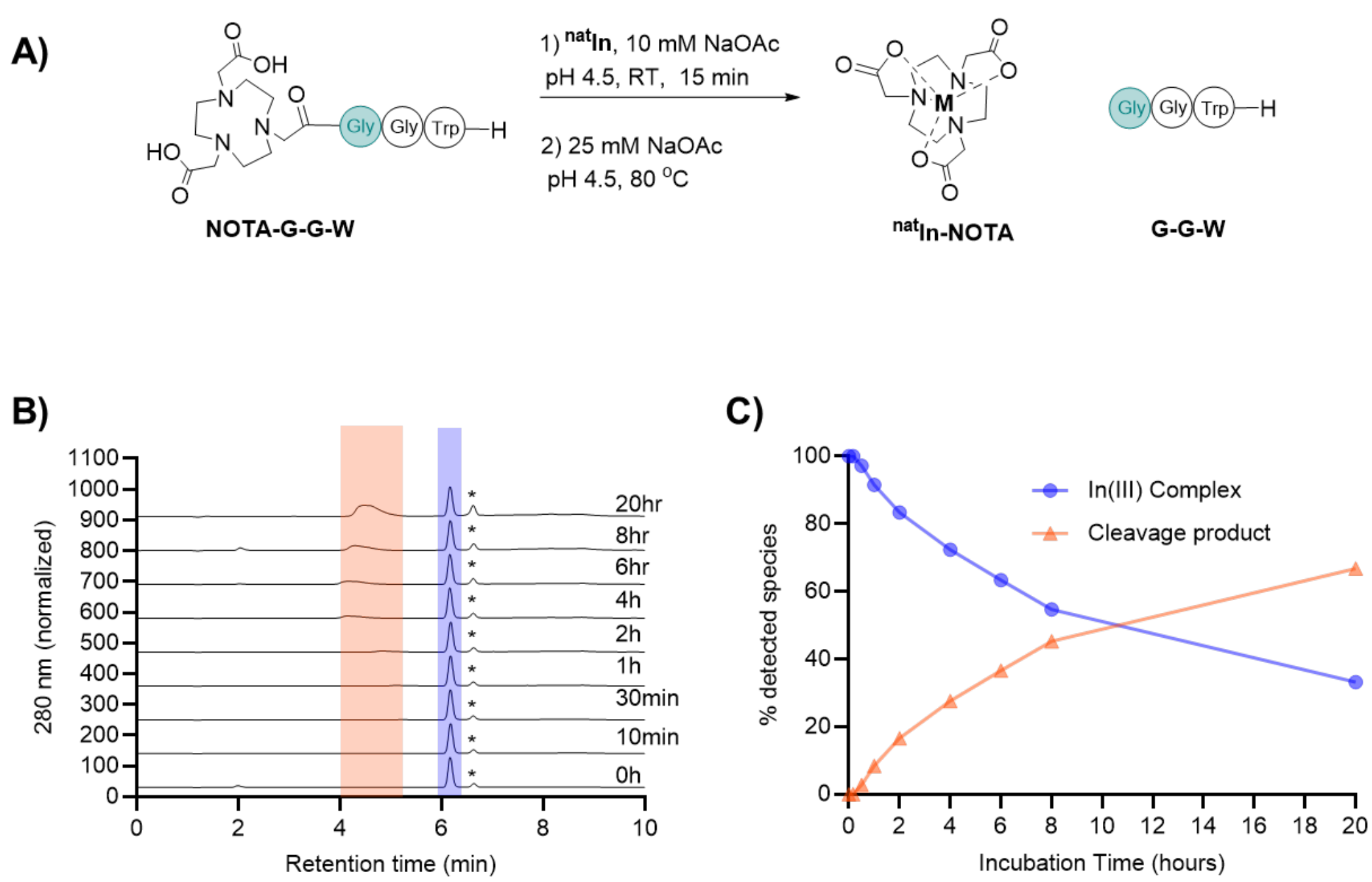

Figure S75. (A) Synthesis scheme and conditions for cleavage of **[natIn(1)]**$^{+}$. **(B)** Analytical HPLC chromatograms show the autolytic release of the natIn-NOTA. Asterisk (*) indicates the impurity from the ligand stock. **(C)** Quantification of the pH-dependent cleavage at 80 ºC and pH 4.5.

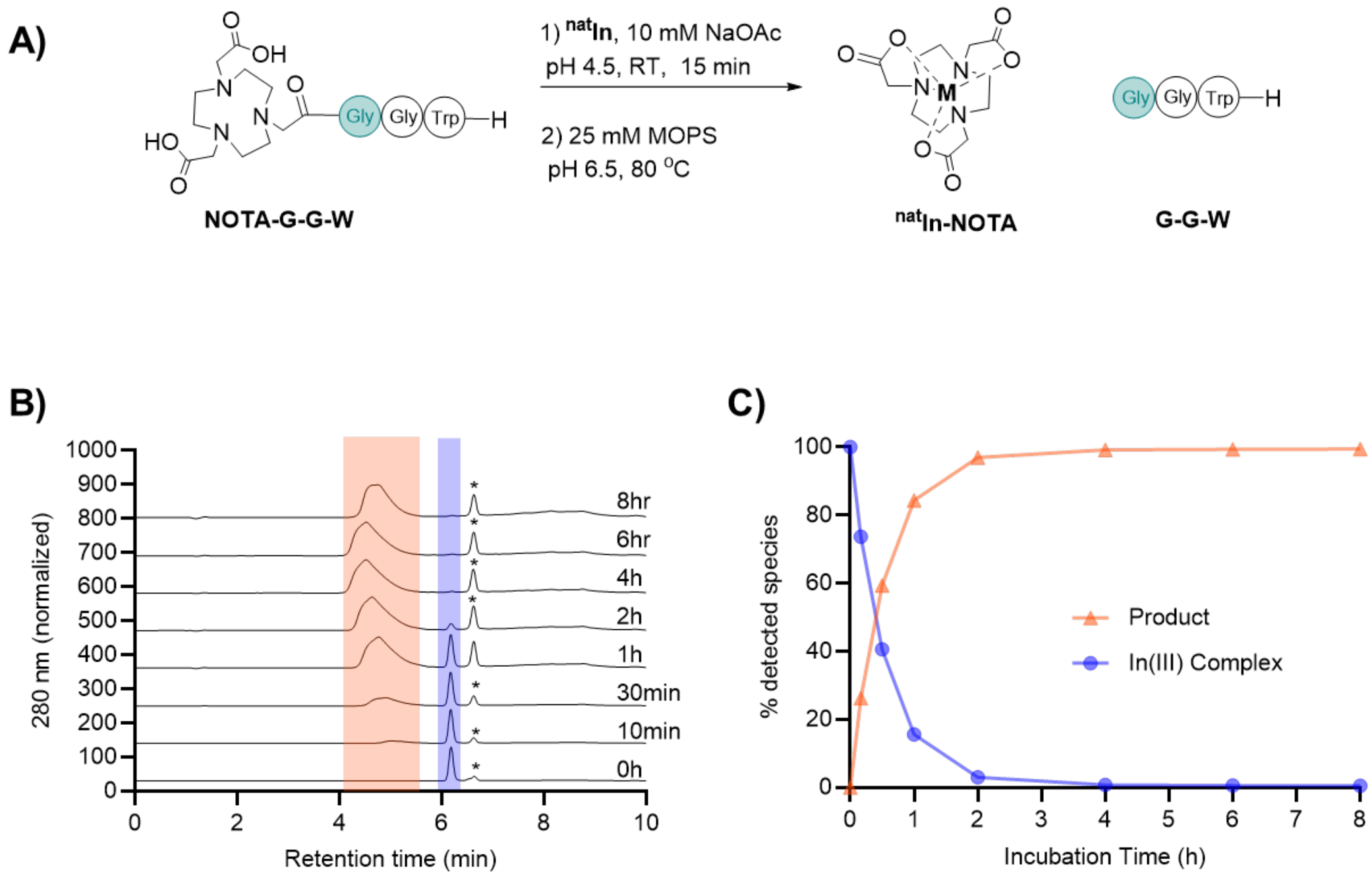

Figure S76. (A) Synthesis scheme and conditions for cleavage of **[natIn(1)]**$^+$. **(B)** Analytical HPLC chromatograms (Method C) show the autolytic release of the natIn-NOTA. Asterisk (*) indicates the impurity from the ligand stock. **(C)** Quantification of the pH-dependent cleavage at 80 °C and pH 6.5.

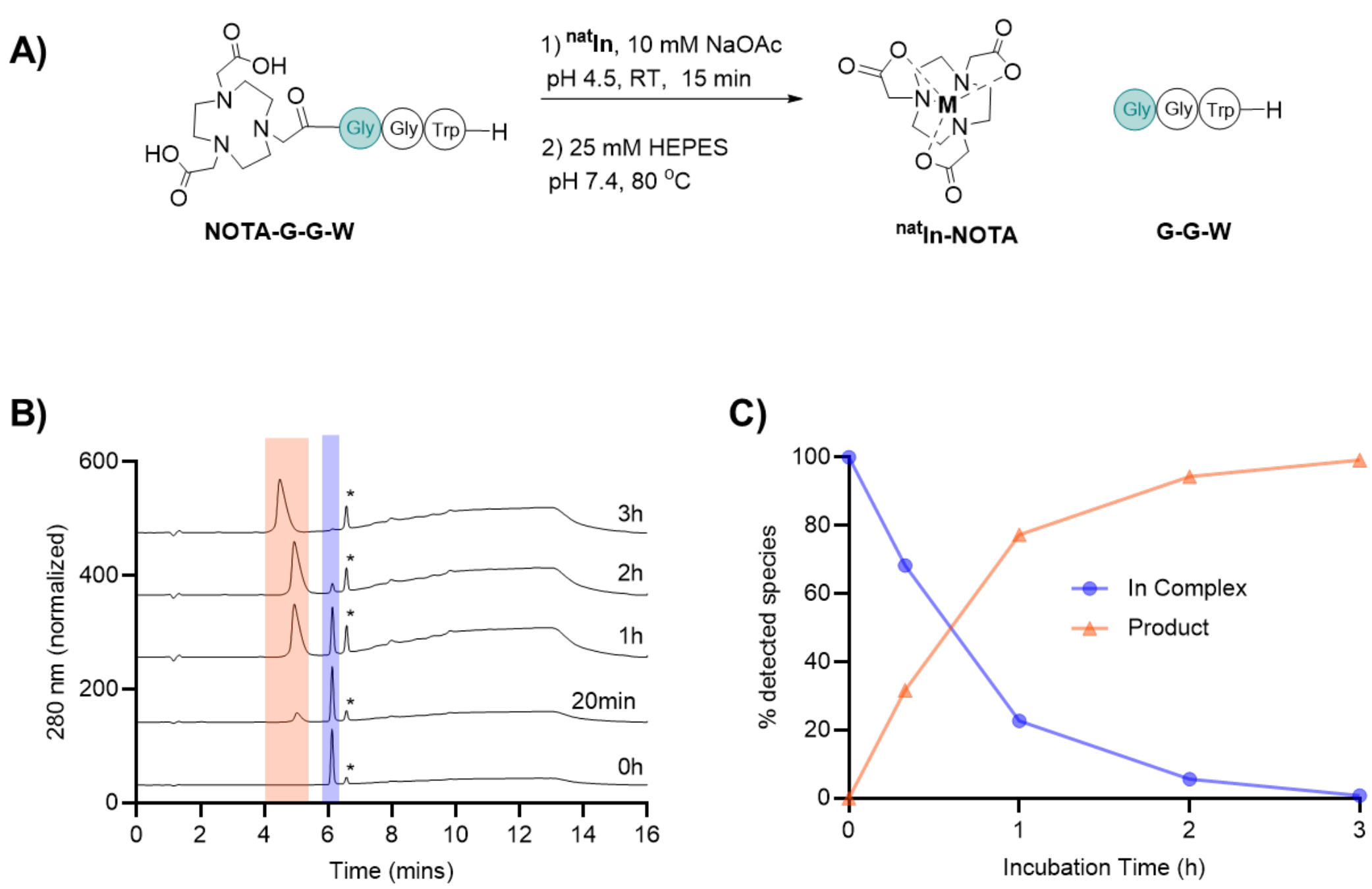

Figure S77. (A) Synthesis scheme and conditions for cleavage of **[natIn(1)]**$^+$. **(B)** Analytical HPLC chromatograms (Method B) show the autolytic release of the natIn-NOTA. Asterisk (*) indicates the impurity from the ligand stock. **(C)** Quantification of the pH-dependent cleavage at 80 °C and pH 7.4.

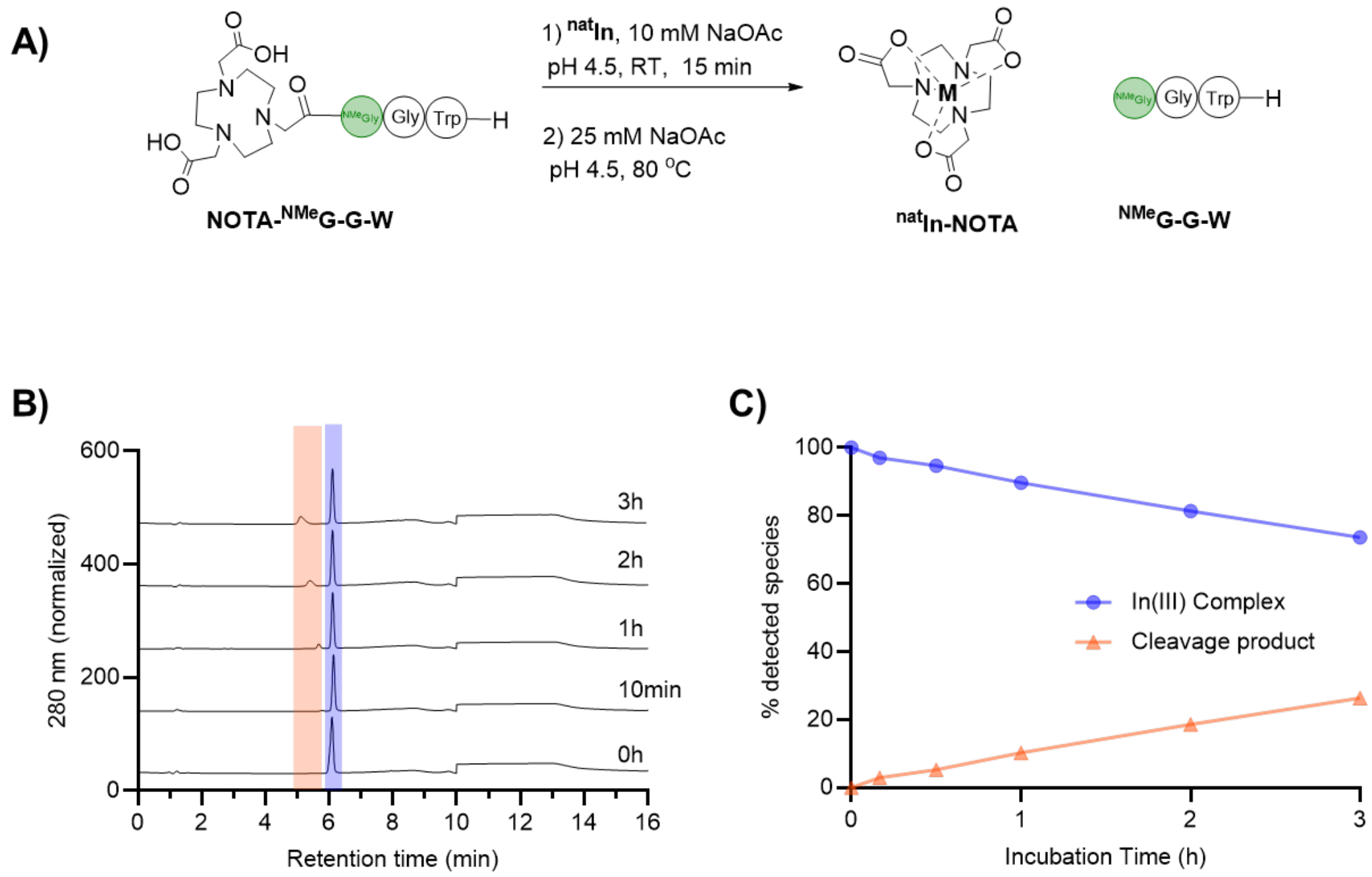

Figure S78. (A) Synthesis scheme and conditions for cleavage of **[natIn(2)]**$^{+}$. **(B)** Analytical HPLC chromatograms (Method B) show the autolytic release of the natIn-NOTA. **(C)** Quantification of the pH-dependent cleavage at 80 ºC and pH 4.5.

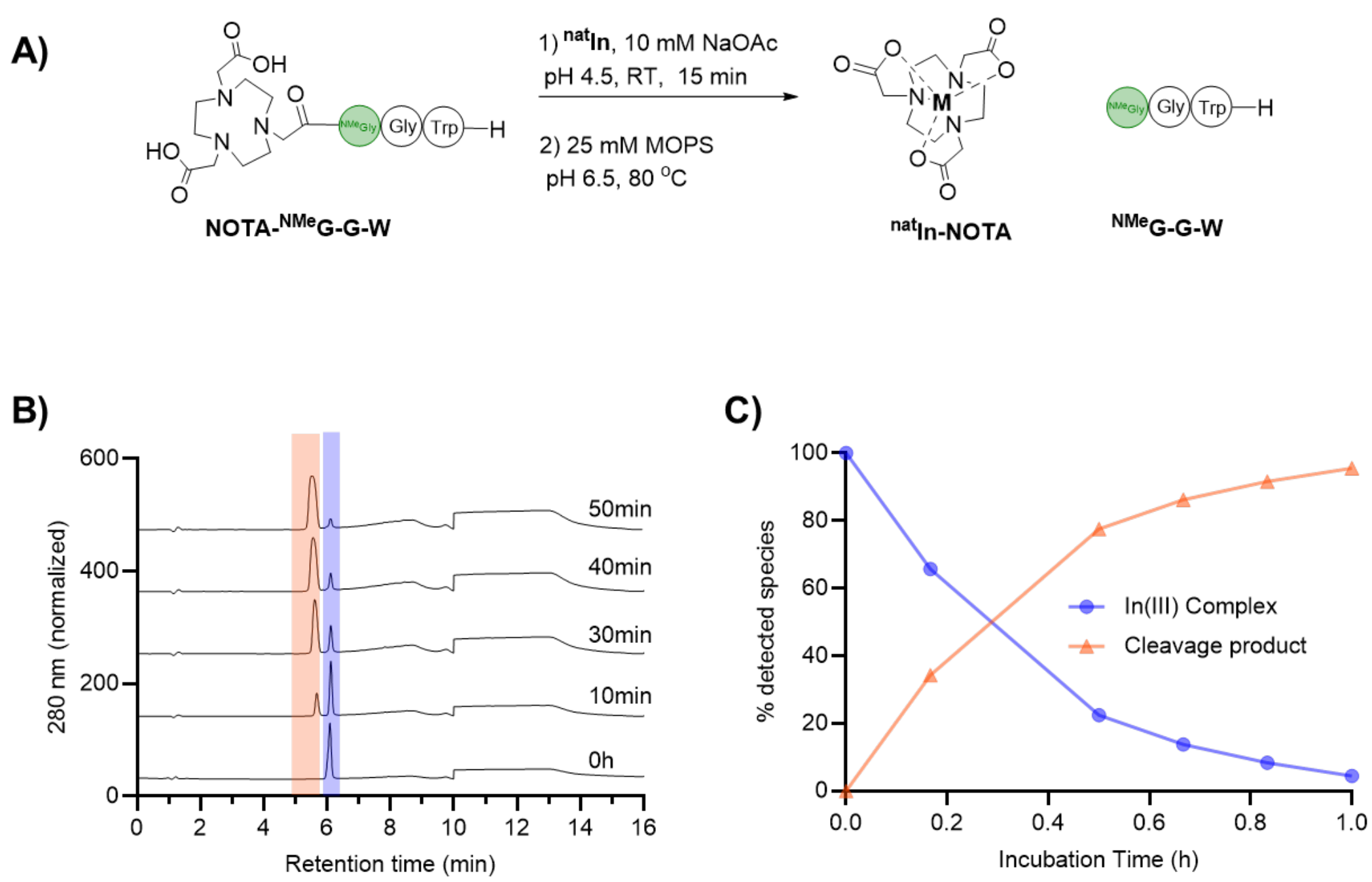

Figure S79. (A) Synthesis scheme and conditions for cleavage of **[natIn(2)]**$^{+}$. **(B)** Analytical HPLC chromatograms (Method B) show the autolytic release of the natIn-NOTA. **(C)** Quantification of the pH-dependent cleavage at 80 ºC and pH 6.5.

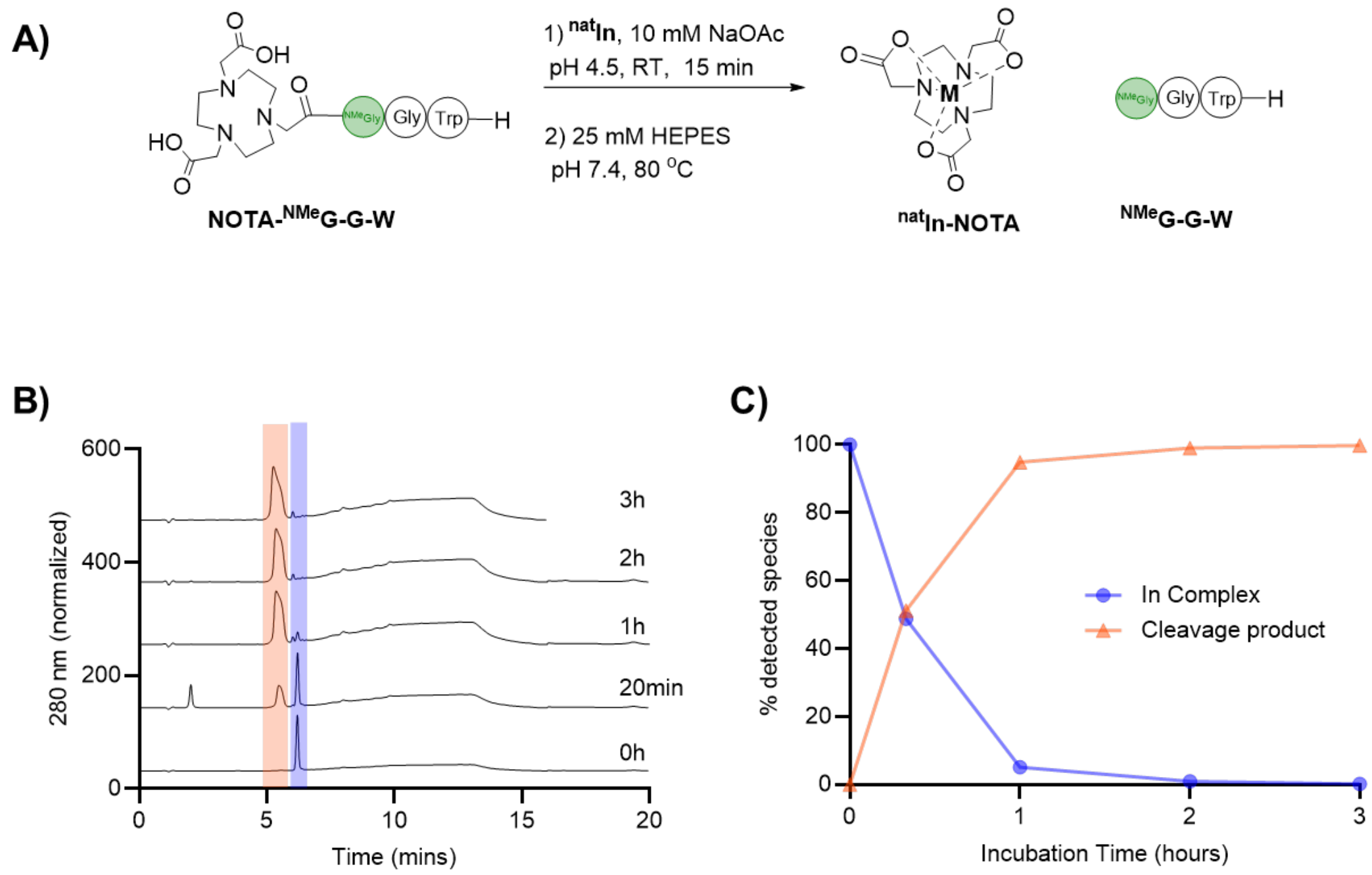

Figure S80. (A) Synthesis scheme and conditions for cleavage of **[natIn(2)]$^{+}$**. **(B)** Analytical HPLC chromatograms (Method A) show the autolytic release of the natIn-NOTA. **(C)** Quantification of the pH-dependent cleavage at 80 ºC and pH 7.4.

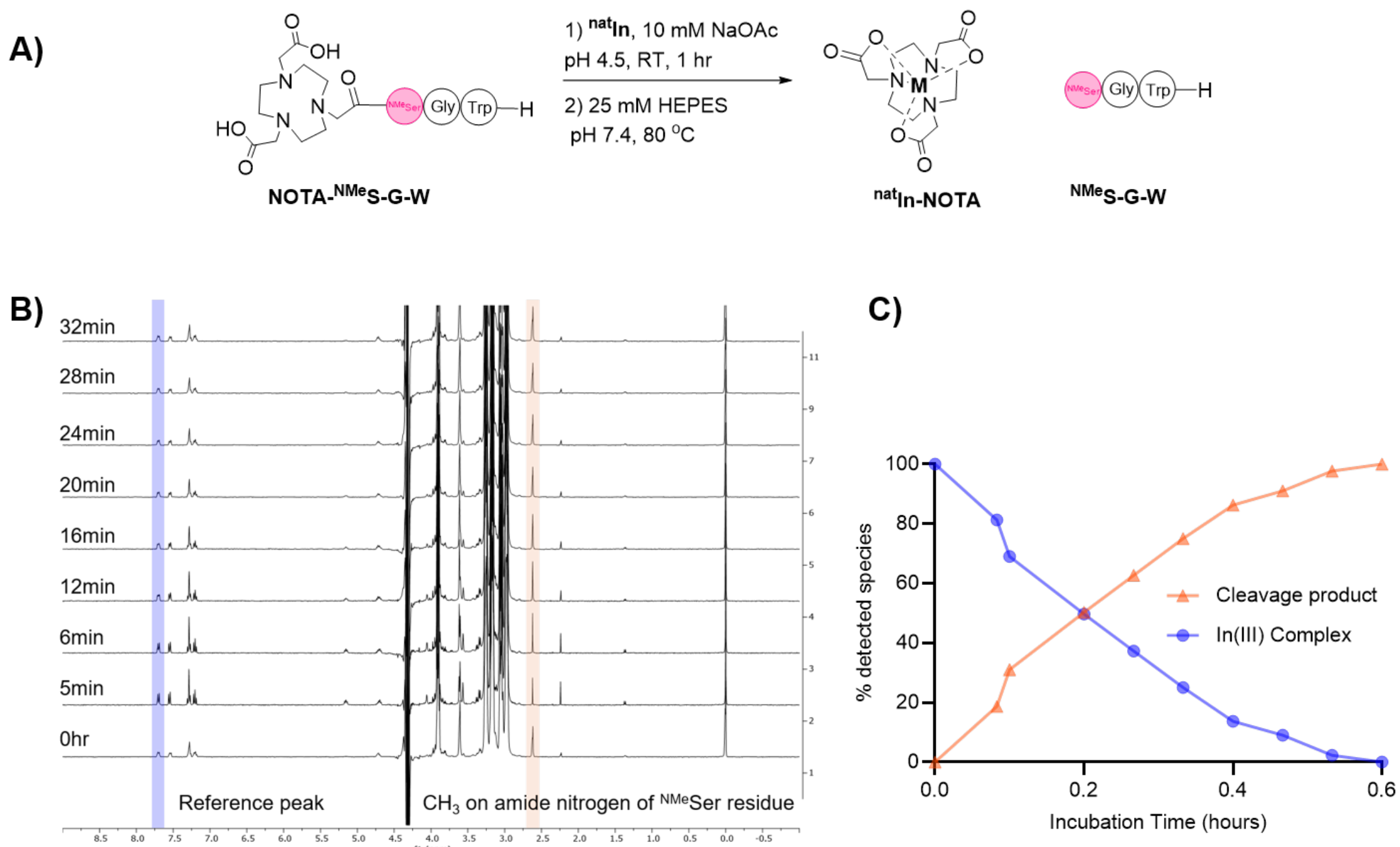

Figure S81. (A) Synthesis scheme and conditions for cleavage of **[natIn(3)]$^{+}$**. **(B)** Stacked VT-NMR spectra show the autolytic release of natIn-NOTA (VT NMR). **(C)** Quantification of the pH-dependent cleavage at 80 ºC and pH 7.4.

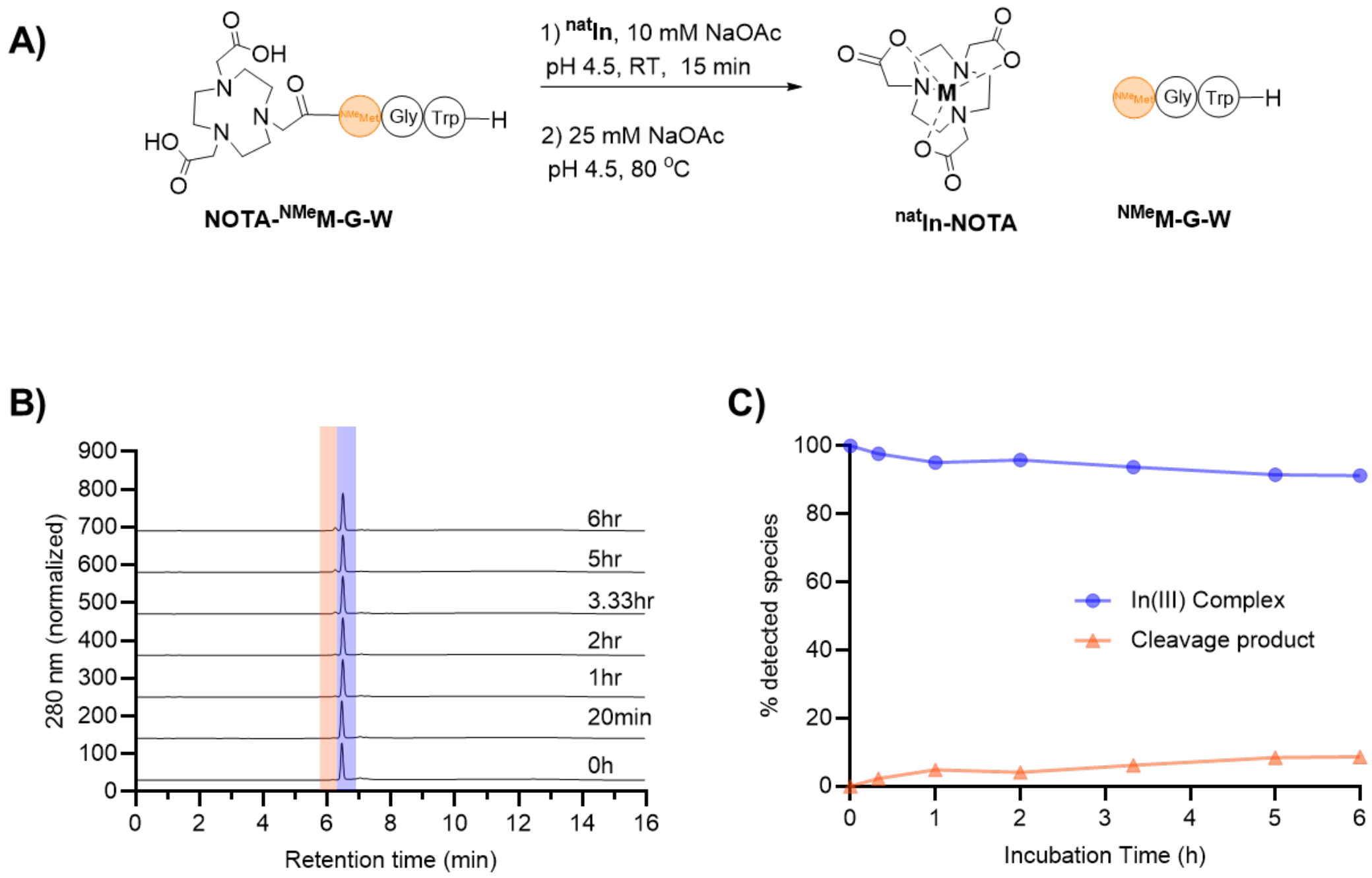

Figure S82. (A) Synthesis scheme and conditions for cleavage of **[^{nat}In(4)]$^{+}$**. **(B)** Analytical HPLC chromatograms (Method B) show the autolytic release of the ^{nat}In-NOTA. **(C)** Quantification of the pH-dependent cleavage at 80 °C and pH 4.5.

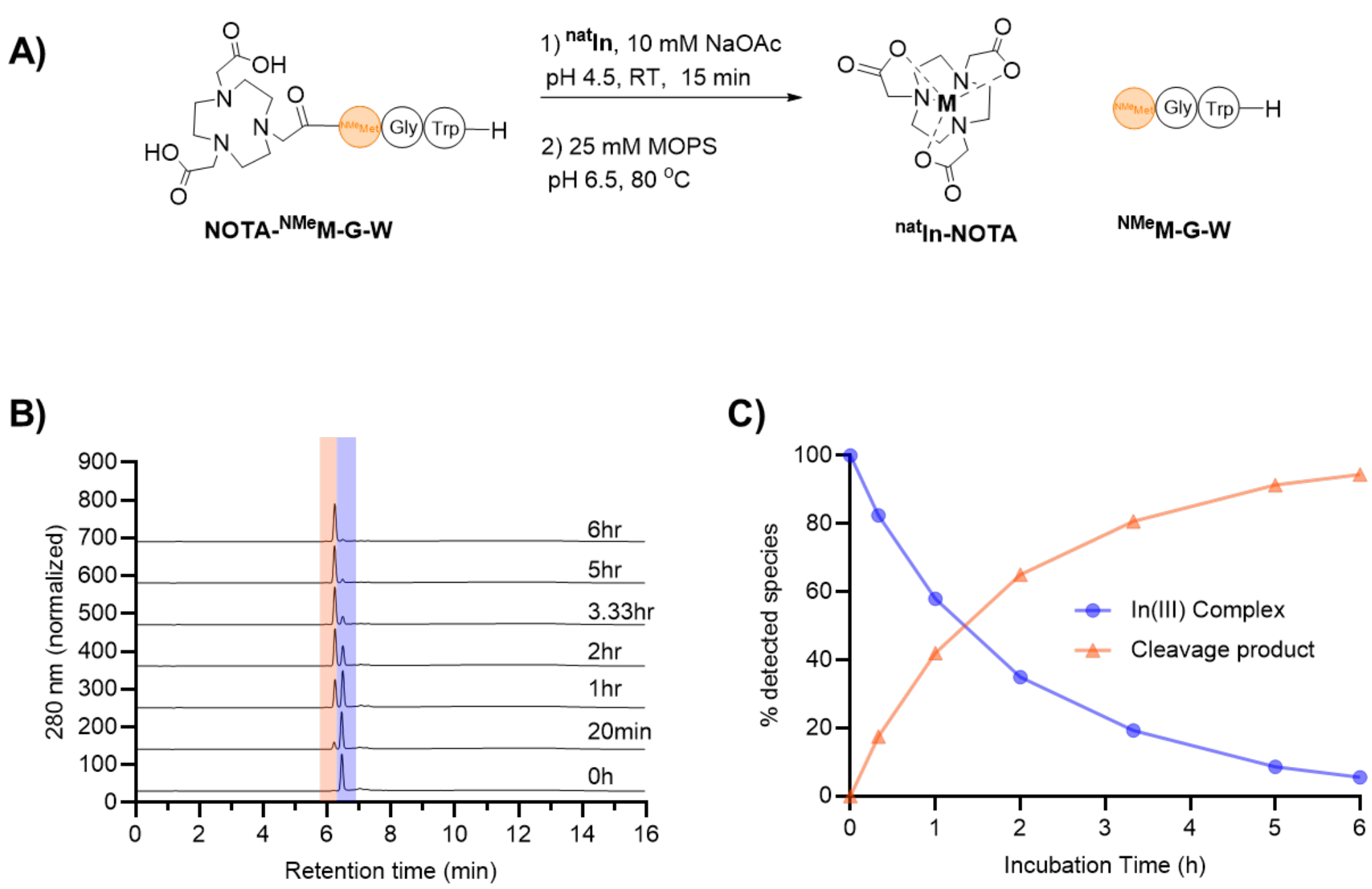

Figure S83. (A) Synthesis scheme and conditions for cleavage of **[^{nat}In(4)]$^{+}$**. **(B)** Analytical HPLC chromatograms (Method B) show the autolytic release of the ^{nat}In-NOTA. **(C)** Quantification of the pH-dependent cleavage at 80 °C and pH 6.5.

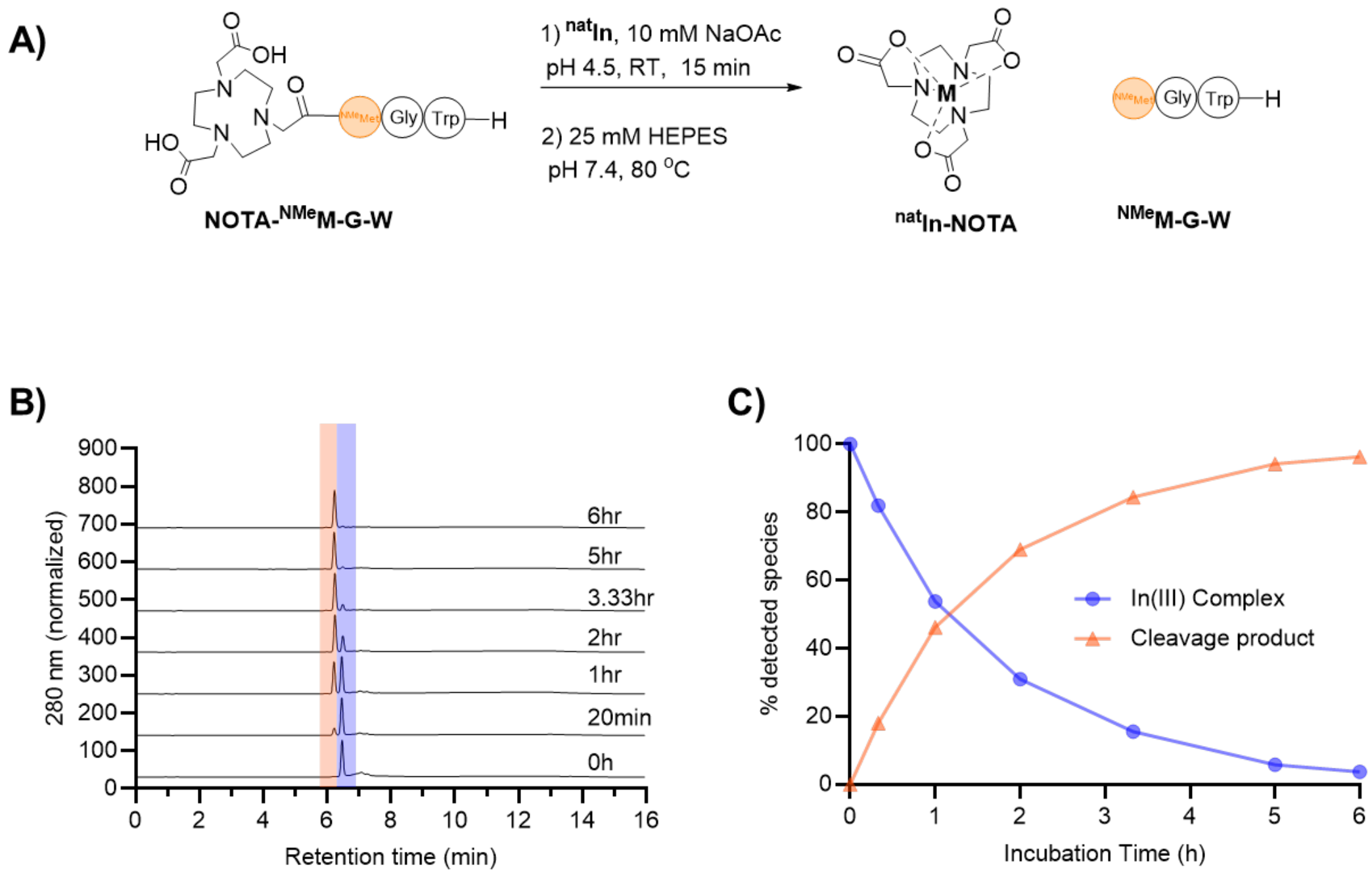

Figure S84. (A) Synthesis scheme and conditions for cleavage of **[natIn(4)]**$^+$. **(B)** Analytical HPLC chromatograms (Method B) show the autolytic release of the natIn-NOTA. **(C)** Quantification of the pH-dependent cleavage at 80 °C and pH 7.4.

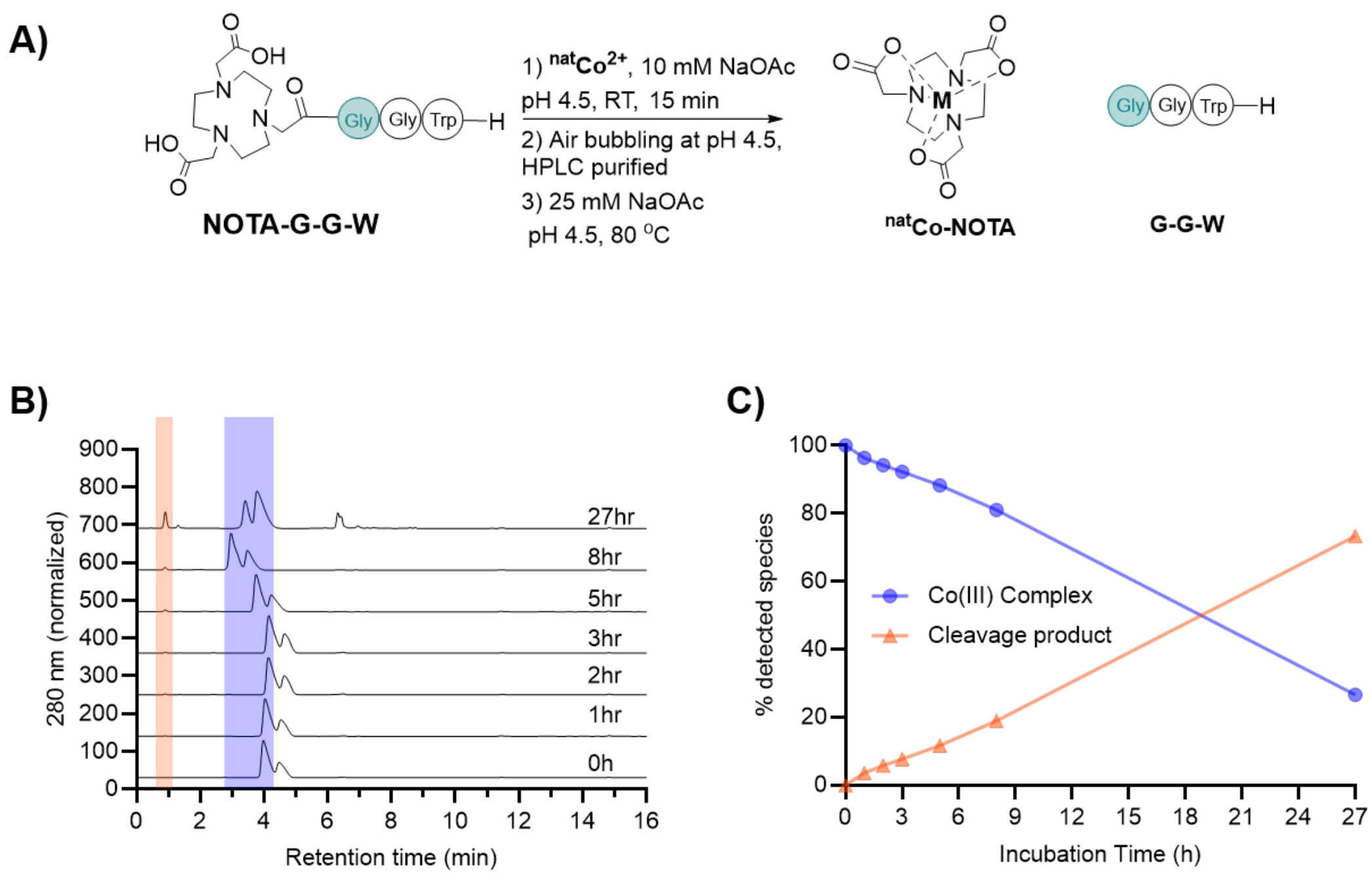

Figure S85. (A) Synthesis scheme and conditions for cleavage of **[natCo(1)]**$^+$. The Co^{3+} complex was formed at pH 4.5. **(B)** Analytical HPLC chromatograms (Method B) show the autolytic release of the natCo-NOTA. **(C)** Quantification of the pH-dependent cleavage at 80 °C and pH 4.5.

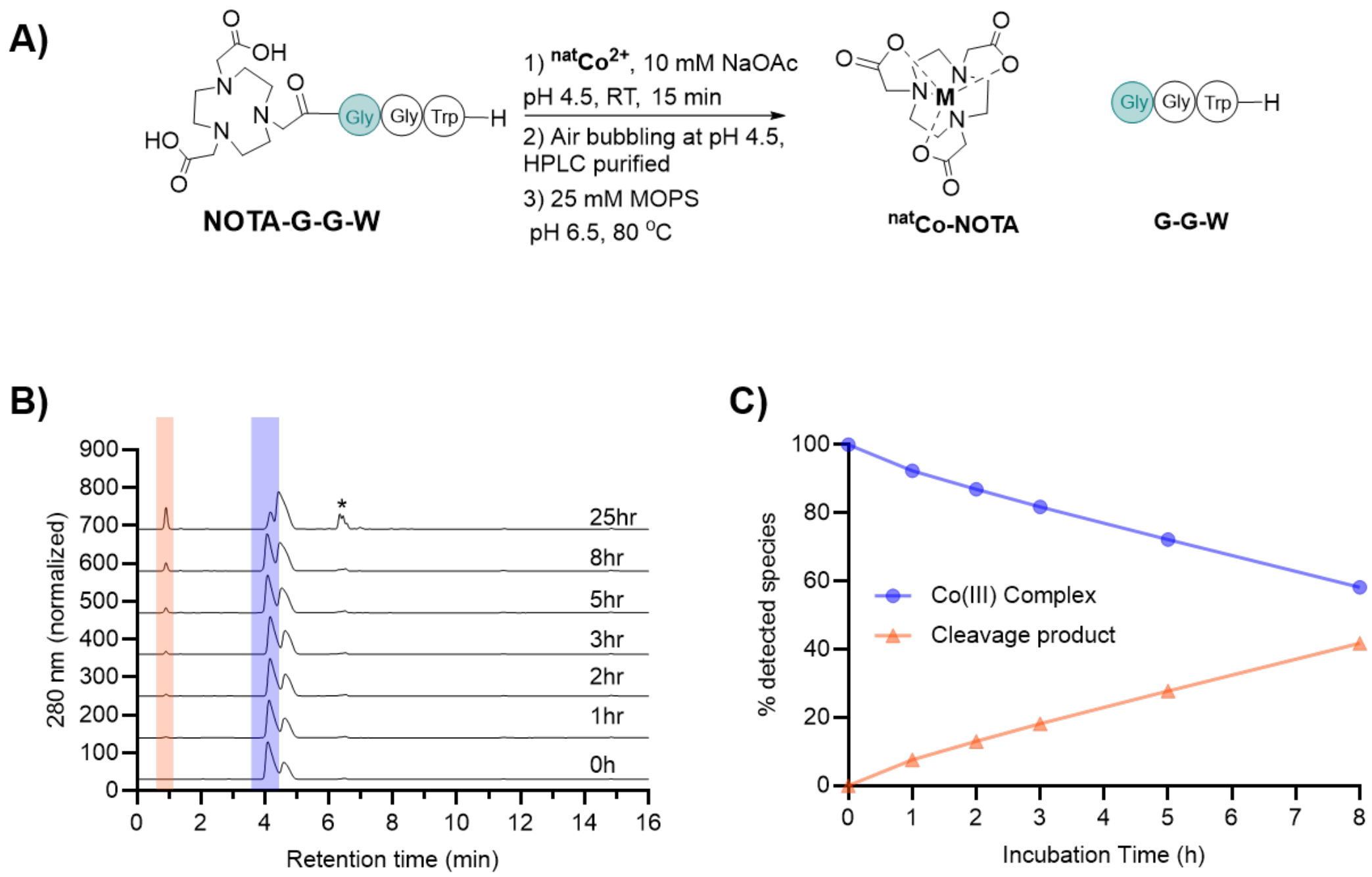

Figure S86. (A) Synthesis scheme and conditions for cleavage of **[^{nat}Co(1)]**$^{+}$. The Co^{3+} complex was formed at pH 4.5. **(B)** Analytical HPLC chromatograms (Method B) show the autolytic release of the ^{nat}Co-NOTA. Asterisk (*) indicates impurity. **(C)** Quantification of the pH-dependent cleavage at 80 °C and pH 6.5.

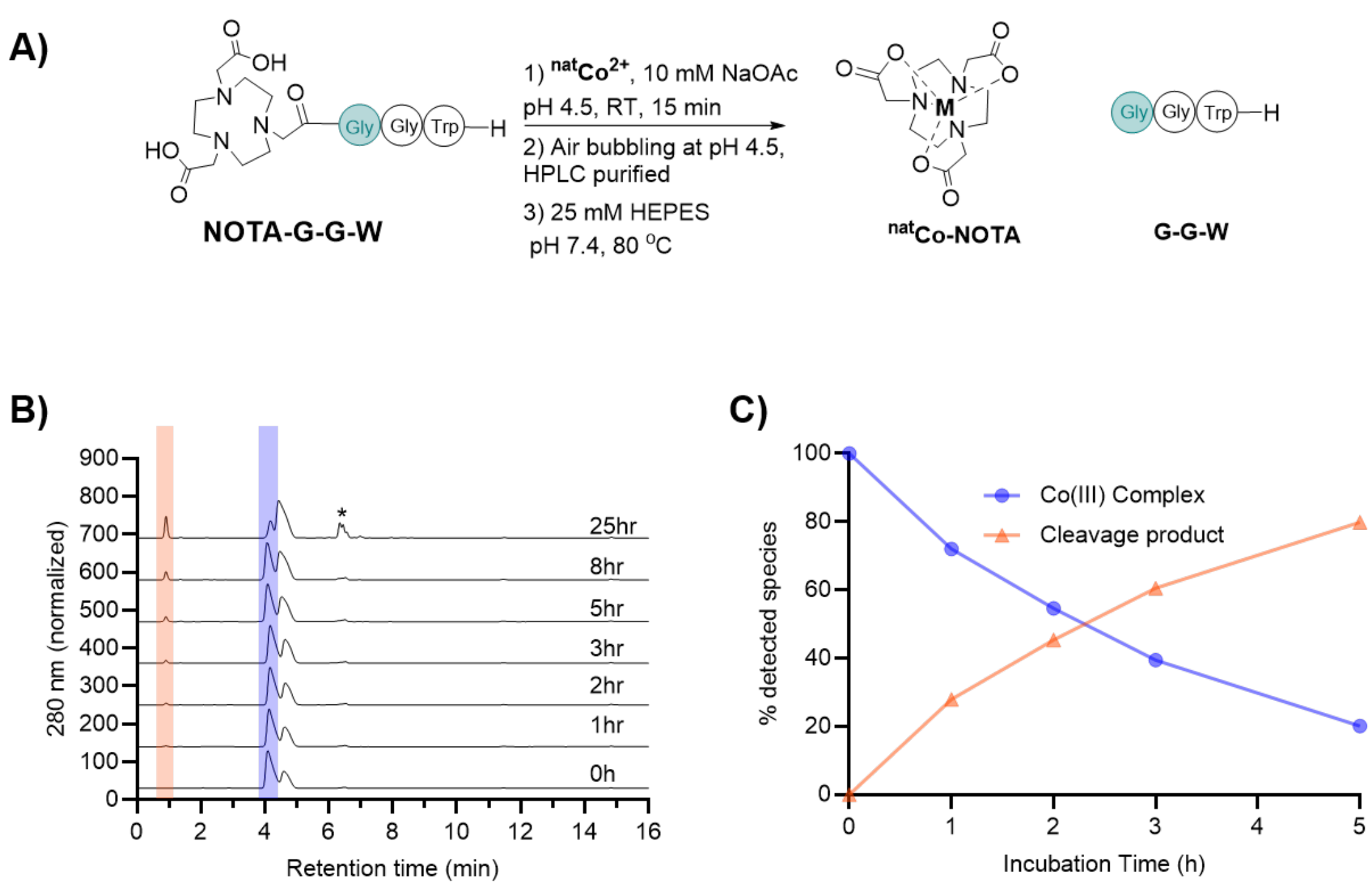

Figure S87. (A) Synthesis scheme and conditions for cleavage of **[^{nat}Co(1)]**$^{+}$. The Co^{3+} complex was formed at pH 4.5. **(B)** Analytical HPLC chromatograms (Method B) show the autolytic release of the ^{nat}Co-NOTA. Asterisk (*) indicates impurity. **(C)** Quantification of the pH-dependent cleavage at 80 °C and pH 7.4.

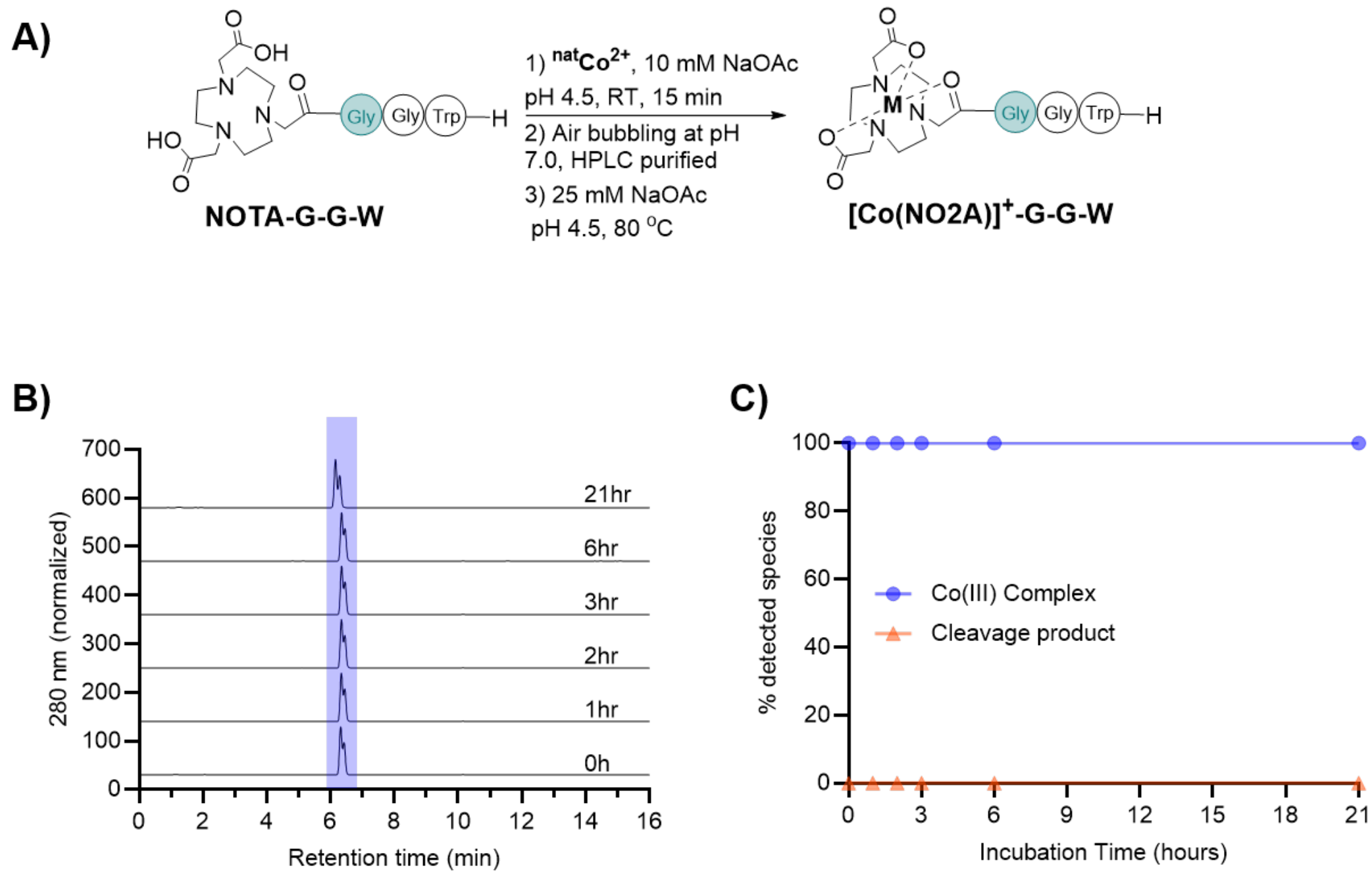

Figure S88. (A) Synthesis scheme and conditions for cleavage of **[^{nat}Co(1)]**$^{+}$. The Co^{3+} complex was formed at pH 7.0. **(B)** Analytical HPLC chromatograms (Method B) show no autolytic release of the ^{nat}Co-NOTA. **(C)** Quantification of the pH-dependent cleavage at 80 °C and pH 4.5.

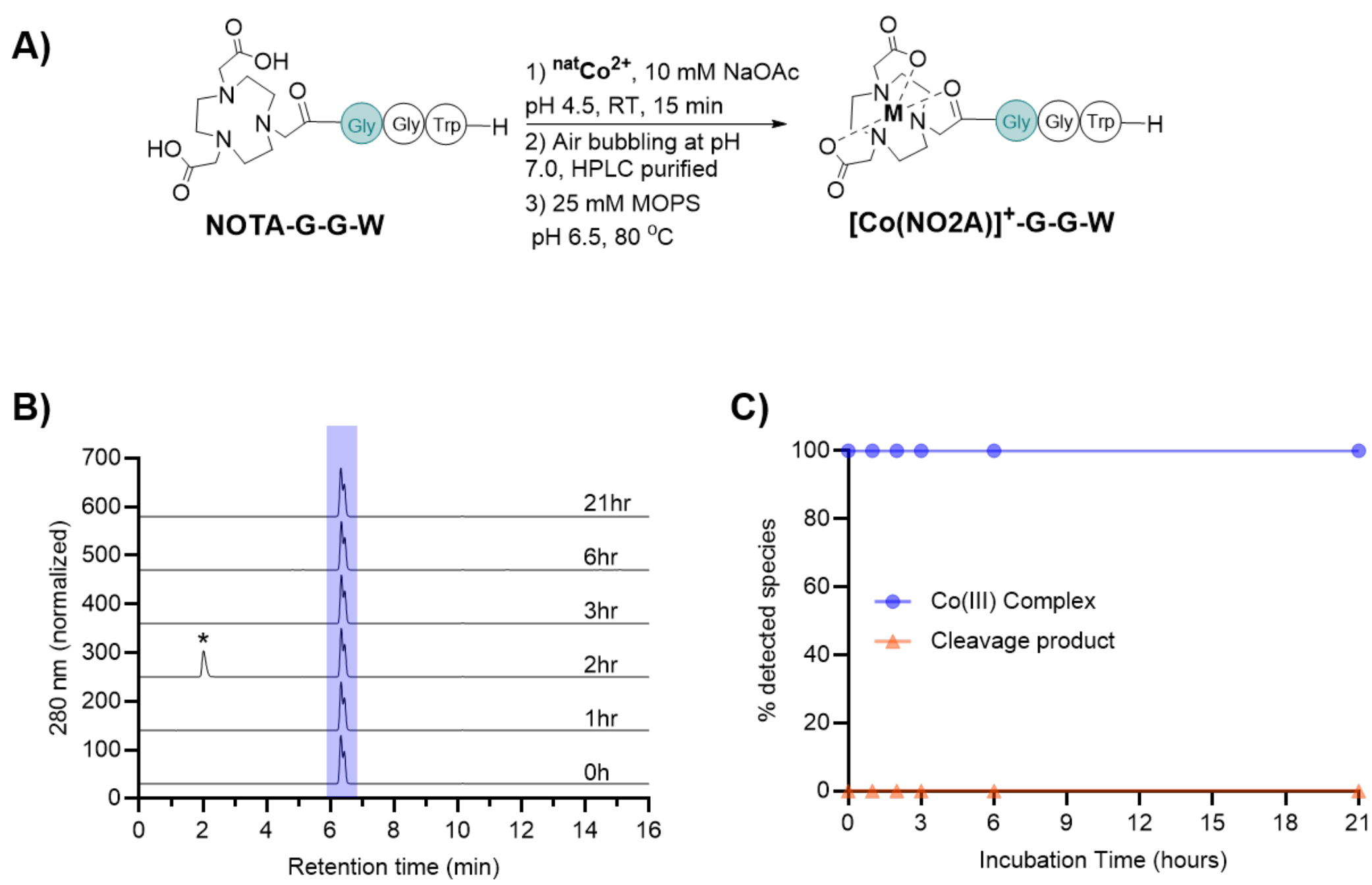

Figure S89. (A) Synthesis scheme and conditions for cleavage of **[^{nat}Co(1)]**$^{+}$. The Co^{3+} complex was formed at pH 7.0. **(B)** Analytical HPLC chromatograms (Method B) showing no autolytic release of the ^{nat}Co-NOTA. Asterisk (*) indicates impurity. **(C)** Quantification of the pH-dependent cleavage at 80 °C and pH 6.5.

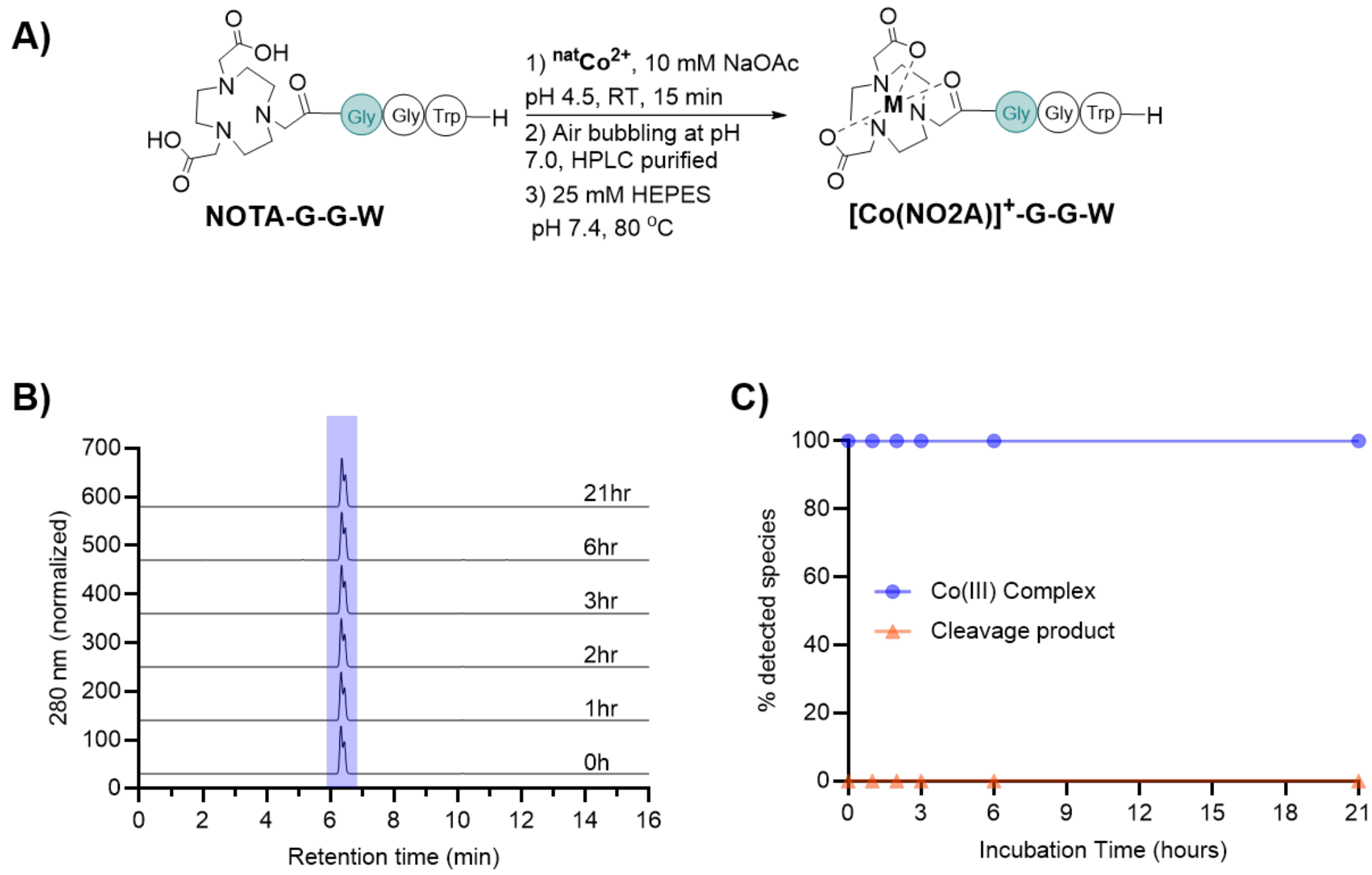

Figure S90. (A) Synthesis scheme and conditions for cleavage of **[^{nat}Co(1)]**$^{+}$. The Co^{3+} complex was formed at pH 7.0. **(B)** Analytical HPLC chromatograms (Method B) show no autolytic release of the ^{nat}Co-NOTA. **(C)** Quantification of the pH-dependent cleavage at 80 °C and pH 7.4.

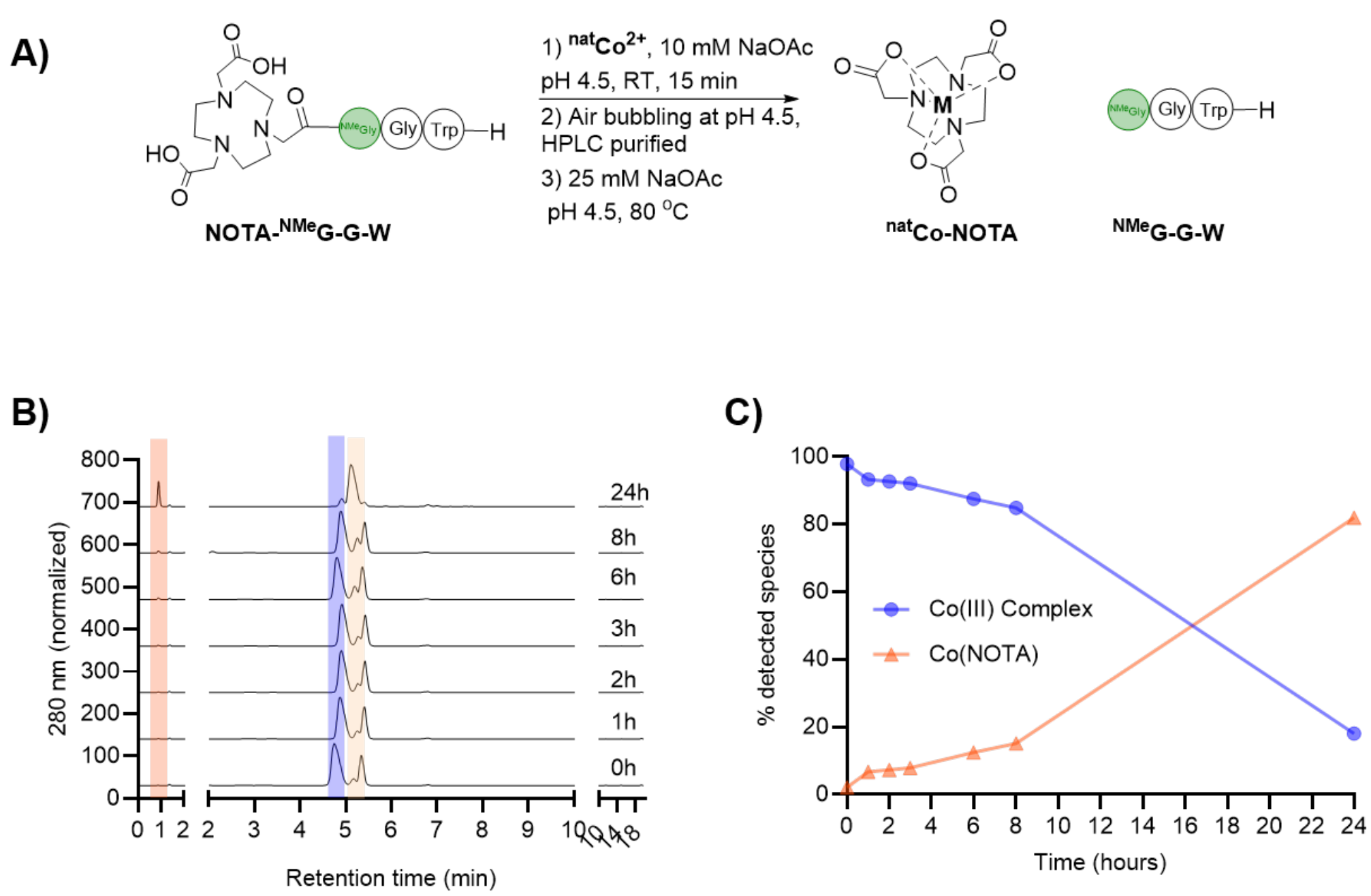

Figure S91. (A) Synthesis scheme and conditions for cleavage of **[^{nat}Co(2)]**$^{+}$. The Co^{3+} complex was formed at pH 4.5. **(B)** Analytical HPLC chromatograms (Method A) show the autolytic release of the ^{nat}Co-NOTA. **(C)** Quantification of the pH-dependent cleavage at 80 °C and pH 4.5.

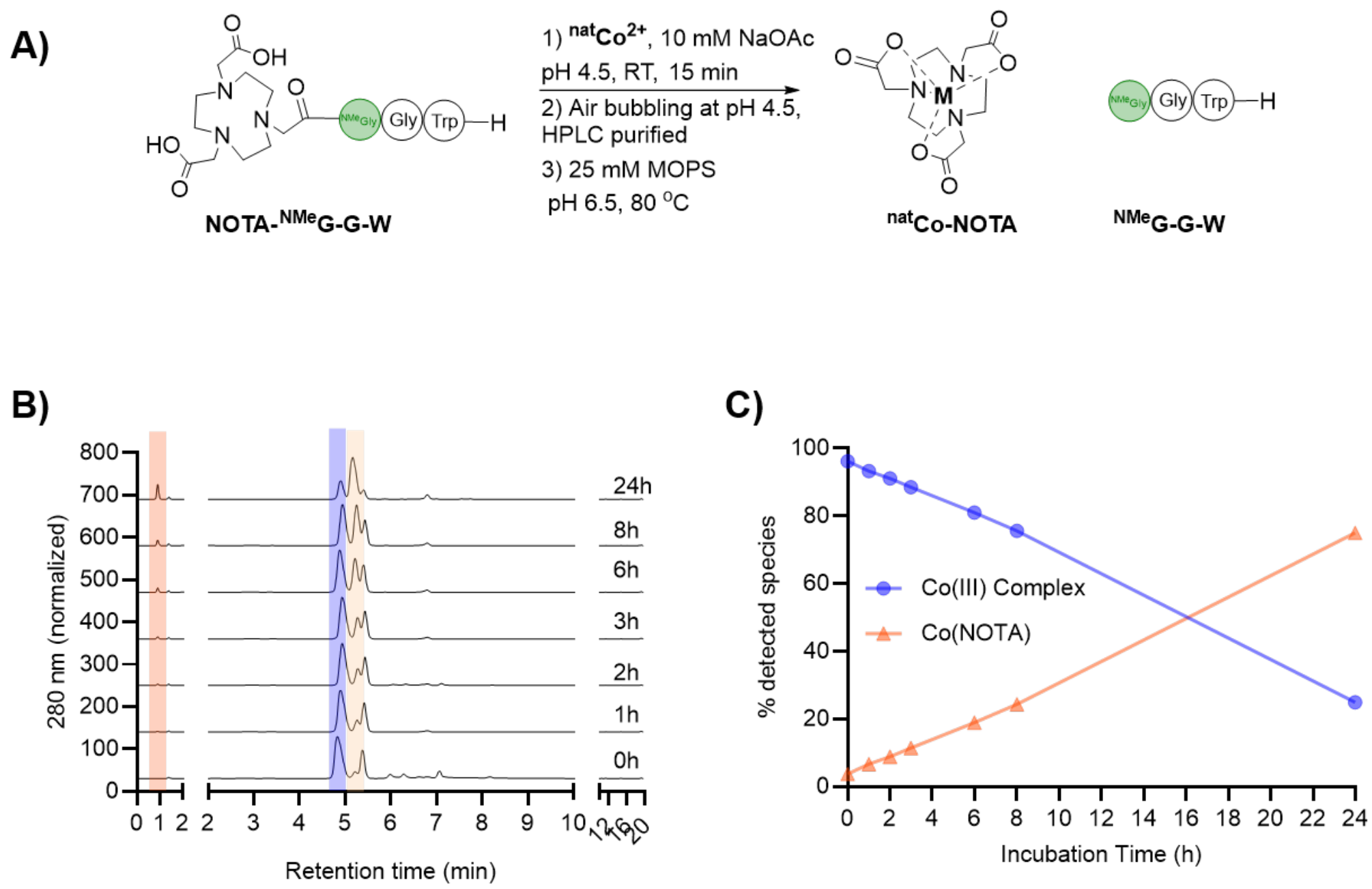

Figure S92. (A) Synthesis scheme and conditions for cleavage of **[natCo(2)]**$^{+}$.The Co^{3+} complex was formed at pH 4.5. **(B)** Analytical HPLC chromatograms (Method A) show the autolytic release of the natCo-NOTA. **(C)** Quantification of the pH-dependent cleavage at 80 °C and pH 6.5.

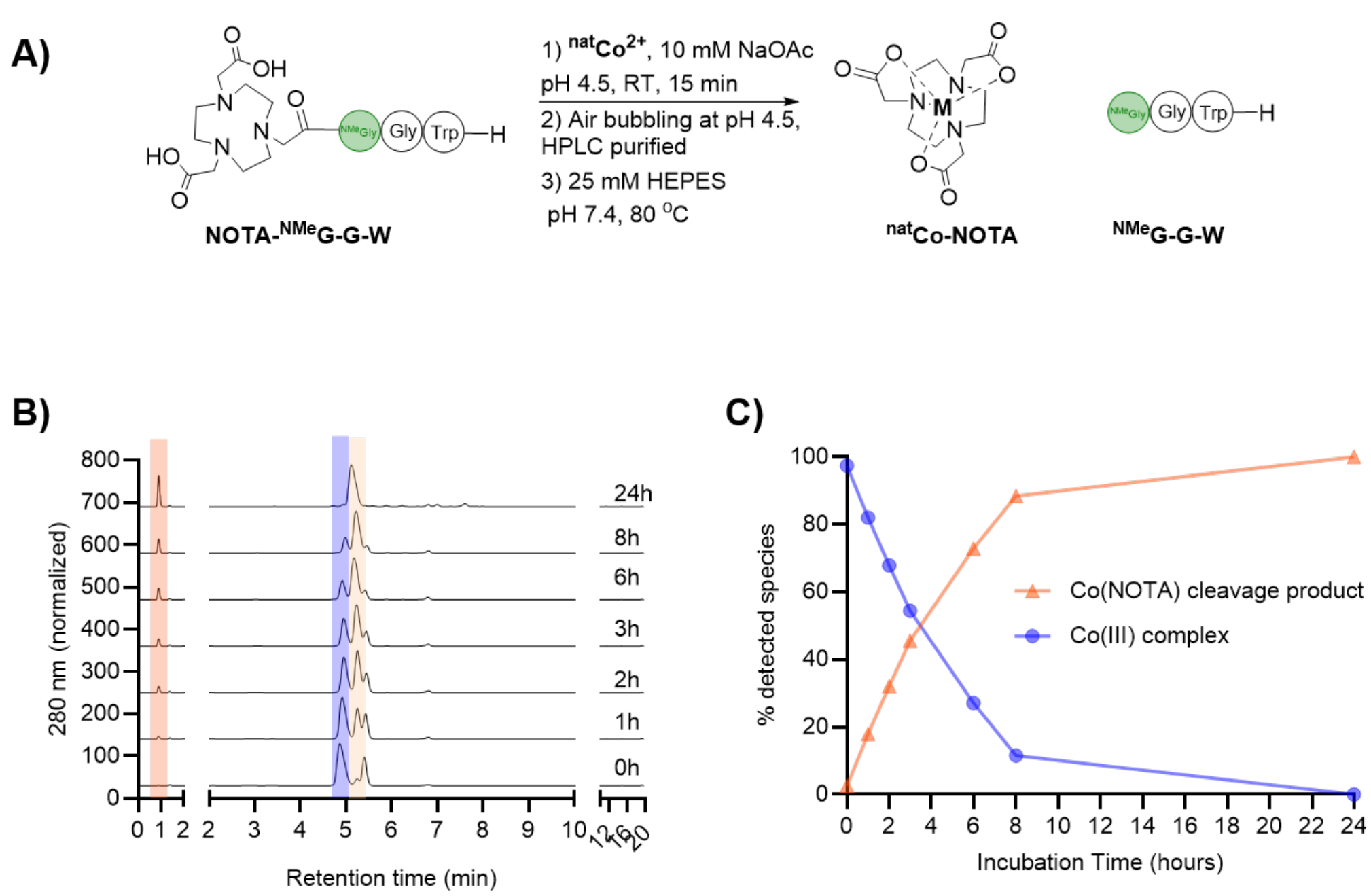

Figure S93. (A) Synthesis scheme and conditions for cleavage of **[natCo(2)]**$^{+}$. The Co^{3+} complex was formed at pH 4.5. **(B)** Analytical HPLC chromatograms (Method A) show the autolytic release of the natCo-NOTA. **(C)** Quantification of the pH-dependent cleavage at 80 °C and pH 7.4.

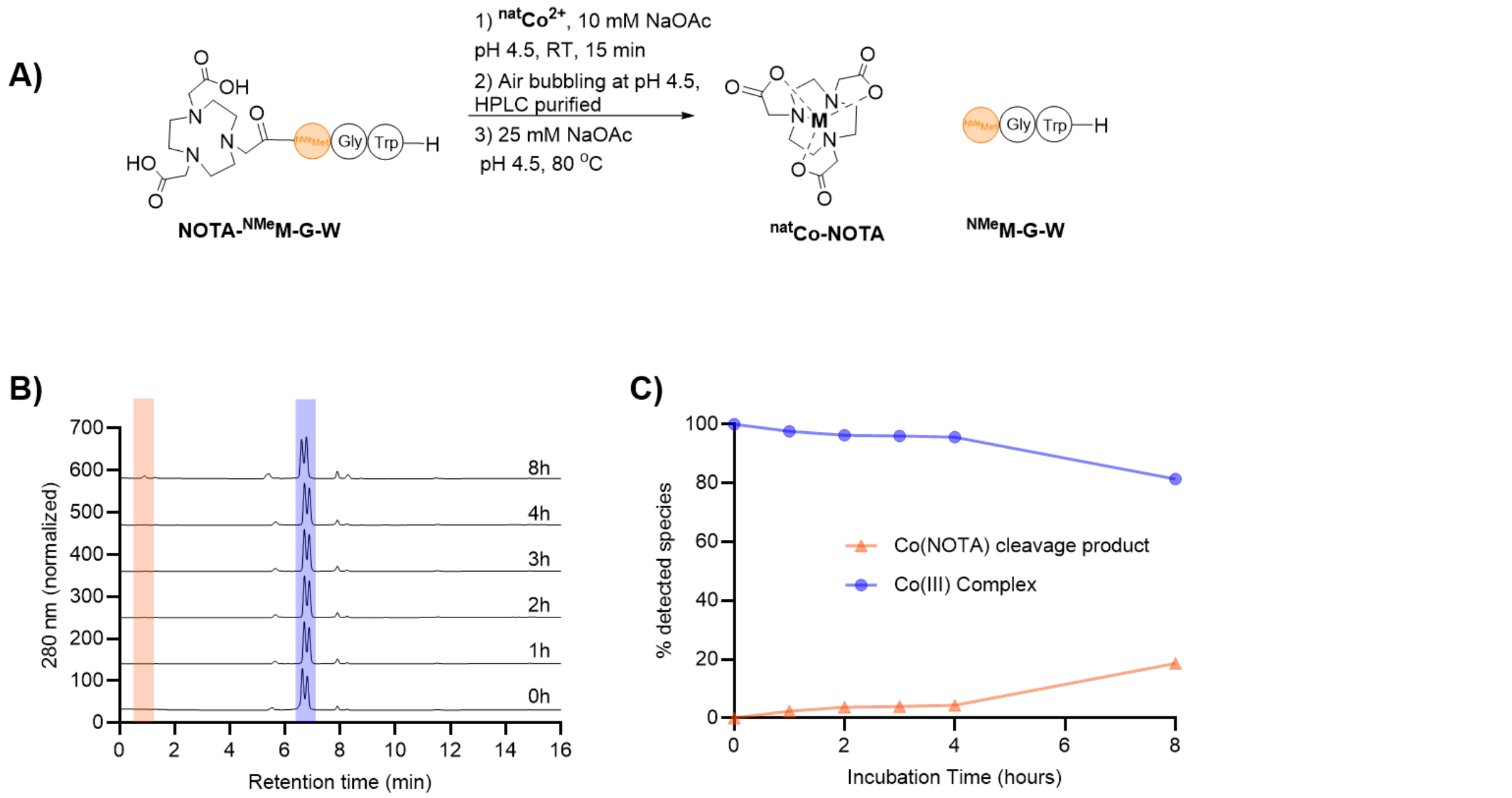

Figure S94. (A) Synthesis scheme and conditions for cleavage of **[^{nat}Co(4)]**$^{+}$. The Co^{3+} complex was formed at pH 4.5. **(B)** Analytical HPLC chromatograms (Method B) show the autolytic release of the ^{nat}Co-NOTA. **(C)** Quantification of the pH-dependent cleavage at 80 °C and pH 4.5.

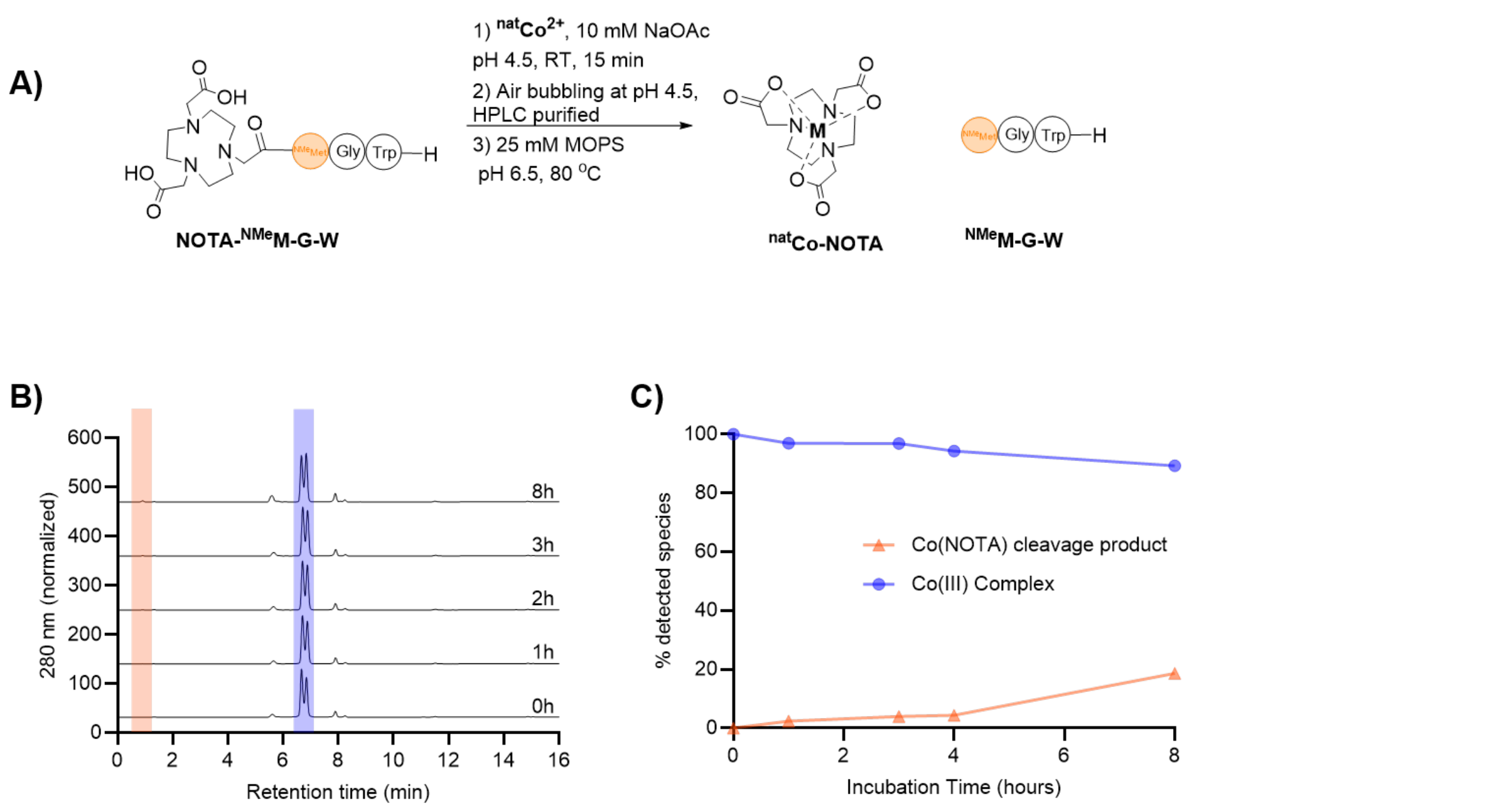

Figure S95. (A) Synthesis scheme and conditions for cleavage of **[^{nat}Co(4)]**$^{+}$. The Co^{3+} complex was formed at pH 4.5. **(B)** Analytical HPLC chromatograms (Method B) show the autolytic release of the ^{nat}Co-NOTA. **(C)** Quantification of the pH-dependent cleavage at 80 °C and pH 6.5.

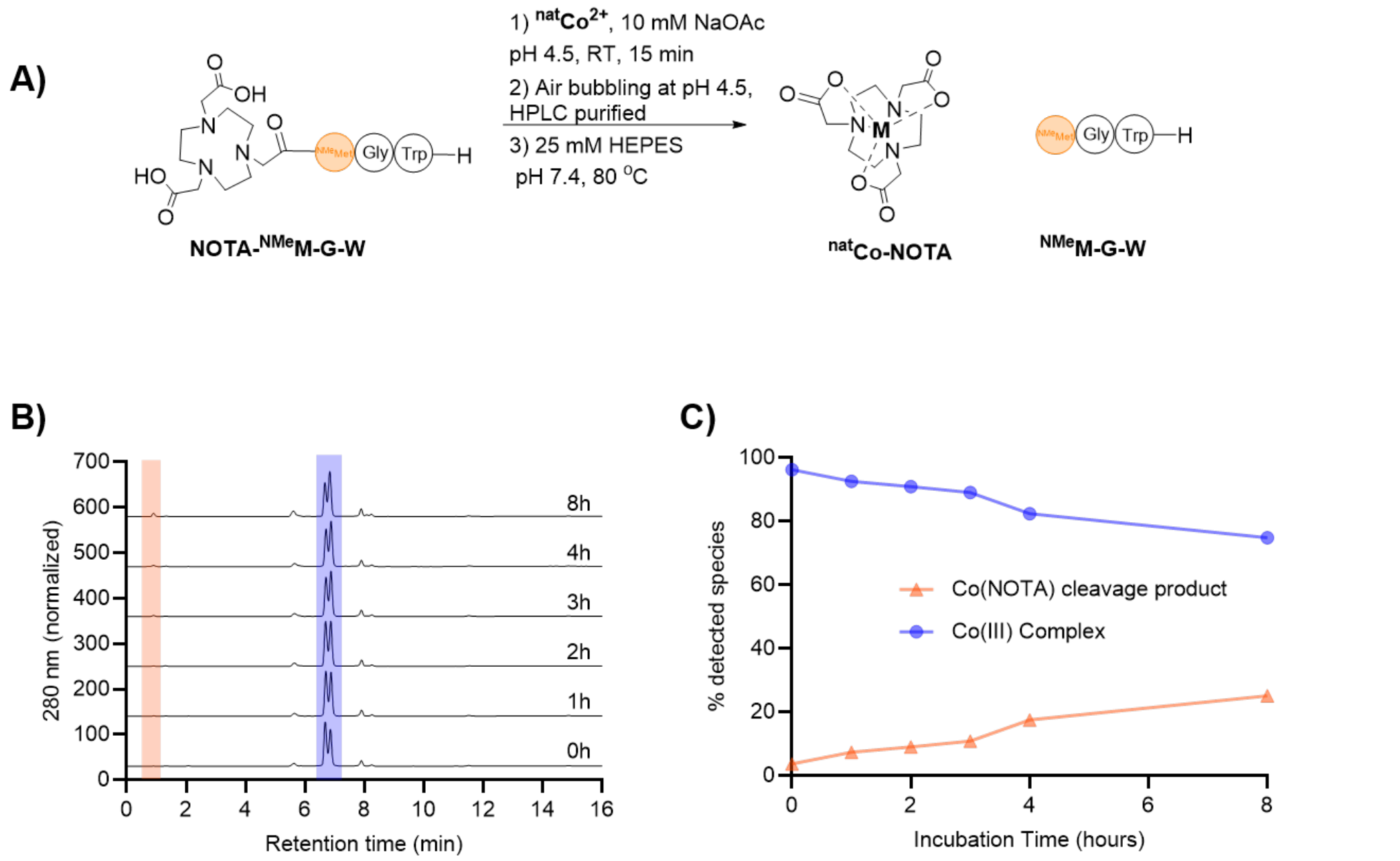

Figure S96. (A) Synthesis scheme and conditions for cleavage of **[natCo(4)]**$^{+}$. The Co^{3+} complex was formed at pH 4.5. **(B)** Analytical HPLC chromatograms (Method B) show the autolytic release of the natCo-NOTA. **(C)** Quantification of the pH-dependent cleavage at 80 ºC and pH 7.4.

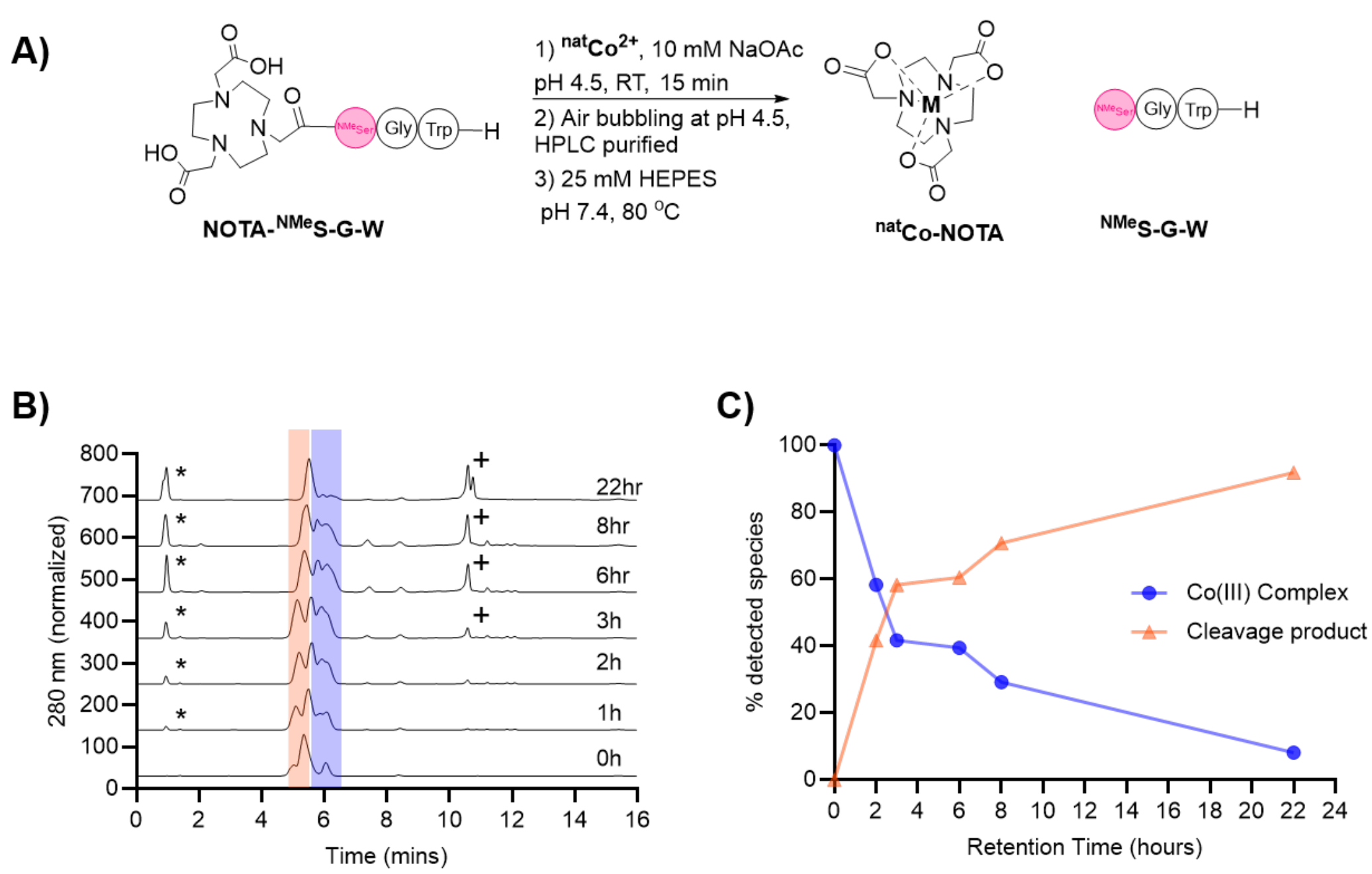

Figure S97. (A) Synthesis scheme and conditions for cleavage of **[natCo(3)]**$^{+}$. The Co^{3+} complex was formed at pH 4.5. **(B)** Analytical HPLC chromatograms (Method B) show the autolytic release of the natCo-NOTA (*). Plus sign (+) indicates the reaction side product. **(C)** Quantification of the pH-dependent cleavage at 80 ºC and pH 7.4.

5 Mechanism Studies

5.1 Metal Complex Speciation

NMR (^1H) analysis of spectroscopic titrations was performed on a Bruker Avance 400 instrument at 25 ºC. For metal-ligand complex speciation, pH-adjusted aliquots were prepared from stock solutions ([Ligand] = 0.2 mM; [Metal] = 0.2 mM; 0.1 M KCl; 0.01 M HCl). Each NMR sample was prepared by adding TMSP-d_4 (10 μL, 10 μM) to 600 μL of the pH-adjusted sample in water. A water suppression method with number of scan of 256 was employed in the absence of the deuterated solvent. ^{1}H NMR spectra were referenced to TMSP-d_4 (0.00 pm).

Potentiometric titrations were performed to determine the pH-dependent speciation of the Fe^{3+} model complexes. All solutions were prepared using CO_2-free Mili-Q water and were maintained at constant ionic strength with 0.1 M KCl. The titrations were carried out at 24.0 ± 0.1 ºC under an argon atmosphere to minimize CO_2 absorption. Titrations were carried out from pH 2.0 – 12.0, and pH was monitored by a Metrohm 6.0234.100 glass pH electrode with 3.0 M KCl reference. The glass pH electrode was calibrated before each experiment using standard buffer solutions (pH 4.0, 7.0, 10.0) and further standardized by titration of a HCl solution (0.01 M) with standardized KOH (0.1 M). The results were analyzed with the Gram procedure to obtain the standard potential (E^o) and the ionic product of water pK_w with T = 24.0 ºC and 0.1 M KCl as the supporting electrolyte. Each titration was composed of 350 with equilibrium time of 3 minutes. The results were analyzed using the Hyperquad2013 program.[4] Speciation plots were generated with HySS2009.[5]

UV-Vis spectroscopic titrations and HPLC analysis were conducted to determine pH-dependent Fe^{3+} model complex species. The titrations were conducted out-of-cell titrations in water. Metal-ligand titrations were carried out in the range of pH 2.0 – 8.0 and were monitored using a NanoDrop One UV-visible spectrophotometer and analytical HPLC (Method E). Stock solutions containing equal concentrations (0.1 mM) of metal and ligand at pH 2.0 (0.01 mM HCl) and constant ionic strength (0.1 M KCl) were prepared.

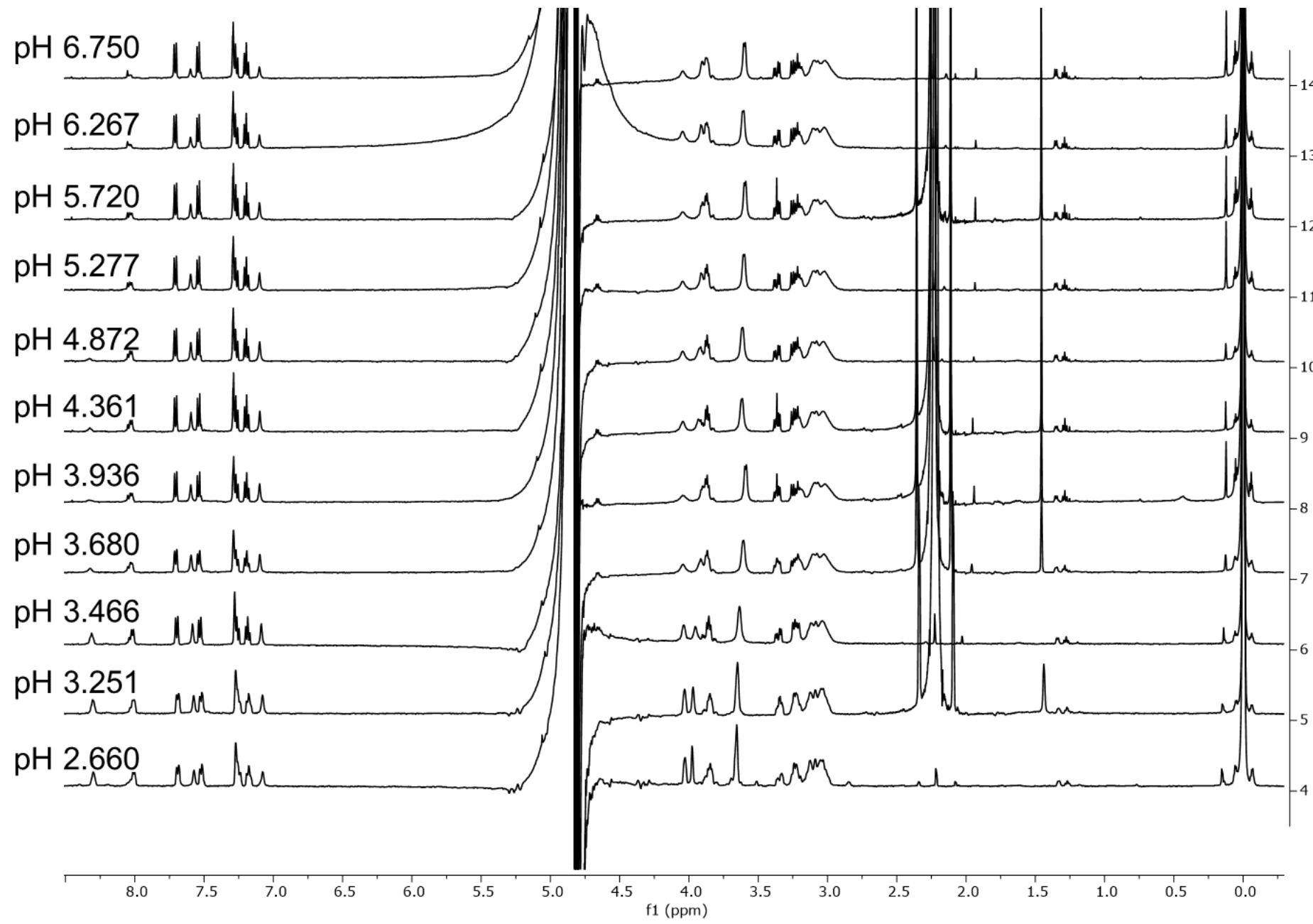

Figure S98. [In(NO2A)]$^+$-Gly-Gly-Trp ^{1}H NMR spectra as a function of pH in H_2O. [1] = 0.2 mM, [In^{3+}] = 0.2 mM, [HCl] = 0.01 M, [KCl] = 0.1 M, T = 25 °C. The NMR-based pH-dependent speciation shows no isomerization of ^{nat}In Gly-NO2A complex.

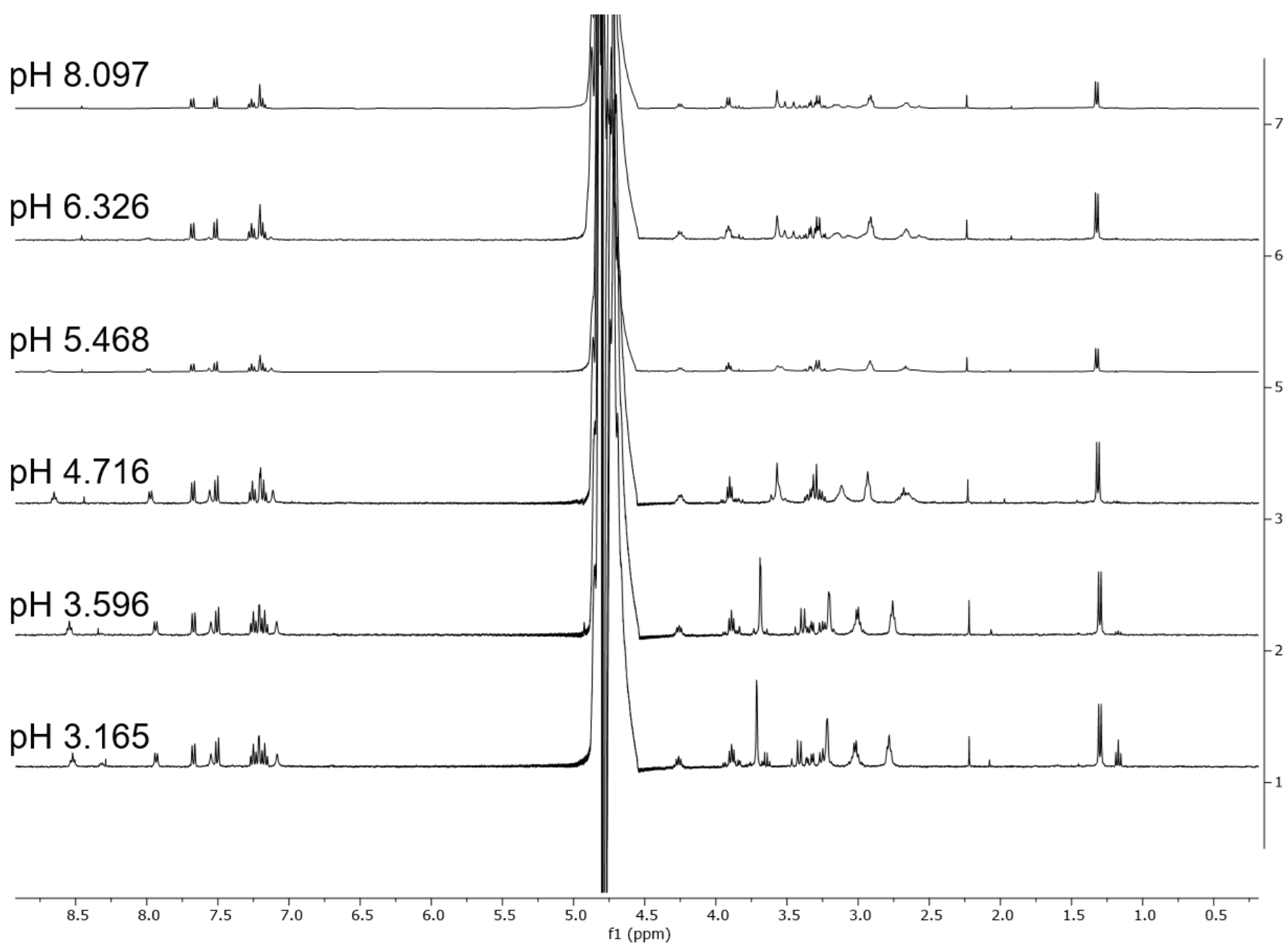

Figure S99. Ala-NO2A free ligand ^{1}H NMR spectra as a function of pH in H_2O. [5] = 0.2 mM, [HCl] = 0.01 M, [KCl] = 0.1 M, T = 25 °C.

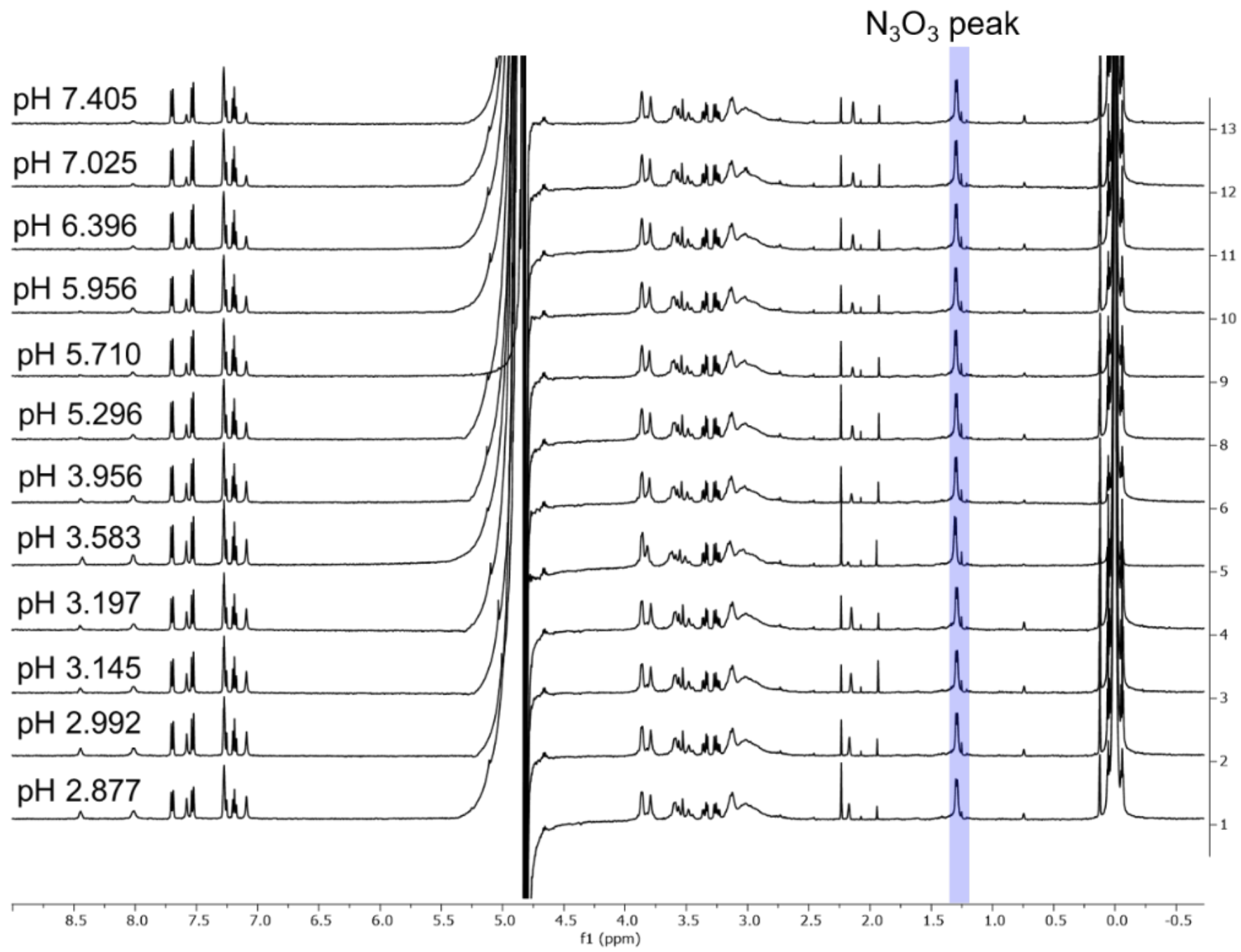

Figure S100. $[In(NO2A)]^{+}$-Ala-Gly-Gly-Trp ^{1}H NMR spectra as a function of pH in H_2O. [5] = 0.2 mM, $[In^{3+}]$ = 0.2 mM, [HCl] = 0.01 M, [KCl] = 0.1 M, T = 25 °C. The NMR-based pH-dependent speciation shows no isomerization of ^{nat}In Ala-NO2A complex.

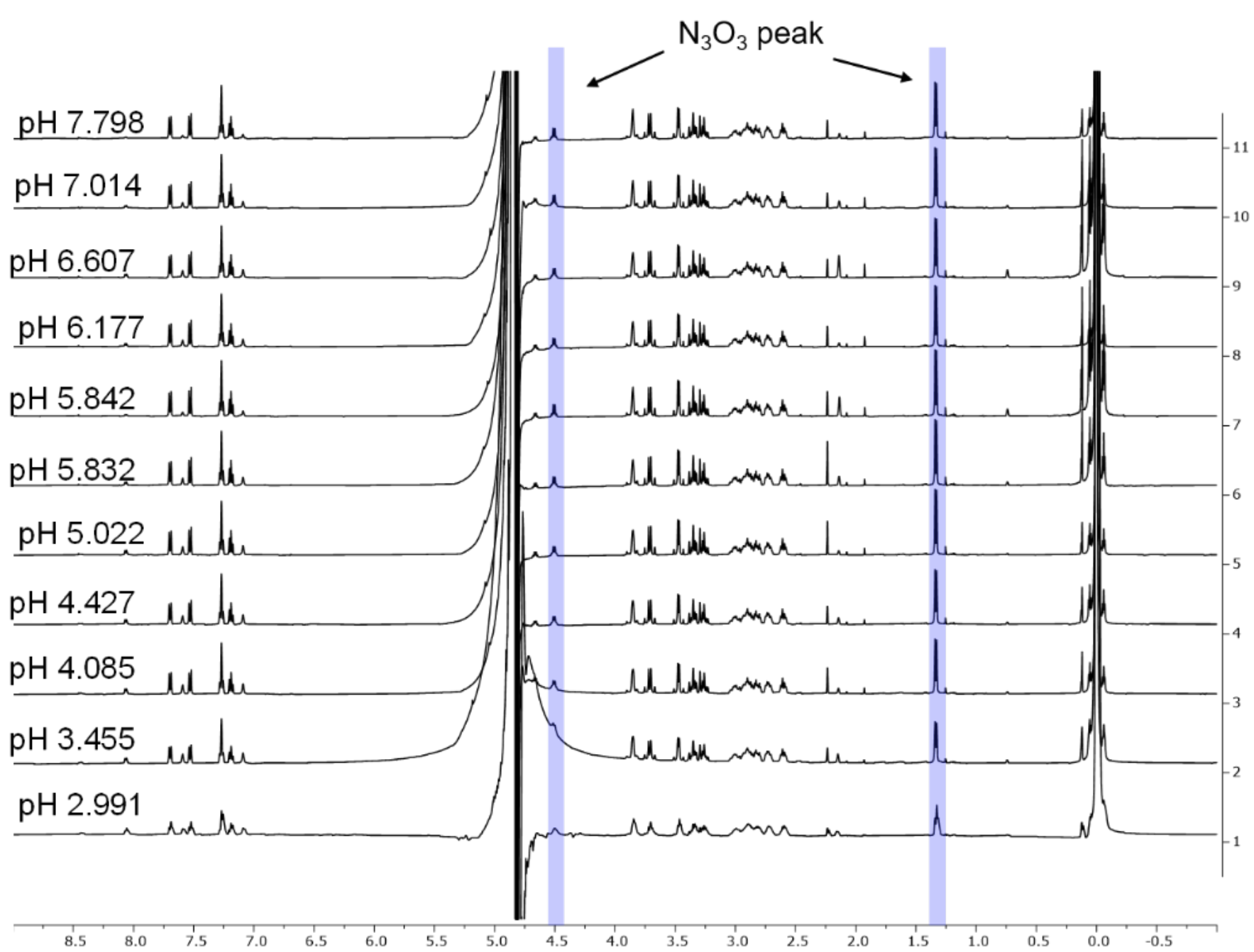

Figure S101. [Zn(NO2A)]-Ala-Gly-Trp ^{1}H NMR spectra as a function of pH in H_2O. [5] = 0.2 mM, $[Zn^{2+}]$ = 0.2 mM, [HCl] = 0.01 M, [KCl] = 0.1 M, T = 25 °C. The NMR-based pH-dependent speciation shows no isomerization of ^{nat}Zn Ala-NO2A complex.

The pH of the Co complex solution was adjusted to either 4.5 (25 mM pH 4.5 NaOAc) or 7.4 (25 mM pH 7.4 HEPES) during oxidation. The resulting Co^{3+} species was purified by semi-preparative HPLC. The concentrations of purified Co^{3+} complexes were determined by ICP-OES.

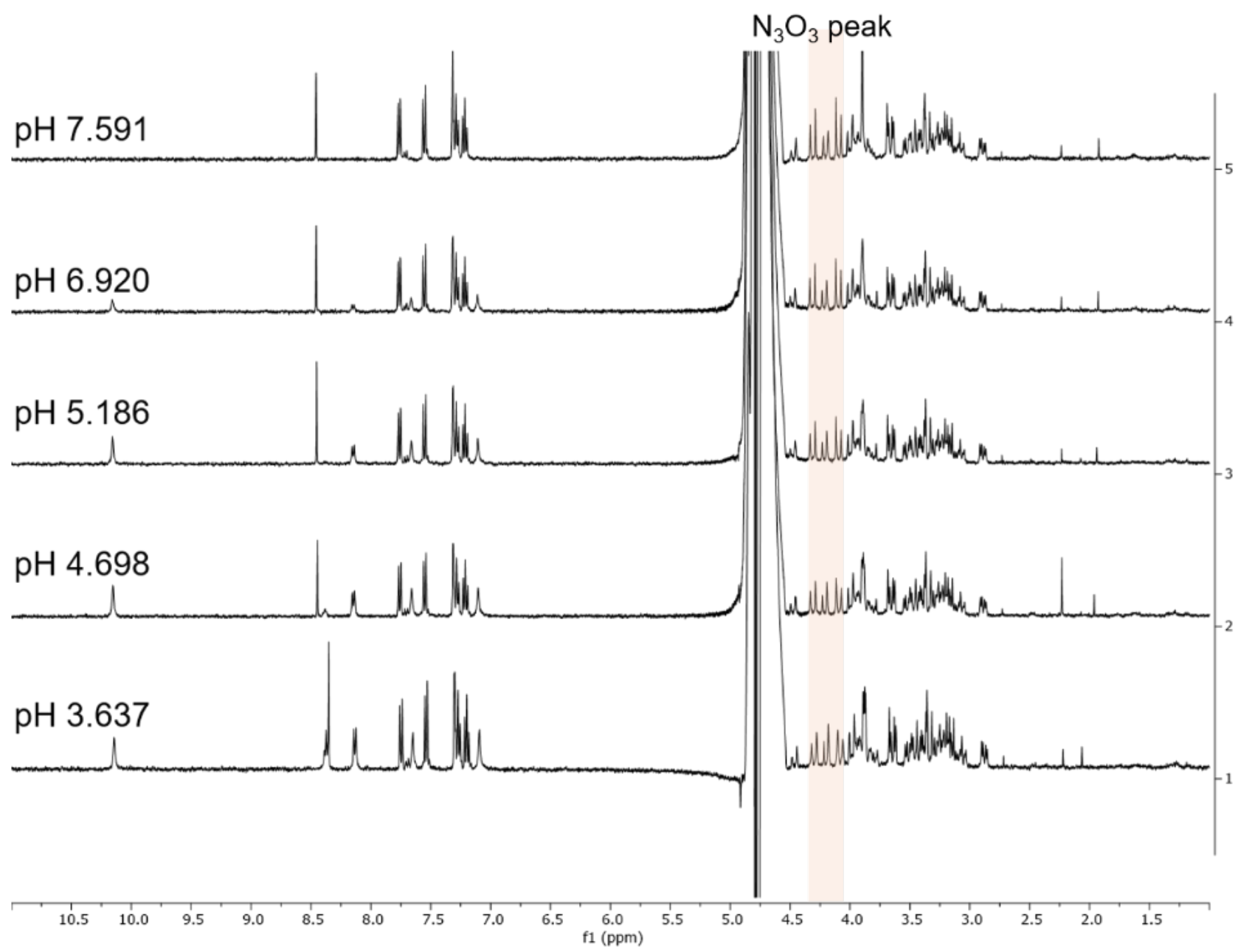

Figure S102. $[Co(NO2A)]^+$-Gly-Gly-Trp (oxidized at pH 4.5) ^1H NMR spectra as a function of pH in H_2O. [Co complex] = 0.2 mM, [HCl] = 0.01 M, [KCl] = 0.1 M, T = 25 °C.

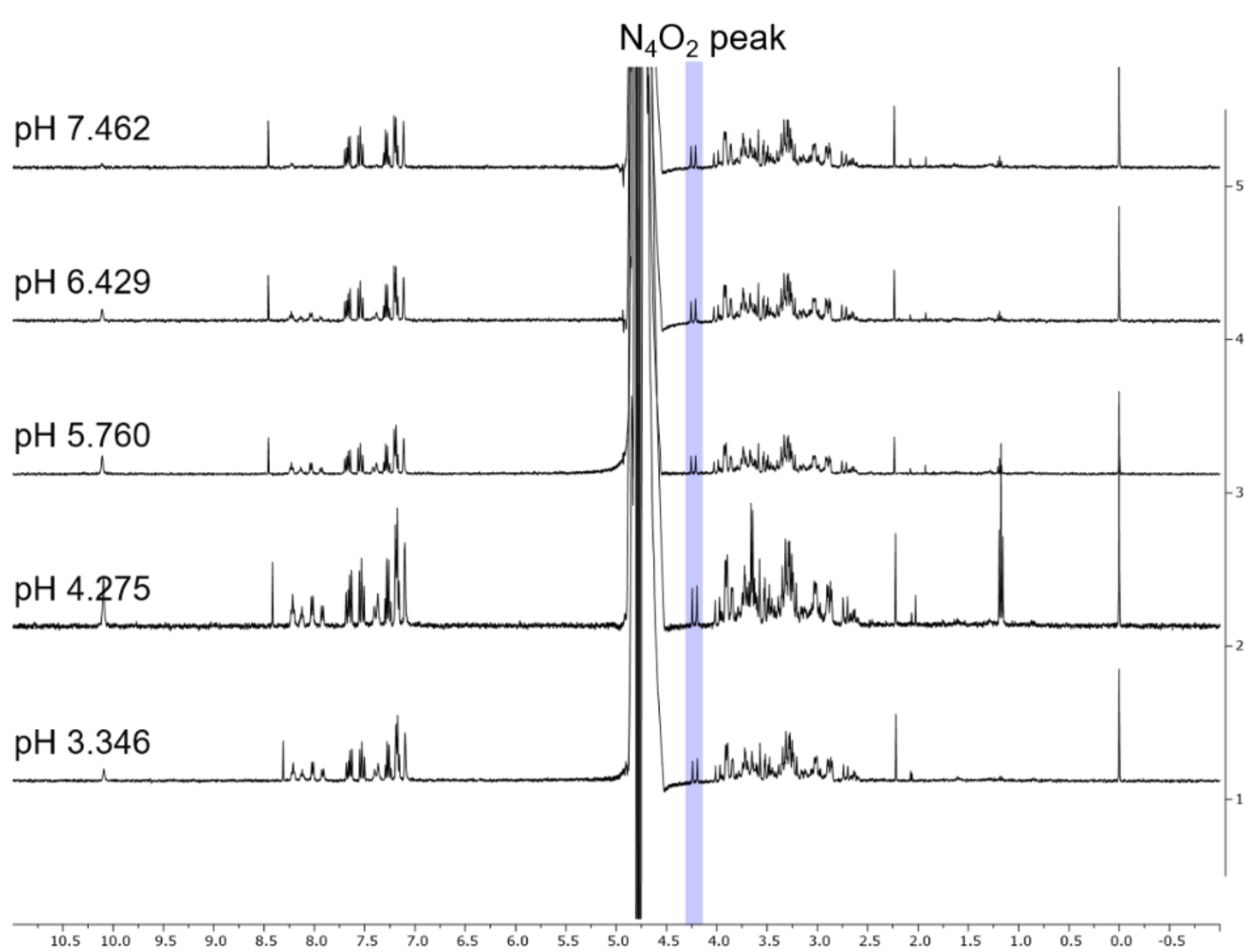

Figure S103. [Co(NO2A)]-Gly-Gly-Trp (oxidized at pH 7.0) ^1H NMR spectra as a function of pH in H_2O. [Co complex] = 0.2 mM, [HCl] = 0.01 M, [KCl] = 0.1 M, T = 25 °C.

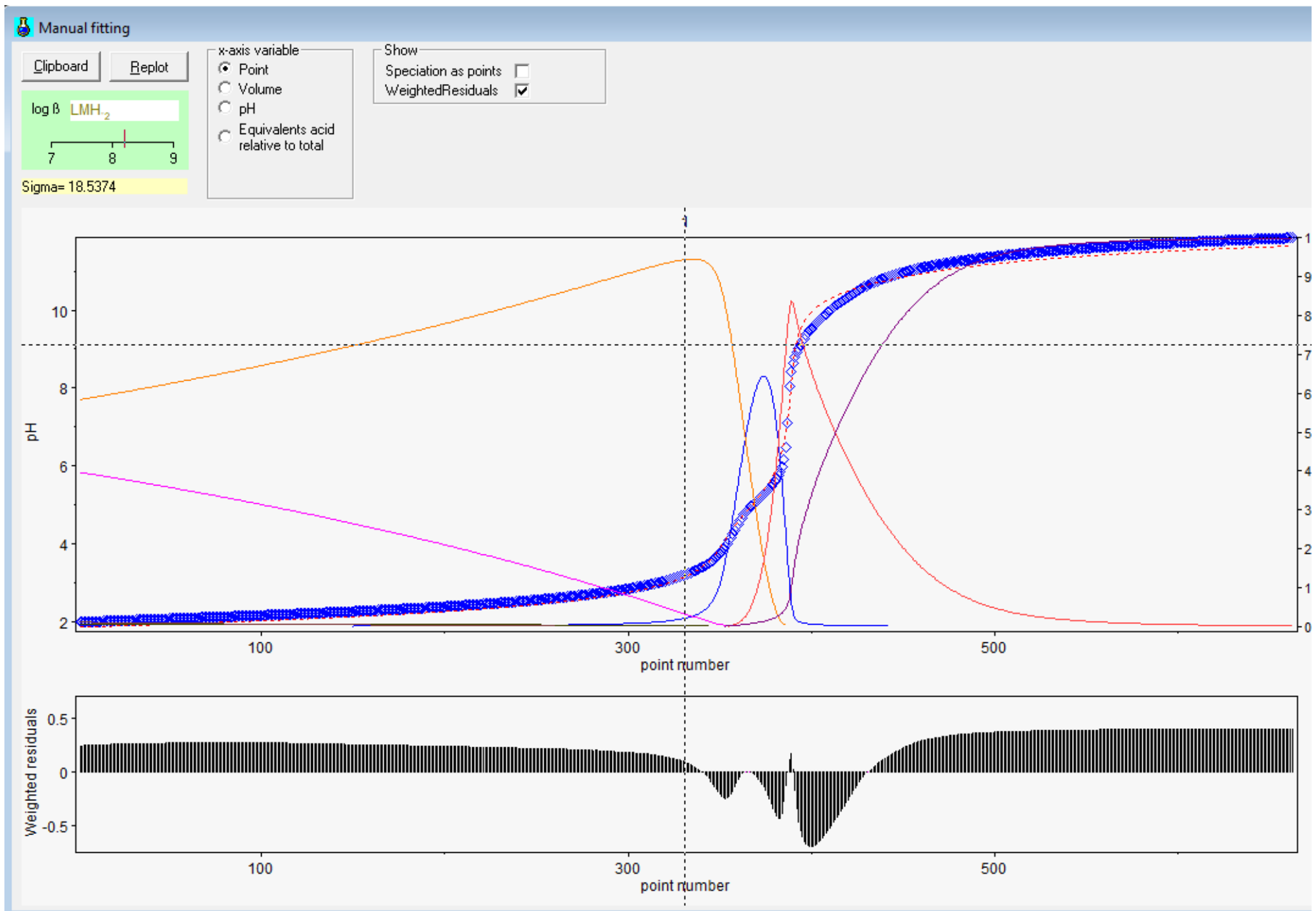

Figure S104. Representative potentiometric titration plot of 25 mL [Fe(6)]$^{+}$ ([$FeCl_3$] = 1.0 mM, [6] = 1.0 mM, [KCl] = 0.1 M, T = 23 ºC) with KOH (0.1 M) between pH 2.0 and 12.0. Experimental data points (blue) overlaid with data fit (dotted red line).

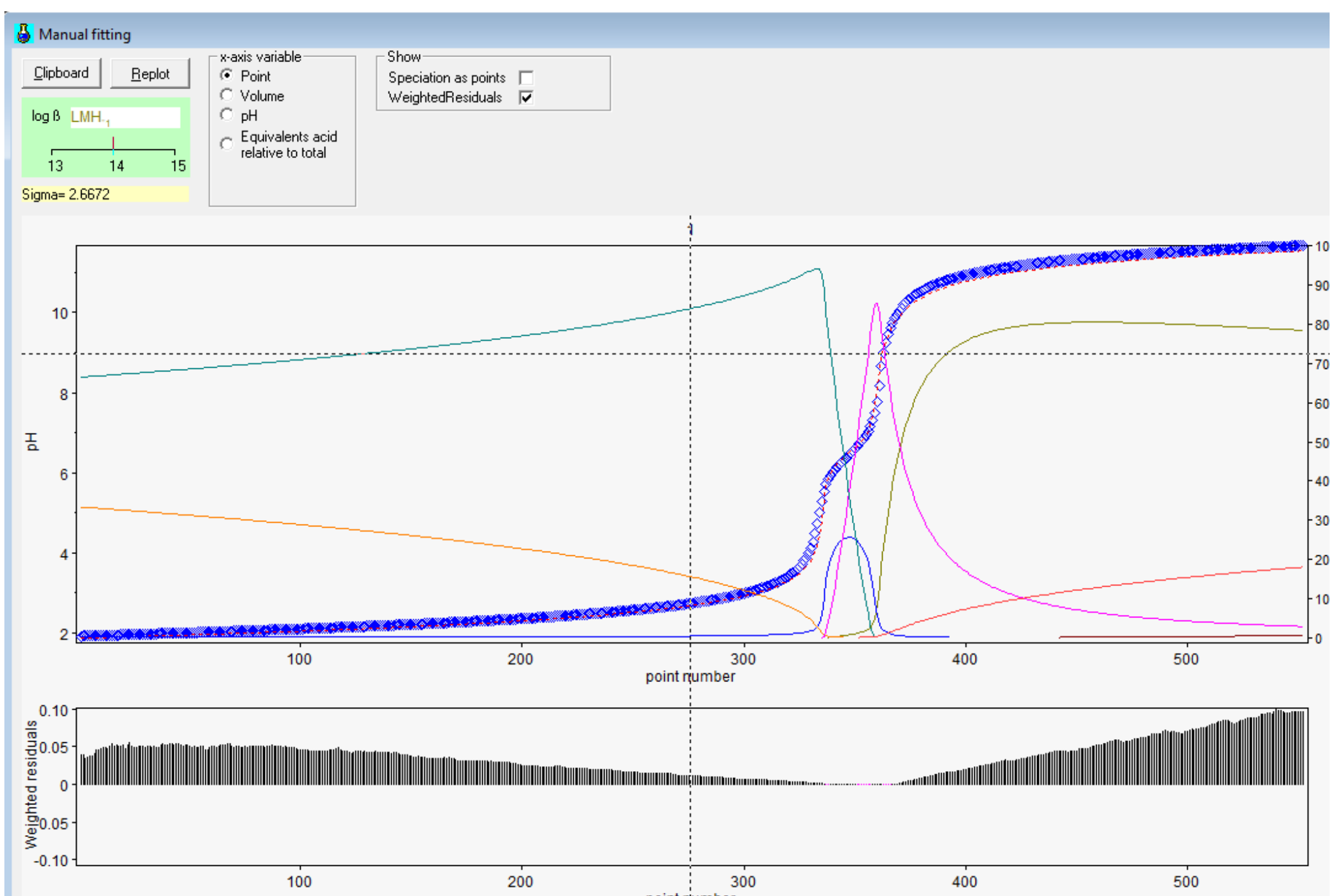

Figure S105. Representative potentiometric titration plot of 25 mL [Fe(7)]$^{+}$ ([$FeCl_3$] = 1.0 mM, [7] = 1.0 mM, [KCl] = 0.1 M, T = 23 ºC) with KOH (0.1 M) between pH 2.0 and 12.0. Experimental data points (blue) overlaid with data fit (dotted red line).

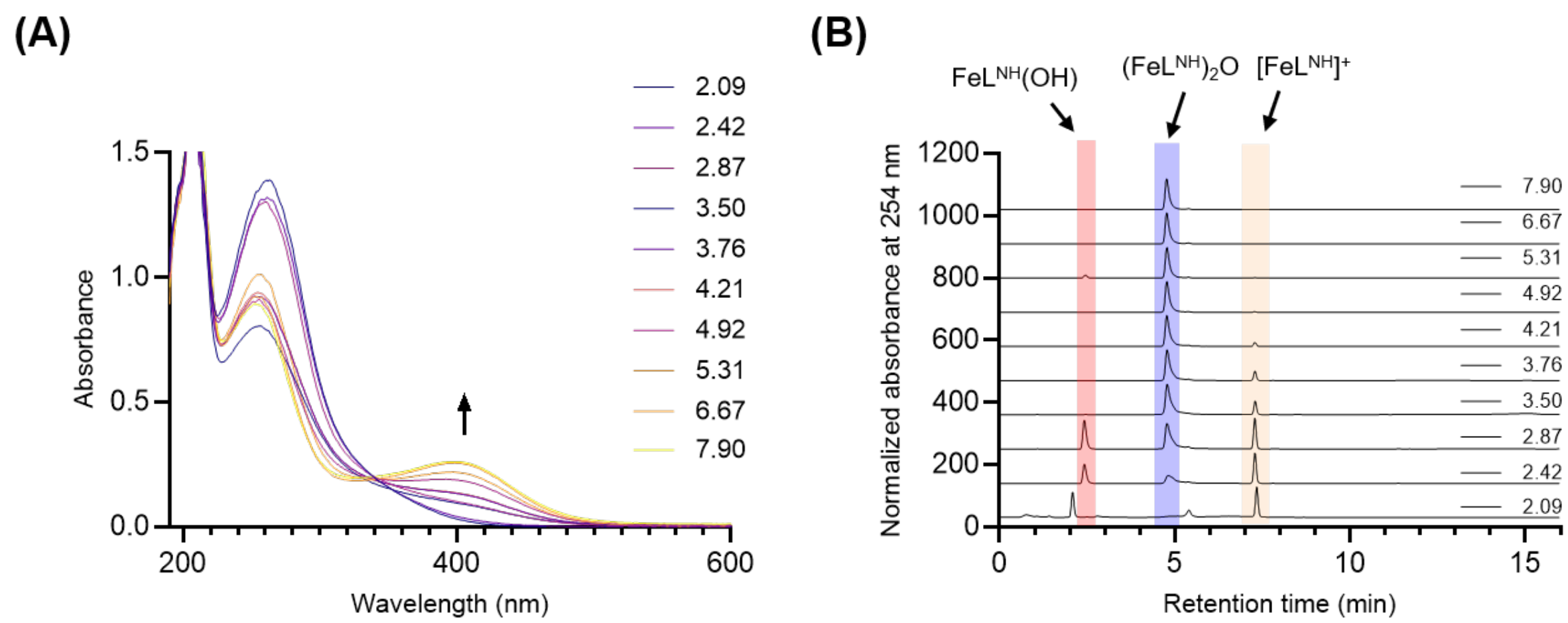

Figure S106. (A) Representative UV spectra of Fe^{3+} - L^{NHBn} (6) from UV-Vis spectrophotometric titrations (pH 2.09 – 7.90). **(B)** Corresponding HPLC analysis of titration samples (pH 2.09 – 7.90) using gradient Method E.

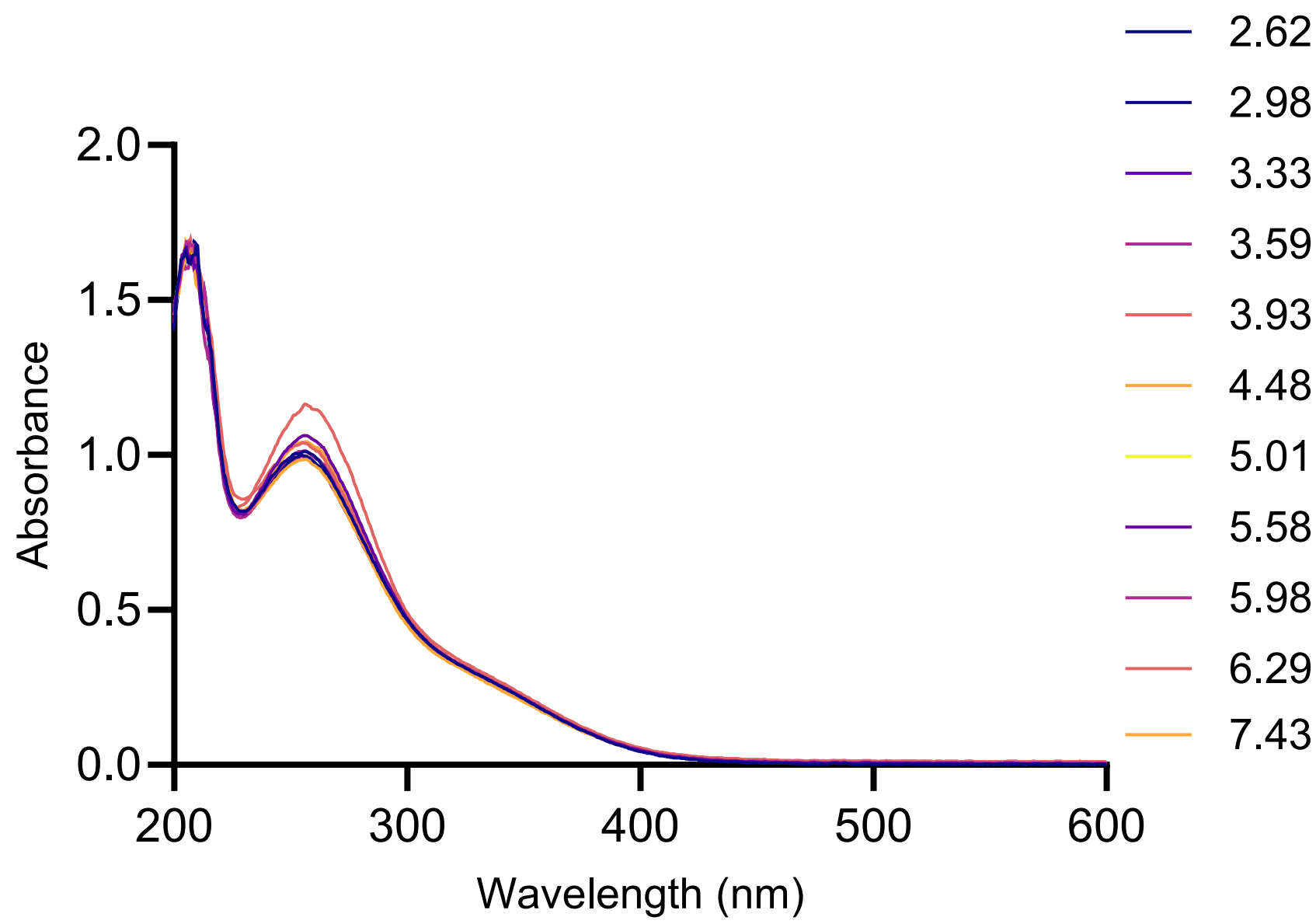

Figure S107. Representative UV spectra of Fe^{3+} - L^{NMeBn} (7) from UV-Vis spectrophotometric titrations (pH 2.62 – 7.43).

5.2 Chloride ion challenge assay on indium and gallium complexes cleavage experiment

In this study, the ^{NMe}Gly-NO2A model tripeptide's cleavage was studied. The ligand concentration and In^{3+} stock concentration were determined by UV-Vis and ICP-OES respectively. The ligand and metal stock solution were mixed in 1:1 ratio, and the pH was adjusted to desired values. Cleavage of the indium and gallium model complexes was observed under room temperature and at pH of 7.4. The reaction was monitored via analytical HPLC.

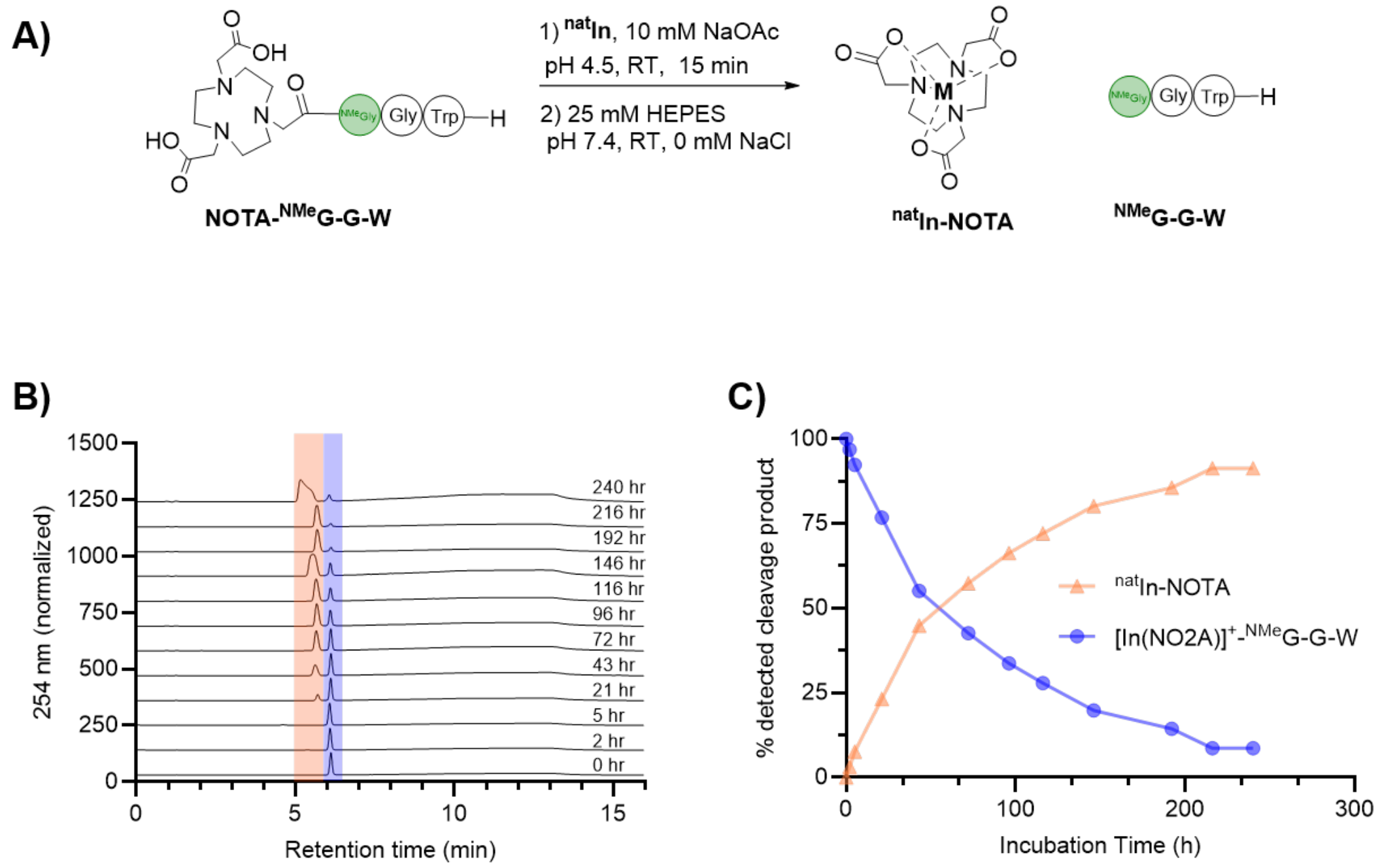

Figure S108. (A) Synthesis scheme and conditions for cleavage of [^{nat}**In(2)**]$^+$. **(B)** Analytical HPLC chromatograms (Method B) show the autolytic release of the ^{nat}In-NOTA. **(C)** Quantification of the cleavage at room temperature, pH 7.4, and 0 mM NaCl.

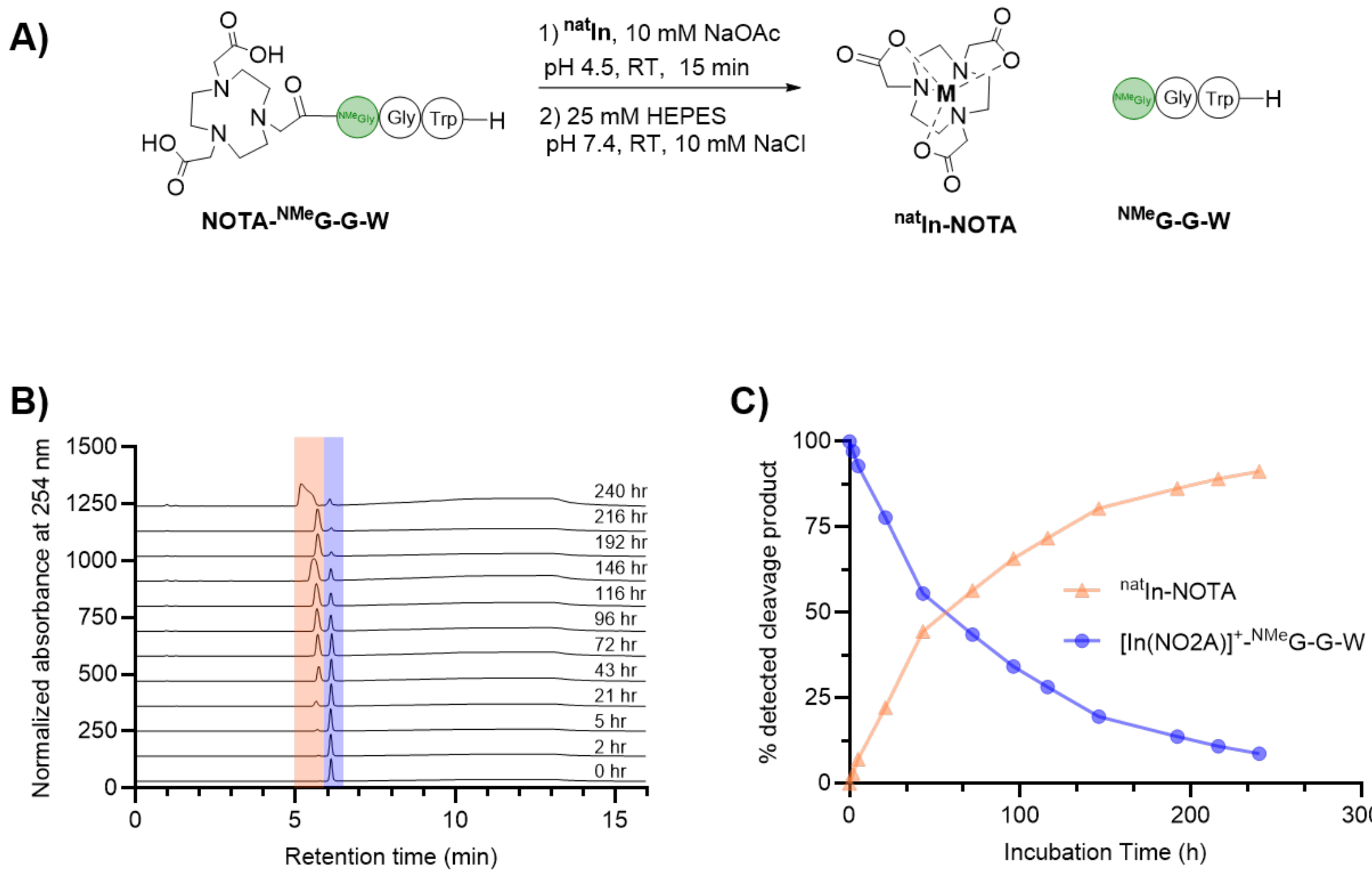

Figure S109. (A) Synthesis scheme and conditions for cleavage of [^{nat}**In(2)**]$^{+}$. **(B)** Analytical HPLC chromatograms (Method B) show the autolytic release of the ^{nat}In-NOTA. **(C)** Quantification of the cleavage at room temperature, pH 7.4, and 10 mM NaCl.

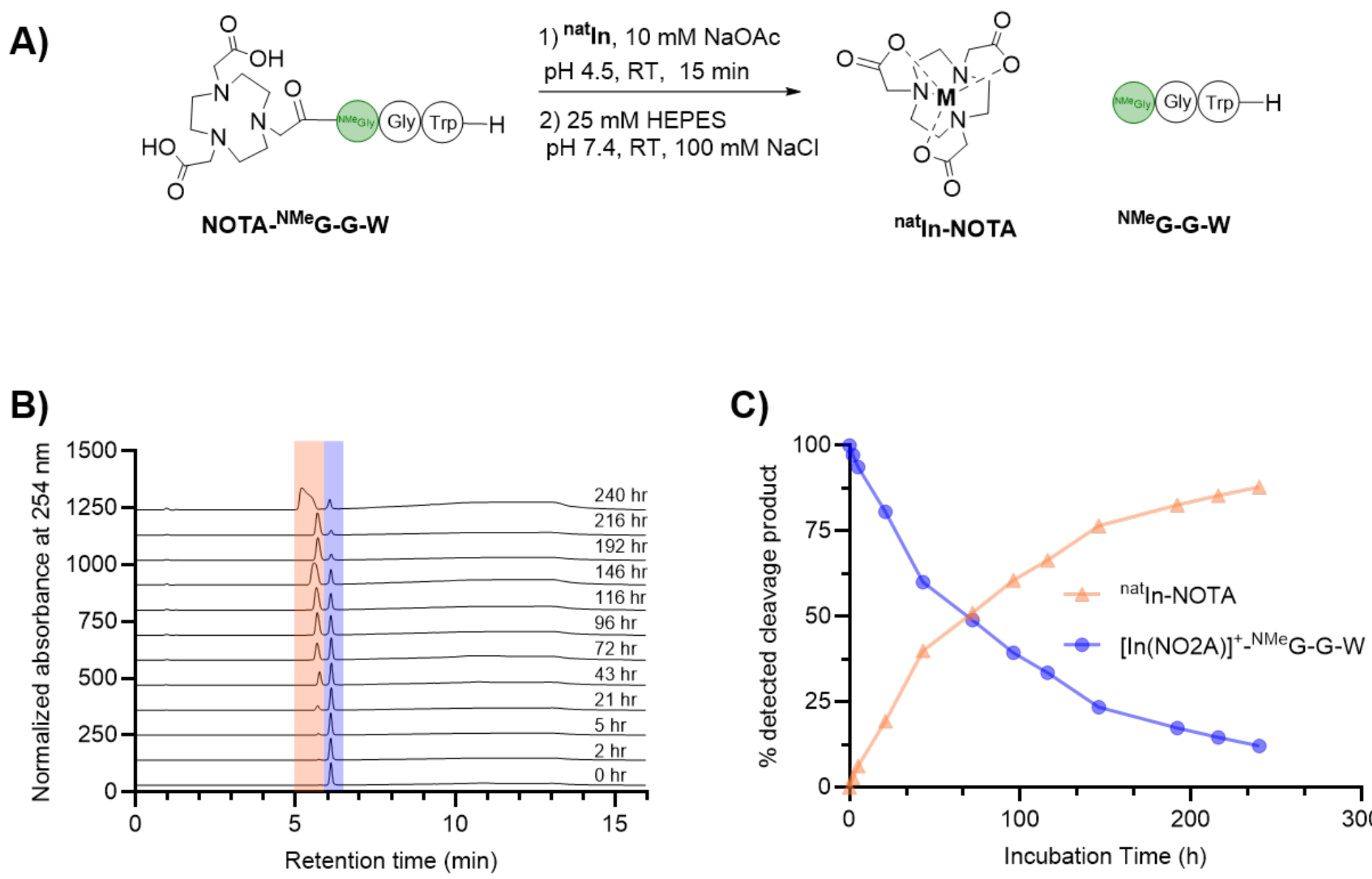

Figure S110. (A) Synthesis scheme and conditions for cleavage of [^{nat}**In(2)**]$^{+}$. **(B)** Analytical HPLC chromatograms (Method B) show the autolytic release of the ^{nat}In-NOTA. **(C)** Quantification of the cleavage at room temperature, pH 7.4, and 100 mM NaCl.

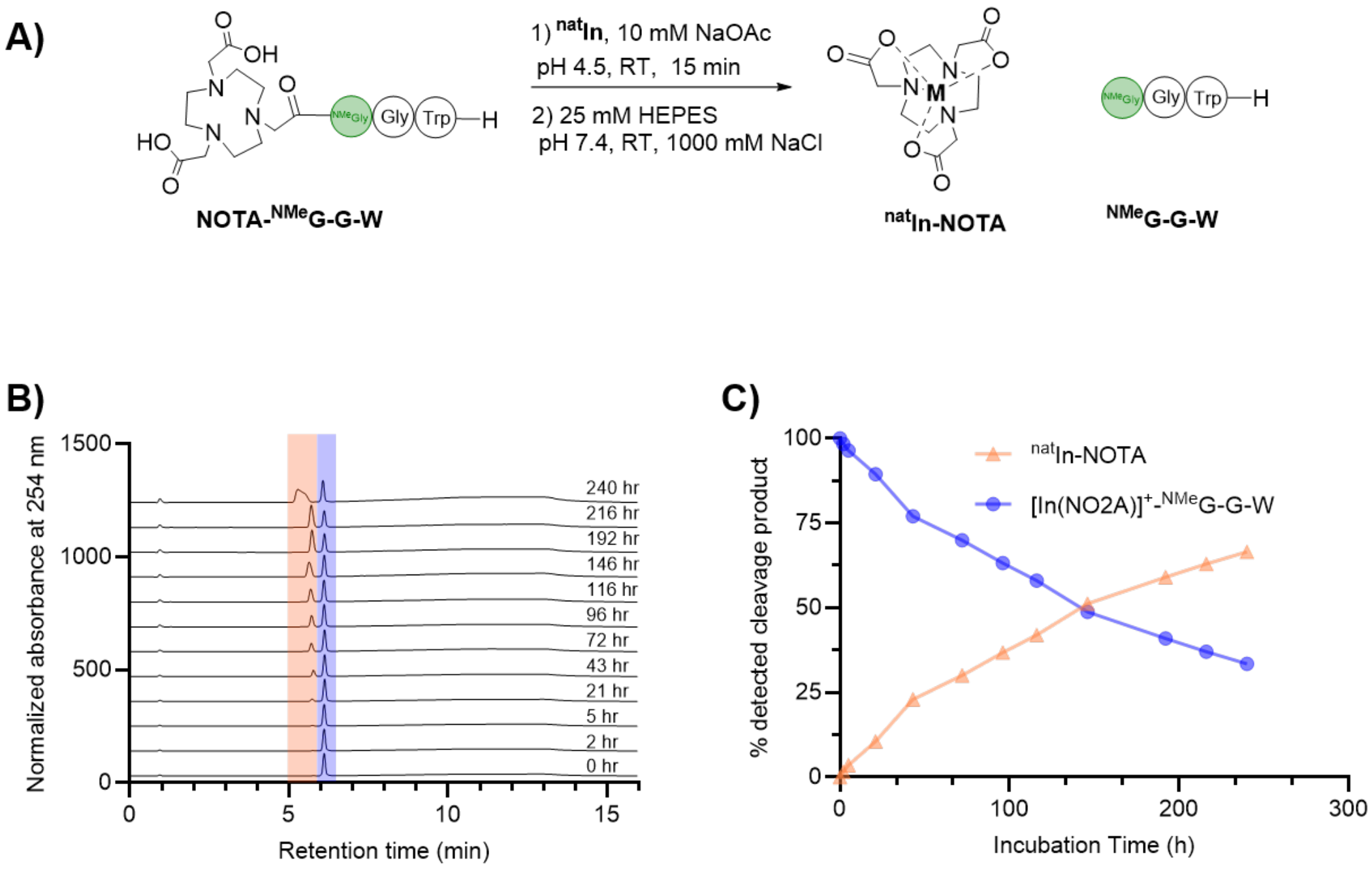

Figure S111. (A) Synthesis scheme and conditions for cleavage of [^{nat}**In(2)**]$^{+}$. **(B)** Analytical HPLC chromatograms (Method B) show the autolytic release of the ^{nat}In-NOTA. **(C)** Quantification of the cleavage at room temperature, pH 7.4, and 1000 mM NaCl.

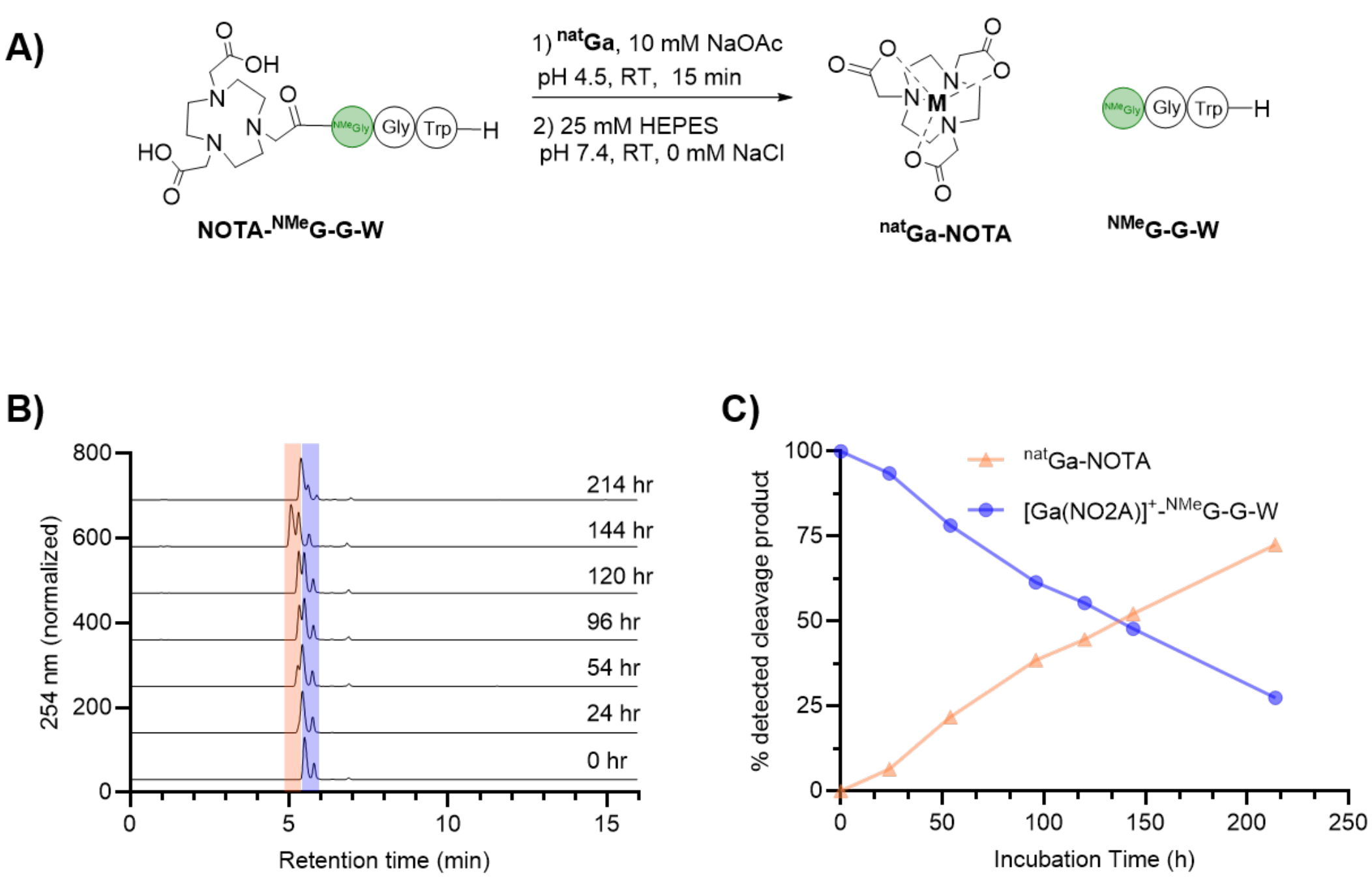

Figure S112. (A) Synthesis scheme and conditions for cleavage of [^{nat}**Ga(2)**]$^{+}$. **(B)** Analytical HPLC chromatograms (Method B) show the autolytic release of the ^{nat}Ga-NOTA. **(C)** Quantification of the cleavage at room temperature, pH 7.4, and 0 mM NaCl.

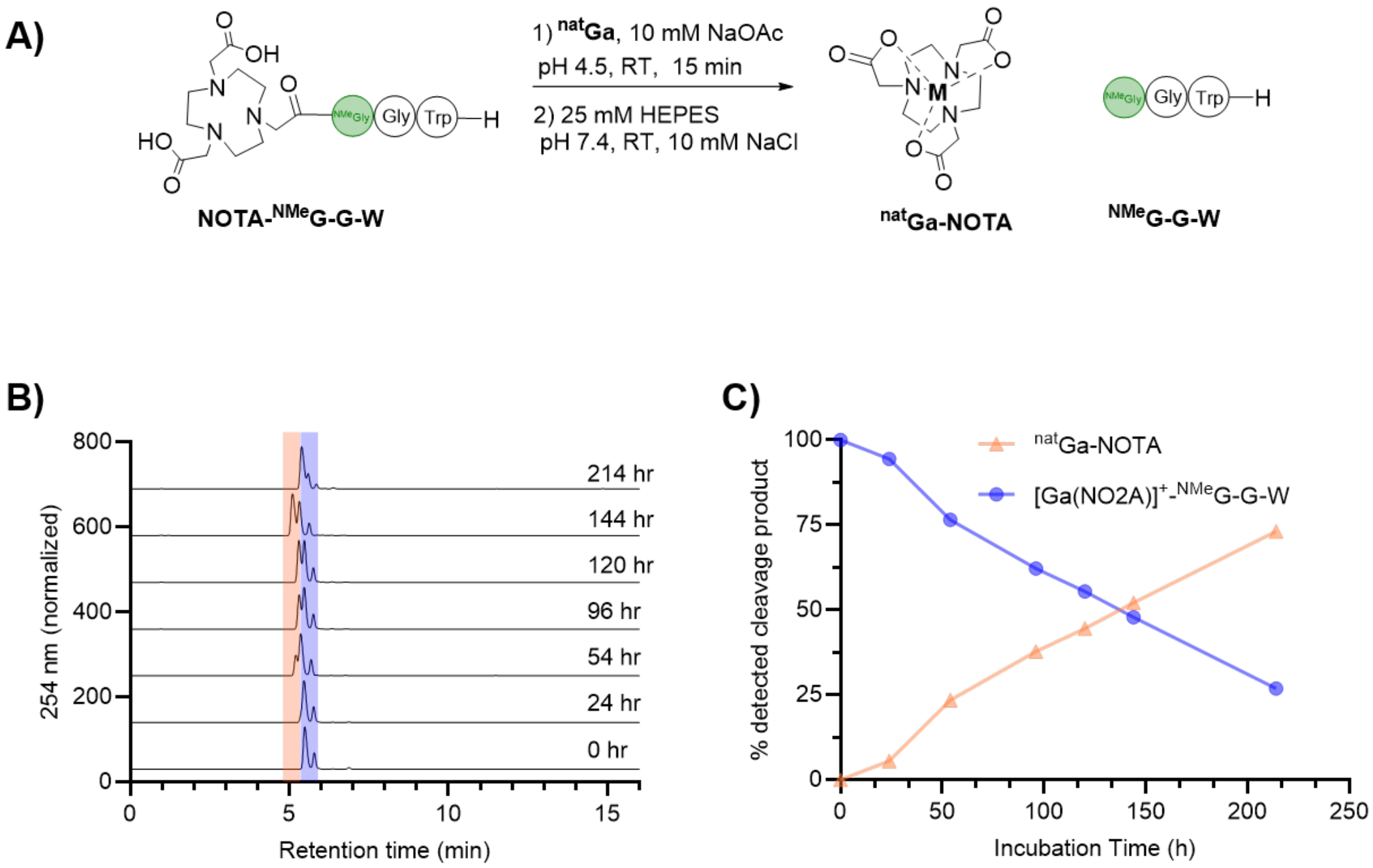

Figure S113. **(A)** Synthesis scheme and conditions for cleavage of [^{nat}**Ga(2)**]$^+$. **(B)** Analytical HPLC chromatograms (Method B) show the autolytic release of the ^{nat}Ga-NOTA. **(C)** Quantification of the cleavage at room temperature, pH 7.4, and 10 mM NaCl.

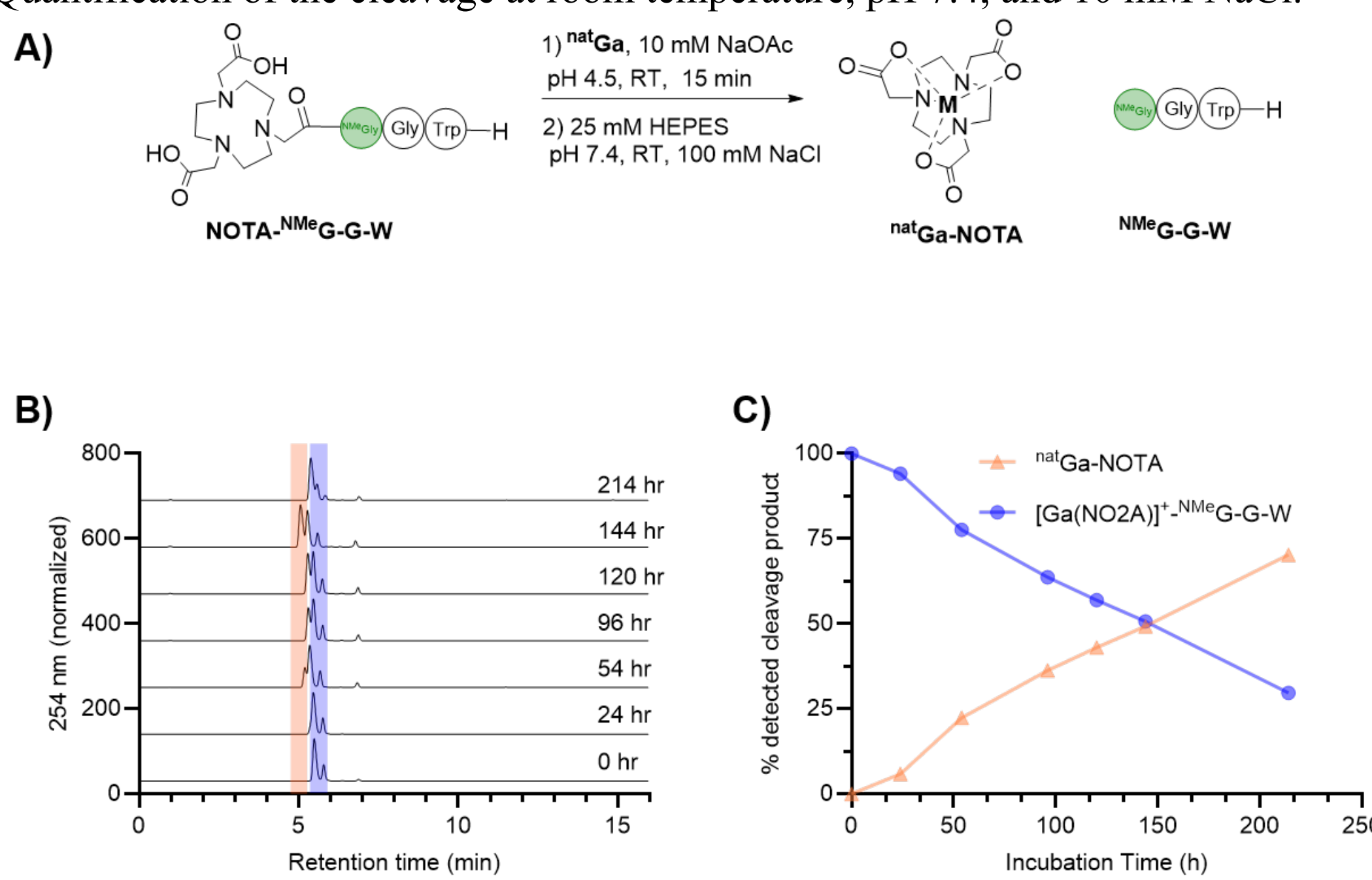

Figure S114. **(A)** Synthesis scheme and conditions for cleavage of [^{nat}**Ga(2)**]$^+$. **(B)** Analytical HPLC chromatograms (Method B) show the autolytic release of the ^{nat}Ga-NOTA. **(C)** Quantification of the cleavage at room temperature, pH 7.4, and 100 mM NaCl.

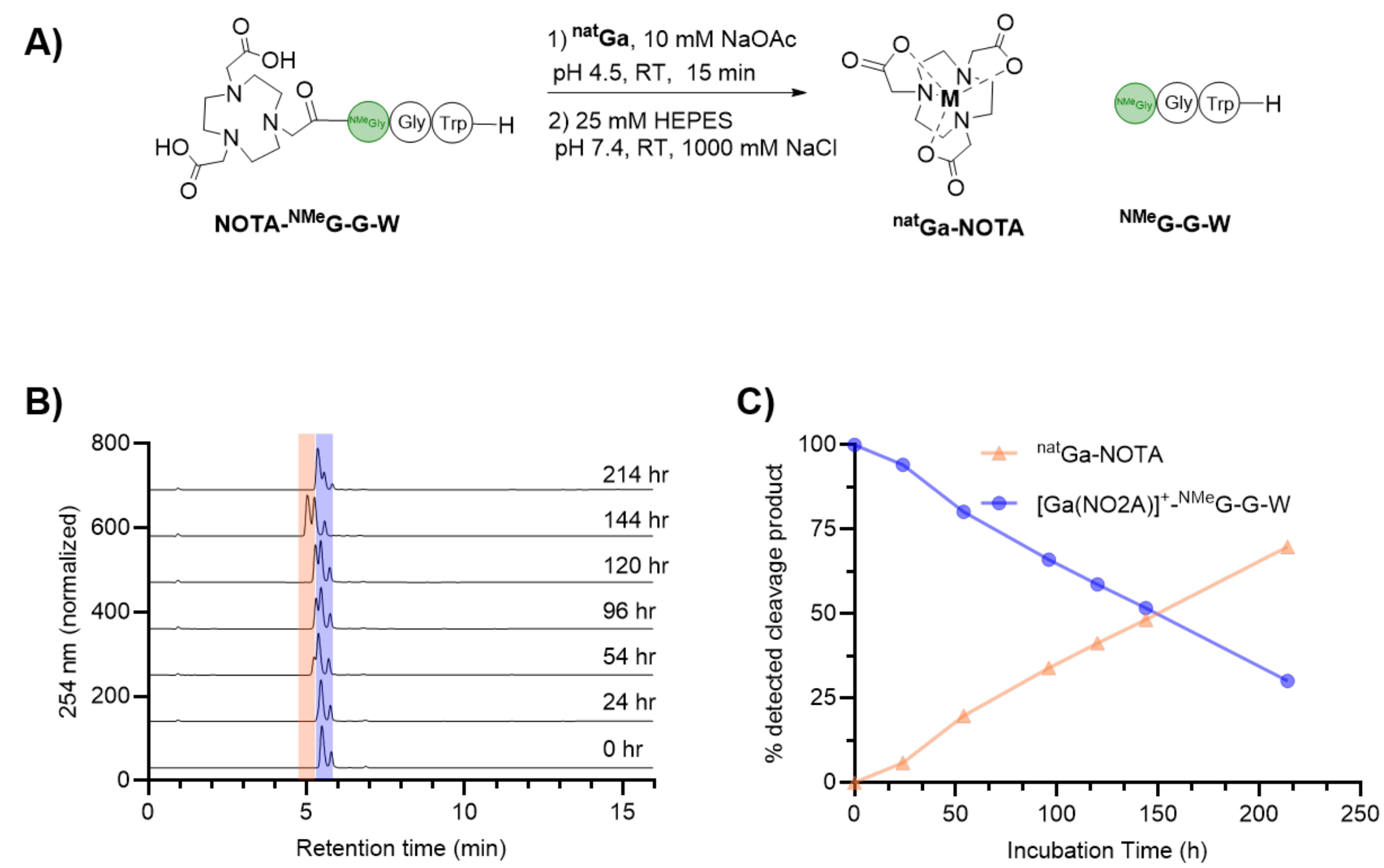

Figure S115. (A) Synthesis scheme and conditions for cleavage of [**natGa(2)**]$^{+}$. **(B)** Analytical HPLC chromatograms (Method B) show the autolytic release of the natGa-NOTA. **(C)** Quantification of the cleavage at room temperature, pH 7.4, and 1000 mM NaCl.

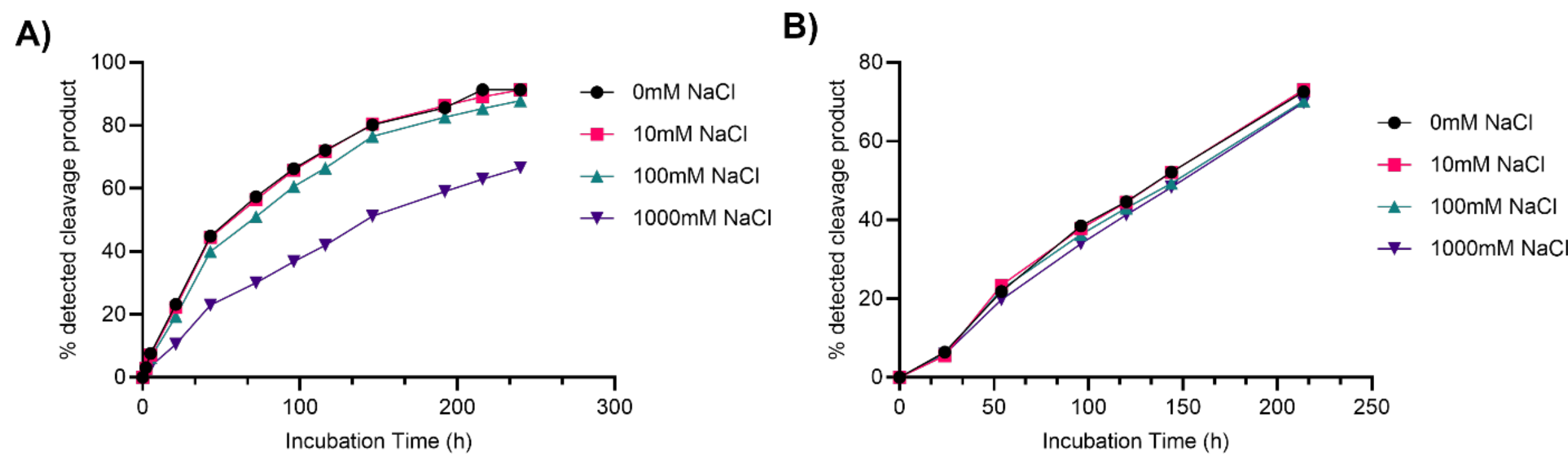

Figure S116. (A) Quantification of [In(NO2A)]$^{+}$-^{NMe}G-G-W complex cleavage experiment with increasing concentration of chloride ions at room temperature and pH 7.4. **(B)** Quantification of [Ga(NO2A)]$^{+}$-^{NMe}G-G-W complex cleavage experiment with increasing concentration of chloride ions at room temperature and pH 7.4.

6 Synthesis and Characterizations of MMAE Conjugates.

6.1 Synthesis and characterization of PD1 and PD2 conjugates

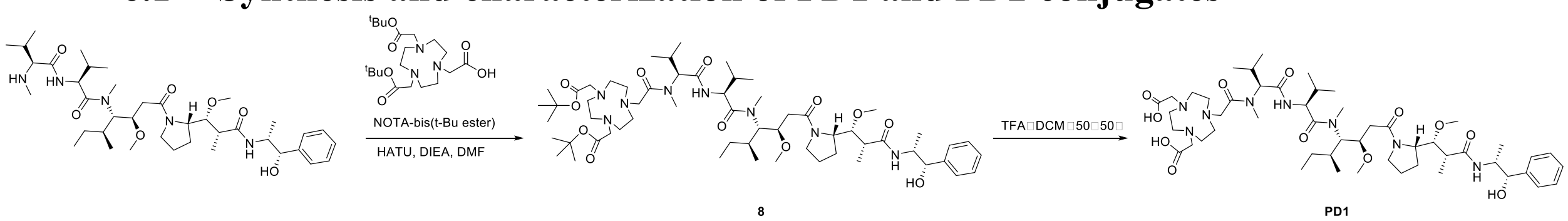

Scheme S1. Synthesis scheme for the PD1 conjugate.

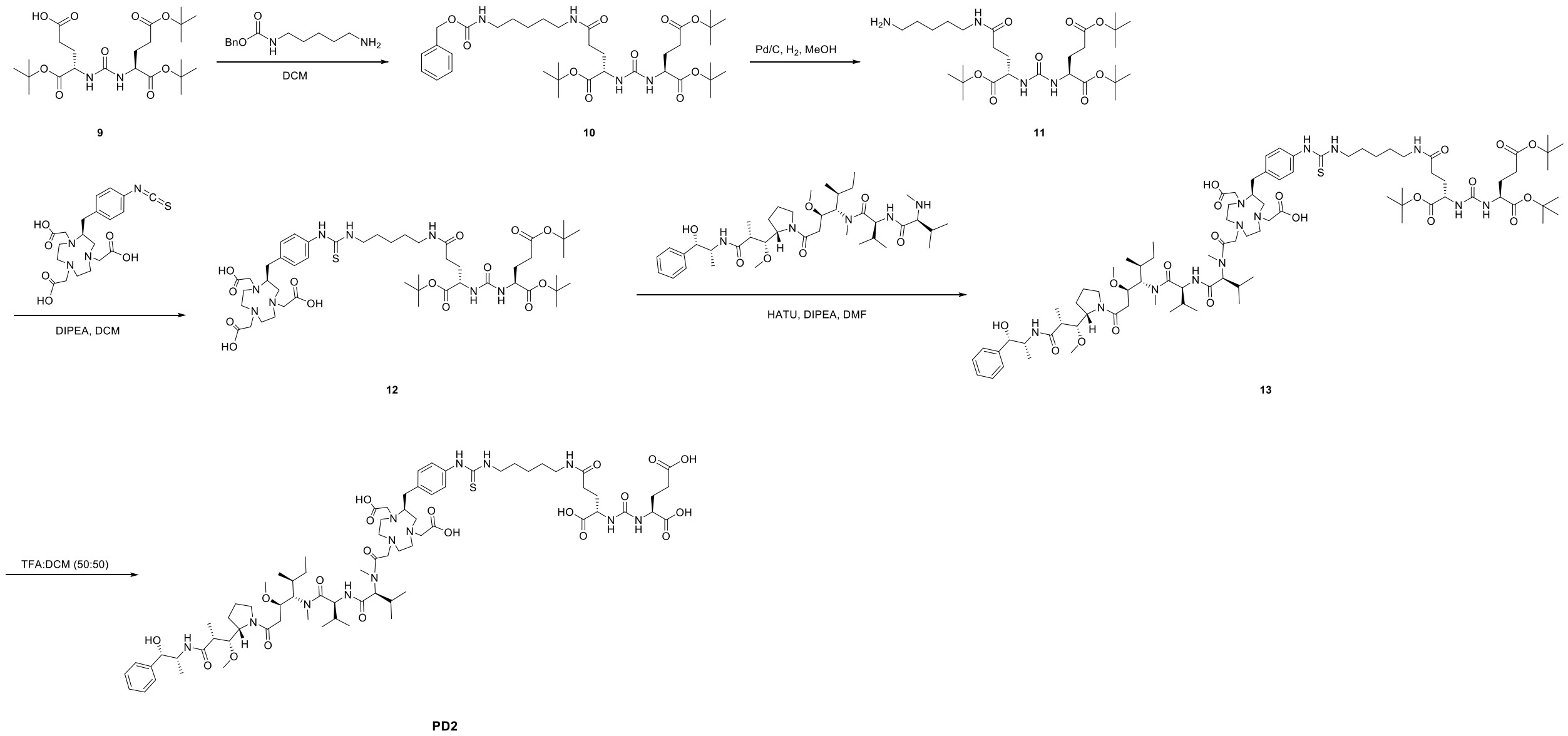

Scheme S2. Synthesis scheme for the PD2 conjugate.

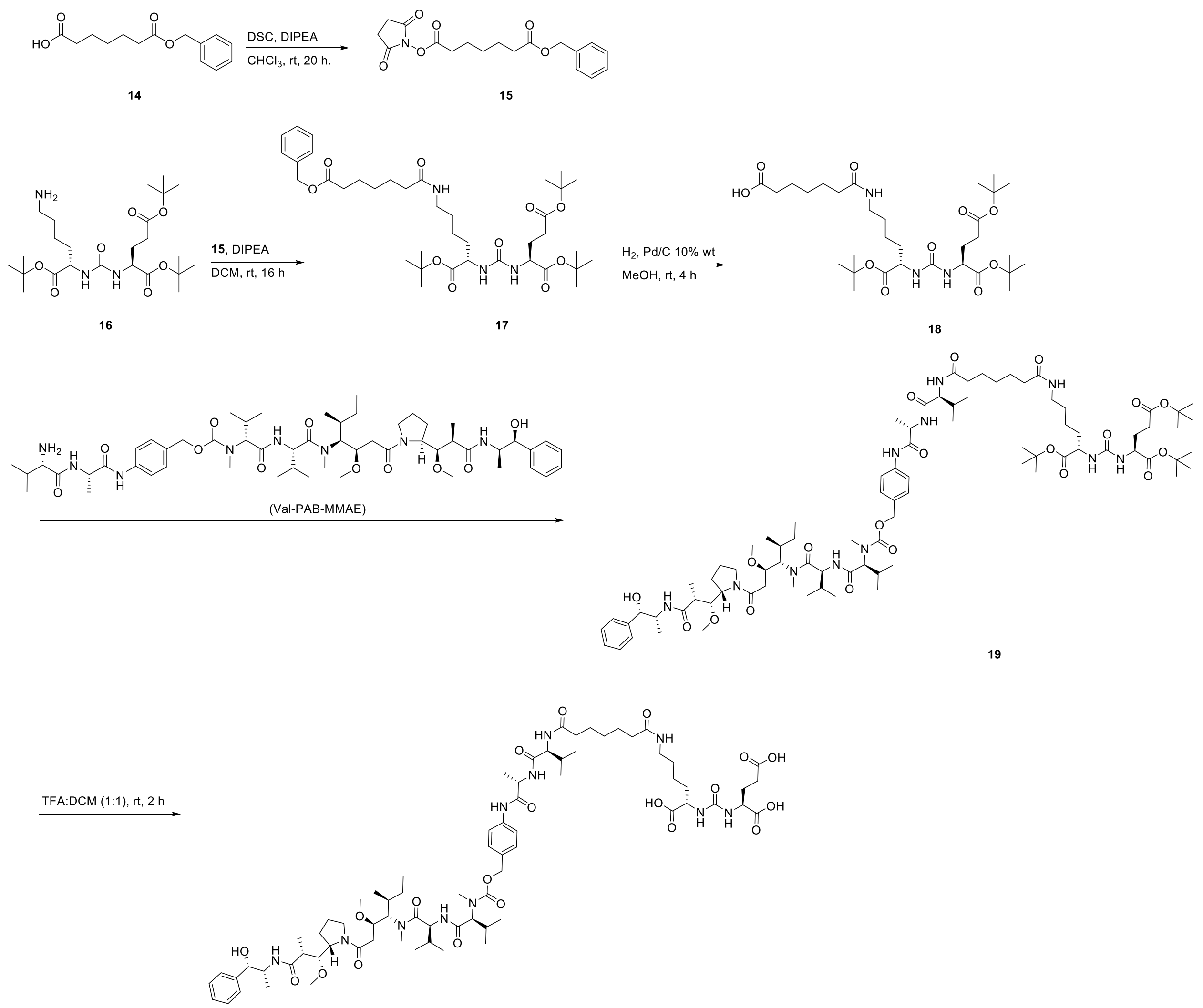

Scheme S3. Synthesis scheme for the PD3 conjugate.

Synthesis of tert-butyl protected PD1 conjugate, 8. NOTA-bis(t-Bu ester) (57.9 mg, 0.14 mmol, 2 equiv.) and HATU (39.7 mg, 0.10 mmol, 1.5 equiv) were dissolved in 1 mL of DMF. To the solution DIPEA (48.5 µL, 0.28 mmol, 4 equiv.) was added. The solution immediately turned to yellow. After stirring for 15 minutes, MMAE (50.0 mg, 0.07 mmol, 1 equiv.) was added. The solution was allowed to stir for 2 hours at room temperature. After 2 hours, the solvent was removed by a stream of N_2 gas. The crude material was purified using Semi-preparative HPLC (Method G). Pure fractions were collected and lyophilized to yield a white product (15.8 mg, 0.014 mmol, 20.3%). ^{1}H NMR (500 MHz, CD3CN) δ 10.38 (s, 1H), 7.44 – 7.10 (m, 5H), 4.74 – 4.51 (m, 2H), 4.42 (dd, J = 19.5, 9.4 Hz, 1H), 4.09 (ddq, J = 17.5, 7.3, 3.2 Hz, 2H), 3.95 (d, J = 4.7 Hz, 1H), 3.80 (dd, J = 9.0, 2.4 Hz, 1H), 3.70 – 3.42 (m, 6H), 3.35 – 3.21 (m, 6H), 3.21 – 2.79 (m, 18H), 2.28 (ddt, J = 17.7, 12.8, 5.0 Hz, 1H), 2.18 (s, 11H), 2.11 – 2.01 (m, 1H), 1.91 – 1.54 (m, 4H), 1.45 (d, J = 2.7 Hz, 18H), 1.12 (dd, J = 16.5, 6.8 Hz, 3H), 1.05 – 0.98 (m, 4H), 0.98 – 0.78 (m, 18H), 0.79 – 0.62 (m, 1H).

Synthesis of PD1. Compound **8** (15.8 mg, 0.014 mmol) was dissolved in 2 mL of 2:8 DCM:TFA. The solution was allowed to stir for 2 hours. The excess solvent was removed by blowing with N_2 gas. The crude material was purified using Semi-preparative HPLC (Method

G). Pure fractions were collected and lyophilized to yield a white product (2.50 mg, 0.0025 mmol, 17.6%). ^{1}H NMR (600 MHz, CD3CN) δ 7.41 – 7.28 (m, 4H), 7.27 – 7.16 (m, 1H), 6.81 (d, J = 9.9 Hz, 1H), 6.70 – 6.54 (m, 1H), 4.71 (t, J = 5.0 Hz, 1H), 4.69 – 4.63 (m, 1H), 4.63 – 4.58 (m, 1H), 4.58 – 4.40 (m, 1H), 4.17 – 3.97 (m, 2H), 3.89 (ddt, J = 7.6, 4.8, 2.4 Hz, 1H), 3.80 (qt, J = 7.5, 4.4 Hz, 1H), 3.71 – 3.63 (m, 2H), 3.63 – 3.50 (m, 5H), 3.46 (dtd, J = 16.3, 9.9, 4.1 Hz, 2H), 3.37 (dt, J = 10.0, 6.6 Hz, 1H), 3.34 – 3.30 (m, 4H), 3.25 (d, J = 6.6 Hz, 3H), 3.17 (d, J = 31.2 Hz, 3H), 3.11 – 2.54 (m, 24H), 2.49 – 2.42 (m, 1H), 2.38 (dd, J = 7.0, 3.7 Hz, 2H), 2.26 – 2.13 (m, 2H), 2.06 (dddd, J = 28.9, 19.6, 11.6, 6.6 Hz, 2H), 1.90 – 1.78 (m, 2H), 1.79 – 1.65 (m, 2H), 1.64 – 1.48 (m, 1H), 1.40 (d, J = 40.3 Hz, 1H), 1.13 (dd, J = 6.8, 3.8 Hz, 1H), 1.10 (d, J = 6.8 Hz, 2H), 1.05 – 1.00 (m, 2H), 0.98 (dd, J = 6.8, 3.9 Hz, 2H), 0.97 – 0.91 (m, 5H), 0.91 – 0.73 (m, 15H). ^{13}C NMR (151 MHz, CD3CN) δ 174.46, 170.07, 143.28, 143.11, 128.68, 128.50, 127.58, 127.14, 127.03, 82.73, 76.01, 61.46, 61.00, 59.92, 57.72, 54.53, 51.90, 51.39, 51.32, 50.31, 48.07, 46.91, 45.09, 44.81, 37.95, 31.32, 30.08, 26.37, 26.34, 26.28, 25.21, 25.12, 23.87, 19.46, 19.07, 18.56, 17.90, 17.83, 15.56, 14.71, 14.64, 14.58, 10.67.

Compound **9** was synthesized according to reported procedure.[6]

Synthesis of tri-tert-butyl (14S,18S)-3,11,16-trioxo-1-phenyl-2-oxa-4,10,15,17-tetraazaicosane-14,18,20-tricarboxylate, 10. Compounds **9** (2.02 g, 4.13 mmol, 1 equiv.) and HATU (1.26 g, 3.30 mmol, 0.8 equiv.) were dissolved in 10 mL DCM. To the mixture 2.37 mL DIPEA (13.63 mmol, 3.3 equiv.) was added to give yellow-colored solution. After stirring for 15 minutes, benzyl N-(5-aminoamyl)carbamate hydrochloride (1.13 g, 4.13 mmol, 1 equiv.) was added. The reaction mixture was stirred overnight. After completion, the excess solvent was removed by vacuum evaporation to give yellow oil crude product. The crude product was purified using automatic reverse-phase chromatography (0.1% TFA H_2O:MeCN gradient, 50 g HP C18 column (Method H). The pure fractions were collected and lyophilized to give light yellow oil product (839 mg, 1.19 mmol, 28.7%). ^{1}H NMR (600 MHz, MeOD) δ 7.43 – 7.17 (m, 5H), 5.06 (s, 2H), 4.33 – 4.02 (m, 3H), 3.57 – 3.49 (m, 1H), 3.47 (t, J = 5.5 Hz, 1H), 3.31 (p, J = 1.6 Hz, 1H), 3.20 – 3.06 (m, 4H), 2.15 – 1.99 (m, 4H), 1.92 – 1.75 (m, 4H), 1.71 – 1.54 (m, 3H), 1.47 – 1.43 (m, 34H).

Synthesis of di-tert-butyl (((S)-5-((5-aminopentyl)amino)-1-(tert-butoxy)-1,5-dioxopentan-2-yl)carbamoyl)-L-glutamate, 11. To a suspension of palladium on carbon (100 mg) (methano10 mL) compound 17 (775 mg, 1.10 mmol) was slowly added under N_2. Ballons containing H_2 gas were connected to the reaction vessel, and the reaction mixture was stirred for 4 hours. Upon completion, the palladium on carbon was filtered, and excess solvent was removed by vacuum evaporation. The crude material was purified using semi-preparative HPLC (Method G) (103 mg, 0.18 mmol, 16.4%).

Synthesis of NOTA-Bn-Pent-EuE, 12. Compound **11** (60.0 mg, 0.10 mmol, 1 equiv.) was dissolved in 1 mL DCM. To the solution p-SCN-Bn-NOTA (51.9 mg, 0.12 mmol, 1.1 equiv.) and DIPEA (73 µL, 0.42 mmol, 4 equiv.) were added. The reaction mixture was allowed to stir overnight. The excess solvent was removed by vacuum evaporation to give white, waxy product. The material was used for the next step without purification.

Synthesis of tert-butyl protected PD2 conjugate, 13. Compound **19** (50.2 mg, 0.050 mmol, 1.5 equiv.) and HATU (31.1 mg, 0.082 mmol, 2.5 equiv.) were dissolved in 500 µL DMF. Following the addition of DIPEA (46 µL, 0.26 mmol, 8 equiv.), the solution turned yellow. After stirring for 15 minutes, MMAE (23.5 mg, 0.033 mmol, 1 equiv.) was added, and the reaction was allowed to stir for 2 hours. The solvent was removed by a stream of N_2. The crude material was purified using semi-preparative HPLC (Method G) to give a white powdered product.

Synthesis of PD2. Compound **20** was dissolved in 1 mL 8:2 TFA:DCM. The reaction mixture was stirred for 2 hours. The solvent was evaporated by a stream of N_2. The crude material was purified using analytical HPLC (Method A).

Synthesis of 1-benzyl 7-(2,5-dioxopyrrolidin-1-yl) heptanedioate, 15. Compound **15** was synthesized using a modified procedure previously in the literature.[7] To a solution of **14** (500 mg, 2.00 mmol) in chloroform (20 mL) was added N-succinimidyl carbonate (9.21 mg, 3.60 mmol) and DIPEA (696 µL, 4.00 mmol). The resulting mixture was stirred at room temperature under N_2 atmosphere for 20 hours. The crude material was washed with DI water twice and dried over Na_2SO_4. The solvent was removed under reduced pressue to yield an oil. The final NHS ester compound **15** was purified using silica gel flash chromatography using a gradient of EtOAc (0 – 100%) in hexane to give a colorless oil (515 mg, 1.48 mmol, 74%). ^{1}H NMR (500 MHz, $CDCl_3$) δ 7.40 – 7.28 (m, 5H), 5.12 (s, 2H), 2.83 (s, 4H), 2.59 (t, J = 7.4 Hz, 2H), 2.38 (t, J = 7.4 Hz, 2H), 1.76 (p, J = 7.5 Hz, 2H), 1.69 (p, J = 7.5 Hz, 2H), 1.49 – 1.37 (m, 2H). ^{13}C NMR (126 MHz, $CDCl_3$) δ 173.24, 169.10, 168.45, 136.07, 128.57, 128.23, 128.21, 66.19, 33.96, 30.77, 28.20, 25.60, 24.40, 24.25.

Compound **16** was synthesized according to published procedure.[8]

Synthesis of di-tert-butyl (((S)-6-(7-(benzyloxy)-7-oxoheptanamido)-1-(tert-butoxy)-1-oxohexan-2-yl)carbamoyl)-L-glutamate, 17. Compounds **15** (158 mg, 455 µmol) and **16** (222 mg, 455 µmol) were mixed in chloroform (20 mL) and stirred at room temperature for 15 minutes. Then, DIPEA (80 µL, 0.46 mmol) was added to the solution. The crude reaction was stirred overnight while heating at 60 ºC. Volatiles were removed under reduced pressure and the resulting residue purified via C18 RP flash chromatography using a gradient of acetonitrile (0-100%) in water with 0.1% FA as additive in both phases. Compound **17** was then recoverd as a colorless oil (246 mg, 342 µmol, 75%). ^{1}H NMR (500 MHz, $CDCl_3$) δ 7.37 – 7.30 (m, 5H), 6.09 (s, 1H), 5.40 (t, J = 7.9 Hz, 2H), 5.12 (s, 1H), 4.35 – 4.26 (m, 2H), 3.35 – 3.28 (m, 1H), 3.21 – 3.08 (m, 1H), 2.43 – 2.24 (m, 4H), 2.19 (t, J = 7.4 Hz, 2H), 2.11 – 2.03 (m, 1H), 1.92 – 1.75 (m, 2H), 1.72 – 1.58 (m, 4H), 1.51 (t, J = 7.0 Hz, 2H), 1.46 (s, 9H), 1.45 (s, 9H), 1.43 (s, 9H), 1.36 (p, J = 7.2 Hz, 4H). ^{13}C NMR (126 MHz, CDCl3) δ 173.84, 173.51, 172.48, 172.40, 172.34, 157.27, 136.00, 128.57, 128.50, 128.21, 128.17, 82.16, 81.82, 80.65, 66.27, 53.21, 38.94, 36.20, 34.19, 32.43, 31.65, 28.66, 28.57, 28.09, 28.02, 25.34, 24.55, 22.17.

Synthesis of (7S,11S)-7,11-bis(tert-butoxycarbonyl)-2,2-dimethyl-4,9,17-trioxo-3-oxa-8,10,16-triazatricosan-23-oic acid, 18. To compound 17 (130 mg, 181 µmol) dissolved in MeOH (4 mL) was added 10% Pd/C (21.8 mg, 205 µmol). The resulting crude material was hydrogenated in a Parr pressured vessel at ~30 psi and at room temperature for 4 hours. The crude was then filtered through celite and the solvent removed under reduced pressure to yield an oil (yield: 97.2 mg, 154 µmol, 85%). ^{1}H NMR (500 MHz, $CDCl_3$) δ 6.36 (s, 1H), 5.99 (s, 1H), 5.71 (d, *J* = 8.3 Hz, 1H), 4.40 – 4.24 (m, 2H), 3.43 – 3.34 (m, 1H), 3.13 – 3.01 (m, 1H), 2.46 – 2.17 (m, 5H), 2.13 – 2.01 (m, 1H), 1.91 – 1.77 (m, 2H), 1.76 – 1.17 (m, 39H). ^{13}C NMR (126 MHz, $CDCl_3$) δ 173.52, 172.99, 172.83, 157.70, 82.19, 82.10, 80.94, 53.43, 53.27, 38.77, 36.11, 33.90, 32.15, 31.84, 28.51, 28.07, 28.02, 28.00, 27.93, 27.88, 25.28, 24.17, 21.81.

Synthesis of tert-butyl protected PD3 conjugate, 19. Compounds **18** (15.5 mg, 24.6 µmol, 1.5 equiv.) and **Val-Ala-MMAE** (17.0 mg, 16.4 µmol, 1 equiv.) were mixed in DMF (1 mL). To the solution DIPEA (11.4 µL, 65.6 µmol, 4 equiv.) and HATU (7.8 mg, 20.5 µmol, 1.25 equiv.) were added. The reaction was stirred overnight at room temperature. The solvent was removed by a stream of N_2 gas. The crude material was purified using semi-preparative HPLC (Method E) to give a white powder.

Synthesis of PD3 conjugate. Compound **19** was dissolved in 1 mL 8:2 TFA:DCM. The reaction mixture was stirred for 2 hours. The solvent was removed by a stream of N_2. The crude material was purified using analytical HPLC (Method A).

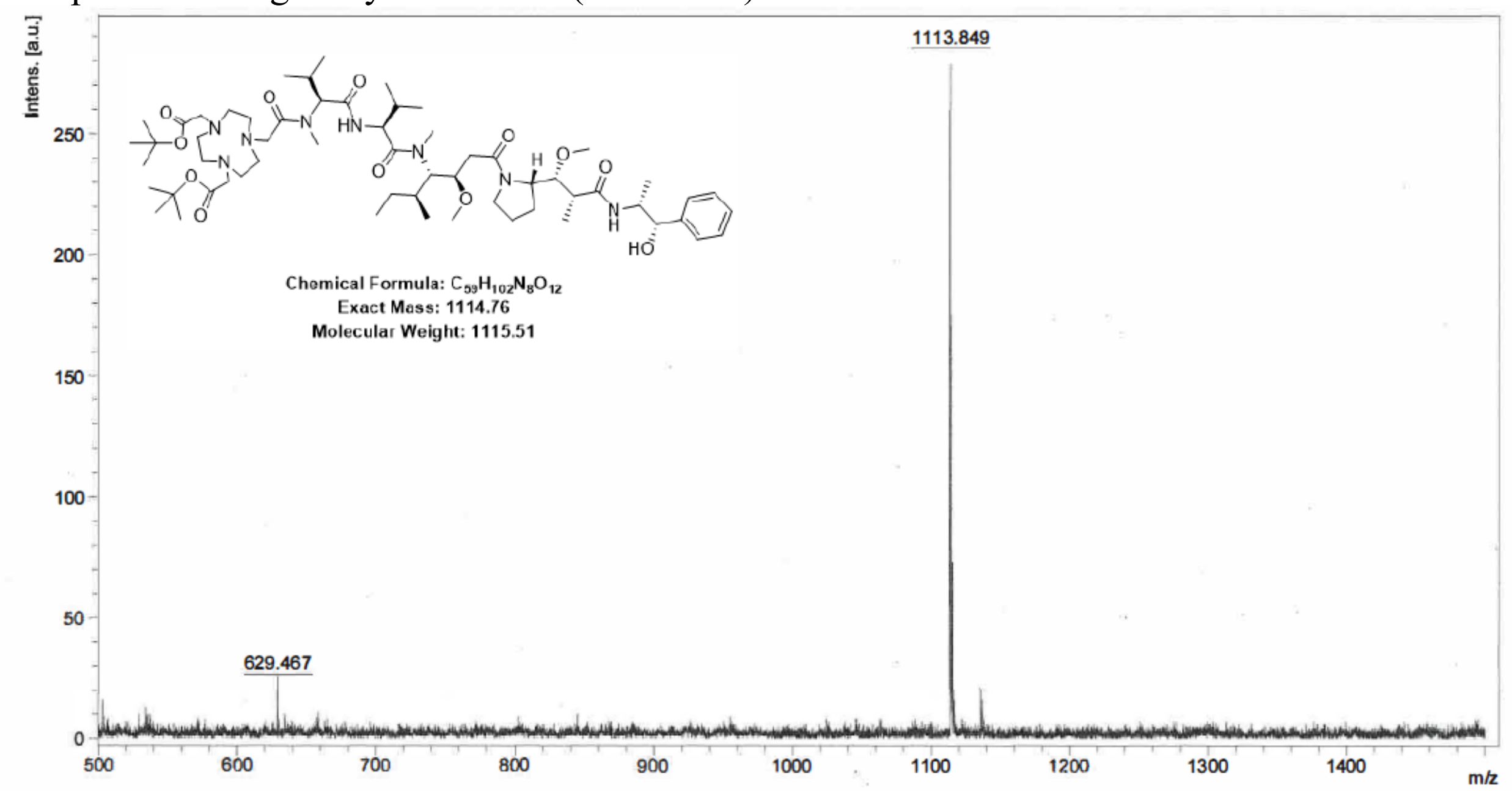

Figure S117. MALDI for **8**.

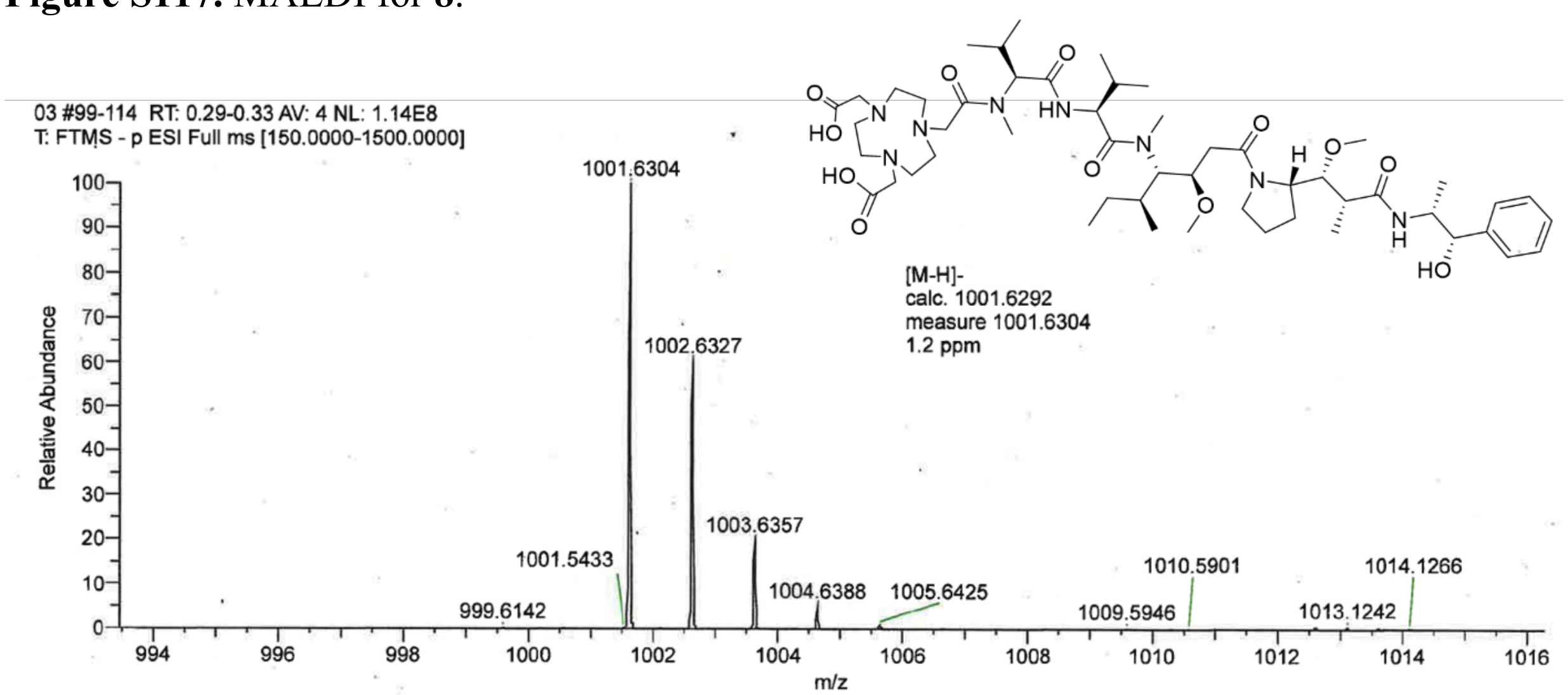

Figure S118. HRESI-MS of PD1. HRMS calc. for C51H86N8O12 1002.6365. Found 1001.6292 $[M-H]^-$.

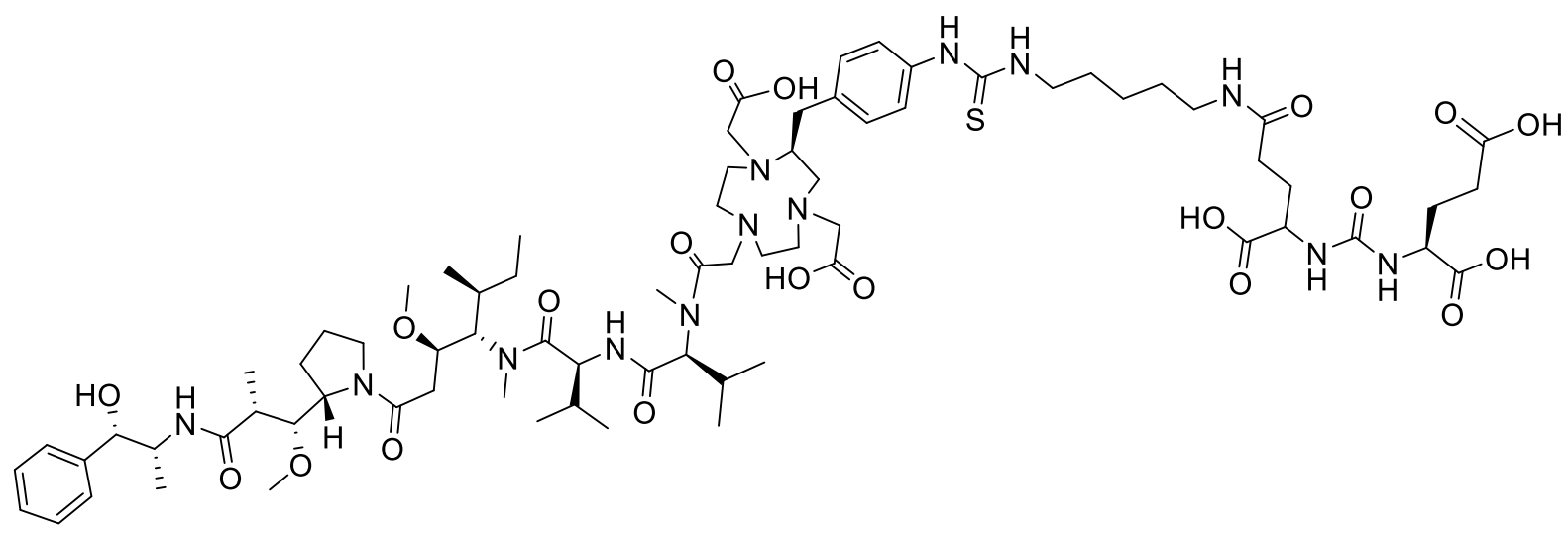

Chemical Formula: $C_{75}H_{119}N_{13}O_{20}S$
Exact Mass: 1553.84
Molecular Weight: 1554.91

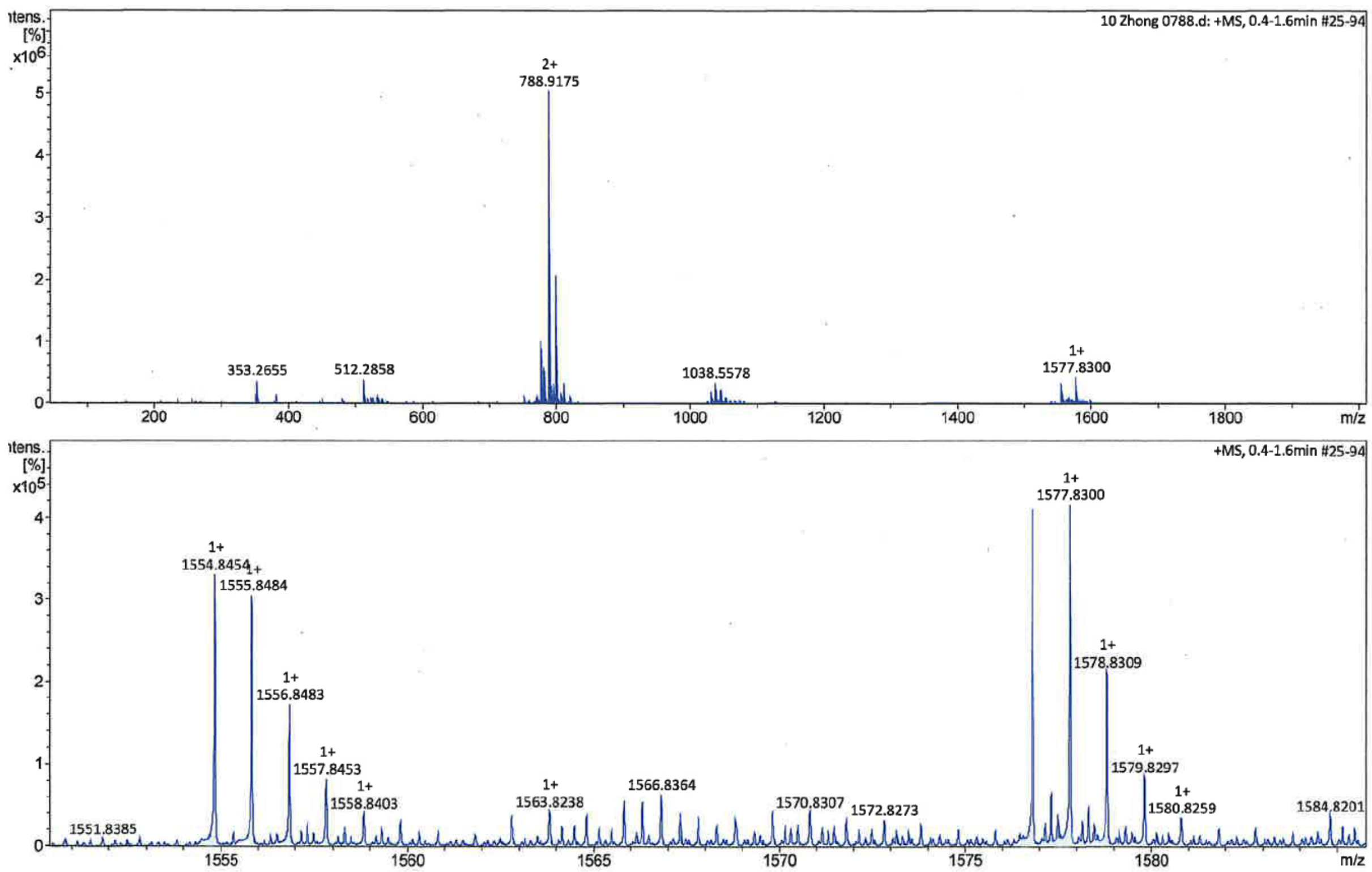

Figure S119. HRESI-MS of PD2. HRESI calc. for $C_{75}H_{119}N_{13}O_{20}S$ 1553.8415. Found 1554.8454 $[M+H]^+$.

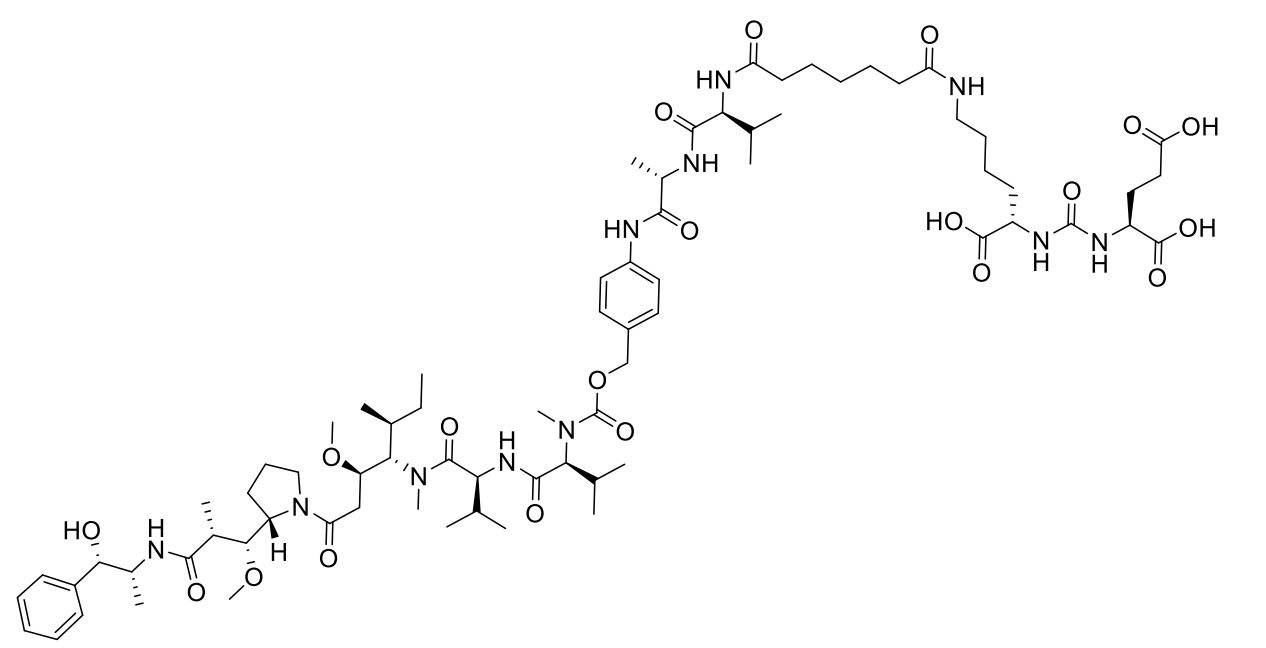

Chemical formula: $C_{74}H_{117}N_{11}O_{20}$
Exact mass: 1479.85
Molecular weight: 1480.81

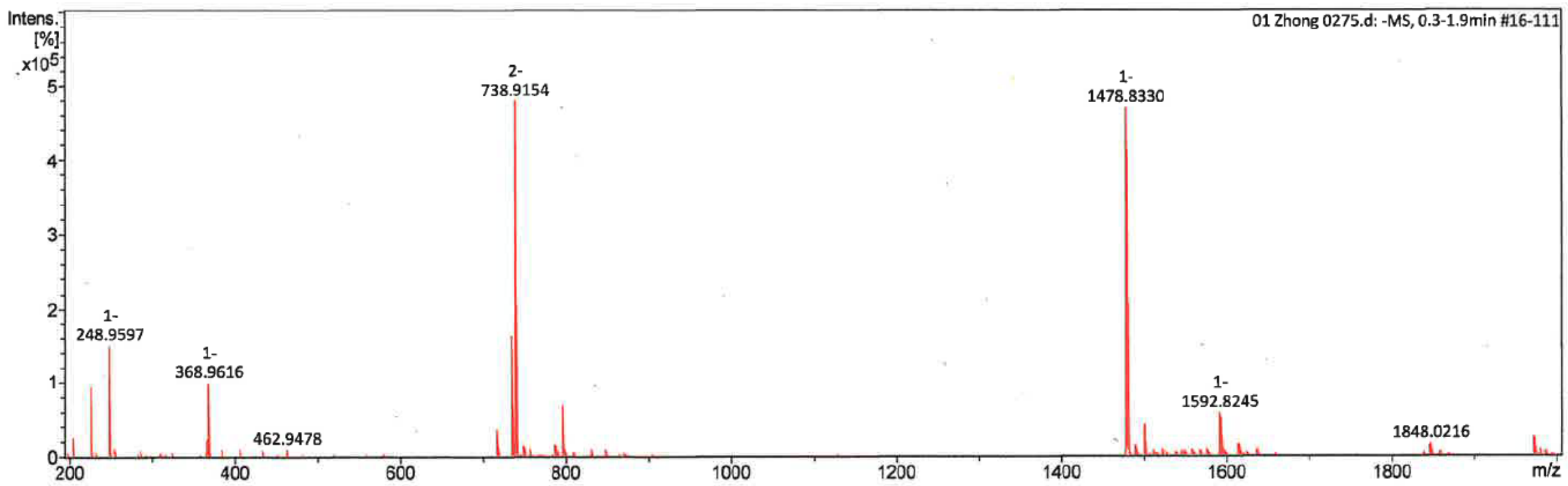

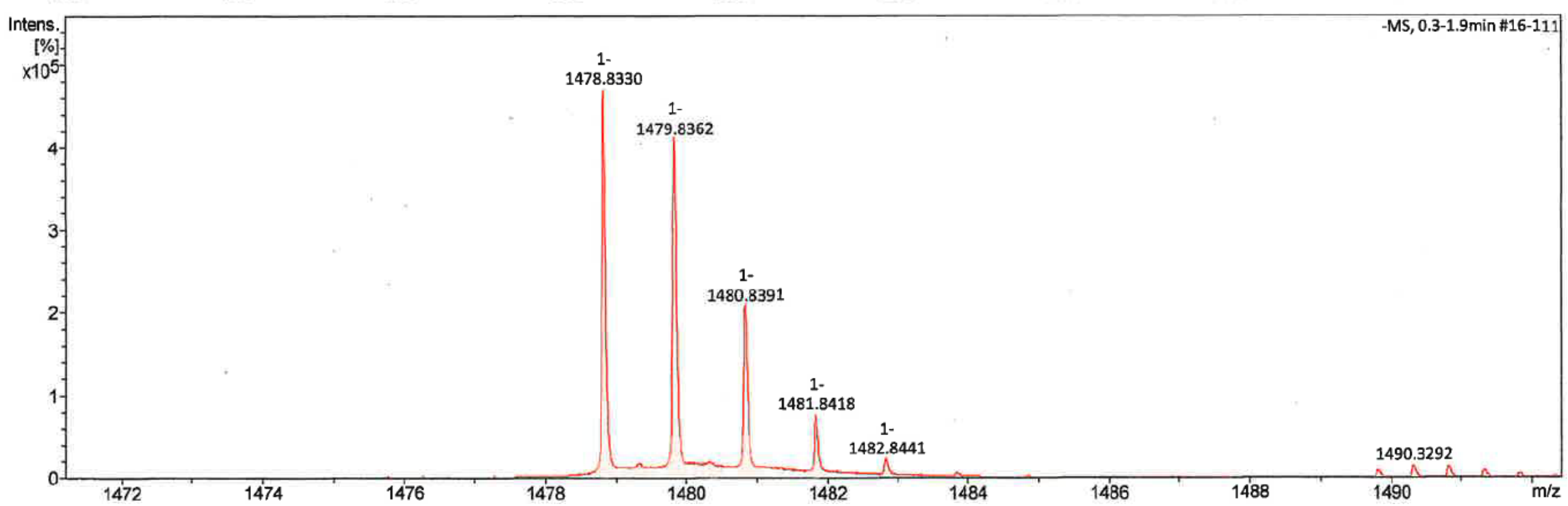

Figure S120. HRESI-MS of PD3. HRESI calc. for $C_{74}H_{117}N_{11}O_{20}$ 1479.8476. Found 1478.8330 $[M\text{-}H]^{-}$.

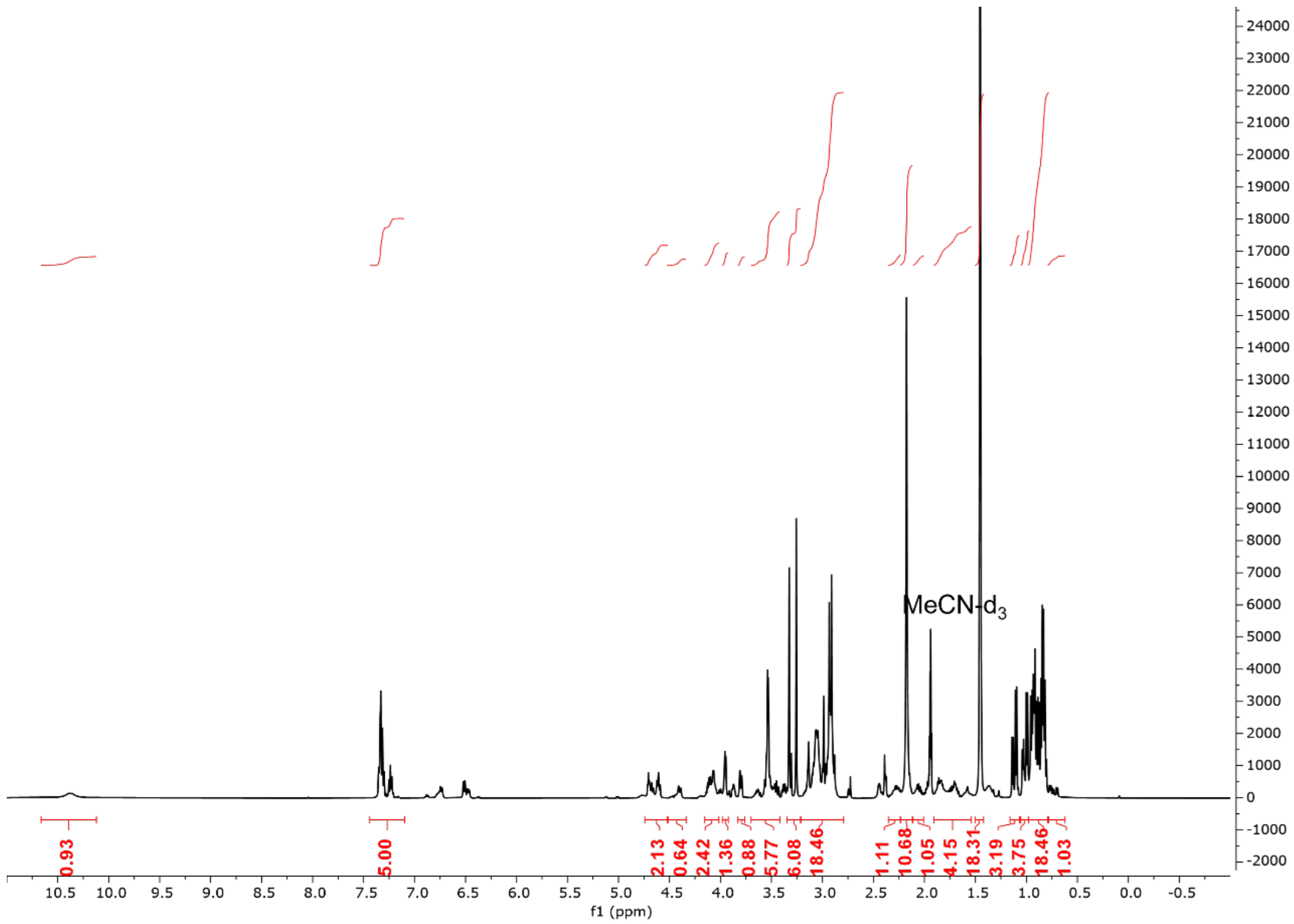

Figure S121. ^{1}H NMR (500 MHz, MeCN-d$_3$) for 8.

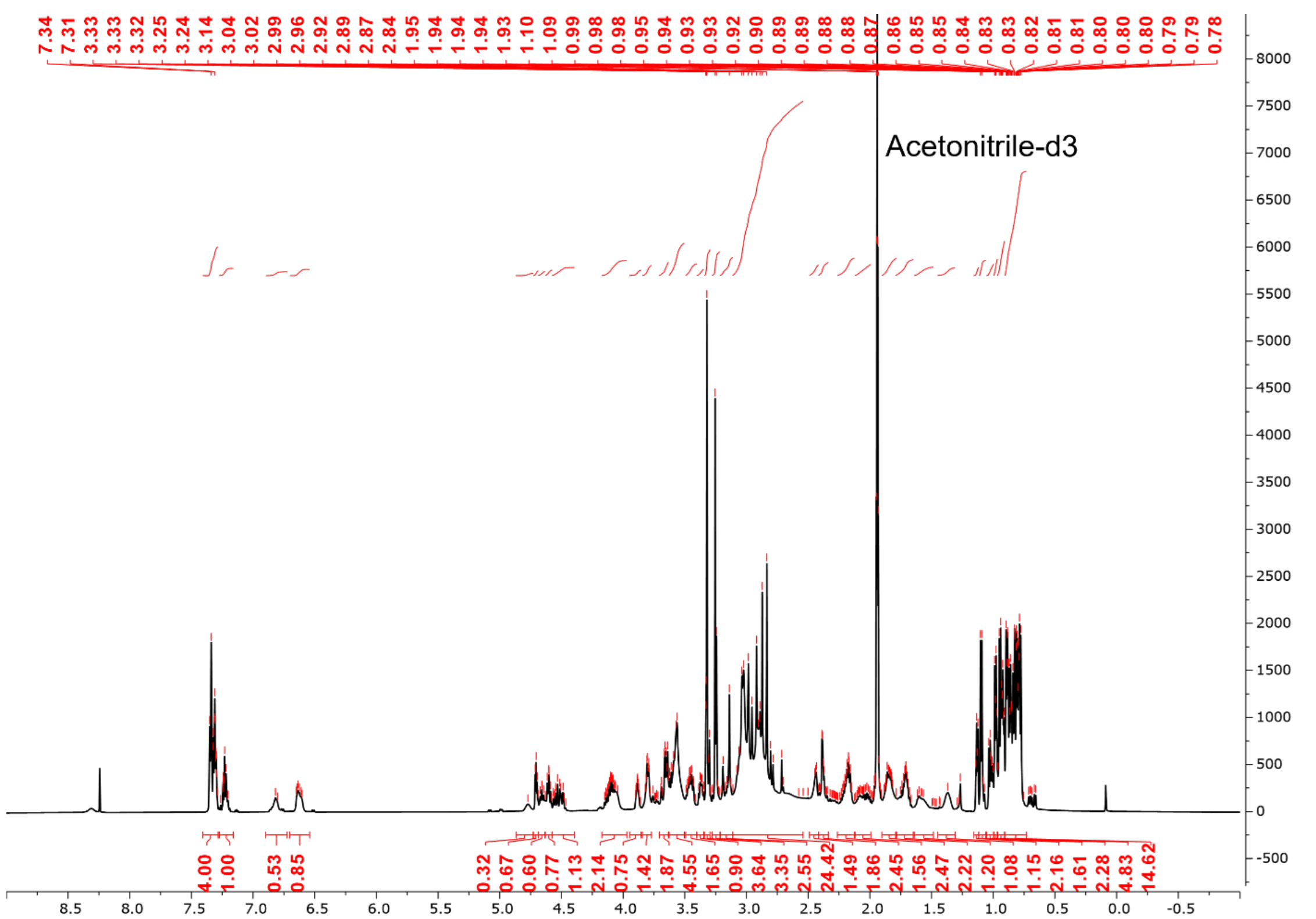

Figure S122. ^{1}H NMR (600 MHz, MeCN-d$_3$) for PD1.

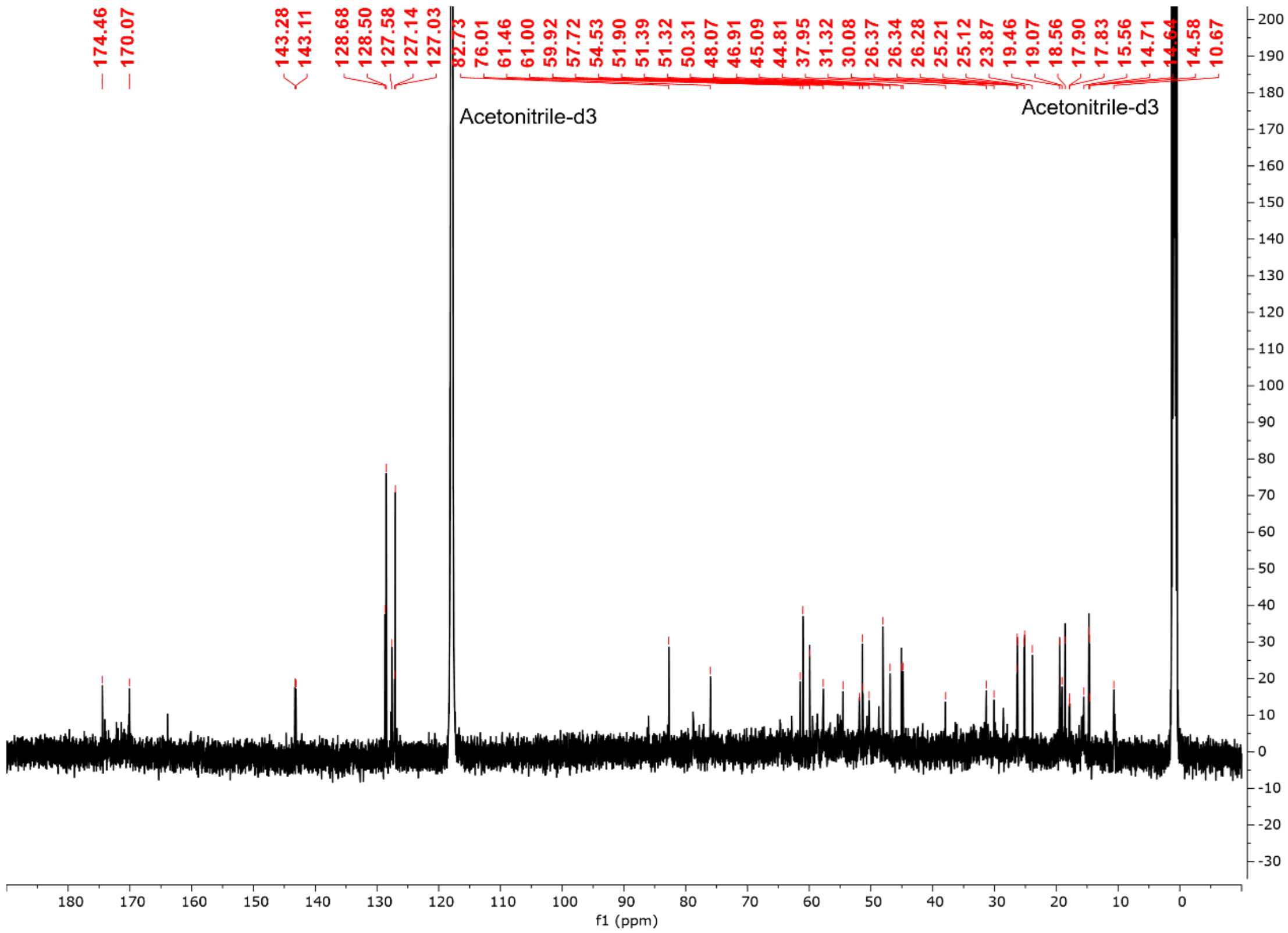

Figure S123. ^{13}C NMR (600 MHz, MeCN-d_3) for PD1.

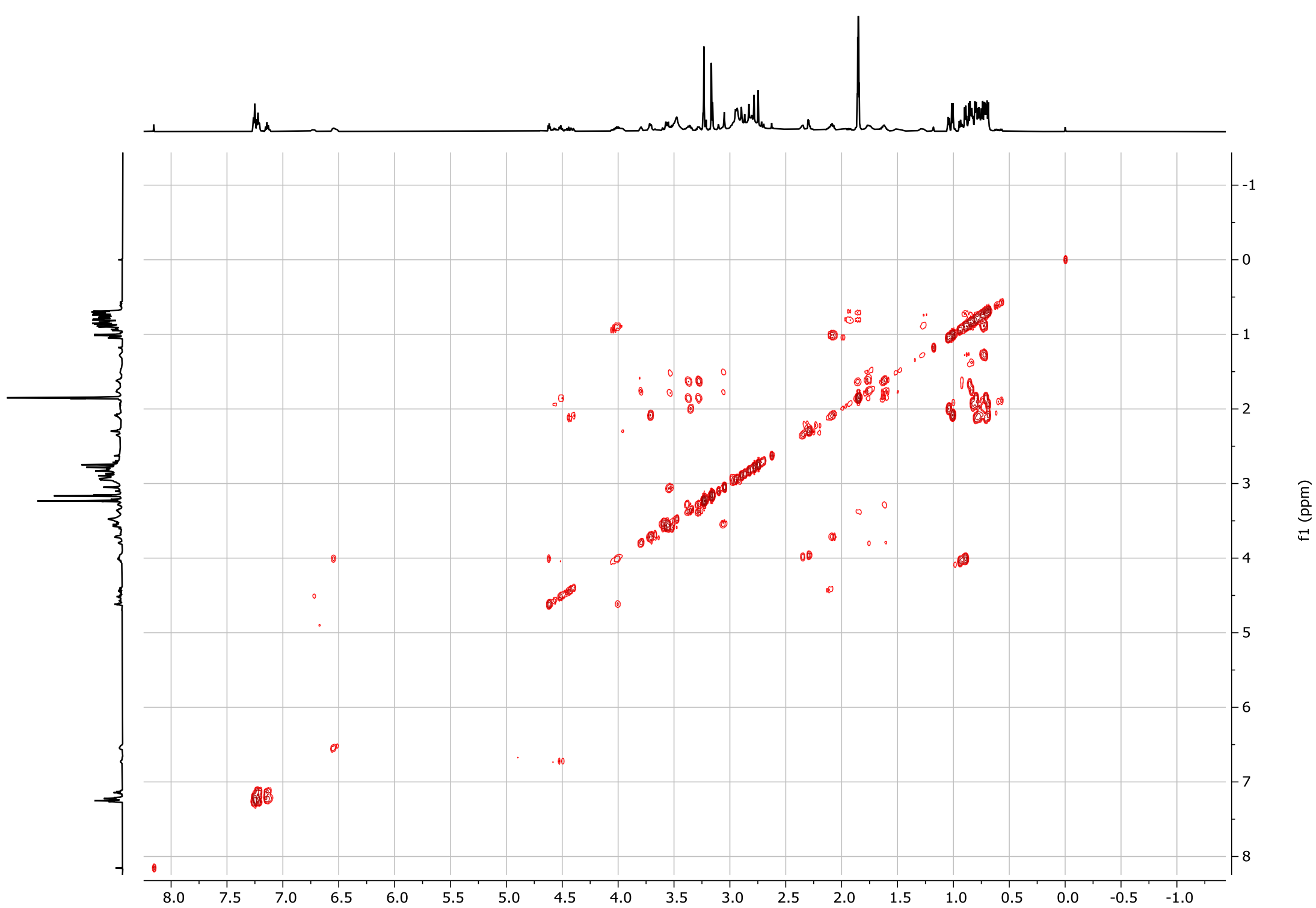

Figure S124. The ^{1}H-^{1}H COSY NMR spectrum for PD1.

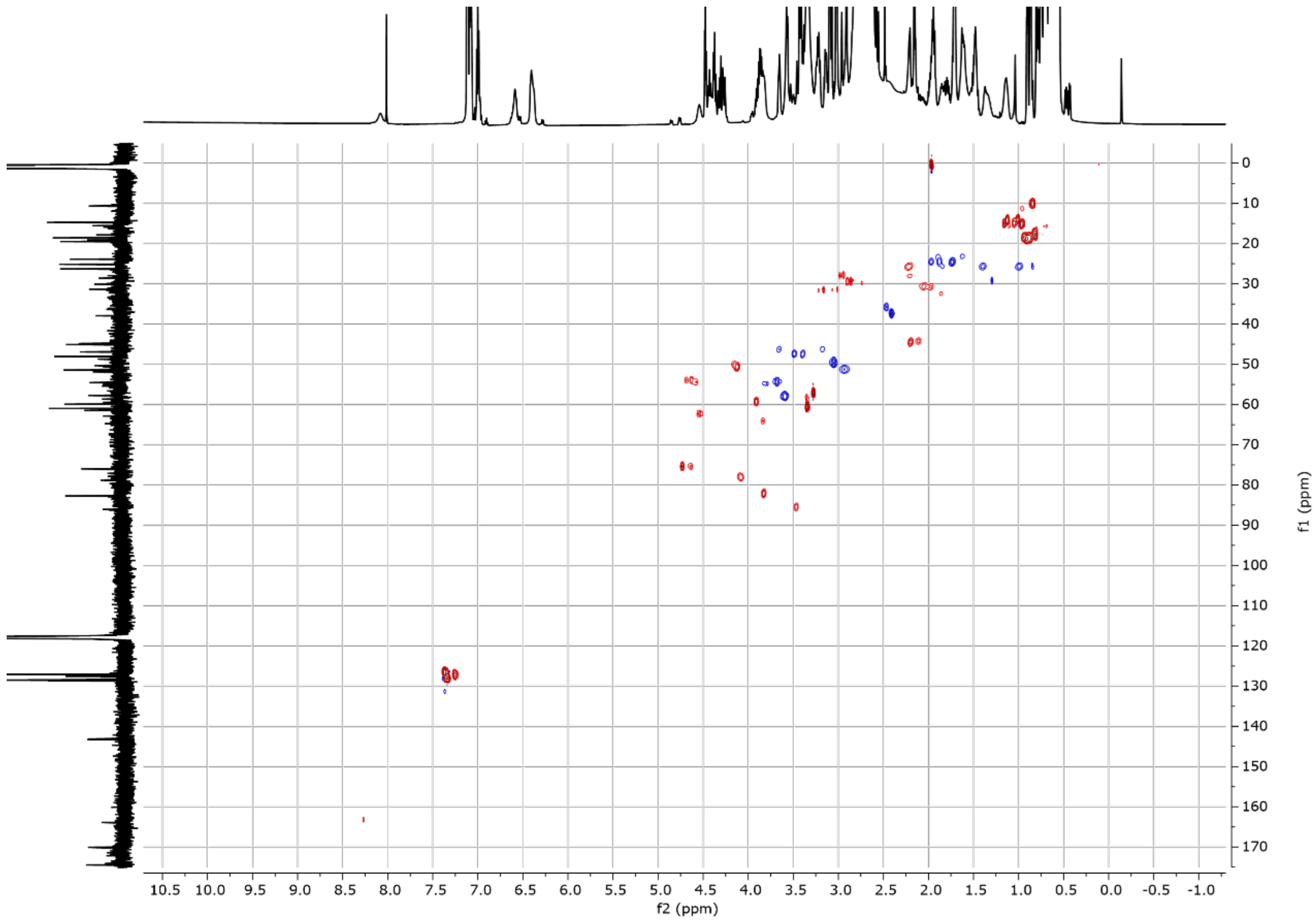

Figure S125. The ^{1}H-^{13}C HSQC NMR spectrum for PD1. Edited HSQC method: CH + CH_3 peak shows positive, and CH_2 peak shows negative.

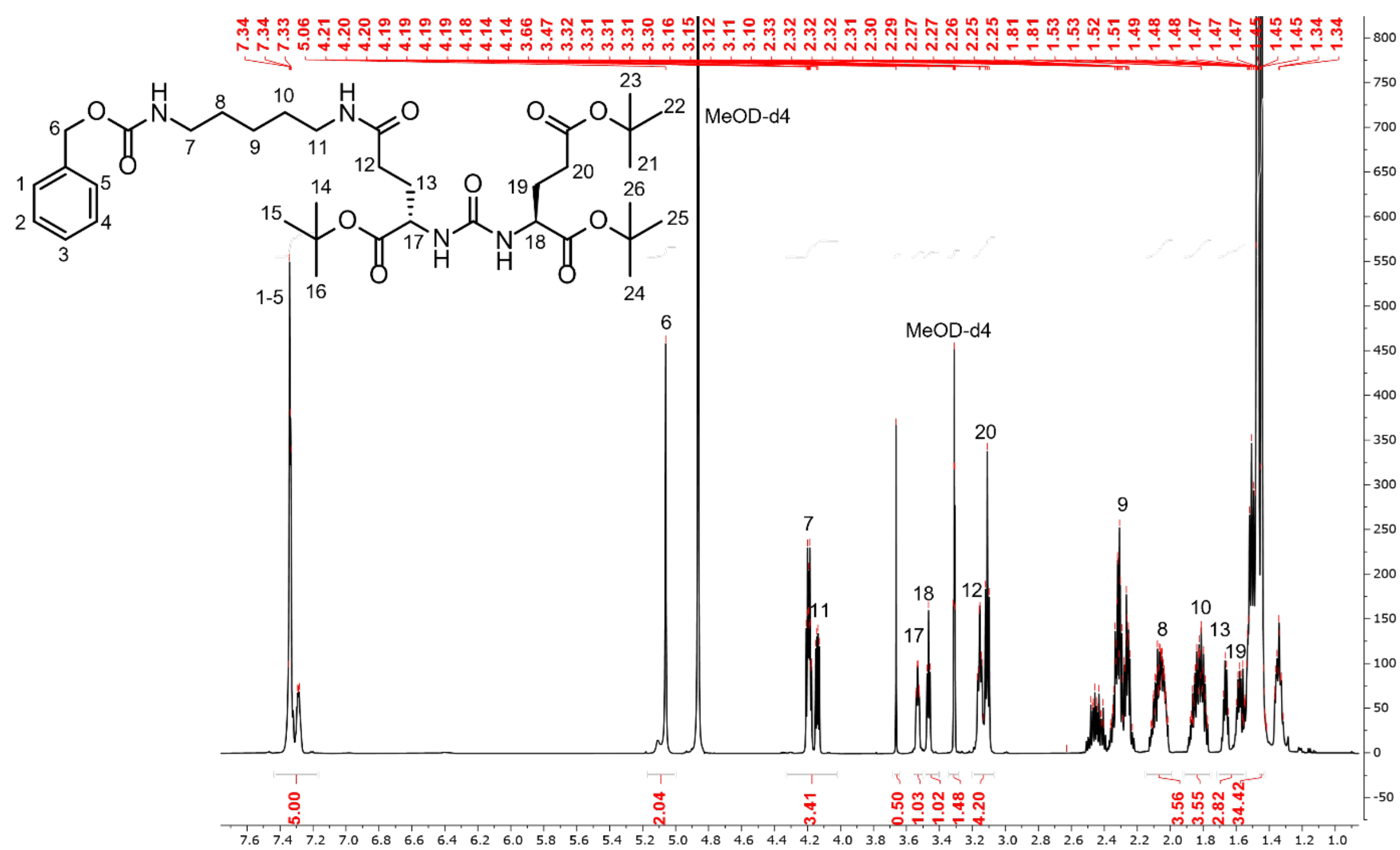

Figure S126. ^{1}H NMR (600 MHz, MeOD-d_4) for 10.

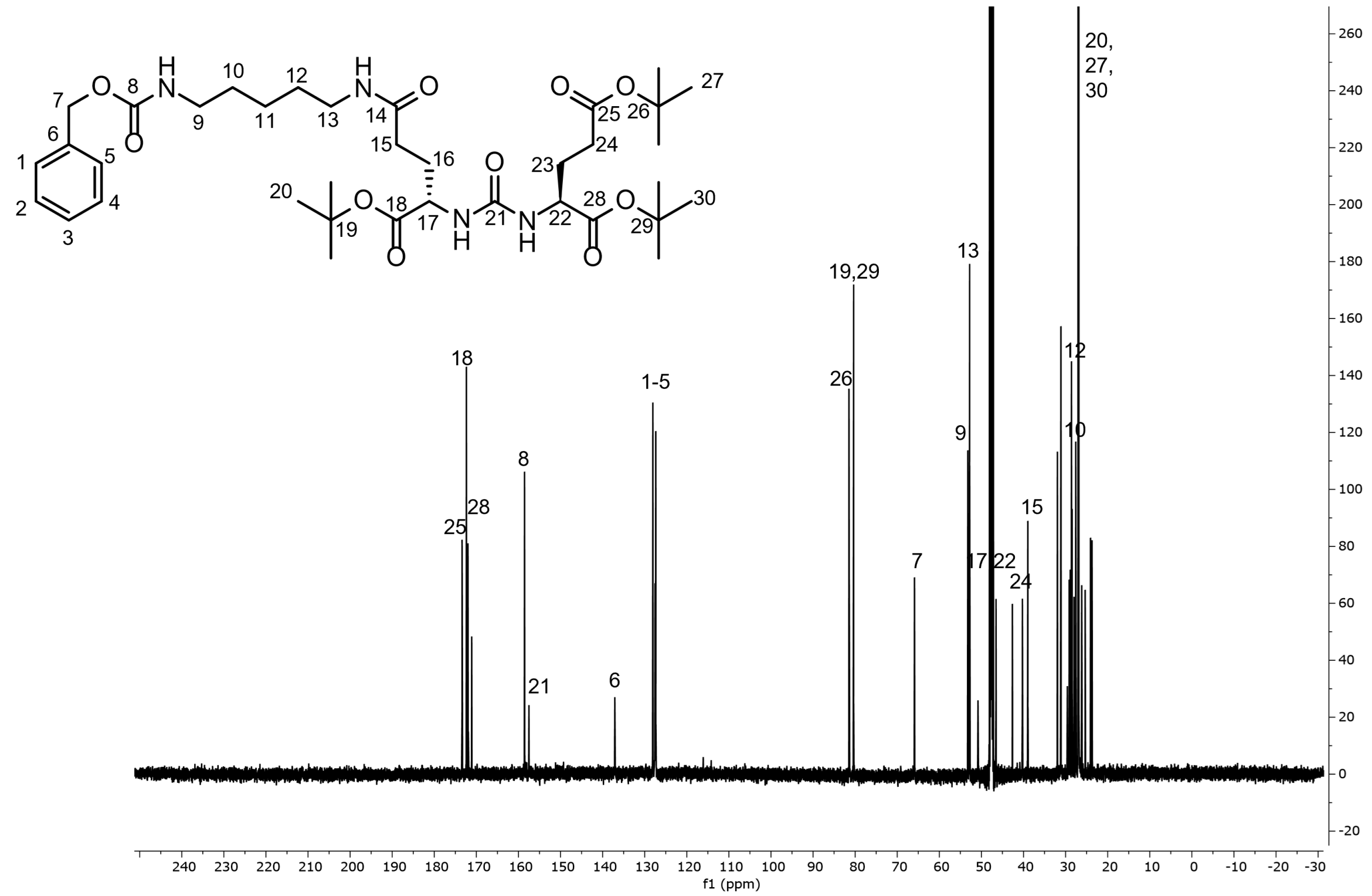

Figure S127. ^{13}C NMR (600 MHz, MeOD-d$_4$) for 10.

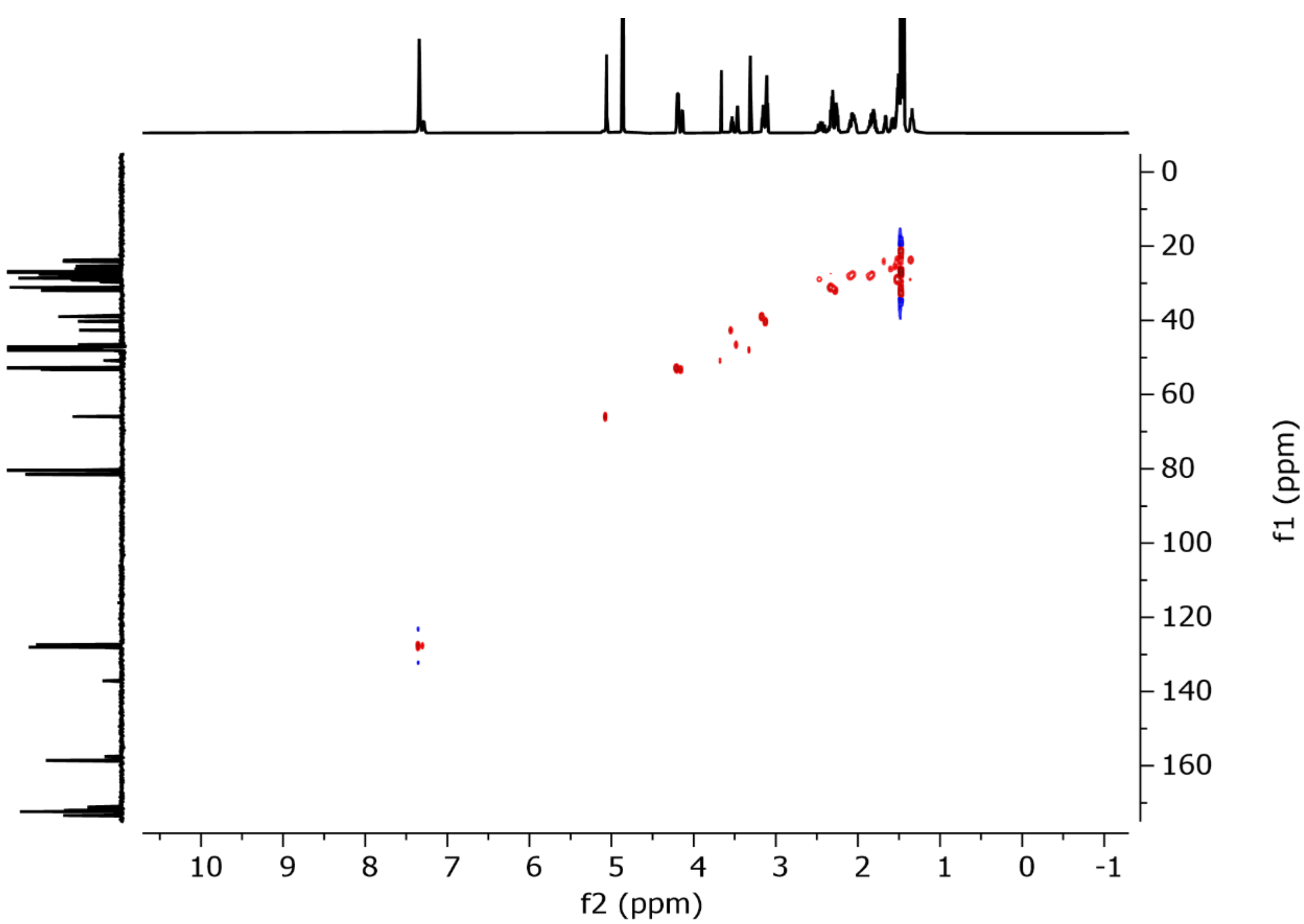

Figure S128. The ^{1}H-^{13}C HSQC NMR spectrum for 10.

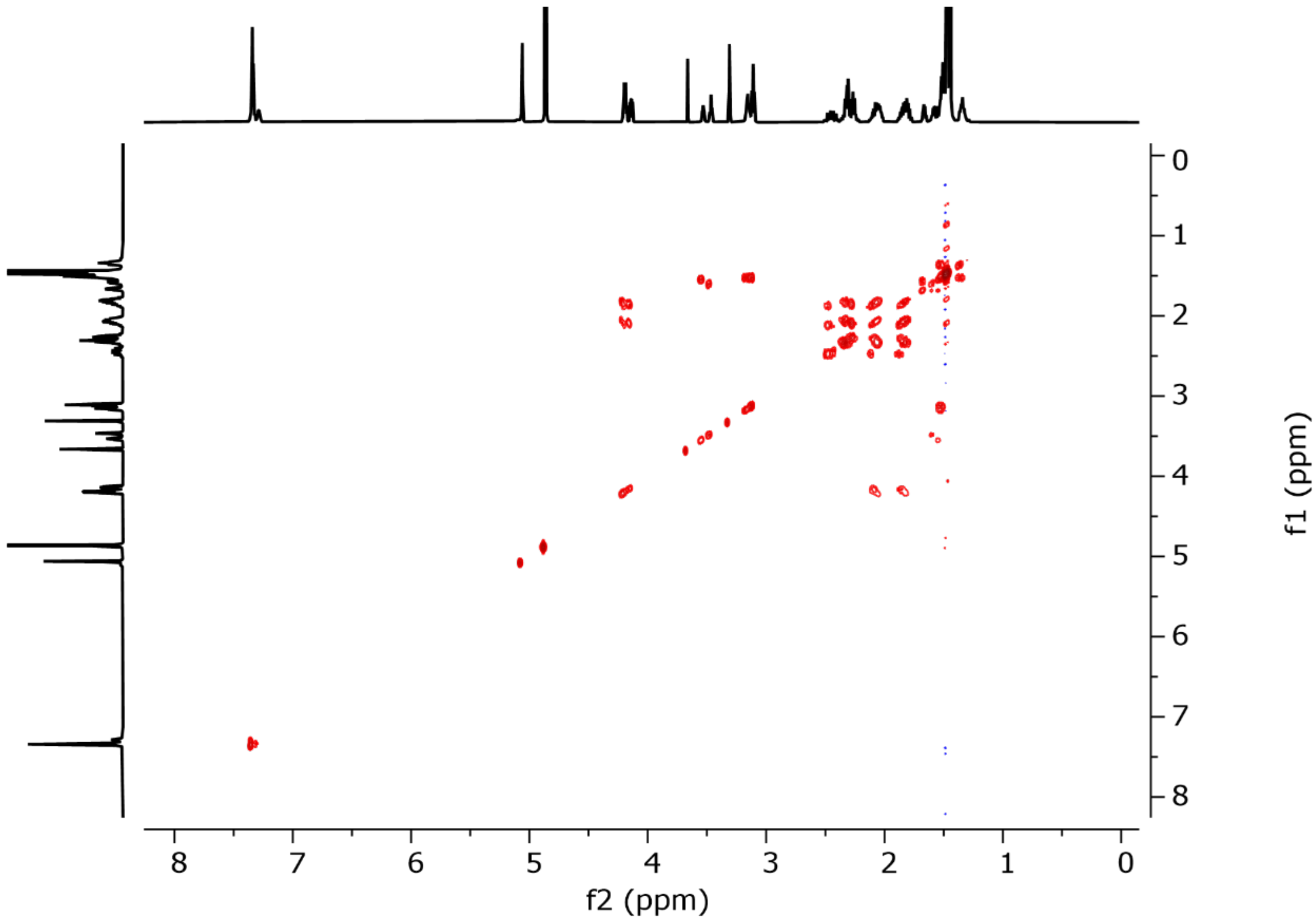

Figure S129. The ^{1}H-^{1}H COSY NMR spectrum for 10.

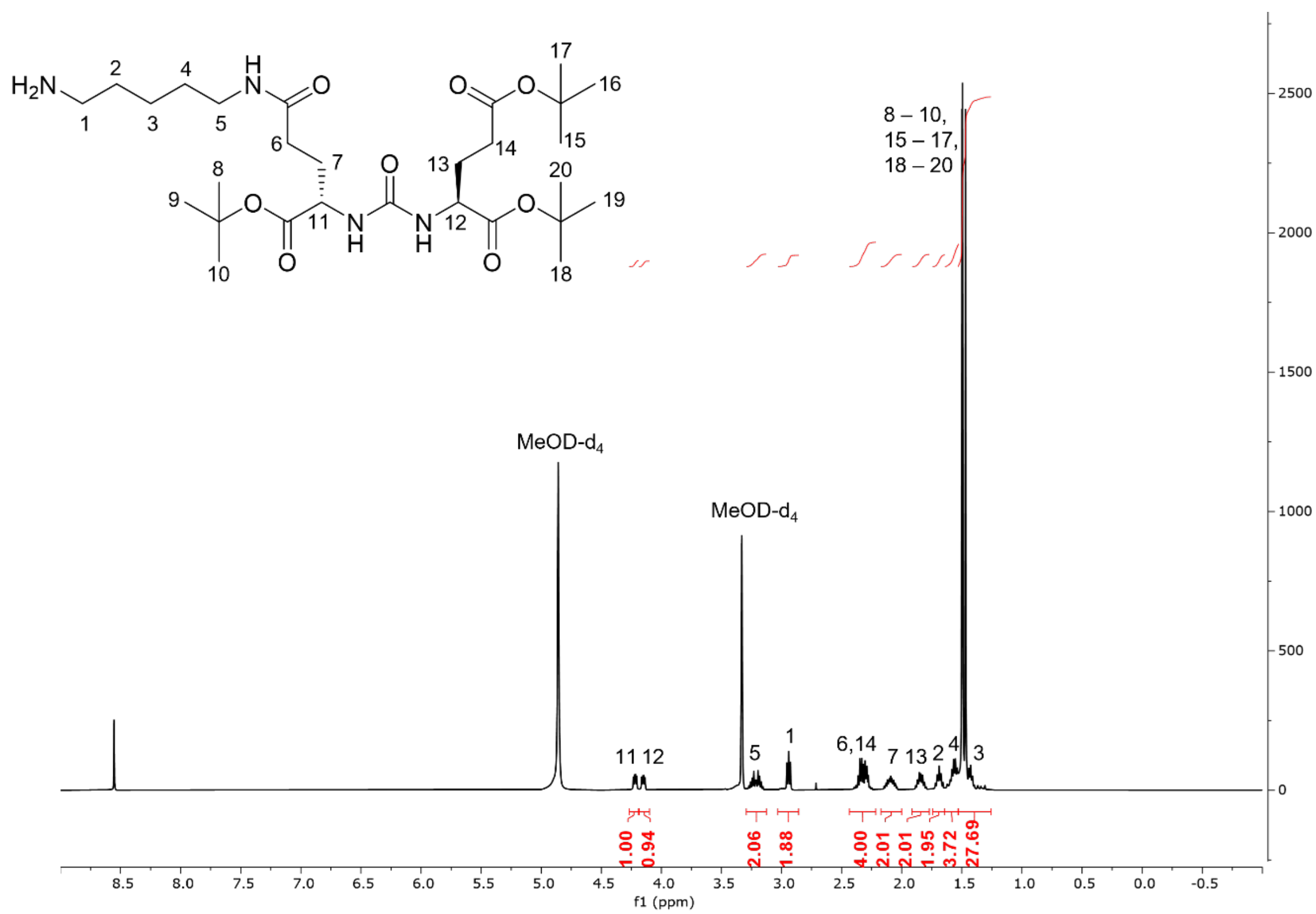

Figure S130. ^{1}H NMR (500 MHz, MeOD-d_4) for 11.

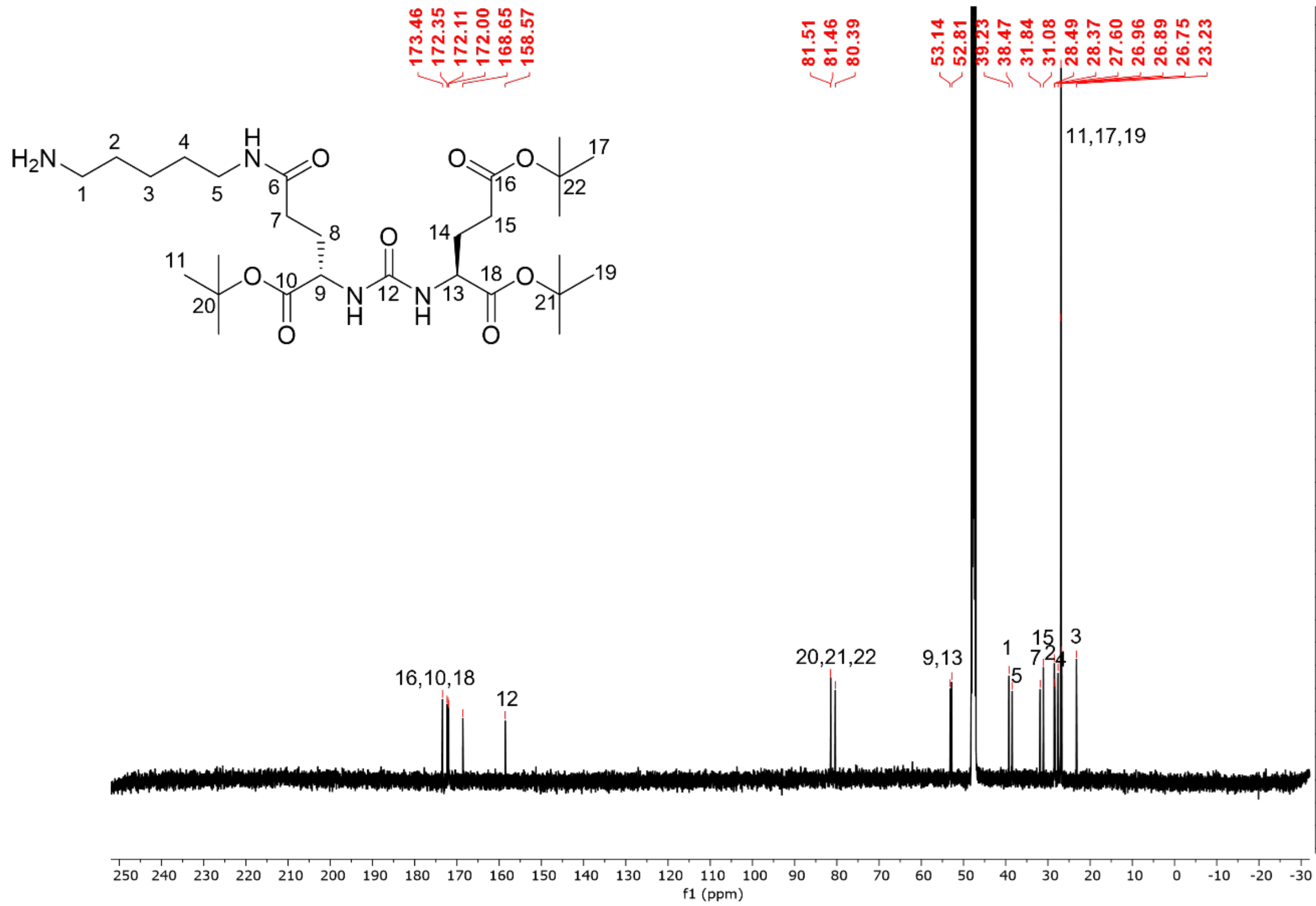

Figure S131. ^{13}C NMR (600 MHz, MeOD-d_4) for 11.

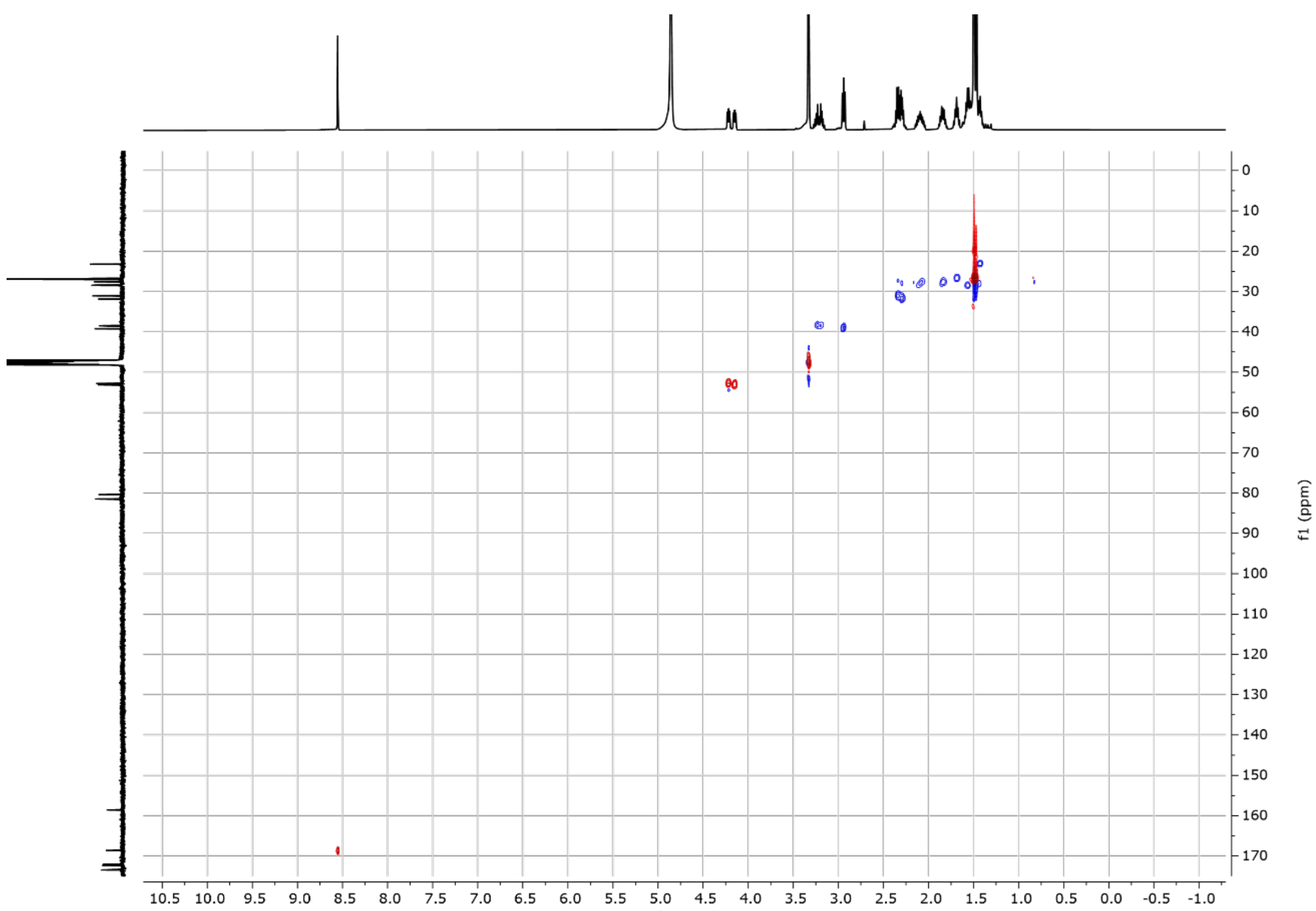

Figure S132. The ^{1}H-^{13}C HSQC NMR spectrum for 11. Edited HSQC method: CH + CH_3 peak shows positive, and CH_2 peak shows negative.

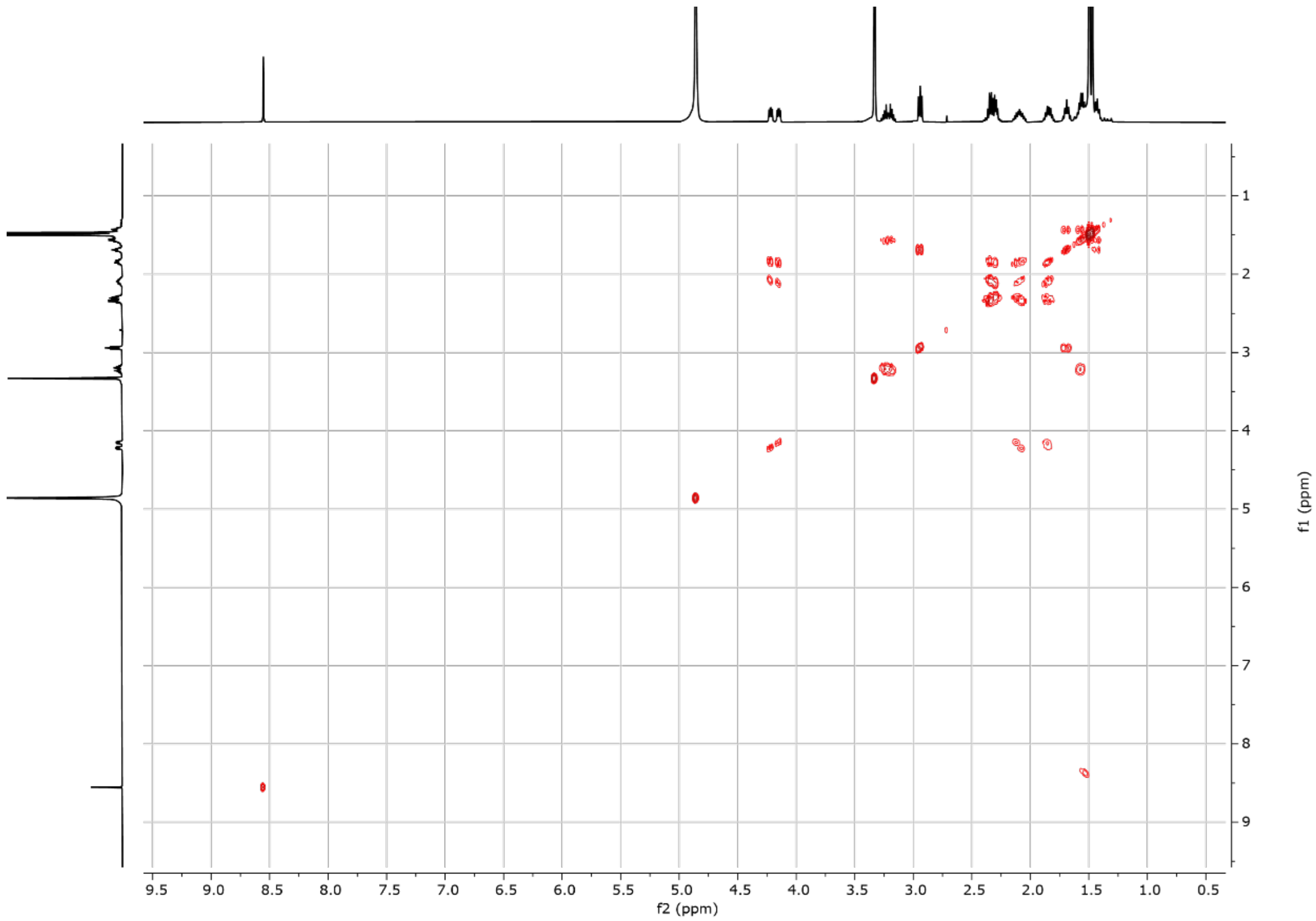

Figure S133. The ^{1}H-^{1}H COSY NMR spectrum for 11.

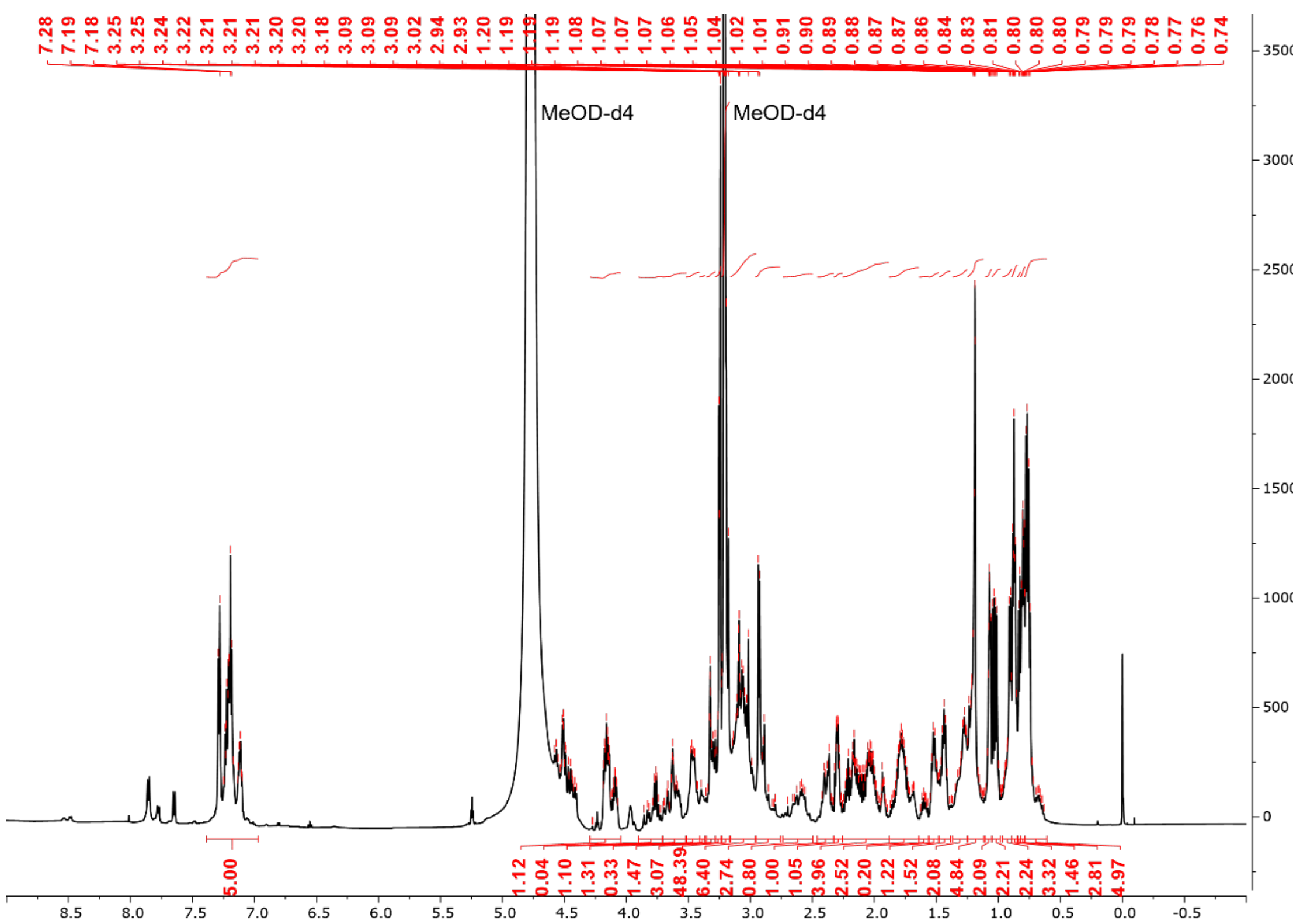

Figure S134. ^{1}H NMR (600 MHz, MeCN-d_6) for PD2.

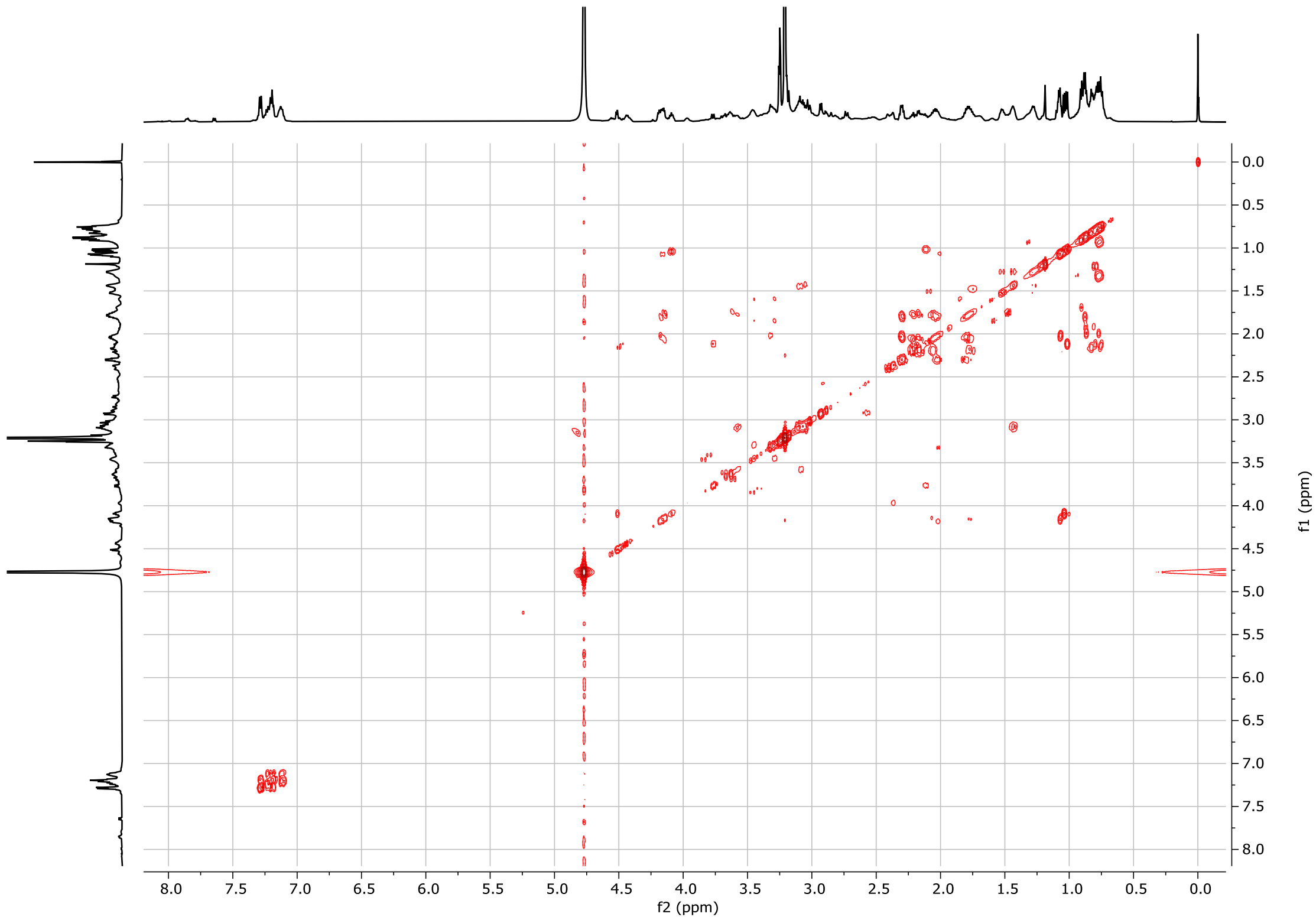

Figure S135. The ^{1}H-^{1}H COSY NMR spectrum for PD2.

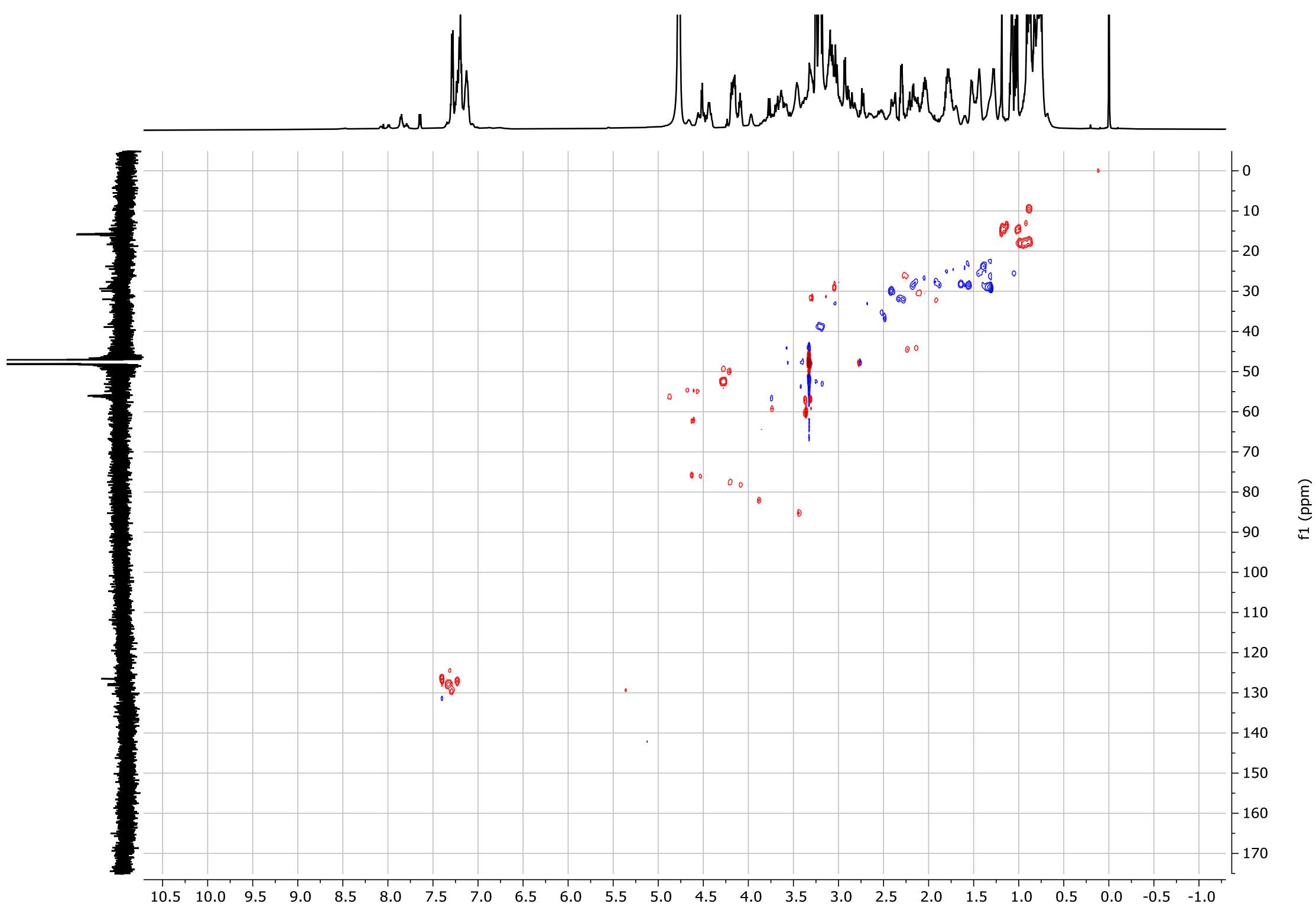

Figure S136. The ^{1}H-^{13}C HSQC NMR spectrum for PD2. Edited HSQC method: CH + CH_3 peaks show positive, and CH_2 peak shows negative.

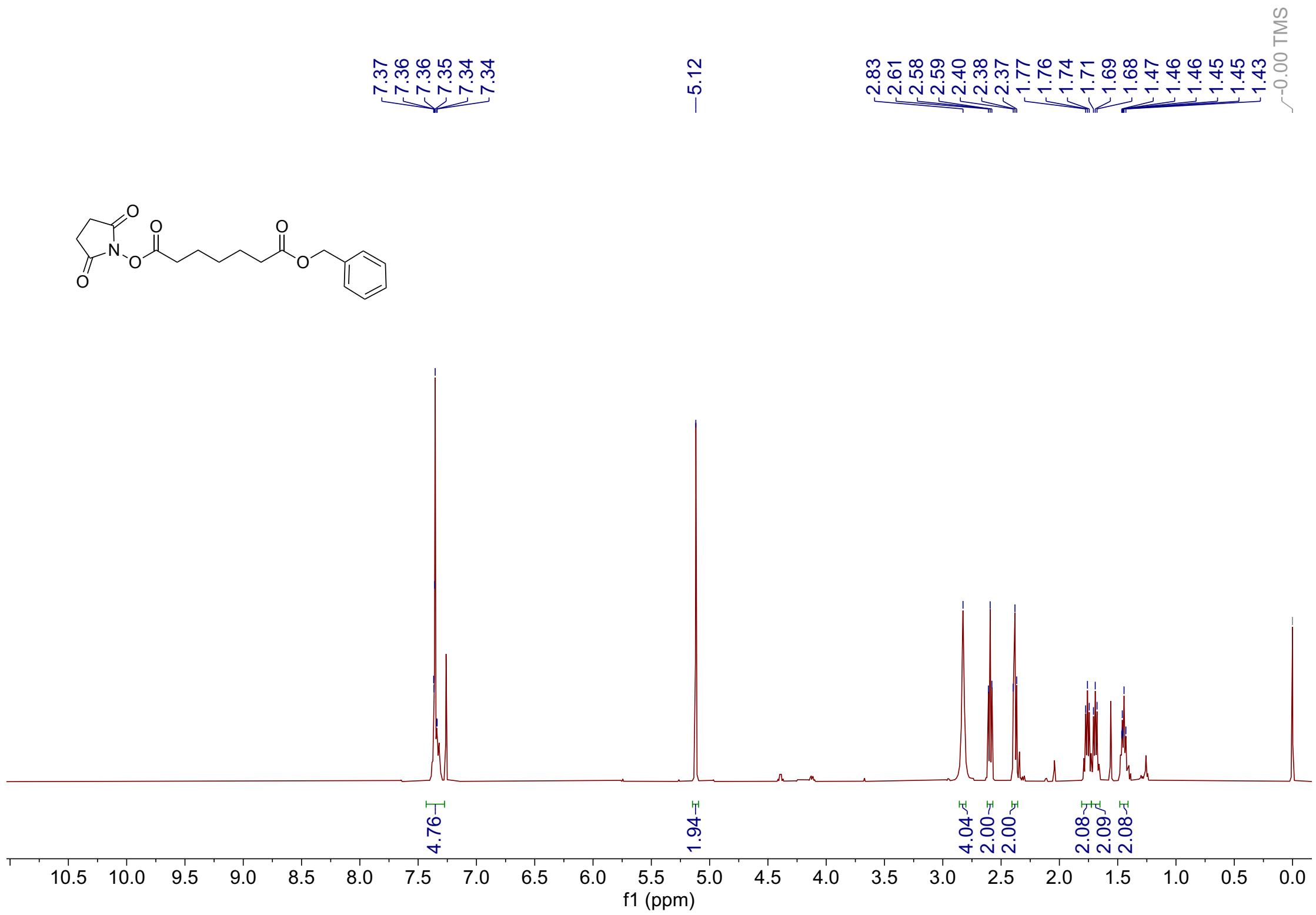

Figure S137. ^{1}H NMR (500 MHz, $CDCl_3$) for 15.

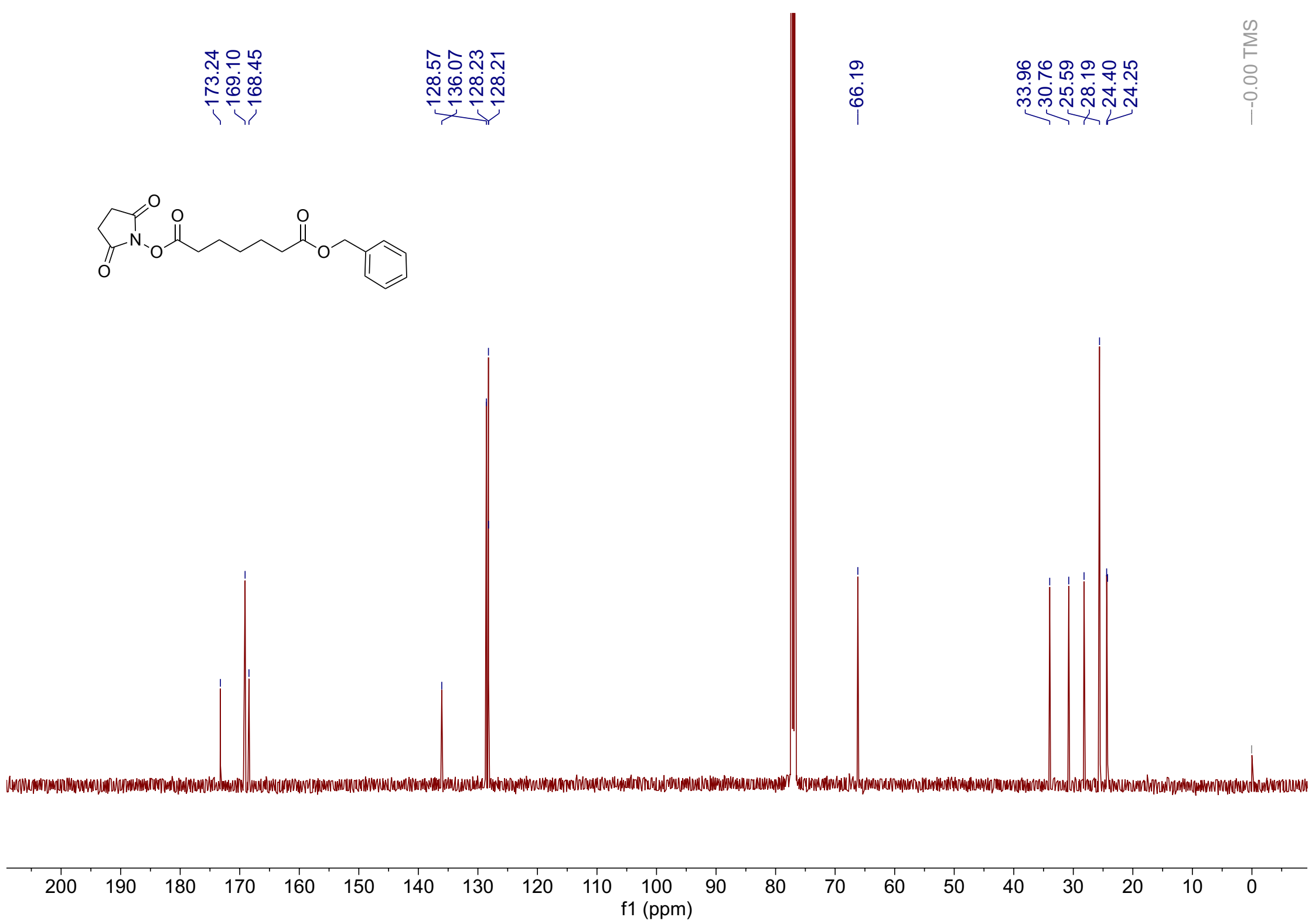

Figure S138. ^{13}C NMR (126 MHz, $CDCl_3$) for 15.

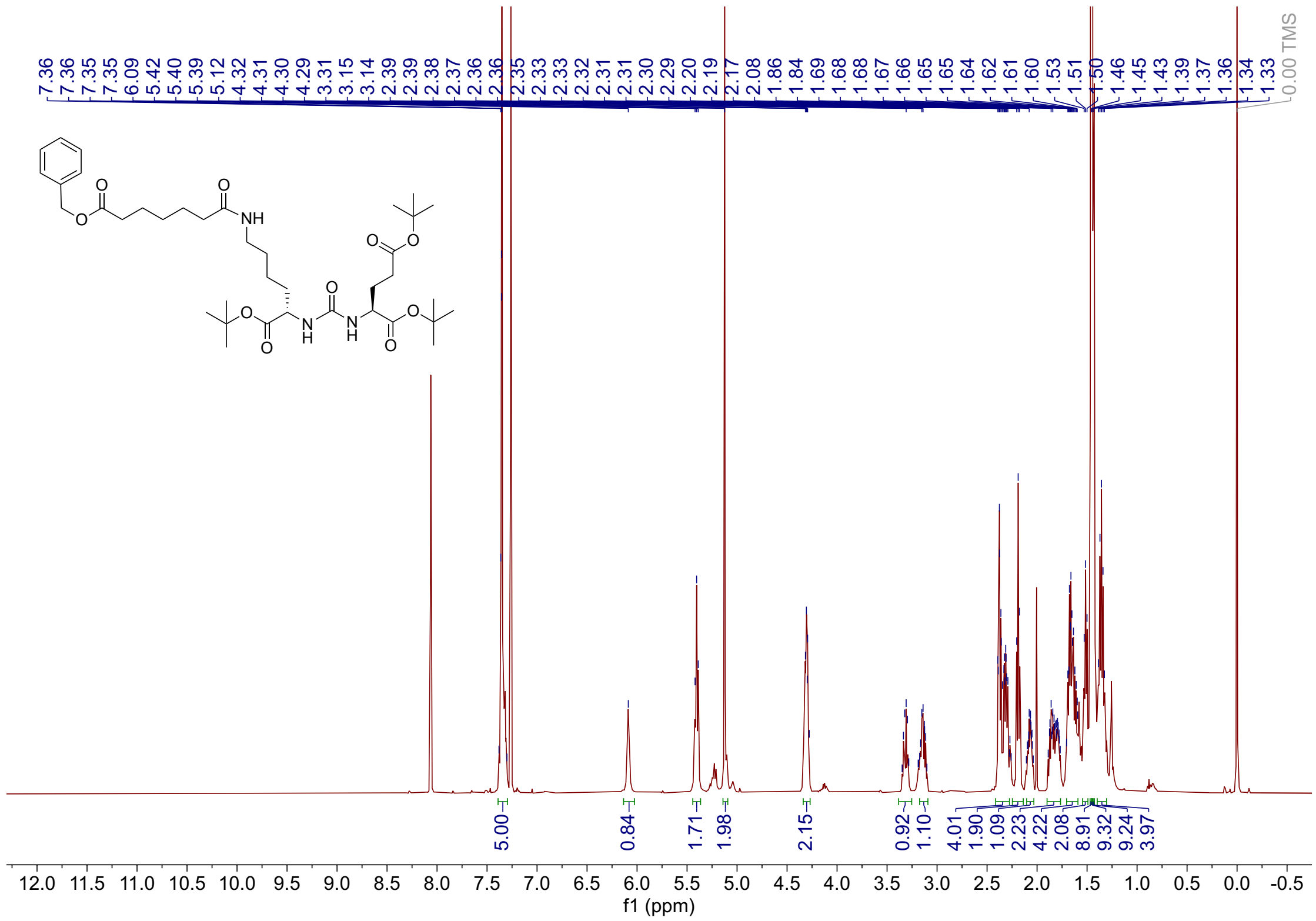

Figure S139. ^{1}H NMR (500 MHz, $CDCl_3$) for 17.

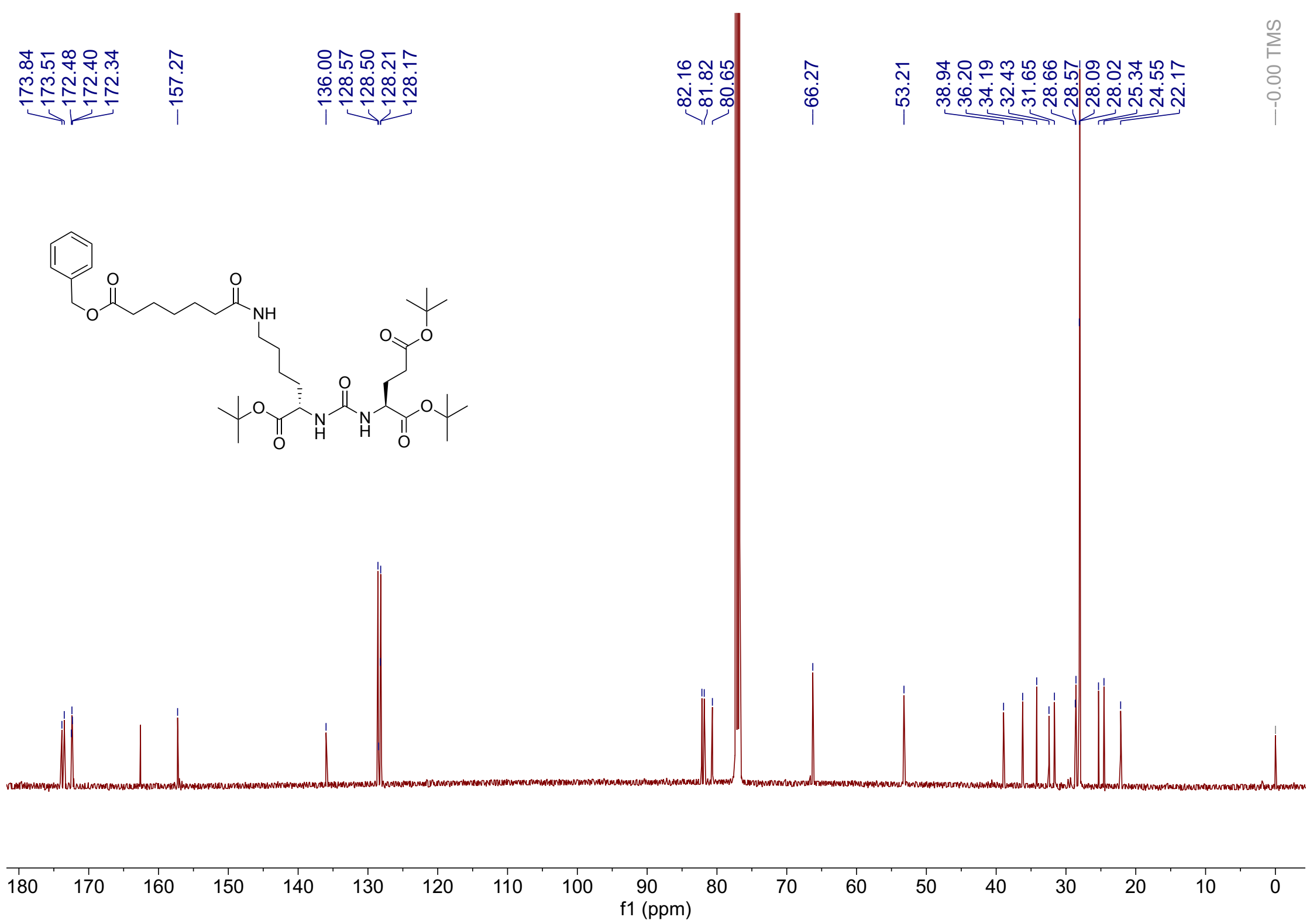

Figure S140. ^{13}C NMR (126MHz, $CDCl_3$) for 17.

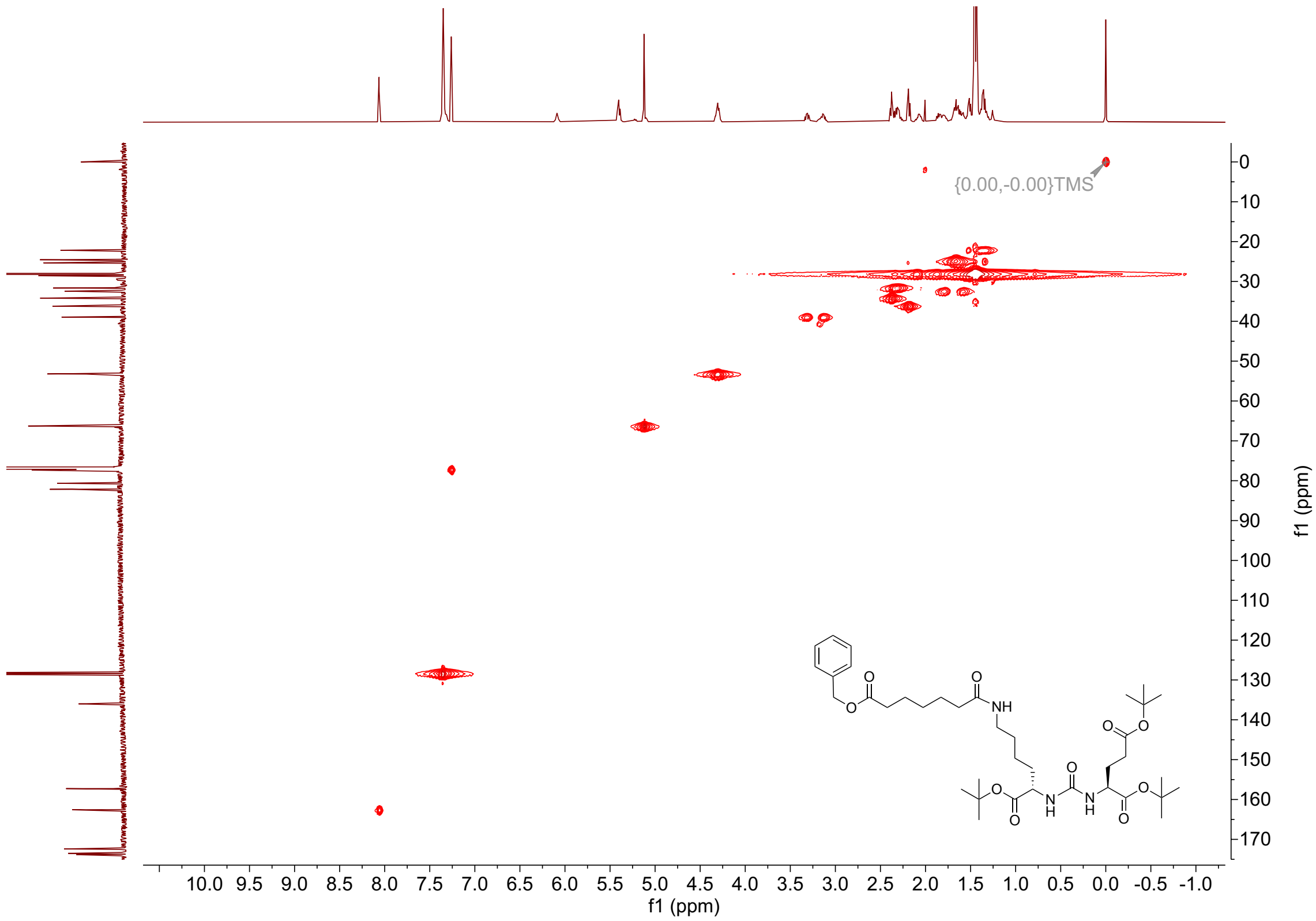

Figure S141. ^1H – ^{13}C NMR spectrum (126MHz, $CDCl_3$) for 17.

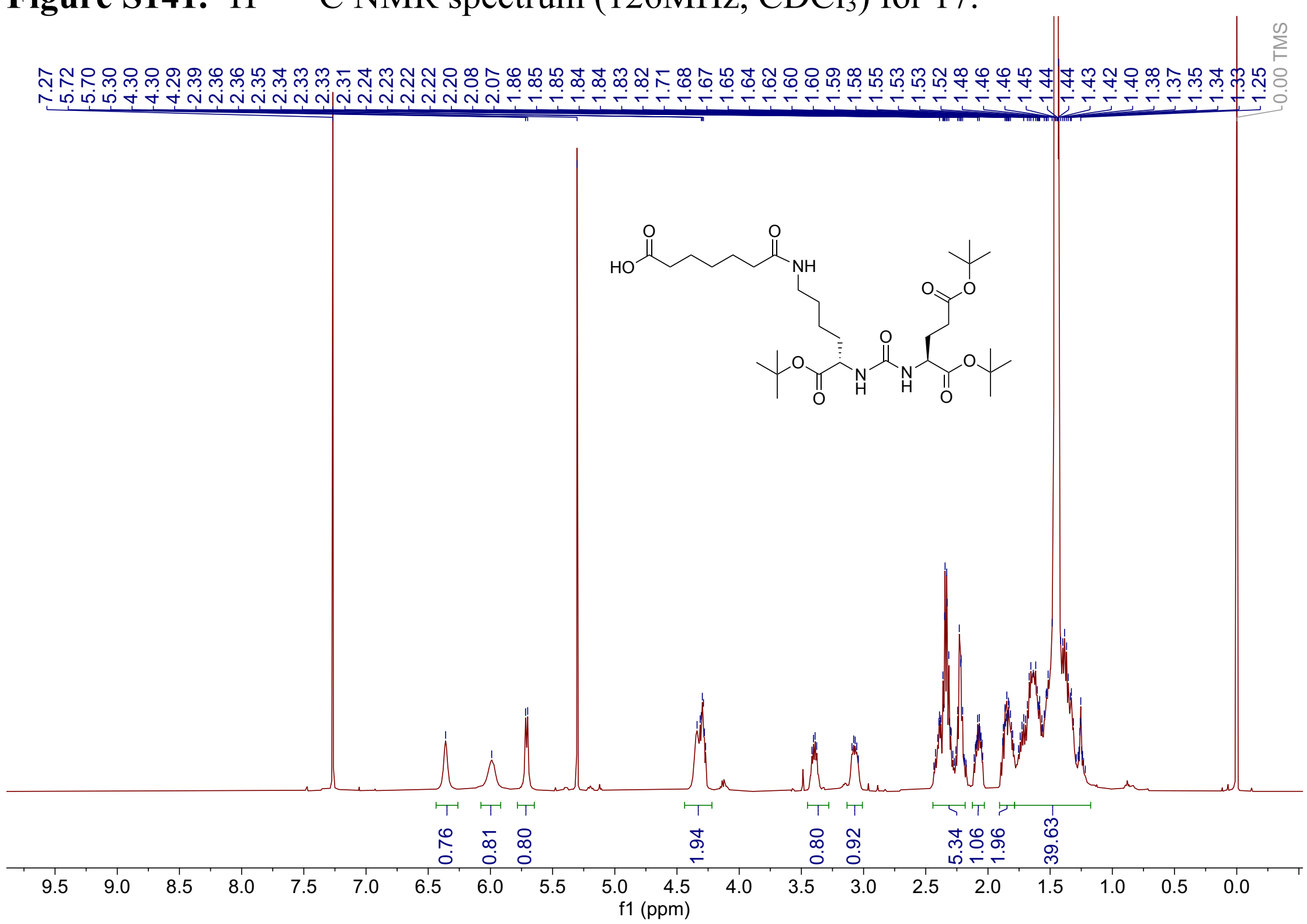

Figure S142. ^1H NMR (500 MHz, $CDCl_3$) for 18.

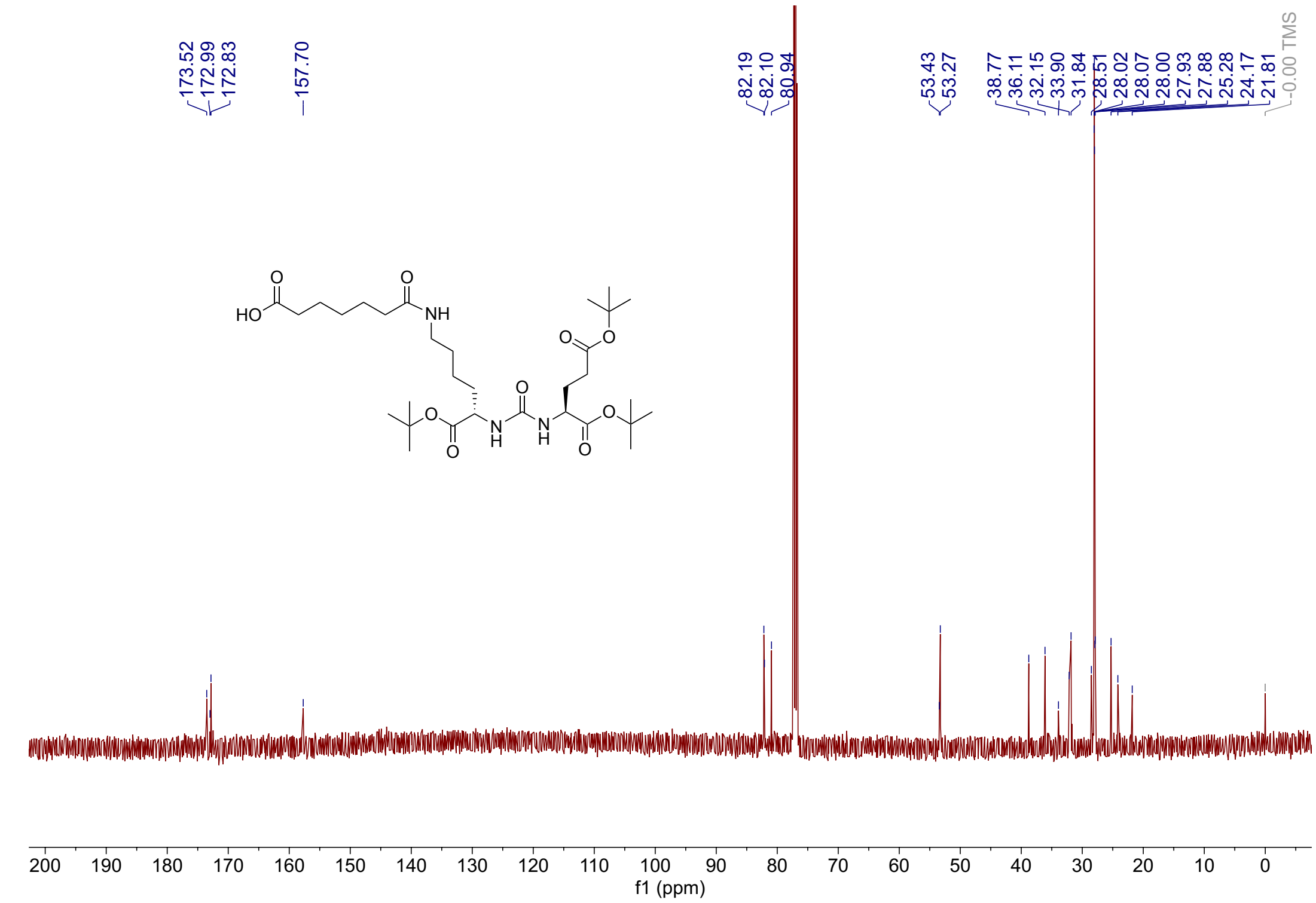

Figure S143. ^{13}C NMR (126MHz, $CDCl_3$) for 18.

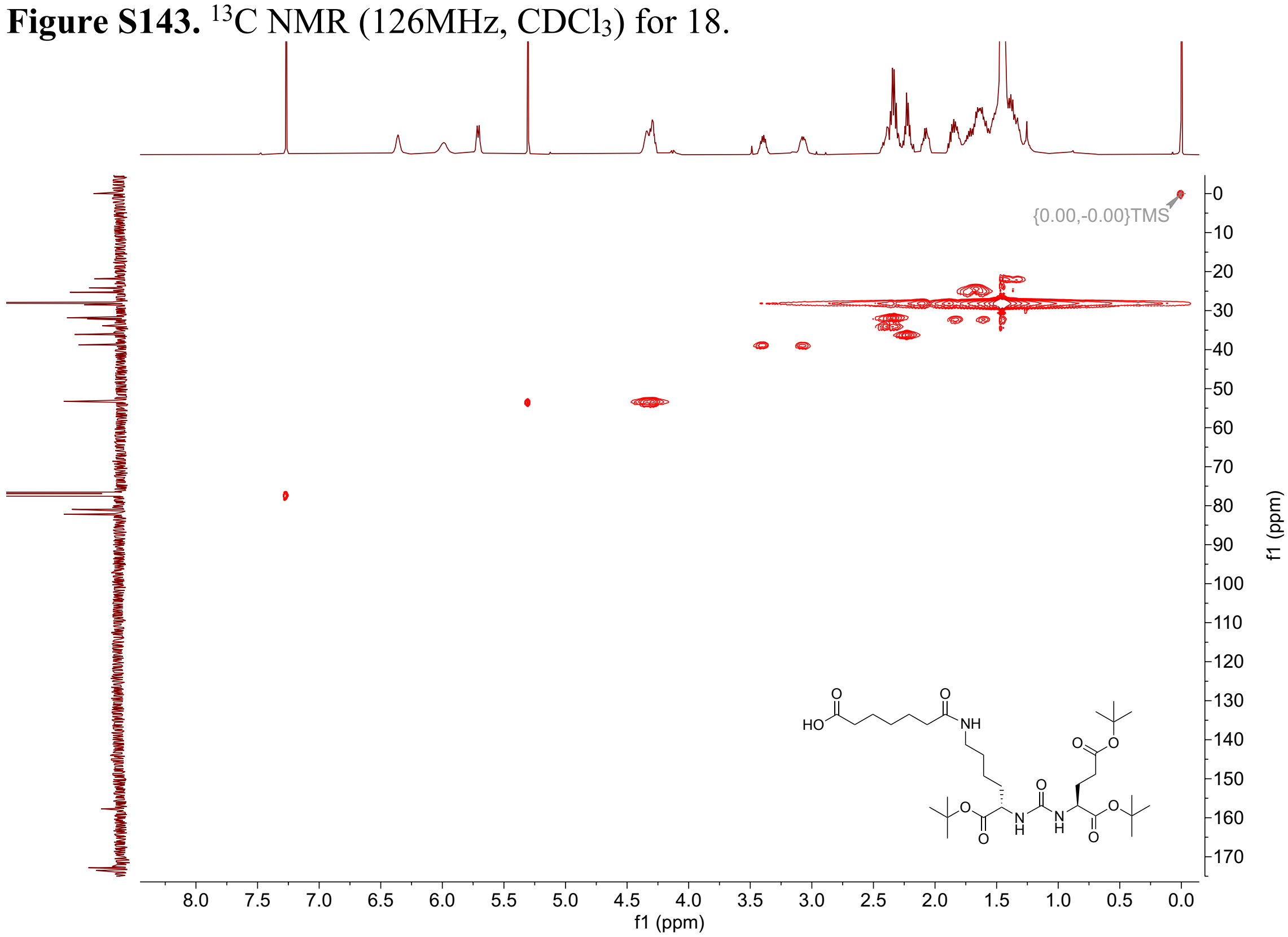

Figure S144. ^1H-^{13}C NMR spectrum for 18.

6.2 PD1 and PD2 Complexation Protocol

To a ligand stock solution of known concentration, as determined by UV-vis spectroscopy (PD1: 27256 M^{-1} cm^{-1} at 213 nm in 10 mM pH 5.5 NH4OAc buffer; PD2: 17075 M^{-1} cm^{-1} at 260 nm in 10 mM pH 5.5 NH4OAc buffer determined by Cu^{2+} titration), 1 equivalent of Zn^{2+}, Ga^{3+}, Fe^{3+}, In^{3+}, and Co^{2+} was added. Total reaction volumes were 150 µL. The reactions proceeded at room temperature for 1 hour, with the complexation confirmed by LCMS (Method I).
The Co^{3+} complex was generated by bubbling air through a solution of the Co^{2+} complex for 3 – 5 days. Formation of the oxidized product was confirmed by LC-MS, and the reaction mixture was subsequently purified by analytical HPLC (Method A).
Cleavage of PD1 and PD2 cold complexes (0.75 mM) was monitored at 80 ºC and pH values of 7.4 (25 mM pH 7.4 HEPES buffer). An aliquot of the complex was placed in an aqueous solution at pH 7.4 and incubated for 24 hours at 80 ºC. The reaction was monitored via analytical HPLC at relevant time periods.
Cathepsin B enzyme cleavage assay of PD3 conjugate was performed according to previously published procedure.[9]

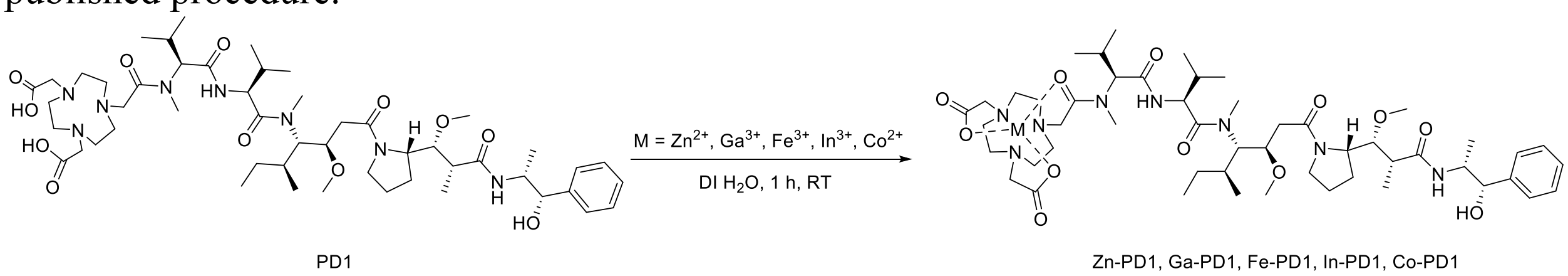

Scheme S4. Complexation scheme for Zn-PD1, Ga-PD1, Fe-PD1, In-PD1, and Co-PD1.

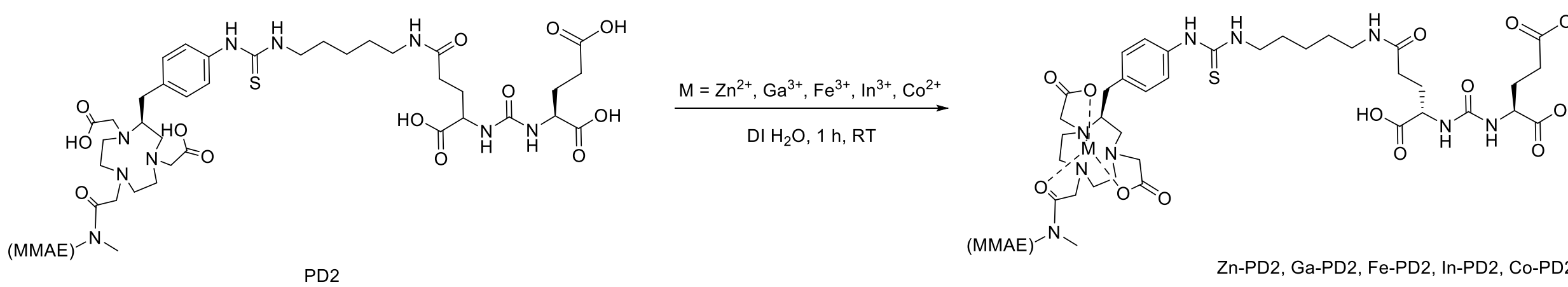

Scheme S5. Complexation scheme for Zn-PD2, Ga-PD2, Fe-PD2, In-PD2, and Co-PD2.

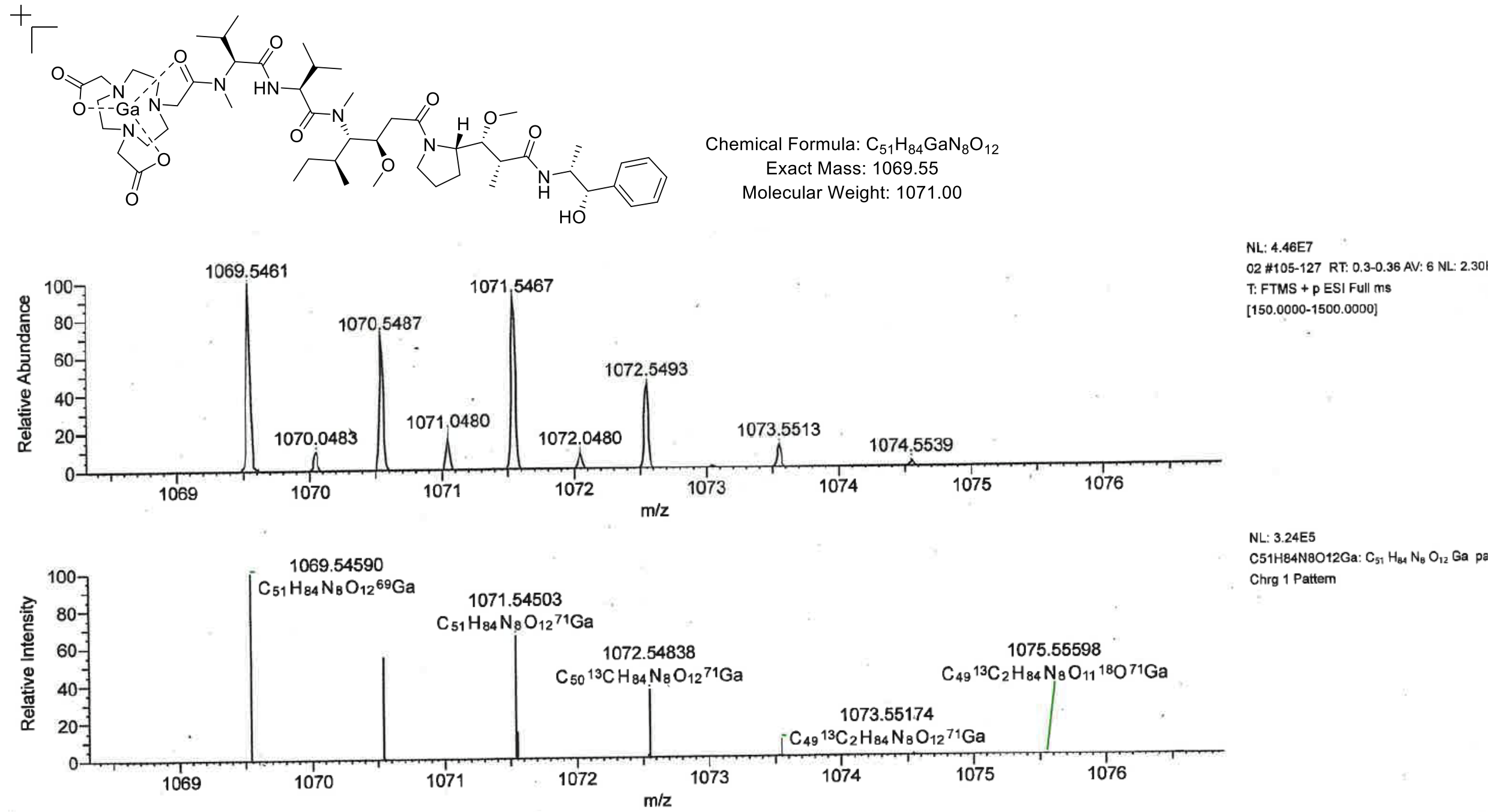

Figure S145. ESI-HRMS of Ga-PD1. HRESI calc. for $C_{51}H_{84}GaN_8O_{12}$ 1069.5464 Found 1069.5461 $[M]^+$.

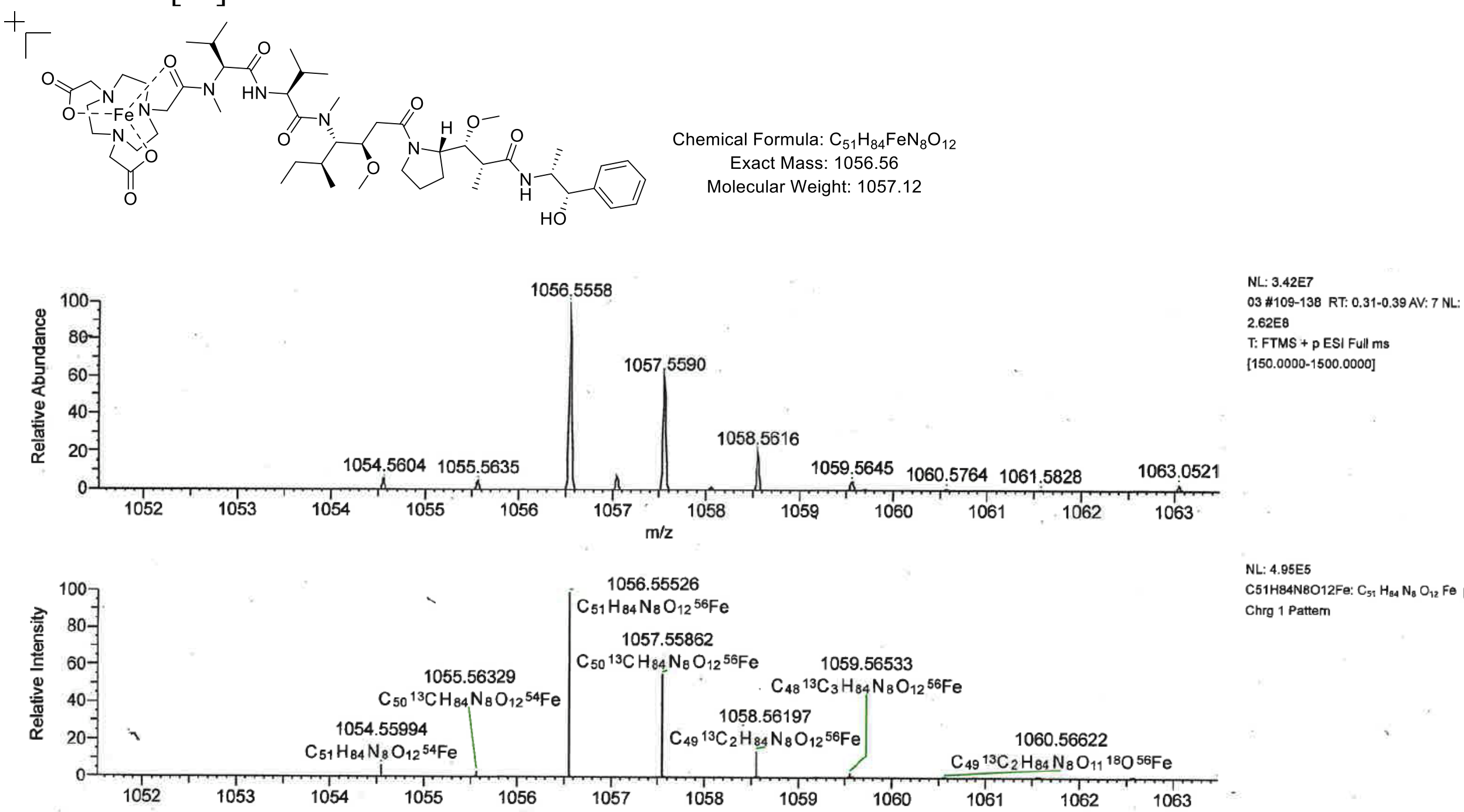

Figure S146. HRESI-MS of Fe-PD1. HRESI calc. for $C_{51}H_{84}FeN_8O_{12}$ 1056.5558. Found 1056.5558 $[M]^+$.

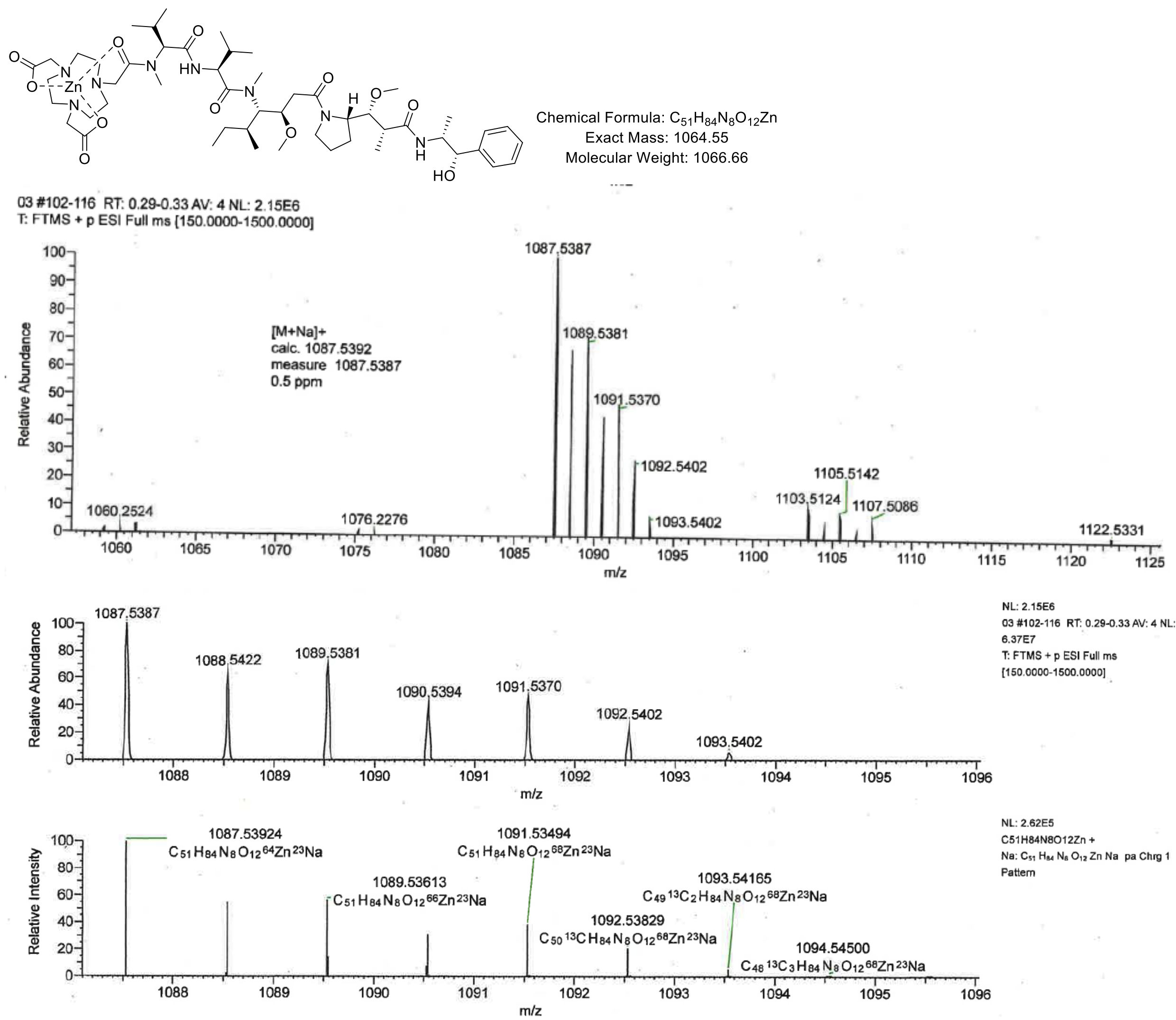

Figure S147. HRESI-MS of Zn-PD1. HRESI calc. for $C_{51}H_{84}ZnN_8O_{12}$ 1064.5500. Found 1087.5387 $[M+Na]^+$.

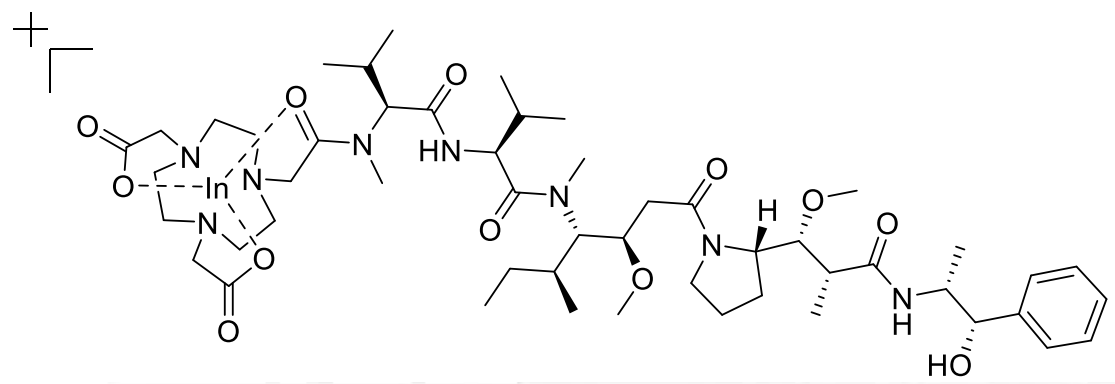

Chemical Formula: $C_{51}H_{84}InN_8O_{12}$
Exact Mass: 1115.52
Molecular Weight: 1116.10

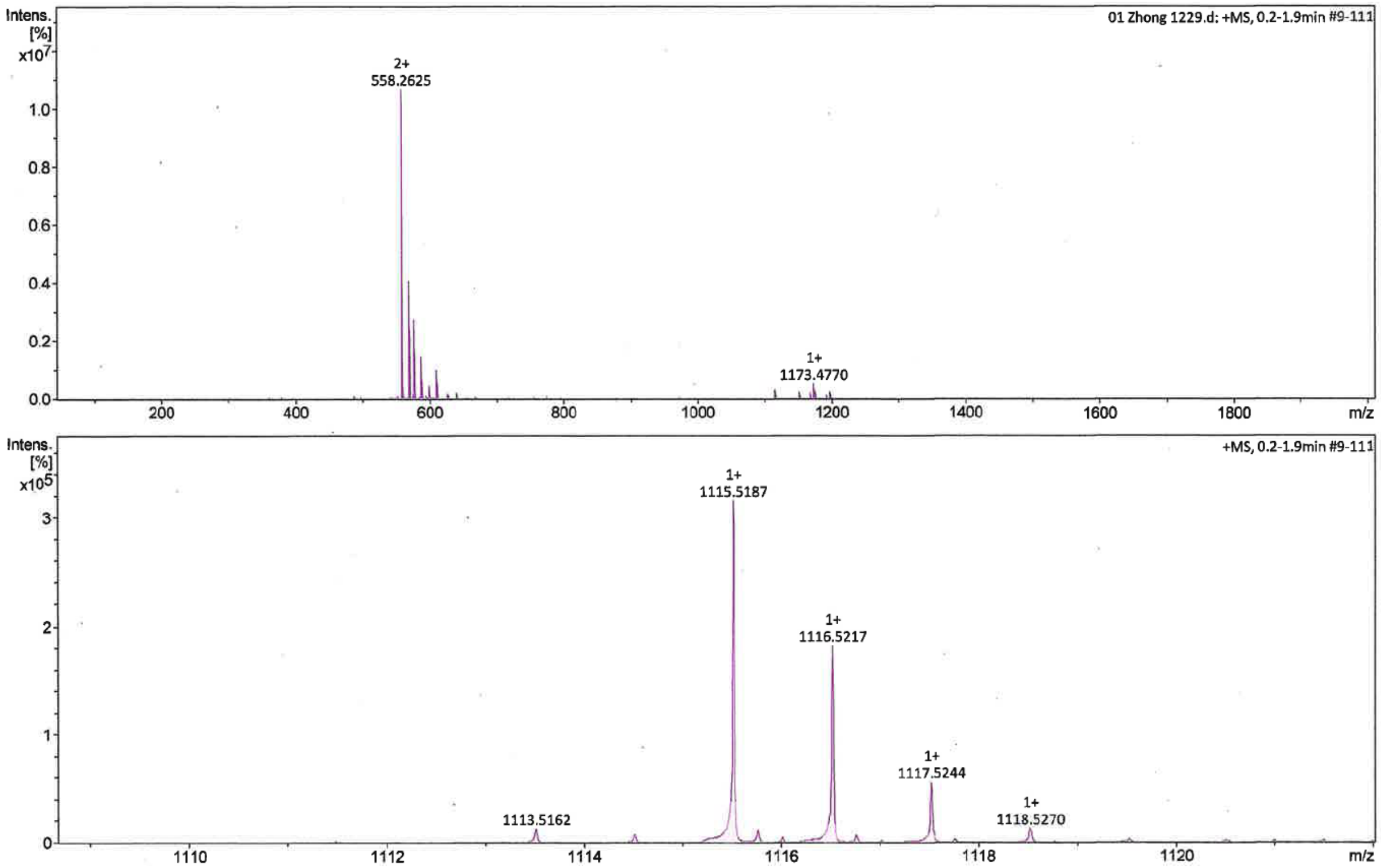

Figure S148. HRESI-MS of In-PD1. HRESI calc. for $C_{51}H_{84}InN_8O_{12}$ 1115.5247. Found 1115.5187 $[M]^{+}$.

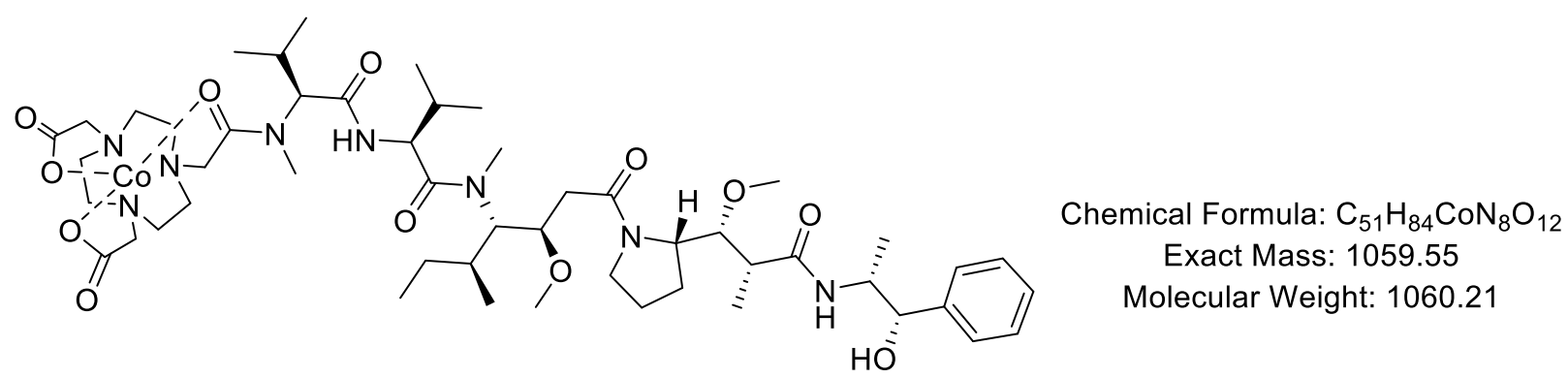

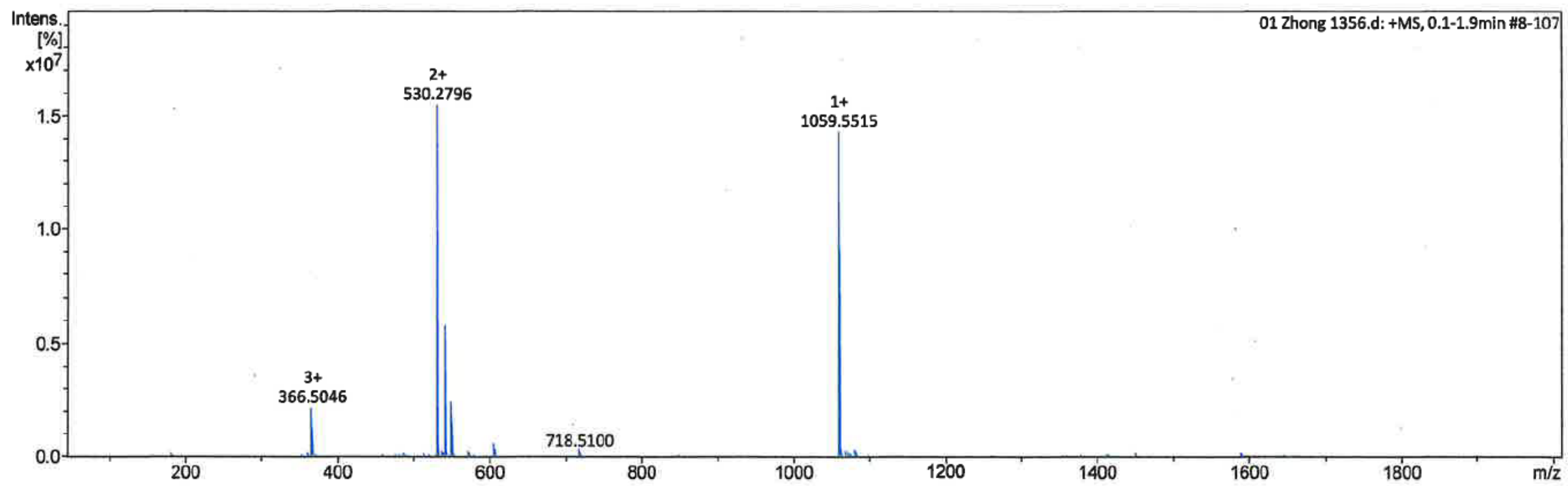

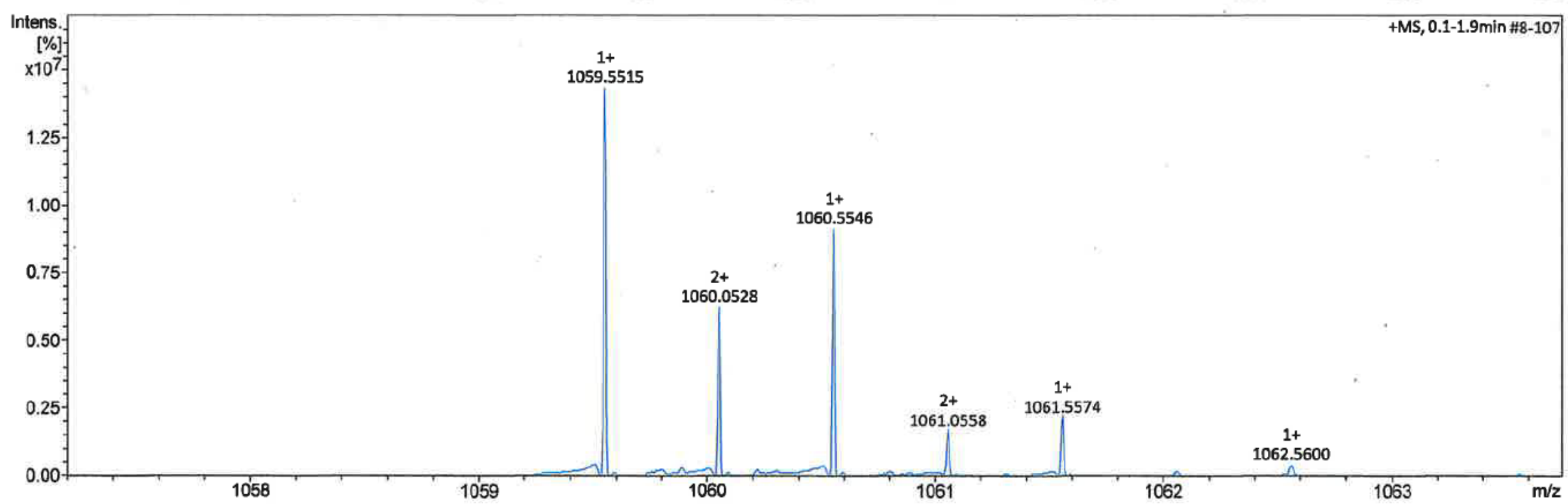

Figure S149. HRESI-MS of Co-PD1. HRESI calc. for $C_{51}H_{84}CoN_8O_{12}$ 1059.5541. Found 1059.5515 $[M]^+$.

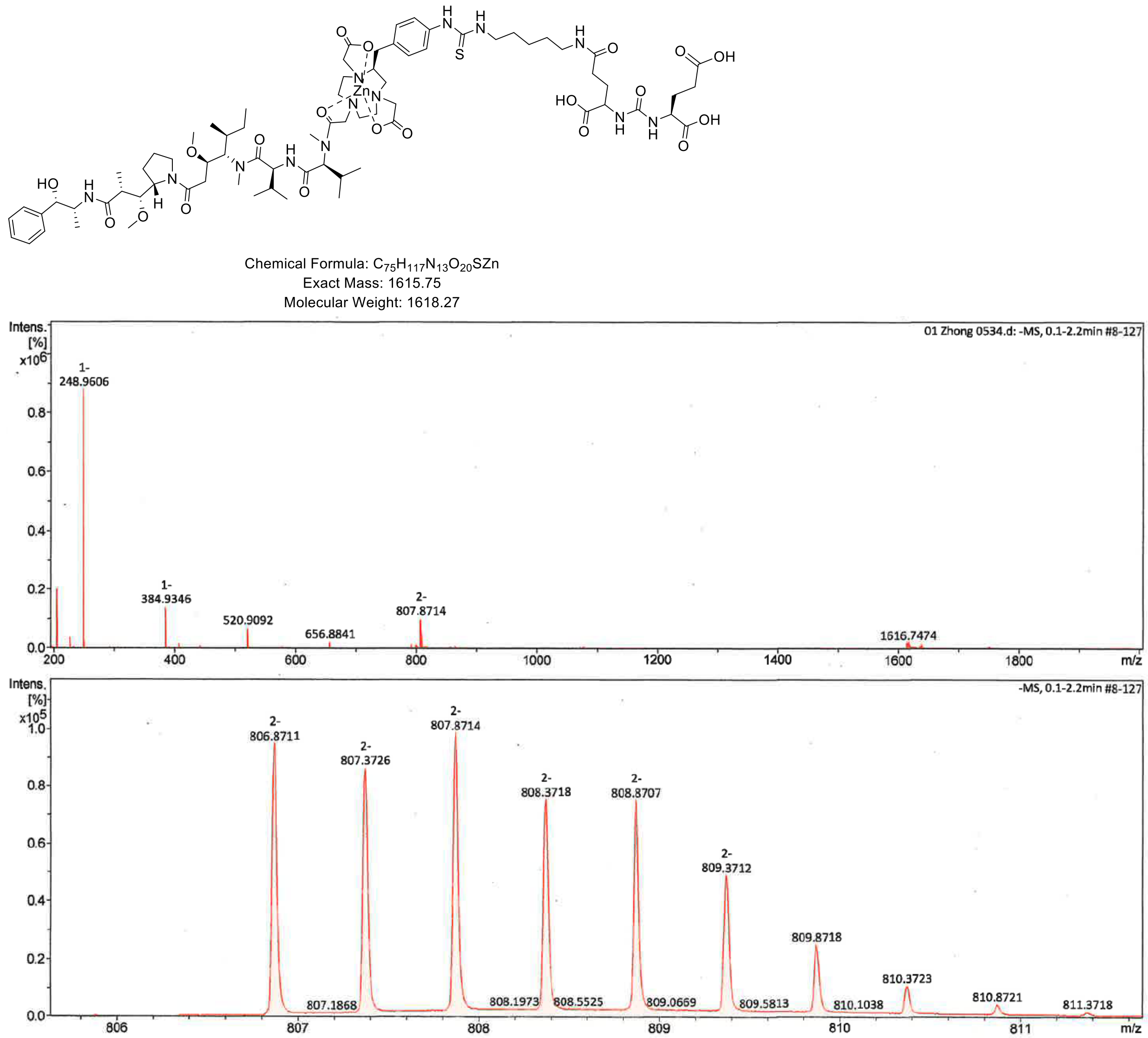

Figure S150. HRESI-MS of Zn-PD2. HRESI calc. for $C_{75}H_{117}N_{13}O_{20}SZn$ 1615.7550. Found 806.8711 $[M+2H]^{2+}$.

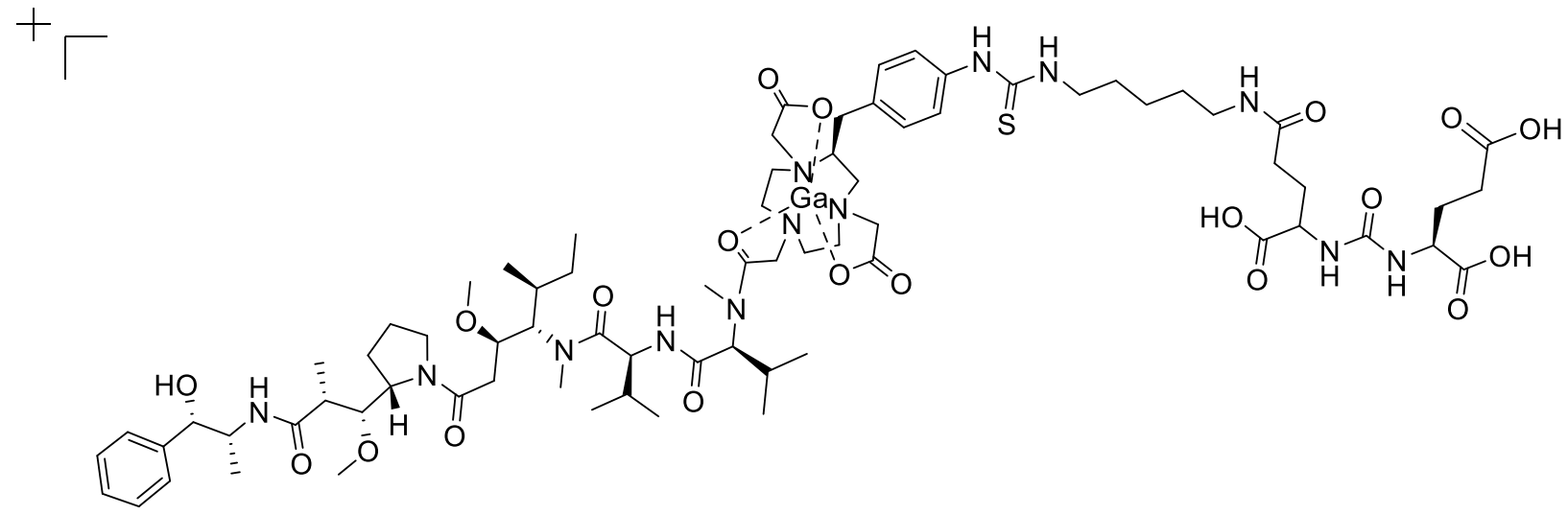

Chemical Formula: $C_{75}H_{117}GaN_{13}O_{20}S$
Exact Mass: 1620.75
Molecular Weight: 1622.61

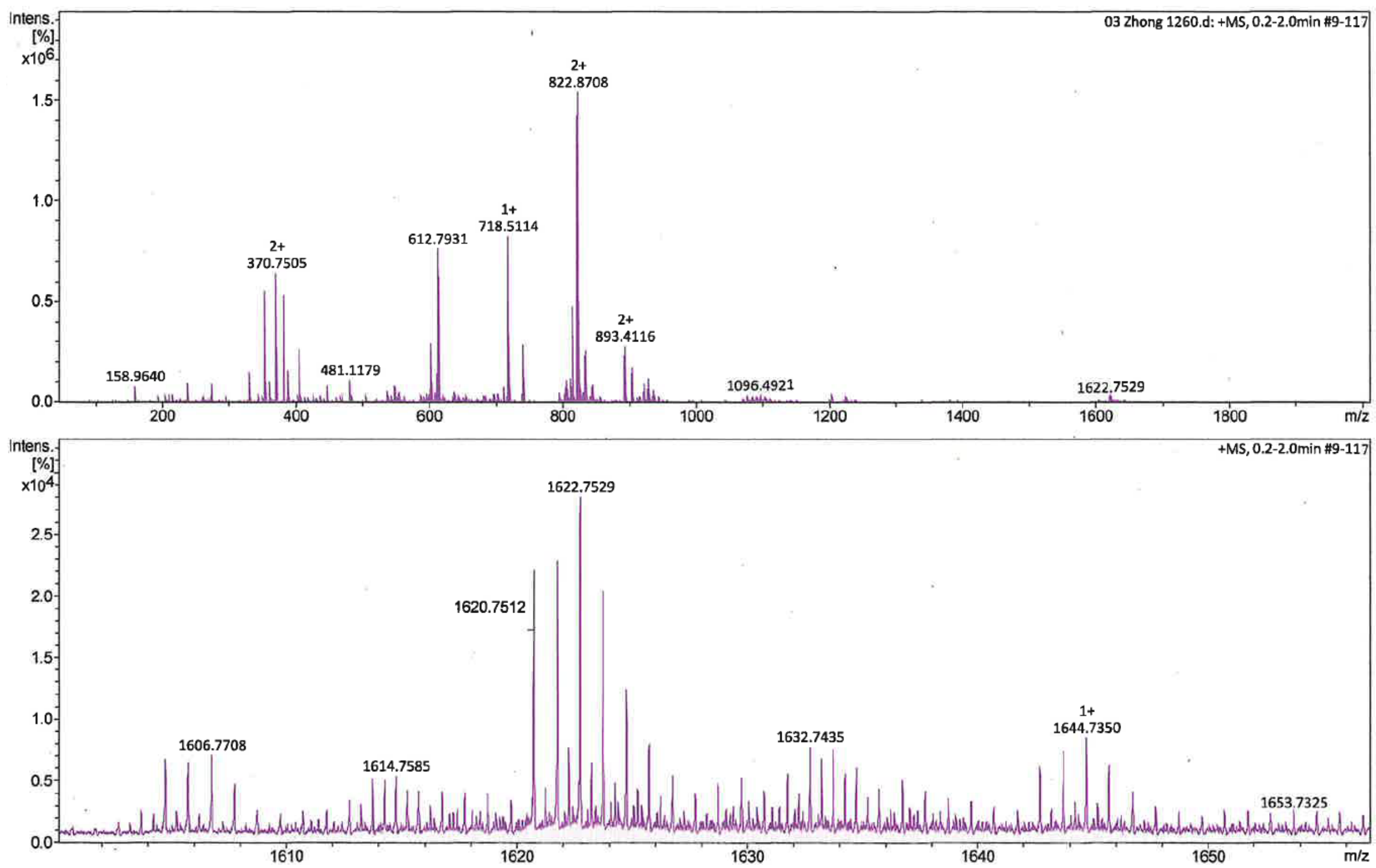

Figure S151. HRESI-MS of Ga-PD2. HRESI calc. for $C_{75}H_{117}GaN_{13}O_{20}S$ 1620.7514. Found 1620.7512 $[M]^{+}$.

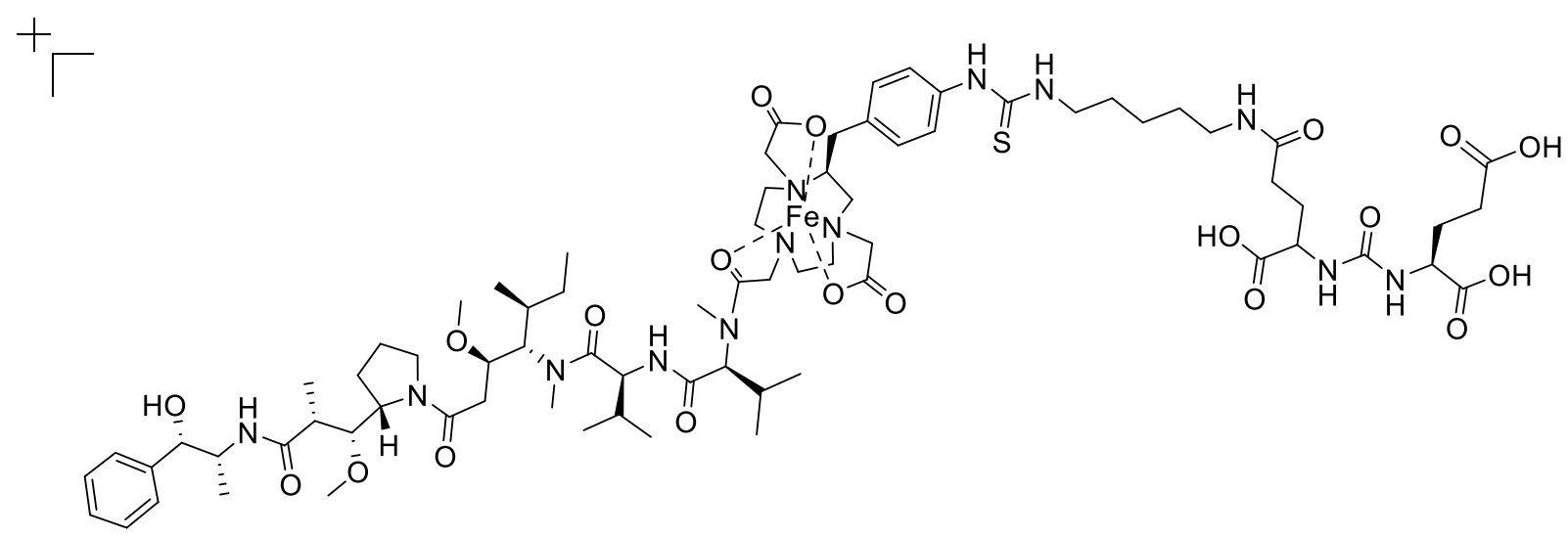

Chemical Formula: $C_{75}H_{117}FeN_{13}O_{20}S$
Exact Mass: 1607.76
Molecular Weight: 1608.74

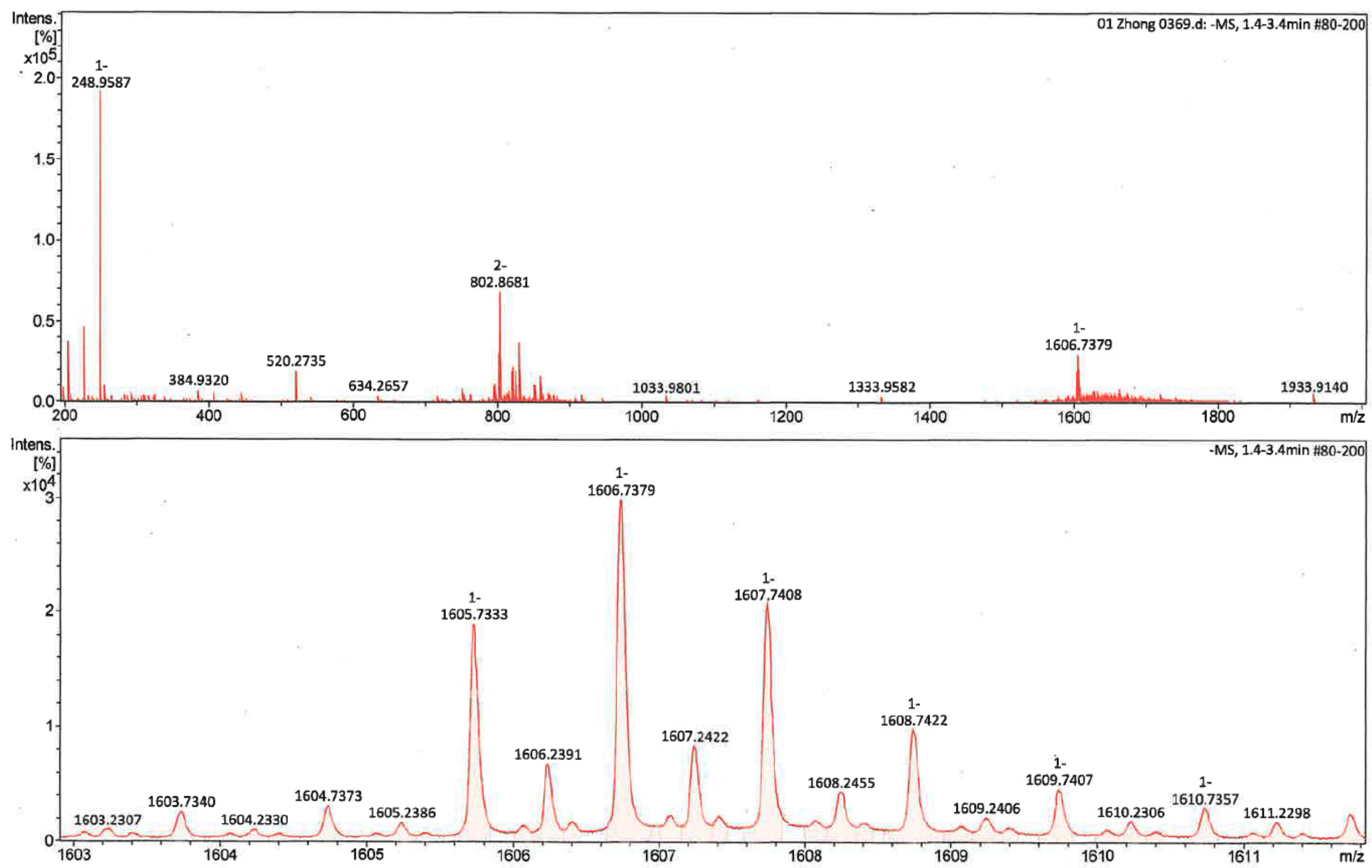

Figure S152. HRESI-MS of Fe-PD2. HRESI calc. for $C_{75}H_{117}FeN_{13}O_{20}S$ 1606.7379. Found 1606.7379 $[M-H]^-$.

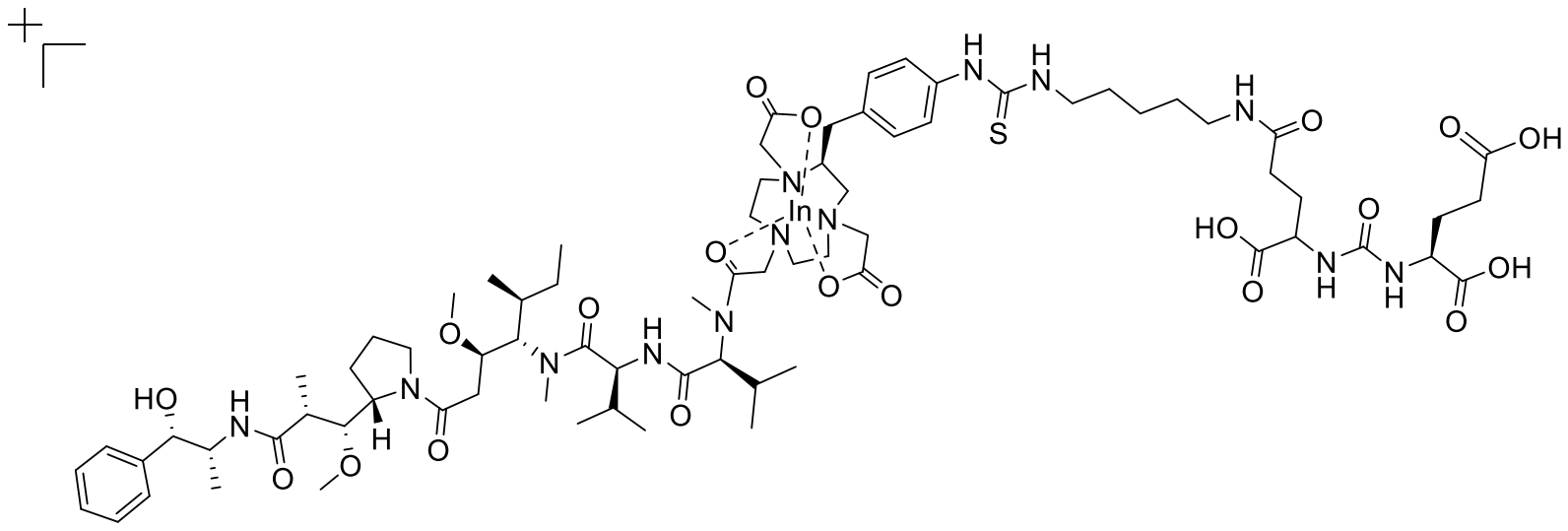

Chemical Formula: $C_{75}H_{117}InN_{13}O_{20}S$
Exact Mass: 1666.73
Molecular Weight: 1667.71

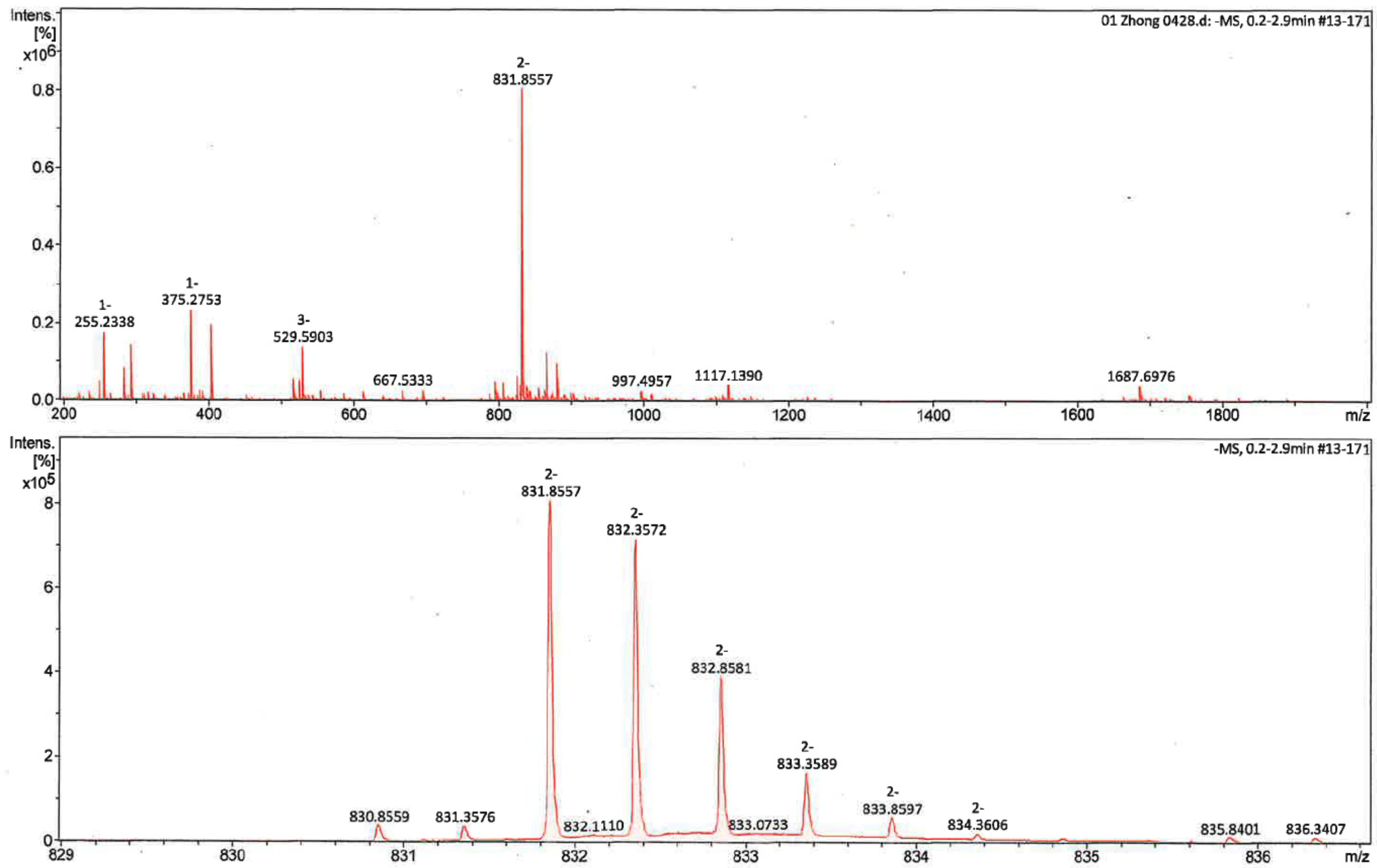

Figure S153. HRESI-MS of In-PD2. HRESI calc. for $C_{75}H_{117}InN_{13}O_{20}S$ 1666.7297. Found 806.8711 [M-H]$^-$.

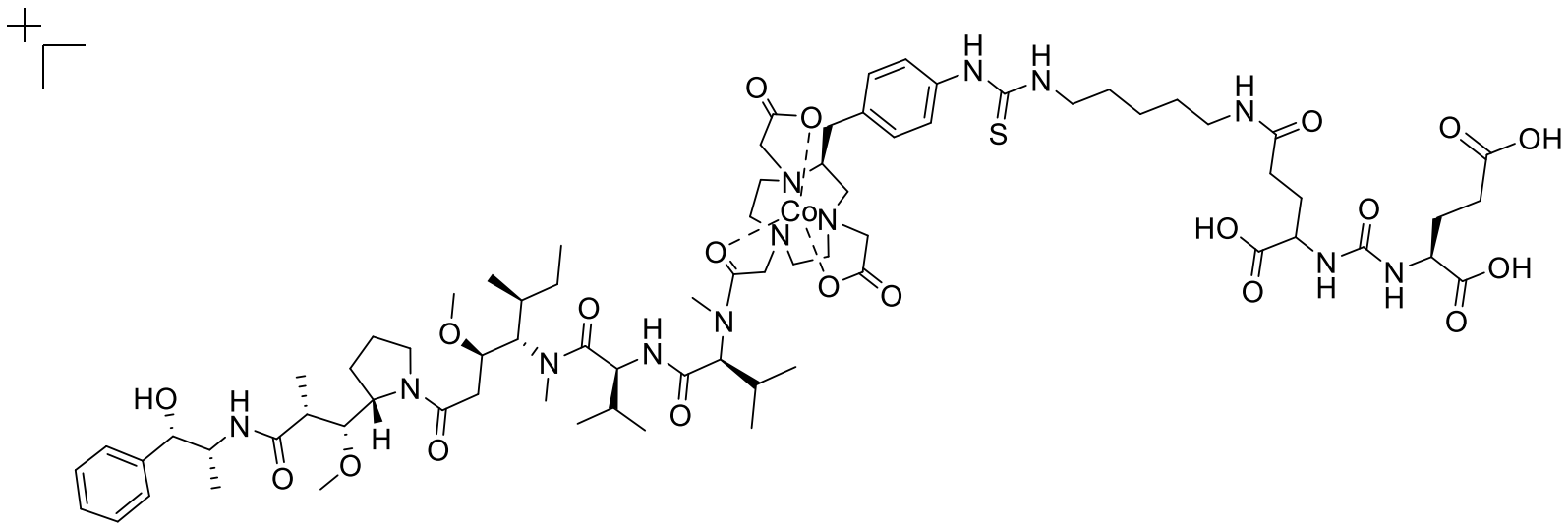

Chemical Formula: $C_{75}H_{117}CoN_{13}O_{20}S$
Exact Mass: 1610.76
Molecular Weight: 1611.83

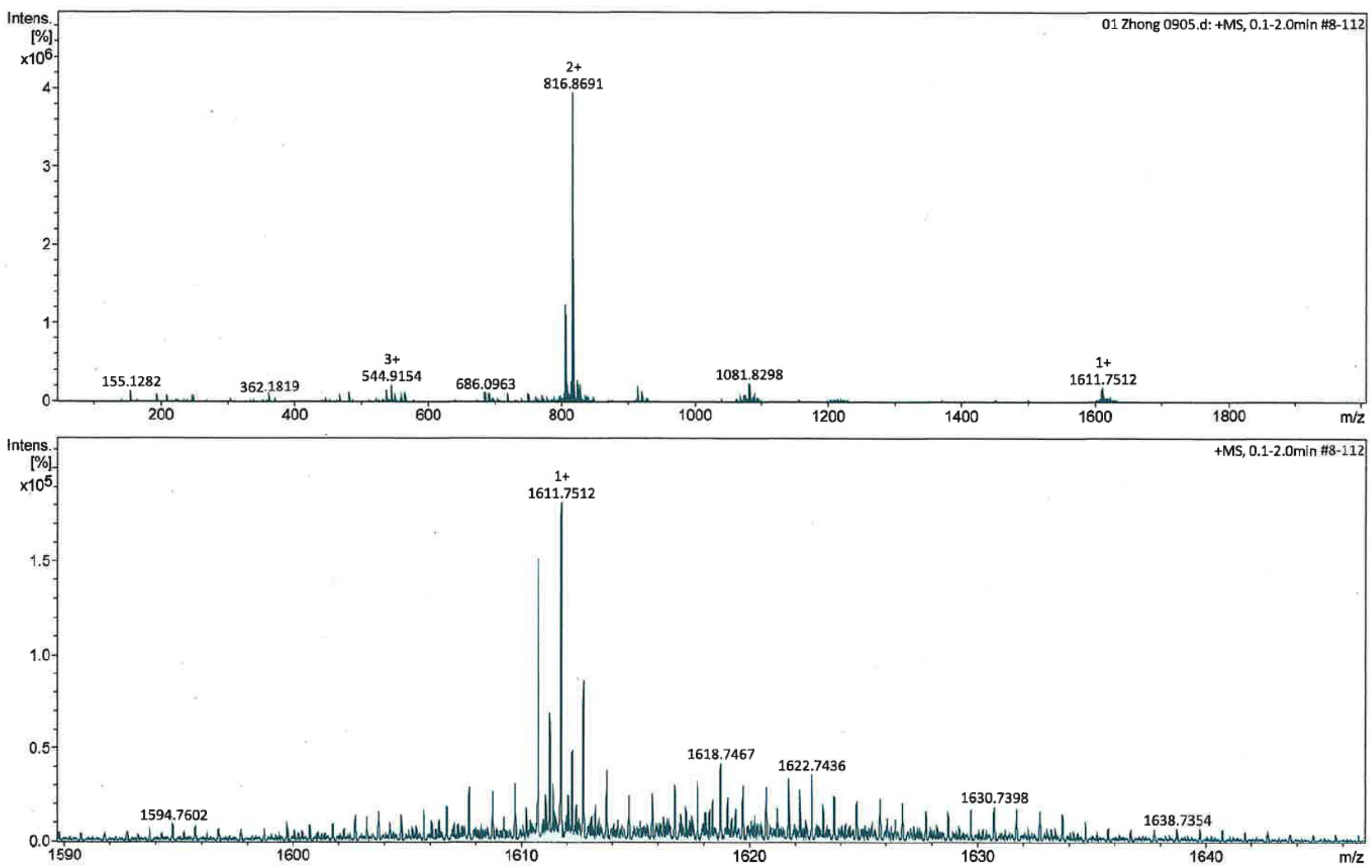

Figure S154. HRESI-MS of Co-PD2. HRESI calc. for $C_{75}H_{117}CoN_{13}O_{20}S$ 1610.7590. Found 816.8691 $[M+Na]^{2+}$.

6.3 Cleavage assay of PD1 and PD2 complexes

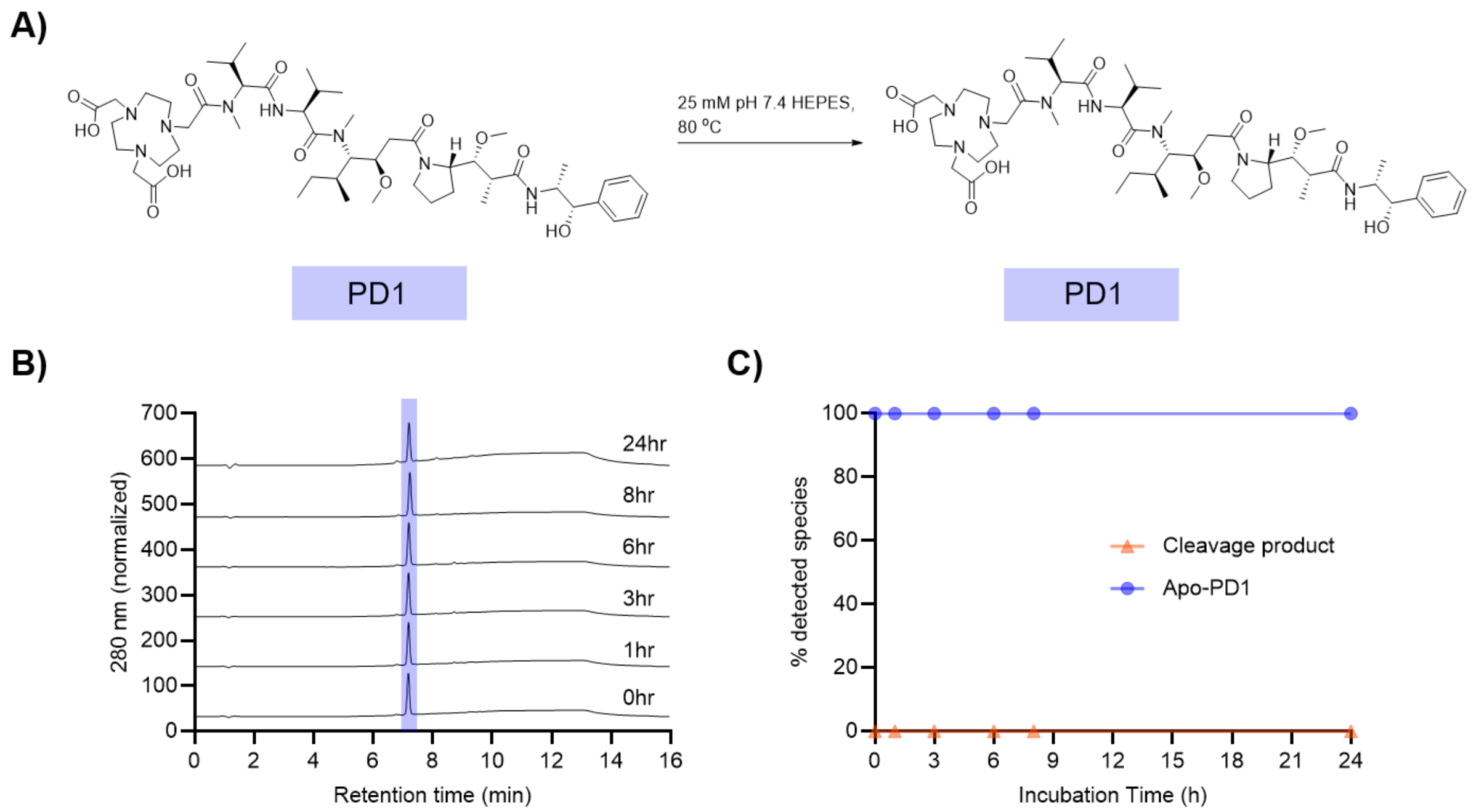

Figure S155. **(A)** Reaction scheme of apo-PD1 cleavage assay. **(B)** Analytical HPLC chromatograms show no autolytic release of the cleavage product and MMAE. **(C)** Quantification of cleavage at 80 °C and pH 7.4.

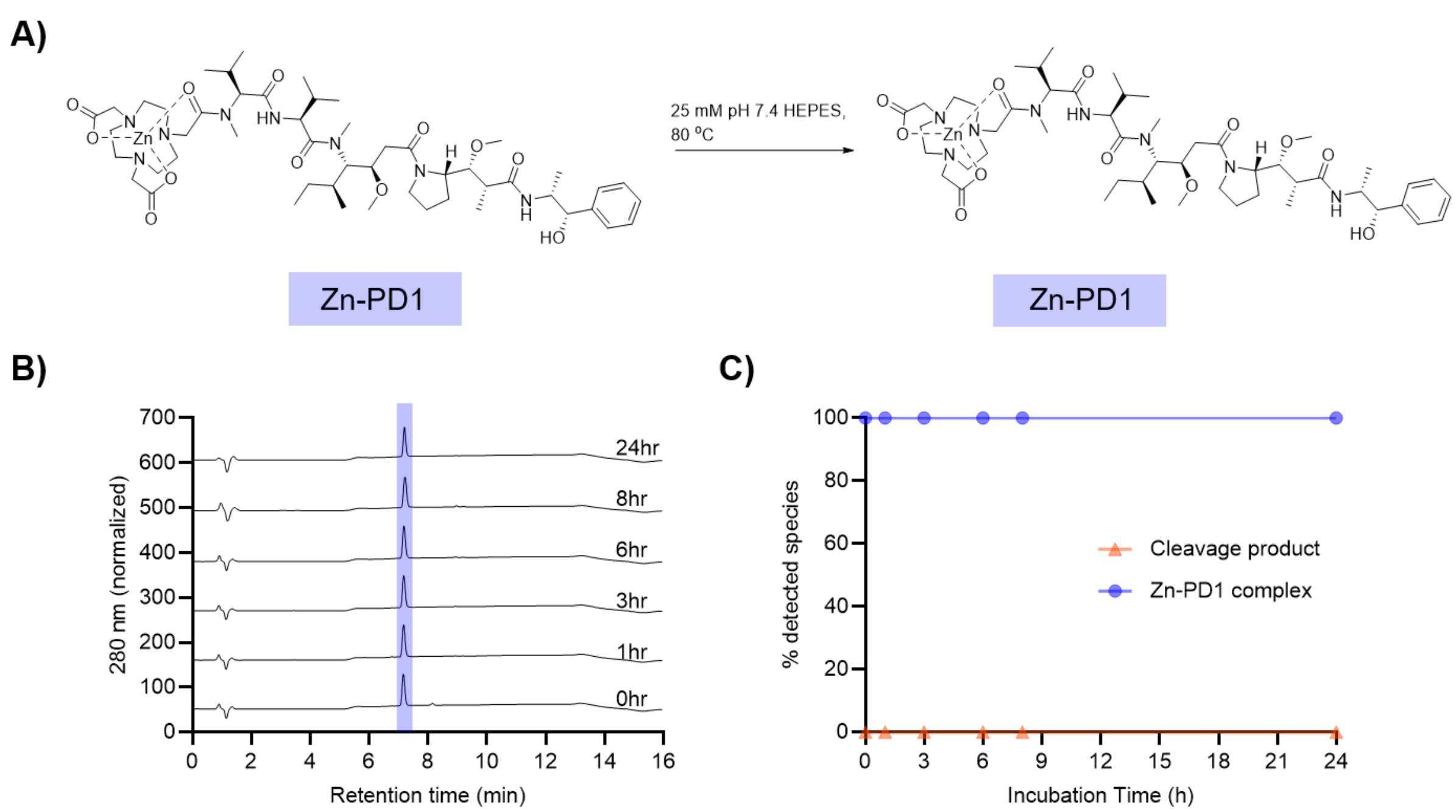

Figure S156. **(A)** Reaction scheme of Zn-PD1 cleavage assay. **(B)** Analytical HPLC chromatograms show no autolytic release of the cleavage product and MMAE. **(C)** Quantification of cleavage at 80 °C and pH 7.4.

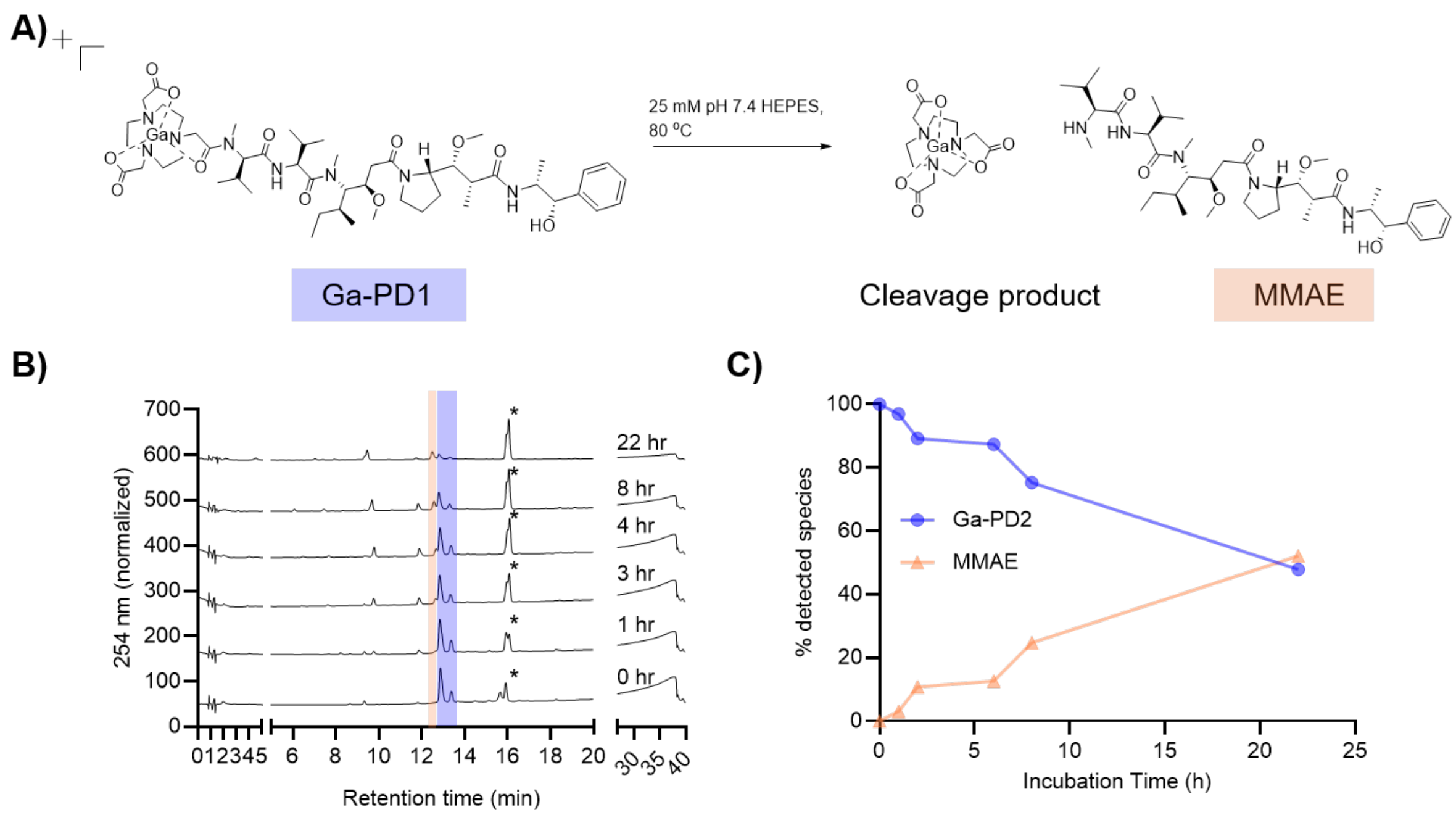

Figure S157. **(A)** Reaction scheme of Ga-PD1 cleavage assay. **(B)** Analytical HPLC chromatograms show the autolytic release of the cleavage product ^{nat}Ga-NOTA and MMAE. Asterisk (*) indicates the impurity from the buffer solution and side product during the cleavage assay. **(C)** Quantification of cleavage at 80 ºC and pH 7.4.

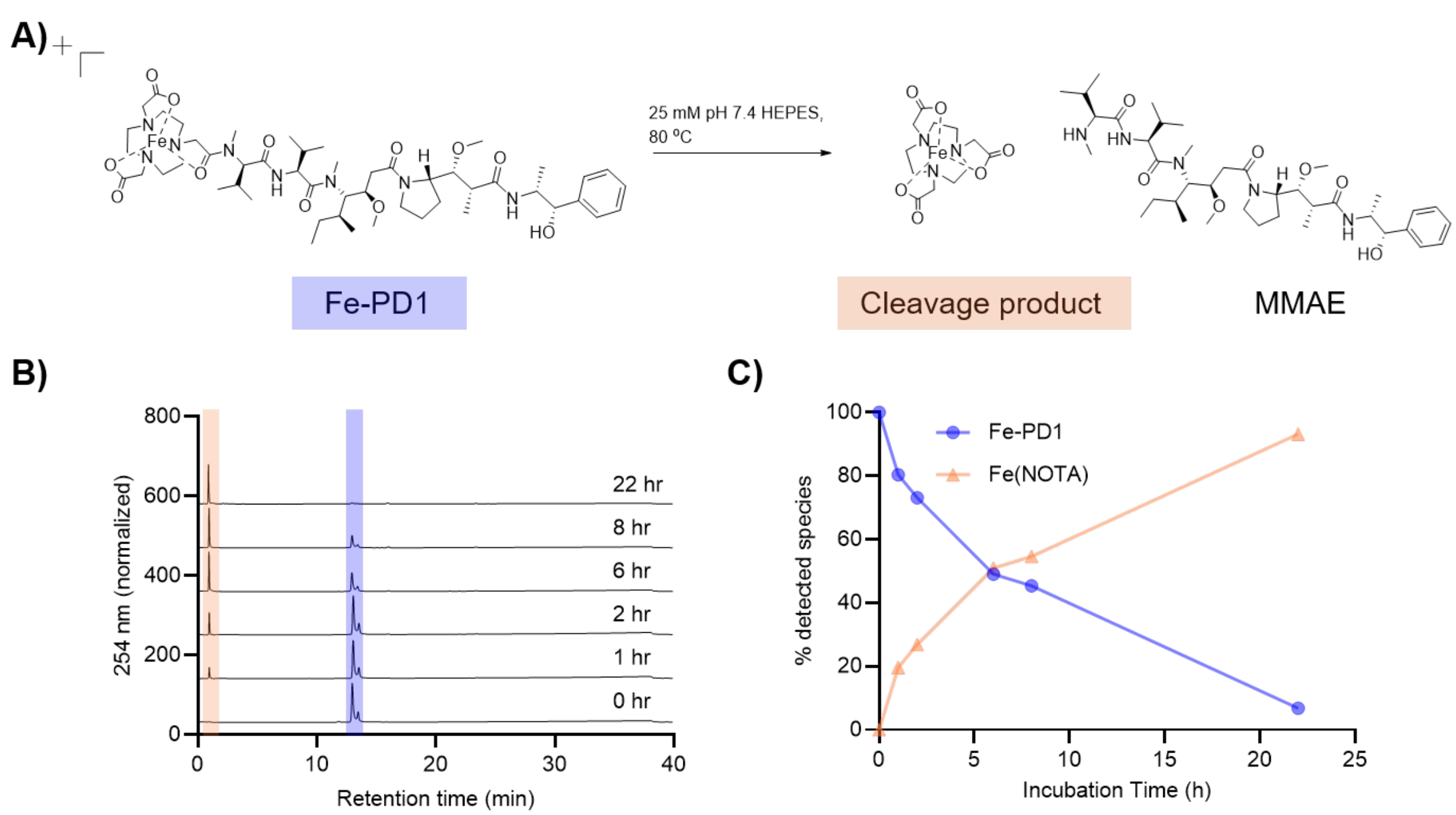

Figure S158. **(A)** Reaction scheme of Fe-PD1 cleavage assay. **(B)** Analytical HPLC chromatograms show the autolytic release of the cleavage product ^{nat}Fe-NOTA complex and MMAE. **(C)** Quantification of cleavage at 80 ºC and pH 7.4.

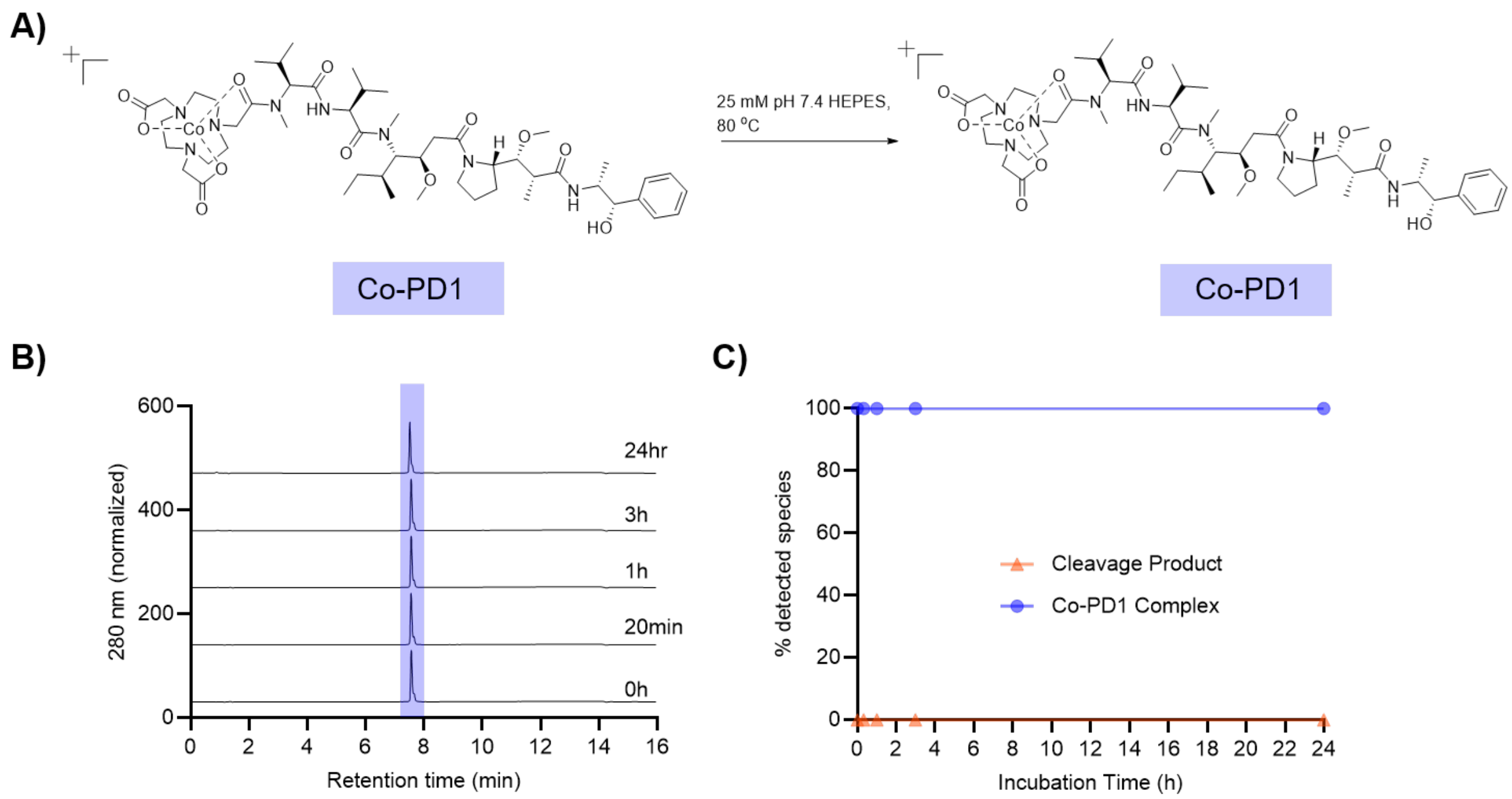

Figure S159. (A) Reaction scheme of Co-PD1 cleavage assay. **(B)** Analytical HPLC chromatograms show no autolytic release of the cleavage product and MMAE. **(C)** Quantification of cleavage at 80 °C and pH 7.4.

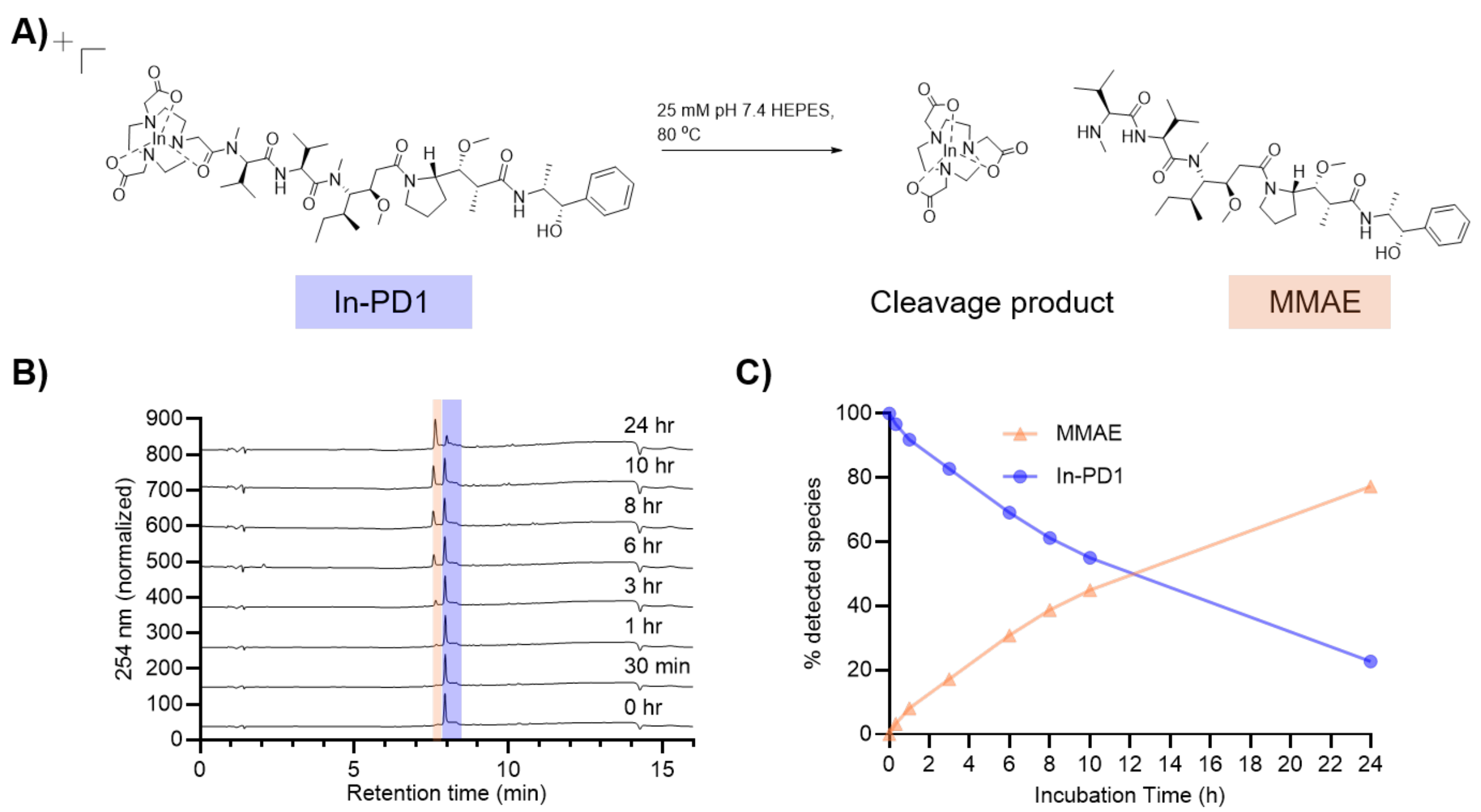

Figure S160. (A) Reaction scheme of In-PD1 cleavage assay. **(B)** Analytical HPLC chromatograms show the autolytic release of the cleavage product ^{nat}In-NOTA and MMAE. **(C)** Quantification of cleavage at 80 °C and pH 7.4.

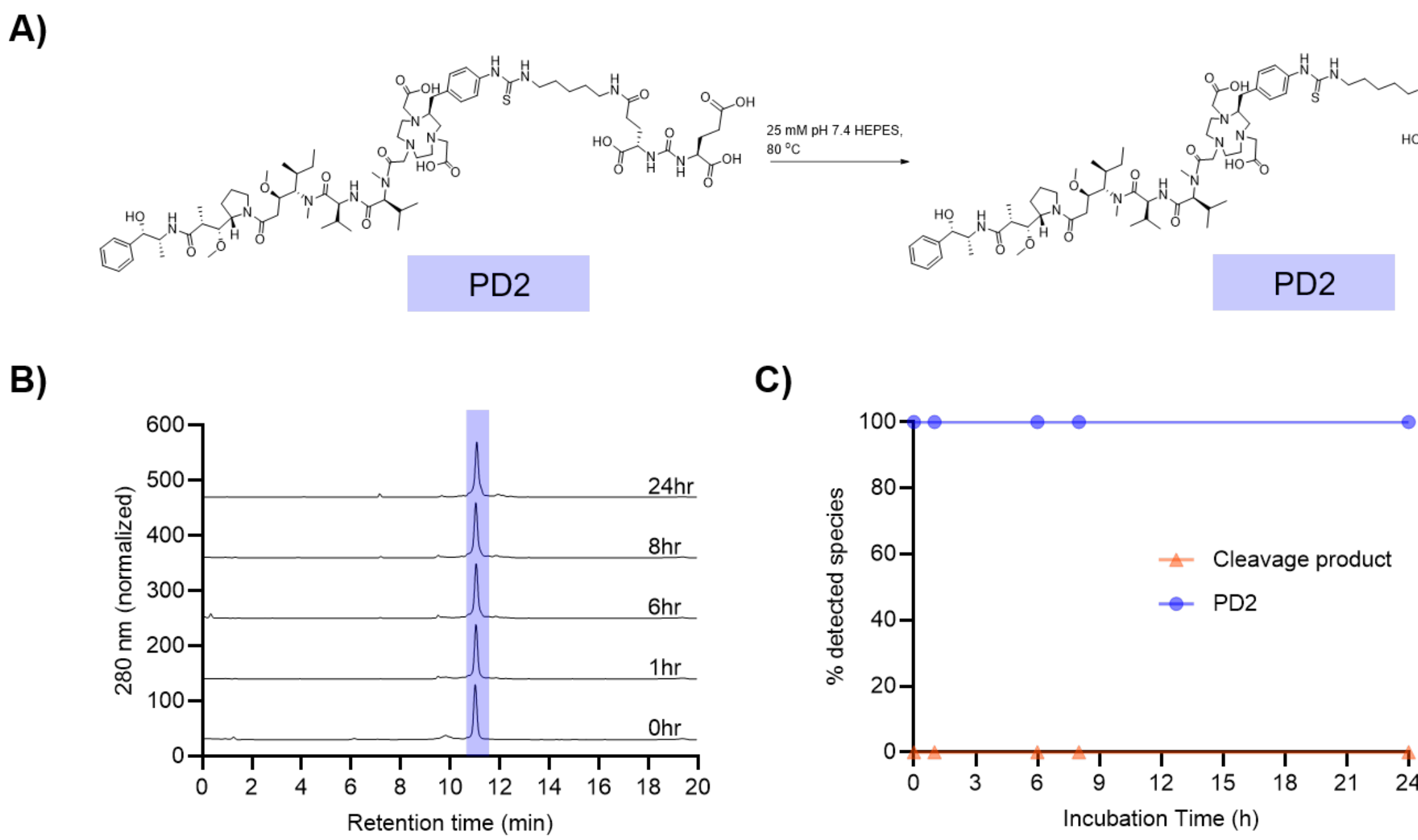

Figure S161. (A) Reaction scheme of PD2 cleavage assay. **(B)** Analytical HPLC chromatograms show no autolytic cleavage. **(C)** Quantification of cleavage at 80 °C and pH 7.4.

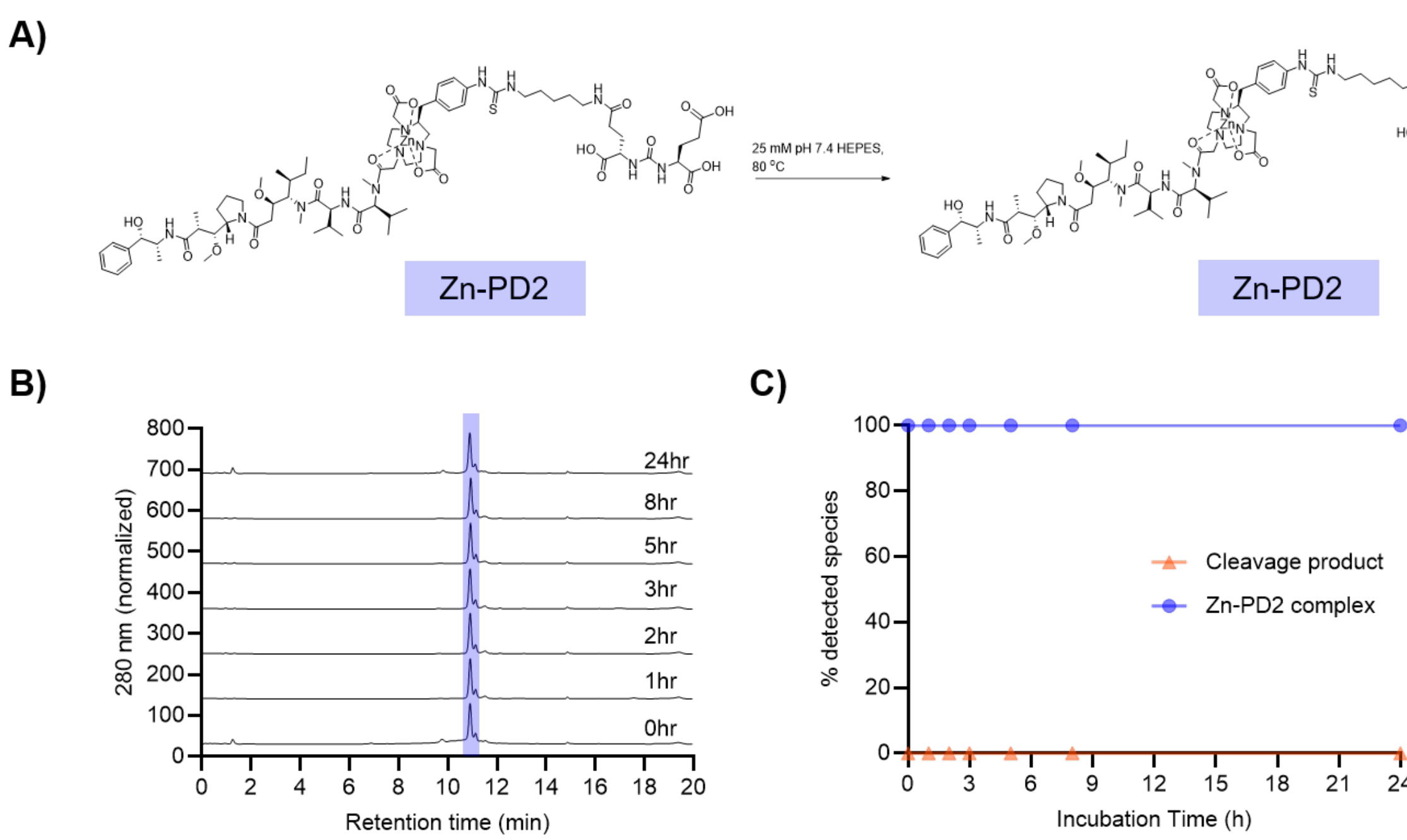

Figure S162. (A) Reaction scheme of Zn-PD2 cleavage assay. **(B)** Analytical HPLC chromatograms show no autolytic cleavage. **(C)** Quantification of cleavage at 80 °C and pH 7.4.

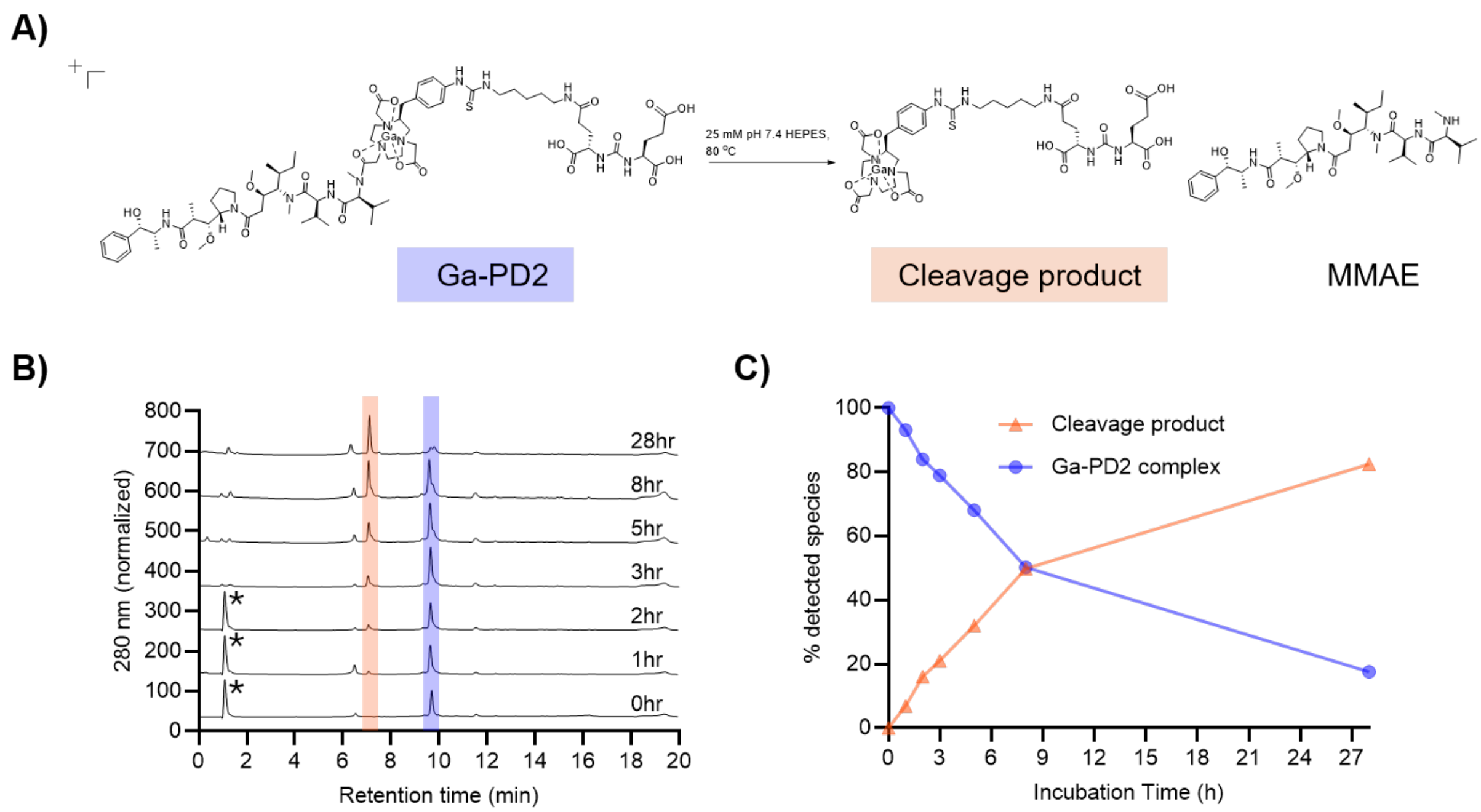

Figure S163. (A) Reaction scheme of Ga-PD2 cleavage assay. **(B)** Analytical HPLC chromatograms show the autolytic release of the cleavage product and MMAE. Asterisk (*) indicates DMSO peak. **(C)** Quantification of cleavage at 80 °C and pH 7.4.

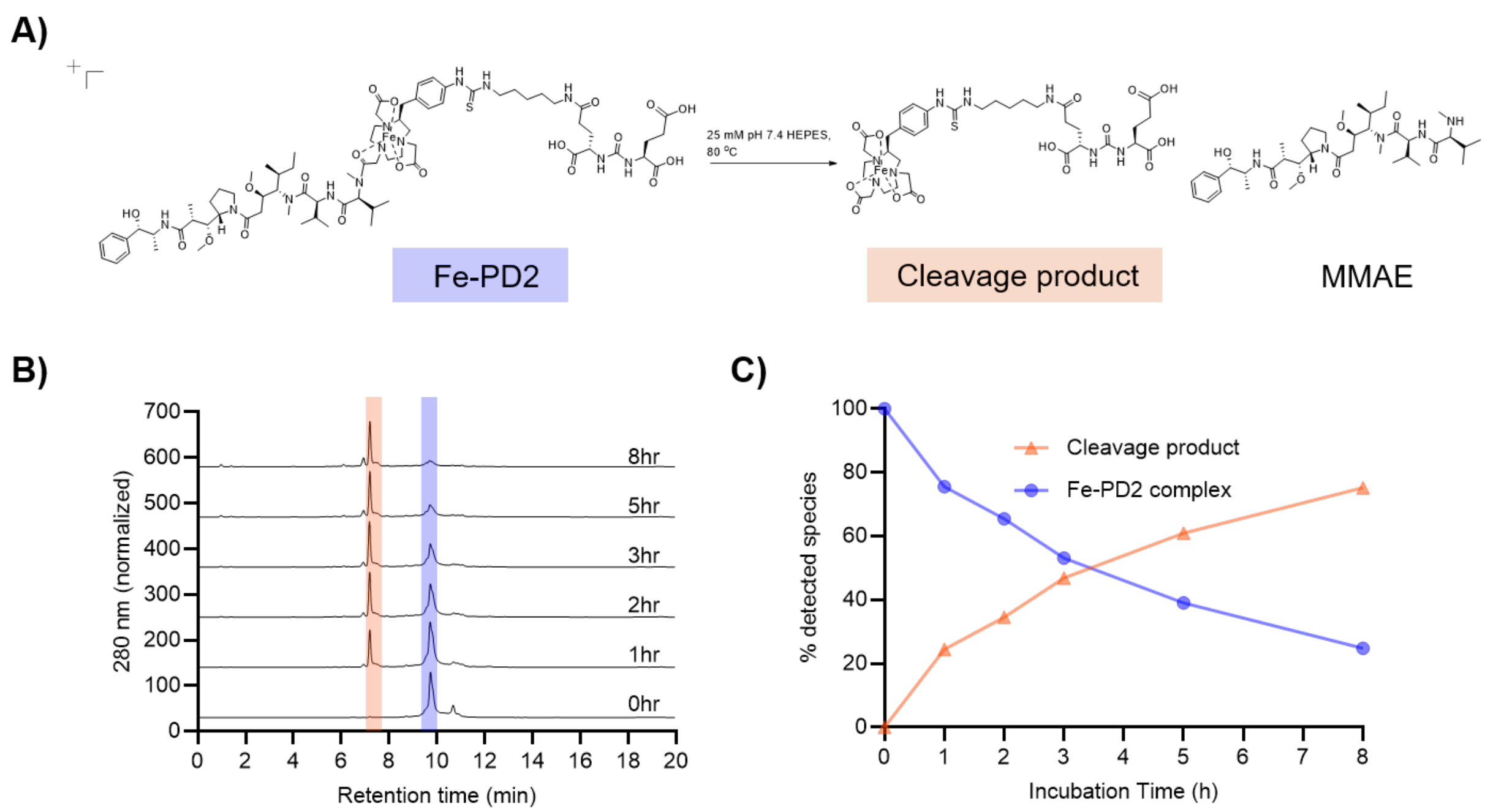

Figure S164. (A) Reaction scheme of Fe-PD2 cleavage assay. **(B)** Analytical HPLC chromatograms show the autolytic release of the cleavage product and MMAE. **(C)** Quantification of cleavage at 80 °C and pH 7.4.

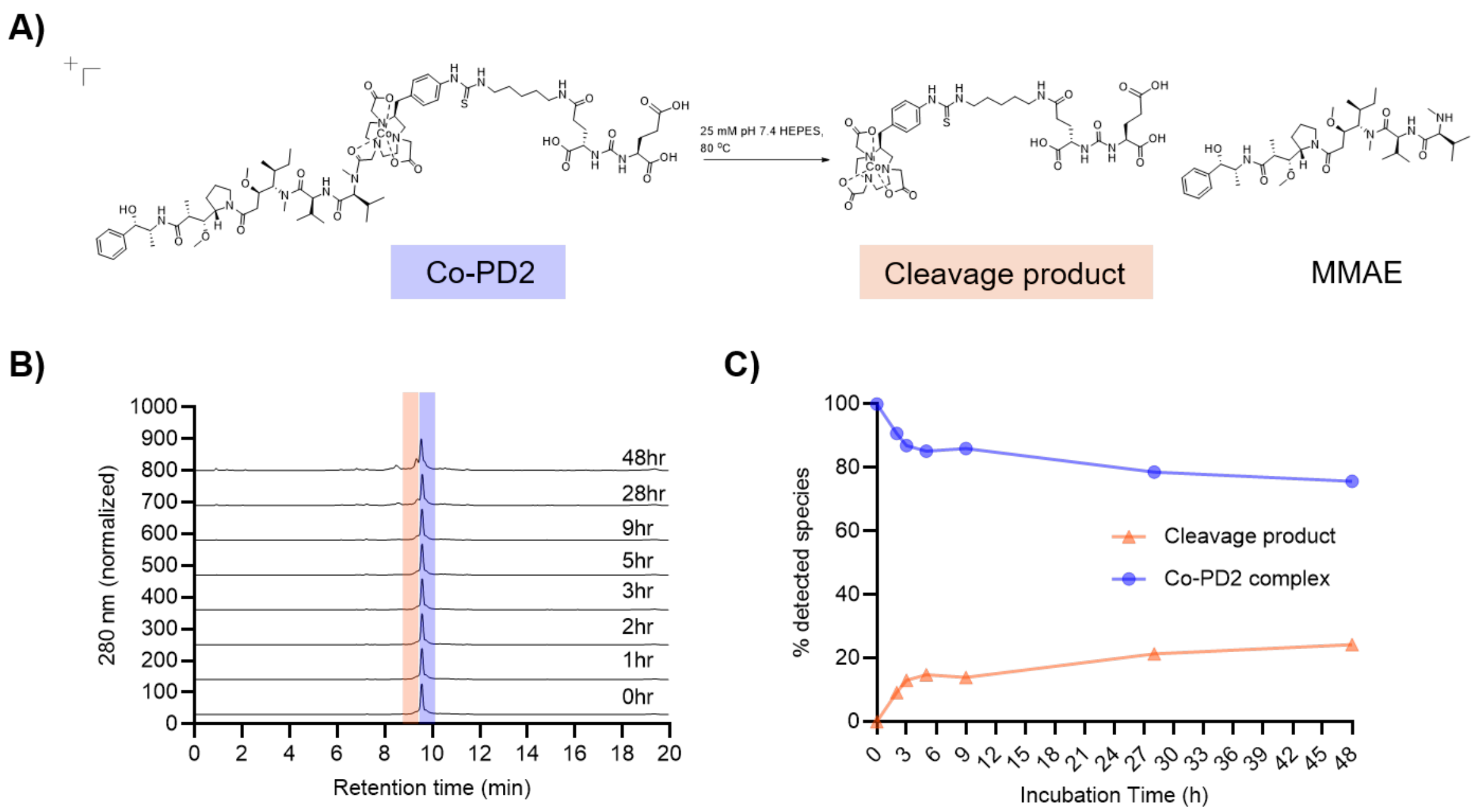

Figure S165. (A) Reaction scheme of Co-PD2 cleavage assay. **(B)** Analytical HPLC chromatograms show the autolytic release of the cleavage product and MMAE. **(C)** Quantification of cleavage at 80 °C and pH 7.4.

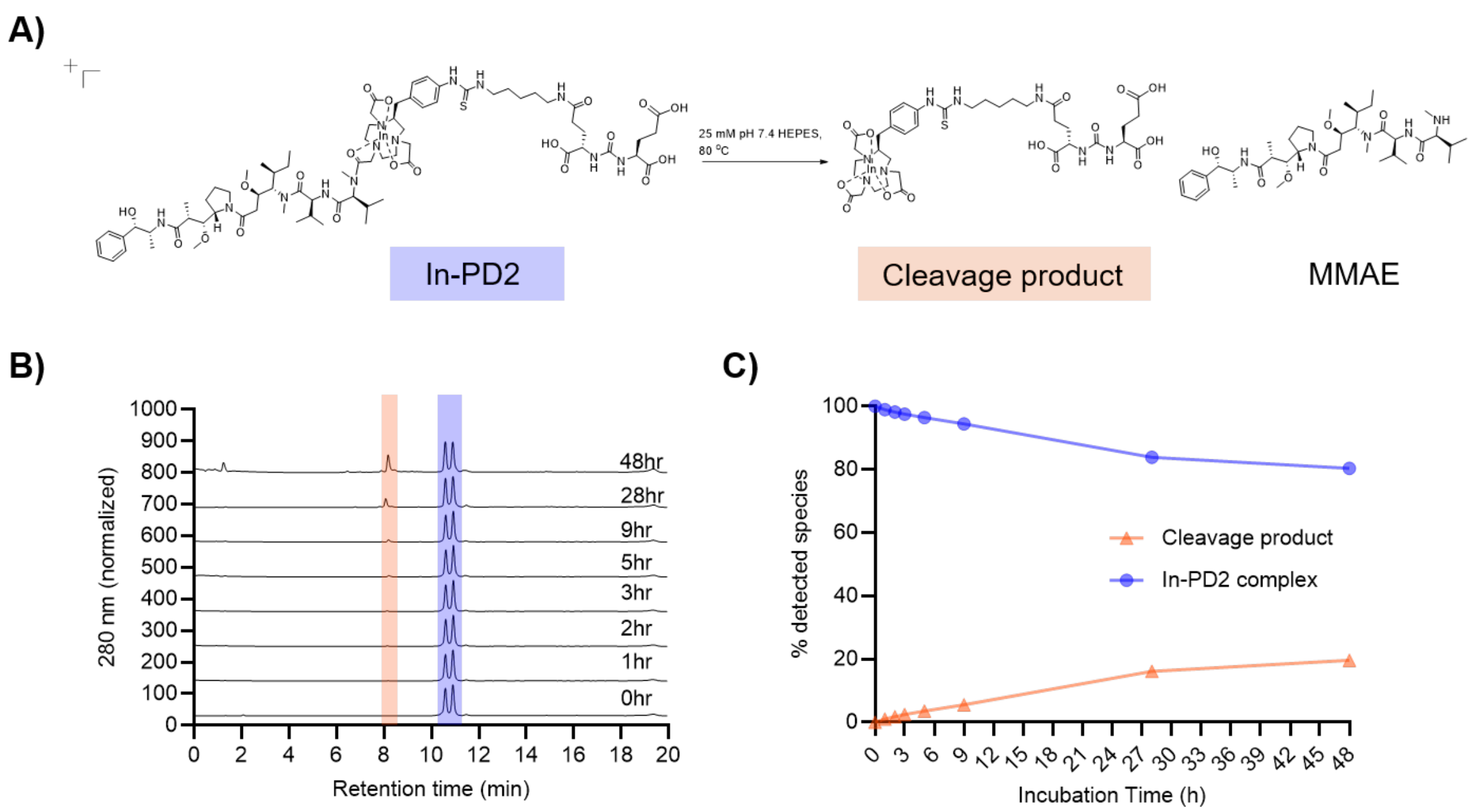

Figure S166. (A) Reaction scheme of In-PD2 cleavage assay. **(B)** Analytical HPLC chromatograms show the autolytic release of the cleavage product and MMAE. **(C)** Quantification of cleavage at 80 °C and pH 7.4.

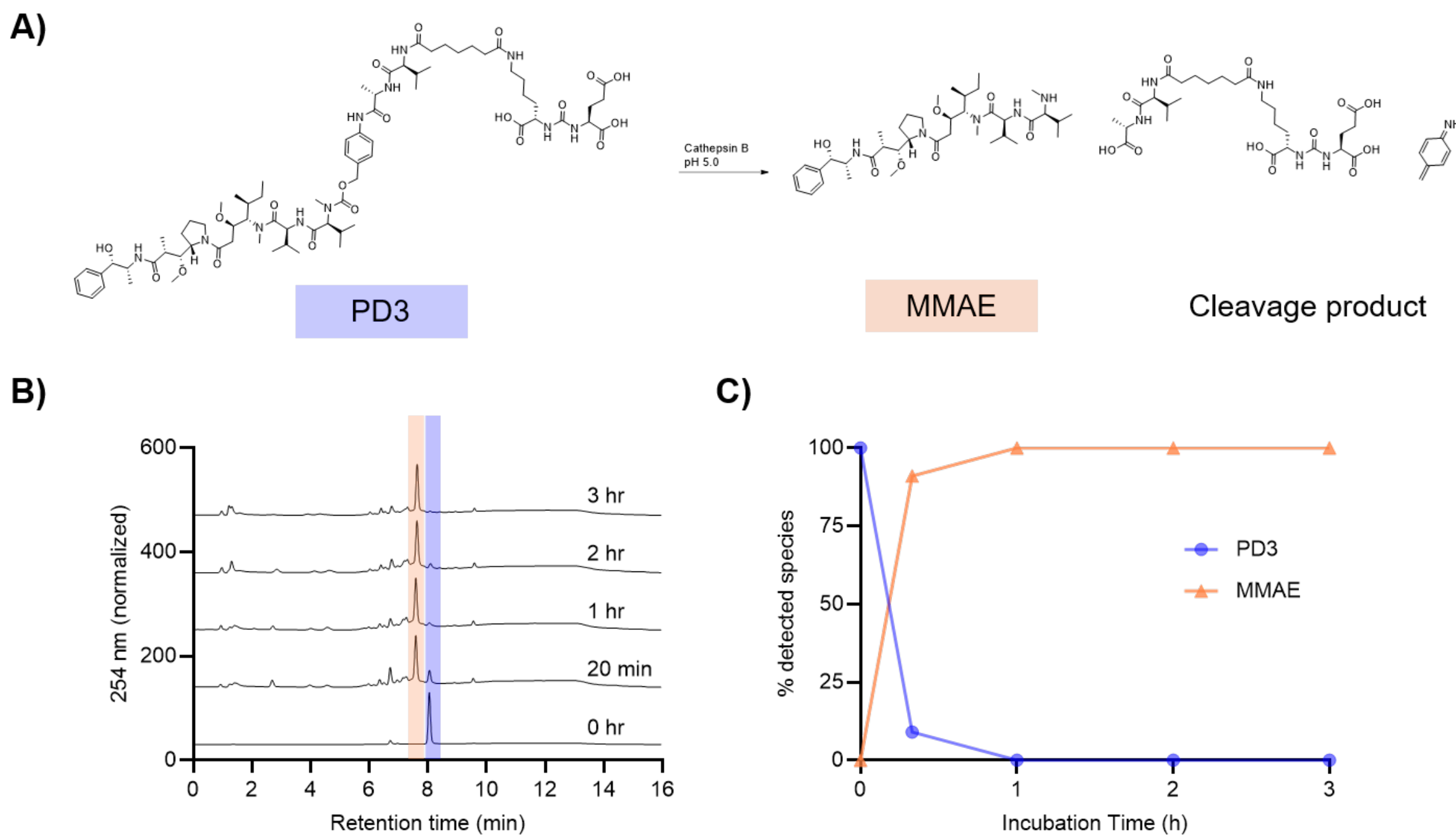

Figure S167. (A) Reaction scheme of PD3 Cathepsin B enzyme cleavage assay. **(B)** Analytical HPLC chromatograms show the autolytic release of the cleavage product and MMAE. **(C)** Quantification of cleavage at 80 °C and pH 7.4.

6.4 Ultraviolate-Visible Spectroscopy Data of PD1 and PD2

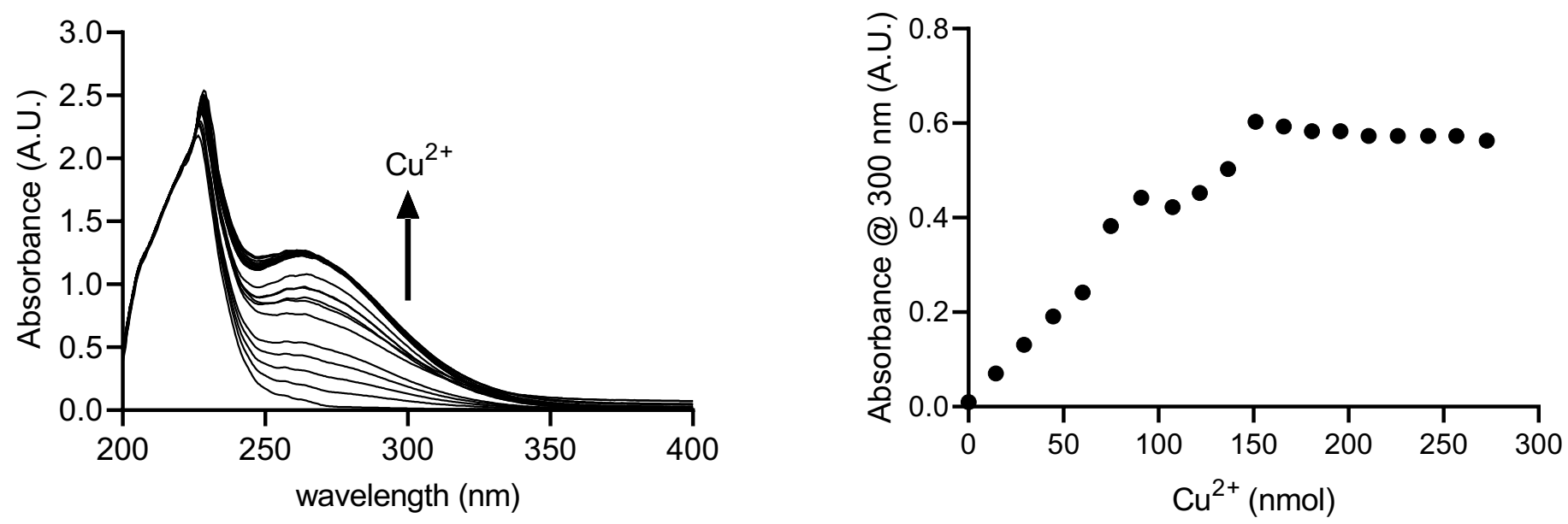

Figure S168. Spectrophotometric titration of PD1 conjugate with Cu^{2+}. UV-vis absorbance spectra of PD1 upon Cu^{2+} addition (left), UV-vis titration to endpoint to determine ligand concentration (right).

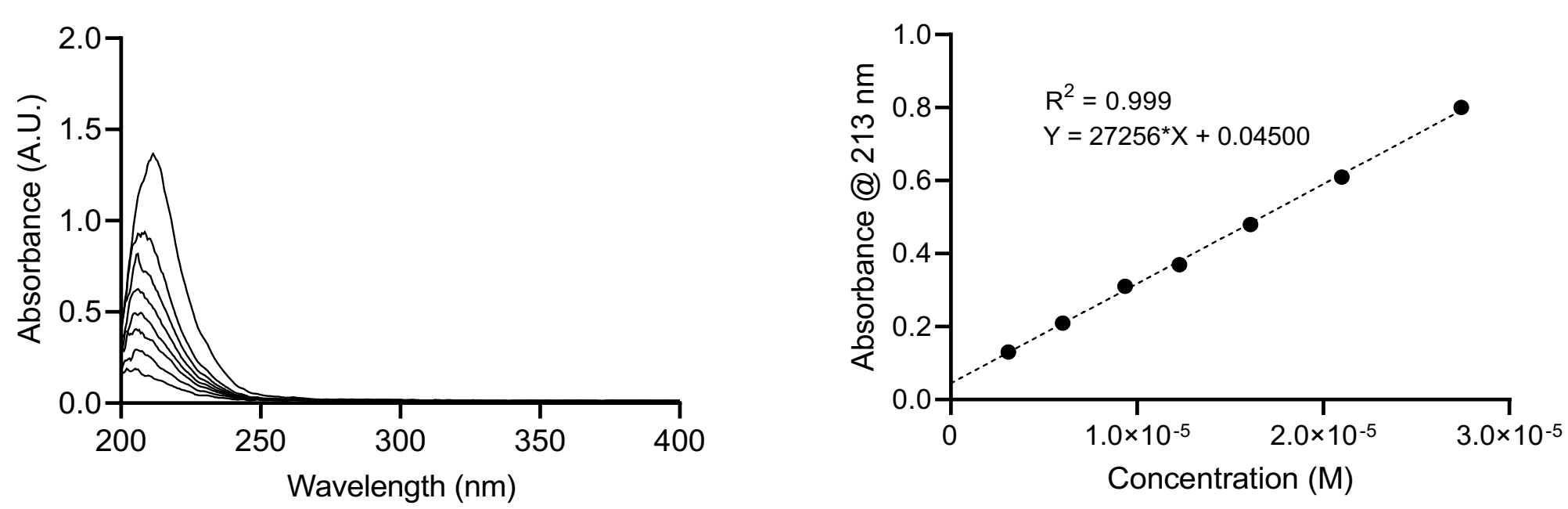

Figure S169. UV-vis analysis of PD1 to determine the molar extinction coefficient. UV-vis absorbance spectra of PD1 at decreasing concentrations (left) and analysis of spectra at 213 nm to determine the extinction coefficient (right).

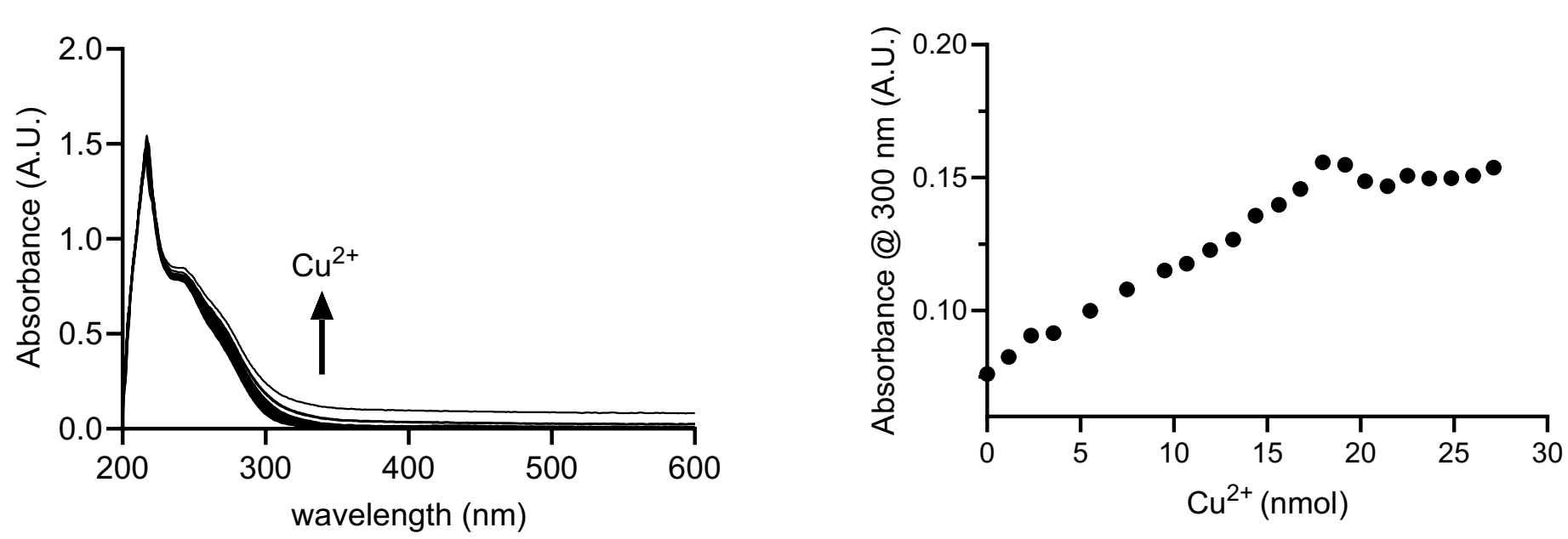

Figure S170. Spectrophotometric titration of PD2 conjugate with Cu^{2+}. UV-vis absorbance spectra of PD2 upon Cu^{2+} addition (left), UV-vis titration to endpoint to determine ligand concentration (right).

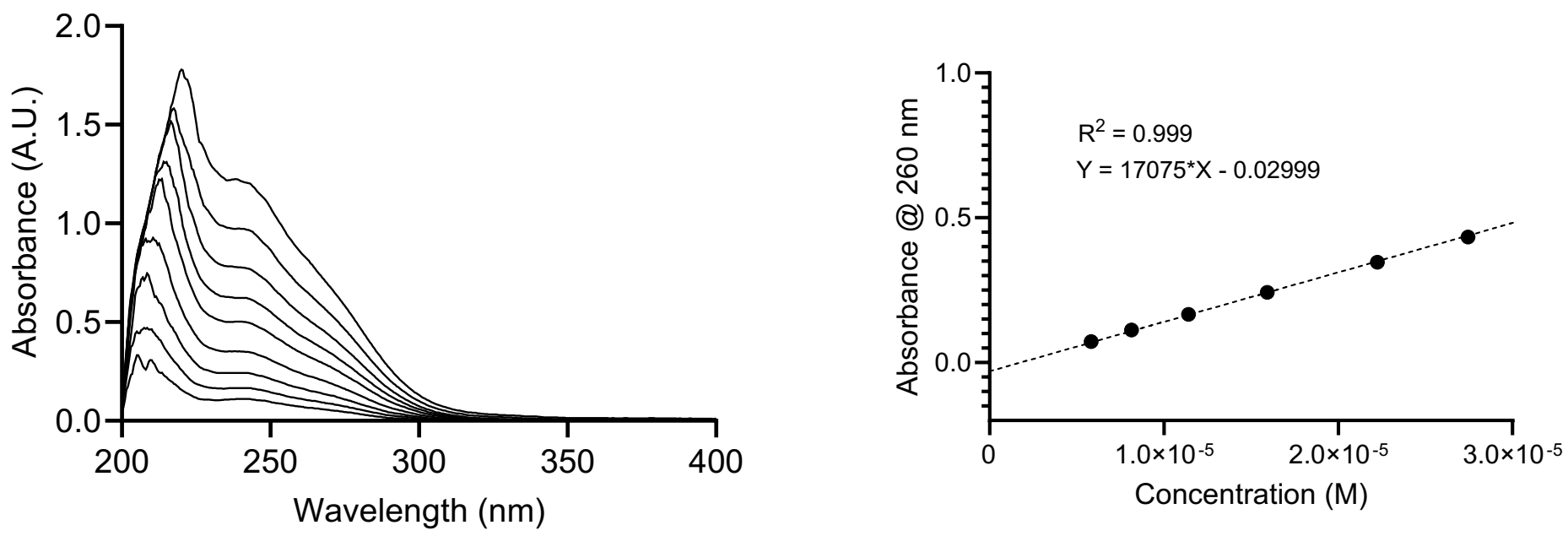

Figure S171. UV-vis analysis of PD2 to determine the molar extinction coefficient. UV-vis absorbance spectra of PD2 at decreasing concentrations (left) and analysis of spectra at 213 nm to determine the extinction coefficient (right).

7 In vitro and in vivo characterization

7.1 Cell viability studies

The 3-(4,5-dimethylthiazol-2-yl)-2,5-diphenyltetrazolium bromide (MTT) assays were carried out for the PD1 conjugate to assess drug potency towards HeLa cells. 96-well plates were seeded with 3000 cells per well (100 µL) and incubated for 24 hours in DMEM supplemented with 10% FBS and 5% streptomycin. A dilution series of metal complexes in sterile PBS was added, and cells were incubated for 72 hours. The cell media was aspirated and replenished in each well with 100 µL fresh media. MTT dye in PBS (5 mg/mL, 10 µL) was added to each well. Cells were incubated for 5 hours. All liquids were removed and 100 µL DMSO was added to each well. The absorbance at 540 nm was measured. Each 96-well plate contained 3 replicates for each concentration. MTT assays for PD2 conjugates were performed using same procedure, with PC3-Pip cell line.

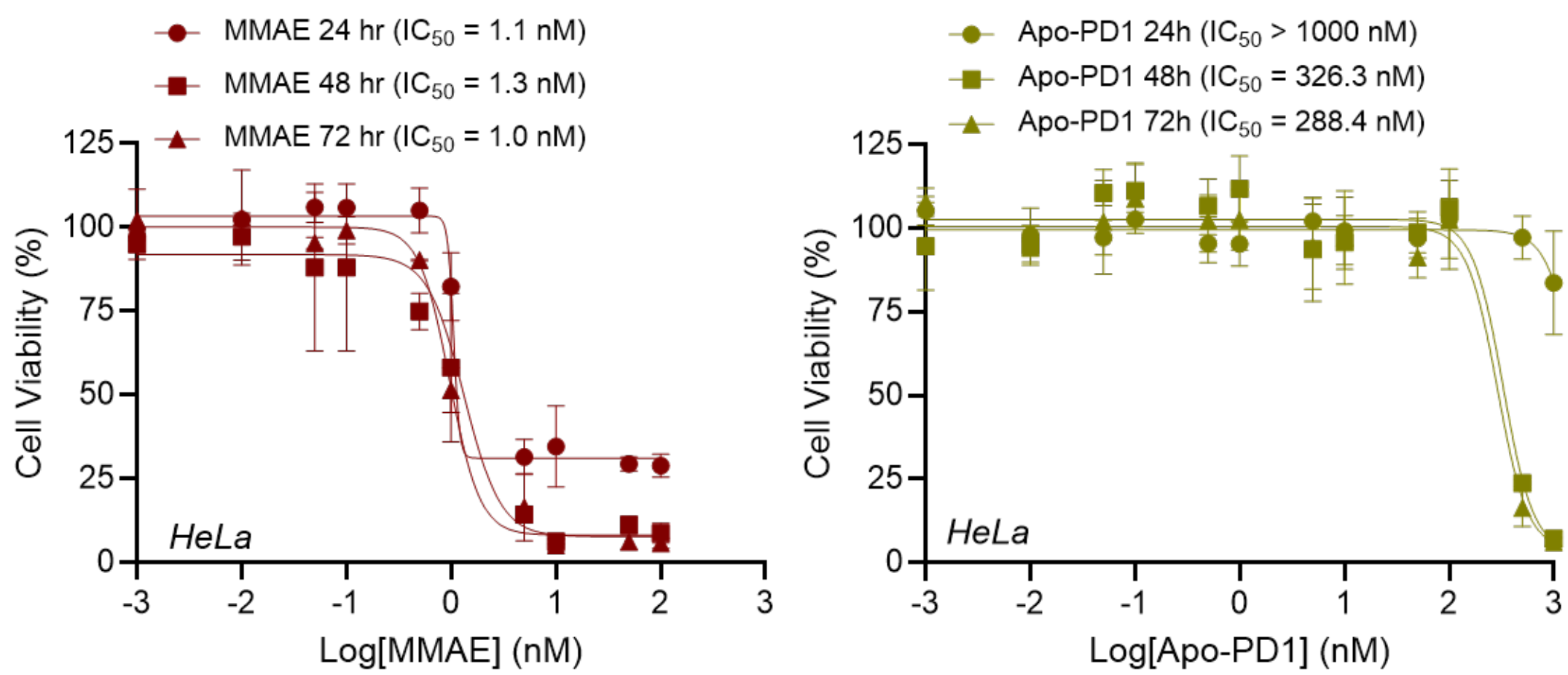

Figure S172. Cytotoxicity assessment in HeLa cells exposed to increasing concentrations of MMAE (left) and apo-PD1 (right) for 24, 48, and 72 hours incubation.

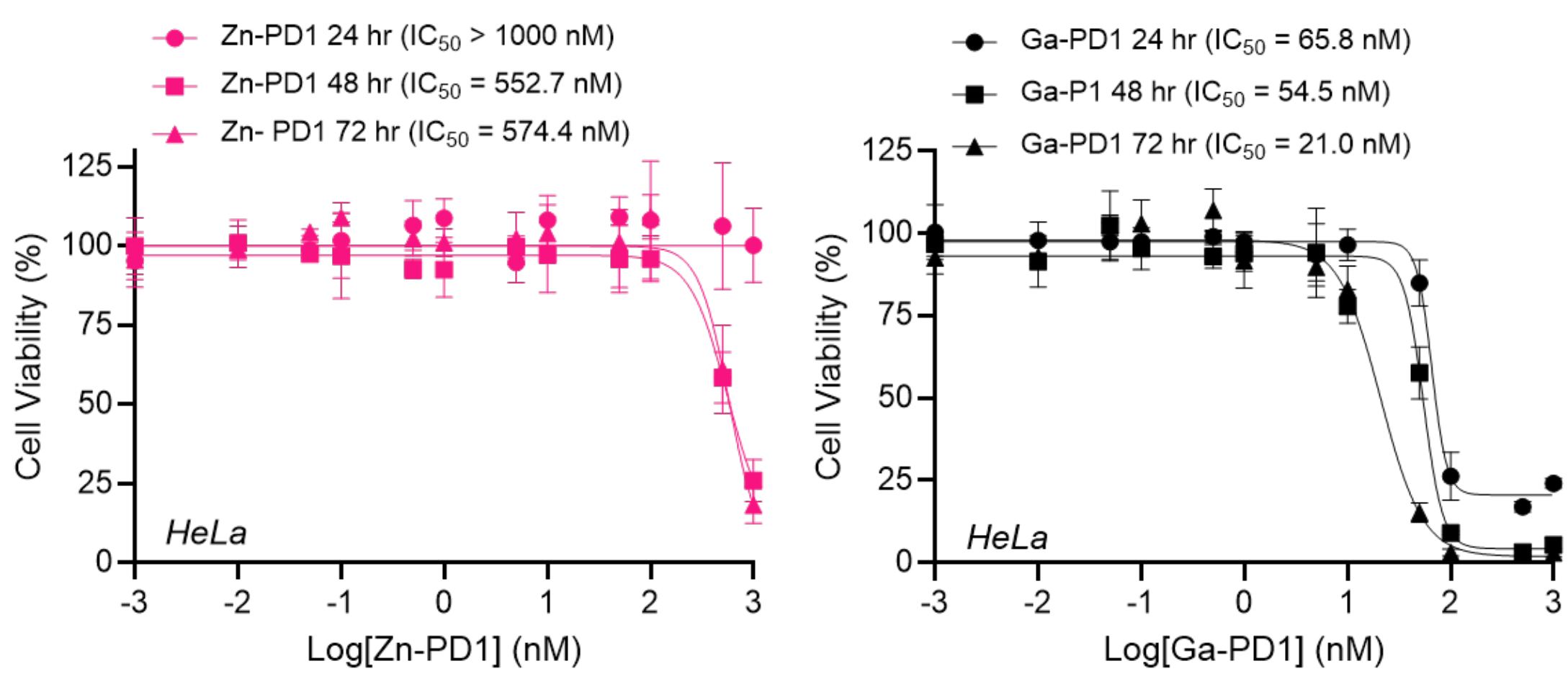

Figure S173. Cytotoxicity assessment in HeLa cells exposed to increasing concentrations of Zn-PD1 (left) and Ga-PD1 (right) for 24, 48, and 72 hours incubation.

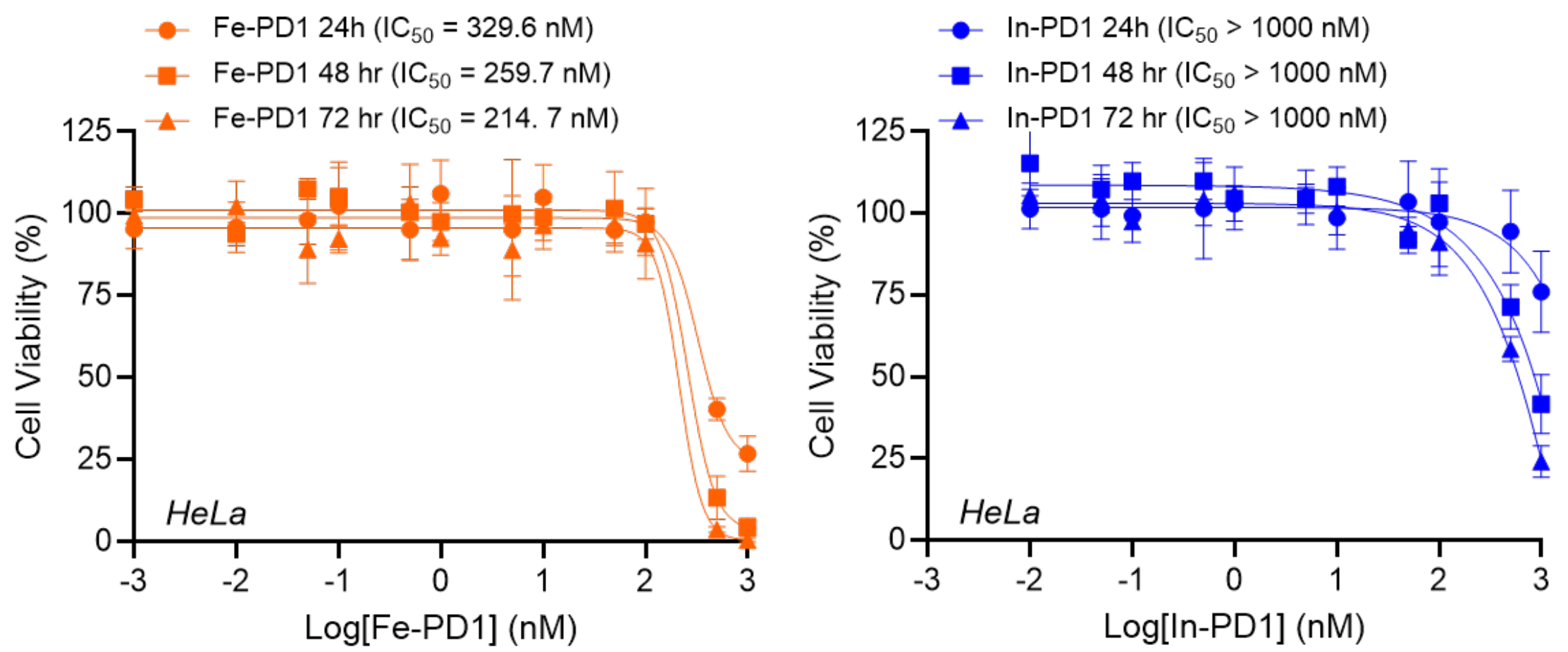

Figure S174. Cytotoxicity assessment in HeLa cells exposed to increasing concentrations of Fe-PD1 (left) and In-PD1 (right) for 24, 48, and 72 hours incubation.

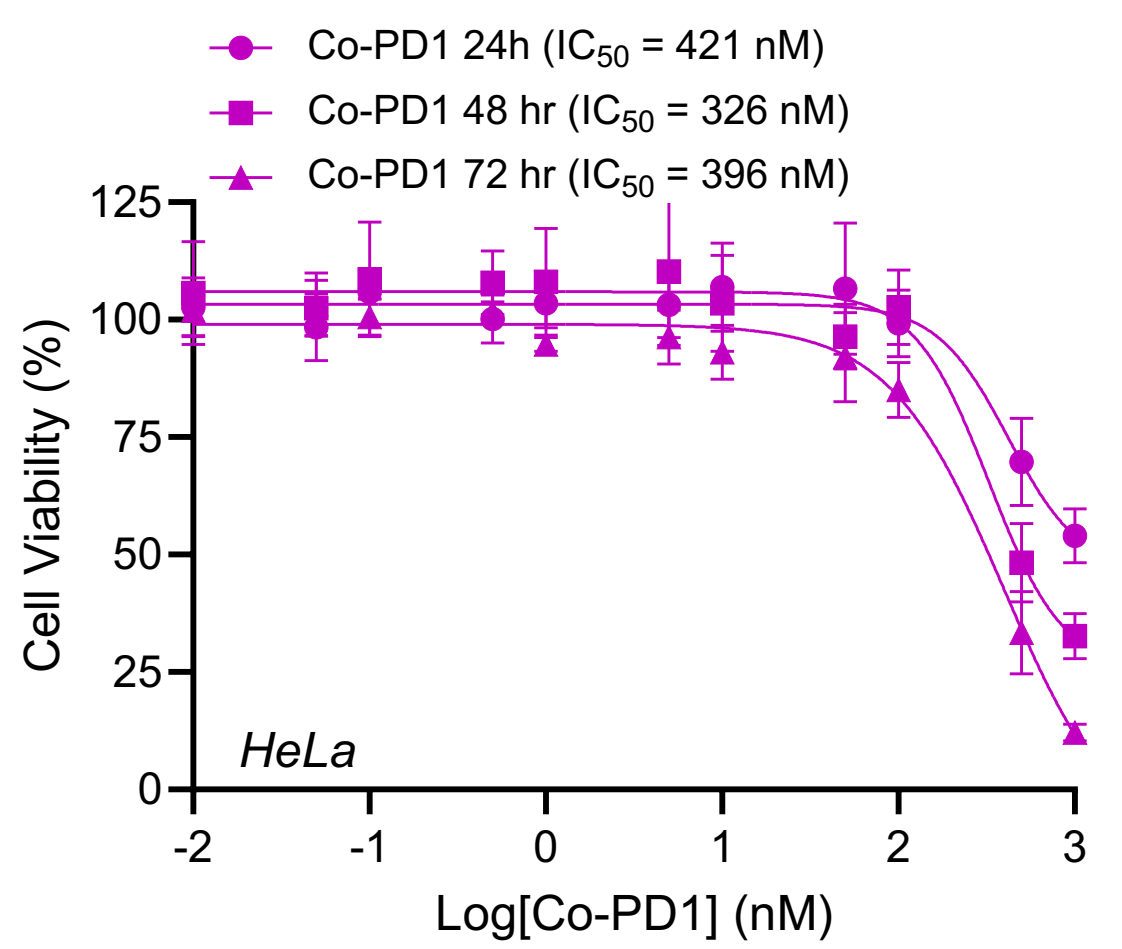

Figure S175. Cytotoxicity assessment in HeLa cells exposed to increasing concentrations of Co-PD1 for 24, 48, and 72 hours incubation.

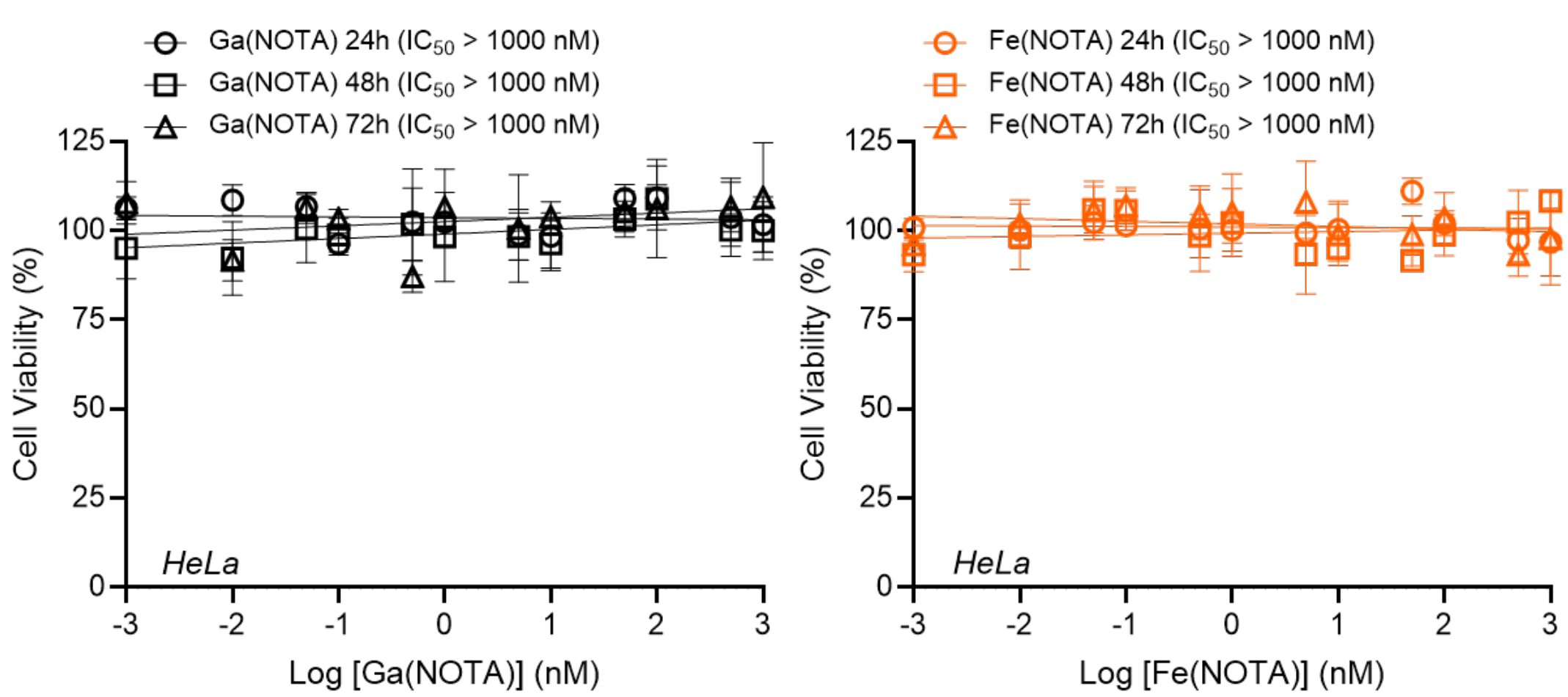

Figure S176. Cytotoxicity assessment in HeLa cells exposed to increasing concentrations of Ga(NOTA) complex (left) and Fe(NOTA) complex (right) for 24, 48, and 72 hours incubation.

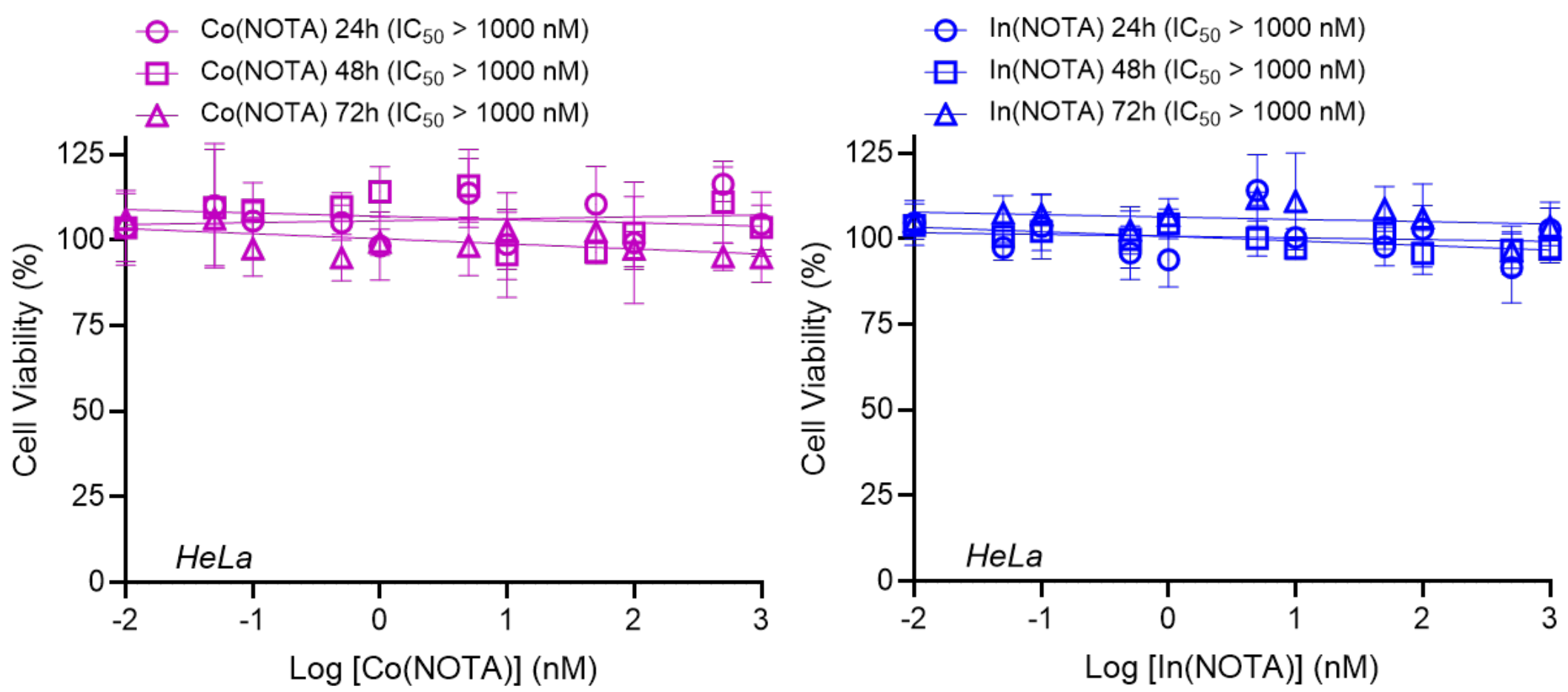

Figure S177. Cytotoxicity assessment in HeLa cells exposed to increasing concentrations of Co(NOTA) complex (left) and In(NOTA) complex (right) for 24, 48, and 72 hours incubation.

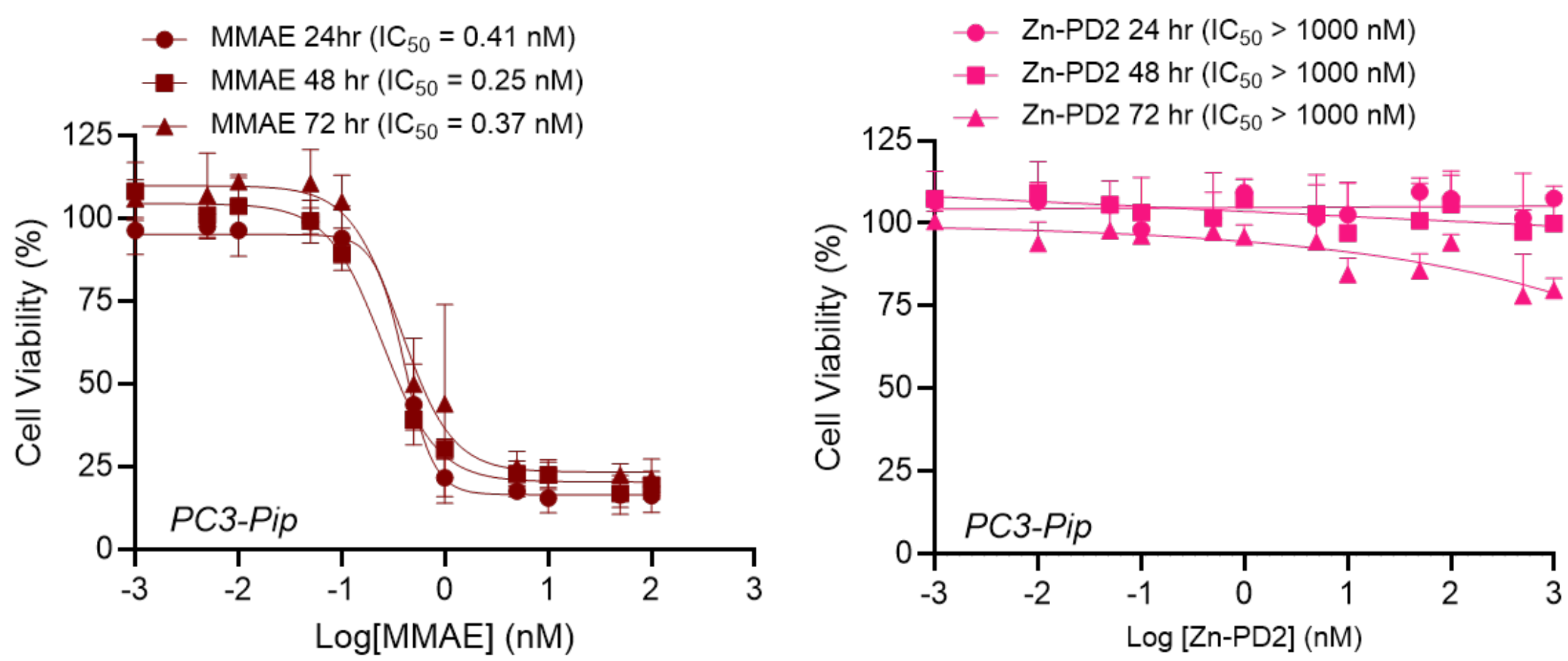

Figure S178. Cytotoxicity assessment in PC3-pip cells exposed to increasing concentrations of MMAE (left) and Zn-PD2 (right) for 24, 48, and 72 hours.

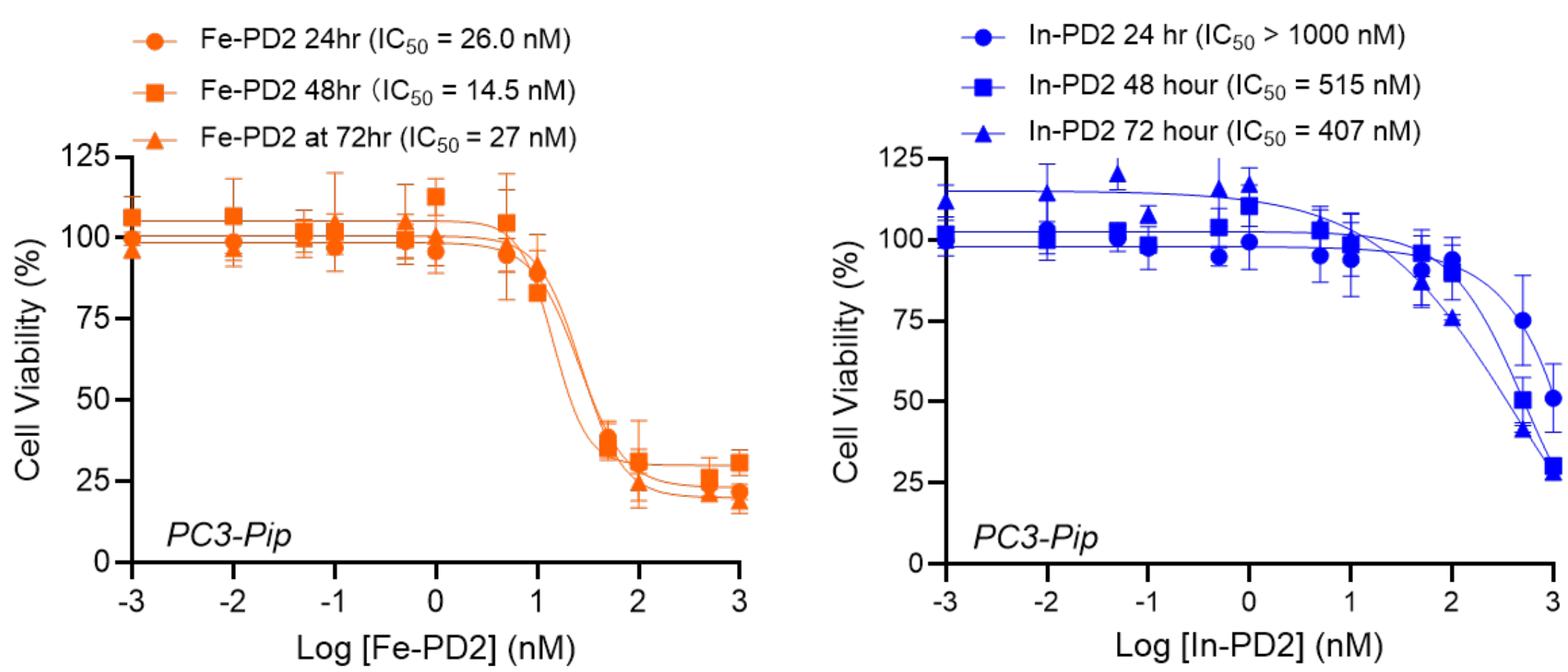

Figure S179. Cytotoxicity assessment in PC3-pip cells exposed to increasing concentrations of Fe-PD2 (left) and In-PD2 (right) for 24, 48, and 72 hours.

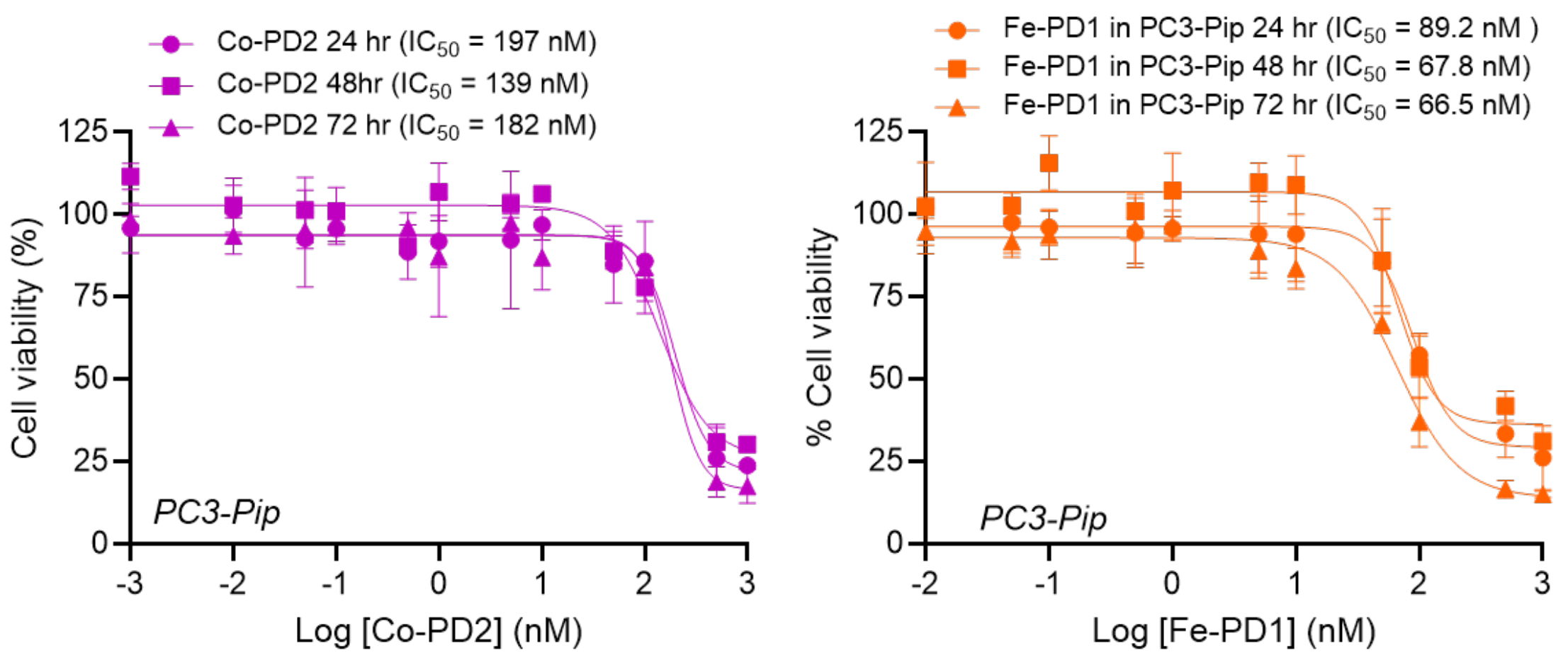

Figure S180. Cytotoxicity assessment in PC3-pip cells exposed to increasing concentrations of Co-PD2 (left) and Fe-PD1 (right) for 24, 48, and 72 hours.

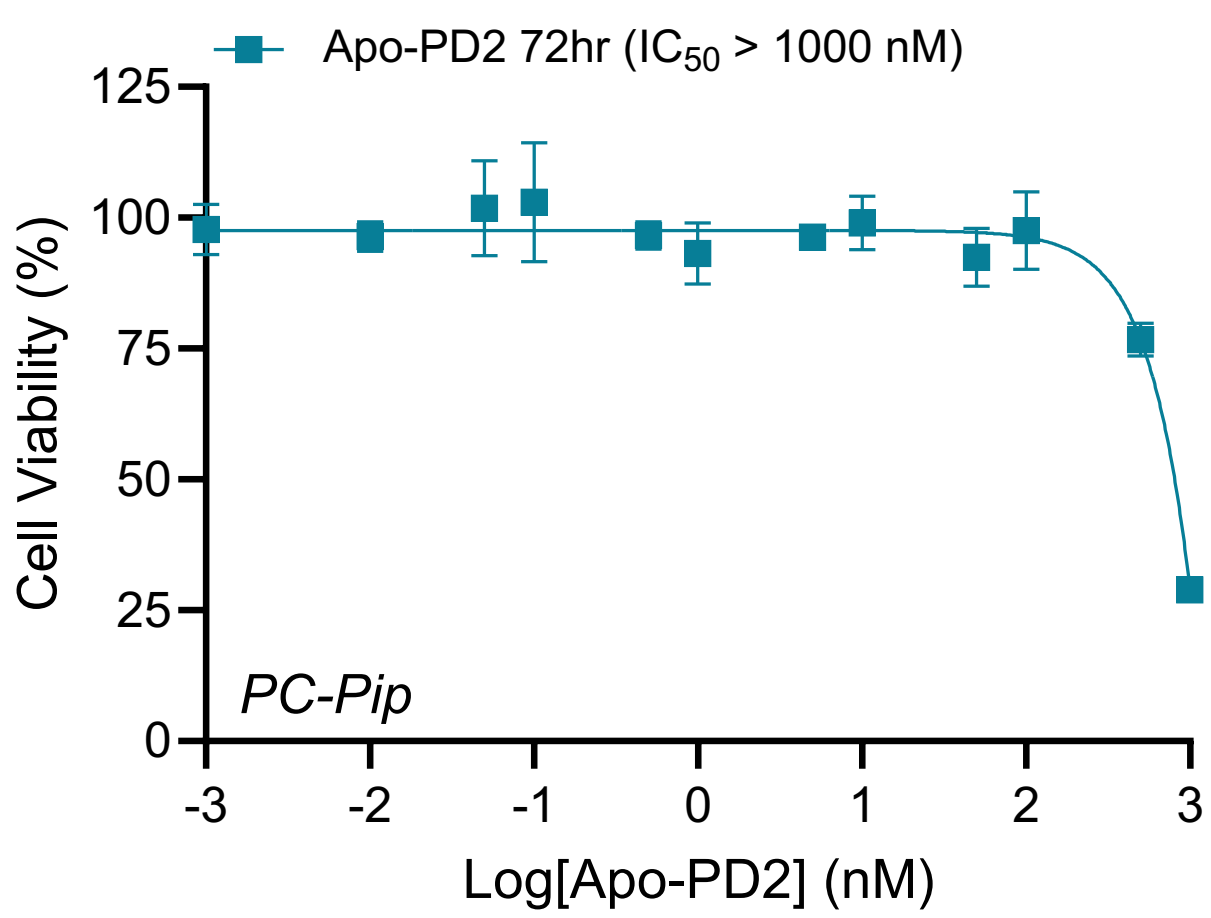

Figure S181. Cytotoxicity assessment in PC3-pip cells exposed to increasing concentrations of apo-PD2 for 72 hours.

7.2 Tumor xenograft model

All animal experiments were conducted with the approval of the University of Wisconsin-Madison Institutional Animal Care and Use Committee (IACUC). All studies were conducted in accordance with the relevant guidelines and regulations and approved under protocol number M006738 (PI: Boros) and conducted at UW-Madison School of Medicine and Public Health, at the Small Animal Imaging and Radiotherapy Facility (SAIRF).

Positron emission tomography/computed tomography (PET/CT) imaging: PET/CT imaging was performed using a Siemens Inveon Hybrid MicroPET/CT Scanner (Siemens Medical Solutions USA, Inc., Knoxville, TN). Mice were anesthetized with 4% isoflurane gas and anesthesia was maintained during scans at 2% isoflurane in oxygen. CT scans were acquired prior to PET scans for anatomical coregistration as well as attenuation correction. CT scan parameters were as follows: 220 rotation degrees, 120 rotation steps, binning factor of 4, exposure time of 250 ms, x-ray energy of 80 kVp, 1 mA current, and 105 µm resolution. PET scans were acquired with 40 million coincidence events per mouse, an energy window of 350-650 keV, and a timing window of 3.432 ns. Quantification of PET/CT images was performed in an Inveon Research Workstation and data is expressed as percent injected dose per gram of tissue (% ID/cc).

7.3 ^{68}Ga Radiolabeling Procedure

An [^{68}Ga]$GaCl_3$ (600 µCi, 35 µL) solution was added to a mixture of NaOAc buffer (0.1 M, pH 4.5, 100 µL) and a ligand stock (PD1 and PD2, 10 nmol) of known concentration as determined UV-vis spectroscopy solution in H_2O. Then, the mixture was incubated at room temperature for 15 minutes. The radiochemical yield of [^{68}Ga][Ga-PD2] was evaluated by radio-HPLC. Rt (Method F): 9.02 min. RCY: 84.74%, RCP: > 99%.

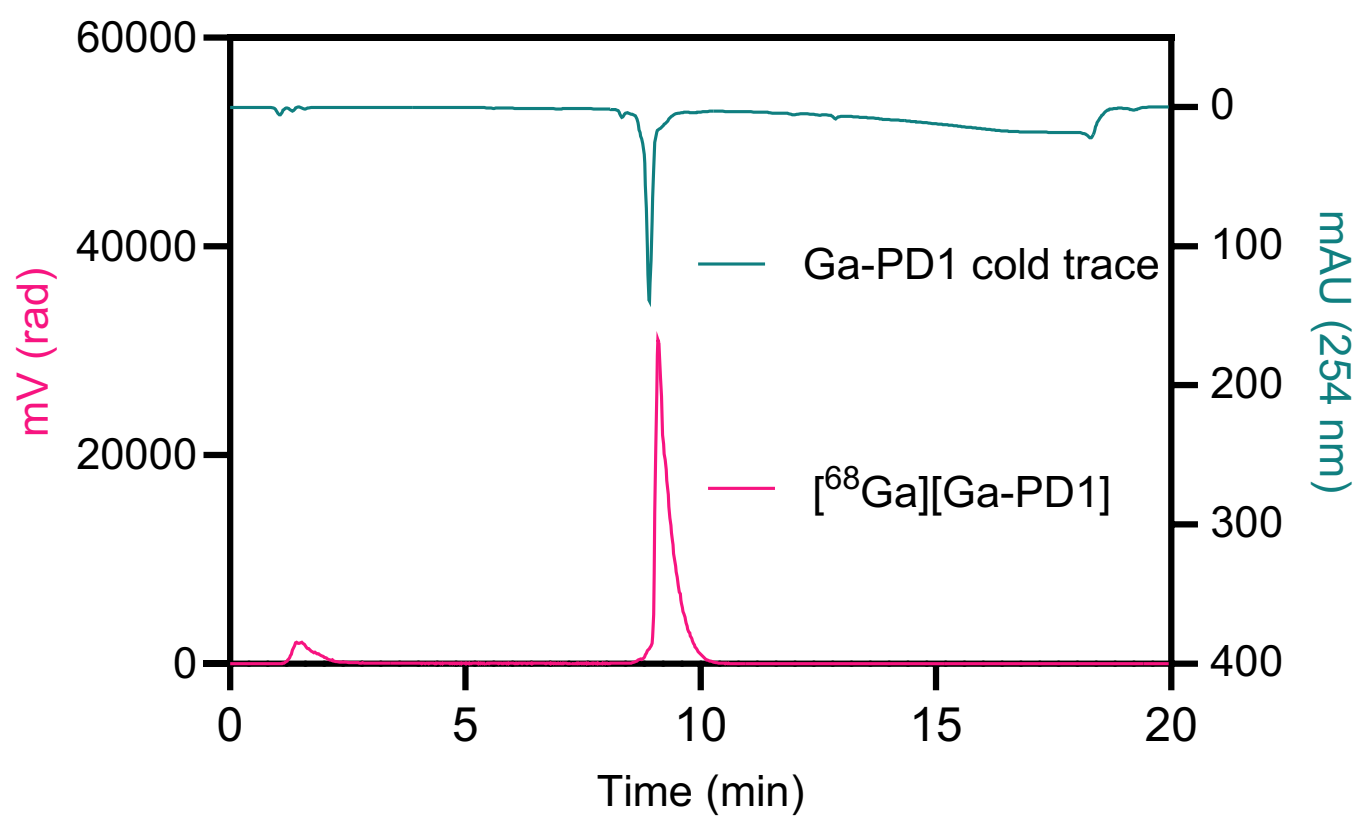

Figure S182. Radio-HPLC traces of Ga-PD1 cold complex and [^{68}Ga][Ga-PD2] radiolabeled complex.

[^{68}Ga][Ga-PD2]. Compound **[^{68}Ga][Ga-PD2]** was synthesized using the general radiolabeling protocol for compound **PD2**. The product was characterized using radio-HPLC chromatography. R_t (Method F): 9.12 min. RCY: > 86.21%, RCP: > 99%.

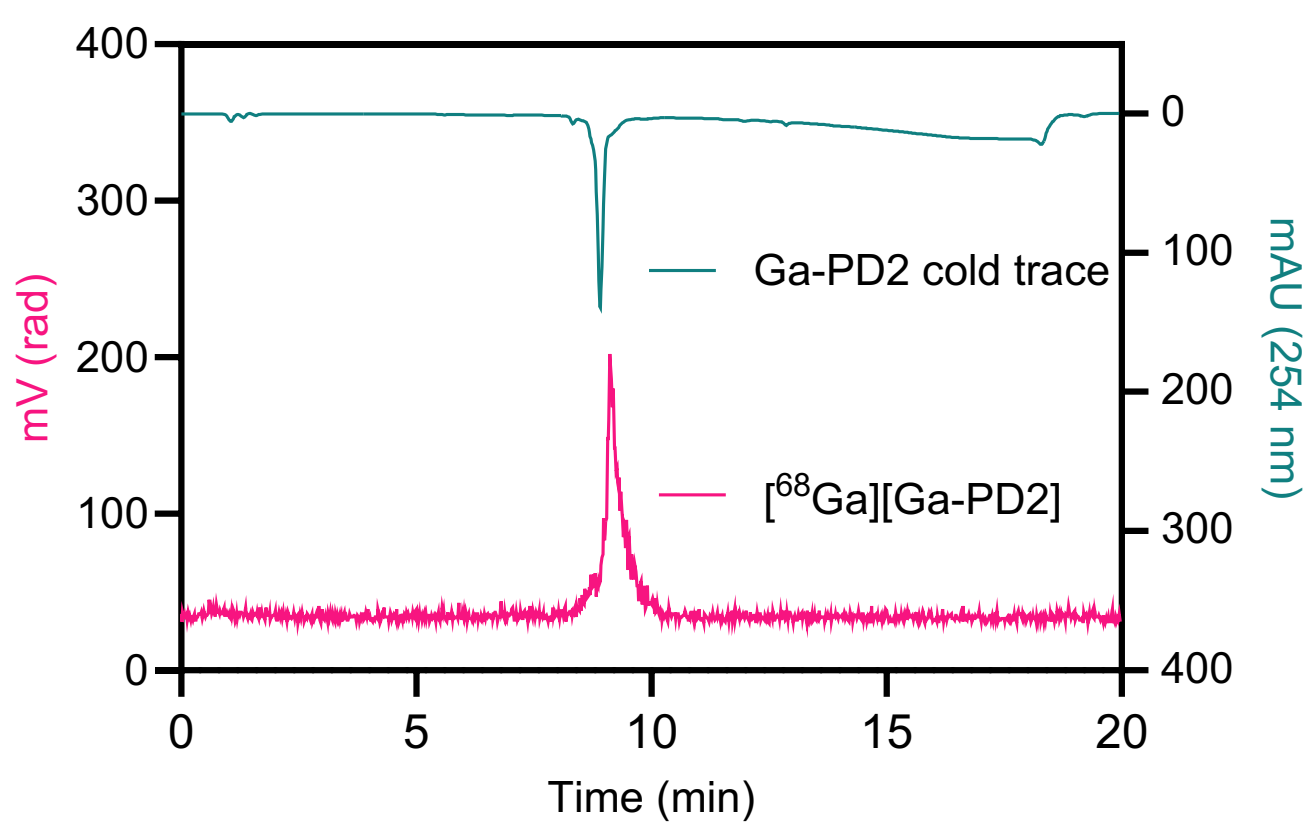

Figure S183. Radio-HPLC traces of Ga-PD2 cold complex and [^{68}Ga][Ga-PD2] radiolabeled complex.

7.4 ^{111}In Radiolabeling Procedure

An [^{111}In]$InCl_3$ (750 µCi, 10 µL) solution was added to a mixture of NH_4OAc buffer (0.1 M, pH 5.5, 100 µL) and a ligand stock (PD2, 4 nmol) of known concentration as determined UV-vis spectroscopy solution in H_2O. Then, the mixture was incubated at room temperature for 15 minutes. The radiochemical yield of [^{111}In][In-PD2] was evaluated by radio-HPLC. Rt (Method F): 9.29 min. RCY: 98.28%. RCP: 98.28%.

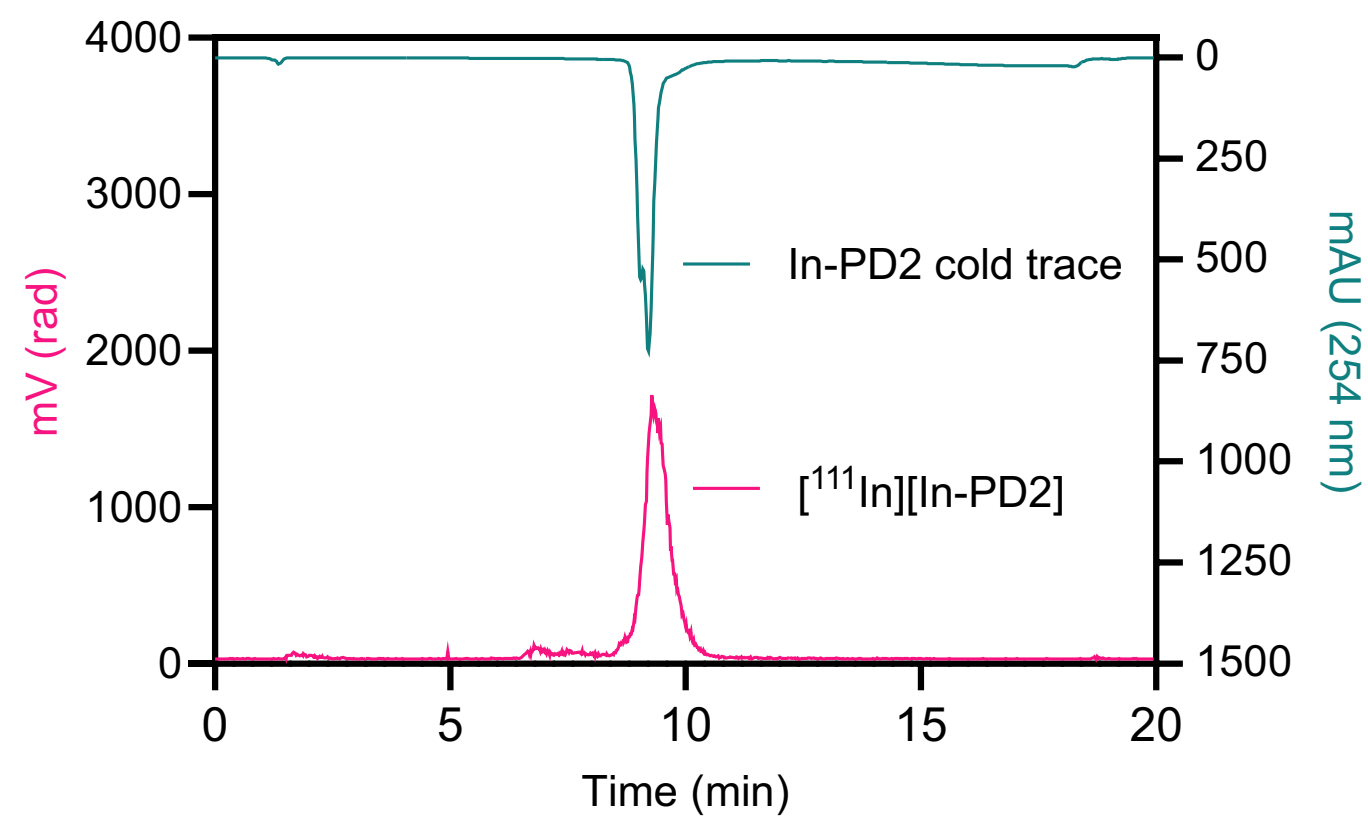

Figure S184. Radio-HPLC traces of In-PD2 cold complex and [^{111}In][In-PD2] radiolabeled complex.

7.5 ^{111}In ^{NMe}Gly-Gly-Trp Radiolabeling Procedure

An [^{111}In]$InCl_3$ (750 µCi, 10 µL) solution was added to a mixture of NH_4OAc buffer (0.1 M, pH 5.5, 100 µL) and a ligand stock (^{NMe}Gly-NO2A, 10 nmol) of known concentration as determined UV-vis spectroscopy solution in H_2O. Then, the mixture was incubated at room temperature for 15 minutes. The radiochemical yield of [^{111}In][In(NO2A)]$^+$-^{NMe}G-G-W was evaluated by radio-HPLC. R_t (Method F): 6.73 min. RCY: 90.0%, RCP: > 99%.

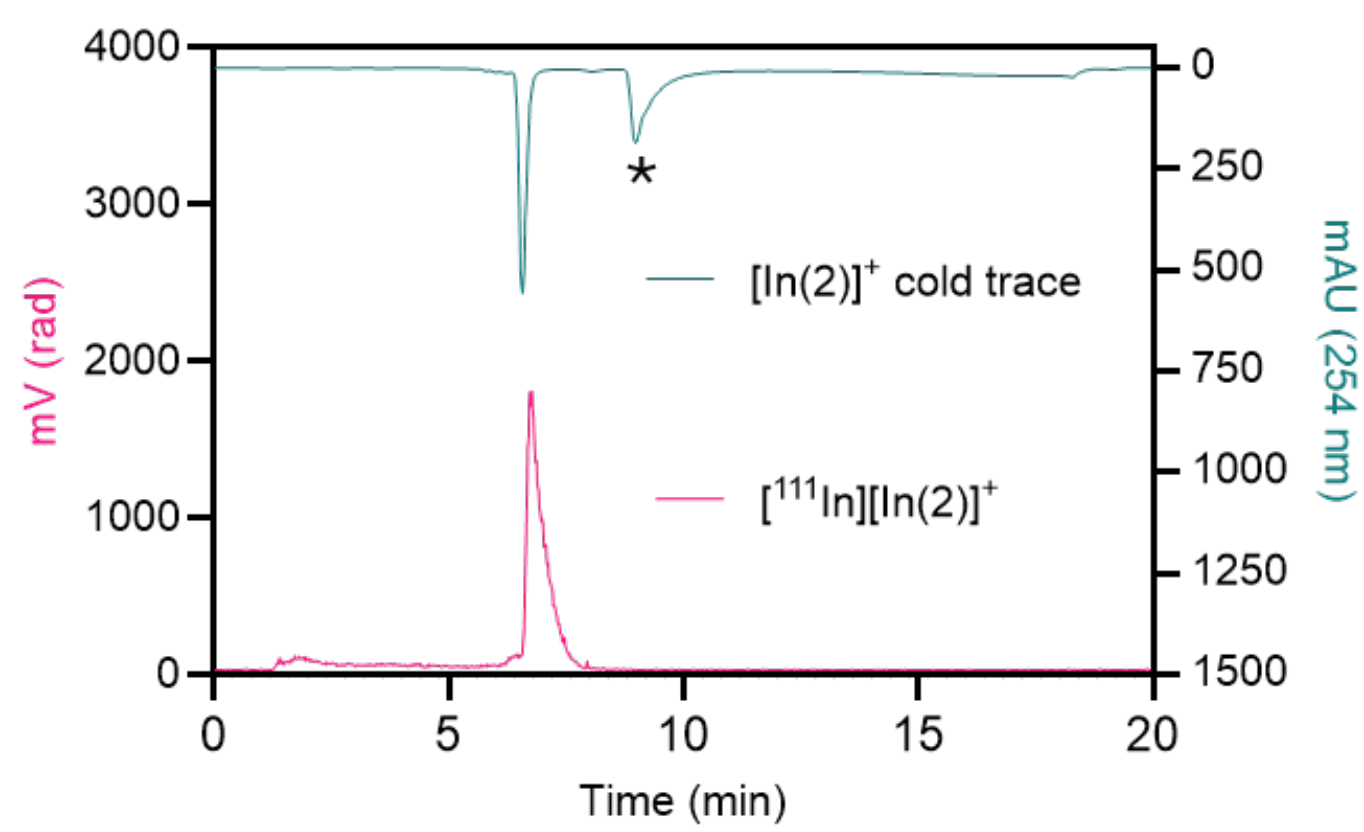

Figure S185. Radio-HPLC traces of [In(NO2A)]$^+$-^{NMe}G-G-W cold complex and [^{111}In][In(NO2A)]$^+$-^{NMe}G-G-W radiolabeled complex. Asterisk (*) indicates the impurity from the HPLC column.

7.6 Chloride ion challenge assay on [^{111}In][In(NO2A)]$^{+}$-^{NMe}G-G-W

For the chloride ion challenge assay, aliquots of the radiolabeled complex were buffered to pH 7.4 (25 mM pH 7.4 HEPES buffer) and treated with NaCl to final concentrations of 10, 100, or 1000 mM. Cleavage assays were conducted at room temperature, and formation of the cleavage product was monitored by radio-HPLC.

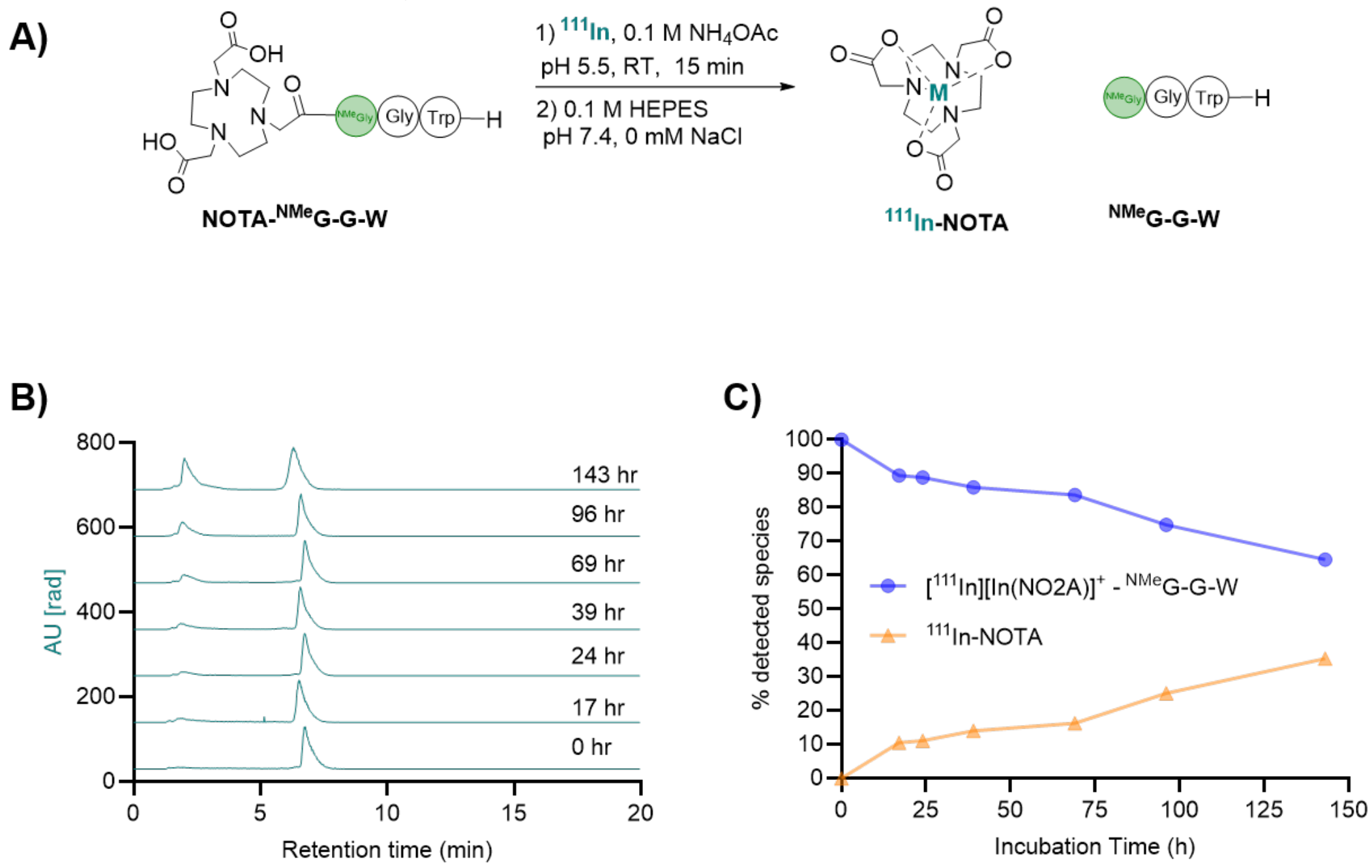

Figure S186. (A) Radiolabeling scheme and condition for the cleavage of **[^{111}In][In(2)]$^{+}$** complex. **(B)** Radio HPLC chromatograms (Method F) show the autolytic release of the ^{111}In-NOTA. **(C)** Quantification of the cleavage at room temperature, pH 7.4, and 0 mM NaCl.

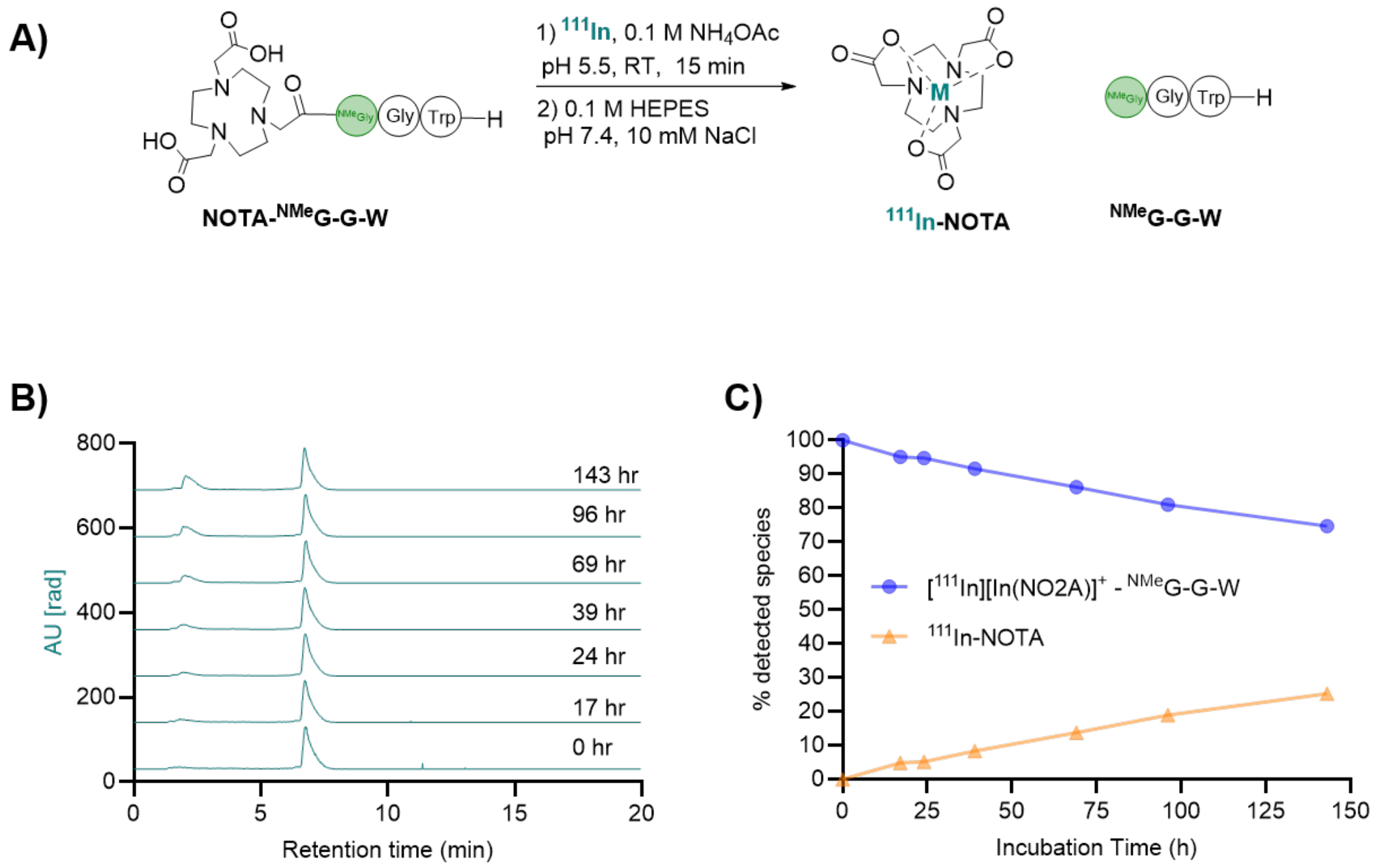

Figure S187. (A) Radiolabeling scheme and condition for the cleavage of **[^{111}In][In(2)]$^+$** complex. **(B)** Radio HPLC chromatograms (Method F) show the autolytic release of the ^{111}In-NOTA. **(C)** Quantification of the cleavage at room temperature, pH 7.4, and 10 mM NaCl.

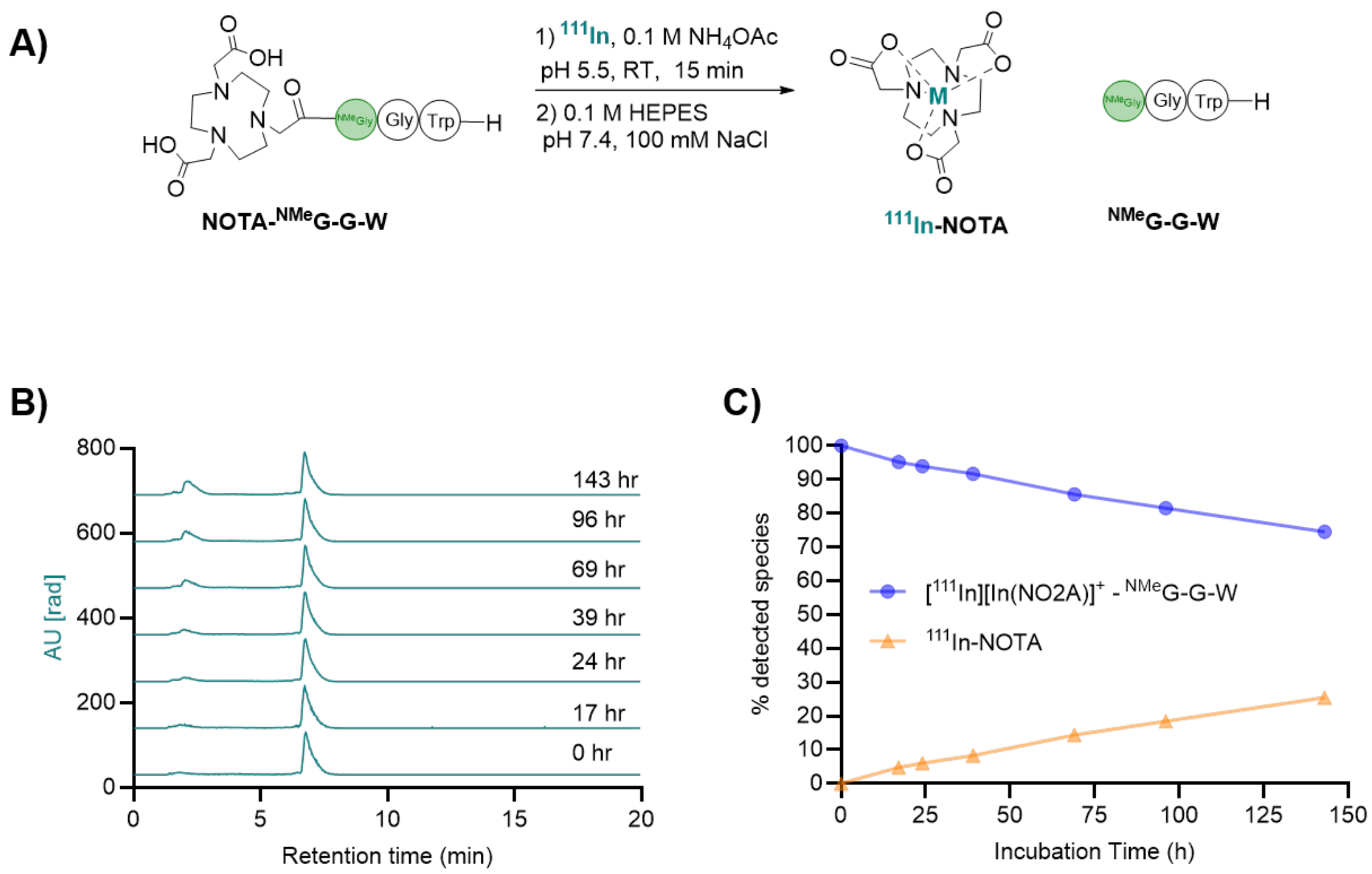

Figure S188. (A) Radiolabeling scheme and condition for the cleavage of **[^{111}In][In(2)]$^+$** complex. **(B)** Radio HPLC chromatograms (Method F) show the autolytic release of the ^{111}In-NOTA. **(C)** Quantification of the cleavage at room temperature, pH 7.4, and 100 mM NaCl.

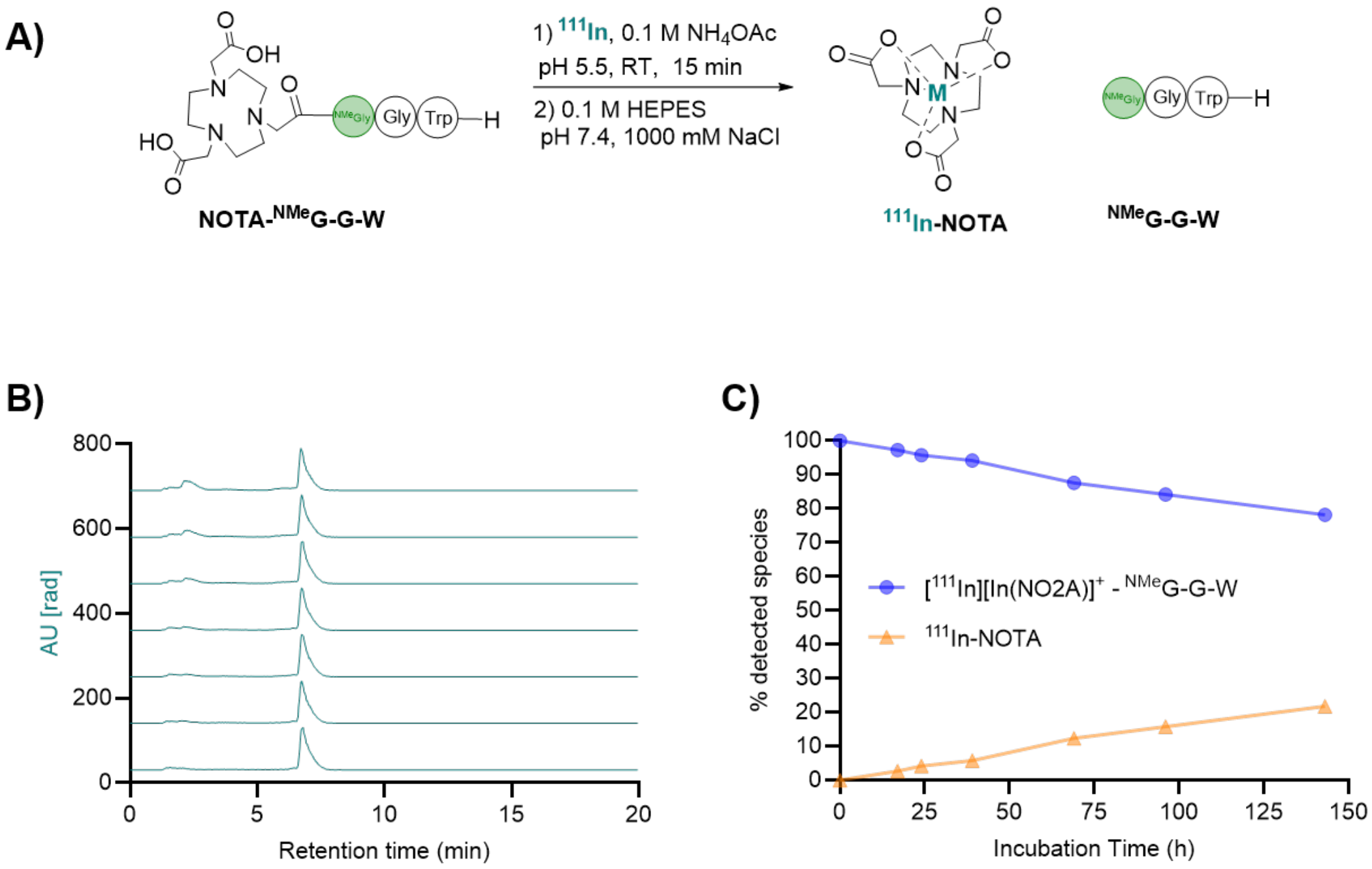

Figure S189. (A) Radiolabeling scheme and condition for the cleavage of **[^{111}In][In(2)]$^{+}$** complex. **(B)** Radio HPLC chromatograms (Method F) show the autolytic release of the ^{111}In-NOTA. **(C)** Quantification of the cleavage at room temperature, pH 7.4, and 1000 mM NaCl.

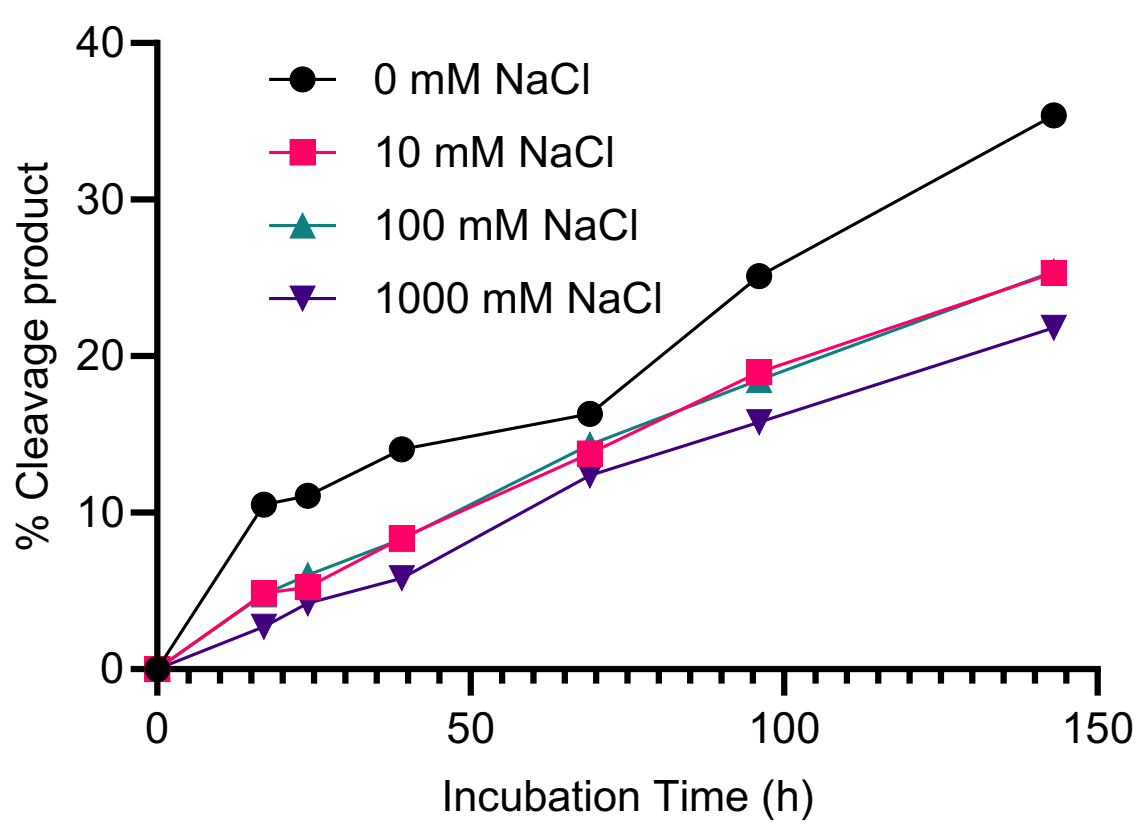

Figure S190. Summary for the quantification of [^{111}In][In(NO2A)]$^{+}$-^{NMe}G-G-W complex cleavage experiment with increasing concentrations of chloride ions at room temperature and pH 7.4.

7.7 Tabulated data

	[^{68}Ga][Ga-PD1]	[^{68}Ga][Ga-PD2]	[^{111}In][In-PD2]
Blood	0.74 ± 0.43	2.66 ± 0.69	1.07 ± 0.45
Heart	0.33 ± 0.08	1.19 ± 0.37	0.28 ± 0.06
Lung	0.82 ± 0.45	2.59 ± 0.69	0.60 ± 0.10
Liver	1.19 ± 0.65	3.06 ± 2.63	0.51 ± 0.10
Spleen	0.44 ± 0.20	2.73 ± 1.36	0.26 ± 0.22
Kidneys	4.94 ± 1.74	33.79 ± 19.96	14.44 ± 1.81
Stomach	0.14 ± 0.03	1.16 ± 0.92	0.27 ± 0.15
Small intestine	2.53 ± 1.41	1.95 ± 1.62	0.42 ± 0.14
Large intestine	25.60 ± 14.54	4.19 ± 5.75	0.99 ± 1.10
Muscle	0.42 ± 0.13	0.63 ± 0.15	0.78 ± 0.57
Bone	0.43 ± 0.17	0.90 ± 0.24	0.80 ± 0.37
Brain	0.07 ± 0.001	0.15 ± 0.04	0.06 ± 0.03
Tumor PSMA +	0.84 ± 0.12	9.61 ± 4.47	7.97 ± 0.41
Tumor PSMA -	0.94 ± 0.24	1.81 ± 0.41	0.65 ± 0.06
Tail	1.24 ± 0.47	21.44 ± 15.43	0.99 ± 0.05

Table S1. Decay-corrected biodistribution of [^{68}Ga][Ga-PD1], [^{68}Ga][Ga-PD2], and [^{111}In][In-PD2] at120 min post-injection (n=3) in NU/J mice models.

7.8 In vivo PET-CT imaging and Ex Vivo Biodistribution Results of PD1 and PD2.

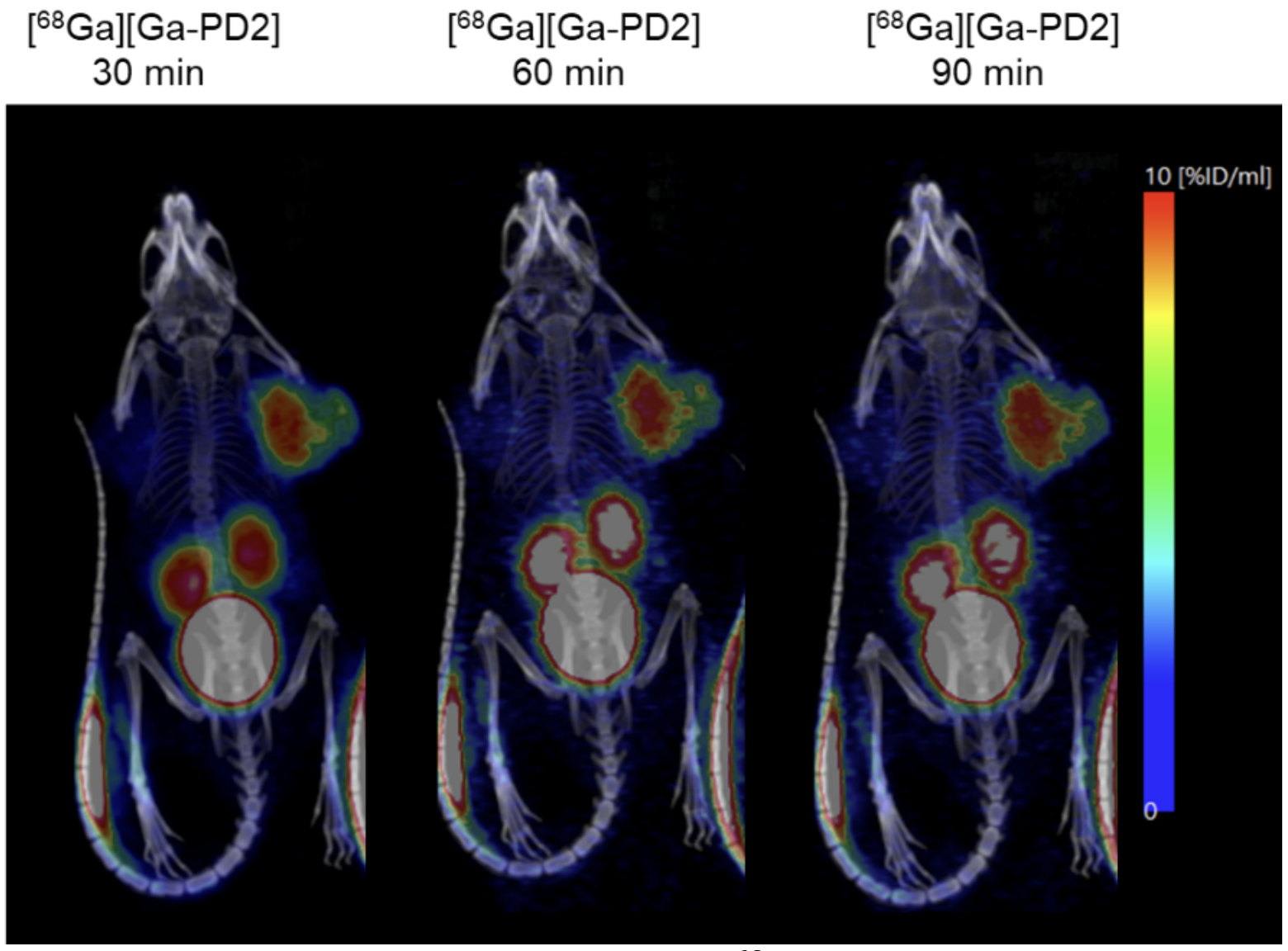

Figure S191. PET-CT images of [^{68}Ga][Ga-PD2] at 30 min, 60 min, and 90 min post-injection.

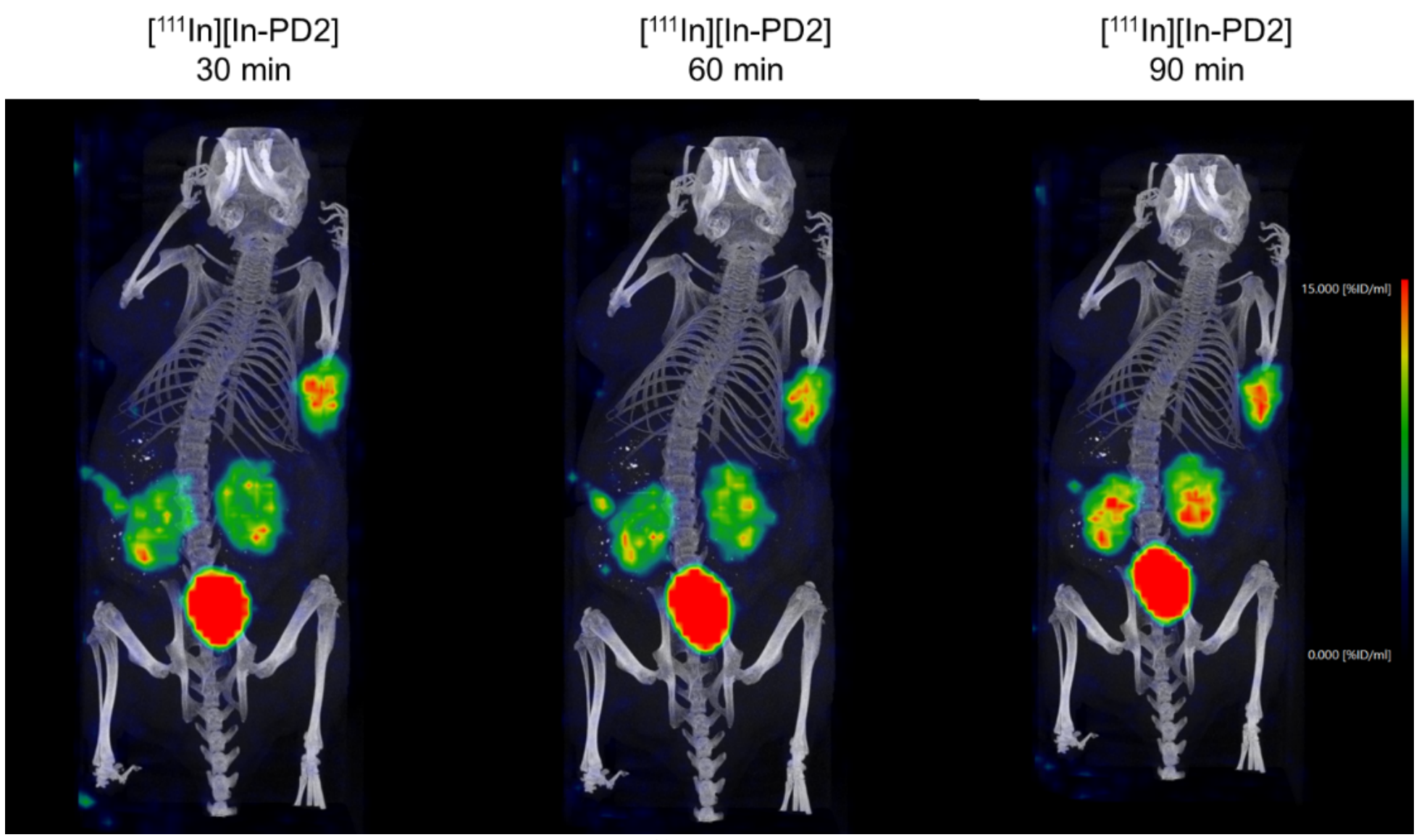

Figure S192. PET-CT images of [^{68}Ga][Ga-PD2] at 30 min, 60 min, and 90 min post-injection.

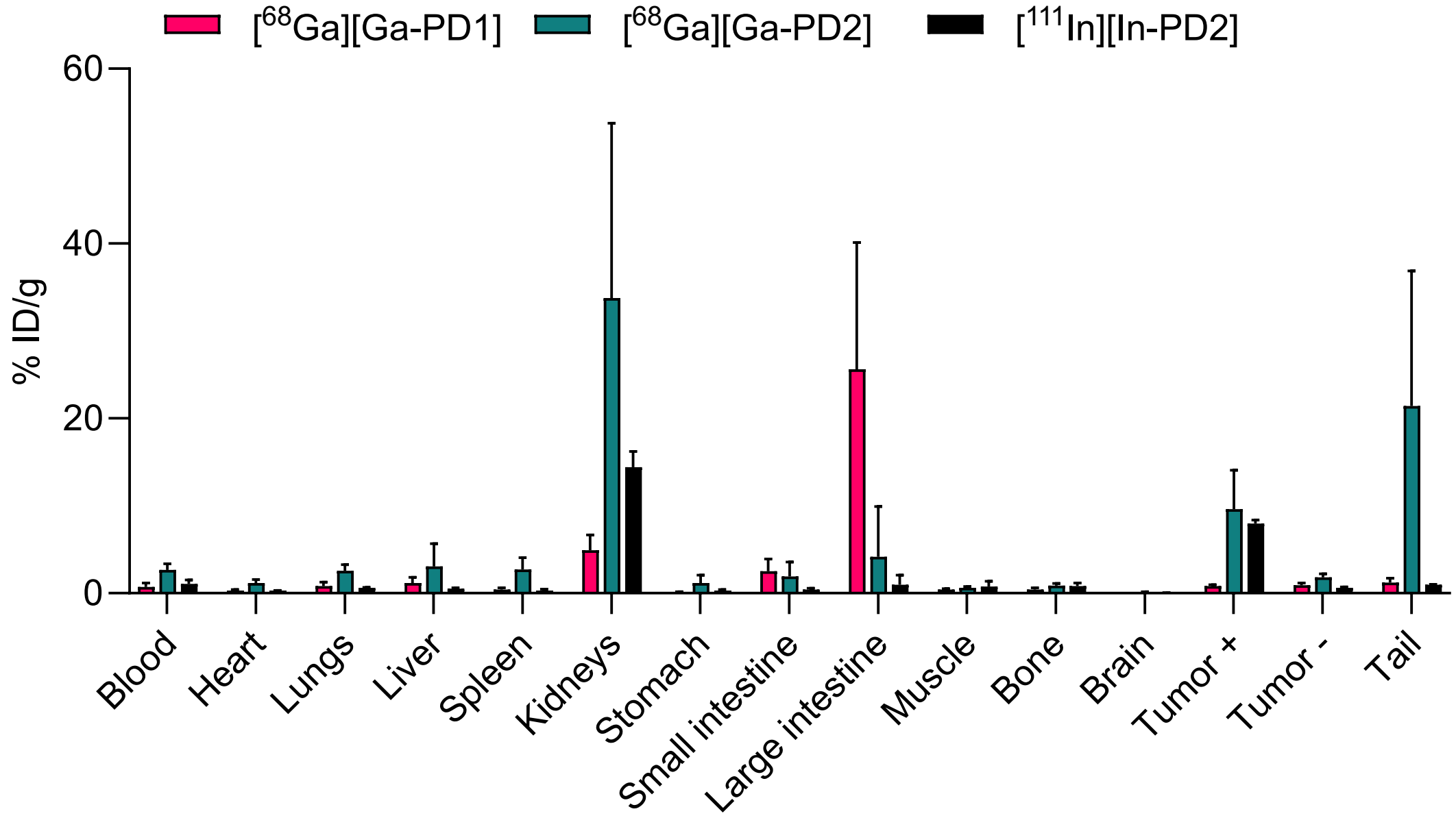

Figure S193. Ex vivo derived organ uptake values for the [^{68}Ga][Ga-PD1], [^{68}Ga][Ga-PD2], and [^{111}In][In-PD2] at 120 min post-injection.

7.9 Urine metabolite analysis

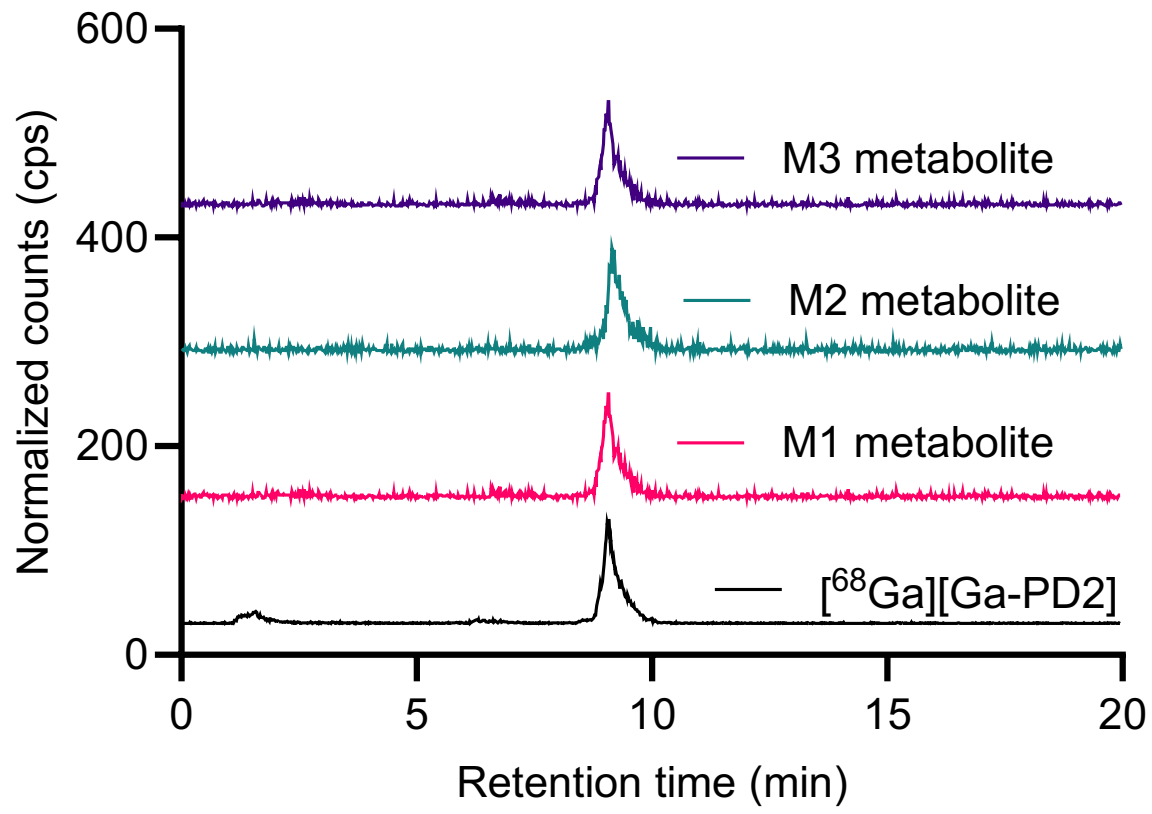

Figure S194. Comparative chromatographic analysis of [^{68}Ga][Ga-PD2] in the original radiolabeled product, the formulation of tracer in 1X DPBS and urine metabolite products found ex vivo. No cleavage product was observed in urine metabolites.

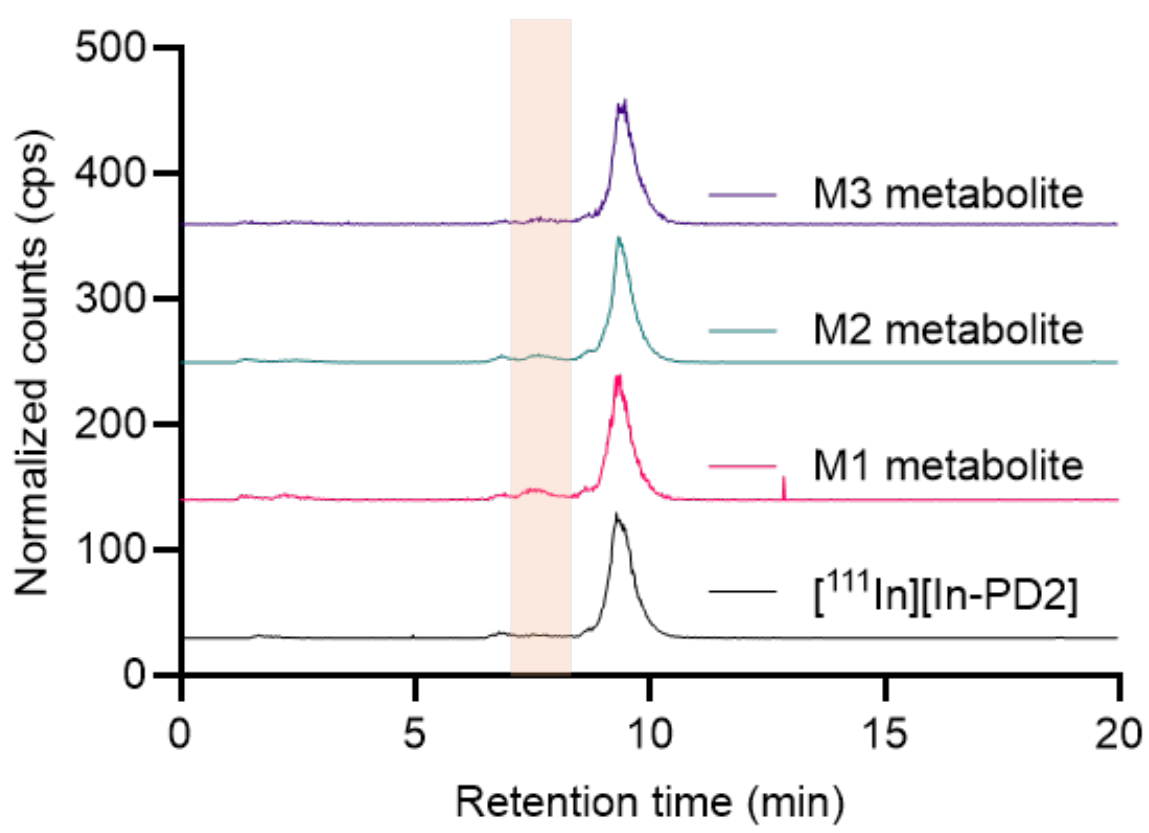

Figure S195. Comparative chromatographic analysis of [^{111}In][In-PD2] in the original radiolabeled product, the formulation of tracer in 1X DPBS and urine metabolites products found ex vivo. Peaks in the orange box were the in vivo cleavage product. 8.51 ± 1.63% cleavage product was observed in urine metabolites 120 min. p.i. time point.

7.10 Therapy study results of apo-PD2 and Ga-PD2 complex

7 – week old male NU/J nude mice were obtained from Jackson. On day 0, 1 × 10^6 PC3-Pip cells were injected subcutaneously into the right flanks of mice with 100 µL of a 1:2 mixture of Dulbecco's phosphate buffered saline (DPBS): Matrigel (Corning). Once tumor volumes reach between 100 – 200 mm^3 (took about 1 week), apo-PD2 (40 nmol/mouse) and Ga-PD2 (40 nmol/mouse) complex were administered by tail vein injection in 100 µL DPBS on days 8, 10, and 12 post tumor implantation. Mice were euthanized when tumor volume reached 2.5 cm^3. All animals were maintained according to protocols approved by IACUC.

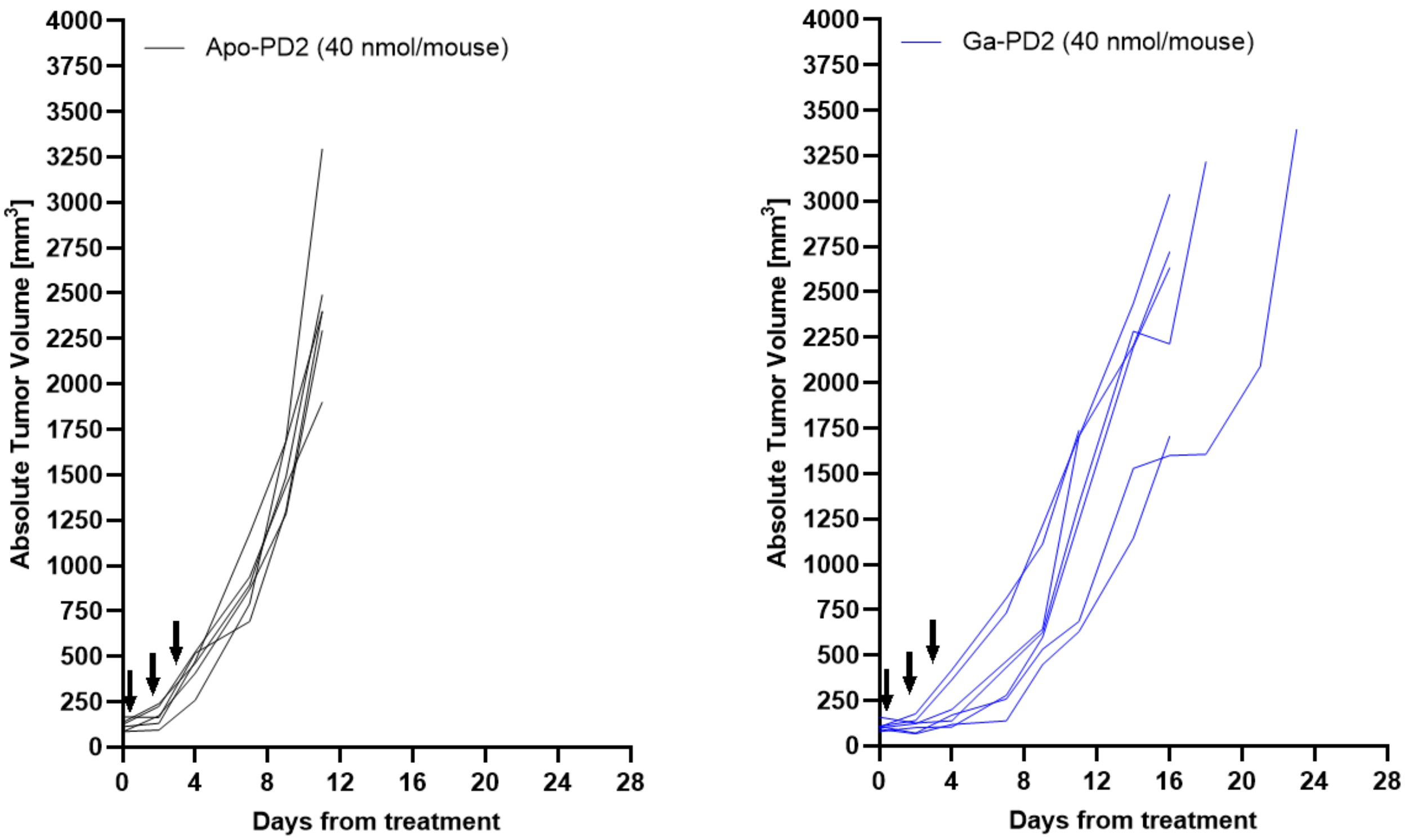

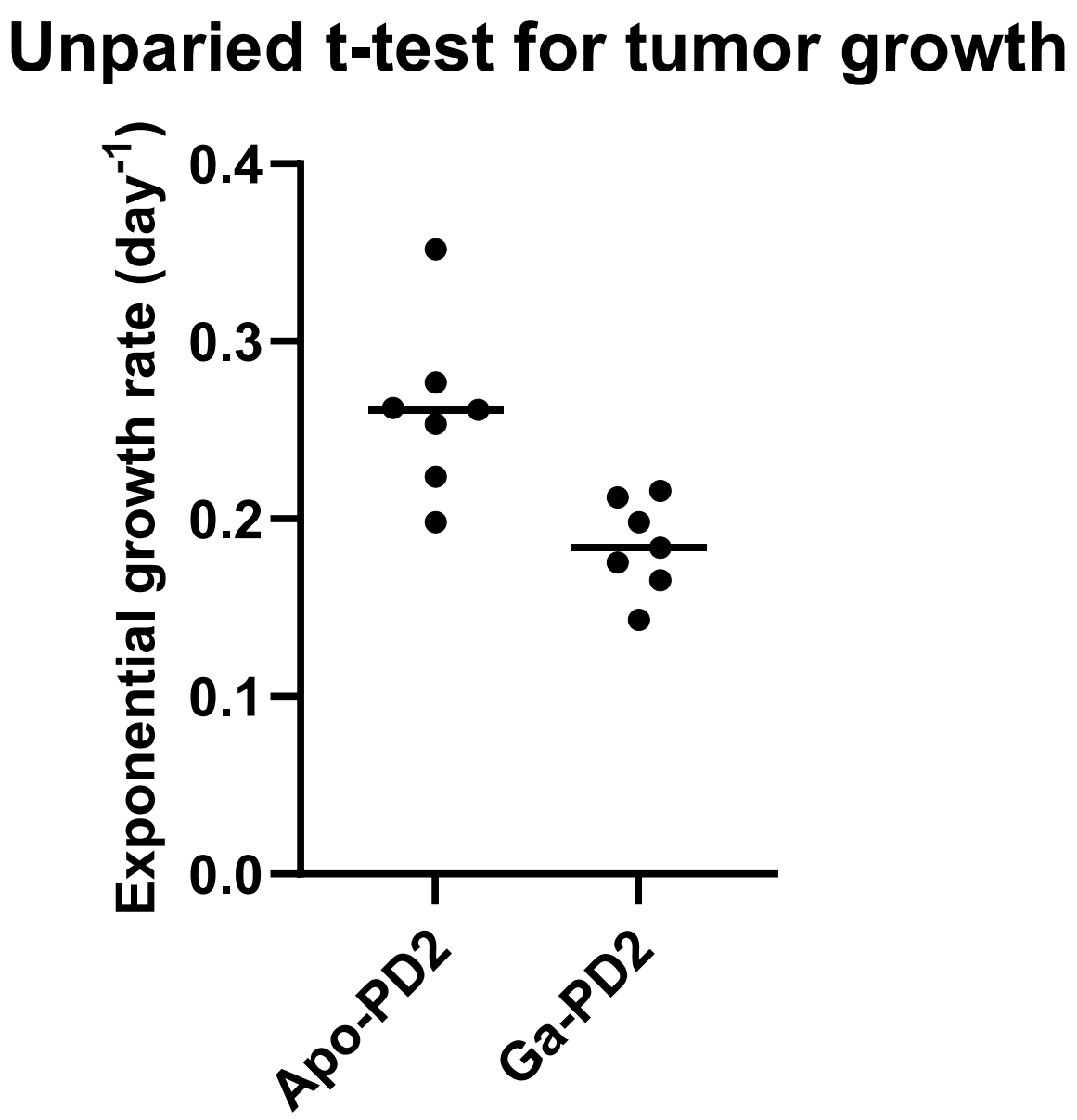

Figure S196. Tumor volume growth curves for individual mice. Left: apo-PD2. Right: Ga-PD2. Bottom: statistical analysis of tumor growth curves.

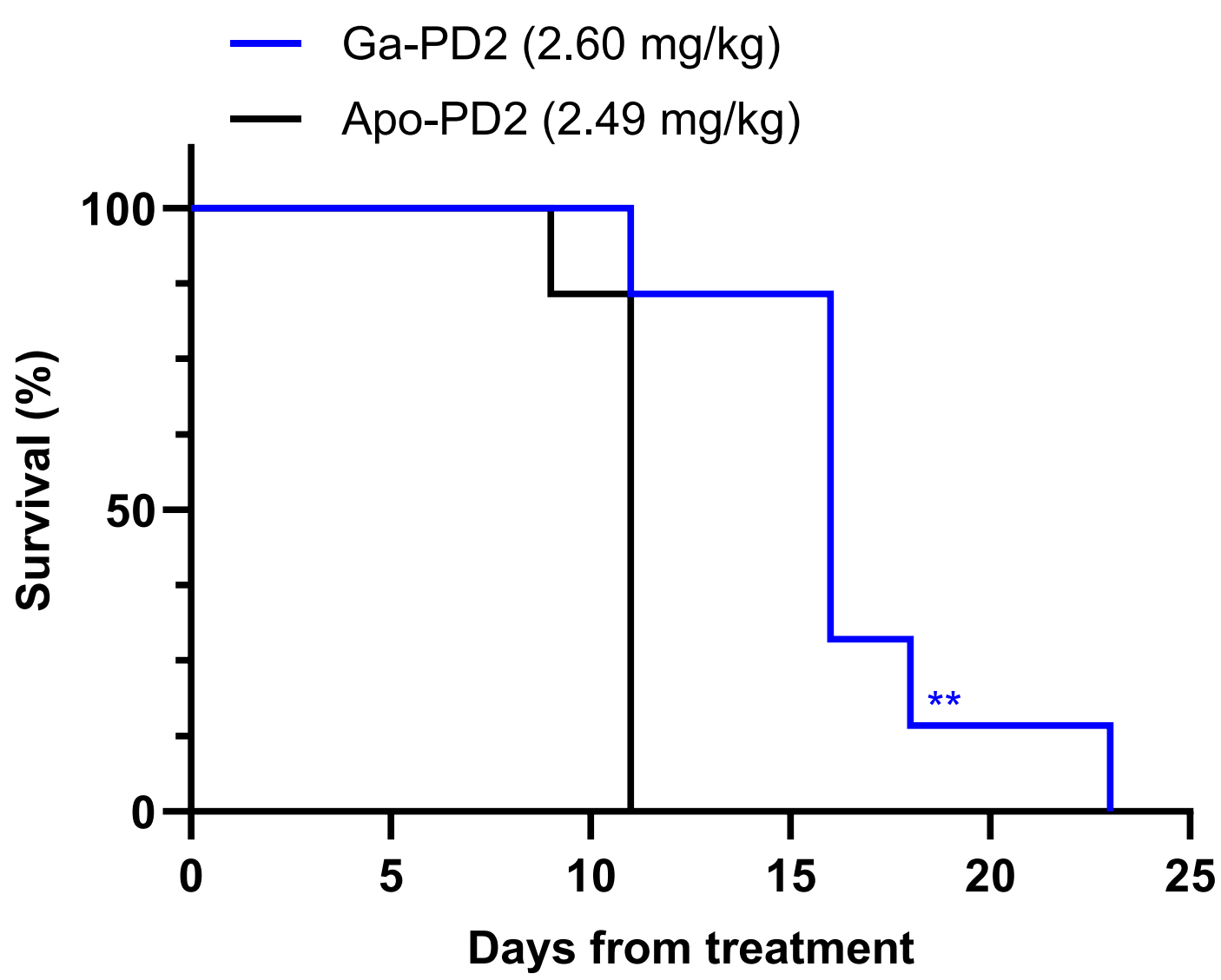

Figure S197. Median endpoint-free survival was 11 days for apo-PD2 and 16 days for Ga-PD2. Statistically significant differences are marked as ** ($p \leq 0.0021$).

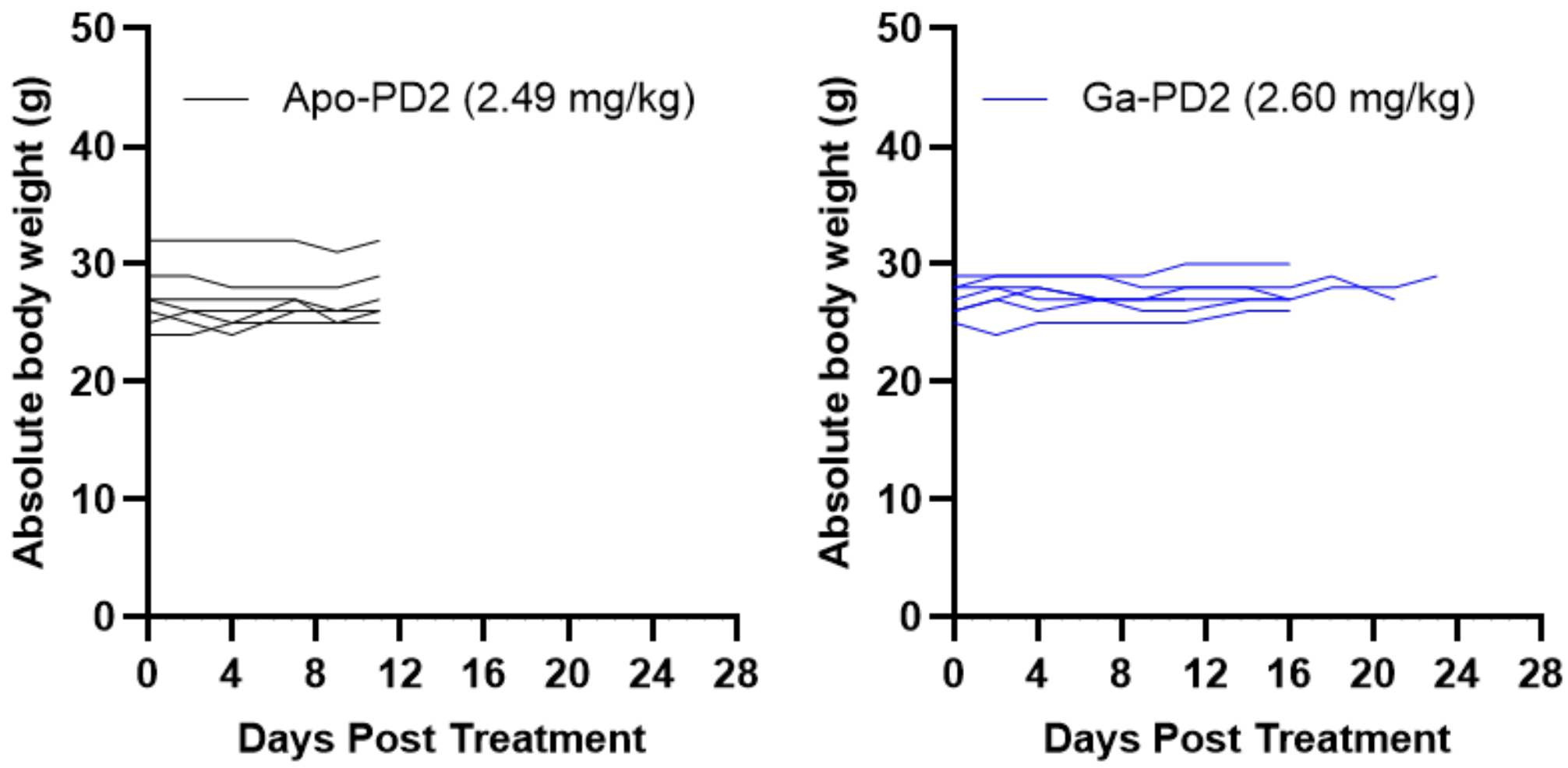

Figure S198. Body weight curve for individual mice throughout the therapy study. Left: apo-PD2. Right: Ga-PD2.

8 Crystallographic data

8.1 Crystallization methods

Crystals suitable for single-crystal X-ray diffraction were obtained under the following conditions. [In(NO2A)Cl]-NHBn·H_2O ([In(6)Cl]·H_2O) was crystallized by slow diffusion of dioxane into an aqueous solution of the complex prepared from $InCl_3$. $[Co(NO2A)]^+$-NHBn ([Co(6)]ClO_4) was crystallized by slow evaporation of a water/acetonitrile solution under ambient conditions in a fume hood, using $Co(ClO_4)_2$ for complexation, followed by oxidation at pH 2.0. [Co(NO2A)]-NHBn·4H_2O ([Co(6)]·4H_2O) was crystallized by slow diffusion of tetrahydrofuran into a methanol solution of the complex prepared from $CoCl_2$·6H_2O and oxidized at pH 7.0. $[Fe(NO2A)]^+$-NHBn ([Fe(6)]ClO_4) was crystallized by slow evaporation of a water/acetonitrile solution under ambient conditions in a fume hood, using $Fe(ClO_4)_3$ for complexation. $[Fe(NO2A)\text{-}NHBn]_2O$ ($[Fe(6)]_2O$) was crystallized by slow diffusion of tetrahydrofuran into a methanol/water solution of the complex prepared from $FeCl_3$.

8.2 X-ray Diffraction Analysis of [In(NO2A)Cl]-NHBn·H_2O ([In(6)Cl]·H_2O).

8.2.1 Data Collection

A colorless crystal with approximate dimensions 0.102 x 0.027 x 0.022 mm^3 was selected under oil under ambient conditions and attached to the tip of a MiTeGen MicroMount©. The crystal was mounted in a stream of cold nitrogen at 100(1) K and centered in the X-ray beam by using a video camera.

The crystal evaluation and data collection were performed on a Bruker D8 VENTURE Photon III four-circle diffractometer with Diamond II IµS Mo Kα (λ = 0.71073 Å) radiation and the detector to crystal distance of 5.0 cm.[10]

The initial cell constants were obtained from a 180° φ scan conducted at a 2θ = 30° angle with the exposure time of 1 second per frame. The reflections were successfully indexed by an automated indexing routine built in the APEX6 program. The final cell constants were calculated from a set of 9389 strong reflections from the actual data collection.

The data were collected by using the full sphere data collection routine to survey the reciprocal space to the extent of a full sphere to a resolution of 0.70 Å. A total of 106911 data were harvested by collecting 16 sets of frames with 0.5º scans in ω and φ with an exposure time of 1–22 sec per frame. These highly redundant datasets were corrected for Lorentz and polarization effects. The absorption correction was based on fitting a function to the empirical transmission surface as sampled by multiple equivalent measurements.[11]

8.2.2 Structure Solution and Refinement

The systematic absences in the diffraction data were uniquely consistent for the space group $P2_1/n$ that yielded chemically reasonable and computationally stable results of refinement.[12-17]

A successful solution by intrinsic phasing provided most non-hydrogen atoms from the *E*-map. The remaining non-hydrogen atoms were located in an alternating series of least-squares cycles and difference Fourier maps. All non-hydrogen atoms were refined with anisotropic atomic displacement coefficients. The hydrogen atoms bonded to N or O atoms were refined with distance restraints and relative isotropic atomic displacement coefficients. All hydrogen atoms bonded to C atoms were included in the structure factor calculation at idealized positions and were allowed to ride on the neighboring atoms with relative isotropic atomic displacement coefficients.

The asymmetric unit contains one $InC_{19}H_{26}ClN_4O_5$ molecule and one solvent water molecule for an overall chemical formula of $InC_{19}H_{26}ClN_4O_5 \cdot H_2O$.

The C14–C19 phenyl group on the $InC_{19}H_{26}ClN_4O_5$ molecule is disordered over two positions with a dominant component occupation of 81.7(13) %. The major and minor components of the disorder are refined with geometric restraints and anisotropic atomic displacement parameter restraints.

The final least-squares refinement of 344 parameters against 6477 data resulted in residuals R (based on F^2 for $I \geq 2\sigma$) and wR (based on F^2 for all data) of 0.0185 and 0.0497, respectively. The final difference Fourier map was featureless.

8.2.3 Summary

Crystal Data for $C_{19}H_{28}ClInN_4O_6$ (M =558.72 g/mol): monoclinic, space group $P2_1/n$ (no. 14), a = 6.9835(7) Å, b = 19.606(3) Å, c = 15.7382(15) Å, β = 99.463(8)°, V = 2125.5(4) Å^3, Z = 4, T = 100.00 K, μ(Mo Kα) = 1.283 mm^{-1}, $Dcalc$ = 1.746 g/cm^3, 105127 reflections measured (4.154° ≤ 2Θ ≤ 61.1°), 6477 unique (R_{int} = 0.0328, R_{sigma} = 0.0127) which were used in all calculations. The final R_1 was 0.0185 (I > 2σ(I)) and wR_2 was 0.0497 (all data).

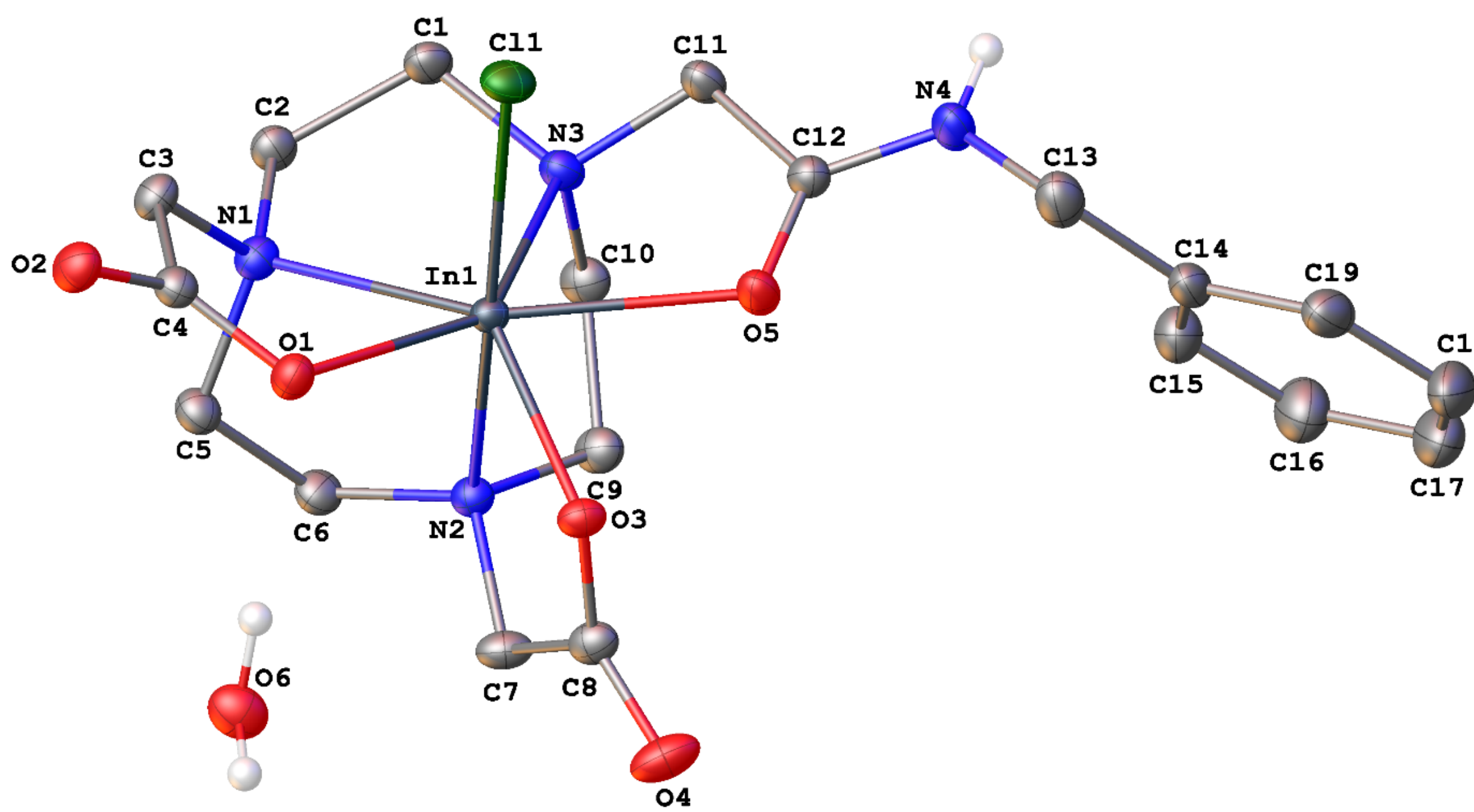

Figure S199. A molecular drawing of [In(6)Cl] shown with 50% probability ellipsoids. All H atoms bonded to C atoms and the minor component of the phenyl disorder are omitted.

Table S2. Crystallographic details for [In(NO2A)Cl]-NHBn·H2O.

Identification code	[In(NO2A)Cl]-NHBn·H_2O
Empirical formula	$InC_{19}H_{26}ClN_4O_5 \cdot H_2O$
Formula weight	558.72
Temperature/K	100.00
Crystal system	monoclinic

Space group	$P2_1/n$
a/Å	6.9835(7)
b/Å	19.606(3)
c/Å	15.7382(15)
α/°	90
β/°	99.463(8)
γ/°	90
Volume/Å^3	2125.5(4)
Z	4
ρ_{calc}g/cm^3	1.746
μ/mm^{-1}	1.283
F(000)	1136.0
Crystal size/mm^3	0.102 × 0.027 × 0.022
Radiation	Mo Kα (λ = 0.71073)
2Θ range for data collection/°	4.154 to 61.1
Index ranges	-9 ≤ h ≤ 9, -28 ≤ k ≤ 28, -22 ≤ l ≤ 22
Reflections collected	105127
Independent reflections	6477 [R_{int} = 0.0328, R_{sigma} = 0.0127]
Data/restraints/parameters	6477/274/344
Goodness-of-fit on F^2	1.047
Final R indexes [I>=2σ (I)]	R_1 = 0.0185, wR_2 = 0.0490
Final R indexes [all data]	R_1 = 0.0196, wR_2 = 0.0497
Largest diff. peak/hole / e Å^{-3}	0.79/-0.27

Table S3. Bond Lengths for [In(NO2A)Cl]-NHBn·H_2O.

Atom	Atom	Length/Å	Atom	Atom	Length/Å
In1	Cl1	2.4339(4)	N4	C13	1.4626(18)
In1	O1	2.2734(10)	C1	C2	1.5248(18)
In1	O3	2.1265(9)	C3	C4	1.5253(18)
In1	O5	2.3291(10)	C5	C6	1.5188(18)
In1	N1	2.3216(11)	C7	C8	1.5245(18)
In1	N2	2.3277(11)	C9	C10	1.5305(18)
In1	N3	2.3808(11)	C11	C12	1.5143(18)
O1	C4	1.2736(15)	C13	C14	1.519(3)
O2	C4	1.2473(16)	C13	C14A	1.515(9)
O3	C8	1.2898(15)	C14	C15	1.387(3)
O4	C8	1.2243(16)	C14	C19	1.405(3)
O5	C12	1.2470(16)	C15	C16	1.399(3)

Table S3. Bond Lengths for [In(NO2A)Cl]-NHBn·H_2O.

Atom	Atom	Length/Å
N1	C2	1.4861(16)
N1	C3	1.4719(16)
N1	C5	1.4934(16)
N2	C6	1.4887(17)
N2	C7	1.4801(16)
N2	C9	1.4852(17)
N3	C1	1.4979(17)
N3	C10	1.4957(17)
N3	C11	1.4754(16)
N4	C12	1.3274(17)
C16	C17	1.391(3)
C17	C18	1.393(4)
C18	C19	1.396(3)
C14A	C15A	1.384(9)
C14A	C19A	1.391(9)
C15A	C16A	1.401(10)
C16A	C17A	1.387(9)
C17A	C18A	1.376(9)
C18A	C19A	1.391(9)

Table S4. Bond Angles for [In(NO2A)Cl]-NHBn·H_2O.

Atom	Atom	Atom	Angle/°
O1	In1	Cl1	84.05(3)
O1	In1	O5	142.82(4)
O1	In1	N1	71.47(4)
O1	In1	N2	104.44(4)
O1	In1	N3	145.23(4)
O3	In1	Cl1	114.95(3)
O3	In1	O1	79.82(4)
O3	In1	O5	73.67(3)
O3	In1	N1	133.62(4)
O3	In1	N2	77.21(4)
O3	In1	N3	132.71(4)
O5	In1	Cl1	84.02(3)
O5	In1	N3	69.74(4)
N1	In1	Cl1	97.71(3)
N1	In1	O5	145.16(4)
N1	In1	N2	75.75(4)
N1	In1	N3	75.45(4)
N2	In1	Cl1	166.59(3)
N2	In1	O5	94.69(4)
N2	In1	N3	77.01(4)
N3	In1	Cl1	90.08(3)
C4	O1	In1	113.74(8)
C8	O3	In1	119.42(8)
C11	N3	C1	110.34(10)
C11	N3	C10	112.60(11)
C12	N4	C13	121.64(12)
N3	C1	C2	112.17(10)
N1	C2	C1	112.05(10)
N1	C3	C4	111.62(10)
O1	C4	C3	117.52(11)
O2	C4	O1	124.79(12)
O2	C4	C3	117.69(11)
N1	C5	C6	110.80(10)
N2	C6	C5	111.59(10)
N2	C7	C8	114.09(10)
O3	C8	C7	117.39(11)
O4	C8	O3	124.12(12)
O4	C8	C7	118.39(12)
N2	C9	C10	111.96(10)
N3	C10	C9	114.09(11)
N3	C11	C12	110.17(10)
O5	C12	N4	122.42(12)
O5	C12	C11	120.33(12)
N4	C12	C11	117.18(11)
N4	C13	C14	111.90(17)
N4	C13	C14A	121.9(8)

Table S4. Bond Angles for [In(NO2A)Cl]-NHBn·H_2O.

Atom	Atom	Atom	Angle/°	Atom	Atom	Atom	Angle/°
C12	O5	In1	116.19(8)	C15	C14	C13	122.6(2)
C2	N1	In1	113.32(8)	C15	C14	C19	118.8(2)
C2	N1	C5	110.95(10)	C19	C14	C13	118.7(2)
C3	N1	In1	104.99(7)	C14	C15	C16	120.7(3)
C3	N1	C2	112.44(10)	C17	C16	C15	120.3(3)
C3	N1	C5	110.26(10)	C16	C17	C18	119.6(2)
C5	N1	In1	104.45(7)	C17	C18	C19	119.9(2)
C6	N2	In1	111.79(7)	C18	C19	C14	120.7(2)
C7	N2	In1	107.09(7)	C15A	C14A	C13	115.9(12)
C7	N2	C6	111.79(10)	C15A	C14A	C19A	126.1(12)
C7	N2	C9	110.83(10)	C19A	C14A	C13	117.9(12)
C9	N2	In1	103.07(7)	C14A	C15A	C16A	118.4(14)
C9	N2	C6	111.86(10)	C17A	C16A	C15A	116.5(15)
C1	N3	In1	104.00(8)	C18A	C17A	C16A	123.2(15)
C10	N3	In1	108.30(8)	C17A	C18A	C19A	122.0(15)
C10	N3	C1	112.84(10)	C18A	C19A	C14A	113.6(13)
C11	N3	In1	108.27(8)				

8.3 X-ray Diffraction Analysis of $[Co(NO2A)]^{+}$-NHBn ($[Co(6)]ClO_4$).

8.3.1 Data Collection

A purple crystal with approximate dimensions 0.1 x 0.03 x 0.02 mm^3 was selected under oil under ambient conditions and attached to the tip of a MiTeGen MicroMount©. The crystal was mounted in a stream of cold nitrogen at 110(1) K and centered in the X-ray beam by using a video camera.

The crystal evaluation and data collection were performed on a Bruker D8 VENTURE PhotonIII four-circle diffractometer with Cu Kα (λ = 1.54178 Å) radiation and the detector to crystal distance of 4.0 and 5.0 cm.[10]

The initial cell constants were obtained from a 180° φ scan conducted at a 2θ = 50° angle with the exposure time of 1 second per frame. The reflections were successfully indexed by an automated indexing routine built in the APEX6 program. The final cell constants were calculated from a set of 9883 strong reflections from the actual data collection.

The data were collected by using the full sphere data collection routine to survey the reciprocal space to the extent of a full sphere to a resolution of 0.79 Å. A total of 25954 data were harvested by collecting 14 sets of frames with 0.9 and 1.0º scans in ω and φ with an exposure time 1–5 sec per frame. These highly redundant datasets were corrected for Lorentz and polarization effects. The absorption correction was based on fitting a function to the empirical transmission surface as sampled by multiple equivalent measurements.[11]

8.3.2 Structure Solution and Refinement

The systematic absences in the diffraction data were uniquely consistent for the space group $P2_1/n$ that yielded chemically reasonable and computationally stable results of refinement.[12-17]

A successful solution by intrinsic phasing provided most non-hydrogen atoms from the *E*-map. The remaining non-hydrogen atoms were located in an alternating series of least-squares cycles and difference Fourier maps. All non-hydrogen atoms were refined with anisotropic displacement coefficients unless specified otherwise. All hydrogen atoms bonded to carbon atoms were included in the structure factor calculation at idealized positions and were allowed to ride on the neighboring atoms with relative isotropic displacement coefficients.

The compound composition is $(CoC_{19}H_{26}N_4O_5)ClO_4$. The N–H bond was refined with a distance restraint. The perchlorate anion (the four oxygen atoms) is disordered over two positions with the minor component contribution of 0.118(4). The oxygen atoms of the minor disorder component were refined isotropically with atomic displacement parameter constraints. The minor disorder component was refined with bond distance restraints.

The final least-squares refinement of 324 parameters against 4568 data resulted in residuals *R* (based on F^2 for $I \geq 2\sigma$) and *wR* (based on F^2 for all data) of 0.0344 and 0.0878, respectively. The final difference Fourier map was featureless.

8.3.3 Summary

Crystal Data for $C_{19}H_{26}ClCoN_4O_9$ (M =548.82 g/mol): monoclinic, space group $P2_1/n$ (no. 14), a = 8.7975(12) Å, b = 20.704(3) Å, c = 11.8290(15) Å, β = 95.148(7)°, V = 2145.8(5) $Å^3$, Z = 4, T = 110.00 K, μ(CuKα) = 7.977 mm^{-1}, $Dcalc$ = 1.699 g/cm^3, 25954 reflections measured (8.636° ≤ 2Θ ≤ 157.774°), 4568 unique (R_{int} = 0.0457, R_{sigma} = 0.0299) which were used in all calculations. The final R_1 was 0.0344 (I > 2σ(I)) and wR_2 was 0.0878 (all data).

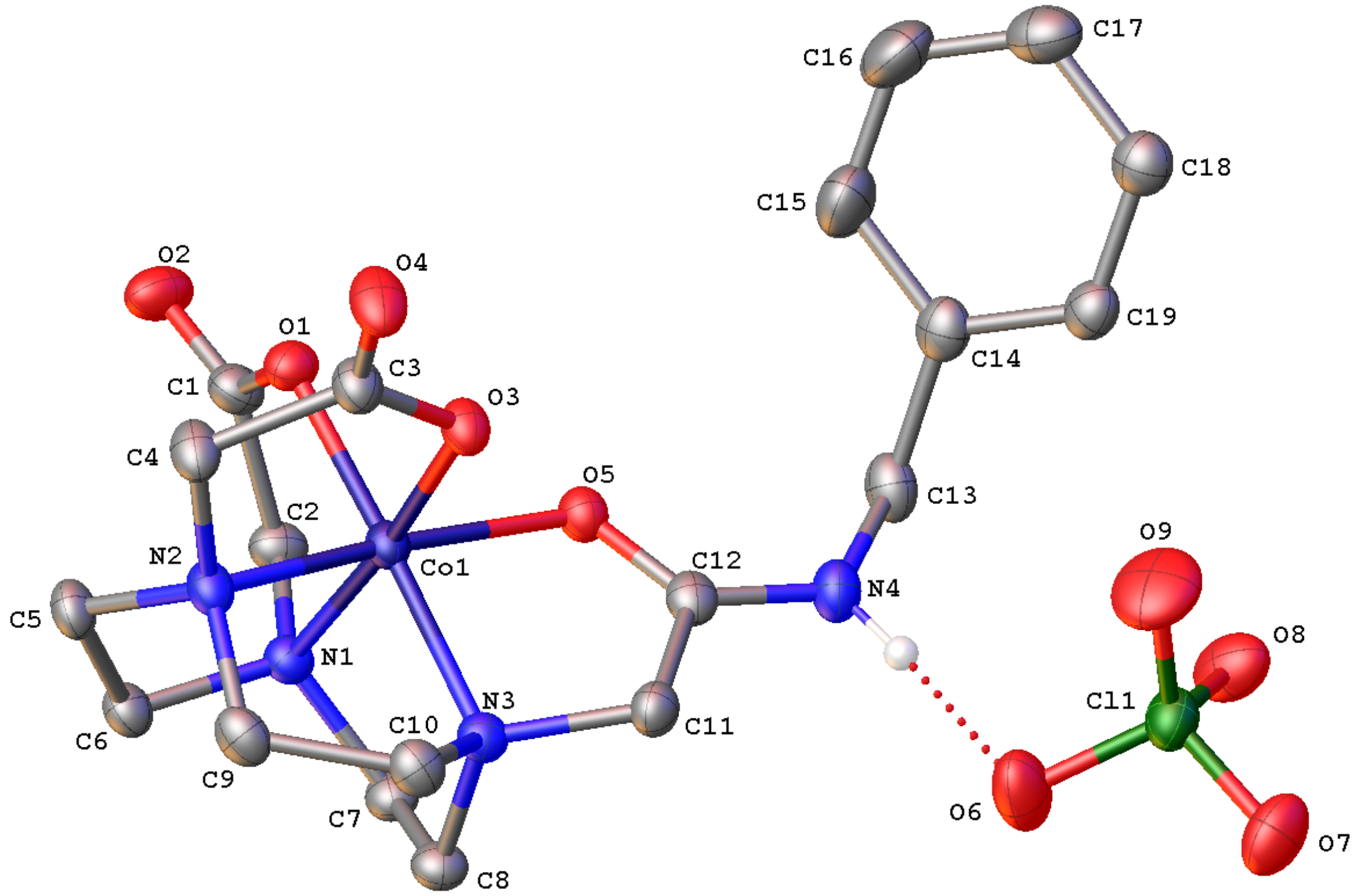

Figure S200. A molecular drawing of [Co(6)]ClO_4 shown with 50% probability ellipsoids. All H atoms bonded to C atoms and the minor component of the phenyl disorder are omitted.

Table S5. Crystallographic details for [Co(NO2A)]$^+$-NHBn.

Identification code	[Co(NO2A)]$^+$-NHBn
Empirical formula	$(CoC_{19}H_{26}N_4O_5)ClO_4$
Formula weight	548.82
Temperature/K	110.00
Crystal system	monoclinic
Space group	*P2₁/n*
a/Å	8.7975(12)
b/Å	20.704(3)
c/Å	11.8290(15)
α/°	90
β/°	95.148(7)
γ/°	90
Volume/Å^3	2145.8(5)
Z	4
ρ_{calc}g/cm^3	1.699
μ/mm^{-1}	7.977
F(000)	1136.0
Crystal size/mm^3	0.1 × 0.03 × 0.02

Radiation	CuKα (λ = 1.54178)
2Θ range for data collection/°	8.636 to 157.774
Index ranges	-11 ≤ h ≤ 10, -23 ≤ k ≤ 26, -15 ≤ l ≤ 14
Reflections collected	25954
Independent reflections	4568 [R_{int} = 0.0457, R_{sigma} = 0.0299]
Data/restraints/parameters	4568/11/324
Goodness-of-fit on F^2	1.046
Final R indexes [I>=2σ (I)]	R_1 = 0.0344, wR_2 = 0.0857
Final R indexes [all data]	R_1 = 0.0377, wR_2 = 0.0878
Largest diff. peak/hole / e Å^{-3}	0.50/-0.36

Table S6. Bond Lengths for [Co(NO2A)]$^+$-NHBn.

Atom	Atom	Length/Å	Atom	Atom	Length/Å
Co1	O1	1.9044(14)	C1	C2	1.519(3)
Co1	O3	1.8994(14)	C3	C4	1.526(3)
Co1	O5	1.9420(14)	C5	C6	1.517(3)
Co1	N1	1.9262(16)	C7	C8	1.520(3)
Co1	N2	1.9192(17)	C9	C10	1.518(3)
Co1	N3	1.9365(17)	C11	C12	1.506(3)
O1	C1	1.303(3)	C13	C14	1.518(3)
O2	C1	1.222(3)	C14	C15	1.382(3)
O3	C3	1.302(2)	C14	C19	1.393(3)
O4	C3	1.219(3)	C15	C16	1.394(4)
O5	C12	1.269(2)	C16	C17	1.377(4)
N1	C2	1.486(2)	C17	C18	1.390(3)
N1	C6	1.514(2)	C18	C19	1.390(3)
N1	C7	1.491(2)	Cl1	O6	1.440(2)
N2	C4	1.486(2)	Cl1	O7	1.4299(19)
N2	C5	1.499(2)	Cl1	O8	1.445(2)
N2	C9	1.514(3)	Cl1	O9	1.418(2)
N3	C8	1.519(2)	Cl1	O6A	1.466(9)
N3	C10	1.494(3)	Cl1	O7A	1.445(9)
N3	C11	1.491(2)	Cl1	O8A	1.401(9)
N4	C12	1.308(3)	Cl1	O9A	1.433(9)
N4	C13	1.464(3)			

Table S7. Bond Angles for [Co(NO2A)]$^+$-NHBn.

Atom	Atom	Atom	Angle/°	Atom	Atom	Atom	Angle/°
O1	Co1	O5	93.87(6)	O2	C1	O1	123.96(19)
O1	Co1	N1	85.52(6)	O2	C1	C2	122.16(19)
O1	Co1	N2	92.55(7)	N1	C2	C1	107.44(16)
O1	Co1	N3	174.30(6)	O3	C3	C4	114.46(17)

Table S7. Bond Angles for [Co(NO2A)]$^{+}$-NHBn.

Atom	Atom	Atom	Angle/°	Atom	Atom	Atom	Angle/°
O3	Co1	O1	91.97(6)	O4	C3	O3	125.02(18)
O3	Co1	O5	90.58(6)	O4	C3	C4	120.52(18)
O3	Co1	N1	174.25(7)	N2	C4	C3	109.37(16)
O3	Co1	N2	86.04(6)	N2	C5	C6	107.22(16)
O3	Co1	N3	93.59(7)	N1	C6	C5	110.46(15)
N1	Co1	O5	94.74(6)	N1	C7	C8	107.53(15)
N1	Co1	N3	89.05(7)	N3	C8	C7	110.26(15)
N2	Co1	O5	172.85(7)	N2	C9	C10	110.89(16)
N2	Co1	N1	88.91(7)	N3	C10	C9	106.82(16)
N2	Co1	N3	89.06(7)	N3	C11	C12	107.14(16)
N3	Co1	O5	84.85(7)	O5	C12	N4	123.18(19)
C1	O1	Co1	113.86(12)	O5	C12	C11	118.85(17)
C3	O3	Co1	113.68(12)	N4	C12	C11	117.96(18)
C12	O5	Co1	111.67(13)	N4	C13	C14	112.77(17)
C2	N1	Co1	104.86(11)	C15	C14	C13	120.3(2)
C2	N1	C6	110.98(15)	C15	C14	C19	119.2(2)
C2	N1	C7	113.65(15)	C19	C14	C13	120.4(2)
C6	N1	Co1	108.76(11)	C14	C15	C16	120.3(2)
C7	N1	Co1	106.15(12)	C17	C16	C15	120.5(2)
C7	N1	C6	111.97(15)	C16	C17	C18	119.7(2)
C4	N2	Co1	105.43(12)	C17	C18	C19	119.9(2)
C4	N2	C5	113.43(16)	C18	C19	C14	120.5(2)
C4	N2	C9	111.64(16)	O6	Cl1	O8	107.81(14)
C5	N2	Co1	106.68(12)	O7	Cl1	O6	108.50(14)
C5	N2	C9	110.91(15)	O7	Cl1	O8	109.18(13)
C9	N2	Co1	108.36(12)	O9	Cl1	O6	110.16(15)
C8	N3	Co1	108.15(12)	O9	Cl1	O7	111.68(15)
C10	N3	Co1	106.24(12)	O9	Cl1	O8	109.43(15)
C10	N3	C8	111.50(15)	O7A	Cl1	O6A	104.7(10)
C11	N3	Co1	106.38(12)	O8A	Cl1	O6A	108.1(10)
C11	N3	C8	111.86(15)	O8A	Cl1	O7A	113.5(10)
C11	N3	C10	112.33(15)	O8A	Cl1	O9A	118.3(10)
C12	N4	C13	124.32(18)	O9A	Cl1	O6A	103.3(9)
O1	C1	C2	113.87(17)	O9A	Cl1	O7A	107.6(10)

8.4 X-ray Diffraction Analysis of [Co(NO2A)]-NHBn·4H_2O ([Co(6)]·4H_2O).

8.4.1 Data Collection

A purple crystal with approximate dimensions 0.057 x 0.017 x 0.012 mm^3 was selected under oil under ambient conditions and attached to the tip of a MiTeGen MicroMount©. The crystal was mounted in a stream of cold nitrogen at 100(1) K and centered in the X-ray beam by using a video camera.

The crystal evaluation and data collection were performed on a Bruker D8 VENTURE Photon III four-circle diffractometer with Diamond II IµS Mo Kα (λ = 0.71073 Å) radiation and the detector to crystal distance of 5.0 cm.[10]

The initial cell constants were obtained from a 180° φ scan conducted at a 2θ = 30° angle with the exposure time of 3 seconds per frame. The reflections were successfully indexed by an automated indexing routine built in the APEX6 program. The final cell constants were calculated from a set of 9319 strong reflections from the actual data collection.

The data were collected by using the full sphere data collection routine to survey the reciprocal space to the extent of a full sphere to a resolution of 0.77 Å. A total of 54941 data were harvested by collecting 8 sets of frames with 0.5–1.0° scans in ω and φ with an exposure time 5, 10, and 143 sec per frame. These highly redundant datasets were corrected for Lorentz and polarization effects. The absorption correction was based on fitting a function to the empirical transmission surface as sampled by multiple equivalent measurements.[11]

8.4.2 Structure Solution and Refinement

The systematic absences in the diffraction data were uniquely consistent for the space group $P2_12_12_1$ that yielded chemically reasonable and computationally stable results of refinement.[12-17]

A successful solution by intrinsic phasing provided most non-hydrogen atoms from the *E*-map. The remaining non-hydrogen atoms were located with an alternating series of least-squares cycles and difference Fourier maps. The atomic form factors were determined by DFT calculations, using the r2SCAN hybrid functional and the def2-TZVPD basis set, in the NoSpherA2 extension of the olex2.refine program.[18-20] All non-hydrogen atoms were refined with anisotropic atomic displacement coefficients. Hydrogen atoms bonded to oxygen atoms were refined with distance restraints and relative isotropic atomic displacement coefficients. All other hydrogen atoms were included in the structure factor calculation at idealized positions and were allowed to ride on the neighboring atoms with relative isotropic atomic displacement coefficients.

The asymmetric unit contains one molecule of $CoC_{19}H_{33}N_4O_5$ and 4 water molecules for an overall chemical formula of $CoC_{19}H_{33}N_4O_5 \cdot 4H_2O$.

The absolute structure was unequivocally established by resonant scattering effects.

The final least-squares refinement of 322 parameters against 5026 data resulted in residuals R (based on F^2 for $I \geq 2\sigma$) and wR (based on F^2 for all data) of 0.0242 and 0.0548, respectively. The final difference Fourier map was featureless.

8.4.3 Summary

Crystal Data for $C_{19}H_{33}CoN_4O_9$ (M =520.429 g/mol): orthorhombic, space group $P2_12_12_1$ (no. 19), a = 8.4228(17) Å, b = 12.210(3) Å, c = 21.223(4) Å, V = 2182.6(9) $Å^3$, Z = 4, T = 100 K, μ(Mo Kα) = 0.847 mm^{-1}, $Dcalc$ = 1.584 g/cm^3, 54755 reflections measured (3.84° ≤ 2Θ ≤ 55.08°), 5026 unique (R_{int} = 0.0594, R_{sigma} = 0.0308) which were used in all calculations. The final R_1 was 0.0242 (I>=2u(I)) and wR_2 was 0.0548 (all data).

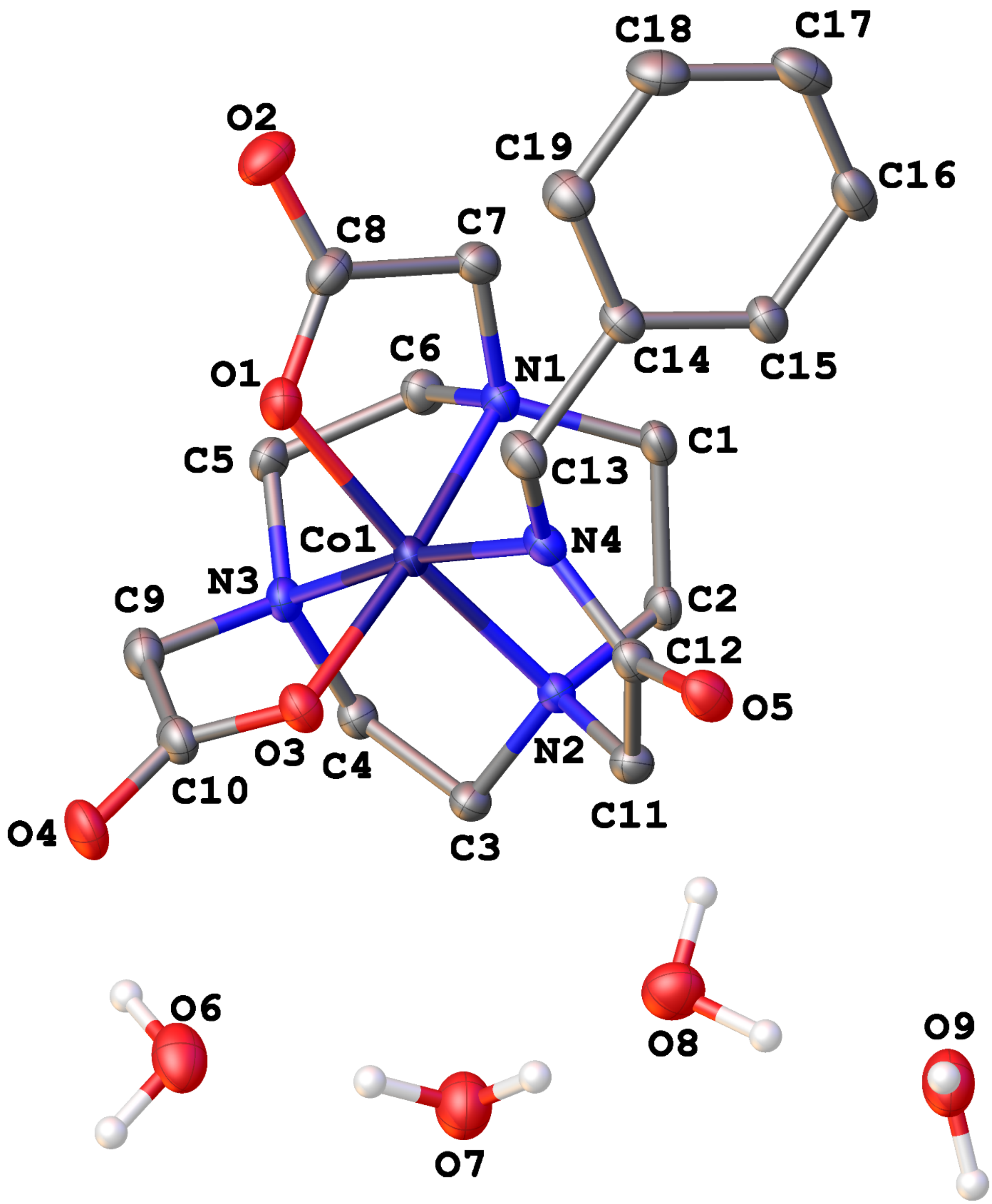

Figure S201. A molecular drawing of [Co(6)]·$4H_2O$ shown with 50% probability ellipsoids. All H atoms bonded to C atoms are omitted.

Table S8. Crystallographic details for [Co(NO2A)]-NHBn·$4H_2O$.

Identification code	[Co(NO2A)]-NHBn·$4H_2O$

Empirical formula	$CoC_{19}H_{33}N_4O_5{\cdot}4H_2O$
Formula weight	520.429
Temperature/K	100
Crystal system	orthorhombic
Space group	$P2_12_12_1$
a/Å	8.4228(17)
b/Å	12.210(3)
c/Å	21.223(4)
α/°	90
β/°	90
γ/°	90
Volume/Å^3	2182.6(9)
Z	4
ρ_{calc}g/cm^3	1.584
μ/mm^{-1}	0.847
F(000)	1098.3
Crystal size/mm^3	0.057 × 0.017 × 0.012
Radiation	Mo Kα (λ = 0.71073)
2Θ range for data collection/°	3.84 to 55.08
Index ranges	$-9 \le h \le 10$, $-15 \le k \le 15$, $-27 \le l \le 27$
Reflections collected	54755
Independent reflections	5026 [R_{int} = 0.0594, R_{sigma} = 0.0308]
Data/restraints/parameters	5026/12/322
Goodness-of-fit on F^2	1.040
Final R indexes [I>=2σ (I)]	R_1 = 0.0242, wR_2 = 0.0533
Final R indexes [all data]	R_1 = 0.0288, wR_2 = 0.0548
Largest diff. peak/hole / e Å^{-3}	0.40/-0.24
Flack parameter	-0.003(5)

Table S9. Bond Lengths for [Co(NO2A)]-NHBn·4H_2O.

Atom	Atom	Length/Å	Atom	Atom	Length/Å
Co1	O1	1.9215(12)	N3	C5	1.484(2)
Co1	O3	1.9204(11)	N3	C9	1.486(2)
Co1	N1	1.9432(13)	N4	C12	1.327(2)
Co1	N2	1.9487(13)	N4	C13	1.460(2)
Co1	N3	1.9566(15)	C1	C2	1.525(2)
Co1	N4	1.9407(14)	C3	C4	1.521(2)
O1	C8	1.287(2)	C5	C6	1.523(2)

Table S9. Bond Lengths for [Co(NO2A)]-NHBn·4H_2O.

Atom	Atom	Length/Å	Atom	Atom	Length/Å
O2	C8	1.2265(19)	C7	C8	1.528(2)
O3	C10	1.290(2)	C9	C10	1.521(2)
O4	C10	1.2347(19)	C11	C12	1.516(2)
O5	C12	1.250(2)	C13	C14	1.515(2)
N1	C1	1.488(2)	C14	C15	1.397(2)
N1	C6	1.506(2)	C14	C19	1.392(2)
N1	C7	1.480(2)	C15	C16	1.396(2)
N2	C2	1.512(2)	C16	C17	1.385(3)
N2	C3	1.491(2)	C17	C18	1.392(3)
N2	C11	1.483(2)	C18	C19	1.392(3)
N3	C4	1.503(2)			

Table S10. Bond Angles for [Co(NO2A)]-NHBn·4H_2O.

Atom	Atom	Atom	Angle/˚	Atom	Atom	Atom	Angle/˚
O3	Co1	O1	92.49(5)	C9	N3	C4	111.47(12)
N1	Co1	O1	85.10(5)	C9	N3	C5	113.84(13)
N1	Co1	O3	172.40(5)	C12	N4	Co1	113.22(11)
N2	Co1	O1	173.51(5)	C13	N4	Co1	126.40(11)
N2	Co1	O3	93.98(5)	C13	N4	C12	116.54(14)
N2	Co1	N1	88.53(5)	C2	C1	N1	107.84(12)
N3	Co1	O1	92.44(5)	C1	C2	N2	110.24(13)
N3	Co1	O3	85.27(5)	C4	C3	N2	108.35(12)
N3	Co1	N1	87.62(6)	C3	C4	N3	110.35(13)
N3	Co1	N2	88.48(6)	C6	C5	N3	107.12(13)
N4	Co1	O1	94.95(6)	C5	C6	N1	110.13(14)
N4	Co1	O3	90.00(5)	C8	C7	N1	108.87(12)
N4	Co1	N1	97.39(6)	O2	C8	O1	125.02(17)
N4	Co1	N2	84.67(6)	C7	C8	O1	115.13(13)
N4	Co1	N3	171.40(6)	C7	C8	O2	119.83(15)
C8	O1	Co1	113.92(11)	C10	C9	N3	109.11(13)
C10	O3	Co1	113.75(10)	O4	C10	O3	124.71(16)
C1	N1	Co1	105.73(9)	C9	C10	O3	115.51(13)
C6	N1	Co1	109.21(10)	C9	C10	O4	119.75(15)
C6	N1	C1	111.30(13)	C12	C11	N2	110.93(12)
C7	N1	Co1	105.58(10)	N4	C12	O5	126.21(16)
C7	N1	C1	114.33(12)	C11	C12	O5	119.03(14)

Table S10. Bond Angles for [Co(NO2A)]-NHBn·4H_2O.

Atom	Atom	Atom	Angle/°	Atom	Atom	Atom	Angle/°
C7	N1	C6	110.32(12)	C11	C12	N4	114.63(14)
C2	N2	Co1	108.48(9)	C14	C13	N4	115.23(14)
C3	N2	Co1	105.54(9)	C15	C14	C13	122.07(15)
C3	N2	C2	111.27(13)	C19	C14	C13	118.94(16)
C11	N2	Co1	106.94(10)	C19	C14	C15	118.97(16)
C11	N2	C2	111.84(12)	C16	C15	C14	120.17(16)
C11	N2	C3	112.40(11)	C17	C16	C15	120.41(17)
C4	N3	Co1	108.55(9)	C18	C17	C16	119.61(16)
C5	N3	Co1	106.43(10)	C19	C18	C17	120.03(17)
C5	N3	C4	111.13(13)	C18	C19	C14	120.71(18)
C9	N3	Co1	104.98(10)				

8.5 X-ray Diffraction Analysis of $[Fe(NO2A)]^{+}$-NHBn ($[Fe(6)]ClO_4$).

8.5.1 *Data Collection*

A colorless crystal with approximate dimensions 0.16 × 0.1 × 0.02 mm^3 was selected under oil under ambient conditions and attached to the tip of a MiTeGen MicroMount©. The crystal was mounted in a stream of cold nitrogen at 100(1) K and centered in the X-ray beam by using a video camera.

The crystal evaluation and data collection were performed on a Bruker D8 VENTURE PhotonIII four-circle diffractometer with Cu Kα (λ = 1.54178 Å) radiation and the detector to crystal distance of 4.0 and 5.0 cm.[10]

The initial cell constants were obtained from a 180° φ scan conducted at a 2θ = 50° angle with the exposure time of 1 second per frame. The reflections were successfully indexed by an automated indexing routine built in the APEX6 program. The final cell constants were calculated from a set of 9495 strong reflections from the actual data collection.

The data were collected by using the full sphere data collection routine to survey the reciprocal space to the extent of a full sphere to a resolution of 0.78 Å. A total of 17386 data were harvested by collecting 15 sets of frames with 1° scans in ω and φ with an exposure time 1–8 sec per frame. These highly redundant datasets were corrected for Lorentz and polarization effects. The absorption correction was based on fitting a function to the empirical transmission surface as sampled by multiple equivalent measurements.[11]

8.5.2 *Structure Solution and Refinement*

The systematic absences in the diffraction data were uniquely consistent for the space group $P2_1/n$ that yielded chemically reasonable and computationally stable results of refinement.[12-17]

A successful solution by intrinsic phasing provided most non-hydrogen atoms from the *E*-map. The remaining non-hydrogen atoms were located in an alternating series of least-squares cycles and difference Fourier maps. All non-hydrogen atoms were refined with anisotropic displacement coefficients. All hydrogen atoms bonded to carbon atoms were included in the structure factor calculation at idealized positions and were allowed to ride on the neighboring atoms with relative isotropic displacement coefficients. Atom H4(N4) was refined with a relative atomic displacement coefficient and an N–H distance restraint.

The composition of the crystallized compound is $(C_{19}H_{26}FeN_4O_5)(ClO_4)$.

The final least-squares refinement of 310 parameters against 4573 data resulted in residuals *R* (based on F^2 for $I \geq 2\sigma$) and *wR* (based on F^2 for all data) of 0.0346 and 0.0824, respectively. The final difference Fourier map was featureless.

8.5.3 *Summary*

Crystal Data for $C_{19}H_{26}ClFeN_4O_9$ (M =545.74 g/mol): monoclinic, space group $P2_1/n$ (no. 14), a = 8.9431(9) Å, b = 20.9832(13) Å, c = 11.8336(12) Å, β = 95.570(9)°, V = 2210.1(3) $Å^3$, Z = 4, T = 100 K, μ(Cu Kα) = 7.129 mm^{-1}, $Dcalc$ = 1.640 g/cm^3, 17386 reflections measured (8.428° ≤ 2Θ ≤ 158.522°), 4573 unique (R_{int} = 0.0457, R_{sigma} = 0.0408) which were used in all calculations. The final R_1 was 0.0346 (I > 2σ(I)) and wR_2 was 0.0824 (all data).

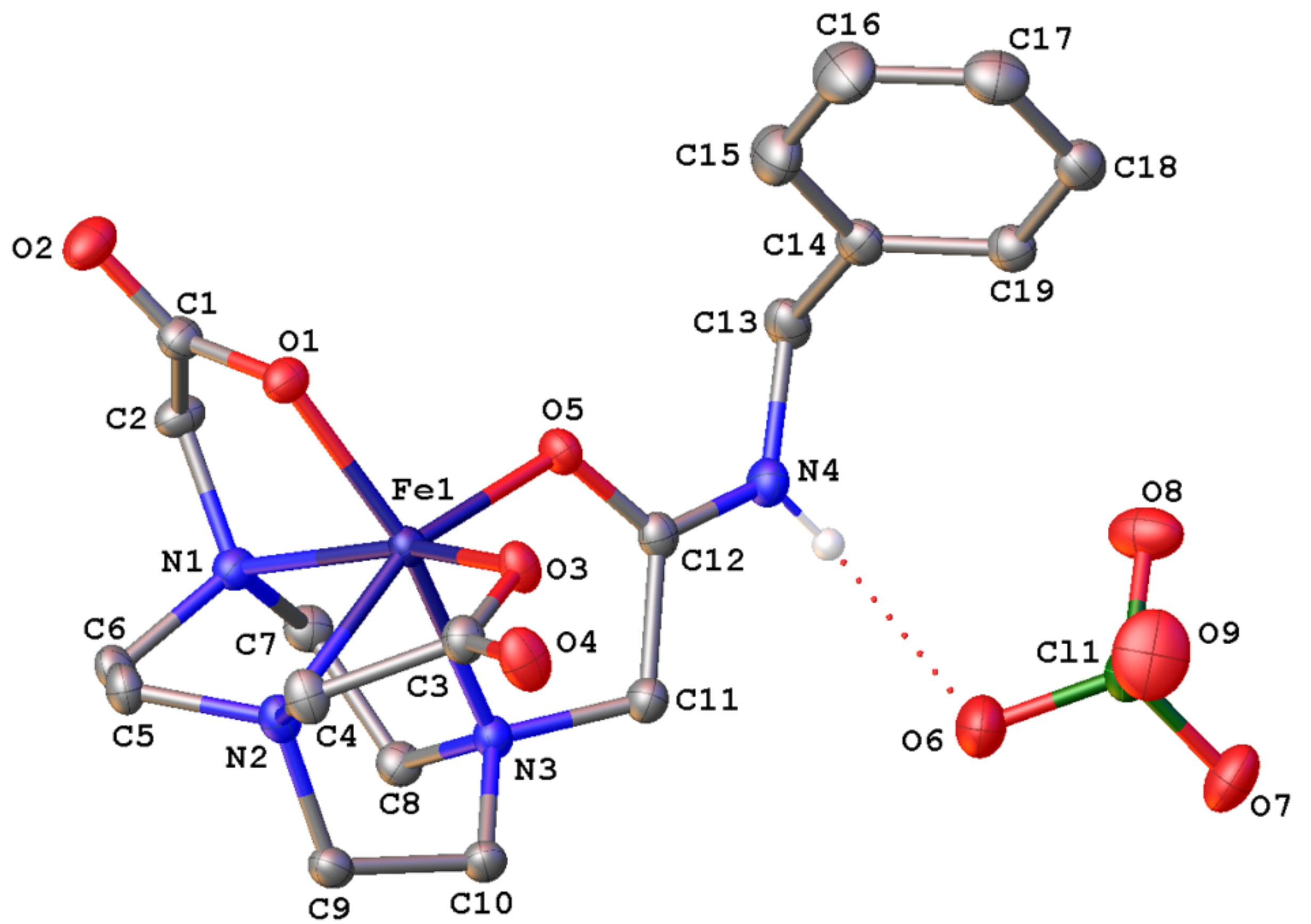

Figure S202. A molecular drawing of [Fe(6)]ClO_4 shown with 50% probability ellipsoids. All H atoms bonded to carbon atoms are omitted.

Table S11. Crystallographic details for $[Fe(NO2A)]^+$-NHBn.

Identification code	$[Fe(NO2A)]^+$-NHBn
Empirical formula	$(C_{19}H_{26}FeN_4O_5)(ClO_4)$
Formula weight	545.74
Temperature/K	100
Crystal system	monoclinic
Space group	$P2_1/n$
a/Å	8.9431(9)
b/Å	20.9832(13)
c/Å	11.8336(12)
α/°	90
β/°	95.570(9)
γ/°	90
Volume/Å^3	2210.1(3)
Z	4
ρ_{calc}g/cm^3	1.640
μ/mm^{-1}	7.129

F(000)	1132.0
Crystal size/mm^3	0.16 × 0.1 × 0.02
Radiation	Cu Kα (λ = 1.54178)
2Θ range for data collection/°	8.428 to 158.522
Index ranges	-10 ≤ h ≤ 11, -26 ≤ k ≤ 26, -15 ≤ l ≤ 13
Reflections collected	17386
Independent reflections	4573 [R_{int} = 0.0457, R_{sigma} = 0.0408]
Data/restraints/parameters	4573/1/310
Goodness-of-fit on F^2	1.030
Final R indexes [I>=2σ (I)]	R_1 = 0.0346, wR_2 = 0.0787
Final R indexes [all data]	R_1 = 0.0424, wR_2 = 0.0824
Largest diff. peak/hole / e Å^{-3}	0.34/-0.38

Table S12. Bond Lengths for $[Fe(NO2A)]^+$-NHBn.

Atom	Atom	Length/Å	Atom	Atom	Length/Å
Fe1	O1	1.9382(16)	N4	C12	1.302(3)
Fe1	O3	1.9322(15)	N4	C13	1.475(3)
Fe1	O5	1.9888(15)	C1	C2	1.524(3)
Fe1	N1	2.1559(18)	C3	C4	1.530(3)
Fe1	N2	2.1707(17)	C5	C6	1.522(3)
Fe1	N3	2.1902(18)	C7	C8	1.524(3)
O1	C1	1.308(3)	C9	C10	1.523(3)
O2	C1	1.221(3)	C11	C12	1.511(3)
O3	C3	1.305(3)	C13	C14	1.513(3)
O4	C3	1.214(3)	C14	C15	1.388(3)
O5	C12	1.279(3)	C14	C19	1.390(3)
N1	C2	1.481(3)	C15	C16	1.388(4)
N1	C6	1.500(3)	C16	C17	1.388(4)
N1	C7	1.491(3)	C17	C18	1.386(3)
N2	C4	1.476(3)	C18	C19	1.393(3)
N2	C5	1.489(3)	Cl1	O6	1.4448(19)
N2	C9	1.499(3)	Cl1	O7	1.4358(19)
N3	C8	1.504(3)	Cl1	O8	1.4462(18)
N3	C10	1.491(3)	Cl1	O9	1.4259(19)
N3	C11	1.480(3)			

Table S13. Bond Angles for $[Fe(NO2A)]^+$-NHBn.

Atom	Atom	Atom	Angle/°	Atom	Atom	Atom	Angle/°
O1	Fe1	O5	102.25(6)	C11	N3	C10	112.08(17)
O1	Fe1	N1	80.49(6)	C12	N4	C13	123.26(19)
O1	Fe1	N2	98.08(7)	O1	C1	C2	114.18(19)
O1	Fe1	N3	161.46(7)	O2	C1	O1	123.75(19)

Table S13. Bond Angles for [Fe(NO2A)]$^{+}$-NHBn.

Atom	Atom	Atom	Angle/°	Atom	Atom	Atom	Angle/°
O3	Fe1	O1	98.90(7)	O2	C1	C2	122.1(2)
O3	Fe1	O5	99.00(6)	N1	C2	C1	108.87(17)
O3	Fe1	N1	161.33(7)	O3	C3	C4	114.51(18)
O3	Fe1	N2	80.75(6)	O4	C3	O3	124.6(2)
O3	Fe1	N3	99.14(7)	O4	C3	C4	120.85(19)
O5	Fe1	N1	99.36(7)	N2	C4	C3	110.64(17)
O5	Fe1	N2	159.43(7)	N2	C5	C6	110.02(17)
O5	Fe1	N3	79.04(6)	N1	C6	C5	111.71(16)
N1	Fe1	N2	80.88(7)	N1	C7	C8	109.85(17)
N1	Fe1	N3	81.06(7)	N3	C8	C7	111.88(16)
N2	Fe1	N3	80.70(7)	N2	C9	C10	112.08(16)
C1	O1	Fe1	118.94(13)	N3	C10	C9	109.63(17)
C3	O3	Fe1	119.49(13)	N3	C11	C12	108.80(17)
C12	O5	Fe1	117.32(13)	O5	C12	N4	121.59(19)
C2	N1	Fe1	103.90(12)	O5	C12	C11	118.94(18)
C2	N1	C6	111.47(17)	N4	C12	C11	119.46(19)
C2	N1	C7	112.93(16)	N4	C13	C14	113.17(18)
C6	N1	Fe1	110.65(12)	C15	C14	C13	120.5(2)
C7	N1	Fe1	105.10(13)	C15	C14	C19	119.1(2)
C7	N1	C6	112.27(16)	C19	C14	C13	120.3(2)
C4	N2	Fe1	104.04(12)	C14	C15	C16	120.5(2)
C4	N2	C5	113.36(17)	C17	C16	C15	120.1(2)
C4	N2	C9	112.00(17)	C18	C17	C16	119.8(2)
C5	N2	Fe1	104.88(12)	C17	C18	C19	119.8(2)
C5	N2	C9	111.74(16)	C14	C19	C18	120.6(2)
C9	N2	Fe1	110.27(13)	O6	Cl1	O8	108.34(12)
C8	N3	Fe1	109.32(13)	O7	Cl1	O6	108.61(11)
C10	N3	Fe1	104.68(12)	O7	Cl1	O8	109.50(11)
C10	N3	C8	112.20(15)	O9	Cl1	O6	109.62(13)
C11	N3	Fe1	105.81(12)	O9	Cl1	O7	110.83(13)
C11	N3	C8	112.23(16)	O9	Cl1	O8	109.89(13)

8.6 X-ray Diffraction Analysis of [Fe(NO2A)-NHBn]$_2$O ([Fe(6)]$_2$O).

8.6.1 Data Collection

A brown crystal with approximate dimensions 0.132 x 0.061 x 0.035 mm^3 was selected under oil under ambient conditions and attached to the tip of a MiTeGen MicroMount©. The crystal was mounted in a stream of cold nitrogen at 100(1) K and centered in the X-ray beam by using a video camera.

The crystal evaluation and data collection were performed on a Bruker D8 VENTURE Photon III four-circle diffractometer with Diamond II IµS Mo Kα (λ = 0.71073 Å) radiation and the detector to crystal distance of 5.0 cm.[10]

The initial cell constants were obtained from a 180° φ scan conducted at a 2θ = 50° angle with the exposure time of 1 second per frame. The reflections were successfully indexed by an automated indexing routine built in the APEX6 program. The final cell constants were calculated from a set of 9920 strong reflections from the actual data collection.

The data were collected by using the full sphere data collection routine to survey the reciprocal space to the extent of a full sphere to a resolution of 0.77 Å. A total of 66388 data were harvested by collecting 11 sets of frames with 0.5º scans in ω and φ with an exposure time 1–40.5 sec per frame. These highly redundant datasets were corrected for Lorentz and polarization effects. The absorption correction was based on fitting a function to the empirical transmission surface as sampled by multiple equivalent measurements.[11]

8.6.2 Structure Solution and Refinement

The systematic absences in the diffraction data were uniquely consistent for the space group $P2_1/c$ that yielded chemically reasonable and computationally stable results of refinement.[12-13]

A successful solution by intrinsic phasing provided most non-hydrogen atoms from the *E*-map. The remaining non-hydrogen atoms were located in an alternating series of least-squares cycles and difference Fourier maps. The atomic structure factors were determined by DFT calculations, using the r2SCAN functional and the def2-TZVPP basis set, in the NoSpherA2 extension of the olex2.refine program.[18-20] The H atoms bonded to C13A–C19A atoms were included in the structure factor calculation at idealized positions and were allowed to ride on the neighboring atoms with relative isotropic atomic displacement coefficients. All other hydrogen atoms were refined with restrained bond distances and isotropic atomic displacement coefficients.

The compound has an overall chemical formula of $(FeC_{14}H_{26}N_4O_5)_2O$ The O1 atom resides on a crystallographic inversion center, thus only one-half of the molecule is symmetry independent.

The only crystal large enough for the single-crystal X-ray diffraction experiment proved to be a non-merohedral twin with an approximate 3.5(2) % second component contribution. The twin components are related by a 179.9° rotation about the *b* axis. The data was treated as single, and only reflections from the first twin domain were included in the data refinement.

The C13 tolyl group is positionally disordered with a dominant component occupation of 86.2(6) %. The minor component of the disorder is refined with restrained geometries and both components of the disorder are refined with restrained anisotropic atomic displacement parameters

The final least-squares refinement of 434 parameters against 4613 data resulted in residuals R (based on F^2 for $I \geq 2\sigma$) and wR (based on F^2 for all data) of 0.0437 and 0.0950, respectively. The final difference Fourier map was featureless.

8.6.3 Summary

Crystal Data for $C_{38}H_{52}Fe_2N_8O_{11}$ (*M* =908.574 g/mol): monoclinic, space group $P2_1/c$ (no. 14), a = 10.465(3) Å, b = 16.988(5) Å, c = 11.435(3) Å, β = 98.283(6)°, V = 2011.7(10) Å^3, Z = 2, T = 100.00 K, μ(Mo Kα) = 0.791 mm^{-1}, *Dcalc* = 1.500 g/cm^3, 65217 reflections measured (3.94° ≤ 2Θ ≤ 54.98°), 4613 unique (R_{int} = 0.0419, R_{sigma} = 0.0189) which were used in all calculations. The final R_1 was 0.0437 (I>=2u(I)) and wR_2 was 0.0950 (all data).

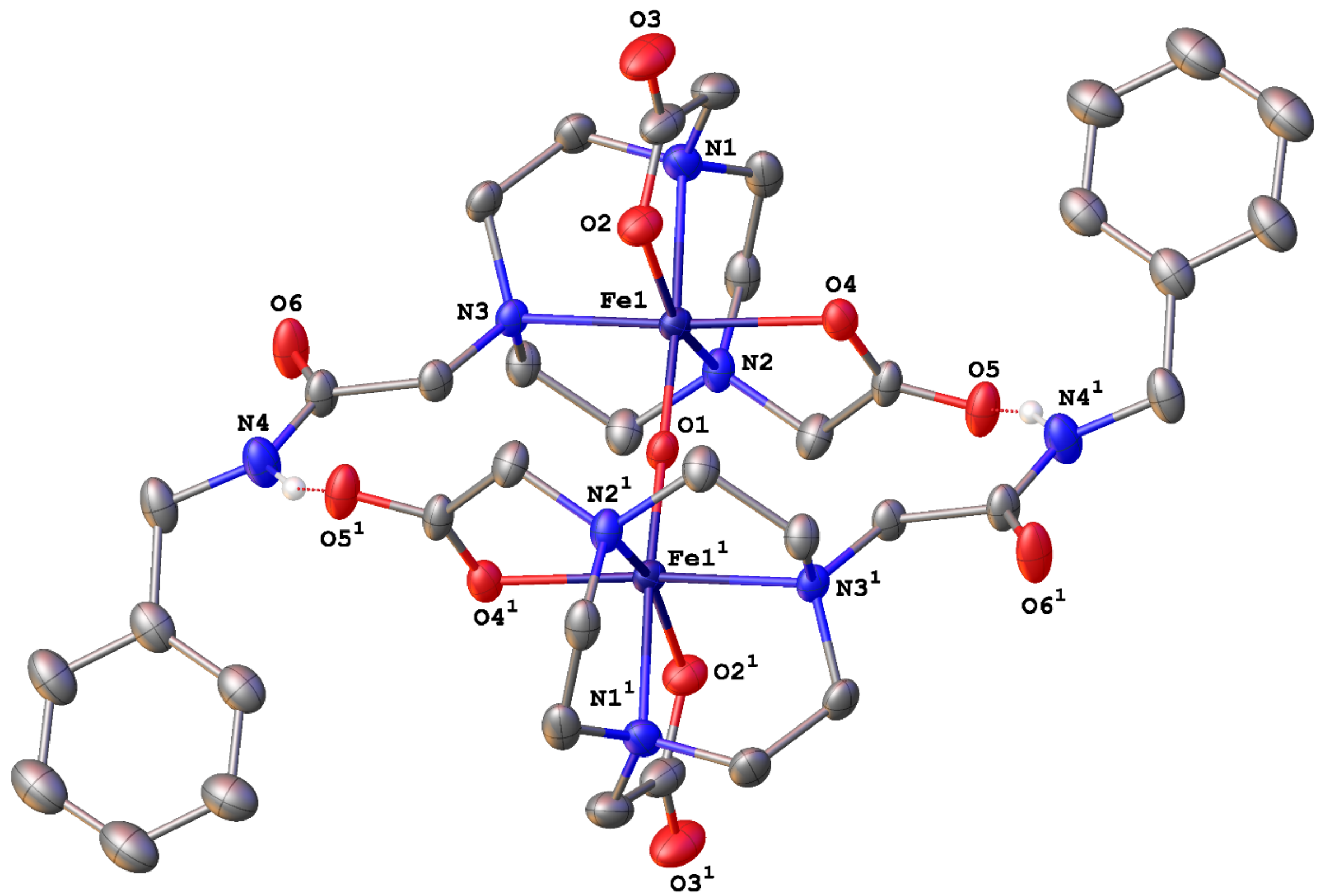

Figure S203. A molecular drawing of [Fe(6)]$_2$O. The figure is drawn with 50% probability ellipsoids, and all H atoms bonded to C atoms and the minor components of the C13 tolyl group disorder are omitted. Symmetry code: (1) 1-X, 1-Y, 1-Z.

Table S14. Crystallographic details for [Fe(NO2A)-NHBn]$_2$O.

Identification code	[Fe(NO2A)-NHBn]$_2$O
Empirical formula	$(FeC_{14}H_{26}N_4O_5)_2O$
Formula weight	908.574
Temperature/K	100.00
Crystal system	monoclinic
Space group	$P2_1/c$
a/Å	10.465(3)
b/Å	16.988(5)

c/Å	11.435(3)
α/°	90
β/°	98.283(6)
γ/°	90
Volume/Å^3	2011.7(10)
Z	2
ρ_{calc}g/cm^3	1.500
μ/mm^{-1}	0.791
F(000)	954.1
Crystal size/mm^3	0.132 × 0.061 × 0.035
Radiation	Mo Kα (λ = 0.71073)
2Θ range for data collection/°	3.94 to 54.98
Index ranges	-13 ≤ h ≤ 13, -22 ≤ k ≤ 22, -11 ≤ l ≤ 14
Reflections collected	65217
Independent reflections	4613 [R_{int} = 0.0419, R_{sigma} = 0.0189]
Data/restraints/parameters	4613/340/434
Goodness-of-fit on F^2	1.064
Final R indexes [I>=2σ (I)]	R_1 = 0.0437, wR_2 = 0.0918
Final R indexes [all data]	R_1 = 0.0508, wR_2 = 0.0950
Largest diff. peak/hole / e Å^{-3}	0.64/-0.39

Table S15. Bond Lengths for [Fe(NO2A)-NHBn]$_2$O.

Atom	Atom	Length/Å	Atom	Atom	Length/Å
Fe1	O1	1.7974(4)	N4	C13A	1.470(9)
Fe1	O2	1.9674(19)	C1	C2	1.532(5)
Fe1	O4	2.0446(18)	C3	C4	1.519(4)
Fe1	N1	2.263(2)	C5	C6	1.519(4)
Fe1	N2	2.183(2)	C7	C8	1.517(4)
Fe1	N3	2.276(2)	C9	C10	1.525(4)
O2	C1	1.300(3)	C11	C12	1.537(3)
O3	C1	1.222(3)	C13	C14	1.547(6)
O4	C6	1.285(3)	C14	C15	1.405(6)
O5	C6	1.235(3)	C14	C19	1.396(5)
O6	C12	1.227(3)	C15	C16	1.392(5)

Table S15. Bond Lengths for [Fe(NO2A)-NHBn]$_2$O.

Atom	Atom	Length/Å	Atom	Atom	Length/Å
N1	C2	1.457(4)	C16	C17	1.399(6)
N1	C3	1.481(4)	C17	C18	1.398(7)
N1	C10	1.491(3)	C18	C19	1.401(6)
N2	C4	1.491(3)	C13A	C14A	1.526(17)
N2	C5	1.465(3)	C14A	C15A	1.398(16)
N2	C7	1.484(3)	C14A	C19A	1.383(17)
N3	C8	1.498(3)	C15A	C16A	1.385(16)
N3	C9	1.488(3)	C16A	C17A	1.392(17)
N3	C11	1.467(3)	C17A	C18A	1.413(17)
N4	C12	1.341(3)	C18A	C19A	1.395(17)
N4	C13	1.446(4)			

Table S16. Bond Angles for [Fe(NO2A)-NHBn]$_2$O.

Atom	Atom	Atom	Angle/°	Atom	Atom	Atom	Angle/°
O2	Fe1	O1	99.48(6)	C13A	N4	C13	20.8(13)
O4	Fe1	O1	96.40(5)	O3	C1	O2	124.4(3)
O4	Fe1	O2	101.61(8)	C2	C1	O2	115.5(2)
N1	Fe1	O1	176.61(6)	C2	C1	O3	120.1(3)
N1	Fe1	O2	78.51(8)	C1	C2	N1	110.7(2)
N1	Fe1	O4	86.70(7)	C4	C3	N1	110.0(2)
N2	Fe1	O1	103.98(6)	C3	C4	N2	112.5(2)
N2	Fe1	O2	156.52(8)	C6	C5	N2	109.2(2)
N2	Fe1	O4	76.87(7)	O5	C6	O4	124.8(3)
N2	Fe1	N1	78.01(8)	C5	C6	O4	116.0(2)
N3	Fe1	O1	99.43(5)	C5	C6	O5	119.0(2)
N3	Fe1	O2	96.40(8)	C8	C7	N2	109.8(2)
N3	Fe1	O4	153.66(7)	C7	C8	N3	111.4(2)
N3	Fe1	N1	78.17(7)	C10	C9	N3	111.5(2)
N3	Fe1	N2	78.95(7)	C9	C10	N1	112.7(2)
Fe1[1]	O1	Fe1	180.0	C12	C11	N3	116.7(2)
C1	O2	Fe1	119.36(19)	N4	C12	O6	124.2(2)
C6	O4	Fe1	115.29(16)	C11	C12	O6	123.6(2)

Table S16. Bond Angles for [Fe(NO2A)-NHBn]$_2$O.

Atom	Atom	Atom	Angle/°	Atom	Atom	Atom	Angle/°
C2	N1	Fe1	102.36(16)	C11	C12	N4	112.2(2)
C3	N1	Fe1	106.00(16)	C14	C13	N4	110.6(3)
C3	N1	C2	113.8(2)	C15	C14	C13	123.6(3)
C10	N1	Fe1	110.84(16)	C19	C14	C13	118.0(4)
C10	N1	C2	111.9(2)	C19	C14	C15	118.3(4)
C10	N1	C3	111.4(2)	C16	C15	C14	120.8(4)
C4	N2	Fe1	113.03(17)	C17	C16	C15	120.1(4)
C5	N2	Fe1	102.61(15)	C18	C17	C16	120.0(4)
C5	N2	C4	111.1(2)	C19	C18	C17	119.2(4)
C7	N2	Fe1	104.25(15)	C18	C19	C14	121.6(4)
C7	N2	C4	112.1(2)	C14A	C13A	N4	114.1(17)
C7	N2	C5	113.3(2)	C15A	C14A	C13A	108(2)
C8	N3	Fe1	108.44(14)	C19A	C14A	C13A	127(2)
C9	N3	Fe1	104.02(14)	C19A	C14A	C15A	121.4(18)
C9	N3	C8	113.35(19)	C16A	C15A	C14A	122.3(18)
C11	N3	Fe1	108.45(14)	C17A	C16A	C15A	116.8(18)
C11	N3	C8	110.6(2)	C18A	C17A	C16A	118.8(19)
C11	N3	C9	111.59(19)	C19A	C18A	C17A	123.2(19)
C13	N4	C12	125.1(3)	C18A	C19A	C14A	115.5(18)
C13A	N4	C12	107.7(11)				

[1]1-X,1-Y,1-Z

8.7 Acknowledgement

This supporting information is the result of funding in part by the National Institutes of Health (NIH, NIBIB, R01EB032349). It is subject to the NIH Public Access Policy. Through acceptance of this federal funding, NIH has been given a right to make this manuscript publicly available in PubMed Central upon the Official Date of Publication, as defined by NIH. The following instrumentation in the Paul Bender Chemical Instrumentation Center at the University of Wisconsin-Madison was supported by NSF CHE-2017891 (Bruker Avance Neo 500); by a generous gift from Paul J. and Margaret M. Bender (Bruker 500); NIH S10 OD012245 (Bruker Avance-600). The Bruker D8 VENTURE Photon III X-ray diffractometer was partially funded by NSF Award #CHE 1919350 to the UW-Madison Department of Chemistry and the Electrospray Ionization-Quadrupole-Ion Trap Mass Spectrometer was funded by NIH 1S10 OD020022. The purchase of the Bruker D8 VENTURE Photon III Diamond II IμS X-ray diffractometer was partially funded by a 2024 University of Wisconsin Research Core Revitalization Program Round 3 Award to the Department of Chemistry.

9 References

(1) Zhong, Z.; Śmiłowicz, D.; Gork, M. J.; Garman, L. C.; Guzei, I. A.; Boros, E. Rate-Tunable, Metal-Mediated Amide Bond Cleavage for the Controlled Release of Pharmaceuticals. *J. Am. Chem. Soc.* **2025,** *147*(46), 42491-42499.
(2) Shetty, D.; Choi, S. Y.; Jeong, J. M.; Hoigebazar, L.; Lee, Y. S.; Lee, D. S.; Chung, J. K.; Lee, M. C.; Chung, Y. K. Formation and Characterization of Gallium (III) Complexes with Monoamide Derivatives of 1, 4, 7-Triazacyclononane-1, 4, 7-triacetic Acid: A Study of the Dependency of Structure on Reaction pH. *Eur. J. Inorg. Chem*. **2010**, *2010*(34), 5432– 5438.
(3) Kelderman, C. A. A.; Glaser, O. M.; Whetter, J. N.; Aluicio-Sarduy, E.; Mixdorf, J. C.; Sanders, K., M.; Guzei, I. A.; Barnhart, T. E.; Engle, J. W.; Boros; E. Charting the coordinative landscape of the ^{18}F–Sc/^{44}Sc/^{177}Lu triad with the tri-aza-cyclononane (tacn) scaffold. *Chem. Sci*. **2024**, *15* (43): 17927–17936.
(4) Gans, P.; Sabatini, A.; Vacca, A. Investigation of Equilibria in Solution. Determination of Equilibrium Constants with the HYPERQUAD Suite of Programs. *Talanta*. **1996**, *43*(10), 1739−1753.
(5) Alderighi, L.; Gans, P.; Ienco, A.; Peters, D.; Sabatini, A.; Vacca, A. Hyperquad Simulation and Speciation (HySS): A Utility Program for the Investigation of Equilibria Involving Soluble and Partially Soluble Species. *Coord. Chem. Rev*. **1999**, *184*, 311−318.
(6) Kularatne, S. A.; Zhou, Z.; Yang, J.; Post, C. B.; Low, P. S. Design, Synthesis, and Preclinical Evaluation of Prostate-Specific Membrane Antigen Targeted ^{99m}Tc-Radioimaging Agents. *Mol. Pharmaceutics.* **2009**, *6*(3), 790–800.
(7) Bassissi, M. F.; Blasco Ferrer, M.; Pérez Ferrer, M. d. M., Ferrer Reynés, M. D.; Salcedo Roca, C.; Gold, A. M.; Perelló Bestard, J. Ip4-4,6 substituted derivative compounds for use in the treatment, inhibition of progression, and prevention of ectopic calcification (International Publication No. WO 2024/023359 A1). World Intellectual Property Organization. [WIPO Patentscope] (wipo.int).
(8) Maresca, K. P.; Hillier, S. M.; Femia, F. J.; Keith. D.; Barone, C.; Joyal. J. L.; Zimmerman C. N.; Kozikowski, A. P.; Barrett, J. A.; Eckelman. W. C.; Babich, J. W. A Series of Halogenated Heterodimeric Inhibitors of Prostate Specific Membrane Antigen (PSMA) as Radiolabeled Probes for Targeting Prostate Cancer. *J. Med. Chem.* **2009**, *52*(2), 347 – 357.
(9) Ahn, S. H.; Iuliano, J. N.; Boros, E. Trivalent metal complex geometry of the substrate governs cathepsin B enzymatic cleavage rate. *Chem. Commun.* **2020**, *56*(53), 7289–7292.
(10) Bruker AXS LLC (2024). APEX6. Version 2024.9-0. Madison, Wisconsin, USA.
(11) Krause, L., Herbst-Irmer, R., Sheldrick, G. M. & Stalke, D. (2015). *J. Appl. Cryst.* 48. 3-10.
(12) Sheldrick, G. M. (2013b). *XPREP*. Version 2013/1. Georg-August-Universität Göttingen, Göttingen, Germany.
(13) Sheldrick, G. M. (2013a). The *SHELX* homepage, http://shelx.uni-ac.gwdg.de/SHELX/.
(14) Sheldrick, G. M. (2015a). *Acta Cryst.* A, 71, 3-8.
(15) Sheldrick, G. M. (2015b). *Acta Cryst.* C, 71, 3-8.
(16) Dolomanov, O. V., Bourhis, L. J., Gildea, R. J., Howard, J. A. K. & Puschmann, H. (2009). *J. Appl. Crystallogr.* 42, 339-341.
(17) Guzei, I. A. (2007-2022). Programs *Gn.* University of Wisconsin-Madison, Madison, Wisconsin, USA.
(18) Bourhis, L.J., Dolomanov, O.V., Gildea, R.J., Howard, J.A.K., Puschmann, H. (2015). *Acta Cryst. A*. 71, 59-75.
(19) Neese, F. (2018). *Wiley Interdiscip. Rev.: Comput. Mol. Sci*. 8, e1327.

(20) Kleemiss, F., Dolomanov, O. V., Bodensteiner, M., Peyerimhoff, N., Midgley, L., Bourhis, L. J., Genoni, A., Malaspina, L. A., Jayatilaka, D., Spencer, J. L., White, F., Grundkötter-Stock, B., Steinhauer, S., Lentz, D., Puschmann, H., & Grabowsky, S. (2021). *Chem. Sci.* 12, 1675-1692.
